# A metagenomic survey of forest soil microbial communities more than a decade after timber harvesting

**DOI:** 10.1038/sdata.2017.92

**Published:** 2017-07-25

**Authors:** Roland C. Wilhelm, Erick Cardenas, Hilary Leung, Kendra Maas, Martin Hartmann, Aria Hahn, Steven Hallam, William W. Mohn

**Affiliations:** 1 Department of Microbiology and Immunology, Life Sciences Institute, University of British Columbia, Vancouver, British Columbia, Canada V6T 1Z3; 2 Swiss Federal Institute for Forest, Snow and Landscape Research, Birmensdorf CH-8903, Switzerland

**Keywords:** Soil microbiology, Molecular ecology, Forest ecology, Metagenomics

## Abstract

The scarcity of long-term data on soil microbial communities in the decades following timber harvesting limits current understanding of the ecological problems associated with maintaining the productivity of managed forests. The high complexity of soil communities and the heterogeneity of forest and soil necessitates a comprehensive approach to understand the role of microbial processes in managed forest ecosystems. Here, we describe a curated collection of well replicated, multi-faceted data from eighteen reforested sites in six different North American ecozones within the Long-term Soil Productivity (LTSP) Study, without detailed analysis of results or discussion. The experiments were designed to contrast microbial community composition and function among forest soils from harvested treatment plots with varying intensities of organic matter removal. The collection includes 724 bacterial (16S) and 658 fungal (ITS2) amplicon libraries, 133 shotgun metagenomic libraries as well as stable isotope probing amplicon libraries capturing the effects of harvesting on hemicellulolytic and cellulolytic populations. This collection serves as a foundation for the LTSP Study and other studies of the ecology of forest soil and forest disturbance.

## Background & Summary

The Long-term Soil Productivity Study (LTSP) was initiated in 1989 to track changes in soil quality and productivity of managed forests. Foresters lacked data on the impact of growing trends in intensified forest management, such as shorter crop cycles, greater biomass extraction, and use of highly mechanized equipment^1^. Partners in the LTSP Study have since collected longitudinal data from a range of North America’s most-productive forests (>100 sites) to assess the long-term effects of soil compaction and organic matter (OM) removal. The objective is to develop indices for monitoring soil quality based in physical, chemical, and biological properties of soil. Each LTSP site has replicated experimental plots for harvested treatments with three intensities of OM removal as well as unharvested reference plots. OM removal intensity corresponded to common harvesting strategies, such as debranching *in situ* (OM1) or removal of both trunks and branches (OM2), or the less common, and extreme, practice of removing trunks, branches and top soil (OM3). In the ensuing years, these treatments produced a consistent gradient in soil properties according to harvesting intensity, such as decreasing amounts of total carbon and nitrogen as well as increased dryness, mean daily temperature and temperature fluctuation. While the latest LTSP studies show that the effects of varying degrees of OM removal appear minor on net primary productivity^2,3,4^, the metagenomic datasets presented here show consistent changes in bacterial and fungal populations that reflected harvesting intensity^5,6,7,8,9^.

**Figure 1:**
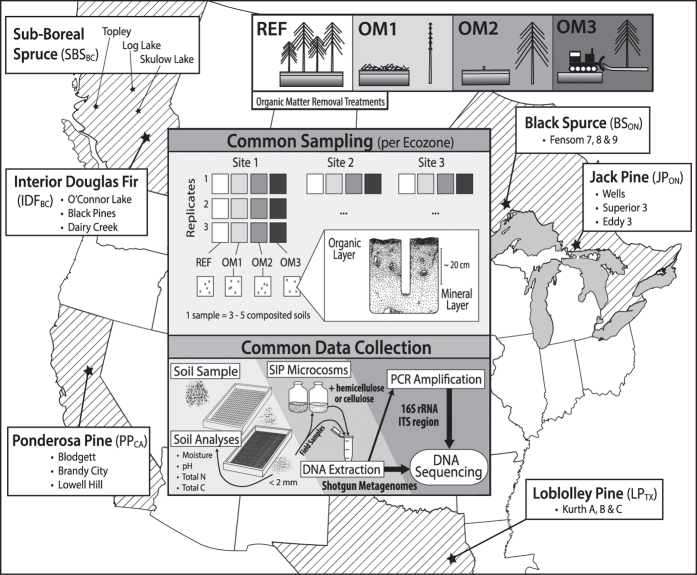
Locations of soil sampling and descriptions of data collection conducted for this study. The locations and names of eighteen North American sampling sites are shown grouped into six ecozones. The term ‘ecozone’ is used to refer to the distinct local assemblages of organisms and climatic factors between groupings of sites. An overview of the design for soil sampling along with data collection are superimposed on the map. OM removal treatments are shaded according to the intensity of OM removal.

We collected data on soil microbial communities from all treatment plots at eighteen LTSP sites in six different ecozones between 2008 and 2014 when reforested stands were 11 to 17 years old (Fig. 1; Table 1). To capture the extent of harvesting impacts throughout the soil profile, corresponding samples from organic and mineral layers were included in all datasets. The goal was to identify changes in community composition, diversity, and functional potential resulting from the intensity of OM removal. We surveyed soil microbial communities in treatment plots using amplicon sequencing (Table 2 (available online only)) of bacteria (Data Citation 1) and fungi (Data Citation 2), along with shotgun metagenomes from all treatment plots at a single site within each ecozone (Data Citation 3; Table 3 (available online only)). Shotgun metagenomes revealed impacts on the functional potential of communities, such as decomposers involved in carbon cycling^5^, which led to further targeted studies of hemicellulolytic^7^ (Data Citations 4,5) and cellulolytic^8^ populations (Data Citation 6) using ^13^C-stable isotope probing (Table 4 (available online only)). Stable isotope probing (SIP) can be used to track populations that assimilate ^13^C-label into their biomass by recovering and sequencing the resultant ‘heavier’^13^C-enriched DNA^10^.

Here, we provide an overview of the data collection, without detailed analysis of results or discussion, to draw attention to its unparalleled comprehensive and multi-faceted view into forest soil microbial communities. The collection is the first high-throughput sequencing-based survey of LTSP sites, and, as such, provides base-line data for future LTSP investigations at an important, stage of forest regeneration just prior to canopy closure. Researchers can revisit this collection as our understanding of the ecology of forest soils advances. This will be important given the substantial proportion of unknown taxa in these collections affected by timber harvesting^9^. SIP data offers unique insights into the effects of timber harvesting on decomposer populations, including detailed information on uncultured taxa provided by the ten partial genomes recovered from SIP-cellulose shotgun metagenomes (Data Citation 6). The consistency in experimental design, sequencing methodology and sample sources ensures the value of this collection for on-going studies of forest soil microbial communities, in particular those pertaining to biogeography, soil strata and forest disturbance.

## Methods

### Experimental design

**Figure 2:**
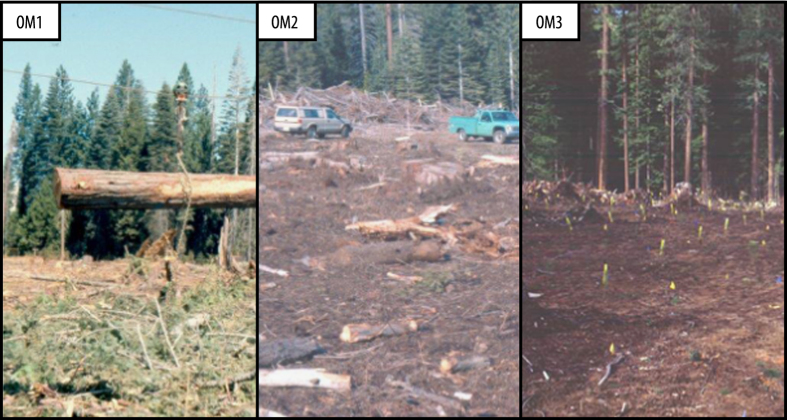
Plot conditions immediately following harvesting (year zero) in the PP_CA_ ecozone. These photographs capture the initial variation in the amount of organic matter (OM) removal at plots which were sampled 11–17 years for this study. Plots like these were replicated at three sites within every ecozone [photo credit: Dr Matt Busse; mbusse@fs.fed.us

Soil samples were collected from reforested experimental plots within the Long-Term Soil Productivity (LTSP) Study from eighteen different sites across six conifer-dominated North American ecozones named after the predominant tree species (Fig. 1; Table 1): IDF_BC_ (interior Douglas-fir), SBS_BC_ (sub-boreal spruce), PP_CA_ (ponderosa pine), BS_ON_ (black spruce), JP_ON_ (jack pine) and LP_TX_ (loblolly pine). Ecozones were chosen to exemplify a broad range of climates and regions in North America where forestry is a major industry. These differed by several factors, including soil type, mean annual temperature and precipitation, tree species and bulk soil chemistry, such as carbon and nitrogen content and pH (Table 1). Each ecozone contained three sites with four treatments: REF (or OM0), a neighbouring unharvested reference plot; and three harvested treatments: OM1, where only tree boles (stems) were removed and woody debris was left in place; OM2, where whole trees including branches were removed and; OM3, where whole trees were removed and the upper organic layer of forest floor scraped away (Fig. 2). Compaction was controlled and, in all cases, plots with minimum compaction (‘C0’) were sampled. Moderate (C1) and severe (C2) compaction treatments were included in 16S rRNA gene and ITS pyrotag libraries in SBS_BC_ and IDF_BC_ (Table 2 (available online only)). Similarly, additional amplicon libraries were prepared from soils in JP_ON_ and LP_TX_ which had been exposed to glyphosate (Table 2 (available online only)). Samples were collected from triplicate plots at each of the three sites in BS_ON_ and JP_ON_, while at sites in the other four ecozones triplicate samples were collected from a single, larger plot. Each sample corresponds to a composite from between three to five sampling points (consistent within a given ecozone) in a plot which helped account for soil heterogeneity. Organic layer samples (O-horizons) were first sampled with a trowel and then the top 20 cm of mineral layer soil (A and upper B-horizon) were collected using a Stoney auger (5 cm diameter). For several 16S rRNA gene amplicon and whole shotgun sequencing libraries from Skulow Lake (denoted in Tables 2 and 3 (available online only)), samples were collected from five soil horizons: LFH and mineral horizons (Ahe, Ae, AB and Bt), distinguished using criteria from the Canadian System of Soil Classification. Samples were stored at 4 °C during transport and until each sample was sieved through 2-mm mesh to remove roots, then stored at −80 °C until DNA was extracted within three months of sampling. Soil samples used in metatranscriptomics were flash frozen in liquid nitrogen, transported on dry ice and subsequently stored at −80 °C until DNA and RNA was extracted within 12 months of sampling.

### Amplicon and shotgun metagenome sequencing

DNA was extracted from field samples (0.5 g of soil) using the manufacturer’s recommended protocol for the FastDNA Spin Kit for Soil (MPBio, Santa Ana, CA). Amplicon libraries were prepared for the 16S rRNA gene (V1-V3 regions) and fungal internal transcribed spacer region (ITS2) according to the procedure of Hartmann *et al.*^5^. The region spanning V1–V3 was amplified using barcoded universal primers: 27F (5′- AGA GTT TGA TCM TGG CTC AG–3′) and 519R (5′- GWA TTA CCG CGG CKG CTG–3′)^11,12^ and the fungal internal transcribed spacer (ITS2) region was amplified using barcoded primers: ITS3 (5′- GCA TCG ATG AAG AAC GCA GC–3′) and ITS4 (5′- TCC TCC GCT TAT TGA TAT GC–3′)^13^. Amplicons were generated via polymerase chain reaction (PCR) in triplicate for each sample and pooled prior to purification and quantification. DNA quantitation was performed using Pico-Green fluorescent dye (ThermoFisher, MA, USA). Samples were sequenced using the Roche 454 Titanium platform at the McGill University and at the Genome Québec Innovation Centre with a maximum of 40 samples multiplexed on each quarter plate. Table 2 (available online only) contains information on all amplicon libraries created from field soil samples, including 16S rRNA gene amplicon (Data Citation 1) and ITS amplicon libraries (Data Citation 2). This includes a second set of samples from Skulow Lake with a narrower focus on five soil depths in only REF and OM3 (Table 2 (available online only)). These amplicon libraries were made from primers targeting the V6-V8 region of the 16S rRNA gene and were amplified according to Gies *et al.*^14^ using barcoded universal primers: 926F (5′- CC TAT CCC CTG TGT GCC TTG GCA GTC TCA GAA ACT YAA AKG AAT TGR CGG-3′) and 1392R (5′‐ CGT ATC GCC TCC CTC GCG CCA TCA GAC GGG CGG TGT GTR C-3′). PCR product was pooled, purified, quantified and sequenced as for other libraries.

Whole shotgun metagenomes were generated for a single site in each of the six ecozones resulting in three replicates for each treatment and each soil horizon for each ecozone. At Skulow Lake (SBS_BC_), sampling for metagenomes focused on changes along the soil profile and, thus, did not cover all four OM harvesting treatments, only REF versus OM3. Unlike in pyrotag libraries, a second complete set of shotgun metagenomes from Skulow Lake does not exist. Insufficient organic layer soil was available from OM3 to prepare shotgun metagenomes for BS_ON_, IDF_BC_, JP_ON_, and PP_CA_. The same soil samples were used for shotgun and amplicon metagenomes, but from separate DNA extractions. After quantification, triplicate samples were multiplexed in each Illumina HiSeq lane for sequencing. Samples from ecozones BS_ON_, JP_ON_, PP_CA_ and LP_TX_ were sequenced at the US DOE Joint Genome Institute (Walnut Creek, CA) producing paired-end, 150-bp Illumina libraries while samples for the IDF_BC_ and SBS_BC_ ecozones were sequenced at the Michael Smith Genome Sciences Centre (Vancouver, Canada) resulting in paired-end, 75-bp and 100-bp Illumina libraries, respectively.

### Stable isotope probing amplicon and shotgun metagenome sequencing

Microcosms were prepared by adding between 0.75 and 2.0 g of 2 mm sieved organic or mineral layer soil to 30-mL serum vials. Larger quantities of mineral soil were necessary because of lower microbial biomass, requiring two DNA extractions per mineral soil to obtain the necessary 5 μg of DNA. Moisture content was standardized to 60% w/v for mineral soil, due to lower absorptive capacity, and 125% w/v for organic soil and pre-incubating at 20 °C for one week. Microcosms were then amended with either 1.0% w/w of ^13^C-labeled hemicellulose (97 atom %; IsoLife; U-10509, Lot: 0901-0273) or custom prepared bacterial cellulose (99 atom % ^13^C). Each soil sample was paired with a microcosm containing the same amount of unlabelled (~1.1 atom % ^13^C) substrate. Separation between ^13^C-enriched DNA and unlabelled DNA is never complete, in part, due to variation in GC content^15^. Thus, ^13^C-libraries are always compared to identically prepared sequencing libraries from samples amended with unlabelled substrate. Following either 2-day (hemicellulose), 11-day (cellulose—organic layer) or 14-day (cellulose—mineral layer) incubations, soil was lyophilized and stored at −80 °C until DNA was extracted as previously described. DNA extracts from replicates within each site were pooled in equal amounts and unlabelled controls were processed identically. Harvested treatment OM2 was not included in SIP-hemicellulose libraries.

High purity ^13^C-labled cellulose was necessary to ensure that only organisms possessing the necessary metabolic capability could assimilate the ^13^C. Bacterial cellulose was utilized due to irremediable impurity in plant-derived cellulose from IsoLife (58% glucose+4.4% lignin+remainder of sugars from hemicellulose). Bacterial cellulose was produced by cultivating *Gluconacetobacter xylinus* str. KCCM 10,100 with either ^13^C-labeled glucose (99 atom % ^13^C, Cambridge Isotope Laboratories, MA, USA), or unlabelled glucose, as sole carbon source in Yamanaka medium^16^. Cellulose pellicules were purified by boiling in sodium hydroxide per previously described methods^17^, with the addition of a second boiling step and an increase in boiling time to 4 h. While IsoLife hemicellulose was also impure (53% hemicellulose sugars), the sources of impurity were more recalcitrant forms of carbon, such as cellulose and lignin, which were not substantially degraded during the 2-day incubation.

^13^C-enriched DNA was separated and recovered using cesium chloride density gradient ultracentrifugation^10^. The atom % ^13^C was measured before and after density separation using UHPLC-MS/MS^18^. Amplicon libraries were prepared and sequenced from ^13^C-enriched DNA for SIP-hemicellulose (Data Citations 4,5) and SIP-cellulose (Data Citation 6) experiments targeting both bacterial and fungal phylogenetic markers as previously described (Table 4 (available online only)). Four shotgun metagenome libraries were generated from ^13^C-enriched DNA from SIP-cellulose incubations for REF, OM1 and OM3 treatments and one unlabelled control sample (REF) from PP_CA_. Sufficient DNA for metagenome library preparation was achieved by pooling the corresponding DNA extracts from mineral layer samples at all three sites in PP_CA_. Shotgun metagenomes were prepared from 40–50 ng of enriched DNA using the Nextera DNA Sample Preparation Kit (Illumina Inc., CA, USA) and were multiplexed on two lanes of Illumina HiSeq (2×100-bp), yielding 285 million paired-end reads (Data Citation 6). There was insufficient ^13^C-enriched DNA to generate metagenomes from the organic layer samples. Raw sequences were quality-controlled, assembled and binned into partial genomes (Table 4 (available online only); Data Citation 6) according to methods described in Wilhelm *et al.*^8^.

## Data Records

The raw pyrosequencing output (~400-bp reads) for all 16S rRNA gene (*n*=724 samples) and ITS (*n*=658 samples) amplicon libraries that were generated from field soil samples averaged 8,900 and 8,400 reads per library (post-QC), respectively, and are archived at the European Sequencing Archive (Table 2 (available online only)). The raw SFF files for 16S rRNA amplicon libraries can be found with the study accession PRJEB8599 (Data Citation 1), except for all libraries from British Columbia which were archived in study accession PRJEB12501 in ‘fastq’ format (Data Citation 2). The latter contains the entire collection of ITS amplicon libraries in ‘fastq’ format. All libraries were extracted from raw SFF files using the mothur command ‘sffinfo’ and either uploaded in standard flowgram format (SFF) or converted to ‘fastq’ format^19^ using ‘sffinfo’ to produce paired ‘fasta’ and ‘qual’ files, which were merged into ‘fastq’ format using ‘PairedFastaQualIterator’ from the SeqIO module in BioPython^20^.

Raw shotgun metagenomic data for all ecozones can be downloaded in ‘fastq’ format with the study accession PRJEB8420 from the European Nucleotide Archive (Table 3 (available online only), Data Citation 3). After our quality filtering, libraries had a median count of 59.3 million sequences for 150-bp read libraries, while shorter read libraries (75-bp) had higher median counts (115 million). These numbers are provided as an estimate of the number of high quality reads obtainable from our raw data, but will change depending on the parameters selected during quality processing.

Due to cost of ^13^C-labeled materials and additional labour required to process SIP samples, only a subset of ecozones were selected for SIP-hemicellulose (PP_CA_ & IDF_BC_) and SIP-Cellulose (PP_CA_) characterizations. The raw pyrosequencing output (~400-bp reads) for SIP-hemicellulose 16S rRNA (Data Citation 4) and ITS (Data Citation 5) amplicon libraries, which averaged 4,200 and 3,800 reads per library (post-QC), respectively, are available in SFF format from the European Nucleotide Archive (Table 4 (available online only)). These datasets include both ^13^C- and ^12^C-libraries for 16S rRNA genes, *n*=35 and 15, respectively, and for ITS, 18 and 2, respectively. Similarly, the raw sequencing data for all SIP-cellulose 16S rRNA amplicon and ITS amplicon libraries, averaging 11,800 and 10,300 reads per library, respectively, are available in ‘fastq’ format from the European Nucleotide Archive (Table 4 (available online only)). The ^13^C- and ^12^C-libraries from these data are more balanced, with a total of 2416S rRNA gene libraries for both, and 24 and 23, respectively, for ITS libraries. Raw Illumina, paired-end, 100-bp shotgun metagenome sequencing libraries for pooled, SIP-cellulose mineral soil incubations were archived in ‘fastq’ format (Data Citation 6; Table 4 (available online only)), along with 10 partial genomes in ‘fasta’ format comprised of assembled scaffolds (Data Citation 6; Sample accessions: ERZ288956—ERZ288966).

## Technical Validation

**Figure 3:**
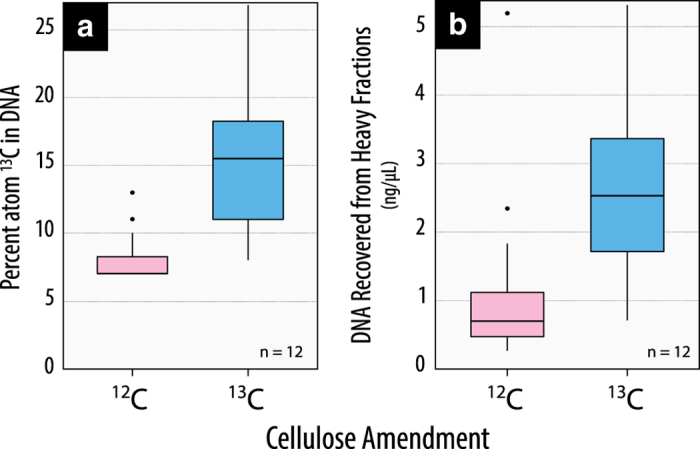
The successful enrichment and recovery of ^13^C-enriched DNA in SIP experiments. The assimilation of ^13^C by functional guilds during stable isotope probing experiments was evident in (**a**) the total ^13^C-enrichment of soil DNA extract and (**b**) recovery of DNA from the densest CsCl gradient fractions (F_1_-F_7_). These differences were evident in comparing soils amended with either ^12^C- (unlabelled) or ^13^C-cellulose. These trends were apparent in both cellulose and hemicellulose SIP experiments. Boxplots depict the average (centre line) and spread (from 25th to 75th percentile) of data, while the whiskers extend to the extrema. A total of twelve samples were averaged for each factor.

The recovery of ^13^C-enriched DNA was validated by quantifying ^13^C-content of nucleic acids^18^ (Fig. 3). The completeness of draft genomes recovered from SIP-Cellulose metagenomes was assessed by scanning for essential single-copy, house-keeping genes with hidden Markov models^21^.

## Usage Notes

Metagenomes for all ecozones, except IDF_BC_, can be also found at the DOE JGI portal (http://genome.jgi.doe.gov/) under proposal ID 543. This site provides the raw sequencing data and annotation for the assembled and unassembled metagenomes.

## Additional Information

Tables 2–4 are only available in the online version of this paper.

**How to cite this article**: Wilhelm, R. C. *et al.* A metagenomic survey of forest soil microbial communities more than a decade after timber harvesting. *Sci. Data* 4:170092 doi: 10.1038/sdata.2017.92 (2017).

**Publisher**’**s note**: Springer Nature remains neutral with regard to jurisdictional claims in published maps and institutional affiliations.

## Supplementary Material



## Figures and Tables

**Table 1:** Site information for all sampling locations utilized in this study.

**Site Name**	**Ecozone code**	**Site code**	**Region**	**Latitude**	**Longitude**	**Elevation (m)**	**Soil classification**	**Tree cover**	**Climatic zone**	**Annual mean temp (°C)**	**Precipitation**—**warmest quarter (mm)**	**Year established**	**Sample collection date**	**Country**
Fensom	BS_ON_	A7	Ontario	49.07	−89.41	445	Orthic Dystric Brunisol	Black Spruce	Dfb, Humid Continental warm summer	2.4	266	1995	7/3/2011	Canada
Fensom	BS_ON_	A8	Ontario	49.08	−89.38	450	Orthic Dystric Brunisol	Black Spruce	Dfb, Humid Continental warm summer	1.8	266	1995	7/4/2011	Canada
Fensom	BS_ON_	A9	Ontario	49.07	−89.39	442	Gleyed Dystric Brunisol	Black Spruce	Dfb, Humid Continental warm summer	1.5	266	1995	7/5/2011	Canada
Brandy City	PP_CA_	BR	California	39.55	−121.04	1135	Mesic Ultic Haploxeralfs	Ponderosa pine, sugar pine, white fir, giant sequoia	Csa, Mediterranean hot summer	11.2	55	1995	6/22/2011	USA
Blodgett	PP_CA_	BL	California	38.88	−120.64	1350	Mesic Ultic Haploxeralfs	Ponderosa pine, sugar pine, white fir, giant sequoia	Csa, Mediterranean hot summer	11.2	55	1995	9/16/2011	USA
Lowell Hill	PP_CA_	LH	California	39.26	−120.78	1268	Mesic Ultic Haploxeralfs	Ponderosa pine, sugar pine, white fir, giant sequoia	Csa, Mediterranean hot summer	11.2	55	1995	9/16/2011	USA
Wells	JP_ON_	JW	Ontario	46.42	−83.37	228	Orthic Humo-Ferric Podzol	Jack Pine, Black Spruce, Red Pine	Dfb, Humid Continental cool summer	4.4	248	1993–1994	7/7/2011	Canada
Superior	JP_ON_	JS	Ontario	47.57	−82.85	426	Orthic Dystric Brunisol	Jack Pine, Black Spruce	Dfb, Humid Continental cool summer	1.7	250	1993–1994	8/4/2011	Canada
Eddy	JP_ON_	JE	Ontario	46.75	−82.25	490	NA	Jack Pine, Balsam fir, White birch	Dfb, Humid Continental cool summer	2.8	242	1993–1994	8/3/2011	Canada
Kurth	LP_TX_	TXA	Texas	31.11	−95.15	88	Aquic Glossudalfs	Loblolly Pine, Beautyberry, Yaupon, Sweetgum, Oaks, Wax Myrtle	Cfa, Humid subtropical	19.0	253	1997	3/12/2012	USA
Kurth	LP_TX_	TXB	Texas	31.11	−95.15	88	Aquic Glossudalfs	Loblolly Pine, Beautyberry, Yaupon, Sweetgum, Oaks, Wax Myrtle	Cfa, Humid subtropical	19.0	253	1997	3/12/2012	USA
Kurth	LP_TX_	TXC	Texas	31.11	−95.15	88	Aquic Glossudalfs	Loblolly Pine, Beautyberry, Yaupon, Sweetgum, Oaks, Wax Myrtle	Cfa, Humid subtropical	19.0	253	1997	3/12/2012	USA
O’Connor Lake	IDF_BC_	OC	British Columbia	50.88	−120.35	1075	Brunisolic Gray Luvisol	Douglas fir	Dfb, Humid Continental warm summer	2.5	300	1999	6/26/2010	Canada
Black Pines	IDF_BC_	BP	British Columbia	50.93	−120.28	1180	Brunisolic Gray Luvisol	Douglas fir, Lodgepole pine	Dfb, Humid Continental warm summer	2.5	300	1999	6/22/2010	Canada
Dairy Creek	IDF_BC_	DC	British Columbia	50.85	−120.42	1150	Brunisolic Gray Luvisol	Douglas fir, Subalpine fir, Lodgepole pine	Dfb, Humid Continental warm summer	2.5	300	1999	6/25/2010	Canada
Log Lake	SBS_BC_	LL	British Columbia	54.35	−122.61	780	Orthic Humo-Ferric Podzol, Gleyed Eluviated Dystric Brunisol	Subalpine fir, Douglas fir, Interior Spruce	Dfc, Boreal cool summer	3.3	146–193	1994	7/9/2008	Canada
Topley	SBS_BC_	TO	British Columbia	52.32	−126.31	1100	Orthic Gray Luvisol, Gleyed Gray Luvisol	Lodgepole pine, Subalpine fir, Interior spruce	Dfc, Boreal cool summer	1.7	146–193	1994	7/11/2008	Canada
Skulow Lake	SBS_BC_	SL	British Columbia	52.32	−121.92	1050	Orthic Gray Luvisol	Lodgepole pine, Interior spruce	Dfc, Boreal cool summer	3.8	146–193	1995	8/14/2009	Canada

**Table 2:** Sequencing and sample data for all field whole genome shotgun libraries

**Sample Alias**	**Sample ID**	**Ecozone**	**Region**	**Site**	**Environmental Source**	**DNA Source**	**Library Preparation**	**Library Type**	**Common Name**	**Instrument Model**	**Data Repository**	**ENA Study Accession**	**Sample Accession**	**Secondary Sample Accession**	**Experiment Accession**	**Run Accession**	**Phylogenetic Marker**	**Target Gene Subfragment**	**Barcode**	**Forward Primer**	**Reverse Primer**	**Collection Date**	**Latitude**	**Longitude**	**Country**	**Sampling Depth**	**Elevation**	**Mean Annual Temperature (Celsius)**	**Mean Annual Precipitation (mm)**	**Soil Classification**	**Tree Cover**	**Climatic Zone**	**LTSP Treatment**	**Compaction Treatment**	**Herbicide Use**	**Horizon**	**Moisture Content**	**Total Carbon**	**Total Nitrogen**	**pH**	**Soil Bulk Density**	**CN Ratio**
A7001F	A7001	BS_ON_	Ontario	A7	Forest Soil	Whole Community DNA	PCR	Amplicon	pyrotag library	454 GS FLX Titanium	European Nucleotide Archive	https://www.ebi.ac.uk/ena/data/search?query=PRJEB12501	ERS1039589	SAMEA3732440	ERX1297926	ERR1225714	ITS	ITS2	ACGAGTGCGT	TCCTCCGCTTATTGATATGC	GCATCGATGAAGAACGCAGC	2011-07-03	49.07	-89.41	Canada	0.1	445	0.4	266	Orthic Dystric Brunisol	Black Spruce	Dfb, Humid Continental warm summer	OM2	C0	0	O horizon	48.0	42.5	1.1	5.4	0.2	37.9
A7002F	A7002	BS_ON_	Ontario	A7	Forest Soil	Whole Community DNA	PCR	Amplicon	pyrotag library	454 GS FLX Titanium	European Nucleotide Archive	https://www.ebi.ac.uk/ena/data/search?query=PRJEB12501	ERS1039590	SAMEA3732441	ERX1297927	ERR1225715	ITS	ITS2	TCACGTACTA	TCCTCCGCTTATTGATATGC	GCATCGATGAAGAACGCAGC	2011-07-03	49.07	-89.41	Canada	0.3	445	0.4	266	Orthic Dystric Brunisol	Black Spruce	Dfb, Humid Continental warm summer	OM2	C0	0	A horizon	18.0	0.6	0.0	5.7	1.3	16.3
A7004F	A7004	BS_ON_	Ontario	A7	Forest Soil	Whole Community DNA	PCR	Amplicon	pyrotag library	454 GS FLX Titanium	European Nucleotide Archive	https://www.ebi.ac.uk/ena/data/search?query=PRJEB12501	ERS1039591	SAMEA3732442	ERX1297928	ERR1225716	ITS	ITS2	TAGTGTAGAT	TCCTCCGCTTATTGATATGC	GCATCGATGAAGAACGCAGC	2011-07-03	49.07	-89.41	Canada	0.3	445	0.4	266	Orthic Dystric Brunisol	Black Spruce	Dfb, Humid Continental warm summer	OM3	C0	0	A horizon	16.0	1.2	0.1	6.0	1.0	20.1
A7007F	A7007	BS_ON_	Ontario	A7	Forest Soil	Whole Community DNA	PCR	Amplicon	pyrotag library	454 GS FLX Titanium	European Nucleotide Archive	https://www.ebi.ac.uk/ena/data/search?query=PRJEB12501	ERS1039592	SAMEA3732443	ERX1297929	ERR1225717	ITS	ITS2	CTCGCGTGTC	TCCTCCGCTTATTGATATGC	GCATCGATGAAGAACGCAGC	2011-07-03	49.07	-89.41	Canada	0.1	445	0.4	266	Orthic Dystric Brunisol	Black Spruce	Dfb, Humid Continental warm summer	OM1	C0	0	O horizon	54.0	34.6	0.8	5.0	0.2	42.4
A7009F	A7009	BS_ON_	Ontario	A7	Forest Soil	Whole Community DNA	PCR	Amplicon	pyrotag library	454 GS FLX Titanium	European Nucleotide Archive	https://www.ebi.ac.uk/ena/data/search?query=PRJEB12501	ERS1039593	SAMEA3732444	ERX1297930	ERR1225718	ITS	ITS2	AGACTATACT	TCCTCCGCTTATTGATATGC	GCATCGATGAAGAACGCAGC	2011-07-03	49.07	-89.41	Canada	0.1	445	0.4	266	Orthic Dystric Brunisol	Black Spruce	Dfb, Humid Continental warm summer	OM2	C0	0	O horizon	30.0	34.9	0.9	4.4	0.2	39.8
A7011F	A7011	BS_ON_	Ontario	A7	Forest Soil	Whole Community DNA	PCR	Amplicon	pyrotag library	454 GS FLX Titanium	European Nucleotide Archive	https://www.ebi.ac.uk/ena/data/search?query=PRJEB12501	ERS1039594	SAMEA3732445	ERX1297931	ERR1225719	ITS	ITS2	TGATACGTCT	TCCTCCGCTTATTGATATGC	GCATCGATGAAGAACGCAGC	2011-07-03	49.07	-89.41	Canada	0.1	445	0.4	266	Orthic Dystric Brunisol	Black Spruce	Dfb, Humid Continental warm summer	OM3	C0	0	O horizon	34.0	33.8	0.9	5.1	0.2	38.1
A7012F	A7012	BS_ON_	Ontario	A7	Forest Soil	Whole Community DNA	PCR	Amplicon	pyrotag library	454 GS FLX Titanium	European Nucleotide Archive	https://www.ebi.ac.uk/ena/data/search?query=PRJEB12501	ERS1039595	SAMEA3732446	ERX1297932	ERR1225720	ITS	ITS2	TCTCTATGCG	TCCTCCGCTTATTGATATGC	GCATCGATGAAGAACGCAGC	2011-07-03	49.07	-89.41	Canada	0.3	445	0.4	266	Orthic Dystric Brunisol	Black Spruce	Dfb, Humid Continental warm summer	OM3	C0	0	A horizon	19.0	1.4	0.1	5.7	1.0	21.5
A7015F	A7015	BS_ON_	Ontario	A7	Forest Soil	Whole Community DNA	PCR	Amplicon	pyrotag library	454 GS FLX Titanium	European Nucleotide Archive	https://www.ebi.ac.uk/ena/data/search?query=PRJEB12501	ERS1039596	SAMEA3732447	ERX1297933	ERR1225721	ITS	ITS2	ACTGTACAGT	TCCTCCGCTTATTGATATGC	GCATCGATGAAGAACGCAGC	2011-07-03	49.07	-89.41	Canada	0.1	445	0.4	266	Orthic Dystric Brunisol	Black Spruce	Dfb, Humid Continental warm summer	OM1	C0	0	O horizon	37.0	37.3	1.0	5.1	0.2	39.3
A7016F	A7016	BS_ON_	Ontario	A7	Forest Soil	Whole Community DNA	PCR	Amplicon	pyrotag library	454 GS FLX Titanium	European Nucleotide Archive	https://www.ebi.ac.uk/ena/data/search?query=PRJEB12501	ERS1039597	SAMEA3732448	ERX1297934	ERR1225722	ITS	ITS2	ATAGAGTACT	TCCTCCGCTTATTGATATGC	GCATCGATGAAGAACGCAGC	2011-07-03	49.07	-89.41	Canada	0.3	445	0.4	266	Orthic Dystric Brunisol	Black Spruce	Dfb, Humid Continental warm summer	OM1	C0	0	A horizon	25.0	2.5	0.1	5.5	1.1	21.3
A7019F	A7019	BS_ON_	Ontario	A7	Forest Soil	Whole Community DNA	PCR	Amplicon	pyrotag library	454 GS FLX Titanium	European Nucleotide Archive	https://www.ebi.ac.uk/ena/data/search?query=PRJEB12501	ERS1039598	SAMEA3732449	ERX1297935	ERR1225723	ITS	ITS2	CGAGAGATAC	TCCTCCGCTTATTGATATGC	GCATCGATGAAGAACGCAGC	2011-07-03	49.07	-89.41	Canada	0.1	445	0.4	266	Orthic Dystric Brunisol	Black Spruce	Dfb, Humid Continental warm summer	OM2	C0	0	O horizon	50.0	46.9	1.2	4.3	0.2	40.1
A7021F	A7021	BS_ON_	Ontario	A7	Forest Soil	Whole Community DNA	PCR	Amplicon	pyrotag library	454 GS FLX Titanium	European Nucleotide Archive	https://www.ebi.ac.uk/ena/data/search?query=PRJEB12501	ERS1039599	SAMEA3732450	ERX1297936	ERR1225724	ITS	ITS2	CAGTAGACGT	TCCTCCGCTTATTGATATGC	GCATCGATGAAGAACGCAGC	2011-07-03	49.07	-89.41	Canada	0.1	445	0.4	266	Orthic Dystric Brunisol	Black Spruce	Dfb, Humid Continental warm summer	OM3	C0	0	O horizon	45.0	40.5	1.2	4.5	0.2	33.7
A7022F	A7022	BS_ON_	Ontario	A7	Forest Soil	Whole Community DNA	PCR	Amplicon	pyrotag library	454 GS FLX Titanium	European Nucleotide Archive	https://www.ebi.ac.uk/ena/data/search?query=PRJEB12501	ERS1039600	SAMEA3732451	ERX1297937	ERR1225725	ITS	ITS2	TACACGTGAT	TCCTCCGCTTATTGATATGC	GCATCGATGAAGAACGCAGC	2011-07-03	49.07	-89.41	Canada	0.3	445	0.4	266	Orthic Dystric Brunisol	Black Spruce	Dfb, Humid Continental warm summer	OM3	C0	0	A horizon	17.0	1.2	0.1	5.6	1.4	24.2
A7023F	A7023	BS_ON_	Ontario	A7	Forest Soil	Whole Community DNA	PCR	Amplicon	pyrotag library	454 GS FLX Titanium	European Nucleotide Archive	https://www.ebi.ac.uk/ena/data/search?query=PRJEB12501	ERS1039601	SAMEA3732452	ERX1297938	ERR1225726	ITS	ITS2	TACAGATCGT	TCCTCCGCTTATTGATATGC	GCATCGATGAAGAACGCAGC	2011-07-03	49.07	-89.41	Canada	0.1	445	0.4	266	Orthic Dystric Brunisol	Black Spruce	Dfb, Humid Continental warm summer	OM1	C0	0	O horizon	64.0	44.9	1.1	4.5	0.2	42.1
A7024F	A7024	BS_ON_	Ontario	A7	Forest Soil	Whole Community DNA	PCR	Amplicon	pyrotag library	454 GS FLX Titanium	European Nucleotide Archive	https://www.ebi.ac.uk/ena/data/search?query=PRJEB12501	ERS1039602	SAMEA3732453	ERX1297939	ERR1225727	ITS	ITS2	AGCGTCGTCT	TCCTCCGCTTATTGATATGC	GCATCGATGAAGAACGCAGC	2011-07-03	49.07	-89.41	Canada	0.3	445	0.4	266	Orthic Dystric Brunisol	Black Spruce	Dfb, Humid Continental warm summer	OM1	C0	0	A horizon	26.0	1.1	0.0	5.8	1.5	27.3
A7026F	A7026	BS_ON_	Ontario	A7	Forest Soil	Whole Community DNA	PCR	Amplicon	pyrotag library	454 GS FLX Titanium	European Nucleotide Archive	https://www.ebi.ac.uk/ena/data/search?query=PRJEB12501	ERS1039603	SAMEA3732454	ERX1297940	ERR1225728	ITS	ITS2	AGCACTGTAG	TCCTCCGCTTATTGATATGC	GCATCGATGAAGAACGCAGC	2011-07-03	49.07	-89.41	Canada	0.3	445	0.4	266	Orthic Dystric Brunisol	Black Spruce	Dfb, Humid Continental warm summer	REF	REF	0	A horizon	26.0	1.4	0.1	5.3	1.1	21.4
A7027F	A7027	BS_ON_	Ontario	A7	Forest Soil	Whole Community DNA	PCR	Amplicon	pyrotag library	454 GS FLX Titanium	European Nucleotide Archive	https://www.ebi.ac.uk/ena/data/search?query=PRJEB12501	ERS1039604	SAMEA3732455	ERX1297941	ERR1225729	ITS	ITS2	CGTGTCTCTA	TCCTCCGCTTATTGATATGC	GCATCGATGAAGAACGCAGC	2011-07-03	49.07	-89.41	Canada	0.1	445	0.4	266	Orthic Dystric Brunisol	Black Spruce	Dfb, Humid Continental warm summer	REF	REF	0	O horizon	58.0	44.7	1.0	4.4	0.1	44.4
A7028F	A7028	BS_ON_	Ontario	A7	Forest Soil	Whole Community DNA	PCR	Amplicon	pyrotag library	454 GS FLX Titanium	European Nucleotide Archive	https://www.ebi.ac.uk/ena/data/search?query=PRJEB12501	ERS1039605	SAMEA3732456	ERX1297942	ERR1225730	ITS	ITS2	TCTCTATGCG	TCCTCCGCTTATTGATATGC	GCATCGATGAAGAACGCAGC	2011-07-03	49.07	-89.41	Canada	0.3	445	0.4	266	Orthic Dystric Brunisol	Black Spruce	Dfb, Humid Continental warm summer	REF	REF	0	A horizon	25.0	2.1	0.1	5.3	0.9	24.3
A7029F	A7029	BS_ON_	Ontario	A7	Forest Soil	Whole Community DNA	PCR	Amplicon	pyrotag library	454 GS FLX Titanium	European Nucleotide Archive	https://www.ebi.ac.uk/ena/data/search?query=PRJEB12501	ERS1039606	SAMEA3732457	ERX1297943	ERR1225731	ITS	ITS2	AGACTATACT	TCCTCCGCTTATTGATATGC	GCATCGATGAAGAACGCAGC	2011-07-03	49.07	-89.41	Canada	0.1	445	0.4	266	Orthic Dystric Brunisol	Black Spruce	Dfb, Humid Continental warm summer	REF	REF	0	O horizon	58.0	40.7	1.0	4.2	0.2	40.2
A7030F	A7030	BS_ON_	Ontario	A7	Forest Soil	Whole Community DNA	PCR	Amplicon	pyrotag library	454 GS FLX Titanium	European Nucleotide Archive	https://www.ebi.ac.uk/ena/data/search?query=PRJEB12501	ERS1039607	SAMEA3732458	ERX1297944	ERR1225732	ITS	ITS2	ATAGAGTACT	TCCTCCGCTTATTGATATGC	GCATCGATGAAGAACGCAGC	2011-07-03	49.07	-89.41	Canada	0.3	445	0.4	266	Orthic Dystric Brunisol	Black Spruce	Dfb, Humid Continental warm summer	REF	REF	0	A horizon	16.0	2.4	0.1	5.2	0.9	20.3
A8031F	A8031	BS_ON_	Ontario	A8	Forest Soil	Whole Community DNA	PCR	Amplicon	pyrotag library	454 GS FLX Titanium	European Nucleotide Archive	https://www.ebi.ac.uk/ena/data/search?query=PRJEB12501	ERS1039608	SAMEA3732459	ERX1297945	ERR1225733	ITS	ITS2	ACGAGTGCGT	TCCTCCGCTTATTGATATGC	GCATCGATGAAGAACGCAGC	2011-07-04	49.08	-89.38	Canada	0.1	450	0.4	266	Orthic Dystric Brunisol	Black Spruce	Dfb, Humid Continental warm summer	OM1	C0	0	O horizon	54.0	41.4	1.2	4.6	0.2	35.4
A8036F	A8036	BS_ON_	Ontario	A8	Forest Soil	Whole Community DNA	PCR	Amplicon	pyrotag library	454 GS FLX Titanium	European Nucleotide Archive	https://www.ebi.ac.uk/ena/data/search?query=PRJEB12501	ERS1039609	SAMEA3732460	ERX1297946	ERR1225734	ITS	ITS2	CACGCTACGT	TCCTCCGCTTATTGATATGC	GCATCGATGAAGAACGCAGC	2011-07-04	49.08	-89.38	Canada	0.3	450	0.4	266	Orthic Dystric Brunisol	Black Spruce	Dfb, Humid Continental warm summer	OM3	C0	0	A horizon	14.0	0.8	0.0	5.6	1.7	21.6
A8037F	A8037	BS_ON_	Ontario	A8	Forest Soil	Whole Community DNA	PCR	Amplicon	pyrotag library	454 GS FLX Titanium	European Nucleotide Archive	https://www.ebi.ac.uk/ena/data/search?query=PRJEB12501	ERS1039610	SAMEA3732461	ERX1297947	ERR1225735	ITS	ITS2	CGAGAGATAC	TCCTCCGCTTATTGATATGC	GCATCGATGAAGAACGCAGC	2011-07-04	49.08	-89.38	Canada	0.1	450	0.4	266	Orthic Dystric Brunisol	Black Spruce	Dfb, Humid Continental warm summer	OM2	C0	0	O horizon	46.0	42.3	1.1	5.0	0.2	39.1
A8038F	A8038	BS_ON_	Ontario	A8	Forest Soil	Whole Community DNA	PCR	Amplicon	pyrotag library	454 GS FLX Titanium	European Nucleotide Archive	https://www.ebi.ac.uk/ena/data/search?query=PRJEB12501	ERS1039611	SAMEA3732462	ERX1297948	ERR1225736	ITS	ITS2	AGACTATACT	TCCTCCGCTTATTGATATGC	GCATCGATGAAGAACGCAGC	2011-07-04	49.08	-89.38	Canada	0.3	450	0.4	266	Orthic Dystric Brunisol	Black Spruce	Dfb, Humid Continental warm summer	OM2	C0	0	A horizon	19.0	1.3	0.1	5.0	1.3	20.8
A8040F	A8040	BS_ON_	Ontario	A8	Forest Soil	Whole Community DNA	PCR	Amplicon	pyrotag library	454 GS FLX Titanium	European Nucleotide Archive	https://www.ebi.ac.uk/ena/data/search?query=PRJEB12501	ERS1039612	SAMEA3732463	ERX1297949	ERR1225737	ITS	ITS2	ACTACTATGT	TCCTCCGCTTATTGATATGC	GCATCGATGAAGAACGCAGC	2011-07-04	49.08	-89.38	Canada	0.3	450	0.4	266	Orthic Dystric Brunisol	Black Spruce	Dfb, Humid Continental warm summer	OM2	C0	0	A horizon	25.5	1.6	0.1	5.4	1.3	21.6
A8041F	A8041	BS_ON_	Ontario	A8	Forest Soil	Whole Community DNA	PCR	Amplicon	pyrotag library	454 GS FLX Titanium	European Nucleotide Archive	https://www.ebi.ac.uk/ena/data/search?query=PRJEB12501	ERS1039613	SAMEA3732464	ERX1297950	ERR1225738	ITS	ITS2	ACGCTCGACA	TCCTCCGCTTATTGATATGC	GCATCGATGAAGAACGCAGC	2011-07-04	49.08	-89.38	Canada	0.1	450	0.4	266	Orthic Dystric Brunisol	Black Spruce	Dfb, Humid Continental warm summer	OM3	C0	0	O horizon	43.2	42.0	1.2	5.0	0.1	35.4
A8042F	A8042	BS_ON_	Ontario	A8	Forest Soil	Whole Community DNA	PCR	Amplicon	pyrotag library	454 GS FLX Titanium	European Nucleotide Archive	https://www.ebi.ac.uk/ena/data/search?query=PRJEB12501	ERS1039614	SAMEA3732465	ERX1297951	ERR1225739	ITS	ITS2	CGTCTAGTAC	TCCTCCGCTTATTGATATGC	GCATCGATGAAGAACGCAGC	2011-07-04	49.08	-89.38	Canada	0.3	450	0.4	266	Orthic Dystric Brunisol	Black Spruce	Dfb, Humid Continental warm summer	OM3	C0	0	A horizon	18.0	2.1	0.1	5.2	1.6	20.9
A8045F	A8045	BS_ON_	Ontario	A8	Forest Soil	Whole Community DNA	PCR	Amplicon	pyrotag library	454 GS FLX Titanium	European Nucleotide Archive	https://www.ebi.ac.uk/ena/data/search?query=PRJEB12501	ERS1039615	SAMEA3732466	ERX1297952	ERR1225740	ITS	ITS2	AGCACTGTAG	TCCTCCGCTTATTGATATGC	GCATCGATGAAGAACGCAGC	2011-07-04	49.08	-89.38	Canada	0.1	450	0.4	266	Orthic Dystric Brunisol	Black Spruce	Dfb, Humid Continental warm summer	OM1	C0	0	O horizon	36.0	41.6	1.2	4.7	0.2	34.2
A8046F	A8046	BS_ON_	Ontario	A8	Forest Soil	Whole Community DNA	PCR	Amplicon	pyrotag library	454 GS FLX Titanium	European Nucleotide Archive	https://www.ebi.ac.uk/ena/data/search?query=PRJEB12501	ERS1039616	SAMEA3732467	ERX1297953	ERR1225741	ITS	ITS2	TCTACGTAGC	TCCTCCGCTTATTGATATGC	GCATCGATGAAGAACGCAGC	2011-07-04	49.08	-89.38	Canada	0.3	450	0.4	266	Orthic Dystric Brunisol	Black Spruce	Dfb, Humid Continental warm summer	OM1	C0	0	A horizon	24.0	2.1	0.1	5.3	1.1	21.7
A8047F	A8047	BS_ON_	Ontario	A8	Forest Soil	Whole Community DNA	PCR	Amplicon	pyrotag library	454 GS FLX Titanium	European Nucleotide Archive	https://www.ebi.ac.uk/ena/data/search?query=PRJEB12501	ERS1039617	SAMEA3732468	ERX1297954	ERR1225742	ITS	ITS2	AGACGCACTC	TCCTCCGCTTATTGATATGC	GCATCGATGAAGAACGCAGC	2011-07-04	49.08	-89.38	Canada	0.1	450	0.4	266	Orthic Dystric Brunisol	Black Spruce	Dfb, Humid Continental warm summer	OM2	C0	0	O horizon	53.0	41.7	1.2	4.5	0.3	35.5
A8048F	A8048	BS_ON_	Ontario	A8	Forest Soil	Whole Community DNA	PCR	Amplicon	pyrotag library	454 GS FLX Titanium	European Nucleotide Archive	https://www.ebi.ac.uk/ena/data/search?query=PRJEB12501	ERS1039618	SAMEA3732469	ERX1297955	ERR1225743	ITS	ITS2	CGTGTCTCTA	TCCTCCGCTTATTGATATGC	GCATCGATGAAGAACGCAGC	2011-07-04	49.08	-89.38	Canada	0.3	450	0.4	266	Orthic Dystric Brunisol	Black Spruce	Dfb, Humid Continental warm summer	OM2	C0	0	A horizon	21.0	0.9	0.0	5.4	1.7	23.6
A8050F	A8050	BS_ON_	Ontario	A8	Forest Soil	Whole Community DNA	PCR	Amplicon	pyrotag library	454 GS FLX Titanium	European Nucleotide Archive	https://www.ebi.ac.uk/ena/data/search?query=PRJEB12501	ERS1039619	SAMEA3732470	ERX1297956	ERR1225744	ITS	ITS2	TACACGTGAT	TCCTCCGCTTATTGATATGC	GCATCGATGAAGAACGCAGC	2011-07-04	49.08	-89.38	Canada	0.3	450	0.4	266	Orthic Dystric Brunisol	Black Spruce	Dfb, Humid Continental warm summer	OM3	C0	0	A horizon	21.0	1.3	0.1	5.7	1.7	20.3
A8053F	A8053	BS_ON_	Ontario	A8	Forest Soil	Whole Community DNA	PCR	Amplicon	pyrotag library	454 GS FLX Titanium	European Nucleotide Archive	https://www.ebi.ac.uk/ena/data/search?query=PRJEB12501	ERS1039620	SAMEA3732471	ERX1297957	ERR1225745	ITS	ITS2	TCACGTACTA	TCCTCCGCTTATTGATATGC	GCATCGATGAAGAACGCAGC	2011-07-04	49.08	-89.38	Canada	0.1	450	0.4	266	Orthic Dystric Brunisol	Black Spruce	Dfb, Humid Continental warm summer	OM1	C0	0	O horizon	52.0	37.3	0.8	4.5	0.2	47.4
A8054F	A8054	BS_ON_	Ontario	A8	Forest Soil	Whole Community DNA	PCR	Amplicon	pyrotag library	454 GS FLX Titanium	European Nucleotide Archive	https://www.ebi.ac.uk/ena/data/search?query=PRJEB12501	ERS1039621	SAMEA3732472	ERX1297958	ERR1225746	ITS	ITS2	CTCGCGTGTC	TCCTCCGCTTATTGATATGC	GCATCGATGAAGAACGCAGC	2011-07-04	49.08	-89.38	Canada	0.3	450	0.4	266	Orthic Dystric Brunisol	Black Spruce	Dfb, Humid Continental warm summer	OM1	C0	0	A horizon	23.0	1.7	0.1	5.4	1.3	23.7
A8056F	A8056	BS_ON_	Ontario	A8	Forest Soil	Whole Community DNA	PCR	Amplicon	pyrotag library	454 GS FLX Titanium	European Nucleotide Archive	https://www.ebi.ac.uk/ena/data/search?query=PRJEB12501	ERS1039622	SAMEA3732473	ERX1297959	ERR1225747	ITS	ITS2	CATAGTAGTG	TCCTCCGCTTATTGATATGC	GCATCGATGAAGAACGCAGC	2011-07-04	49.08	-89.38	Canada	0.3	450	0.4	266	Orthic Dystric Brunisol	Black Spruce	Dfb, Humid Continental warm summer	REF	REF	0	A horizon	26.0	2.5	0.1	5.2	1.0	23.1
A8058F	A8058	BS_ON_	Ontario	A8	Forest Soil	Whole Community DNA	PCR	Amplicon	pyrotag library	454 GS FLX Titanium	European Nucleotide Archive	https://www.ebi.ac.uk/ena/data/search?query=PRJEB12501	ERS1039623	SAMEA3732474	ERX1297960	ERR1225748	ITS	ITS2	ACTACTATGT	TCCTCCGCTTATTGATATGC	GCATCGATGAAGAACGCAGC	2011-07-04	49.08	-89.38	Canada	0.3	450	0.4	266	Orthic Dystric Brunisol	Black Spruce	Dfb, Humid Continental warm summer	REF	REF	0	A horizon	28.0	3.3	0.1	4.9	0.8	27.3
A8059F	A8059	BS_ON_	Ontario	A8	Forest Soil	Whole Community DNA	PCR	Amplicon	pyrotag library	454 GS FLX Titanium	European Nucleotide Archive	https://www.ebi.ac.uk/ena/data/search?query=PRJEB12501	ERS1039624	SAMEA3732475	ERX1297961	ERR1225749	ITS	ITS2	ATAGAGTACT	TCCTCCGCTTATTGATATGC	GCATCGATGAAGAACGCAGC	2011-07-04	49.08	-89.38	Canada	0.1	450	0.4	266	Orthic Dystric Brunisol	Black Spruce	Dfb, Humid Continental warm summer	REF	REF	0	O horizon	73.0	45.7	1.1	4.2	0.1	43.5
A8060F	A8060	BS_ON_	Ontario	A8	Forest Soil	Whole Community DNA	PCR	Amplicon	pyrotag library	454 GS FLX Titanium	European Nucleotide Archive	https://www.ebi.ac.uk/ena/data/search?query=PRJEB12501	ERS1039625	SAMEA3732476	ERX1297962	ERR1225750	ITS	ITS2	TACACGTGAT	TCCTCCGCTTATTGATATGC	GCATCGATGAAGAACGCAGC	2011-07-04	49.08	-89.38	Canada	0.3	450	0.4	266	Orthic Dystric Brunisol	Black Spruce	Dfb, Humid Continental warm summer	REF	REF	0	A horizon	29.0	1.7	0.1	5.2	1.0	18.4
A9062F	A9062	BS_ON_	Ontario	A9	Forest Soil	Whole Community DNA	PCR	Amplicon	pyrotag library	454 GS FLX Titanium	European Nucleotide Archive	https://www.ebi.ac.uk/ena/data/search?query=PRJEB12501	ERS1039626	SAMEA3732477	ERX1297963	ERR1225751	ITS	ITS2	ATCAGACACG	TCCTCCGCTTATTGATATGC	GCATCGATGAAGAACGCAGC	2011-07-05	49.07	-89.39	Canada	0.3	442	0.4	266	Gleyed Dystric Brunisol	Black Spruce	Dfb, Humid Continental warm summer	OM1	C0	0	A horizon	20.0	3.3	0.1	5.4	0.9	23.5
A9063F	A9063	BS_ON_	Ontario	A9	Forest Soil	Whole Community DNA	PCR	Amplicon	pyrotag library	454 GS FLX Titanium	European Nucleotide Archive	https://www.ebi.ac.uk/ena/data/search?query=PRJEB12501	ERS1039627	SAMEA3732478	ERX1297964	ERR1225752	ITS	ITS2	ATACGACGTA	TCCTCCGCTTATTGATATGC	GCATCGATGAAGAACGCAGC	2011-07-05	49.07	-89.39	Canada	0.1	442	0.4	266	Gleyed Dystric Brunisol	Black Spruce	Dfb, Humid Continental warm summer	OM2	C0	0	O horizon	59.0	35.3	1.1	5.4	0.2	31.9
A9064F	A9064	BS_ON_	Ontario	A9	Forest Soil	Whole Community DNA	PCR	Amplicon	pyrotag library	454 GS FLX Titanium	European Nucleotide Archive	https://www.ebi.ac.uk/ena/data/search?query=PRJEB12501	ERS1039628	SAMEA3732479	ERX1297965	ERR1225753	ITS	ITS2	AGTACGCTAT	TCCTCCGCTTATTGATATGC	GCATCGATGAAGAACGCAGC	2011-07-05	49.07	-89.39	Canada	0.3	442	0.4	266	Gleyed Dystric Brunisol	Black Spruce	Dfb, Humid Continental warm summer	OM2	C0	0	A horizon	24.0	2.2	0.1	5.6	1.2	21.2
A9067F	A9067	BS_ON_	Ontario	A9	Forest Soil	Whole Community DNA	PCR	Amplicon	pyrotag library	454 GS FLX Titanium	European Nucleotide Archive	https://www.ebi.ac.uk/ena/data/search?query=PRJEB12501	ERS1039629	SAMEA3732480	ERX1297966	ERR1225754	ITS	ITS2	CACGCTACGT	TCCTCCGCTTATTGATATGC	GCATCGATGAAGAACGCAGC	2011-07-05	49.07	-89.39	Canada	0.1	442	0.4	266	Gleyed Dystric Brunisol	Black Spruce	Dfb, Humid Continental warm summer	OM3	C0	0	O horizon	53.0	32.6	0.9	5.4	0.2	37.2
A9068F	A9068	BS_ON_	Ontario	A9	Forest Soil	Whole Community DNA	PCR	Amplicon	pyrotag library	454 GS FLX Titanium	European Nucleotide Archive	https://www.ebi.ac.uk/ena/data/search?query=PRJEB12501	ERS1039630	SAMEA3732481	ERX1297967	ERR1225755	ITS	ITS2	TACACGTGAT	TCCTCCGCTTATTGATATGC	GCATCGATGAAGAACGCAGC	2011-07-05	49.07	-89.39	Canada	0.3	442	0.4	266	Gleyed Dystric Brunisol	Black Spruce	Dfb, Humid Continental warm summer	OM3	C0	0	A horizon	23.0	2.3	0.1	5.8	1.2	18.3
A9070F	A9070	BS_ON_	Ontario	A9	Forest Soil	Whole Community DNA	PCR	Amplicon	pyrotag library	454 GS FLX Titanium	European Nucleotide Archive	https://www.ebi.ac.uk/ena/data/search?query=PRJEB12501	ERS1039631	SAMEA3732482	ERX1297968	ERR1225756	ITS	ITS2	TCTCTATGCG	TCCTCCGCTTATTGATATGC	GCATCGATGAAGAACGCAGC	2011-07-05	49.07	-89.39	Canada	0.3	442	0.4	266	Gleyed Dystric Brunisol	Black Spruce	Dfb, Humid Continental warm summer	OM2	C0	0	A horizon	20.0	0.7	0.0	5.8	1.2	17.5
A9073F	A9073	BS_ON_	Ontario	A9	Forest Soil	Whole Community DNA	PCR	Amplicon	pyrotag library	454 GS FLX Titanium	European Nucleotide Archive	https://www.ebi.ac.uk/ena/data/search?query=PRJEB12501	ERS1039632	SAMEA3732483	ERX1297969	ERR1225757	ITS	ITS2	TACGCTGTCT	TCCTCCGCTTATTGATATGC	GCATCGATGAAGAACGCAGC	2011-07-05	49.07	-89.39	Canada	0.1	442	0.4	266	Gleyed Dystric Brunisol	Black Spruce	Dfb, Humid Continental warm summer	OM2	C0	0	O horizon	64.0	45.1	1.1	5.0	0.2	40.2
A9074F	A9074	BS_ON_	Ontario	A9	Forest Soil	Whole Community DNA	PCR	Amplicon	pyrotag library	454 GS FLX Titanium	European Nucleotide Archive	https://www.ebi.ac.uk/ena/data/search?query=PRJEB12501	ERS1039633	SAMEA3732484	ERX1297970	ERR1225758	ITS	ITS2	TCGATCACGT	TCCTCCGCTTATTGATATGC	GCATCGATGAAGAACGCAGC	2011-07-05	49.07	-89.39	Canada	0.3	442	0.4	266	Gleyed Dystric Brunisol	Black Spruce	Dfb, Humid Continental warm summer	OM2	C0	0	A horizon	32.0	2.8	0.1	5.3	1.2	24.4
A9076F	A9076	BS_ON_	Ontario	A9	Forest Soil	Whole Community DNA	PCR	Amplicon	pyrotag library	454 GS FLX Titanium	European Nucleotide Archive	https://www.ebi.ac.uk/ena/data/search?query=PRJEB12501	ERS1039634	SAMEA3732485	ERX1297971	ERR1225759	ITS	ITS2	CACGCTACGT	TCCTCCGCTTATTGATATGC	GCATCGATGAAGAACGCAGC	2011-07-05	49.07	-89.39	Canada	0.3	442	0.4	266	Gleyed Dystric Brunisol	Black Spruce	Dfb, Humid Continental warm summer	OM1	C0	0	A horizon	21.0	1.5	0.1	5.6	1.2	19.3
A9077F	A9077	BS_ON_	Ontario	A9	Forest Soil	Whole Community DNA	PCR	Amplicon	pyrotag library	454 GS FLX Titanium	European Nucleotide Archive	https://www.ebi.ac.uk/ena/data/search?query=PRJEB12501	ERS1039635	SAMEA3732486	ERX1297972	ERR1225760	ITS	ITS2	TAGTGTAGAT	TCCTCCGCTTATTGATATGC	GCATCGATGAAGAACGCAGC	2011-07-05	49.07	-89.39	Canada	0.1	442	0.4	266	Gleyed Dystric Brunisol	Black Spruce	Dfb, Humid Continental warm summer	OM3	C0	0	O horizon	42.0	27.7	0.7	5.8	0.2	40.1
A9078F	A9078	BS_ON_	Ontario	A9	Forest Soil	Whole Community DNA	PCR	Amplicon	pyrotag library	454 GS FLX Titanium	European Nucleotide Archive	https://www.ebi.ac.uk/ena/data/search?query=PRJEB12501	ERS1039636	SAMEA3732487	ERX1297973	ERR1225761	ITS	ITS2	AGCACTGTAG	TCCTCCGCTTATTGATATGC	GCATCGATGAAGAACGCAGC	2011-07-05	49.07	-89.39	Canada	0.3	442	0.4	266	Gleyed Dystric Brunisol	Black Spruce	Dfb, Humid Continental warm summer	OM3	C0	0	A horizon	15.0	0.7	0.0	6.0	1.3	18.6
A9081F	A9081	BS_ON_	Ontario	A9	Forest Soil	Whole Community DNA	PCR	Amplicon	pyrotag library	454 GS FLX Titanium	European Nucleotide Archive	https://www.ebi.ac.uk/ena/data/search?query=PRJEB12501	ERS1039637	SAMEA3732488	ERX1297974	ERR1225762	ITS	ITS2	TCTCTATGCG	TCCTCCGCTTATTGATATGC	GCATCGATGAAGAACGCAGC	2011-07-05	49.07	-89.39	Canada	0.1	442	0.4	266	Gleyed Dystric Brunisol	Black Spruce	Dfb, Humid Continental warm summer	OM3	C0	0	O horizon	76.0	13.4	0.3	5.3	0.2	46.2
A9082F	A9082	BS_ON_	Ontario	A9	Forest Soil	Whole Community DNA	PCR	Amplicon	pyrotag library	454 GS FLX Titanium	European Nucleotide Archive	https://www.ebi.ac.uk/ena/data/search?query=PRJEB12501	ERS1039638	SAMEA3732489	ERX1297975	ERR1225763	ITS	ITS2	AGACGCACTC	TCCTCCGCTTATTGATATGC	GCATCGATGAAGAACGCAGC	2011-07-05	49.07	-89.39	Canada	0.3	442	0.4	266	Gleyed Dystric Brunisol	Black Spruce	Dfb, Humid Continental warm summer	OM3	C0	0	A horizon	19.0	1.6	0.1	6.1	1.1	19.9
A9083F	A9083	BS_ON_	Ontario	A9	Forest Soil	Whole Community DNA	PCR	Amplicon	pyrotag library	454 GS FLX Titanium	European Nucleotide Archive	https://www.ebi.ac.uk/ena/data/search?query=PRJEB12501	ERS1039639	SAMEA3732490	ERX1297976	ERR1225764	ITS	ITS2	ACGAGTGCGT	TCCTCCGCTTATTGATATGC	GCATCGATGAAGAACGCAGC	2011-07-05	49.07	-89.39	Canada	0.1	442	0.4	266	Gleyed Dystric Brunisol	Black Spruce	Dfb, Humid Continental warm summer	OM1	C0	0	O horizon	69.0	29.0	0.9	5.4	0.2	30.9
A9085F	A9085	BS_ON_	Ontario	A9	Forest Soil	Whole Community DNA	PCR	Amplicon	pyrotag library	454 GS FLX Titanium	European Nucleotide Archive	https://www.ebi.ac.uk/ena/data/search?query=PRJEB12501	ERS1039640	SAMEA3732491	ERX1297977	ERR1225765	ITS	ITS2	CGTCTAGTAC	TCCTCCGCTTATTGATATGC	GCATCGATGAAGAACGCAGC	2011-07-05	49.07	-89.39	Canada	0.1	442	0.4	266	Gleyed Dystric Brunisol	Black Spruce	Dfb, Humid Continental warm summer	REF	REF	0	O horizon	64.0	43.9	1.2	4.8	0.1	35.7
A9086F	A9086	BS_ON_	Ontario	A9	Forest Soil	Whole Community DNA	PCR	Amplicon	pyrotag library	454 GS FLX Titanium	European Nucleotide Archive	https://www.ebi.ac.uk/ena/data/search?query=PRJEB12501	ERS1039641	SAMEA3732492	ERX1297978	ERR1225766	ITS	ITS2	AGTACGCTAT	TCCTCCGCTTATTGATATGC	GCATCGATGAAGAACGCAGC	2011-07-05	49.07	-89.39	Canada	0.3	442	0.4	266	Gleyed Dystric Brunisol	Black Spruce	Dfb, Humid Continental warm summer	REF	REF	0	A horizon	28.0	1.9	0.1	5.4	1.2	18.9
A9087F	A9087	BS_ON_	Ontario	A9	Forest Soil	Whole Community DNA	PCR	Amplicon	pyrotag library	454 GS FLX Titanium	European Nucleotide Archive	https://www.ebi.ac.uk/ena/data/search?query=PRJEB12501	ERS1039642	SAMEA3732493	ERX1297979	ERR1225767	ITS	ITS2	ATATCGCGAG	TCCTCCGCTTATTGATATGC	GCATCGATGAAGAACGCAGC	2011-07-05	49.07	-89.39	Canada	0.1	442	0.4	266	Gleyed Dystric Brunisol	Black Spruce	Dfb, Humid Continental warm summer	REF	REF	0	O horizon	57.0	45.5	1.1	4.8	0.1	39.9
A9088F	A9088	BS_ON_	Ontario	A9	Forest Soil	Whole Community DNA	PCR	Amplicon	pyrotag library	454 GS FLX Titanium	European Nucleotide Archive	https://www.ebi.ac.uk/ena/data/search?query=PRJEB12501	ERS1039643	SAMEA3732494	ERX1297980	ERR1225768	ITS	ITS2	AGCGTCGTCT	TCCTCCGCTTATTGATATGC	GCATCGATGAAGAACGCAGC	2011-07-05	49.07	-89.39	Canada	0.3	442	0.4	266	Gleyed Dystric Brunisol	Black Spruce	Dfb, Humid Continental warm summer	REF	REF	0	A horizon	30.0	1.4	0.1	5.9	1.4	20.1
A9089F	A9089	BS_ON_	Ontario	A9	Forest Soil	Whole Community DNA	PCR	Amplicon	pyrotag library	454 GS FLX Titanium	European Nucleotide Archive	https://www.ebi.ac.uk/ena/data/search?query=PRJEB12501	ERS1039644	SAMEA3732495	ERX1297981	ERR1225769	ITS	ITS2	TACACACACT	TCCTCCGCTTATTGATATGC	GCATCGATGAAGAACGCAGC	2011-07-05	49.07	-89.39	Canada	0.1	442	0.4	266	Gleyed Dystric Brunisol	Black Spruce	Dfb, Humid Continental warm summer	REF	REF	0	O horizon	71.0	44.2	1.0	4.7	0.1	43.1
A9090F	A9090	BS_ON_	Ontario	A9	Forest Soil	Whole Community DNA	PCR	Amplicon	pyrotag library	454 GS FLX Titanium	European Nucleotide Archive	https://www.ebi.ac.uk/ena/data/search?query=PRJEB12501	ERS1039645	SAMEA3732496	ERX1297982	ERR1225770	ITS	ITS2	ATATCGCGAG	TCCTCCGCTTATTGATATGC	GCATCGATGAAGAACGCAGC	2011-07-05	49.07	-89.39	Canada	0.3	442	0.4	266	Gleyed Dystric Brunisol	Black Spruce	Dfb, Humid Continental warm summer	REF	REF	0	A horizon	25.0	1.2	0.1	5.6	1.5	22.3
A9092F	A9092	BS_ON_	Ontario	A9	Forest Soil	Whole Community DNA	PCR	Amplicon	pyrotag library	454 GS FLX Titanium	European Nucleotide Archive	https://www.ebi.ac.uk/ena/data/search?query=PRJEB12501	ERS1039646	SAMEA3732497	ERX1297983	ERR1225771	ITS	ITS2	ACGCTCGACA	TCCTCCGCTTATTGATATGC	GCATCGATGAAGAACGCAGC	2011-07-05	49.07	-89.39	Canada	0.3	442	0.4	266	Gleyed Dystric Brunisol	Black Spruce	Dfb, Humid Continental warm summer	99	C0	0	A horizon	19.0	NA	NA	0.0	NA	NA
A9093F	A9093	BS_ON_	Ontario	A9	Forest Soil	Whole Community DNA	PCR	Amplicon	pyrotag library	454 GS FLX Titanium	European Nucleotide Archive	https://www.ebi.ac.uk/ena/data/search?query=PRJEB12501	ERS1039647	SAMEA3732498	ERX1297984	ERR1225772	ITS	ITS2	TGATACGTCT	TCCTCCGCTTATTGATATGC	GCATCGATGAAGAACGCAGC	2011-07-05	49.07	-89.39	Canada	0.1	442	0.4	266	Gleyed Dystric Brunisol	Black Spruce	Dfb, Humid Continental warm summer	99	C0	0	O horizon	54.0	NA	NA	0.0	NA	NA
A9094F	A9094	BS_ON_	Ontario	A9	Forest Soil	Whole Community DNA	PCR	Amplicon	pyrotag library	454 GS FLX Titanium	European Nucleotide Archive	https://www.ebi.ac.uk/ena/data/search?query=PRJEB12501	ERS1039648	SAMEA3732499	ERX1297985	ERR1225773	ITS	ITS2	ACTGTACAGT	TCCTCCGCTTATTGATATGC	GCATCGATGAAGAACGCAGC	2011-07-05	49.07	-89.39	Canada	0.3	442	0.4	266	Gleyed Dystric Brunisol	Black Spruce	Dfb, Humid Continental warm summer	99	C0	0	A horizon	17.0	NA	NA	0.0	NA	NA
A9096F	A9096	BS_ON_	Ontario	A9	Forest Soil	Whole Community DNA	PCR	Amplicon	pyrotag library	454 GS FLX Titanium	European Nucleotide Archive	https://www.ebi.ac.uk/ena/data/search?query=PRJEB12501	ERS1039649	SAMEA3732500	ERX1297986	ERR1225774	ITS	ITS2	AGACTATACT	TCCTCCGCTTATTGATATGC	GCATCGATGAAGAACGCAGC	2011-07-05	49.07	-89.39	Canada	0.3	442	0.4	266	Gleyed Dystric Brunisol	Black Spruce	Dfb, Humid Continental warm summer	99	C0	0	A horizon	9.0	NA	NA	0.0	NA	NA
A9097F	A9097	BS_ON_	Ontario	A9	Forest Soil	Whole Community DNA	PCR	Amplicon	pyrotag library	454 GS FLX Titanium	European Nucleotide Archive	https://www.ebi.ac.uk/ena/data/search?query=PRJEB12501	ERS1039650	SAMEA3732501	ERX1297987	ERR1225775	ITS	ITS2	ATAGAGTACT	TCCTCCGCTTATTGATATGC	GCATCGATGAAGAACGCAGC	2011-07-05	49.07	-89.39	Canada	0.1	442	0.4	266	Gleyed Dystric Brunisol	Black Spruce	Dfb, Humid Continental warm summer	99	C0	0	O horizon	39.0	NA	NA	0.0	NA	NA
A9099F	A9099	BS_ON_	Ontario	A9	Forest Soil	Whole Community DNA	PCR	Amplicon	pyrotag library	454 GS FLX Titanium	European Nucleotide Archive	https://www.ebi.ac.uk/ena/data/search?query=PRJEB12501	ERS1039651	SAMEA3732502	ERX1297988	ERR1225776	ITS	ITS2	ACTACTATGT	TCCTCCGCTTATTGATATGC	GCATCGATGAAGAACGCAGC	2011-07-05	49.07	-89.39	Canada	0.1	442	0.4	266	Gleyed Dystric Brunisol	Black Spruce	Dfb, Humid Continental warm summer	99	C0	0	O horizon	48.0	NA	NA	0.0	NA	NA
A9100F	A9100	BS_ON_	Ontario	A9	Forest Soil	Whole Community DNA	PCR	Amplicon	pyrotag library	454 GS FLX Titanium	European Nucleotide Archive	https://www.ebi.ac.uk/ena/data/search?query=PRJEB12501	ERS1039652	SAMEA3732503	ERX1297989	ERR1225777	ITS	ITS2	TACAGATCGT	TCCTCCGCTTATTGATATGC	GCATCGATGAAGAACGCAGC	2011-07-05	49.07	-89.39	Canada	0.3	442	0.4	266	Gleyed Dystric Brunisol	Black Spruce	Dfb, Humid Continental warm summer	99	C0	0	A horizon	13.0	NA	NA	0.0	NA	NA
A9101F	A9101	BS_ON_	Ontario	A9	Forest Soil	Whole Community DNA	PCR	Amplicon	pyrotag library	454 GS FLX Titanium	European Nucleotide Archive	https://www.ebi.ac.uk/ena/data/search?query=PRJEB12501	ERS1039653	SAMEA3732504	ERX1297990	ERR1225778	ITS	ITS2	TACACACACT	TCCTCCGCTTATTGATATGC	GCATCGATGAAGAACGCAGC	2011-07-05	49.07	-89.39	Canada	0.1	442	0.4	266	Gleyed Dystric Brunisol	Black Spruce	Dfb, Humid Continental warm summer	99	C0	0	O horizon	29.0	NA	NA	0.0	NA	NA
A9102F	A9102	BS_ON_	Ontario	A9	Forest Soil	Whole Community DNA	PCR	Amplicon	pyrotag library	454 GS FLX Titanium	European Nucleotide Archive	https://www.ebi.ac.uk/ena/data/search?query=PRJEB12501	ERS1039654	SAMEA3732505	ERX1297991	ERR1225779	ITS	ITS2	ATCAGACACG	TCCTCCGCTTATTGATATGC	GCATCGATGAAGAACGCAGC	2011-07-05	49.07	-89.39	Canada	0.3	442	0.4	266	Gleyed Dystric Brunisol	Black Spruce	Dfb, Humid Continental warm summer	99	C0	0	A horizon	7.6	NA	NA	0.0	NA	NA
A9103F	A9103	BS_ON_	Ontario	A9	Forest Soil	Whole Community DNA	PCR	Amplicon	pyrotag library	454 GS FLX Titanium	European Nucleotide Archive	https://www.ebi.ac.uk/ena/data/search?query=PRJEB12501	ERS1039655	SAMEA3732506	ERX1297992	ERR1225780	ITS	ITS2	AGACGCACTC	TCCTCCGCTTATTGATATGC	GCATCGATGAAGAACGCAGC	2011-07-05	49.07	-89.39	Canada	0.1	442	0.4	266	Gleyed Dystric Brunisol	Black Spruce	Dfb, Humid Continental warm summer	99	C0	0	O horizon	14.0	NA	NA	0.0	NA	NA
A9104F	A9104	BS_ON_	Ontario	A9	Forest Soil	Whole Community DNA	PCR	Amplicon	pyrotag library	454 GS FLX Titanium	European Nucleotide Archive	https://www.ebi.ac.uk/ena/data/search?query=PRJEB12501	ERS1039656	SAMEA3732507	ERX1297993	ERR1225781	ITS	ITS2	AGACTATACT	TCCTCCGCTTATTGATATGC	GCATCGATGAAGAACGCAGC	2011-07-05	49.07	-89.39	Canada	0.3	442	0.4	266	Gleyed Dystric Brunisol	Black Spruce	Dfb, Humid Continental warm summer	99	C0	0	A horizon	13.0	NA	NA	0.0	NA	NA
A9105F	A9105	BS_ON_	Ontario	A9	Forest Soil	Whole Community DNA	PCR	Amplicon	pyrotag library	454 GS FLX Titanium	European Nucleotide Archive	https://www.ebi.ac.uk/ena/data/search?query=PRJEB12501	ERS1039657	SAMEA3732508	ERX1297994	ERR1225782	ITS	ITS2	ATACGACGTA	TCCTCCGCTTATTGATATGC	GCATCGATGAAGAACGCAGC	2011-07-05	49.07	-89.39	Canada	0.1	442	0.4	266	Gleyed Dystric Brunisol	Black Spruce	Dfb, Humid Continental warm summer	99	C0	0	O horizon	48.0	NA	NA	0.0	NA	NA
A9106F	A9106	BS_ON_	Ontario	A9	Forest Soil	Whole Community DNA	PCR	Amplicon	pyrotag library	454 GS FLX Titanium	European Nucleotide Archive	https://www.ebi.ac.uk/ena/data/search?query=PRJEB12501	ERS1039658	SAMEA3732509	ERX1297995	ERR1225783	ITS	ITS2	AGACGCACTC	TCCTCCGCTTATTGATATGC	GCATCGATGAAGAACGCAGC	2011-07-05	49.07	-89.39	Canada	0.3	442	0.4	266	Gleyed Dystric Brunisol	Black Spruce	Dfb, Humid Continental warm summer	99	C0	0	A horizon	19.0	NA	NA	0.0	NA	NA
A9107F	A9107	BS_ON_	Ontario	A9	Forest Soil	Whole Community DNA	PCR	Amplicon	pyrotag library	454 GS FLX Titanium	European Nucleotide Archive	https://www.ebi.ac.uk/ena/data/search?query=PRJEB12501	ERS1039659	SAMEA3732510	ERX1297996	ERR1225784	ITS	ITS2	ATAGAGTACT	TCCTCCGCTTATTGATATGC	GCATCGATGAAGAACGCAGC	2011-07-05	49.07	-89.39	Canada	0.1	442	0.4	266	Gleyed Dystric Brunisol	Black Spruce	Dfb, Humid Continental warm summer	99	C0	0	O horizon	38.0	NA	NA	0.0	NA	NA
A9108F	A9108	BS_ON_	Ontario	A9	Forest Soil	Whole Community DNA	PCR	Amplicon	pyrotag library	454 GS FLX Titanium	European Nucleotide Archive	https://www.ebi.ac.uk/ena/data/search?query=PRJEB12501	ERS1039660	SAMEA3732511	ERX1297997	ERR1225785	ITS	ITS2	CGTGTCTCTA	TCCTCCGCTTATTGATATGC	GCATCGATGAAGAACGCAGC	2011-07-05	49.07	-89.39	Canada	0.3	442	0.4	266	Gleyed Dystric Brunisol	Black Spruce	Dfb, Humid Continental warm summer	99	C0	0	A horizon	21.0	NA	NA	0.0	NA	NA
BL025F	BL025	PP_CA_	California	BL	Forest Soil	Whole Community DNA	PCR	Amplicon	pyrotag library	454 GS FLX Titanium	European Nucleotide Archive	https://www.ebi.ac.uk/ena/data/search?query=PRJEB12501	ERS1039661	SAMEA3732512	ERX1297998	ERR1225786	ITS	ITS2	ATATCGCGAG	TCCTCCGCTTATTGATATGC	GCATCGATGAAGAACGCAGC	2011-09-16	38.88	-120.64	USA	0.1	1350	11.2	55	Mesic Ultic Haploxeralfs	Ponderosa pine, sugar pine, white fir, giant sequoia	Csa, Mediterranean hot summer	OM1	C0	0	O horizon	18.0	NA	NA	4.8	NA	NA
BL026F	BL026	PP_CA_	California	BL	Forest Soil	Whole Community DNA	PCR	Amplicon	pyrotag library	454 GS FLX Titanium	European Nucleotide Archive	https://www.ebi.ac.uk/ena/data/search?query=PRJEB12501	ERS1039662	SAMEA3732513	ERX1297999	ERR1225787	ITS	ITS2	ACGCGAGTAT	TCCTCCGCTTATTGATATGC	GCATCGATGAAGAACGCAGC	2011-09-16	38.88	-120.64	USA	0.3	1350	11.2	55	Mesic Ultic Haploxeralfs	Ponderosa pine, sugar pine, white fir, giant sequoia	Csa, Mediterranean hot summer	OM1	C0	0	A horizon	22.0	5.5	0.3	5.7	NA	19.8
BL027F	BL027	PP_CA_	California	BL	Forest Soil	Whole Community DNA	PCR	Amplicon	pyrotag library	454 GS FLX Titanium	European Nucleotide Archive	https://www.ebi.ac.uk/ena/data/search?query=PRJEB12501	ERS1039663	SAMEA3732514	ERX1298000	ERR1225788	ITS	ITS2	TCTACGTAGC	TCCTCCGCTTATTGATATGC	GCATCGATGAAGAACGCAGC	2011-09-16	38.88	-120.64	USA	0.1	1350	11.2	55	Mesic Ultic Haploxeralfs	Ponderosa pine, sugar pine, white fir, giant sequoia	Csa, Mediterranean hot summer	OM1	C0	0	O horizon	17.0	NA	NA	4.9	NA	NA
BL028F	BL028	PP_CA_	California	BL	Forest Soil	Whole Community DNA	PCR	Amplicon	pyrotag library	454 GS FLX Titanium	European Nucleotide Archive	https://www.ebi.ac.uk/ena/data/search?query=PRJEB12501	ERS1039664	SAMEA3732515	ERX1298001	ERR1225789	ITS	ITS2	ATAGAGTACT	TCCTCCGCTTATTGATATGC	GCATCGATGAAGAACGCAGC	2011-09-16	38.88	-120.64	USA	0.3	1350	11.2	55	Mesic Ultic Haploxeralfs	Ponderosa pine, sugar pine, white fir, giant sequoia	Csa, Mediterranean hot summer	OM1	C0	0	A horizon	21.0	5.5	0.3	5.9	NA	19.8
BL029F	BL029	PP_CA_	California	BL	Forest Soil	Whole Community DNA	PCR	Amplicon	pyrotag library	454 GS FLX Titanium	European Nucleotide Archive	https://www.ebi.ac.uk/ena/data/search?query=PRJEB12501	ERS1039665	SAMEA3732516	ERX1298002	ERR1225790	ITS	ITS2	ACTACTATGT	TCCTCCGCTTATTGATATGC	GCATCGATGAAGAACGCAGC	2011-09-16	38.88	-120.64	USA	0.1	1350	11.2	55	Mesic Ultic Haploxeralfs	Ponderosa pine, sugar pine, white fir, giant sequoia	Csa, Mediterranean hot summer	OM1	C0	0	O horizon	19.0	NA	NA	4.7	NA	NA
BL030F	BL030	PP_CA_	California	BL	Forest Soil	Whole Community DNA	PCR	Amplicon	pyrotag library	454 GS FLX Titanium	European Nucleotide Archive	https://www.ebi.ac.uk/ena/data/search?query=PRJEB12501	ERS1039666	SAMEA3732517	ERX1298003	ERR1225791	ITS	ITS2	CGTGTCTCTA	TCCTCCGCTTATTGATATGC	GCATCGATGAAGAACGCAGC	2011-09-16	38.88	-120.64	USA	0.3	1350	11.2	55	Mesic Ultic Haploxeralfs	Ponderosa pine, sugar pine, white fir, giant sequoia	Csa, Mediterranean hot summer	OM1	C0	0	A horizon	24.0	5.5	0.3	5.9	NA	19.8
BL031F	BL031	PP_CA_	California	BL	Forest Soil	Whole Community DNA	PCR	Amplicon	pyrotag library	454 GS FLX Titanium	European Nucleotide Archive	https://www.ebi.ac.uk/ena/data/search?query=PRJEB12501	ERS1039667	SAMEA3732518	ERX1298004	ERR1225792	ITS	ITS2	TCGATCACGT	TCCTCCGCTTATTGATATGC	GCATCGATGAAGAACGCAGC	2011-09-16	38.88	-120.64	USA	0.1	1350	11.2	55	Mesic Ultic Haploxeralfs	Ponderosa pine, sugar pine, white fir, giant sequoia	Csa, Mediterranean hot summer	OM2	C0	0	O horizon	29.0	NA	NA	5.7	NA	NA
BL032F	BL032	PP_CA_	California	BL	Forest Soil	Whole Community DNA	PCR	Amplicon	pyrotag library	454 GS FLX Titanium	European Nucleotide Archive	https://www.ebi.ac.uk/ena/data/search?query=PRJEB12501	ERS1039668	SAMEA3732519	ERX1298005	ERR1225793	ITS	ITS2	ATCAGACACG	TCCTCCGCTTATTGATATGC	GCATCGATGAAGAACGCAGC	2011-09-16	38.88	-120.64	USA	0.3	1350	11.2	55	Mesic Ultic Haploxeralfs	Ponderosa pine, sugar pine, white fir, giant sequoia	Csa, Mediterranean hot summer	OM2	C0	0	A horizon	21.0	5.4	0.3	5.8	NA	20.6
BL033F	BL033	PP_CA_	California	BL	Forest Soil	Whole Community DNA	PCR	Amplicon	pyrotag library	454 GS FLX Titanium	European Nucleotide Archive	https://www.ebi.ac.uk/ena/data/search?query=PRJEB12501	ERS1039669	SAMEA3732520	ERX1298006	ERR1225794	ITS	ITS2	CAGTAGACGT	TCCTCCGCTTATTGATATGC	GCATCGATGAAGAACGCAGC	2011-09-16	38.88	-120.64	USA	0.1	1350	11.2	55	Mesic Ultic Haploxeralfs	Ponderosa pine, sugar pine, white fir, giant sequoia	Csa, Mediterranean hot summer	OM2	C0	0	O horizon	22.0	NA	NA	5.2	NA	NA
BL034F	BL034	PP_CA_	California	BL	Forest Soil	Whole Community DNA	PCR	Amplicon	pyrotag library	454 GS FLX Titanium	European Nucleotide Archive	https://www.ebi.ac.uk/ena/data/search?query=PRJEB12501	ERS1039670	SAMEA3732521	ERX1298007	ERR1225795	ITS	ITS2	CTCGCGTGTC	TCCTCCGCTTATTGATATGC	GCATCGATGAAGAACGCAGC	2011-09-16	38.88	-120.64	USA	0.3	1350	11.2	55	Mesic Ultic Haploxeralfs	Ponderosa pine, sugar pine, white fir, giant sequoia	Csa, Mediterranean hot summer	OM2	C0	0	A horizon	21.0	5.4	0.3	5.3	NA	20.6
BL035F	BL035	PP_CA_	California	BL	Forest Soil	Whole Community DNA	PCR	Amplicon	pyrotag library	454 GS FLX Titanium	European Nucleotide Archive	https://www.ebi.ac.uk/ena/data/search?query=PRJEB12501	ERS1039671	SAMEA3732522	ERX1298008	ERR1225796	ITS	ITS2	ACGCTCGACA	TCCTCCGCTTATTGATATGC	GCATCGATGAAGAACGCAGC	2011-09-16	38.88	-120.64	USA	0.1	1350	11.2	55	Mesic Ultic Haploxeralfs	Ponderosa pine, sugar pine, white fir, giant sequoia	Csa, Mediterranean hot summer	OM2	C0	0	O horizon	16.0	NA	NA	5.1	NA	NA
BL038F	BL038	PP_CA_	California	BL	Forest Soil	Whole Community DNA	PCR	Amplicon	pyrotag library	454 GS FLX Titanium	European Nucleotide Archive	https://www.ebi.ac.uk/ena/data/search?query=PRJEB12501	ERS1039672	SAMEA3732523	ERX1298009	ERR1225797	ITS	ITS2	CTCGCGTGTC	TCCTCCGCTTATTGATATGC	GCATCGATGAAGAACGCAGC	2011-09-16	38.88	-120.64	USA	0.3	1350	11.2	55	Mesic Ultic Haploxeralfs	Ponderosa pine, sugar pine, white fir, giant sequoia	Csa, Mediterranean hot summer	OM3	C0	0	A horizon	22.0	5.3	0.3	5.4	NA	18.9
BL039F	BL039	PP_CA_	California	BL	Forest Soil	Whole Community DNA	PCR	Amplicon	pyrotag library	454 GS FLX Titanium	European Nucleotide Archive	https://www.ebi.ac.uk/ena/data/search?query=PRJEB12501	ERS1039673	SAMEA3732524	ERX1298010	ERR1225798	ITS	ITS2	TGATACGTCT	TCCTCCGCTTATTGATATGC	GCATCGATGAAGAACGCAGC	2011-09-16	38.88	-120.64	USA	0.1	1350	11.2	55	Mesic Ultic Haploxeralfs	Ponderosa pine, sugar pine, white fir, giant sequoia	Csa, Mediterranean hot summer	OM3	C0	0	O horizon	24.0	NA	NA	5.3	NA	NA
BL041F	BL041	PP_CA_	California	BL	Forest Soil	Whole Community DNA	PCR	Amplicon	pyrotag library	454 GS FLX Titanium	European Nucleotide Archive	https://www.ebi.ac.uk/ena/data/search?query=PRJEB12501	ERS1039674	SAMEA3732525	ERX1298011	ERR1225799	ITS	ITS2	ACGCTCGACA	TCCTCCGCTTATTGATATGC	GCATCGATGAAGAACGCAGC	2011-09-16	38.88	-120.64	USA	0.1	1350	11.2	55	Mesic Ultic Haploxeralfs	Ponderosa pine, sugar pine, white fir, giant sequoia	Csa, Mediterranean hot summer	OM3	C0	0	O horizon	23.0	NA	NA	5.1	NA	NA
BL043F	BL043	PP_CA_	California	BL	Forest Soil	Whole Community DNA	PCR	Amplicon	pyrotag library	454 GS FLX Titanium	European Nucleotide Archive	https://www.ebi.ac.uk/ena/data/search?query=PRJEB12501	ERS1039675	SAMEA3732526	ERX1298012	ERR1225800	ITS	ITS2	TACACACACT	TCCTCCGCTTATTGATATGC	GCATCGATGAAGAACGCAGC	2011-09-16	38.88	-120.64	USA	0.1	1350	11.2	55	Mesic Ultic Haploxeralfs	Ponderosa pine, sugar pine, white fir, giant sequoia	Csa, Mediterranean hot summer	REF	REF	0	O horizon	27.0	NA	NA	4.5	NA	NA
BL044F	BL044	PP_CA_	California	BL	Forest Soil	Whole Community DNA	PCR	Amplicon	pyrotag library	454 GS FLX Titanium	European Nucleotide Archive	https://www.ebi.ac.uk/ena/data/search?query=PRJEB12501	ERS1039676	SAMEA3732527	ERX1298013	ERR1225801	ITS	ITS2	ATCAGACACG	TCCTCCGCTTATTGATATGC	GCATCGATGAAGAACGCAGC	2011-09-16	38.88	-120.64	USA	0.3	1350	11.2	55	Mesic Ultic Haploxeralfs	Ponderosa pine, sugar pine, white fir, giant sequoia	Csa, Mediterranean hot summer	REF	REF	0	A horizon	18.0	6.3	0.3	5.5	NA	20.3
BL046F	BL046	PP_CA_	California	BL	Forest Soil	Whole Community DNA	PCR	Amplicon	pyrotag library	454 GS FLX Titanium	European Nucleotide Archive	https://www.ebi.ac.uk/ena/data/search?query=PRJEB12501	ERS1039677	SAMEA3732528	ERX1298014	ERR1225802	ITS	ITS2	TCGATCACGT	TCCTCCGCTTATTGATATGC	GCATCGATGAAGAACGCAGC	2011-09-16	38.88	-120.64	USA	0.3	1350	11.2	55	Mesic Ultic Haploxeralfs	Ponderosa pine, sugar pine, white fir, giant sequoia	Csa, Mediterranean hot summer	REF	REF	0	A horizon	18.0	6.3	0.3	5.5	NA	20.3
BL047F	BL047	PP_CA_	California	BL	Forest Soil	Whole Community DNA	PCR	Amplicon	pyrotag library	454 GS FLX Titanium	European Nucleotide Archive	https://www.ebi.ac.uk/ena/data/search?query=PRJEB12501	ERS1039678	SAMEA3732529	ERX1298015	ERR1225803	ITS	ITS2	CACGCTACGT	TCCTCCGCTTATTGATATGC	GCATCGATGAAGAACGCAGC	2011-09-16	38.88	-120.64	USA	0.1	1350	11.2	55	Mesic Ultic Haploxeralfs	Ponderosa pine, sugar pine, white fir, giant sequoia	Csa, Mediterranean hot summer	REF	REF	0	O horizon	30.0	NA	NA	4.9	NA	NA
BL048F	BL048	PP_CA_	California	BL	Forest Soil	Whole Community DNA	PCR	Amplicon	pyrotag library	454 GS FLX Titanium	European Nucleotide Archive	https://www.ebi.ac.uk/ena/data/search?query=PRJEB12501	ERS1039679	SAMEA3732530	ERX1298016	ERR1225804	ITS	ITS2	CGAGAGATAC	TCCTCCGCTTATTGATATGC	GCATCGATGAAGAACGCAGC	2011-09-16	38.88	-120.64	USA	0.3	1350	11.2	55	Mesic Ultic Haploxeralfs	Ponderosa pine, sugar pine, white fir, giant sequoia	Csa, Mediterranean hot summer	REF	REF	0	A horizon	15.0	6.3	0.3	6.0	NA	20.3
BR049F	BR049	PP_CA_	California	BR	Forest Soil	Whole Community DNA	PCR	Amplicon	pyrotag library	454 GS FLX Titanium	European Nucleotide Archive	https://www.ebi.ac.uk/ena/data/search?query=PRJEB12501	ERS1039680	SAMEA3732531	ERX1298017	ERR1225805	ITS	ITS2	TCTCTATGCG	TCCTCCGCTTATTGATATGC	GCATCGATGAAGAACGCAGC	2011-06-22	39.55	-121.04	USA	0.1	1135	11.2	55	Mesic Ultic Haploxeralfs	Ponderosa pine, sugar pine, white fir, giant sequoia	Csa, Mediterranean hot summer	OM1	C0	0	O horizon	54.0	NA	NA	5.4	NA	NA
BR050F	BR050	PP_CA_	California	BR	Forest Soil	Whole Community DNA	PCR	Amplicon	pyrotag library	454 GS FLX Titanium	European Nucleotide Archive	https://www.ebi.ac.uk/ena/data/search?query=PRJEB12501	ERS1039681	SAMEA3732532	ERX1298018	ERR1225806	ITS	ITS2	CGAGAGATAC	TCCTCCGCTTATTGATATGC	GCATCGATGAAGAACGCAGC	2011-06-22	39.55	-121.04	USA	0.3	1135	11.2	55	Mesic Ultic Haploxeralfs	Ponderosa pine, sugar pine, white fir, giant sequoia	Csa, Mediterranean hot summer	OM1	C0	0	A horizon	35.0	6.4	0.3	5.9	NA	25.8
BR051F	BR051	PP_CA_	California	BR	Forest Soil	Whole Community DNA	PCR	Amplicon	pyrotag library	454 GS FLX Titanium	European Nucleotide Archive	https://www.ebi.ac.uk/ena/data/search?query=PRJEB12501	ERS1039682	SAMEA3732533	ERX1298019	ERR1225807	ITS	ITS2	ATATCGCGAG	TCCTCCGCTTATTGATATGC	GCATCGATGAAGAACGCAGC	2011-06-22	39.55	-121.04	USA	0.1	1135	11.2	55	Mesic Ultic Haploxeralfs	Ponderosa pine, sugar pine, white fir, giant sequoia	Csa, Mediterranean hot summer	OM1	C0	0	O horizon	55.0	NA	NA	4.3	NA	NA
BR052F	BR052	PP_CA_	California	BR	Forest Soil	Whole Community DNA	PCR	Amplicon	pyrotag library	454 GS FLX Titanium	European Nucleotide Archive	https://www.ebi.ac.uk/ena/data/search?query=PRJEB12501	ERS1039683	SAMEA3732534	ERX1298020	ERR1225808	ITS	ITS2	CGTGTCTCTA	TCCTCCGCTTATTGATATGC	GCATCGATGAAGAACGCAGC	2011-06-22	39.55	-121.04	USA	0.3	1135	11.2	55	Mesic Ultic Haploxeralfs	Ponderosa pine, sugar pine, white fir, giant sequoia	Csa, Mediterranean hot summer	OM1	C0	0	A horizon	34.0	6.4	0.3	5.2	NA	25.8
BR053F	BR053	PP_CA_	California	BR	Forest Soil	Whole Community DNA	PCR	Amplicon	pyrotag library	454 GS FLX Titanium	European Nucleotide Archive	https://www.ebi.ac.uk/ena/data/search?query=PRJEB12501	ERS1039684	SAMEA3732535	ERX1298021	ERR1225809	ITS	ITS2	AGTACGCTAT	TCCTCCGCTTATTGATATGC	GCATCGATGAAGAACGCAGC	2011-06-22	39.55	-121.04	USA	0.1	1135	11.2	55	Mesic Ultic Haploxeralfs	Ponderosa pine, sugar pine, white fir, giant sequoia	Csa, Mediterranean hot summer	OM1	C0	0	O horizon	58.0	NA	NA	4.4	NA	NA
BR054F	BR054	PP_CA_	California	BR	Forest Soil	Whole Community DNA	PCR	Amplicon	pyrotag library	454 GS FLX Titanium	European Nucleotide Archive	https://www.ebi.ac.uk/ena/data/search?query=PRJEB12501	ERS1039685	SAMEA3732536	ERX1298022	ERR1225810	ITS	ITS2	TCGATCACGT	TCCTCCGCTTATTGATATGC	GCATCGATGAAGAACGCAGC	2011-06-22	39.55	-121.04	USA	0.3	1135	11.2	55	Mesic Ultic Haploxeralfs	Ponderosa pine, sugar pine, white fir, giant sequoia	Csa, Mediterranean hot summer	OM1	C0	0	A horizon	32.0	6.4	0.3	5.3	NA	25.8
BR055F	BR055	PP_CA_	California	BR	Forest Soil	Whole Community DNA	PCR	Amplicon	pyrotag library	454 GS FLX Titanium	European Nucleotide Archive	https://www.ebi.ac.uk/ena/data/search?query=PRJEB12501	ERS1039686	SAMEA3732537	ERX1298023	ERR1225811	ITS	ITS2	TGATACGTCT	TCCTCCGCTTATTGATATGC	GCATCGATGAAGAACGCAGC	2011-06-22	39.55	-121.04	USA	0.1	1135	11.2	55	Mesic Ultic Haploxeralfs	Ponderosa pine, sugar pine, white fir, giant sequoia	Csa, Mediterranean hot summer	OM2	C0	0	O horizon	50.0	NA	NA	5.7	NA	NA
BR056F	BR056	PP_CA_	California	BR	Forest Soil	Whole Community DNA	PCR	Amplicon	pyrotag library	454 GS FLX Titanium	European Nucleotide Archive	https://www.ebi.ac.uk/ena/data/search?query=PRJEB12501	ERS1039687	SAMEA3732538	ERX1298024	ERR1225812	ITS	ITS2	ATATCGCGAG	TCCTCCGCTTATTGATATGC	GCATCGATGAAGAACGCAGC	2011-06-22	39.55	-121.04	USA	0.3	1135	11.2	55	Mesic Ultic Haploxeralfs	Ponderosa pine, sugar pine, white fir, giant sequoia	Csa, Mediterranean hot summer	OM2	C0	0	A horizon	36.0	5.7	0.3	6.1	NA	20.9
BR057F	BR057	PP_CA_	California	BR	Forest Soil	Whole Community DNA	PCR	Amplicon	pyrotag library	454 GS FLX Titanium	European Nucleotide Archive	https://www.ebi.ac.uk/ena/data/search?query=PRJEB12501	ERS1039688	SAMEA3732539	ERX1298025	ERR1225813	ITS	ITS2	ACGCTCGACA	TCCTCCGCTTATTGATATGC	GCATCGATGAAGAACGCAGC	2011-06-22	39.55	-121.04	USA	0.1	1135	11.2	55	Mesic Ultic Haploxeralfs	Ponderosa pine, sugar pine, white fir, giant sequoia	Csa, Mediterranean hot summer	OM2	C0	0	O horizon	51.0	NA	NA	5.0	NA	NA
BR058F	BR058	PP_CA_	California	BR	Forest Soil	Whole Community DNA	PCR	Amplicon	pyrotag library	454 GS FLX Titanium	European Nucleotide Archive	https://www.ebi.ac.uk/ena/data/search?query=PRJEB12501	ERS1039689	SAMEA3732540	ERX1298026	ERR1225814	ITS	ITS2	CATAGTAGTG	TCCTCCGCTTATTGATATGC	GCATCGATGAAGAACGCAGC	2011-06-22	39.55	-121.04	USA	0.3	1135	11.2	55	Mesic Ultic Haploxeralfs	Ponderosa pine, sugar pine, white fir, giant sequoia	Csa, Mediterranean hot summer	OM2	C0	0	A horizon	35.0	5.7	0.3	6.3	NA	20.9
BR059F	BR059	PP_CA_	California	BR	Forest Soil	Whole Community DNA	PCR	Amplicon	pyrotag library	454 GS FLX Titanium	European Nucleotide Archive	https://www.ebi.ac.uk/ena/data/search?query=PRJEB12501	ERS1039690	SAMEA3732541	ERX1298027	ERR1225815	ITS	ITS2	TACGCTGTCT	TCCTCCGCTTATTGATATGC	GCATCGATGAAGAACGCAGC	2011-06-22	39.55	-121.04	USA	0.1	1135	11.2	55	Mesic Ultic Haploxeralfs	Ponderosa pine, sugar pine, white fir, giant sequoia	Csa, Mediterranean hot summer	OM2	C0	0	O horizon	45.0	NA	NA	5.9	NA	NA
BR060F	BR060	PP_CA_	California	BR	Forest Soil	Whole Community DNA	PCR	Amplicon	pyrotag library	454 GS FLX Titanium	European Nucleotide Archive	https://www.ebi.ac.uk/ena/data/search?query=PRJEB12501	ERS1039691	SAMEA3732542	ERX1298028	ERR1225816	ITS	ITS2	TCTCTATGCG	TCCTCCGCTTATTGATATGC	GCATCGATGAAGAACGCAGC	2011-06-22	39.55	-121.04	USA	0.3	1135	11.2	55	Mesic Ultic Haploxeralfs	Ponderosa pine, sugar pine, white fir, giant sequoia	Csa, Mediterranean hot summer	OM2	C0	0	A horizon	37.0	5.7	0.3	6.1	NA	20.9
BR062F	BR062	PP_CA_	California	BR	Forest Soil	Whole Community DNA	PCR	Amplicon	pyrotag library	454 GS FLX Titanium	European Nucleotide Archive	https://www.ebi.ac.uk/ena/data/search?query=PRJEB12501	ERS1039692	SAMEA3732543	ERX1298029	ERR1225817	ITS	ITS2	CTCGCGTGTC	TCCTCCGCTTATTGATATGC	GCATCGATGAAGAACGCAGC	2011-06-22	39.55	-121.04	USA	0.3	1135	11.2	55	Mesic Ultic Haploxeralfs	Ponderosa pine, sugar pine, white fir, giant sequoia	Csa, Mediterranean hot summer	OM3	C0	0	A horizon	34.0	5.2	0.3	5.5	NA	19.2
BR063F	BR063	PP_CA_	California	BR	Forest Soil	Whole Community DNA	PCR	Amplicon	pyrotag library	454 GS FLX Titanium	European Nucleotide Archive	https://www.ebi.ac.uk/ena/data/search?query=PRJEB12501	ERS1039693	SAMEA3732544	ERX1298030	ERR1225818	ITS	ITS2	CGACGTGACT	TCCTCCGCTTATTGATATGC	GCATCGATGAAGAACGCAGC	2011-06-22	39.55	-121.04	USA	0.1	1135	11.2	55	Mesic Ultic Haploxeralfs	Ponderosa pine, sugar pine, white fir, giant sequoia	Csa, Mediterranean hot summer	OM3	C0	0	O horizon	50.0	NA	NA	4.8	NA	NA
BR064F	BR064	PP_CA_	California	BR	Forest Soil	Whole Community DNA	PCR	Amplicon	pyrotag library	454 GS FLX Titanium	European Nucleotide Archive	https://www.ebi.ac.uk/ena/data/search?query=PRJEB12501	ERS1039694	SAMEA3732545	ERX1298031	ERR1225819	ITS	ITS2	ATCAGACACG	TCCTCCGCTTATTGATATGC	GCATCGATGAAGAACGCAGC	2011-06-22	39.55	-121.04	USA	0.3	1135	11.2	55	Mesic Ultic Haploxeralfs	Ponderosa pine, sugar pine, white fir, giant sequoia	Csa, Mediterranean hot summer	OM3	C0	0	A horizon	33.0	5.2	0.3	5.4	NA	19.2
BR065F	BR065	PP_CA_	California	BR	Forest Soil	Whole Community DNA	PCR	Amplicon	pyrotag library	454 GS FLX Titanium	European Nucleotide Archive	https://www.ebi.ac.uk/ena/data/search?query=PRJEB12501	ERS1039695	SAMEA3732546	ERX1298032	ERR1225820	ITS	ITS2	AGCGTCGTCT	TCCTCCGCTTATTGATATGC	GCATCGATGAAGAACGCAGC	2011-06-22	39.55	-121.04	USA	0.1	1135	11.2	55	Mesic Ultic Haploxeralfs	Ponderosa pine, sugar pine, white fir, giant sequoia	Csa, Mediterranean hot summer	OM3	C0	0	O horizon	51.0	NA	NA	5.6	NA	NA
BR066F	BR066	PP_CA_	California	BR	Forest Soil	Whole Community DNA	PCR	Amplicon	pyrotag library	454 GS FLX Titanium	European Nucleotide Archive	https://www.ebi.ac.uk/ena/data/search?query=PRJEB12501	ERS1039696	SAMEA3732547	ERX1298033	ERR1225821	ITS	ITS2	AGTACGCTAT	TCCTCCGCTTATTGATATGC	GCATCGATGAAGAACGCAGC	2011-06-22	39.55	-121.04	USA	0.3	1135	11.2	55	Mesic Ultic Haploxeralfs	Ponderosa pine, sugar pine, white fir, giant sequoia	Csa, Mediterranean hot summer	OM3	C0	0	A horizon	35.0	5.2	0.3	5.8	NA	19.2
BR067F	BR067	PP_CA_	California	BR	Forest Soil	Whole Community DNA	PCR	Amplicon	pyrotag library	454 GS FLX Titanium	European Nucleotide Archive	https://www.ebi.ac.uk/ena/data/search?query=PRJEB12501	ERS1039697	SAMEA3732548	ERX1298034	ERR1225822	ITS	ITS2	TCTCTATGCG	TCCTCCGCTTATTGATATGC	GCATCGATGAAGAACGCAGC	2011-06-22	39.55	-121.04	USA	0.1	1135	11.2	55	Mesic Ultic Haploxeralfs	Ponderosa pine, sugar pine, white fir, giant sequoia	Csa, Mediterranean hot summer	REF	REF	0	O horizon	61.0	NA	NA	5.0	NA	NA
BR068F	BR068	PP_CA_	California	BR	Forest Soil	Whole Community DNA	PCR	Amplicon	pyrotag library	454 GS FLX Titanium	European Nucleotide Archive	https://www.ebi.ac.uk/ena/data/search?query=PRJEB12501	ERS1039698	SAMEA3732549	ERX1298035	ERR1225823	ITS	ITS2	ACGCGAGTAT	TCCTCCGCTTATTGATATGC	GCATCGATGAAGAACGCAGC	2011-06-22	39.55	-121.04	USA	0.3	1135	11.2	55	Mesic Ultic Haploxeralfs	Ponderosa pine, sugar pine, white fir, giant sequoia	Csa, Mediterranean hot summer	REF	REF	0	A horizon	28.0	5.8	0.3	6.0	NA	23.2
BR069F	BR069	PP_CA_	California	BR	Forest Soil	Whole Community DNA	PCR	Amplicon	pyrotag library	454 GS FLX Titanium	European Nucleotide Archive	https://www.ebi.ac.uk/ena/data/search?query=PRJEB12501	ERS1039699	SAMEA3732550	ERX1298036	ERR1225824	ITS	ITS2	TACGCTGTCT	TCCTCCGCTTATTGATATGC	GCATCGATGAAGAACGCAGC	2011-06-22	39.55	-121.04	USA	0.1	1135	11.2	55	Mesic Ultic Haploxeralfs	Ponderosa pine, sugar pine, white fir, giant sequoia	Csa, Mediterranean hot summer	REF	REF	0	O horizon	48.0	NA	NA	6.3	NA	NA
BR070F	BR070	PP_CA_	California	BR	Forest Soil	Whole Community DNA	PCR	Amplicon	pyrotag library	454 GS FLX Titanium	European Nucleotide Archive	https://www.ebi.ac.uk/ena/data/search?query=PRJEB12501	ERS1039700	SAMEA3732551	ERX1298037	ERR1225825	ITS	ITS2	ACTACTATGT	TCCTCCGCTTATTGATATGC	GCATCGATGAAGAACGCAGC	2011-06-22	39.55	-121.04	USA	0.3	1135	11.2	55	Mesic Ultic Haploxeralfs	Ponderosa pine, sugar pine, white fir, giant sequoia	Csa, Mediterranean hot summer	REF	REF	0	A horizon	36.0	5.8	0.3	6.7	NA	23.2
BR072F	BR072	PP_CA_	California	BR	Forest Soil	Whole Community DNA	PCR	Amplicon	pyrotag library	454 GS FLX Titanium	European Nucleotide Archive	https://www.ebi.ac.uk/ena/data/search?query=PRJEB12501	ERS1039701	SAMEA3732552	ERX1298038	ERR1225826	ITS	ITS2	ACGCTCGACA	TCCTCCGCTTATTGATATGC	GCATCGATGAAGAACGCAGC	2011-06-22	39.55	-121.04	USA	0.3	1135	11.2	55	Mesic Ultic Haploxeralfs	Ponderosa pine, sugar pine, white fir, giant sequoia	Csa, Mediterranean hot summer	REF	REF	0	A horizon	31.0	5.8	0.3	6.3	NA	23.2
JE085F	JE085	JP_ON_	Ontario	JE	Forest Soil	Whole Community DNA	PCR	Amplicon	pyrotag library	454 GS FLX Titanium	European Nucleotide Archive	https://www.ebi.ac.uk/ena/data/search?query=PRJEB12501	ERS1039702	SAMEA3732553	ERX1298039	ERR1225827	ITS	ITS2	CGAGAGATAC	TCCTCCGCTTATTGATATGC	GCATCGATGAAGAACGCAGC	2011-08-03	46.75	-82.25	Canada	0.1	490	2.8	242	NA	Jack Pine, Balsam fir, White birch	Dfb, Humid Continental cool summer	OM1	C0	1	O horizon	34.0	41.5	1.3	3.7	5.7	34.6
JE088F	JE088	JP_ON_	Ontario	JE	Forest Soil	Whole Community DNA	PCR	Amplicon	pyrotag library	454 GS FLX Titanium	European Nucleotide Archive	https://www.ebi.ac.uk/ena/data/search?query=PRJEB12501	ERS1039703	SAMEA3732554	ERX1298040	ERR1225828	ITS	ITS2	CGAGAGATAC	TCCTCCGCTTATTGATATGC	GCATCGATGAAGAACGCAGC	2011-08-03	46.75	-82.25	Canada	0.3	490	2.8	242	NA	Jack Pine, Balsam fir, White birch	Dfb, Humid Continental cool summer	OM1	C0	0	A horizon	10.0	2.6	0.2	5.0	5.7	15.9
JE090F	JE090	JP_ON_	Ontario	JE	Forest Soil	Whole Community DNA	PCR	Amplicon	pyrotag library	454 GS FLX Titanium	European Nucleotide Archive	https://www.ebi.ac.uk/ena/data/search?query=PRJEB12501	ERS1039704	SAMEA3732555	ERX1298041	ERR1225829	ITS	ITS2	TACAGATCGT	TCCTCCGCTTATTGATATGC	GCATCGATGAAGAACGCAGC	2011-08-03	46.75	-82.25	Canada	0.3	490	2.8	242	NA	Jack Pine, Balsam fir, White birch	Dfb, Humid Continental cool summer	OM3	C0	1	A horizon	8.0	3.0	0.2	5.0	5.7	16.4
JE092F	JE092	JP_ON_	Ontario	JE	Forest Soil	Whole Community DNA	PCR	Amplicon	pyrotag library	454 GS FLX Titanium	European Nucleotide Archive	https://www.ebi.ac.uk/ena/data/search?query=PRJEB12501	ERS1039705	SAMEA3732556	ERX1298042	ERR1225830	ITS	ITS2	TACGCTGTCT	TCCTCCGCTTATTGATATGC	GCATCGATGAAGAACGCAGC	2011-08-03	46.75	-82.25	Canada	0.3	490	2.8	242	NA	Jack Pine, Balsam fir, White birch	Dfb, Humid Continental cool summer	OM3	C0	0	A horizon	8.0	3.0	0.2	5.0	5.7	16.4
JE093F	JE093	JP_ON_	Ontario	JE	Forest Soil	Whole Community DNA	PCR	Amplicon	pyrotag library	454 GS FLX Titanium	European Nucleotide Archive	https://www.ebi.ac.uk/ena/data/search?query=PRJEB12501	ERS1039706	SAMEA3732557	ERX1298043	ERR1225831	ITS	ITS2	ATACGACGTA	TCCTCCGCTTATTGATATGC	GCATCGATGAAGAACGCAGC	2011-08-03	46.75	-82.25	Canada	0.1	490	2.8	242	NA	Jack Pine, Balsam fir, White birch	Dfb, Humid Continental cool summer	OM2	C0	1	O horizon	20.0	40.5	1.3	3.7	5.7	32.3
JE095F	JE095	JP_ON_	Ontario	JE	Forest Soil	Whole Community DNA	PCR	Amplicon	pyrotag library	454 GS FLX Titanium	European Nucleotide Archive	https://www.ebi.ac.uk/ena/data/search?query=PRJEB12501	ERS1039707	SAMEA3732558	ERX1298044	ERR1225832	ITS	ITS2	ACTGTACAGT	TCCTCCGCTTATTGATATGC	GCATCGATGAAGAACGCAGC	2011-08-03	46.75	-82.25	Canada	0.1	490	2.8	242	NA	Jack Pine, Balsam fir, White birch	Dfb, Humid Continental cool summer	OM2	C0	0	O horizon	32.0	40.5	1.3	3.7	5.7	32.3
JE096F	JE096	JP_ON_	Ontario	JE	Forest Soil	Whole Community DNA	PCR	Amplicon	pyrotag library	454 GS FLX Titanium	European Nucleotide Archive	https://www.ebi.ac.uk/ena/data/search?query=PRJEB12501	ERS1039708	SAMEA3732559	ERX1298045	ERR1225833	ITS	ITS2	AGCACTGTAG	TCCTCCGCTTATTGATATGC	GCATCGATGAAGAACGCAGC	2011-08-03	46.75	-82.25	Canada	0.3	490	2.8	242	NA	Jack Pine, Balsam fir, White birch	Dfb, Humid Continental cool summer	OM2	C0	0	A horizon	13.0	2.9	0.2	5.0	5.7	15.9
JE098F	JE098	JP_ON_	Ontario	JE	Forest Soil	Whole Community DNA	PCR	Amplicon	pyrotag library	454 GS FLX Titanium	European Nucleotide Archive	https://www.ebi.ac.uk/ena/data/search?query=PRJEB12501	ERS1039709	SAMEA3732560	ERX1298046	ERR1225834	ITS	ITS2	AGCGTCGTCT	TCCTCCGCTTATTGATATGC	GCATCGATGAAGAACGCAGC	2011-08-03	46.75	-82.25	Canada	0.3	490	2.8	242	NA	Jack Pine, Balsam fir, White birch	Dfb, Humid Continental cool summer	OM3	C0	1	A horizon	11.0	1.9	0.1	5.2	5.7	13.6
JE100F	JE100	JP_ON_	Ontario	JE	Forest Soil	Whole Community DNA	PCR	Amplicon	pyrotag library	454 GS FLX Titanium	European Nucleotide Archive	https://www.ebi.ac.uk/ena/data/search?query=PRJEB12501	ERS1039710	SAMEA3732561	ERX1298047	ERR1225835	ITS	ITS2	CTCGCGTGTC	TCCTCCGCTTATTGATATGC	GCATCGATGAAGAACGCAGC	2011-08-03	46.75	-82.25	Canada	0.3	490	2.8	242	NA	Jack Pine, Balsam fir, White birch	Dfb, Humid Continental cool summer	OM3	C0	0	A horizon	11.0	1.9	0.1	5.2	5.7	13.6
JE101F	JE101	JP_ON_	Ontario	JE	Forest Soil	Whole Community DNA	PCR	Amplicon	pyrotag library	454 GS FLX Titanium	European Nucleotide Archive	https://www.ebi.ac.uk/ena/data/search?query=PRJEB12501	ERS1039711	SAMEA3732562	ERX1298048	ERR1225836	ITS	ITS2	TCTACGTAGC	TCCTCCGCTTATTGATATGC	GCATCGATGAAGAACGCAGC	2011-08-03	46.75	-82.25	Canada	0.1	490	2.8	242	NA	Jack Pine, Balsam fir, White birch	Dfb, Humid Continental cool summer	OM2	C0	1	O horizon	31.0	38.5	1.2	3.6	5.7	32.7
JE102F	JE102	JP_ON_	Ontario	JE	Forest Soil	Whole Community DNA	PCR	Amplicon	pyrotag library	454 GS FLX Titanium	European Nucleotide Archive	https://www.ebi.ac.uk/ena/data/search?query=PRJEB12501	ERS1039712	SAMEA3732563	ERX1298049	ERR1225837	ITS	ITS2	CGACGTGACT	TCCTCCGCTTATTGATATGC	GCATCGATGAAGAACGCAGC	2011-08-03	46.75	-82.25	Canada	0.3	490	2.8	242	NA	Jack Pine, Balsam fir, White birch	Dfb, Humid Continental cool summer	OM2	C0	1	A horizon	7.0	2.1	0.2	5.1	5.7	13.5
JE103F	JE103	JP_ON_	Ontario	JE	Forest Soil	Whole Community DNA	PCR	Amplicon	pyrotag library	454 GS FLX Titanium	European Nucleotide Archive	https://www.ebi.ac.uk/ena/data/search?query=PRJEB12501	ERS1039713	SAMEA3732564	ERX1298050	ERR1225838	ITS	ITS2	ACGAGTGCGT	TCCTCCGCTTATTGATATGC	GCATCGATGAAGAACGCAGC	2011-08-03	46.75	-82.25	Canada	0.1	490	2.8	242	NA	Jack Pine, Balsam fir, White birch	Dfb, Humid Continental cool summer	OM2	C0	0	O horizon	24.0	38.5	1.2	3.6	5.7	32.7
JE104F	JE104	JP_ON_	Ontario	JE	Forest Soil	Whole Community DNA	PCR	Amplicon	pyrotag library	454 GS FLX Titanium	European Nucleotide Archive	https://www.ebi.ac.uk/ena/data/search?query=PRJEB12501	ERS1039714	SAMEA3732565	ERX1298051	ERR1225839	ITS	ITS2	CATAGTAGTG	TCCTCCGCTTATTGATATGC	GCATCGATGAAGAACGCAGC	2011-08-03	46.75	-82.25	Canada	0.3	490	2.8	242	NA	Jack Pine, Balsam fir, White birch	Dfb, Humid Continental cool summer	OM2	C0	0	A horizon	13.0	2.1	0.2	5.1	5.7	13.5
JE105F	JE105	JP_ON_	Ontario	JE	Forest Soil	Whole Community DNA	PCR	Amplicon	pyrotag library	454 GS FLX Titanium	European Nucleotide Archive	https://www.ebi.ac.uk/ena/data/search?query=PRJEB12501	ERS1039715	SAMEA3732566	ERX1298052	ERR1225840	ITS	ITS2	TACAGATCGT	TCCTCCGCTTATTGATATGC	GCATCGATGAAGAACGCAGC	2011-08-03	46.75	-82.25	Canada	0.1	490	2.8	242	NA	Jack Pine, Balsam fir, White birch	Dfb, Humid Continental cool summer	OM1	C0	1	O horizon	27.0	41.4	1.3	3.8	5.7	33.8
JE106F	JE106	JP_ON_	Ontario	JE	Forest Soil	Whole Community DNA	PCR	Amplicon	pyrotag library	454 GS FLX Titanium	European Nucleotide Archive	https://www.ebi.ac.uk/ena/data/search?query=PRJEB12501	ERS1039716	SAMEA3732567	ERX1298053	ERR1225841	ITS	ITS2	ATATCGCGAG	TCCTCCGCTTATTGATATGC	GCATCGATGAAGAACGCAGC	2011-08-03	46.75	-82.25	Canada	0.3	490	2.8	242	NA	Jack Pine, Balsam fir, White birch	Dfb, Humid Continental cool summer	OM1	C0	1	A horizon	7.0	2.4	0.2	5.1	5.7	13.9
JE107F	JE107	JP_ON_	Ontario	JE	Forest Soil	Whole Community DNA	PCR	Amplicon	pyrotag library	454 GS FLX Titanium	European Nucleotide Archive	https://www.ebi.ac.uk/ena/data/search?query=PRJEB12501	ERS1039717	SAMEA3732568	ERX1298054	ERR1225842	ITS	ITS2	ATAGAGTACT	TCCTCCGCTTATTGATATGC	GCATCGATGAAGAACGCAGC	2011-08-03	46.75	-82.25	Canada	0.1	490	2.8	242	NA	Jack Pine, Balsam fir, White birch	Dfb, Humid Continental cool summer	OM1	C0	0	O horizon	24.0	41.4	1.3	3.8	5.7	33.8
JE108F	JE108	JP_ON_	Ontario	JE	Forest Soil	Whole Community DNA	PCR	Amplicon	pyrotag library	454 GS FLX Titanium	European Nucleotide Archive	https://www.ebi.ac.uk/ena/data/search?query=PRJEB12501	ERS1039718	SAMEA3732569	ERX1298055	ERR1225843	ITS	ITS2	TCTCTATGCG	TCCTCCGCTTATTGATATGC	GCATCGATGAAGAACGCAGC	2011-08-03	46.75	-82.25	Canada	0.3	490	2.8	242	NA	Jack Pine, Balsam fir, White birch	Dfb, Humid Continental cool summer	OM1	C0	0	A horizon	12.0	2.4	0.2	5.1	5.7	13.9
JE110F	JE110	JP_ON_	Ontario	JE	Forest Soil	Whole Community DNA	PCR	Amplicon	pyrotag library	454 GS FLX Titanium	European Nucleotide Archive	https://www.ebi.ac.uk/ena/data/search?query=PRJEB12501	ERS1039719	SAMEA3732570	ERX1298056	ERR1225844	ITS	ITS2	CAGTAGACGT	TCCTCCGCTTATTGATATGC	GCATCGATGAAGAACGCAGC	2011-08-03	46.75	-82.25	Canada	0.3	490	2.8	242	NA	Jack Pine, Balsam fir, White birch	Dfb, Humid Continental cool summer	OM3	C0	1	A horizon	14.0	1.9	0.1	5.2	5.7	13.9
JE113F	JE113	JP_ON_	Ontario	JE	Forest Soil	Whole Community DNA	PCR	Amplicon	pyrotag library	454 GS FLX Titanium	European Nucleotide Archive	https://www.ebi.ac.uk/ena/data/search?query=PRJEB12501	ERS1039720	SAMEA3732571	ERX1298057	ERR1225845	ITS	ITS2	TCACGTACTA	TCCTCCGCTTATTGATATGC	GCATCGATGAAGAACGCAGC	2011-08-03	46.75	-82.25	Canada	0.1	490	2.8	242	NA	Jack Pine, Balsam fir, White birch	Dfb, Humid Continental cool summer	OM2	C0	1	O horizon	50.0	41.6	1.4	3.7	5.7	31.6
JE114F	JE114	JP_ON_	Ontario	JE	Forest Soil	Whole Community DNA	PCR	Amplicon	pyrotag library	454 GS FLX Titanium	European Nucleotide Archive	https://www.ebi.ac.uk/ena/data/search?query=PRJEB12501	ERS1039721	SAMEA3732572	ERX1298058	ERR1225846	ITS	ITS2	TACACACACT	TCCTCCGCTTATTGATATGC	GCATCGATGAAGAACGCAGC	2011-08-03	46.75	-82.25	Canada	0.3	490	2.8	242	NA	Jack Pine, Balsam fir, White birch	Dfb, Humid Continental cool summer	OM2	C0	1	A horizon	11.0	2.6	0.2	5.0	5.7	16.1
JE115F	JE115	JP_ON_	Ontario	JE	Forest Soil	Whole Community DNA	PCR	Amplicon	pyrotag library	454 GS FLX Titanium	European Nucleotide Archive	https://www.ebi.ac.uk/ena/data/search?query=PRJEB12501	ERS1039722	SAMEA3732573	ERX1298059	ERR1225847	ITS	ITS2	AGCGTCGTCT	TCCTCCGCTTATTGATATGC	GCATCGATGAAGAACGCAGC	2011-08-03	46.75	-82.25	Canada	0.1	490	2.8	242	NA	Jack Pine, Balsam fir, White birch	Dfb, Humid Continental cool summer	OM2	C0	0	O horizon	56.0	41.6	1.4	3.7	5.7	31.6
JE117F	JE117	JP_ON_	Ontario	JE	Forest Soil	Whole Community DNA	PCR	Amplicon	pyrotag library	454 GS FLX Titanium	European Nucleotide Archive	https://www.ebi.ac.uk/ena/data/search?query=PRJEB12501	ERS1039723	SAMEA3732574	ERX1298060	ERR1225848	ITS	ITS2	ACTACTATGT	TCCTCCGCTTATTGATATGC	GCATCGATGAAGAACGCAGC	2011-08-03	46.75	-82.25	Canada	0.1	490	2.8	242	NA	Jack Pine, Balsam fir, White birch	Dfb, Humid Continental cool summer	OM1	C0	1	O horizon	42.0	39.6	1.3	3.8	5.7	33.5
JE118F	JE118	JP_ON_	Ontario	JE	Forest Soil	Whole Community DNA	PCR	Amplicon	pyrotag library	454 GS FLX Titanium	European Nucleotide Archive	https://www.ebi.ac.uk/ena/data/search?query=PRJEB12501	ERS1039724	SAMEA3732575	ERX1298061	ERR1225849	ITS	ITS2	CGACGTGACT	TCCTCCGCTTATTGATATGC	GCATCGATGAAGAACGCAGC	2011-08-03	46.75	-82.25	Canada	0.3	490	2.8	242	NA	Jack Pine, Balsam fir, White birch	Dfb, Humid Continental cool summer	OM1	C0	1	A horizon	21.0	2.5	0.2	4.9	5.7	15.3
JE119F	JE119	JP_ON_	Ontario	JE	Forest Soil	Whole Community DNA	PCR	Amplicon	pyrotag library	454 GS FLX Titanium	European Nucleotide Archive	https://www.ebi.ac.uk/ena/data/search?query=PRJEB12501	ERS1039725	SAMEA3732576	ERX1298062	ERR1225850	ITS	ITS2	TACACACACT	TCCTCCGCTTATTGATATGC	GCATCGATGAAGAACGCAGC	2011-08-03	46.75	-82.25	Canada	0.1	490	2.8	242	NA	Jack Pine, Balsam fir, White birch	Dfb, Humid Continental cool summer	OM1	C0	0	O horizon	48.0	39.6	1.3	3.8	5.7	33.5
JE120F	JE120	JP_ON_	Ontario	JE	Forest Soil	Whole Community DNA	PCR	Amplicon	pyrotag library	454 GS FLX Titanium	European Nucleotide Archive	https://www.ebi.ac.uk/ena/data/search?query=PRJEB12501	ERS1039726	SAMEA3732577	ERX1298063	ERR1225851	ITS	ITS2	CGTCTAGTAC	TCCTCCGCTTATTGATATGC	GCATCGATGAAGAACGCAGC	2011-08-03	46.75	-82.25	Canada	0.3	490	2.8	242	NA	Jack Pine, Balsam fir, White birch	Dfb, Humid Continental cool summer	OM1	C0	0	A horizon	10.0	2.5	0.2	4.9	5.7	15.3
JE121F	JE121	JP_ON_	Ontario	JE	Forest Soil	Whole Community DNA	PCR	Amplicon	pyrotag library	454 GS FLX Titanium	European Nucleotide Archive	https://www.ebi.ac.uk/ena/data/search?query=PRJEB12501	ERS1039727	SAMEA3732578	ERX1298064	ERR1225852	ITS	ITS2	ATCAGACACG	TCCTCCGCTTATTGATATGC	GCATCGATGAAGAACGCAGC	2011-08-03	46.75	-82.25	Canada	0.1	490	2.8	242	NA	Jack Pine, Balsam fir, White birch	Dfb, Humid Continental cool summer	REF	REF	0	O horizon	69.0	43.2	1.4	3.6	5.7	30.8
JE122F	JE122	JP_ON_	Ontario	JE	Forest Soil	Whole Community DNA	PCR	Amplicon	pyrotag library	454 GS FLX Titanium	European Nucleotide Archive	https://www.ebi.ac.uk/ena/data/search?query=PRJEB12501	ERS1039728	SAMEA3732579	ERX1298065	ERR1225853	ITS	ITS2	AGCACTGTAG	TCCTCCGCTTATTGATATGC	GCATCGATGAAGAACGCAGC	2011-08-03	46.75	-82.25	Canada	0.3	490	2.8	242	NA	Jack Pine, Balsam fir, White birch	Dfb, Humid Continental cool summer	REF	REF	0	A horizon	15.0	2.4	0.2	4.9	5.7	16.4
JE123F	JE123	JP_ON_	Ontario	JE	Forest Soil	Whole Community DNA	PCR	Amplicon	pyrotag library	454 GS FLX Titanium	European Nucleotide Archive	https://www.ebi.ac.uk/ena/data/search?query=PRJEB12501	ERS1039729	SAMEA3732580	ERX1298066	ERR1225854	ITS	ITS2	CGTGTCTCTA	TCCTCCGCTTATTGATATGC	GCATCGATGAAGAACGCAGC	2011-08-03	46.75	-82.25	Canada	0.1	490	2.8	242	NA	Jack Pine, Balsam fir, White birch	Dfb, Humid Continental cool summer	REF	REF	0	O horizon	47.0	38.1	1.2	3.7	5.7	30.8
JE124F	JE124	JP_ON_	Ontario	JE	Forest Soil	Whole Community DNA	PCR	Amplicon	pyrotag library	454 GS FLX Titanium	European Nucleotide Archive	https://www.ebi.ac.uk/ena/data/search?query=PRJEB12501	ERS1039730	SAMEA3732581	ERX1298067	ERR1225855	ITS	ITS2	CATAGTAGTG	TCCTCCGCTTATTGATATGC	GCATCGATGAAGAACGCAGC	2011-08-03	46.75	-82.25	Canada	0.3	490	2.8	242	NA	Jack Pine, Balsam fir, White birch	Dfb, Humid Continental cool summer	REF	REF	0	A horizon	15.0	2.8	0.2	4.9	5.7	17.8
JE125F	JE125	JP_ON_	Ontario	JE	Forest Soil	Whole Community DNA	PCR	Amplicon	pyrotag library	454 GS FLX Titanium	European Nucleotide Archive	https://www.ebi.ac.uk/ena/data/search?query=PRJEB12501	ERS1039731	SAMEA3732582	ERX1298068	ERR1225856	ITS	ITS2	AGCGTCGTCT	TCCTCCGCTTATTGATATGC	GCATCGATGAAGAACGCAGC	2011-08-03	46.75	-82.25	Canada	0.1	490	2.8	242	NA	Jack Pine, Balsam fir, White birch	Dfb, Humid Continental cool summer	REF	REF	0	O horizon	74.0	38.9	1.3	3.6	5.7	29.8
JE126F	JE126	JP_ON_	Ontario	JE	Forest Soil	Whole Community DNA	PCR	Amplicon	pyrotag library	454 GS FLX Titanium	European Nucleotide Archive	https://www.ebi.ac.uk/ena/data/search?query=PRJEB12501	ERS1039732	SAMEA3732583	ERX1298069	ERR1225857	ITS	ITS2	CGTCTAGTAC	TCCTCCGCTTATTGATATGC	GCATCGATGAAGAACGCAGC	2011-08-03	46.75	-82.25	Canada	0.3	490	2.8	242	NA	Jack Pine, Balsam fir, White birch	Dfb, Humid Continental cool summer	REF	REF	0	A horizon	22.0	1.7	0.1	4.8	5.7	16.4
JS043F	JS043	JP_ON_	Ontario	JS	Forest Soil	Whole Community DNA	PCR	Amplicon	pyrotag library	454 GS FLX Titanium	European Nucleotide Archive	https://www.ebi.ac.uk/ena/data/search?query=PRJEB12501	ERS1039733	SAMEA3732584	ERX1298070	ERR1225858	ITS	ITS2	TCGATCACGT	TCCTCCGCTTATTGATATGC	GCATCGATGAAGAACGCAGC	2011-08-04	47.57	-82.85	Canada	0.1	426	1.7	250	Orthic Dystric Brunisol	Jack Pine, Black Spruce	Dfb, Humid Continental cool summer	OM1	C0	1	O horizon	43.0	44.5	1.1	4.0	7.9	39.6
JS044F	JS044	JP_ON_	Ontario	JS	Forest Soil	Whole Community DNA	PCR	Amplicon	pyrotag library	454 GS FLX Titanium	European Nucleotide Archive	https://www.ebi.ac.uk/ena/data/search?query=PRJEB12501	ERS1039734	SAMEA3732585	ERX1298071	ERR1225859	ITS	ITS2	TACACGTGAT	TCCTCCGCTTATTGATATGC	GCATCGATGAAGAACGCAGC	2011-08-04	47.57	-82.85	Canada	0.3	426	1.7	250	Orthic Dystric Brunisol	Jack Pine, Black Spruce	Dfb, Humid Continental cool summer	OM1	C0	1	A horizon	8.0	0.8	0.0	5.2	7.9	27.1
JS045F	JS045	JP_ON_	Ontario	JS	Forest Soil	Whole Community DNA	PCR	Amplicon	pyrotag library	454 GS FLX Titanium	European Nucleotide Archive	https://www.ebi.ac.uk/ena/data/search?query=PRJEB12501	ERS1039735	SAMEA3732586	ERX1298072	ERR1225860	ITS	ITS2	AGTACGCTAT	TCCTCCGCTTATTGATATGC	GCATCGATGAAGAACGCAGC	2011-08-04	47.57	-82.85	Canada	0.1	426	1.7	250	Orthic Dystric Brunisol	Jack Pine, Black Spruce	Dfb, Humid Continental cool summer	OM1	C0	0	O horizon	34.0	44.5	1.1	4.0	7.9	39.6
JS050F	JS050	JP_ON_	Ontario	JS	Forest Soil	Whole Community DNA	PCR	Amplicon	pyrotag library	454 GS FLX Titanium	European Nucleotide Archive	https://www.ebi.ac.uk/ena/data/search?query=PRJEB12501	ERS1039736	SAMEA3732587	ERX1298073	ERR1225861	ITS	ITS2	ACGCGAGTAT	TCCTCCGCTTATTGATATGC	GCATCGATGAAGAACGCAGC	2011-08-04	47.57	-82.85	Canada	0.3	426	1.7	250	Orthic Dystric Brunisol	Jack Pine, Black Spruce	Dfb, Humid Continental cool summer	OM3	C0	0	A horizon	3.0	0.4	0.0	5.5	7.9	26.6
JS051F	JS051	JP_ON_	Ontario	JS	Forest Soil	Whole Community DNA	PCR	Amplicon	pyrotag library	454 GS FLX Titanium	European Nucleotide Archive	https://www.ebi.ac.uk/ena/data/search?query=PRJEB12501	ERS1039737	SAMEA3732588	ERX1298074	ERR1225862	ITS	ITS2	ACGCTCGACA	TCCTCCGCTTATTGATATGC	GCATCGATGAAGAACGCAGC	2011-08-04	47.57	-82.85	Canada	0.1	426	1.7	250	Orthic Dystric Brunisol	Jack Pine, Black Spruce	Dfb, Humid Continental cool summer	OM2	C0	1	O horizon	47.0	43.1	1.1	3.9	7.9	38.9
JS052F	JS052	JP_ON_	Ontario	JS	Forest Soil	Whole Community DNA	PCR	Amplicon	pyrotag library	454 GS FLX Titanium	European Nucleotide Archive	https://www.ebi.ac.uk/ena/data/search?query=PRJEB12501	ERS1039738	SAMEA3732589	ERX1298075	ERR1225863	ITS	ITS2	ACGCGAGTAT	TCCTCCGCTTATTGATATGC	GCATCGATGAAGAACGCAGC	2011-08-04	47.57	-82.85	Canada	0.3	426	1.7	250	Orthic Dystric Brunisol	Jack Pine, Black Spruce	Dfb, Humid Continental cool summer	OM2	C0	1	A horizon	13.0	0.6	0.0	5.3	7.9	26.0
JS053F	JS053	JP_ON_	Ontario	JS	Forest Soil	Whole Community DNA	PCR	Amplicon	pyrotag library	454 GS FLX Titanium	European Nucleotide Archive	https://www.ebi.ac.uk/ena/data/search?query=PRJEB12501	ERS1039739	SAMEA3732590	ERX1298076	ERR1225864	ITS	ITS2	CGAGAGATAC	TCCTCCGCTTATTGATATGC	GCATCGATGAAGAACGCAGC	2011-08-04	47.57	-82.85	Canada	0.1	426	1.7	250	Orthic Dystric Brunisol	Jack Pine, Black Spruce	Dfb, Humid Continental cool summer	OM2	C0	0	O horizon	48.0	43.1	1.1	3.9	7.9	38.9
JS054F	JS054	JP_ON_	Ontario	JS	Forest Soil	Whole Community DNA	PCR	Amplicon	pyrotag library	454 GS FLX Titanium	European Nucleotide Archive	https://www.ebi.ac.uk/ena/data/search?query=PRJEB12501	ERS1039740	SAMEA3732591	ERX1298077	ERR1225865	ITS	ITS2	CATAGTAGTG	TCCTCCGCTTATTGATATGC	GCATCGATGAAGAACGCAGC	2011-08-04	47.57	-82.85	Canada	0.3	426	1.7	250	Orthic Dystric Brunisol	Jack Pine, Black Spruce	Dfb, Humid Continental cool summer	OM2	C0	0	A horizon	7.0	0.6	0.0	5.3	7.9	26.0
JS058F	JS058	JP_ON_	Ontario	JS	Forest Soil	Whole Community DNA	PCR	Amplicon	pyrotag library	454 GS FLX Titanium	European Nucleotide Archive	https://www.ebi.ac.uk/ena/data/search?query=PRJEB12501	ERS1039741	SAMEA3732592	ERX1298078	ERR1225866	ITS	ITS2	CGTCTAGTAC	TCCTCCGCTTATTGATATGC	GCATCGATGAAGAACGCAGC	2011-08-04	47.57	-82.85	Canada	0.3	426	1.7	250	Orthic Dystric Brunisol	Jack Pine, Black Spruce	Dfb, Humid Continental cool summer	OM3	C0	0	A horizon	12.0	12.7	0.3	5.1	7.9	31.3
JS059F	JS059	JP_ON_	Ontario	JS	Forest Soil	Whole Community DNA	PCR	Amplicon	pyrotag library	454 GS FLX Titanium	European Nucleotide Archive	https://www.ebi.ac.uk/ena/data/search?query=PRJEB12501	ERS1039742	SAMEA3732593	ERX1298079	ERR1225867	ITS	ITS2	CATAGTAGTG	TCCTCCGCTTATTGATATGC	GCATCGATGAAGAACGCAGC	2011-08-04	47.57	-82.85	Canada	0.1	426	1.7	250	Orthic Dystric Brunisol	Jack Pine, Black Spruce	Dfb, Humid Continental cool summer	OM2	C0	1	O horizon	45.0	42.7	1.0	4.0	7.9	42.6
JS060F	JS060	JP_ON_	Ontario	JS	Forest Soil	Whole Community DNA	PCR	Amplicon	pyrotag library	454 GS FLX Titanium	European Nucleotide Archive	https://www.ebi.ac.uk/ena/data/search?query=PRJEB12501	ERS1039743	SAMEA3732594	ERX1298080	ERR1225868	ITS	ITS2	CGTGTCTCTA	TCCTCCGCTTATTGATATGC	GCATCGATGAAGAACGCAGC	2011-08-04	47.57	-82.85	Canada	0.3	426	1.7	250	Orthic Dystric Brunisol	Jack Pine, Black Spruce	Dfb, Humid Continental cool summer	OM2	C0	1	A horizon	18.0	1.2	0.1	5.3	7.9	25.6
JS061F	JS061	JP_ON_	Ontario	JS	Forest Soil	Whole Community DNA	PCR	Amplicon	pyrotag library	454 GS FLX Titanium	European Nucleotide Archive	https://www.ebi.ac.uk/ena/data/search?query=PRJEB12501	ERS1039744	SAMEA3732595	ERX1298081	ERR1225869	ITS	ITS2	AGCGTCGTCT	TCCTCCGCTTATTGATATGC	GCATCGATGAAGAACGCAGC	2011-08-04	47.57	-82.85	Canada	0.1	426	1.7	250	Orthic Dystric Brunisol	Jack Pine, Black Spruce	Dfb, Humid Continental cool summer	OM2	C0	0	O horizon	42.0	42.7	1.0	4.0	7.9	42.6
JS062F	JS062	JP_ON_	Ontario	JS	Forest Soil	Whole Community DNA	PCR	Amplicon	pyrotag library	454 GS FLX Titanium	European Nucleotide Archive	https://www.ebi.ac.uk/ena/data/search?query=PRJEB12501	ERS1039745	SAMEA3732596	ERX1298082	ERR1225870	ITS	ITS2	TACGCTGTCT	TCCTCCGCTTATTGATATGC	GCATCGATGAAGAACGCAGC	2011-08-04	47.57	-82.85	Canada	0.3	426	1.7	250	Orthic Dystric Brunisol	Jack Pine, Black Spruce	Dfb, Humid Continental cool summer	OM2	C0	0	A horizon	7.0	1.2	0.1	5.3	7.9	25.6
JS063F	JS063	JP_ON_	Ontario	JS	Forest Soil	Whole Community DNA	PCR	Amplicon	pyrotag library	454 GS FLX Titanium	European Nucleotide Archive	https://www.ebi.ac.uk/ena/data/search?query=PRJEB12501	ERS1039746	SAMEA3732597	ERX1298083	ERR1225871	ITS	ITS2	CGACGTGACT	TCCTCCGCTTATTGATATGC	GCATCGATGAAGAACGCAGC	2011-08-04	47.57	-82.85	Canada	0.1	426	1.7	250	Orthic Dystric Brunisol	Jack Pine, Black Spruce	Dfb, Humid Continental cool summer	OM1	C0	1	O horizon	28.0	46.5	1.2	4.1	7.9	40.7
JS064F	JS064	JP_ON_	Ontario	JS	Forest Soil	Whole Community DNA	PCR	Amplicon	pyrotag library	454 GS FLX Titanium	European Nucleotide Archive	https://www.ebi.ac.uk/ena/data/search?query=PRJEB12501	ERS1039747	SAMEA3732598	ERX1298084	ERR1225872	ITS	ITS2	TCACGTACTA	TCCTCCGCTTATTGATATGC	GCATCGATGAAGAACGCAGC	2011-08-04	47.57	-82.85	Canada	0.3	426	1.7	250	Orthic Dystric Brunisol	Jack Pine, Black Spruce	Dfb, Humid Continental cool summer	OM1	C0	1	A horizon	6.0	1.0	0.0	5.2	7.9	24.1
JS065F	JS065	JP_ON_	Ontario	JS	Forest Soil	Whole Community DNA	PCR	Amplicon	pyrotag library	454 GS FLX Titanium	European Nucleotide Archive	https://www.ebi.ac.uk/ena/data/search?query=PRJEB12501	ERS1039748	SAMEA3732599	ERX1298085	ERR1225873	ITS	ITS2	TCACGTACTA	TCCTCCGCTTATTGATATGC	GCATCGATGAAGAACGCAGC	2011-08-04	47.57	-82.85	Canada	0.1	426	1.7	250	Orthic Dystric Brunisol	Jack Pine, Black Spruce	Dfb, Humid Continental cool summer	OM1	C0	0	O horizon	36.0	46.5	1.2	4.1	7.9	40.7
JS066F	JS066	JP_ON_	Ontario	JS	Forest Soil	Whole Community DNA	PCR	Amplicon	pyrotag library	454 GS FLX Titanium	European Nucleotide Archive	https://www.ebi.ac.uk/ena/data/search?query=PRJEB12501	ERS1039749	SAMEA3732600	ERX1298086	ERR1225874	ITS	ITS2	ACGAGTGCGT	TCCTCCGCTTATTGATATGC	GCATCGATGAAGAACGCAGC	2011-08-04	47.57	-82.85	Canada	0.3	426	1.7	250	Orthic Dystric Brunisol	Jack Pine, Black Spruce	Dfb, Humid Continental cool summer	OM1	C0	0	A horizon	8.0	1.0	0.0	5.2	7.9	24.1
JS067F	JS067	JP_ON_	Ontario	JS	Forest Soil	Whole Community DNA	PCR	Amplicon	pyrotag library	454 GS FLX Titanium	European Nucleotide Archive	https://www.ebi.ac.uk/ena/data/search?query=PRJEB12501	ERS1039750	SAMEA3732601	ERX1298087	ERR1225875	ITS	ITS2	CAGTAGACGT	TCCTCCGCTTATTGATATGC	GCATCGATGAAGAACGCAGC	2011-08-04	47.57	-82.85	Canada	0.1	426	1.7	250	Orthic Dystric Brunisol	Jack Pine, Black Spruce	Dfb, Humid Continental cool summer	OM2	C0	1	O horizon	29.0	42.1	0.9	3.9	7.9	46.1
JS068F	JS068	JP_ON_	Ontario	JS	Forest Soil	Whole Community DNA	PCR	Amplicon	pyrotag library	454 GS FLX Titanium	European Nucleotide Archive	https://www.ebi.ac.uk/ena/data/search?query=PRJEB12501	ERS1039751	SAMEA3732602	ERX1298088	ERR1225876	ITS	ITS2	AGACGCACTC	TCCTCCGCTTATTGATATGC	GCATCGATGAAGAACGCAGC	2011-08-04	47.57	-82.85	Canada	0.3	426	1.7	250	Orthic Dystric Brunisol	Jack Pine, Black Spruce	Dfb, Humid Continental cool summer	OM2	C0	1	A horizon	14.0	0.8	0.0	5.2	7.9	26.8
JS072F	JS072	JP_ON_	Ontario	JS	Forest Soil	Whole Community DNA	PCR	Amplicon	pyrotag library	454 GS FLX Titanium	European Nucleotide Archive	https://www.ebi.ac.uk/ena/data/search?query=PRJEB12501	ERS1039752	SAMEA3732603	ERX1298089	ERR1225877	ITS	ITS2	TGATACGTCT	TCCTCCGCTTATTGATATGC	GCATCGATGAAGAACGCAGC	2011-08-04	47.57	-82.85	Canada	0.3	426	1.7	250	Orthic Dystric Brunisol	Jack Pine, Black Spruce	Dfb, Humid Continental cool summer	OM3	C0	1	A horizon	9.0	0.4	0.0	5.3	7.9	24.1
JS074F	JS074	JP_ON_	Ontario	JS	Forest Soil	Whole Community DNA	PCR	Amplicon	pyrotag library	454 GS FLX Titanium	European Nucleotide Archive	https://www.ebi.ac.uk/ena/data/search?query=PRJEB12501	ERS1039753	SAMEA3732604	ERX1298090	ERR1225878	ITS	ITS2	CGTGTCTCTA	TCCTCCGCTTATTGATATGC	GCATCGATGAAGAACGCAGC	2011-08-04	47.57	-82.85	Canada	0.3	426	1.7	250	Orthic Dystric Brunisol	Jack Pine, Black Spruce	Dfb, Humid Continental cool summer	OM3	C0	0	A horizon	6.0	0.4	0.0	5.3	7.9	24.1
JS075F	JS075	JP_ON_	Ontario	JS	Forest Soil	Whole Community DNA	PCR	Amplicon	pyrotag library	454 GS FLX Titanium	European Nucleotide Archive	https://www.ebi.ac.uk/ena/data/search?query=PRJEB12501	ERS1039754	SAMEA3732605	ERX1298091	ERR1225879	ITS	ITS2	TACACGTGAT	TCCTCCGCTTATTGATATGC	GCATCGATGAAGAACGCAGC	2011-08-04	47.57	-82.85	Canada	0.1	426	1.7	250	Orthic Dystric Brunisol	Jack Pine, Black Spruce	Dfb, Humid Continental cool summer	OM1	C0	1	O horizon	32.0	43.4	1.1	3.9	7.9	40.0
JS076F	JS076	JP_ON_	Ontario	JS	Forest Soil	Whole Community DNA	PCR	Amplicon	pyrotag library	454 GS FLX Titanium	European Nucleotide Archive	https://www.ebi.ac.uk/ena/data/search?query=PRJEB12501	ERS1039755	SAMEA3732606	ERX1298092	ERR1225880	ITS	ITS2	CACGCTACGT	TCCTCCGCTTATTGATATGC	GCATCGATGAAGAACGCAGC	2011-08-04	47.57	-82.85	Canada	0.3	426	1.7	250	Orthic Dystric Brunisol	Jack Pine, Black Spruce	Dfb, Humid Continental cool summer	OM1	C0	1	A horizon	6.0	0.8	0.0	5.3	7.9	23.0
JS077F	JS077	JP_ON_	Ontario	JS	Forest Soil	Whole Community DNA	PCR	Amplicon	pyrotag library	454 GS FLX Titanium	European Nucleotide Archive	https://www.ebi.ac.uk/ena/data/search?query=PRJEB12501	ERS1039756	SAMEA3732607	ERX1298093	ERR1225881	ITS	ITS2	TCACGTACTA	TCCTCCGCTTATTGATATGC	GCATCGATGAAGAACGCAGC	2011-08-04	47.57	-82.85	Canada	0.1	426	1.7	250	Orthic Dystric Brunisol	Jack Pine, Black Spruce	Dfb, Humid Continental cool summer	OM1	C0	0	O horizon	34.0	43.4	1.1	3.9	7.9	40.0
JS078F	JS078	JP_ON_	Ontario	JS	Forest Soil	Whole Community DNA	PCR	Amplicon	pyrotag library	454 GS FLX Titanium	European Nucleotide Archive	https://www.ebi.ac.uk/ena/data/search?query=PRJEB12501	ERS1039757	SAMEA3732608	ERX1298094	ERR1225882	ITS	ITS2	CAGTAGACGT	TCCTCCGCTTATTGATATGC	GCATCGATGAAGAACGCAGC	2011-08-04	47.57	-82.85	Canada	0.3	426	1.7	250	Orthic Dystric Brunisol	Jack Pine, Black Spruce	Dfb, Humid Continental cool summer	OM1	C0	0	A horizon	4.0	0.8	0.0	5.3	7.9	23.0
JS079F	JS079	JP_ON_	Ontario	JS	Forest Soil	Whole Community DNA	PCR	Amplicon	pyrotag library	454 GS FLX Titanium	European Nucleotide Archive	https://www.ebi.ac.uk/ena/data/search?query=PRJEB12501	ERS1039758	SAMEA3732609	ERX1298095	ERR1225883	ITS	ITS2	ACTGTACAGT	TCCTCCGCTTATTGATATGC	GCATCGATGAAGAACGCAGC	2011-08-04	47.57	-82.85	Canada	0.1	426	1.7	250	Orthic Dystric Brunisol	Jack Pine, Black Spruce	Dfb, Humid Continental cool summer	REF	REF	0	O horizon	47.0	45.7	1.3	3.7	7.9	36.6
JS080F	JS080	JP_ON_	Ontario	JS	Forest Soil	Whole Community DNA	PCR	Amplicon	pyrotag library	454 GS FLX Titanium	European Nucleotide Archive	https://www.ebi.ac.uk/ena/data/search?query=PRJEB12501	ERS1039759	SAMEA3732610	ERX1298096	ERR1225884	ITS	ITS2	CGTCTAGTAC	TCCTCCGCTTATTGATATGC	GCATCGATGAAGAACGCAGC	2011-08-04	47.57	-82.85	Canada	0.3	426	1.7	250	Orthic Dystric Brunisol	Jack Pine, Black Spruce	Dfb, Humid Continental cool summer	REF	REF	0	A horizon	16.0	1.0	0.1	5.2	7.9	16.4
JS081F	JS081	JP_ON_	Ontario	JS	Forest Soil	Whole Community DNA	PCR	Amplicon	pyrotag library	454 GS FLX Titanium	European Nucleotide Archive	https://www.ebi.ac.uk/ena/data/search?query=PRJEB12501	ERS1039760	SAMEA3732611	ERX1298097	ERR1225885	ITS	ITS2	ATATCGCGAG	TCCTCCGCTTATTGATATGC	GCATCGATGAAGAACGCAGC	2011-08-04	47.57	-82.85	Canada	0.1	426	1.7	250	Orthic Dystric Brunisol	Jack Pine, Black Spruce	Dfb, Humid Continental cool summer	REF	REF	0	O horizon	45.0	44.8	1.3	3.8	7.8	34.7
JS082F	JS082	JP_ON_	Ontario	JS	Forest Soil	Whole Community DNA	PCR	Amplicon	pyrotag library	454 GS FLX Titanium	European Nucleotide Archive	https://www.ebi.ac.uk/ena/data/search?query=PRJEB12501	ERS1039761	SAMEA3732612	ERX1298098	ERR1225886	ITS	ITS2	AGTACGCTAT	TCCTCCGCTTATTGATATGC	GCATCGATGAAGAACGCAGC	2011-08-04	47.57	-82.85	Canada	0.3	426	1.7	250	Orthic Dystric Brunisol	Jack Pine, Black Spruce	Dfb, Humid Continental cool summer	REF	REF	0	A horizon	17.0	1.0	0.0	5.1	7.8	23.8
JS083F	JS083	JP_ON_	Ontario	JS	Forest Soil	Whole Community DNA	PCR	Amplicon	pyrotag library	454 GS FLX Titanium	European Nucleotide Archive	https://www.ebi.ac.uk/ena/data/search?query=PRJEB12501	ERS1039762	SAMEA3732613	ERX1298099	ERR1225887	ITS	ITS2	AGACGCACTC	TCCTCCGCTTATTGATATGC	GCATCGATGAAGAACGCAGC	2011-08-04	47.57	-82.85	Canada	0.1	426	1.7	250	Orthic Dystric Brunisol	Jack Pine, Black Spruce	Dfb, Humid Continental cool summer	REF	REF	0	O horizon	49.0	44.6	1.2	3.6	7.8	35.9
JS084F	JS084	JP_ON_	Ontario	JS	Forest Soil	Whole Community DNA	PCR	Amplicon	pyrotag library	454 GS FLX Titanium	European Nucleotide Archive	https://www.ebi.ac.uk/ena/data/search?query=PRJEB12501	ERS1039763	SAMEA3732614	ERX1298100	ERR1225888	ITS	ITS2	TACGCTGTCT	TCCTCCGCTTATTGATATGC	GCATCGATGAAGAACGCAGC	2011-08-04	47.57	-82.85	Canada	0.3	426	1.7	250	Orthic Dystric Brunisol	Jack Pine, Black Spruce	Dfb, Humid Continental cool summer	REF	REF	0	A horizon	12.0	1.2	0.1	5.0	7.8	23.2
JW002F	JW002	JP_ON_	Ontario	JW	Forest Soil	Whole Community DNA	PCR	Amplicon	pyrotag library	454 GS FLX Titanium	European Nucleotide Archive	https://www.ebi.ac.uk/ena/data/search?query=PRJEB12501	ERS1039764	SAMEA3732615	ERX1298101	ERR1225889	ITS	ITS2	CTCGCGTGTC	TCCTCCGCTTATTGATATGC	GCATCGATGAAGAACGCAGC	2011-07-07	46.42	-83.37	Canada	0.3	228	NA	248	Orthic Humo-Ferric Podzol	Jack Pine, Black Spruce, Red Pine	Dfb, Humid Continental cool summer	OM3	C0	1	A horizon	12.0	2.2	0.1	5.4	7.2	17.8
JW003F	JW003	JP_ON_	Ontario	JW	Forest Soil	Whole Community DNA	PCR	Amplicon	pyrotag library	454 GS FLX Titanium	European Nucleotide Archive	https://www.ebi.ac.uk/ena/data/search?query=PRJEB12501	ERS1039765	SAMEA3732616	ERX1298102	ERR1225890	ITS	ITS2	TCTACGTAGC	TCCTCCGCTTATTGATATGC	GCATCGATGAAGAACGCAGC	2011-07-07	46.42	-83.37	Canada	0.1	228	NA	248	Orthic Humo-Ferric Podzol	Jack Pine, Black Spruce, Red Pine	Dfb, Humid Continental cool summer	OM3	C0	0	O horizon	37.0	38.3	1.1	4.1	7.2	37.3
JW004F	JW004	JP_ON_	Ontario	JW	Forest Soil	Whole Community DNA	PCR	Amplicon	pyrotag library	454 GS FLX Titanium	European Nucleotide Archive	https://www.ebi.ac.uk/ena/data/search?query=PRJEB12501	ERS1039766	SAMEA3732617	ERX1298103	ERR1225891	ITS	ITS2	ACGCGAGTAT	TCCTCCGCTTATTGATATGC	GCATCGATGAAGAACGCAGC	2011-07-07	46.42	-83.37	Canada	0.3	228	NA	248	Orthic Humo-Ferric Podzol	Jack Pine, Black Spruce, Red Pine	Dfb, Humid Continental cool summer	OM3	C0	0	A horizon	11.0	2.2	0.1	5.4	7.2	17.8
JW005F	JW005	JP_ON_	Ontario	JW	Forest Soil	Whole Community DNA	PCR	Amplicon	pyrotag library	454 GS FLX Titanium	European Nucleotide Archive	https://www.ebi.ac.uk/ena/data/search?query=PRJEB12501	ERS1039767	SAMEA3732618	ERX1298104	ERR1225892	ITS	ITS2	ACGCGAGTAT	TCCTCCGCTTATTGATATGC	GCATCGATGAAGAACGCAGC	2011-07-07	46.42	-83.37	Canada	0.1	228	NA	248	Orthic Humo-Ferric Podzol	Jack Pine, Black Spruce, Red Pine	Dfb, Humid Continental cool summer	OM1	C0	1	O horizon	38.1	36.4	1.1	4.2	6.3	33.0
JW006F	JW006	JP_ON_	Ontario	JW	Forest Soil	Whole Community DNA	PCR	Amplicon	pyrotag library	454 GS FLX Titanium	European Nucleotide Archive	https://www.ebi.ac.uk/ena/data/search?query=PRJEB12501	ERS1039768	SAMEA3732619	ERX1298105	ERR1225893	ITS	ITS2	ACTGTACAGT	TCCTCCGCTTATTGATATGC	GCATCGATGAAGAACGCAGC	2011-07-07	46.42	-83.37	Canada	0.3	228	NA	248	Orthic Humo-Ferric Podzol	Jack Pine, Black Spruce, Red Pine	Dfb, Humid Continental cool summer	OM1	C0	1	A horizon	16.0	2.7	0.2	5.3	6.3	16.0
JW007F	JW007	JP_ON_	Ontario	JW	Forest Soil	Whole Community DNA	PCR	Amplicon	pyrotag library	454 GS FLX Titanium	European Nucleotide Archive	https://www.ebi.ac.uk/ena/data/search?query=PRJEB12501	ERS1039769	SAMEA3732620	ERX1298106	ERR1225894	ITS	ITS2	TCTACGTAGC	TCCTCCGCTTATTGATATGC	GCATCGATGAAGAACGCAGC	2011-07-07	46.42	-83.37	Canada	0.1	228	NA	248	Orthic Humo-Ferric Podzol	Jack Pine, Black Spruce, Red Pine	Dfb, Humid Continental cool summer	OM1	C0	0	O horizon	40.0	36.4	1.1	4.2	6.3	33.0
JW008F	JW008	JP_ON_	Ontario	JW	Forest Soil	Whole Community DNA	PCR	Amplicon	pyrotag library	454 GS FLX Titanium	European Nucleotide Archive	https://www.ebi.ac.uk/ena/data/search?query=PRJEB12501	ERS1039770	SAMEA3732621	ERX1298107	ERR1225895	ITS	ITS2	AGTACGCTAT	TCCTCCGCTTATTGATATGC	GCATCGATGAAGAACGCAGC	2011-07-07	46.42	-83.37	Canada	0.3	228	NA	248	Orthic Humo-Ferric Podzol	Jack Pine, Black Spruce, Red Pine	Dfb, Humid Continental cool summer	OM1	C0	0	A horizon	10.0	2.7	0.2	5.3	6.3	16.0
JW010F	JW010	JP_ON_	Ontario	JW	Forest Soil	Whole Community DNA	PCR	Amplicon	pyrotag library	454 GS FLX Titanium	European Nucleotide Archive	https://www.ebi.ac.uk/ena/data/search?query=PRJEB12501	ERS1039771	SAMEA3732622	ERX1298108	ERR1225896	ITS	ITS2	CGTCTAGTAC	TCCTCCGCTTATTGATATGC	GCATCGATGAAGAACGCAGC	2011-07-07	46.42	-83.37	Canada	0.3	228	NA	248	Orthic Humo-Ferric Podzol	Jack Pine, Black Spruce, Red Pine	Dfb, Humid Continental cool summer	OM3	C0	1	A horizon	18.0	1.5	0.1	5.5	8.9	15.6
JW012F	JW012	JP_ON_	Ontario	JW	Forest Soil	Whole Community DNA	PCR	Amplicon	pyrotag library	454 GS FLX Titanium	European Nucleotide Archive	https://www.ebi.ac.uk/ena/data/search?query=PRJEB12501	ERS1039772	SAMEA3732623	ERX1298109	ERR1225897	ITS	ITS2	CGAGAGATAC	TCCTCCGCTTATTGATATGC	GCATCGATGAAGAACGCAGC	2011-07-07	46.42	-83.37	Canada	0.3	228	NA	248	Orthic Humo-Ferric Podzol	Jack Pine, Black Spruce, Red Pine	Dfb, Humid Continental cool summer	OM3	C0	0	A horizon	13.0	1.5	0.1	5.5	8.9	15.6
JW013F	JW013	JP_ON_	Ontario	JW	Forest Soil	Whole Community DNA	PCR	Amplicon	pyrotag library	454 GS FLX Titanium	European Nucleotide Archive	https://www.ebi.ac.uk/ena/data/search?query=PRJEB12501	ERS1039773	SAMEA3732624	ERX1298110	ERR1225898	ITS	ITS2	TAGTGTAGAT	TCCTCCGCTTATTGATATGC	GCATCGATGAAGAACGCAGC	2011-07-07	46.42	-83.37	Canada	0.1	228	NA	248	Orthic Humo-Ferric Podzol	Jack Pine, Black Spruce, Red Pine	Dfb, Humid Continental cool summer	REF	REF	0	O horizon	31.0	38.1	1.1	4.1	8.9	36.6
JW014F	JW014	JP_ON_	Ontario	JW	Forest Soil	Whole Community DNA	PCR	Amplicon	pyrotag library	454 GS FLX Titanium	European Nucleotide Archive	https://www.ebi.ac.uk/ena/data/search?query=PRJEB12501	ERS1039774	SAMEA3732625	ERX1298111	ERR1225899	ITS	ITS2	TCTACGTAGC	TCCTCCGCTTATTGATATGC	GCATCGATGAAGAACGCAGC	2011-07-07	46.42	-83.37	Canada	0.3	228	NA	248	Orthic Humo-Ferric Podzol	Jack Pine, Black Spruce, Red Pine	Dfb, Humid Continental cool summer	REF	REF	0	A horizon	16.0	1.5	0.1	5.5	8.9	15.6
JW017F	JW017	JP_ON_	Ontario	JW	Forest Soil	Whole Community DNA	PCR	Amplicon	pyrotag library	454 GS FLX Titanium	European Nucleotide Archive	https://www.ebi.ac.uk/ena/data/search?query=PRJEB12501	ERS1039775	SAMEA3732626	ERX1298112	ERR1225900	ITS	ITS2	ACTACTATGT	TCCTCCGCTTATTGATATGC	GCATCGATGAAGAACGCAGC	2011-07-07	46.42	-83.37	Canada	0.1	228	NA	248	Orthic Humo-Ferric Podzol	Jack Pine, Black Spruce, Red Pine	Dfb, Humid Continental cool summer	OM2	C0	0	O horizon	47.0	36.9	1.2	4.2	9.8	30.8
JW020F	JW020	JP_ON_	Ontario	JW	Forest Soil	Whole Community DNA	PCR	Amplicon	pyrotag library	454 GS FLX Titanium	European Nucleotide Archive	https://www.ebi.ac.uk/ena/data/search?query=PRJEB12501	ERS1039776	SAMEA3732627	ERX1298113	ERR1225901	ITS	ITS2	CATAGTAGTG	TCCTCCGCTTATTGATATGC	GCATCGATGAAGAACGCAGC	2011-07-07	46.42	-83.37	Canada	0.3	228	NA	248	Orthic Humo-Ferric Podzol	Jack Pine, Black Spruce, Red Pine	Dfb, Humid Continental cool summer	REF	REF	0	A horizon	12.0	2.6	0.2	5.4	9.8	16.2
JW022F	JW022	JP_ON_	Ontario	JW	Forest Soil	Whole Community DNA	PCR	Amplicon	pyrotag library	454 GS FLX Titanium	European Nucleotide Archive	https://www.ebi.ac.uk/ena/data/search?query=PRJEB12501	ERS1039777	SAMEA3732628	ERX1298114	ERR1225902	ITS	ITS2	ATACGACGTA	TCCTCCGCTTATTGATATGC	GCATCGATGAAGAACGCAGC	2011-07-07	46.42	-83.37	Canada	0.3	228	NA	248	Orthic Humo-Ferric Podzol	Jack Pine, Black Spruce, Red Pine	Dfb, Humid Continental cool summer	OM1	C0	1	A horizon	14.0	3.1	0.2	5.3	7.7	14.8
JW023F	JW023	JP_ON_	Ontario	JW	Forest Soil	Whole Community DNA	PCR	Amplicon	pyrotag library	454 GS FLX Titanium	European Nucleotide Archive	https://www.ebi.ac.uk/ena/data/search?query=PRJEB12501	ERS1039778	SAMEA3732629	ERX1298115	ERR1225903	ITS	ITS2	ATCAGACACG	TCCTCCGCTTATTGATATGC	GCATCGATGAAGAACGCAGC	2011-07-07	46.42	-83.37	Canada	0.1	228	NA	248	Orthic Humo-Ferric Podzol	Jack Pine, Black Spruce, Red Pine	Dfb, Humid Continental cool summer	OM1	C0	0	O horizon	47.0	34.6	1.1	4.1	7.7	30.1
JW024F	JW024	JP_ON_	Ontario	JW	Forest Soil	Whole Community DNA	PCR	Amplicon	pyrotag library	454 GS FLX Titanium	European Nucleotide Archive	https://www.ebi.ac.uk/ena/data/search?query=PRJEB12501	ERS1039779	SAMEA3732630	ERX1298116	ERR1225904	ITS	ITS2	ATATCGCGAG	TCCTCCGCTTATTGATATGC	GCATCGATGAAGAACGCAGC	2011-07-07	46.42	-83.37	Canada	0.3	228	NA	248	Orthic Humo-Ferric Podzol	Jack Pine, Black Spruce, Red Pine	Dfb, Humid Continental cool summer	OM1	C0	0	A horizon	12.0	3.1	0.2	5.3	7.7	14.8
JW025F	JW025	JP_ON_	Ontario	JW	Forest Soil	Whole Community DNA	PCR	Amplicon	pyrotag library	454 GS FLX Titanium	European Nucleotide Archive	https://www.ebi.ac.uk/ena/data/search?query=PRJEB12501	ERS1039780	SAMEA3732631	ERX1298117	ERR1225905	ITS	ITS2	CAGTAGACGT	TCCTCCGCTTATTGATATGC	GCATCGATGAAGAACGCAGC	2011-07-07	46.42	-83.37	Canada	0.1	228	NA	248	Orthic Humo-Ferric Podzol	Jack Pine, Black Spruce, Red Pine	Dfb, Humid Continental cool summer	REF	REF	0	O horizon	40.0	34.6	1.1	4.1	7.7	30.1
JW027F	JW027	JP_ON_	Ontario	JW	Forest Soil	Whole Community DNA	PCR	Amplicon	pyrotag library	454 GS FLX Titanium	European Nucleotide Archive	https://www.ebi.ac.uk/ena/data/search?query=PRJEB12501	ERS1039781	SAMEA3732632	ERX1298118	ERR1225906	ITS	ITS2	ATAGAGTACT	TCCTCCGCTTATTGATATGC	GCATCGATGAAGAACGCAGC	2011-07-07	46.42	-83.37	Canada	0.1	228	NA	248	Orthic Humo-Ferric Podzol	Jack Pine, Black Spruce, Red Pine	Dfb, Humid Continental cool summer	OM1	C0	1	O horizon	34.0	33.8	1.0	4.4	6.3	32.8
JW029F	JW029	JP_ON_	Ontario	JW	Forest Soil	Whole Community DNA	PCR	Amplicon	pyrotag library	454 GS FLX Titanium	European Nucleotide Archive	https://www.ebi.ac.uk/ena/data/search?query=PRJEB12501	ERS1039782	SAMEA3732633	ERX1298119	ERR1225907	ITS	ITS2	CAGTAGACGT	TCCTCCGCTTATTGATATGC	GCATCGATGAAGAACGCAGC	2011-07-07	46.42	-83.37	Canada	0.1	228	NA	248	Orthic Humo-Ferric Podzol	Jack Pine, Black Spruce, Red Pine	Dfb, Humid Continental cool summer	OM1	C0	0	O horizon	57.0	33.8	1.0	4.4	6.3	32.8
JW030F	JW030	JP_ON_	Ontario	JW	Forest Soil	Whole Community DNA	PCR	Amplicon	pyrotag library	454 GS FLX Titanium	European Nucleotide Archive	https://www.ebi.ac.uk/ena/data/search?query=PRJEB12501	ERS1039783	SAMEA3732634	ERX1298120	ERR1225908	ITS	ITS2	ACGCGAGTAT	TCCTCCGCTTATTGATATGC	GCATCGATGAAGAACGCAGC	2011-07-07	46.42	-83.37	Canada	0.3	228	NA	248	Orthic Humo-Ferric Podzol	Jack Pine, Black Spruce, Red Pine	Dfb, Humid Continental cool summer	OM1	C0	0	A horizon	14.0	3.3	0.3	5.3	6.3	13.6
JW031F	JW031	JP_ON_	Ontario	JW	Forest Soil	Whole Community DNA	PCR	Amplicon	pyrotag library	454 GS FLX Titanium	European Nucleotide Archive	https://www.ebi.ac.uk/ena/data/search?query=PRJEB12501	ERS1039784	SAMEA3732635	ERX1298121	ERR1225909	ITS	ITS2	TAGTGTAGAT	TCCTCCGCTTATTGATATGC	GCATCGATGAAGAACGCAGC	2011-07-07	46.42	-83.37	Canada	0.1	228	NA	248	Orthic Humo-Ferric Podzol	Jack Pine, Black Spruce, Red Pine	Dfb, Humid Continental cool summer	OM2	C0	1	O horizon	40.0	37.6	1.1	4.0	7.7	33.6
JW032F	JW032	JP_ON_	Ontario	JW	Forest Soil	Whole Community DNA	PCR	Amplicon	pyrotag library	454 GS FLX Titanium	European Nucleotide Archive	https://www.ebi.ac.uk/ena/data/search?query=PRJEB12501	ERS1039785	SAMEA3732636	ERX1298122	ERR1225910	ITS	ITS2	TGATACGTCT	TCCTCCGCTTATTGATATGC	GCATCGATGAAGAACGCAGC	2011-07-07	46.42	-83.37	Canada	0.3	228	NA	248	Orthic Humo-Ferric Podzol	Jack Pine, Black Spruce, Red Pine	Dfb, Humid Continental cool summer	OM2	C0	1	A horizon	17.0	3.0	0.4	5.1	7.7	13.9
JW033F	JW033	JP_ON_	Ontario	JW	Forest Soil	Whole Community DNA	PCR	Amplicon	pyrotag library	454 GS FLX Titanium	European Nucleotide Archive	https://www.ebi.ac.uk/ena/data/search?query=PRJEB12501	ERS1039786	SAMEA3732637	ERX1298123	ERR1225911	ITS	ITS2	TGATACGTCT	TCCTCCGCTTATTGATATGC	GCATCGATGAAGAACGCAGC	2011-07-07	46.42	-83.37	Canada	0.1	228	NA	248	Orthic Humo-Ferric Podzol	Jack Pine, Black Spruce, Red Pine	Dfb, Humid Continental cool summer	OM2	C0	0	O horizon	60.0	37.6	1.1	4.0	7.7	33.6
JW034F	JW034	JP_ON_	Ontario	JW	Forest Soil	Whole Community DNA	PCR	Amplicon	pyrotag library	454 GS FLX Titanium	European Nucleotide Archive	https://www.ebi.ac.uk/ena/data/search?query=PRJEB12501	ERS1039787	SAMEA3732638	ERX1298124	ERR1225912	ITS	ITS2	TCACGTACTA	TCCTCCGCTTATTGATATGC	GCATCGATGAAGAACGCAGC	2011-07-07	46.42	-83.37	Canada	0.3	228	NA	248	Orthic Humo-Ferric Podzol	Jack Pine, Black Spruce, Red Pine	Dfb, Humid Continental cool summer	OM2	C0	0	A horizon	16.0	3.0	0.4	5.1	7.7	13.9
JW035F	JW035	JP_ON_	Ontario	JW	Forest Soil	Whole Community DNA	PCR	Amplicon	pyrotag library	454 GS FLX Titanium	European Nucleotide Archive	https://www.ebi.ac.uk/ena/data/search?query=PRJEB12501	ERS1039788	SAMEA3732639	ERX1298125	ERR1225913	ITS	ITS2	AGACGCACTC	TCCTCCGCTTATTGATATGC	GCATCGATGAAGAACGCAGC	2011-07-07	46.42	-83.37	Canada	0.1	228	NA	248	Orthic Humo-Ferric Podzol	Jack Pine, Black Spruce, Red Pine	Dfb, Humid Continental cool summer	OM2	C0	1	O horizon	46.0	35.2	1.1	4.3	9.7	32.5
JW036F	JW036	JP_ON_	Ontario	JW	Forest Soil	Whole Community DNA	PCR	Amplicon	pyrotag library	454 GS FLX Titanium	European Nucleotide Archive	https://www.ebi.ac.uk/ena/data/search?query=PRJEB12501	ERS1039789	SAMEA3732640	ERX1298126	ERR1225914	ITS	ITS2	ATACGACGTA	TCCTCCGCTTATTGATATGC	GCATCGATGAAGAACGCAGC	2011-07-07	46.42	-83.37	Canada	0.3	228	NA	248	Orthic Humo-Ferric Podzol	Jack Pine, Black Spruce, Red Pine	Dfb, Humid Continental cool summer	OM2	C0	1	A horizon	13.0	2.7	0.5	5.4	9.7	11.1
JW038F	JW038	JP_ON_	Ontario	JW	Forest Soil	Whole Community DNA	PCR	Amplicon	pyrotag library	454 GS FLX Titanium	European Nucleotide Archive	https://www.ebi.ac.uk/ena/data/search?query=PRJEB12501	ERS1039790	SAMEA3732641	ERX1298127	ERR1225915	ITS	ITS2	AGCGTCGTCT	TCCTCCGCTTATTGATATGC	GCATCGATGAAGAACGCAGC	2011-07-07	46.42	-83.37	Canada	0.3	228	NA	248	Orthic Humo-Ferric Podzol	Jack Pine, Black Spruce, Red Pine	Dfb, Humid Continental cool summer	OM2	C0	0	A horizon	13.0	2.7	0.5	5.4	9.7	11.1
JW040F	JW040	JP_ON_	Ontario	JW	Forest Soil	Whole Community DNA	PCR	Amplicon	pyrotag library	454 GS FLX Titanium	European Nucleotide Archive	https://www.ebi.ac.uk/ena/data/search?query=PRJEB12501	ERS1039791	SAMEA3732642	ERX1298128	ERR1225916	ITS	ITS2	TACAGATCGT	TCCTCCGCTTATTGATATGC	GCATCGATGAAGAACGCAGC	2011-07-07	46.42	-83.37	Canada	0.3	228	NA	248	Orthic Humo-Ferric Podzol	Jack Pine, Black Spruce, Red Pine	Dfb, Humid Continental cool summer	OM3	C0	1	A horizon	19.0	1.3	0.6	5.4	9.0	9.8
JW042F	JW042	JP_ON_	Ontario	JW	Forest Soil	Whole Community DNA	PCR	Amplicon	pyrotag library	454 GS FLX Titanium	European Nucleotide Archive	https://www.ebi.ac.uk/ena/data/search?query=PRJEB12501	ERS1039792	SAMEA3732643	ERX1298129	ERR1225917	ITS	ITS2	TACACACACT	TCCTCCGCTTATTGATATGC	GCATCGATGAAGAACGCAGC	2011-07-07	46.42	-83.37	Canada	0.3	228	NA	248	Orthic Humo-Ferric Podzol	Jack Pine, Black Spruce, Red Pine	Dfb, Humid Continental cool summer	OM3	C0	0	A horizon	14.0	1.3	0.6	5.4	9.0	9.8
LH001F	LH001	PP_CA_	California	LH	Forest Soil	Whole Community DNA	PCR	Amplicon	pyrotag library	454 GS FLX Titanium	European Nucleotide Archive	https://www.ebi.ac.uk/ena/data/search?query=PRJEB12501	ERS1039793	SAMEA3732644	ERX1298130	ERR1225918	ITS	ITS2	ATACGACGTA	TCCTCCGCTTATTGATATGC	GCATCGATGAAGAACGCAGC	2011-09-16	39.26	-120.78	USA	0.1	1268	11.2	55	Mesic Ultic Haploxeralfs	Ponderosa pine, sugar pine, white fir, giant sequoia	Csa, Mediterranean hot summer	OM1	C0	0	O horizon	14.0	NA	NA	3.7	NA	NA
LH002F	LH002	PP_CA_	California	LH	Forest Soil	Whole Community DNA	PCR	Amplicon	pyrotag library	454 GS FLX Titanium	European Nucleotide Archive	https://www.ebi.ac.uk/ena/data/search?query=PRJEB12501	ERS1039794	SAMEA3732645	ERX1298131	ERR1225919	ITS	ITS2	AGACTATACT	TCCTCCGCTTATTGATATGC	GCATCGATGAAGAACGCAGC	2011-09-16	39.26	-120.78	USA	0.3	1268	11.2	55	Mesic Ultic Haploxeralfs	Ponderosa pine, sugar pine, white fir, giant sequoia	Csa, Mediterranean hot summer	OM1	C0	0	A horizon	19.0	3.1	0.1	5.2	NA	24.8
LH003F	LH003	PP_CA_	California	LH	Forest Soil	Whole Community DNA	PCR	Amplicon	pyrotag library	454 GS FLX Titanium	European Nucleotide Archive	https://www.ebi.ac.uk/ena/data/search?query=PRJEB12501	ERS1039795	SAMEA3732646	ERX1298132	ERR1225920	ITS	ITS2	TAGTGTAGAT	TCCTCCGCTTATTGATATGC	GCATCGATGAAGAACGCAGC	2011-09-16	39.26	-120.78	USA	0.1	1268	11.2	55	Mesic Ultic Haploxeralfs	Ponderosa pine, sugar pine, white fir, giant sequoia	Csa, Mediterranean hot summer	OM1	C0	0	O horizon	17.0	NA	NA	3.6	NA	NA
LH004F	LH004	PP_CA_	California	LH	Forest Soil	Whole Community DNA	PCR	Amplicon	pyrotag library	454 GS FLX Titanium	European Nucleotide Archive	https://www.ebi.ac.uk/ena/data/search?query=PRJEB12501	ERS1039796	SAMEA3732647	ERX1298133	ERR1225921	ITS	ITS2	CGACGTGACT	TCCTCCGCTTATTGATATGC	GCATCGATGAAGAACGCAGC	2011-09-16	39.26	-120.78	USA	0.3	1268	11.2	55	Mesic Ultic Haploxeralfs	Ponderosa pine, sugar pine, white fir, giant sequoia	Csa, Mediterranean hot summer	OM1	C0	0	A horizon	34.0	3.1	0.1	5.3	NA	24.8
LH005F	LH005	PP_CA_	California	LH	Forest Soil	Whole Community DNA	PCR	Amplicon	pyrotag library	454 GS FLX Titanium	European Nucleotide Archive	https://www.ebi.ac.uk/ena/data/search?query=PRJEB12501	ERS1039797	SAMEA3732648	ERX1298134	ERR1225922	ITS	ITS2	AGACTATACT	TCCTCCGCTTATTGATATGC	GCATCGATGAAGAACGCAGC	2011-09-16	39.26	-120.78	USA	0.1	1268	11.2	55	Mesic Ultic Haploxeralfs	Ponderosa pine, sugar pine, white fir, giant sequoia	Csa, Mediterranean hot summer	OM1	C0	0	O horizon	15.0	NA	NA	3.9	NA	NA
LH006F	LH006	PP_CA_	California	LH	Forest Soil	Whole Community DNA	PCR	Amplicon	pyrotag library	454 GS FLX Titanium	European Nucleotide Archive	https://www.ebi.ac.uk/ena/data/search?query=PRJEB12501	ERS1039798	SAMEA3732649	ERX1298135	ERR1225923	ITS	ITS2	CATAGTAGTG	TCCTCCGCTTATTGATATGC	GCATCGATGAAGAACGCAGC	2011-09-16	39.26	-120.78	USA	0.3	1268	11.2	55	Mesic Ultic Haploxeralfs	Ponderosa pine, sugar pine, white fir, giant sequoia	Csa, Mediterranean hot summer	OM1	C0	0	A horizon	19.0	3.1	0.1	5.5	NA	24.8
LH007F	LH007	PP_CA_	California	LH	Forest Soil	Whole Community DNA	PCR	Amplicon	pyrotag library	454 GS FLX Titanium	European Nucleotide Archive	https://www.ebi.ac.uk/ena/data/search?query=PRJEB12501	ERS1039799	SAMEA3732650	ERX1298136	ERR1225924	ITS	ITS2	AGACTATACT	TCCTCCGCTTATTGATATGC	GCATCGATGAAGAACGCAGC	2011-09-16	39.26	-120.78	USA	0.1	1268	11.2	55	Mesic Ultic Haploxeralfs	Ponderosa pine, sugar pine, white fir, giant sequoia	Csa, Mediterranean hot summer	OM2	C0	0	O horizon	10.0	NA	NA	4.6	NA	NA
LH009F	LH009	PP_CA_	California	LH	Forest Soil	Whole Community DNA	PCR	Amplicon	pyrotag library	454 GS FLX Titanium	European Nucleotide Archive	https://www.ebi.ac.uk/ena/data/search?query=PRJEB12501	ERS1039800	SAMEA3732651	ERX1298137	ERR1225925	ITS	ITS2	CACGCTACGT	TCCTCCGCTTATTGATATGC	GCATCGATGAAGAACGCAGC	2011-09-16	39.26	-120.78	USA	0.1	1268	11.2	55	Mesic Ultic Haploxeralfs	Ponderosa pine, sugar pine, white fir, giant sequoia	Csa, Mediterranean hot summer	OM2	C0	0	O horizon	34.0	NA	NA	4.5	NA	NA
LH010F	LH010	PP_CA_	California	LH	Forest Soil	Whole Community DNA	PCR	Amplicon	pyrotag library	454 GS FLX Titanium	European Nucleotide Archive	https://www.ebi.ac.uk/ena/data/search?query=PRJEB12501	ERS1039801	SAMEA3732652	ERX1298138	ERR1225926	ITS	ITS2	ATACGACGTA	TCCTCCGCTTATTGATATGC	GCATCGATGAAGAACGCAGC	2011-09-16	39.26	-120.78	USA	0.3	1268	11.2	55	Mesic Ultic Haploxeralfs	Ponderosa pine, sugar pine, white fir, giant sequoia	Csa, Mediterranean hot summer	OM2	C0	0	A horizon	18.0	3.6	0.2	5.9	NA	22.3
LH011F	LH011	PP_CA_	California	LH	Forest Soil	Whole Community DNA	PCR	Amplicon	pyrotag library	454 GS FLX Titanium	European Nucleotide Archive	https://www.ebi.ac.uk/ena/data/search?query=PRJEB12501	ERS1039802	SAMEA3732653	ERX1298139	ERR1225927	ITS	ITS2	AGCACTGTAG	TCCTCCGCTTATTGATATGC	GCATCGATGAAGAACGCAGC	2011-09-16	39.26	-120.78	USA	0.1	1268	11.2	55	Mesic Ultic Haploxeralfs	Ponderosa pine, sugar pine, white fir, giant sequoia	Csa, Mediterranean hot summer	OM2	C0	0	O horizon	7.0	NA	NA	4.2	NA	NA
LH012F	LH012	PP_CA_	California	LH	Forest Soil	Whole Community DNA	PCR	Amplicon	pyrotag library	454 GS FLX Titanium	European Nucleotide Archive	https://www.ebi.ac.uk/ena/data/search?query=PRJEB12501	ERS1039803	SAMEA3732654	ERX1298140	ERR1225928	ITS	ITS2	CGACGTGACT	TCCTCCGCTTATTGATATGC	GCATCGATGAAGAACGCAGC	2011-09-16	39.26	-120.78	USA	0.3	1268	11.2	55	Mesic Ultic Haploxeralfs	Ponderosa pine, sugar pine, white fir, giant sequoia	Csa, Mediterranean hot summer	OM2	C0	0	A horizon	18.0	3.6	0.2	5.7	NA	22.3
LH014F	LH014	PP_CA_	California	LH	Forest Soil	Whole Community DNA	PCR	Amplicon	pyrotag library	454 GS FLX Titanium	European Nucleotide Archive	https://www.ebi.ac.uk/ena/data/search?query=PRJEB12501	ERS1039804	SAMEA3732655	ERX1298141	ERR1225929	ITS	ITS2	ACTGTACAGT	TCCTCCGCTTATTGATATGC	GCATCGATGAAGAACGCAGC	2011-09-16	39.26	-120.78	USA	0.3	1268	11.2	55	Mesic Ultic Haploxeralfs	Ponderosa pine, sugar pine, white fir, giant sequoia	Csa, Mediterranean hot summer	OM3	C0	0	A horizon	17.0	3.3	0.2	5.0	NA	21.6
LH015F	LH015	PP_CA_	California	LH	Forest Soil	Whole Community DNA	PCR	Amplicon	pyrotag library	454 GS FLX Titanium	European Nucleotide Archive	https://www.ebi.ac.uk/ena/data/search?query=PRJEB12501	ERS1039805	SAMEA3732656	ERX1298142	ERR1225930	ITS	ITS2	ACGAGTGCGT	TCCTCCGCTTATTGATATGC	GCATCGATGAAGAACGCAGC	2011-09-16	39.26	-120.78	USA	0.1	1268	11.2	55	Mesic Ultic Haploxeralfs	Ponderosa pine, sugar pine, white fir, giant sequoia	Csa, Mediterranean hot summer	OM3	C0	0	O horizon	14.0	NA	NA	5.8	NA	NA
LH016F	LH016	PP_CA_	California	LH	Forest Soil	Whole Community DNA	PCR	Amplicon	pyrotag library	454 GS FLX Titanium	European Nucleotide Archive	https://www.ebi.ac.uk/ena/data/search?query=PRJEB12501	ERS1039806	SAMEA3732657	ERX1298143	ERR1225931	ITS	ITS2	TACAGATCGT	TCCTCCGCTTATTGATATGC	GCATCGATGAAGAACGCAGC	2011-09-16	39.26	-120.78	USA	0.3	1268	11.2	55	Mesic Ultic Haploxeralfs	Ponderosa pine, sugar pine, white fir, giant sequoia	Csa, Mediterranean hot summer	OM3	C0	0	A horizon	17.0	3.3	0.2	6.3	NA	21.6
LH018F	LH018	PP_CA_	California	LH	Forest Soil	Whole Community DNA	PCR	Amplicon	pyrotag library	454 GS FLX Titanium	European Nucleotide Archive	https://www.ebi.ac.uk/ena/data/search?query=PRJEB12501	ERS1039807	SAMEA3732658	ERX1298144	ERR1225932	ITS	ITS2	TGATACGTCT	TCCTCCGCTTATTGATATGC	GCATCGATGAAGAACGCAGC	2011-09-16	39.26	-120.78	USA	0.3	1268	11.2	55	Mesic Ultic Haploxeralfs	Ponderosa pine, sugar pine, white fir, giant sequoia	Csa, Mediterranean hot summer	OM3	C0	0	A horizon	16.0	3.3	0.2	6.0	NA	21.6
LH019F	LH019	PP_CA_	California	LH	Forest Soil	Whole Community DNA	PCR	Amplicon	pyrotag library	454 GS FLX Titanium	European Nucleotide Archive	https://www.ebi.ac.uk/ena/data/search?query=PRJEB12501	ERS1039808	SAMEA3732659	ERX1298145	ERR1225933	ITS	ITS2	TCTACGTAGC	TCCTCCGCTTATTGATATGC	GCATCGATGAAGAACGCAGC	2011-09-16	39.26	-120.78	USA	0.1	1268	11.2	55	Mesic Ultic Haploxeralfs	Ponderosa pine, sugar pine, white fir, giant sequoia	Csa, Mediterranean hot summer	REF	REF	0	O horizon	22.0	NA	NA	5.6	NA	NA
LH021F	LH021	PP_CA_	California	LH	Forest Soil	Whole Community DNA	PCR	Amplicon	pyrotag library	454 GS FLX Titanium	European Nucleotide Archive	https://www.ebi.ac.uk/ena/data/search?query=PRJEB12501	ERS1039809	SAMEA3732660	ERX1298146	ERR1225934	ITS	ITS2	ACGCTCGACA	TCCTCCGCTTATTGATATGC	GCATCGATGAAGAACGCAGC	2011-09-16	39.26	-120.78	USA	0.1	1268	11.2	55	Mesic Ultic Haploxeralfs	Ponderosa pine, sugar pine, white fir, giant sequoia	Csa, Mediterranean hot summer	REF	REF	0	O horizon	14.0	NA	NA	4.9	NA	NA
LH022F	LH022	PP_CA_	California	LH	Forest Soil	Whole Community DNA	PCR	Amplicon	pyrotag library	454 GS FLX Titanium	European Nucleotide Archive	https://www.ebi.ac.uk/ena/data/search?query=PRJEB12501	ERS1039810	SAMEA3732661	ERX1298147	ERR1225935	ITS	ITS2	AGACGCACTC	TCCTCCGCTTATTGATATGC	GCATCGATGAAGAACGCAGC	2011-09-16	39.26	-120.78	USA	0.3	1268	11.2	55	Mesic Ultic Haploxeralfs	Ponderosa pine, sugar pine, white fir, giant sequoia	Csa, Mediterranean hot summer	REF	REF	0	A horizon	19.0	3.9	0.2	6.1	NA	26.0
LH023F	LH023	PP_CA_	California	LH	Forest Soil	Whole Community DNA	PCR	Amplicon	pyrotag library	454 GS FLX Titanium	European Nucleotide Archive	https://www.ebi.ac.uk/ena/data/search?query=PRJEB12501	ERS1039811	SAMEA3732662	ERX1298148	ERR1225936	ITS	ITS2	ACGAGTGCGT	TCCTCCGCTTATTGATATGC	GCATCGATGAAGAACGCAGC	2011-09-16	39.26	-120.78	USA	0.1	1268	11.2	55	Mesic Ultic Haploxeralfs	Ponderosa pine, sugar pine, white fir, giant sequoia	Csa, Mediterranean hot summer	REF	REF	0	O horizon	13.0	NA	NA	5.7	NA	NA
LH024F	LH024	PP_CA_	California	LH	Forest Soil	Whole Community DNA	PCR	Amplicon	pyrotag library	454 GS FLX Titanium	European Nucleotide Archive	https://www.ebi.ac.uk/ena/data/search?query=PRJEB12501	ERS1039812	SAMEA3732663	ERX1298149	ERR1225937	ITS	ITS2	TAGTGTAGAT	TCCTCCGCTTATTGATATGC	GCATCGATGAAGAACGCAGC	2011-09-16	39.26	-120.78	USA	0.3	1268	11.2	55	Mesic Ultic Haploxeralfs	Ponderosa pine, sugar pine, white fir, giant sequoia	Csa, Mediterranean hot summer	REF	REF	0	A horizon	19.0	3.9	0.2	6.5	NA	26.0
OC362F	OC362	IDF_BC_	British Columbia	OC	Forest Soil	Whole Community DNA	PCR	Amplicon	pyrotag library	454 GS FLX Titanium	European Nucleotide Archive	https://www.ebi.ac.uk/ena/data/search?query=PRJEB12501	ERS1039814	SAMEA3732665	ERX1298151	ERR1225939	ITS	ITS2	CGTCTAGTAC	TCCTCCGCTTATTGATATGC	GCATCGATGAAGAACGCAGC	2010-06-26	50.88	-120.35	Canada	0.1	1075	2.5	300	Brunisolic Gray Luvisol	Douglas fir	Dfb, Humid Continental warm summer	REF	REF	0	O horizon	46.0	NA	NA	0.0	NA	NA
OC363F	OC363	IDF_BC_	British Columbia	OC	Forest Soil	Whole Community DNA	PCR	Amplicon	pyrotag library	454 GS FLX Titanium	European Nucleotide Archive	https://www.ebi.ac.uk/ena/data/search?query=PRJEB12501	ERS1039815	SAMEA3732666	ERX1298152	ERR1225940	ITS	ITS2	AGTACGCTAT	TCCTCCGCTTATTGATATGC	GCATCGATGAAGAACGCAGC	2010-06-26	50.88	-120.35	Canada	0.1	1075	2.5	300	Brunisolic Gray Luvisol	Douglas fir	Dfb, Humid Continental warm summer	REF	REF	0	O horizon	54.0	NA	NA	0.0	NA	NA
OC364F	OC364	IDF_BC_	British Columbia	OC	Forest Soil	Whole Community DNA	PCR	Amplicon	pyrotag library	454 GS FLX Titanium	European Nucleotide Archive	https://www.ebi.ac.uk/ena/data/search?query=PRJEB12501	ERS1039816	SAMEA3732667	ERX1298153	ERR1225941	ITS	ITS2	ACTACTATGT	TCCTCCGCTTATTGATATGC	GCATCGATGAAGAACGCAGC	2010-06-26	50.88	-120.35	Canada	0.3	1075	2.5	300	Brunisolic Gray Luvisol	Douglas fir	Dfb, Humid Continental warm summer	REF	REF	0	A horizon	12.0	NA	NA	0.0	NA	NA
OC365F	OC365	IDF_BC_	British Columbia	OC	Forest Soil	Whole Community DNA	PCR	Amplicon	pyrotag library	454 GS FLX Titanium	European Nucleotide Archive	https://www.ebi.ac.uk/ena/data/search?query=PRJEB12501	ERS1039817	SAMEA3732668	ERX1298154	ERR1225942	ITS	ITS2	ATACGACGTA	TCCTCCGCTTATTGATATGC	GCATCGATGAAGAACGCAGC	2010-06-26	50.88	-120.35	Canada	0.3	1075	2.5	300	Brunisolic Gray Luvisol	Douglas fir	Dfb, Humid Continental warm summer	REF	REF	0	A horizon	13.0	NA	NA	0.0	NA	NA
OC366F	OC366	IDF_BC_	British Columbia	OC	Forest Soil	Whole Community DNA	PCR	Amplicon	pyrotag library	454 GS FLX Titanium	European Nucleotide Archive	https://www.ebi.ac.uk/ena/data/search?query=PRJEB12501	ERS1039818	SAMEA3732669	ERX1298155	ERR1225943	ITS	ITS2	CTCGCGTGTC	TCCTCCGCTTATTGATATGC	GCATCGATGAAGAACGCAGC	2010-06-26	50.88	-120.35	Canada	0.3	1075	2.5	300	Brunisolic Gray Luvisol	Douglas fir	Dfb, Humid Continental warm summer	REF	REF	0	A horizon	19.0	NA	NA	0.0	NA	NA
OC367F	OC367	IDF_BC_	British Columbia	OC	Forest Soil	Whole Community DNA	PCR	Amplicon	pyrotag library	454 GS FLX Titanium	European Nucleotide Archive	https://www.ebi.ac.uk/ena/data/search?query=PRJEB12501	ERS1039819	SAMEA3732670	ERX1298156	ERR1225944	ITS	ITS2	ACGCGAGTAT	TCCTCCGCTTATTGATATGC	GCATCGATGAAGAACGCAGC	2010-06-26	50.88	-120.35	Canada	0.1	1075	2.5	300	Brunisolic Gray Luvisol	Douglas fir	Dfb, Humid Continental warm summer	OM1	C0	0	O horizon	57.0	NA	NA	0.0	NA	NA
OC368F	OC368	IDF_BC_	British Columbia	OC	Forest Soil	Whole Community DNA	PCR	Amplicon	pyrotag library	454 GS FLX Titanium	European Nucleotide Archive	https://www.ebi.ac.uk/ena/data/search?query=PRJEB12501	ERS1039820	SAMEA3732671	ERX1298157	ERR1225945	ITS	ITS2	TCTACGTAGC	TCCTCCGCTTATTGATATGC	GCATCGATGAAGAACGCAGC	2010-06-26	50.88	-120.35	Canada	0.1	1075	2.5	300	Brunisolic Gray Luvisol	Douglas fir	Dfb, Humid Continental warm summer	OM1	C0	0	O horizon	57.0	NA	NA	0.0	NA	NA
OC369F	OC369	IDF_BC_	British Columbia	OC	Forest Soil	Whole Community DNA	PCR	Amplicon	pyrotag library	454 GS FLX Titanium	European Nucleotide Archive	https://www.ebi.ac.uk/ena/data/search?query=PRJEB12501	ERS1039821	SAMEA3732672	ERX1298158	ERR1225946	ITS	ITS2	CGACGTGACT	TCCTCCGCTTATTGATATGC	GCATCGATGAAGAACGCAGC	2010-06-26	50.88	-120.35	Canada	0.1	1075	2.5	300	Brunisolic Gray Luvisol	Douglas fir	Dfb, Humid Continental warm summer	OM1	C0	0	O horizon	61.0	NA	NA	0.0	NA	NA
OC371F	OC371	IDF_BC_	British Columbia	OC	Forest Soil	Whole Community DNA	PCR	Amplicon	pyrotag library	454 GS FLX Titanium	European Nucleotide Archive	https://www.ebi.ac.uk/ena/data/search?query=PRJEB12501	ERS1039822	SAMEA3732673	ERX1298159	ERR1225947	ITS	ITS2	ACTGTACAGT	TCCTCCGCTTATTGATATGC	GCATCGATGAAGAACGCAGC	2010-06-26	50.88	-120.35	Canada	0.3	1075	2.5	300	Brunisolic Gray Luvisol	Douglas fir	Dfb, Humid Continental warm summer	OM1	C0	0	A horizon	20.0	NA	NA	0.0	NA	NA
OC372F	OC372	IDF_BC_	British Columbia	OC	Forest Soil	Whole Community DNA	PCR	Amplicon	pyrotag library	454 GS FLX Titanium	European Nucleotide Archive	https://www.ebi.ac.uk/ena/data/search?query=PRJEB12501	ERS1039823	SAMEA3732674	ERX1298160	ERR1225948	ITS	ITS2	ACTGTACAGT	TCCTCCGCTTATTGATATGC	GCATCGATGAAGAACGCAGC	2010-06-26	50.88	-120.35	Canada	0.3	1075	2.5	300	Brunisolic Gray Luvisol	Douglas fir	Dfb, Humid Continental warm summer	OM1	C0	0	A horizon	22.0	NA	NA	0.0	NA	NA
OC374F	OC374	IDF_BC_	British Columbia	OC	Forest Soil	Whole Community DNA	PCR	Amplicon	pyrotag library	454 GS FLX Titanium	European Nucleotide Archive	https://www.ebi.ac.uk/ena/data/search?query=PRJEB12501	ERS1039824	SAMEA3732675	ERX1298161	ERR1225949	ITS	ITS2	ACGAGTGCGT	TCCTCCGCTTATTGATATGC	GCATCGATGAAGAACGCAGC	2010-06-26	50.88	-120.35	Canada	0.1	1075	2.5	300	Brunisolic Gray Luvisol	Douglas fir	Dfb, Humid Continental warm summer	OM2	C0	0	O horizon	59.0	NA	NA	0.0	NA	NA
OC375F	OC375	IDF_BC_	British Columbia	OC	Forest Soil	Whole Community DNA	PCR	Amplicon	pyrotag library	454 GS FLX Titanium	European Nucleotide Archive	https://www.ebi.ac.uk/ena/data/search?query=PRJEB12501	ERS1039825	SAMEA3732676	ERX1298162	ERR1225950	ITS	ITS2	TCGATCACGT	TCCTCCGCTTATTGATATGC	GCATCGATGAAGAACGCAGC	2010-06-26	50.88	-120.35	Canada	0.1	1075	2.5	300	Brunisolic Gray Luvisol	Douglas fir	Dfb, Humid Continental warm summer	OM2	C0	0	O horizon	60.0	NA	NA	0.0	NA	NA
OC376F	OC376	IDF_BC_	British Columbia	OC	Forest Soil	Whole Community DNA	PCR	Amplicon	pyrotag library	454 GS FLX Titanium	European Nucleotide Archive	https://www.ebi.ac.uk/ena/data/search?query=PRJEB12501	ERS1039826	SAMEA3732677	ERX1298163	ERR1225951	ITS	ITS2	TCACGTACTA	TCCTCCGCTTATTGATATGC	GCATCGATGAAGAACGCAGC	2010-06-26	50.88	-120.35	Canada	0.3	1075	2.5	300	Brunisolic Gray Luvisol	Douglas fir	Dfb, Humid Continental warm summer	OM2	C0	0	A horizon	20.0	NA	NA	0.0	NA	NA
OC377F	OC377	IDF_BC_	British Columbia	OC	Forest Soil	Whole Community DNA	PCR	Amplicon	pyrotag library	454 GS FLX Titanium	European Nucleotide Archive	https://www.ebi.ac.uk/ena/data/search?query=PRJEB12501	ERS1039827	SAMEA3732678	ERX1298164	ERR1225952	ITS	ITS2	AGCACTGTAG	TCCTCCGCTTATTGATATGC	GCATCGATGAAGAACGCAGC	2010-06-26	50.88	-120.35	Canada	0.3	1075	2.5	300	Brunisolic Gray Luvisol	Douglas fir	Dfb, Humid Continental warm summer	OM2	C0	0	A horizon	21.0	NA	NA	0.0	NA	NA
OC378F	OC378	IDF_BC_	British Columbia	OC	Forest Soil	Whole Community DNA	PCR	Amplicon	pyrotag library	454 GS FLX Titanium	European Nucleotide Archive	https://www.ebi.ac.uk/ena/data/search?query=PRJEB12501	ERS1039828	SAMEA3732679	ERX1298165	ERR1225953	ITS	ITS2	AGCACTGTAG	TCCTCCGCTTATTGATATGC	GCATCGATGAAGAACGCAGC	2010-06-26	50.88	-120.35	Canada	0.3	1075	2.5	300	Brunisolic Gray Luvisol	Douglas fir	Dfb, Humid Continental warm summer	OM2	C0	0	A horizon	21.0	NA	NA	0.0	NA	NA
OC379F	OC379	IDF_BC_	British Columbia	OC	Forest Soil	Whole Community DNA	PCR	Amplicon	pyrotag library	454 GS FLX Titanium	European Nucleotide Archive	https://www.ebi.ac.uk/ena/data/search?query=PRJEB12501	ERS1039829	SAMEA3732680	ERX1298166	ERR1225954	ITS	ITS2	ATCAGACACG	TCCTCCGCTTATTGATATGC	GCATCGATGAAGAACGCAGC	2010-06-26	50.88	-120.35	Canada	0.3	1075	2.5	300	Brunisolic Gray Luvisol	Douglas fir	Dfb, Humid Continental warm summer	OM3	C0	0	A horizon	23.0	NA	NA	0.0	NA	NA
OC381F	OC381	IDF_BC_	British Columbia	OC	Forest Soil	Whole Community DNA	PCR	Amplicon	pyrotag library	454 GS FLX Titanium	European Nucleotide Archive	https://www.ebi.ac.uk/ena/data/search?query=PRJEB12501	ERS1039830	SAMEA3732681	ERX1298167	ERR1225955	ITS	ITS2	CACGCTACGT	TCCTCCGCTTATTGATATGC	GCATCGATGAAGAACGCAGC	2010-06-26	50.88	-120.35	Canada	0.3	1075	2.5	300	Brunisolic Gray Luvisol	Douglas fir	Dfb, Humid Continental warm summer	OM3	C0	0	A horizon	16.0	NA	NA	0.0	NA	NA
TXA001F	TXA001	LP_TX_	Texas	TXA	Forest Soil	Whole Community DNA	PCR	Amplicon	pyrotag library	454 GS FLX Titanium	European Nucleotide Archive	https://www.ebi.ac.uk/ena/data/search?query=PRJEB12501	ERS1039831	SAMEA3732682	ERX1298168	ERR1225956	ITS	ITS2	CGTGTCTCTA	TCCTCCGCTTATTGATATGC	GCATCGATGAAGAACGCAGC	2012-03-12	31.11	-95.15	USA	0.3	88	19.0	253	Aquic Glossudalfs	Loblolly Pine, Beautyberry, Yaupon, Sweetgum, Oaks, Wax Myrtle	Cfa, Humid subtropical	OM1	C0	0	A horizon	0.0	1.1	0.1	5.0	1.3	18.5
TXA003F	TXA003	LP_TX_	Texas	TXA	Forest Soil	Whole Community DNA	PCR	Amplicon	pyrotag library	454 GS FLX Titanium	European Nucleotide Archive	https://www.ebi.ac.uk/ena/data/search?query=PRJEB12501	ERS1039832	SAMEA3732683	ERX1298169	ERR1225957	ITS	ITS2	ATATCGCGAG	TCCTCCGCTTATTGATATGC	GCATCGATGAAGAACGCAGC	2012-03-12	31.11	-95.15	USA	0.3	88	19.0	253	Aquic Glossudalfs	Loblolly Pine, Beautyberry, Yaupon, Sweetgum, Oaks, Wax Myrtle	Cfa, Humid subtropical	OM1	C0	0	A horizon	0.0	1.1	0.1	4.6	1.3	18.5
TXA004F	TXA004	LP_TX_	Texas	TXA	Forest Soil	Whole Community DNA	PCR	Amplicon	pyrotag library	454 GS FLX Titanium	European Nucleotide Archive	https://www.ebi.ac.uk/ena/data/search?query=PRJEB12501	ERS1039833	SAMEA3732684	ERX1298170	ERR1225958	ITS	ITS2	TCGCACTAGT	TCCTCCGCTTATTGATATGC	GCATCGATGAAGAACGCAGC	2012-03-12	31.11	-95.15	USA	0.1	88	19.0	253	Aquic Glossudalfs	Loblolly Pine, Beautyberry, Yaupon, Sweetgum, Oaks, Wax Myrtle	Cfa, Humid subtropical	OM1	C0	0	O horizon	0.0	NA	NA	4.7	NA	NA
TXA005F	TXA005	LP_TX_	Texas	TXA	Forest Soil	Whole Community DNA	PCR	Amplicon	pyrotag library	454 GS FLX Titanium	European Nucleotide Archive	https://www.ebi.ac.uk/ena/data/search?query=PRJEB12501	ERS1039834	SAMEA3732685	ERX1298171	ERR1225959	ITS	ITS2	TACAGATCGT	TCCTCCGCTTATTGATATGC	GCATCGATGAAGAACGCAGC	2012-03-12	31.11	-95.15	USA	0.1	88	19.0	253	Aquic Glossudalfs	Loblolly Pine, Beautyberry, Yaupon, Sweetgum, Oaks, Wax Myrtle	Cfa, Humid subtropical	OM1	C0	0	O horizon	0.0	NA	NA	4.7	NA	NA
TXA008F	TXA008	LP_TX_	Texas	TXA	Forest Soil	Whole Community DNA	PCR	Amplicon	pyrotag library	454 GS FLX Titanium	European Nucleotide Archive	https://www.ebi.ac.uk/ena/data/search?query=PRJEB12501	ERS1039835	SAMEA3732686	ERX1298172	ERR1225960	ITS	ITS2	AGACTATACT	TCCTCCGCTTATTGATATGC	GCATCGATGAAGAACGCAGC	2012-03-12	31.11	-95.15	USA	0.3	88	19.0	253	Aquic Glossudalfs	Loblolly Pine, Beautyberry, Yaupon, Sweetgum, Oaks, Wax Myrtle	Cfa, Humid subtropical	OM1	C0	1	A horizon	0.0	1.1	0.1	4.8	1.3	18.5
TXA009F	TXA009	LP_TX_	Texas	TXA	Forest Soil	Whole Community DNA	PCR	Amplicon	pyrotag library	454 GS FLX Titanium	European Nucleotide Archive	https://www.ebi.ac.uk/ena/data/search?query=PRJEB12501	ERS1039836	SAMEA3732687	ERX1298173	ERR1225961	ITS	ITS2	CGAGAGATAC	TCCTCCGCTTATTGATATGC	GCATCGATGAAGAACGCAGC	2012-03-12	31.11	-95.15	USA	0.3	88	19.0	253	Aquic Glossudalfs	Loblolly Pine, Beautyberry, Yaupon, Sweetgum, Oaks, Wax Myrtle	Cfa, Humid subtropical	OM1	C0	1	A horizon	0.0	1.1	0.1	5.1	1.3	18.5
TXA010F	TXA010	LP_TX_	Texas	TXA	Forest Soil	Whole Community DNA	PCR	Amplicon	pyrotag library	454 GS FLX Titanium	European Nucleotide Archive	https://www.ebi.ac.uk/ena/data/search?query=PRJEB12501	ERS1039837	SAMEA3732688	ERX1298174	ERR1225962	ITS	ITS2	ATAGAGTACT	TCCTCCGCTTATTGATATGC	GCATCGATGAAGAACGCAGC	2012-03-12	31.11	-95.15	USA	0.1	88	19.0	253	Aquic Glossudalfs	Loblolly Pine, Beautyberry, Yaupon, Sweetgum, Oaks, Wax Myrtle	Cfa, Humid subtropical	OM1	C0	1	O horizon	0.0	NA	NA	4.8	NA	NA
TXA012F	TXA012	LP_TX_	Texas	TXA	Forest Soil	Whole Community DNA	PCR	Amplicon	pyrotag library	454 GS FLX Titanium	European Nucleotide Archive	https://www.ebi.ac.uk/ena/data/search?query=PRJEB12501	ERS1039838	SAMEA3732689	ERX1298175	ERR1225963	ITS	ITS2	TACACGTGAT	TCCTCCGCTTATTGATATGC	GCATCGATGAAGAACGCAGC	2012-03-12	31.11	-95.15	USA	0.1	88	19.0	253	Aquic Glossudalfs	Loblolly Pine, Beautyberry, Yaupon, Sweetgum, Oaks, Wax Myrtle	Cfa, Humid subtropical	OM1	C0	1	O horizon	0.0	NA	NA	4.8	NA	NA
TXA015F	TXA015	LP_TX_	Texas	TXA	Forest Soil	Whole Community DNA	PCR	Amplicon	pyrotag library	454 GS FLX Titanium	European Nucleotide Archive	https://www.ebi.ac.uk/ena/data/search?query=PRJEB12501	ERS1039839	SAMEA3732690	ERX1298176	ERR1225964	ITS	ITS2	TCACGTACTA	TCCTCCGCTTATTGATATGC	GCATCGATGAAGAACGCAGC	2012-03-12	31.11	-95.15	USA	0.3	88	19.0	253	Aquic Glossudalfs	Loblolly Pine, Beautyberry, Yaupon, Sweetgum, Oaks, Wax Myrtle	Cfa, Humid subtropical	OM2	C0	0	A horizon	0.0	1.2	0.1	4.3	1.3	23.9
TXA016F	TXA016	LP_TX_	Texas	TXA	Forest Soil	Whole Community DNA	PCR	Amplicon	pyrotag library	454 GS FLX Titanium	European Nucleotide Archive	https://www.ebi.ac.uk/ena/data/search?query=PRJEB12501	ERS1039840	SAMEA3732691	ERX1298177	ERR1225965	ITS	ITS2	AGACGCACTC	TCCTCCGCTTATTGATATGC	GCATCGATGAAGAACGCAGC	2012-03-12	31.11	-95.15	USA	0.1	88	19.0	253	Aquic Glossudalfs	Loblolly Pine, Beautyberry, Yaupon, Sweetgum, Oaks, Wax Myrtle	Cfa, Humid subtropical	OM2	C0	0	O horizon	0.0	NA	NA	4.5	NA	NA
TXA017F	TXA017	LP_TX_	Texas	TXA	Forest Soil	Whole Community DNA	PCR	Amplicon	pyrotag library	454 GS FLX Titanium	European Nucleotide Archive	https://www.ebi.ac.uk/ena/data/search?query=PRJEB12501	ERS1039841	SAMEA3732692	ERX1298178	ERR1225966	ITS	ITS2	TACGCTGTCT	TCCTCCGCTTATTGATATGC	GCATCGATGAAGAACGCAGC	2012-03-12	31.11	-95.15	USA	0.1	88	19.0	253	Aquic Glossudalfs	Loblolly Pine, Beautyberry, Yaupon, Sweetgum, Oaks, Wax Myrtle	Cfa, Humid subtropical	OM2	C0	0	O horizon	0.0	NA	NA	5.7	NA	NA
TXA019F	TXA019	LP_TX_	Texas	TXA	Forest Soil	Whole Community DNA	PCR	Amplicon	pyrotag library	454 GS FLX Titanium	European Nucleotide Archive	https://www.ebi.ac.uk/ena/data/search?query=PRJEB12501	ERS1039842	SAMEA3732693	ERX1298179	ERR1225967	ITS	ITS2	TACACGTGAT	TCCTCCGCTTATTGATATGC	GCATCGATGAAGAACGCAGC	2012-03-12	31.11	-95.15	USA	0.3	88	19.0	253	Aquic Glossudalfs	Loblolly Pine, Beautyberry, Yaupon, Sweetgum, Oaks, Wax Myrtle	Cfa, Humid subtropical	OM2	C0	1	A horizon	0.0	1.2	0.1	4.6	1.3	23.9
TXA021F	TXA021	LP_TX_	Texas	TXA	Forest Soil	Whole Community DNA	PCR	Amplicon	pyrotag library	454 GS FLX Titanium	European Nucleotide Archive	https://www.ebi.ac.uk/ena/data/search?query=PRJEB12501	ERS1039843	SAMEA3732694	ERX1298180	ERR1225968	ITS	ITS2	ATATCGCGAG	TCCTCCGCTTATTGATATGC	GCATCGATGAAGAACGCAGC	2012-03-12	31.11	-95.15	USA	0.3	88	19.0	253	Aquic Glossudalfs	Loblolly Pine, Beautyberry, Yaupon, Sweetgum, Oaks, Wax Myrtle	Cfa, Humid subtropical	OM2	C0	1	A horizon	0.0	1.2	0.1	5.1	1.3	23.9
TXA022F	TXA022	LP_TX_	Texas	TXA	Forest Soil	Whole Community DNA	PCR	Amplicon	pyrotag library	454 GS FLX Titanium	European Nucleotide Archive	https://www.ebi.ac.uk/ena/data/search?query=PRJEB12501	ERS1039844	SAMEA3732695	ERX1298181	ERR1225969	ITS	ITS2	CGTCTAGTAC	TCCTCCGCTTATTGATATGC	GCATCGATGAAGAACGCAGC	2012-03-12	31.11	-95.15	USA	0.1	88	19.0	253	Aquic Glossudalfs	Loblolly Pine, Beautyberry, Yaupon, Sweetgum, Oaks, Wax Myrtle	Cfa, Humid subtropical	OM2	C0	1	O horizon	0.0	NA	NA	4.5	NA	NA
TXA023F	TXA023	LP_TX_	Texas	TXA	Forest Soil	Whole Community DNA	PCR	Amplicon	pyrotag library	454 GS FLX Titanium	European Nucleotide Archive	https://www.ebi.ac.uk/ena/data/search?query=PRJEB12501	ERS1039845	SAMEA3732696	ERX1298182	ERR1225970	ITS	ITS2	CATAGTAGTG	TCCTCCGCTTATTGATATGC	GCATCGATGAAGAACGCAGC	2012-03-12	31.11	-95.15	USA	0.1	88	19.0	253	Aquic Glossudalfs	Loblolly Pine, Beautyberry, Yaupon, Sweetgum, Oaks, Wax Myrtle	Cfa, Humid subtropical	OM2	C0	1	O horizon	0.0	NA	NA	4.1	NA	NA
TXA024F	TXA024	LP_TX_	Texas	TXA	Forest Soil	Whole Community DNA	PCR	Amplicon	pyrotag library	454 GS FLX Titanium	European Nucleotide Archive	https://www.ebi.ac.uk/ena/data/search?query=PRJEB12501	ERS1039846	SAMEA3732697	ERX1298183	ERR1225971	ITS	ITS2	TAGTGTAGAT	TCCTCCGCTTATTGATATGC	GCATCGATGAAGAACGCAGC	2012-03-12	31.11	-95.15	USA	0.1	88	19.0	253	Aquic Glossudalfs	Loblolly Pine, Beautyberry, Yaupon, Sweetgum, Oaks, Wax Myrtle	Cfa, Humid subtropical	OM2	C0	1	O horizon	1.0	NA	NA	4.6	NA	NA
TXA025F	TXA025	LP_TX_	Texas	TXA	Forest Soil	Whole Community DNA	PCR	Amplicon	pyrotag library	454 GS FLX Titanium	European Nucleotide Archive	https://www.ebi.ac.uk/ena/data/search?query=PRJEB12501	ERS1039847	SAMEA3732698	ERX1298184	ERR1225972	ITS	ITS2	ACGCGAGTAT	TCCTCCGCTTATTGATATGC	GCATCGATGAAGAACGCAGC	2012-03-12	31.11	-95.15	USA	0.3	88	19.0	253	Aquic Glossudalfs	Loblolly Pine, Beautyberry, Yaupon, Sweetgum, Oaks, Wax Myrtle	Cfa, Humid subtropical	OM3	C0	0	A horizon	0.0	0.8	0.0	4.6	1.3	17.8
TXA027F	TXA027	LP_TX_	Texas	TXA	Forest Soil	Whole Community DNA	PCR	Amplicon	pyrotag library	454 GS FLX Titanium	European Nucleotide Archive	https://www.ebi.ac.uk/ena/data/search?query=PRJEB12501	ERS1039848	SAMEA3732699	ERX1298185	ERR1225973	ITS	ITS2	TACGCTGTCT	TCCTCCGCTTATTGATATGC	GCATCGATGAAGAACGCAGC	2012-03-12	31.11	-95.15	USA	0.3	88	19.0	253	Aquic Glossudalfs	Loblolly Pine, Beautyberry, Yaupon, Sweetgum, Oaks, Wax Myrtle	Cfa, Humid subtropical	OM3	C0	0	A horizon	0.0	0.8	0.0	4.4	1.3	17.8
TXA030F	TXA030	LP_TX_	Texas	TXA	Forest Soil	Whole Community DNA	PCR	Amplicon	pyrotag library	454 GS FLX Titanium	European Nucleotide Archive	https://www.ebi.ac.uk/ena/data/search?query=PRJEB12501	ERS1039849	SAMEA3732700	ERX1298186	ERR1225974	ITS	ITS2	ACGCGAGTAT	TCCTCCGCTTATTGATATGC	GCATCGATGAAGAACGCAGC	2012-03-12	31.11	-95.15	USA	0.1	88	19.0	253	Aquic Glossudalfs	Loblolly Pine, Beautyberry, Yaupon, Sweetgum, Oaks, Wax Myrtle	Cfa, Humid subtropical	OM3	C0	0	O horizon	0.0	NA	NA	4.4	NA	NA
TXA031F	TXA031	LP_TX_	Texas	TXA	Forest Soil	Whole Community DNA	PCR	Amplicon	pyrotag library	454 GS FLX Titanium	European Nucleotide Archive	https://www.ebi.ac.uk/ena/data/search?query=PRJEB12501	ERS1039850	SAMEA3732701	ERX1298187	ERR1225975	ITS	ITS2	TCGATCACGT	TCCTCCGCTTATTGATATGC	GCATCGATGAAGAACGCAGC	2012-03-12	31.11	-95.15	USA	0.3	88	19.0	253	Aquic Glossudalfs	Loblolly Pine, Beautyberry, Yaupon, Sweetgum, Oaks, Wax Myrtle	Cfa, Humid subtropical	OM3	C0	1	A horizon	0.0	0.8	0.0	5.3	1.3	17.8
TXA032F	TXA032	LP_TX_	Texas	TXA	Forest Soil	Whole Community DNA	PCR	Amplicon	pyrotag library	454 GS FLX Titanium	European Nucleotide Archive	https://www.ebi.ac.uk/ena/data/search?query=PRJEB12501	ERS1039851	SAMEA3732702	ERX1298188	ERR1225976	ITS	ITS2	TACACGTGAT	TCCTCCGCTTATTGATATGC	GCATCGATGAAGAACGCAGC	2012-03-12	31.11	-95.15	USA	0.3	88	19.0	253	Aquic Glossudalfs	Loblolly Pine, Beautyberry, Yaupon, Sweetgum, Oaks, Wax Myrtle	Cfa, Humid subtropical	OM3	C0	1	A horizon	0.0	0.8	0.0	4.6	1.3	17.8
TXA033F	TXA033	LP_TX_	Texas	TXA	Forest Soil	Whole Community DNA	PCR	Amplicon	pyrotag library	454 GS FLX Titanium	European Nucleotide Archive	https://www.ebi.ac.uk/ena/data/search?query=PRJEB12501	ERS1039852	SAMEA3732703	ERX1298189	ERR1225977	ITS	ITS2	ACGCGAGTAT	TCCTCCGCTTATTGATATGC	GCATCGATGAAGAACGCAGC	2012-03-12	31.11	-95.15	USA	0.3	88	19.0	253	Aquic Glossudalfs	Loblolly Pine, Beautyberry, Yaupon, Sweetgum, Oaks, Wax Myrtle	Cfa, Humid subtropical	OM3	C0	1	A horizon	0.0	0.8	0.0	4.5	1.3	17.8
TXA034F	TXA034	LP_TX_	Texas	TXA	Forest Soil	Whole Community DNA	PCR	Amplicon	pyrotag library	454 GS FLX Titanium	European Nucleotide Archive	https://www.ebi.ac.uk/ena/data/search?query=PRJEB12501	ERS1039853	SAMEA3732704	ERX1298190	ERR1225978	ITS	ITS2	AGCGTCGTCT	TCCTCCGCTTATTGATATGC	GCATCGATGAAGAACGCAGC	2012-03-12	31.11	-95.15	USA	0.1	88	19.0	253	Aquic Glossudalfs	Loblolly Pine, Beautyberry, Yaupon, Sweetgum, Oaks, Wax Myrtle	Cfa, Humid subtropical	OM3	C0	1	O horizon	0.0	NA	NA	4.5	NA	NA
TXA036F	TXA036	LP_TX_	Texas	TXA	Forest Soil	Whole Community DNA	PCR	Amplicon	pyrotag library	454 GS FLX Titanium	European Nucleotide Archive	https://www.ebi.ac.uk/ena/data/search?query=PRJEB12501	ERS1039854	SAMEA3732705	ERX1298191	ERR1225979	ITS	ITS2	ACTACTATGT	TCCTCCGCTTATTGATATGC	GCATCGATGAAGAACGCAGC	2012-03-12	31.11	-95.15	USA	0.1	88	19.0	253	Aquic Glossudalfs	Loblolly Pine, Beautyberry, Yaupon, Sweetgum, Oaks, Wax Myrtle	Cfa, Humid subtropical	OM3	C0	1	O horizon	0.0	NA	NA	4.5	NA	NA
TXA037F	TXA037	LP_TX_	Texas	TXA	Forest Soil	Whole Community DNA	PCR	Amplicon	pyrotag library	454 GS FLX Titanium	European Nucleotide Archive	https://www.ebi.ac.uk/ena/data/search?query=PRJEB12501	ERS1039855	SAMEA3732706	ERX1298192	ERR1225980	ITS	ITS2	ACTGTACAGT	TCCTCCGCTTATTGATATGC	GCATCGATGAAGAACGCAGC	2012-03-12	31.11	-95.15	USA	0.3	88	19.0	253	Aquic Glossudalfs	Loblolly Pine, Beautyberry, Yaupon, Sweetgum, Oaks, Wax Myrtle	Cfa, Humid subtropical	REF	REF	0	A horizon	0.0	NA	NA	0.0	NA	NA
TXA038F	TXA038	LP_TX_	Texas	TXA	Forest Soil	Whole Community DNA	PCR	Amplicon	pyrotag library	454 GS FLX Titanium	European Nucleotide Archive	https://www.ebi.ac.uk/ena/data/search?query=PRJEB12501	ERS1039856	SAMEA3732707	ERX1298193	ERR1225981	ITS	ITS2	TAGTGTAGAT	TCCTCCGCTTATTGATATGC	GCATCGATGAAGAACGCAGC	2012-03-12	31.11	-95.15	USA	0.3	88	19.0	253	Aquic Glossudalfs	Loblolly Pine, Beautyberry, Yaupon, Sweetgum, Oaks, Wax Myrtle	Cfa, Humid subtropical	REF	REF	0	A horizon	0.0	NA	NA	0.0	NA	NA
TXA040F	TXA040	LP_TX_	Texas	TXA	Forest Soil	Whole Community DNA	PCR	Amplicon	pyrotag library	454 GS FLX Titanium	European Nucleotide Archive	https://www.ebi.ac.uk/ena/data/search?query=PRJEB12501	ERS1039857	SAMEA3732708	ERX1298194	ERR1225982	ITS	ITS2	CGTCTAGTAC	TCCTCCGCTTATTGATATGC	GCATCGATGAAGAACGCAGC	2012-03-12	31.11	-95.15	USA	0.1	88	19.0	253	Aquic Glossudalfs	Loblolly Pine, Beautyberry, Yaupon, Sweetgum, Oaks, Wax Myrtle	Cfa, Humid subtropical	REF	REF	0	O horizon	0.0	NA	NA	4.7	NA	NA
TXA041F	TXA041	LP_TX_	Texas	TXA	Forest Soil	Whole Community DNA	PCR	Amplicon	pyrotag library	454 GS FLX Titanium	European Nucleotide Archive	https://www.ebi.ac.uk/ena/data/search?query=PRJEB12501	ERS1039858	SAMEA3732709	ERX1298195	ERR1225983	ITS	ITS2	TACACACACT	TCCTCCGCTTATTGATATGC	GCATCGATGAAGAACGCAGC	2012-03-12	31.11	-95.15	USA	0.1	88	19.0	253	Aquic Glossudalfs	Loblolly Pine, Beautyberry, Yaupon, Sweetgum, Oaks, Wax Myrtle	Cfa, Humid subtropical	REF	REF	0	O horizon	0.0	NA	NA	4.4	NA	NA
TXB043F	TXB043	LP_TX_	Texas	TXB	Forest Soil	Whole Community DNA	PCR	Amplicon	pyrotag library	454 GS FLX Titanium	European Nucleotide Archive	https://www.ebi.ac.uk/ena/data/search?query=PRJEB12501	ERS1039859	SAMEA3732710	ERX1298196	ERR1225984	ITS	ITS2	TCTACGTAGC	TCCTCCGCTTATTGATATGC	GCATCGATGAAGAACGCAGC	2012-03-12	31.11	-95.15	USA	0.3	88	19.0	253	Aquic Glossudalfs	Loblolly Pine, Beautyberry, Yaupon, Sweetgum, Oaks, Wax Myrtle	Cfa, Humid subtropical	OM1	C0	0	A horizon	0.0	0.9	0.1	5.1	1.3	15.5
TXB045F	TXB045	LP_TX_	Texas	TXB	Forest Soil	Whole Community DNA	PCR	Amplicon	pyrotag library	454 GS FLX Titanium	European Nucleotide Archive	https://www.ebi.ac.uk/ena/data/search?query=PRJEB12501	ERS1039860	SAMEA3732711	ERX1298197	ERR1225985	ITS	ITS2	CGACGTGACT	TCCTCCGCTTATTGATATGC	GCATCGATGAAGAACGCAGC	2012-03-12	31.11	-95.15	USA	0.3	88	19.0	253	Aquic Glossudalfs	Loblolly Pine, Beautyberry, Yaupon, Sweetgum, Oaks, Wax Myrtle	Cfa, Humid subtropical	OM1	C0	0	A horizon	0.0	0.9	0.1	4.9	1.3	15.5
TXB046F	TXB046	LP_TX_	Texas	TXB	Forest Soil	Whole Community DNA	PCR	Amplicon	pyrotag library	454 GS FLX Titanium	European Nucleotide Archive	https://www.ebi.ac.uk/ena/data/search?query=PRJEB12501	ERS1039861	SAMEA3732712	ERX1298198	ERR1225986	ITS	ITS2	TCGATCACGT	TCCTCCGCTTATTGATATGC	GCATCGATGAAGAACGCAGC	2012-03-12	31.11	-95.15	USA	0.1	88	19.0	253	Aquic Glossudalfs	Loblolly Pine, Beautyberry, Yaupon, Sweetgum, Oaks, Wax Myrtle	Cfa, Humid subtropical	OM1	C0	0	O horizon	0.0	NA	NA	5.0	NA	NA
TXB047F	TXB047	LP_TX_	Texas	TXB	Forest Soil	Whole Community DNA	PCR	Amplicon	pyrotag library	454 GS FLX Titanium	European Nucleotide Archive	https://www.ebi.ac.uk/ena/data/search?query=PRJEB12501	ERS1039862	SAMEA3732713	ERX1298199	ERR1225987	ITS	ITS2	CTCGCGTGTC	TCCTCCGCTTATTGATATGC	GCATCGATGAAGAACGCAGC	2012-03-12	31.11	-95.15	USA	0.1	88	19.0	253	Aquic Glossudalfs	Loblolly Pine, Beautyberry, Yaupon, Sweetgum, Oaks, Wax Myrtle	Cfa, Humid subtropical	OM1	C0	0	O horizon	0.0	NA	NA	4.9	NA	NA
TXB048F	TXB048	LP_TX_	Texas	TXB	Forest Soil	Whole Community DNA	PCR	Amplicon	pyrotag library	454 GS FLX Titanium	European Nucleotide Archive	https://www.ebi.ac.uk/ena/data/search?query=PRJEB12501	ERS1039863	SAMEA3732714	ERX1298200	ERR1225988	ITS	ITS2	TGATACGTCT	TCCTCCGCTTATTGATATGC	GCATCGATGAAGAACGCAGC	2012-03-12	31.11	-95.15	USA	0.1	88	19.0	253	Aquic Glossudalfs	Loblolly Pine, Beautyberry, Yaupon, Sweetgum, Oaks, Wax Myrtle	Cfa, Humid subtropical	OM1	C0	0	O horizon	0.0	NA	NA	5.0	NA	NA
TXB049F	TXB049	LP_TX_	Texas	TXB	Forest Soil	Whole Community DNA	PCR	Amplicon	pyrotag library	454 GS FLX Titanium	European Nucleotide Archive	https://www.ebi.ac.uk/ena/data/search?query=PRJEB12501	ERS1039864	SAMEA3732715	ERX1298201	ERR1225989	ITS	ITS2	AGCACTGTAG	TCCTCCGCTTATTGATATGC	GCATCGATGAAGAACGCAGC	2012-03-12	31.11	-95.15	USA	0.3	88	19.0	253	Aquic Glossudalfs	Loblolly Pine, Beautyberry, Yaupon, Sweetgum, Oaks, Wax Myrtle	Cfa, Humid subtropical	OM1	C0	1	A horizon	0.0	0.9	0.1	5.0	1.3	15.5
TXB050F	TXB050	LP_TX_	Texas	TXB	Forest Soil	Whole Community DNA	PCR	Amplicon	pyrotag library	454 GS FLX Titanium	European Nucleotide Archive	https://www.ebi.ac.uk/ena/data/search?query=PRJEB12501	ERS1039865	SAMEA3732716	ERX1298202	ERR1225990	ITS	ITS2	TCTCTATGCG	TCCTCCGCTTATTGATATGC	GCATCGATGAAGAACGCAGC	2012-03-12	31.11	-95.15	USA	0.3	88	19.0	253	Aquic Glossudalfs	Loblolly Pine, Beautyberry, Yaupon, Sweetgum, Oaks, Wax Myrtle	Cfa, Humid subtropical	OM1	C0	1	A horizon	0.0	0.9	0.1	4.7	1.3	15.5
TXB051F	TXB051	LP_TX_	Texas	TXB	Forest Soil	Whole Community DNA	PCR	Amplicon	pyrotag library	454 GS FLX Titanium	European Nucleotide Archive	https://www.ebi.ac.uk/ena/data/search?query=PRJEB12501	ERS1039866	SAMEA3732717	ERX1298203	ERR1225991	ITS	ITS2	TGATACGTCT	TCCTCCGCTTATTGATATGC	GCATCGATGAAGAACGCAGC	2012-03-12	31.11	-95.15	USA	0.3	88	19.0	253	Aquic Glossudalfs	Loblolly Pine, Beautyberry, Yaupon, Sweetgum, Oaks, Wax Myrtle	Cfa, Humid subtropical	OM1	C0	1	A horizon	0.0	0.9	0.1	4.8	1.3	15.5
TXB052F	TXB052	LP_TX_	Texas	TXB	Forest Soil	Whole Community DNA	PCR	Amplicon	pyrotag library	454 GS FLX Titanium	European Nucleotide Archive	https://www.ebi.ac.uk/ena/data/search?query=PRJEB12501	ERS1039867	SAMEA3732718	ERX1298204	ERR1225992	ITS	ITS2	TACACACACT	TCCTCCGCTTATTGATATGC	GCATCGATGAAGAACGCAGC	2012-03-12	31.11	-95.15	USA	0.1	88	19.0	253	Aquic Glossudalfs	Loblolly Pine, Beautyberry, Yaupon, Sweetgum, Oaks, Wax Myrtle	Cfa, Humid subtropical	OM1	C0	1	O horizon	0.0	NA	NA	5.3	NA	NA
TXB054F	TXB054	LP_TX_	Texas	TXB	Forest Soil	Whole Community DNA	PCR	Amplicon	pyrotag library	454 GS FLX Titanium	European Nucleotide Archive	https://www.ebi.ac.uk/ena/data/search?query=PRJEB12501	ERS1039868	SAMEA3732719	ERX1298205	ERR1225993	ITS	ITS2	AGCGTCGTCT	TCCTCCGCTTATTGATATGC	GCATCGATGAAGAACGCAGC	2012-03-12	31.11	-95.15	USA	0.1	88	19.0	253	Aquic Glossudalfs	Loblolly Pine, Beautyberry, Yaupon, Sweetgum, Oaks, Wax Myrtle	Cfa, Humid subtropical	OM1	C0	1	O horizon	0.0	NA	NA	4.9	NA	NA
TXB055F	TXB055	LP_TX_	Texas	TXB	Forest Soil	Whole Community DNA	PCR	Amplicon	pyrotag library	454 GS FLX Titanium	European Nucleotide Archive	https://www.ebi.ac.uk/ena/data/search?query=PRJEB12501	ERS1039869	SAMEA3732720	ERX1298206	ERR1225994	ITS	ITS2	CGTGTCTCTA	TCCTCCGCTTATTGATATGC	GCATCGATGAAGAACGCAGC	2012-03-12	31.11	-95.15	USA	0.3	88	19.0	253	Aquic Glossudalfs	Loblolly Pine, Beautyberry, Yaupon, Sweetgum, Oaks, Wax Myrtle	Cfa, Humid subtropical	OM2	C0	0	A horizon	0.0	1.0	0.1	5.6	1.2	19.4
TXB056F	TXB056	LP_TX_	Texas	TXB	Forest Soil	Whole Community DNA	PCR	Amplicon	pyrotag library	454 GS FLX Titanium	European Nucleotide Archive	https://www.ebi.ac.uk/ena/data/search?query=PRJEB12501	ERS1039870	SAMEA3732721	ERX1298207	ERR1225995	ITS	ITS2	TACACGTGAT	TCCTCCGCTTATTGATATGC	GCATCGATGAAGAACGCAGC	2012-03-12	31.11	-95.15	USA	0.3	88	19.0	253	Aquic Glossudalfs	Loblolly Pine, Beautyberry, Yaupon, Sweetgum, Oaks, Wax Myrtle	Cfa, Humid subtropical	OM2	C0	0	A horizon	0.0	1.0	0.1	5.4	1.2	19.4
TXB057F	TXB057	LP_TX_	Texas	TXB	Forest Soil	Whole Community DNA	PCR	Amplicon	pyrotag library	454 GS FLX Titanium	European Nucleotide Archive	https://www.ebi.ac.uk/ena/data/search?query=PRJEB12501	ERS1039871	SAMEA3732722	ERX1298208	ERR1225996	ITS	ITS2	TAGTGTAGAT	TCCTCCGCTTATTGATATGC	GCATCGATGAAGAACGCAGC	2012-03-12	31.11	-95.15	USA	0.3	88	19.0	253	Aquic Glossudalfs	Loblolly Pine, Beautyberry, Yaupon, Sweetgum, Oaks, Wax Myrtle	Cfa, Humid subtropical	OM2	C0	0	A horizon	0.0	1.0	0.1	5.6	1.2	19.4
TXB060F	TXB060	LP_TX_	Texas	TXB	Forest Soil	Whole Community DNA	PCR	Amplicon	pyrotag library	454 GS FLX Titanium	European Nucleotide Archive	https://www.ebi.ac.uk/ena/data/search?query=PRJEB12501	ERS1039872	SAMEA3732723	ERX1298209	ERR1225997	ITS	ITS2	AGCACTGTAG	TCCTCCGCTTATTGATATGC	GCATCGATGAAGAACGCAGC	2012-03-12	31.11	-95.15	USA	0.1	88	19.0	253	Aquic Glossudalfs	Loblolly Pine, Beautyberry, Yaupon, Sweetgum, Oaks, Wax Myrtle	Cfa, Humid subtropical	OM2	C0	0	O horizon	0.0	NA	NA	5.1	NA	NA
TXB061F	TXB061	LP_TX_	Texas	TXB	Forest Soil	Whole Community DNA	PCR	Amplicon	pyrotag library	454 GS FLX Titanium	European Nucleotide Archive	https://www.ebi.ac.uk/ena/data/search?query=PRJEB12501	ERS1039873	SAMEA3732724	ERX1298210	ERR1225998	ITS	ITS2	TAGTGTAGAT	TCCTCCGCTTATTGATATGC	GCATCGATGAAGAACGCAGC	2012-03-12	31.11	-95.15	USA	0.3	88	19.0	253	Aquic Glossudalfs	Loblolly Pine, Beautyberry, Yaupon, Sweetgum, Oaks, Wax Myrtle	Cfa, Humid subtropical	OM2	C0	1	A horizon	0.0	1.0	0.1	5.0	1.2	19.4
TXB063F	TXB063	LP_TX_	Texas	TXB	Forest Soil	Whole Community DNA	PCR	Amplicon	pyrotag library	454 GS FLX Titanium	European Nucleotide Archive	https://www.ebi.ac.uk/ena/data/search?query=PRJEB12501	ERS1039874	SAMEA3732725	ERX1298211	ERR1225999	ITS	ITS2	TCTACGTAGC	TCCTCCGCTTATTGATATGC	GCATCGATGAAGAACGCAGC	2012-03-12	31.11	-95.15	USA	0.3	88	19.0	253	Aquic Glossudalfs	Loblolly Pine, Beautyberry, Yaupon, Sweetgum, Oaks, Wax Myrtle	Cfa, Humid subtropical	OM2	C0	1	A horizon	0.0	1.0	0.1	4.8	1.2	19.4
TXB064F	TXB064	LP_TX_	Texas	TXB	Forest Soil	Whole Community DNA	PCR	Amplicon	pyrotag library	454 GS FLX Titanium	European Nucleotide Archive	https://www.ebi.ac.uk/ena/data/search?query=PRJEB12501	ERS1039875	SAMEA3732726	ERX1298212	ERR1226000	ITS	ITS2	AGACTATACT	TCCTCCGCTTATTGATATGC	GCATCGATGAAGAACGCAGC	2012-03-12	31.11	-95.15	USA	0.1	88	19.0	253	Aquic Glossudalfs	Loblolly Pine, Beautyberry, Yaupon, Sweetgum, Oaks, Wax Myrtle	Cfa, Humid subtropical	OM2	C0	1	O horizon	0.0	NA	NA	4.4	NA	NA
TXB065F	TXB065	LP_TX_	Texas	TXB	Forest Soil	Whole Community DNA	PCR	Amplicon	pyrotag library	454 GS FLX Titanium	European Nucleotide Archive	https://www.ebi.ac.uk/ena/data/search?query=PRJEB12501	ERS1039876	SAMEA3732727	ERX1298213	ERR1226001	ITS	ITS2	TAGTGTAGAT	TCCTCCGCTTATTGATATGC	GCATCGATGAAGAACGCAGC	2012-03-12	31.11	-95.15	USA	0.1	88	19.0	253	Aquic Glossudalfs	Loblolly Pine, Beautyberry, Yaupon, Sweetgum, Oaks, Wax Myrtle	Cfa, Humid subtropical	OM2	C0	1	O horizon	0.0	NA	NA	5.0	NA	NA
TXB066F	TXB066	LP_TX_	Texas	TXB	Forest Soil	Whole Community DNA	PCR	Amplicon	pyrotag library	454 GS FLX Titanium	European Nucleotide Archive	https://www.ebi.ac.uk/ena/data/search?query=PRJEB12501	ERS1039877	SAMEA3732728	ERX1298214	ERR1226002	ITS	ITS2	ATACGACGTA	TCCTCCGCTTATTGATATGC	GCATCGATGAAGAACGCAGC	2012-03-12	31.11	-95.15	USA	0.1	88	19.0	253	Aquic Glossudalfs	Loblolly Pine, Beautyberry, Yaupon, Sweetgum, Oaks, Wax Myrtle	Cfa, Humid subtropical	OM2	C0	1	O horizon	0.0	NA	NA	4.7	NA	NA
TXB067F	TXB067	LP_TX_	Texas	TXB	Forest Soil	Whole Community DNA	PCR	Amplicon	pyrotag library	454 GS FLX Titanium	European Nucleotide Archive	https://www.ebi.ac.uk/ena/data/search?query=PRJEB12501	ERS1039878	SAMEA3732729	ERX1298215	ERR1226003	ITS	ITS2	ACGAGTGCGT	TCCTCCGCTTATTGATATGC	GCATCGATGAAGAACGCAGC	2012-03-12	31.11	-95.15	USA	0.3	88	19.0	253	Aquic Glossudalfs	Loblolly Pine, Beautyberry, Yaupon, Sweetgum, Oaks, Wax Myrtle	Cfa, Humid subtropical	OM3	C0	0	A horizon	0.0	1.0	0.1	4.5	1.3	20.1
TXB070F	TXB070	LP_TX_	Texas	TXB	Forest Soil	Whole Community DNA	PCR	Amplicon	pyrotag library	454 GS FLX Titanium	European Nucleotide Archive	https://www.ebi.ac.uk/ena/data/search?query=PRJEB12501	ERS1039879	SAMEA3732730	ERX1298216	ERR1226004	ITS	ITS2	TACACACACT	TCCTCCGCTTATTGATATGC	GCATCGATGAAGAACGCAGC	2012-03-12	31.11	-95.15	USA	0.1	88	19.0	253	Aquic Glossudalfs	Loblolly Pine, Beautyberry, Yaupon, Sweetgum, Oaks, Wax Myrtle	Cfa, Humid subtropical	OM3	C0	0	O horizon	0.0	NA	NA	4.3	NA	NA
TXB071F	TXB071	LP_TX_	Texas	TXB	Forest Soil	Whole Community DNA	PCR	Amplicon	pyrotag library	454 GS FLX Titanium	European Nucleotide Archive	https://www.ebi.ac.uk/ena/data/search?query=PRJEB12501	ERS1039880	SAMEA3732731	ERX1298217	ERR1226005	ITS	ITS2	TCGATCACGT	TCCTCCGCTTATTGATATGC	GCATCGATGAAGAACGCAGC	2012-03-12	31.11	-95.15	USA	0.1	88	19.0	253	Aquic Glossudalfs	Loblolly Pine, Beautyberry, Yaupon, Sweetgum, Oaks, Wax Myrtle	Cfa, Humid subtropical	OM3	C0	0	O horizon	0.0	NA	NA	4.5	NA	NA
TXB073F	TXB073	LP_TX_	Texas	TXB	Forest Soil	Whole Community DNA	PCR	Amplicon	pyrotag library	454 GS FLX Titanium	European Nucleotide Archive	https://www.ebi.ac.uk/ena/data/search?query=PRJEB12501	ERS1039881	SAMEA3732732	ERX1298218	ERR1226006	ITS	ITS2	ATATCGCGAG	TCCTCCGCTTATTGATATGC	GCATCGATGAAGAACGCAGC	2012-03-12	31.11	-95.15	USA	0.3	88	19.0	253	Aquic Glossudalfs	Loblolly Pine, Beautyberry, Yaupon, Sweetgum, Oaks, Wax Myrtle	Cfa, Humid subtropical	OM3	C0	1	A horizon	0.0	1.0	0.1	5.0	1.3	20.1
TXB076F	TXB076	LP_TX_	Texas	TXB	Forest Soil	Whole Community DNA	PCR	Amplicon	pyrotag library	454 GS FLX Titanium	European Nucleotide Archive	https://www.ebi.ac.uk/ena/data/search?query=PRJEB12501	ERS1039882	SAMEA3732733	ERX1298219	ERR1226007	ITS	ITS2	TCTCTATGCG	TCCTCCGCTTATTGATATGC	GCATCGATGAAGAACGCAGC	2012-03-12	31.11	-95.15	USA	0.1	88	19.0	253	Aquic Glossudalfs	Loblolly Pine, Beautyberry, Yaupon, Sweetgum, Oaks, Wax Myrtle	Cfa, Humid subtropical	OM3	C0	1	O horizon	0.0	NA	NA	4.3	NA	NA
TXB077F	TXB077	LP_TX_	Texas	TXB	Forest Soil	Whole Community DNA	PCR	Amplicon	pyrotag library	454 GS FLX Titanium	European Nucleotide Archive	https://www.ebi.ac.uk/ena/data/search?query=PRJEB12501	ERS1039883	SAMEA3732734	ERX1298220	ERR1226008	ITS	ITS2	CGTGTCTCTA	TCCTCCGCTTATTGATATGC	GCATCGATGAAGAACGCAGC	2012-03-12	31.11	-95.15	USA	0.1	88	19.0	253	Aquic Glossudalfs	Loblolly Pine, Beautyberry, Yaupon, Sweetgum, Oaks, Wax Myrtle	Cfa, Humid subtropical	OM3	C0	1	O horizon	0.0	NA	NA	4.4	NA	NA
TXB078F	TXB078	LP_TX_	Texas	TXB	Forest Soil	Whole Community DNA	PCR	Amplicon	pyrotag library	454 GS FLX Titanium	European Nucleotide Archive	https://www.ebi.ac.uk/ena/data/search?query=PRJEB12501	ERS1039884	SAMEA3732735	ERX1298221	ERR1226009	ITS	ITS2	ACGAGTGCGT	TCCTCCGCTTATTGATATGC	GCATCGATGAAGAACGCAGC	2012-03-12	31.11	-95.15	USA	0.1	88	19.0	253	Aquic Glossudalfs	Loblolly Pine, Beautyberry, Yaupon, Sweetgum, Oaks, Wax Myrtle	Cfa, Humid subtropical	OM3	C0	1	O horizon	0.0	NA	NA	4.3	NA	NA
TXB079F	TXB079	LP_TX_	Texas	TXB	Forest Soil	Whole Community DNA	PCR	Amplicon	pyrotag library	454 GS FLX Titanium	European Nucleotide Archive	https://www.ebi.ac.uk/ena/data/search?query=PRJEB12501	ERS1039885	SAMEA3732736	ERX1298222	ERR1226010	ITS	ITS2	TCGATCACGT	TCCTCCGCTTATTGATATGC	GCATCGATGAAGAACGCAGC	2012-03-12	31.11	-95.15	USA	0.3	88	19.0	253	Aquic Glossudalfs	Loblolly Pine, Beautyberry, Yaupon, Sweetgum, Oaks, Wax Myrtle	Cfa, Humid subtropical	REF	REF	0	A horizon	0.0	NA	NA	0.0	NA	NA
TXB080F	TXB080	LP_TX_	Texas	TXB	Forest Soil	Whole Community DNA	PCR	Amplicon	pyrotag library	454 GS FLX Titanium	European Nucleotide Archive	https://www.ebi.ac.uk/ena/data/search?query=PRJEB12501	ERS1039886	SAMEA3732737	ERX1298223	ERR1226011	ITS	ITS2	TACACGTGAT	TCCTCCGCTTATTGATATGC	GCATCGATGAAGAACGCAGC	2012-03-12	31.11	-95.15	USA	0.3	88	19.0	253	Aquic Glossudalfs	Loblolly Pine, Beautyberry, Yaupon, Sweetgum, Oaks, Wax Myrtle	Cfa, Humid subtropical	REF	REF	0	A horizon	0.0	NA	NA	0.0	NA	NA
TXB081F	TXB081	LP_TX_	Texas	TXB	Forest Soil	Whole Community DNA	PCR	Amplicon	pyrotag library	454 GS FLX Titanium	European Nucleotide Archive	https://www.ebi.ac.uk/ena/data/search?query=PRJEB12501	ERS1039887	SAMEA3732738	ERX1298224	ERR1226012	ITS	ITS2	ACGCTCGACA	TCCTCCGCTTATTGATATGC	GCATCGATGAAGAACGCAGC	2012-03-12	31.11	-95.15	USA	0.3	88	19.0	253	Aquic Glossudalfs	Loblolly Pine, Beautyberry, Yaupon, Sweetgum, Oaks, Wax Myrtle	Cfa, Humid subtropical	REF	REF	0	A horizon	0.0	NA	NA	0.0	NA	NA
TXB082F	TXB082	LP_TX_	Texas	TXB	Forest Soil	Whole Community DNA	PCR	Amplicon	pyrotag library	454 GS FLX Titanium	European Nucleotide Archive	https://www.ebi.ac.uk/ena/data/search?query=PRJEB12501	ERS1039888	SAMEA3732739	ERX1298225	ERR1226013	ITS	ITS2	CACGCTACGT	TCCTCCGCTTATTGATATGC	GCATCGATGAAGAACGCAGC	2012-03-12	31.11	-95.15	USA	0.1	88	19.0	253	Aquic Glossudalfs	Loblolly Pine, Beautyberry, Yaupon, Sweetgum, Oaks, Wax Myrtle	Cfa, Humid subtropical	REF	REF	0	O horizon	0.0	NA	NA	4.5	NA	NA
TXB083F	TXB083	LP_TX_	Texas	TXB	Forest Soil	Whole Community DNA	PCR	Amplicon	pyrotag library	454 GS FLX Titanium	European Nucleotide Archive	https://www.ebi.ac.uk/ena/data/search?query=PRJEB12501	ERS1039889	SAMEA3732740	ERX1298226	ERR1226014	ITS	ITS2	AGACTATACT	TCCTCCGCTTATTGATATGC	GCATCGATGAAGAACGCAGC	2012-03-12	31.11	-95.15	USA	0.1	88	19.0	253	Aquic Glossudalfs	Loblolly Pine, Beautyberry, Yaupon, Sweetgum, Oaks, Wax Myrtle	Cfa, Humid subtropical	REF	REF	0	O horizon	0.0	NA	NA	4.4	NA	NA
TXB084F	TXB084	LP_TX_	Texas	TXB	Forest Soil	Whole Community DNA	PCR	Amplicon	pyrotag library	454 GS FLX Titanium	European Nucleotide Archive	https://www.ebi.ac.uk/ena/data/search?query=PRJEB12501	ERS1039890	SAMEA3732741	ERX1298227	ERR1226015	ITS	ITS2	TACGCTGTCT	TCCTCCGCTTATTGATATGC	GCATCGATGAAGAACGCAGC	2012-03-12	31.11	-95.15	USA	0.1	88	19.0	253	Aquic Glossudalfs	Loblolly Pine, Beautyberry, Yaupon, Sweetgum, Oaks, Wax Myrtle	Cfa, Humid subtropical	REF	REF	0	O horizon	0.0	NA	NA	4.9	NA	NA
TXB085F	TXB085	LP_TX_	Texas	TXB	Forest Soil	Whole Community DNA	PCR	Amplicon	pyrotag library	454 GS FLX Titanium	European Nucleotide Archive	https://www.ebi.ac.uk/ena/data/search?query=PRJEB12501	ERS1039891	SAMEA3732742	ERX1298228	ERR1226016	ITS	ITS2	CGACGTGACT	TCCTCCGCTTATTGATATGC	GCATCGATGAAGAACGCAGC	2012-03-12	31.11	-95.15	USA	0.3	88	19.0	253	Aquic Glossudalfs	Loblolly Pine, Beautyberry, Yaupon, Sweetgum, Oaks, Wax Myrtle	Cfa, Humid subtropical	OM1	C0	0	A horizon	0.1	0.9	0.1	5.0	1.3	17.4
TXB086F	TXB086	LP_TX_	Texas	TXB	Forest Soil	Whole Community DNA	PCR	Amplicon	pyrotag library	454 GS FLX Titanium	European Nucleotide Archive	https://www.ebi.ac.uk/ena/data/search?query=PRJEB12501	ERS1039892	SAMEA3732743	ERX1298229	ERR1226017	ITS	ITS2	ACTACTATGT	TCCTCCGCTTATTGATATGC	GCATCGATGAAGAACGCAGC	2012-03-12	31.11	-95.15	USA	0.3	88	19.0	253	Aquic Glossudalfs	Loblolly Pine, Beautyberry, Yaupon, Sweetgum, Oaks, Wax Myrtle	Cfa, Humid subtropical	OM1	C0	0	A horizon	0.1	0.9	0.1	4.7	1.3	17.4
TXB087F	TXB087	LP_TX_	Texas	TXB	Forest Soil	Whole Community DNA	PCR	Amplicon	pyrotag library	454 GS FLX Titanium	European Nucleotide Archive	https://www.ebi.ac.uk/ena/data/search?query=PRJEB12501	ERS1039893	SAMEA3732744	ERX1298230	ERR1226018	ITS	ITS2	CGAGAGATAC	TCCTCCGCTTATTGATATGC	GCATCGATGAAGAACGCAGC	2012-03-12	31.11	-95.15	USA	0.3	88	19.0	253	Aquic Glossudalfs	Loblolly Pine, Beautyberry, Yaupon, Sweetgum, Oaks, Wax Myrtle	Cfa, Humid subtropical	OM1	C0	0	A horizon	0.1	0.9	0.1	5.0	1.3	17.4
TXB090F	TXB090	LP_TX_	Texas	TXB	Forest Soil	Whole Community DNA	PCR	Amplicon	pyrotag library	454 GS FLX Titanium	European Nucleotide Archive	https://www.ebi.ac.uk/ena/data/search?query=PRJEB12501	ERS1039894	SAMEA3732745	ERX1298231	ERR1226019	ITS	ITS2	AGCGTCGTCT	TCCTCCGCTTATTGATATGC	GCATCGATGAAGAACGCAGC	2012-03-12	31.11	-95.15	USA	0.1	88	19.0	253	Aquic Glossudalfs	Loblolly Pine, Beautyberry, Yaupon, Sweetgum, Oaks, Wax Myrtle	Cfa, Humid subtropical	OM1	C0	0	O horizon	0.4	NA	NA	5.3	NA	NA
TXB091F	TXB091	LP_TX_	Texas	TXB	Forest Soil	Whole Community DNA	PCR	Amplicon	pyrotag library	454 GS FLX Titanium	European Nucleotide Archive	https://www.ebi.ac.uk/ena/data/search?query=PRJEB12501	ERS1039895	SAMEA3732746	ERX1298232	ERR1226020	ITS	ITS2	CAGTAGACGT	TCCTCCGCTTATTGATATGC	GCATCGATGAAGAACGCAGC	2012-03-12	31.11	-95.15	USA	0.3	88	19.0	253	Aquic Glossudalfs	Loblolly Pine, Beautyberry, Yaupon, Sweetgum, Oaks, Wax Myrtle	Cfa, Humid subtropical	OM1	C0	1	A horizon	0.1	0.9	0.1	5.2	1.3	17.4
TXB093F	TXB093	LP_TX_	Texas	TXB	Forest Soil	Whole Community DNA	PCR	Amplicon	pyrotag library	454 GS FLX Titanium	European Nucleotide Archive	https://www.ebi.ac.uk/ena/data/search?query=PRJEB12501	ERS1039896	SAMEA3732747	ERX1298233	ERR1226021	ITS	ITS2	TCACGTACTA	TCCTCCGCTTATTGATATGC	GCATCGATGAAGAACGCAGC	2012-03-12	31.11	-95.15	USA	0.3	88	19.0	253	Aquic Glossudalfs	Loblolly Pine, Beautyberry, Yaupon, Sweetgum, Oaks, Wax Myrtle	Cfa, Humid subtropical	OM1	C0	1	A horizon	0.1	0.9	0.1	5.4	1.3	17.4
TXB095F	TXB095	LP_TX_	Texas	TXB	Forest Soil	Whole Community DNA	PCR	Amplicon	pyrotag library	454 GS FLX Titanium	European Nucleotide Archive	https://www.ebi.ac.uk/ena/data/search?query=PRJEB12501	ERS1039897	SAMEA3732748	ERX1298234	ERR1226022	ITS	ITS2	CAGTAGACGT	TCCTCCGCTTATTGATATGC	GCATCGATGAAGAACGCAGC	2012-03-12	31.11	-95.15	USA	0.1	88	19.0	253	Aquic Glossudalfs	Loblolly Pine, Beautyberry, Yaupon, Sweetgum, Oaks, Wax Myrtle	Cfa, Humid subtropical	OM1	C0	1	O horizon	0.0	NA	NA	4.5	NA	NA
TXB096F	TXB096	LP_TX_	Texas	TXB	Forest Soil	Whole Community DNA	PCR	Amplicon	pyrotag library	454 GS FLX Titanium	European Nucleotide Archive	https://www.ebi.ac.uk/ena/data/search?query=PRJEB12501	ERS1039898	SAMEA3732749	ERX1298235	ERR1226023	ITS	ITS2	TCTACGTAGC	TCCTCCGCTTATTGATATGC	GCATCGATGAAGAACGCAGC	2012-03-12	31.11	-95.15	USA	0.1	88	19.0	253	Aquic Glossudalfs	Loblolly Pine, Beautyberry, Yaupon, Sweetgum, Oaks, Wax Myrtle	Cfa, Humid subtropical	OM1	C0	1	O horizon	0.3	NA	NA	5.0	NA	NA
TXC099F	TXC099	LP_TX_	Texas	TXC	Forest Soil	Whole Community DNA	PCR	Amplicon	pyrotag library	454 GS FLX Titanium	European Nucleotide Archive	https://www.ebi.ac.uk/ena/data/search?query=PRJEB12501	ERS1039900	SAMEA3732751	ERX1298237	ERR1226025	ITS	ITS2	ATCAGACACG	TCCTCCGCTTATTGATATGC	GCATCGATGAAGAACGCAGC	2012-03-12	31.11	-95.15	USA	0.3	88	19.0	253	Aquic Glossudalfs	Loblolly Pine, Beautyberry, Yaupon, Sweetgum, Oaks, Wax Myrtle	Cfa, Humid subtropical	OM2	C0	0	A horizon	0.0	0.9	0.1	5.9	1.3	17.4
TXC100F	TXC100	LP_TX_	Texas	TXC	Forest Soil	Whole Community DNA	PCR	Amplicon	pyrotag library	454 GS FLX Titanium	European Nucleotide Archive	https://www.ebi.ac.uk/ena/data/search?query=PRJEB12501	ERS1039901	SAMEA3732752	ERX1298238	ERR1226026	ITS	ITS2	AGCACTGTAG	TCCTCCGCTTATTGATATGC	GCATCGATGAAGAACGCAGC	2012-03-12	31.11	-95.15	USA	0.1	88	19.0	253	Aquic Glossudalfs	Loblolly Pine, Beautyberry, Yaupon, Sweetgum, Oaks, Wax Myrtle	Cfa, Humid subtropical	OM2	C0	0	O horizon	0.0	NA	NA	4.3	NA	NA
TXC101F	TXC101	LP_TX_	Texas	TXC	Forest Soil	Whole Community DNA	PCR	Amplicon	pyrotag library	454 GS FLX Titanium	European Nucleotide Archive	https://www.ebi.ac.uk/ena/data/search?query=PRJEB12501	ERS1039902	SAMEA3732753	ERX1298239	ERR1226027	ITS	ITS2	CGACGTGACT	TCCTCCGCTTATTGATATGC	GCATCGATGAAGAACGCAGC	2012-03-12	31.11	-95.15	USA	0.1	88	19.0	253	Aquic Glossudalfs	Loblolly Pine, Beautyberry, Yaupon, Sweetgum, Oaks, Wax Myrtle	Cfa, Humid subtropical	OM2	C0	0	O horizon	0.0	NA	NA	4.8	NA	NA
TXC102F	TXC102	LP_TX_	Texas	TXC	Forest Soil	Whole Community DNA	PCR	Amplicon	pyrotag library	454 GS FLX Titanium	European Nucleotide Archive	https://www.ebi.ac.uk/ena/data/search?query=PRJEB12501	ERS1039903	SAMEA3732754	ERX1298240	ERR1226028	ITS	ITS2	AGTACGCTAT	TCCTCCGCTTATTGATATGC	GCATCGATGAAGAACGCAGC	2012-03-12	31.11	-95.15	USA	0.1	88	19.0	253	Aquic Glossudalfs	Loblolly Pine, Beautyberry, Yaupon, Sweetgum, Oaks, Wax Myrtle	Cfa, Humid subtropical	OM2	C0	0	O horizon	0.0	NA	NA	4.6	NA	NA
TXC103F	TXC103	LP_TX_	Texas	TXC	Forest Soil	Whole Community DNA	PCR	Amplicon	pyrotag library	454 GS FLX Titanium	European Nucleotide Archive	https://www.ebi.ac.uk/ena/data/search?query=PRJEB12501	ERS1039904	SAMEA3732755	ERX1298241	ERR1226029	ITS	ITS2	CAGTAGACGT	TCCTCCGCTTATTGATATGC	GCATCGATGAAGAACGCAGC	2012-03-12	31.11	-95.15	USA	0.3	88	19.0	253	Aquic Glossudalfs	Loblolly Pine, Beautyberry, Yaupon, Sweetgum, Oaks, Wax Myrtle	Cfa, Humid subtropical	OM2	C0	1	A horizon	0.0	0.8	0.0	4.4	1.3	15.7
TXC104F	TXC104	LP_TX_	Texas	TXC	Forest Soil	Whole Community DNA	PCR	Amplicon	pyrotag library	454 GS FLX Titanium	European Nucleotide Archive	https://www.ebi.ac.uk/ena/data/search?query=PRJEB12501	ERS1039905	SAMEA3732756	ERX1298242	ERR1226030	ITS	ITS2	ATACGACGTA	TCCTCCGCTTATTGATATGC	GCATCGATGAAGAACGCAGC	2012-03-12	31.11	-95.15	USA	0.3	88	19.0	253	Aquic Glossudalfs	Loblolly Pine, Beautyberry, Yaupon, Sweetgum, Oaks, Wax Myrtle	Cfa, Humid subtropical	OM2	C0	1	A horizon	0.0	0.8	0.0	4.5	1.3	15.7
TXC105F	TXC105	LP_TX_	Texas	TXC	Forest Soil	Whole Community DNA	PCR	Amplicon	pyrotag library	454 GS FLX Titanium	European Nucleotide Archive	https://www.ebi.ac.uk/ena/data/search?query=PRJEB12501	ERS1039906	SAMEA3732757	ERX1298243	ERR1226031	ITS	ITS2	TCGCACTAGT	TCCTCCGCTTATTGATATGC	GCATCGATGAAGAACGCAGC	2012-03-12	31.11	-95.15	USA	0.3	88	19.0	253	Aquic Glossudalfs	Loblolly Pine, Beautyberry, Yaupon, Sweetgum, Oaks, Wax Myrtle	Cfa, Humid subtropical	OM2	C0	1	A horizon	0.1	0.8	0.0	4.5	1.3	15.7
TXC106F	TXC106	LP_TX_	Texas	TXC	Forest Soil	Whole Community DNA	PCR	Amplicon	pyrotag library	454 GS FLX Titanium	European Nucleotide Archive	https://www.ebi.ac.uk/ena/data/search?query=PRJEB12501	ERS1039907	SAMEA3732758	ERX1298244	ERR1226032	ITS	ITS2	AGACGCACTC	TCCTCCGCTTATTGATATGC	GCATCGATGAAGAACGCAGC	2012-03-12	31.11	-95.15	USA	0.1	88	19.0	253	Aquic Glossudalfs	Loblolly Pine, Beautyberry, Yaupon, Sweetgum, Oaks, Wax Myrtle	Cfa, Humid subtropical	OM2	C0	1	O horizon	0.0	NA	NA	4.6	NA	NA
TXC107F	TXC107	LP_TX_	Texas	TXC	Forest Soil	Whole Community DNA	PCR	Amplicon	pyrotag library	454 GS FLX Titanium	European Nucleotide Archive	https://www.ebi.ac.uk/ena/data/search?query=PRJEB12501	ERS1039908	SAMEA3732759	ERX1298245	ERR1226033	ITS	ITS2	TCGATCACGT	TCCTCCGCTTATTGATATGC	GCATCGATGAAGAACGCAGC	2012-03-12	31.11	-95.15	USA	0.1	88	19.0	253	Aquic Glossudalfs	Loblolly Pine, Beautyberry, Yaupon, Sweetgum, Oaks, Wax Myrtle	Cfa, Humid subtropical	OM2	C0	1	O horizon	0.0	NA	NA	4.5	NA	NA
TXC109F	TXC109	LP_TX_	Texas	TXC	Forest Soil	Whole Community DNA	PCR	Amplicon	pyrotag library	454 GS FLX Titanium	European Nucleotide Archive	https://www.ebi.ac.uk/ena/data/search?query=PRJEB12501	ERS1039909	SAMEA3732760	ERX1298246	ERR1226034	ITS	ITS2	ACTACTATGT	TCCTCCGCTTATTGATATGC	GCATCGATGAAGAACGCAGC	2012-03-12	31.11	-95.15	USA	0.3	88	19.0	253	Aquic Glossudalfs	Loblolly Pine, Beautyberry, Yaupon, Sweetgum, Oaks, Wax Myrtle	Cfa, Humid subtropical	OM1	C0	0	A horizon	0.0	0.8	0.0	4.8	1.3	16.3
TXC110F	TXC110	LP_TX_	Texas	TXC	Forest Soil	Whole Community DNA	PCR	Amplicon	pyrotag library	454 GS FLX Titanium	European Nucleotide Archive	https://www.ebi.ac.uk/ena/data/search?query=PRJEB12501	ERS1039910	SAMEA3732761	ERX1298247	ERR1226035	ITS	ITS2	TACAGATCGT	TCCTCCGCTTATTGATATGC	GCATCGATGAAGAACGCAGC	2012-03-12	31.11	-95.15	USA	0.3	88	19.0	253	Aquic Glossudalfs	Loblolly Pine, Beautyberry, Yaupon, Sweetgum, Oaks, Wax Myrtle	Cfa, Humid subtropical	OM1	C0	0	A horizon	0.0	0.8	0.0	4.9	1.3	16.3
TXC111F	TXC111	LP_TX_	Texas	TXC	Forest Soil	Whole Community DNA	PCR	Amplicon	pyrotag library	454 GS FLX Titanium	European Nucleotide Archive	https://www.ebi.ac.uk/ena/data/search?query=PRJEB12501	ERS1039911	SAMEA3732762	ERX1298248	ERR1226036	ITS	ITS2	CATAGTAGTG	TCCTCCGCTTATTGATATGC	GCATCGATGAAGAACGCAGC	2012-03-12	31.11	-95.15	USA	0.3	88	19.0	253	Aquic Glossudalfs	Loblolly Pine, Beautyberry, Yaupon, Sweetgum, Oaks, Wax Myrtle	Cfa, Humid subtropical	OM1	C0	0	A horizon	0.0	0.8	0.0	5.0	1.3	16.3
TXC112F	TXC112	LP_TX_	Texas	TXC	Forest Soil	Whole Community DNA	PCR	Amplicon	pyrotag library	454 GS FLX Titanium	European Nucleotide Archive	https://www.ebi.ac.uk/ena/data/search?query=PRJEB12501	ERS1039912	SAMEA3732763	ERX1298249	ERR1226037	ITS	ITS2	TACAGATCGT	TCCTCCGCTTATTGATATGC	GCATCGATGAAGAACGCAGC	2012-03-12	31.11	-95.15	USA	0.1	88	19.0	253	Aquic Glossudalfs	Loblolly Pine, Beautyberry, Yaupon, Sweetgum, Oaks, Wax Myrtle	Cfa, Humid subtropical	OM1	C0	0	O horizon	0.0	NA	NA	4.5	NA	NA
TXC113F	TXC113	LP_TX_	Texas	TXC	Forest Soil	Whole Community DNA	PCR	Amplicon	pyrotag library	454 GS FLX Titanium	European Nucleotide Archive	https://www.ebi.ac.uk/ena/data/search?query=PRJEB12501	ERS1039913	SAMEA3732764	ERX1298250	ERR1226038	ITS	ITS2	TACACACACT	TCCTCCGCTTATTGATATGC	GCATCGATGAAGAACGCAGC	2012-03-12	31.11	-95.15	USA	0.1	88	19.0	253	Aquic Glossudalfs	Loblolly Pine, Beautyberry, Yaupon, Sweetgum, Oaks, Wax Myrtle	Cfa, Humid subtropical	OM1	C0	0	O horizon	0.0	NA	NA	4.4	NA	NA
TXC114F	TXC114	LP_TX_	Texas	TXC	Forest Soil	Whole Community DNA	PCR	Amplicon	pyrotag library	454 GS FLX Titanium	European Nucleotide Archive	https://www.ebi.ac.uk/ena/data/search?query=PRJEB12501	ERS1039914	SAMEA3732765	ERX1298251	ERR1226039	ITS	ITS2	ATCAGACACG	TCCTCCGCTTATTGATATGC	GCATCGATGAAGAACGCAGC	2012-03-12	31.11	-95.15	USA	0.1	88	19.0	253	Aquic Glossudalfs	Loblolly Pine, Beautyberry, Yaupon, Sweetgum, Oaks, Wax Myrtle	Cfa, Humid subtropical	OM1	C0	0	O horizon	0.0	NA	NA	5.3	NA	NA
TXC115F	TXC115	LP_TX_	Texas	TXC	Forest Soil	Whole Community DNA	PCR	Amplicon	pyrotag library	454 GS FLX Titanium	European Nucleotide Archive	https://www.ebi.ac.uk/ena/data/search?query=PRJEB12501	ERS1039915	SAMEA3732766	ERX1298252	ERR1226040	ITS	ITS2	TACGCTGTCT	TCCTCCGCTTATTGATATGC	GCATCGATGAAGAACGCAGC	2012-03-12	31.11	-95.15	USA	0.3	88	19.0	253	Aquic Glossudalfs	Loblolly Pine, Beautyberry, Yaupon, Sweetgum, Oaks, Wax Myrtle	Cfa, Humid subtropical	OM1	C0	1	A horizon	0.0	0.8	0.0	5.3	1.3	16.3
TXC116F	TXC116	LP_TX_	Texas	TXC	Forest Soil	Whole Community DNA	PCR	Amplicon	pyrotag library	454 GS FLX Titanium	European Nucleotide Archive	https://www.ebi.ac.uk/ena/data/search?query=PRJEB12501	ERS1039916	SAMEA3732767	ERX1298253	ERR1226041	ITS	ITS2	ACGCTCGACA	TCCTCCGCTTATTGATATGC	GCATCGATGAAGAACGCAGC	2012-03-12	31.11	-95.15	USA	0.3	88	19.0	253	Aquic Glossudalfs	Loblolly Pine, Beautyberry, Yaupon, Sweetgum, Oaks, Wax Myrtle	Cfa, Humid subtropical	OM1	C0	1	A horizon	0.0	0.8	0.0	4.7	1.3	16.3
TXC117F	TXC117	LP_TX_	Texas	TXC	Forest Soil	Whole Community DNA	PCR	Amplicon	pyrotag library	454 GS FLX Titanium	European Nucleotide Archive	https://www.ebi.ac.uk/ena/data/search?query=PRJEB12501	ERS1039917	SAMEA3732768	ERX1298254	ERR1226042	ITS	ITS2	CAGTAGACGT	TCCTCCGCTTATTGATATGC	GCATCGATGAAGAACGCAGC	2012-03-12	31.11	-95.15	USA	0.3	88	19.0	253	Aquic Glossudalfs	Loblolly Pine, Beautyberry, Yaupon, Sweetgum, Oaks, Wax Myrtle	Cfa, Humid subtropical	OM1	C0	1	A horizon	0.0	0.8	0.0	5.2	1.3	16.3
TXC118F	TXC118	LP_TX_	Texas	TXC	Forest Soil	Whole Community DNA	PCR	Amplicon	pyrotag library	454 GS FLX Titanium	European Nucleotide Archive	https://www.ebi.ac.uk/ena/data/search?query=PRJEB12501	ERS1039918	SAMEA3732769	ERX1298255	ERR1226043	ITS	ITS2	TACACACACT	TCCTCCGCTTATTGATATGC	GCATCGATGAAGAACGCAGC	2012-03-12	31.11	-95.15	USA	0.1	88	19.0	253	Aquic Glossudalfs	Loblolly Pine, Beautyberry, Yaupon, Sweetgum, Oaks, Wax Myrtle	Cfa, Humid subtropical	OM1	C0	1	O horizon	0.0	NA	NA	4.2	NA	NA
TXC119F	TXC119	LP_TX_	Texas	TXC	Forest Soil	Whole Community DNA	PCR	Amplicon	pyrotag library	454 GS FLX Titanium	European Nucleotide Archive	https://www.ebi.ac.uk/ena/data/search?query=PRJEB12501	ERS1039919	SAMEA3732770	ERX1298256	ERR1226044	ITS	ITS2	CGAGAGATAC	TCCTCCGCTTATTGATATGC	GCATCGATGAAGAACGCAGC	2012-03-12	31.11	-95.15	USA	0.1	88	19.0	253	Aquic Glossudalfs	Loblolly Pine, Beautyberry, Yaupon, Sweetgum, Oaks, Wax Myrtle	Cfa, Humid subtropical	OM1	C0	1	O horizon	0.0	NA	NA	4.5	NA	NA
TXC120F	TXC120	LP_TX_	Texas	TXC	Forest Soil	Whole Community DNA	PCR	Amplicon	pyrotag library	454 GS FLX Titanium	European Nucleotide Archive	https://www.ebi.ac.uk/ena/data/search?query=PRJEB12501	ERS1039920	SAMEA3732771	ERX1298257	ERR1226045	ITS	ITS2	CACGCTACGT	TCCTCCGCTTATTGATATGC	GCATCGATGAAGAACGCAGC	2012-03-12	31.11	-95.15	USA	0.1	88	19.0	253	Aquic Glossudalfs	Loblolly Pine, Beautyberry, Yaupon, Sweetgum, Oaks, Wax Myrtle	Cfa, Humid subtropical	OM1	C0	1	O horizon	0.0	NA	NA	4.3	NA	NA
TXC122F	TXC122	LP_TX_	Texas	TXC	Forest Soil	Whole Community DNA	PCR	Amplicon	pyrotag library	454 GS FLX Titanium	European Nucleotide Archive	https://www.ebi.ac.uk/ena/data/search?query=PRJEB12501	ERS1039921	SAMEA3732772	ERX1298258	ERR1226046	ITS	ITS2	CACGCTACGT	TCCTCCGCTTATTGATATGC	GCATCGATGAAGAACGCAGC	2012-03-12	31.11	-95.15	USA	0.3	88	19.0	253	Aquic Glossudalfs	Loblolly Pine, Beautyberry, Yaupon, Sweetgum, Oaks, Wax Myrtle	Cfa, Humid subtropical	OM2	C0	0	A horizon	0.0	0.7	0.0	4.7	1.4	15.7
TXC124F	TXC124	LP_TX_	Texas	TXC	Forest Soil	Whole Community DNA	PCR	Amplicon	pyrotag library	454 GS FLX Titanium	European Nucleotide Archive	https://www.ebi.ac.uk/ena/data/search?query=PRJEB12501	ERS1039922	SAMEA3732773	ERX1298259	ERR1226047	ITS	ITS2	ACTGTACAGT	TCCTCCGCTTATTGATATGC	GCATCGATGAAGAACGCAGC	2012-03-12	31.11	-95.15	USA	0.1	88	19.0	253	Aquic Glossudalfs	Loblolly Pine, Beautyberry, Yaupon, Sweetgum, Oaks, Wax Myrtle	Cfa, Humid subtropical	OM2	C0	0	O horizon	0.0	NA	NA	4.2	NA	NA
TXC125F	TXC125	LP_TX_	Texas	TXC	Forest Soil	Whole Community DNA	PCR	Amplicon	pyrotag library	454 GS FLX Titanium	European Nucleotide Archive	https://www.ebi.ac.uk/ena/data/search?query=PRJEB12501	ERS1039923	SAMEA3732774	ERX1298260	ERR1226048	ITS	ITS2	CATAGTAGTG	TCCTCCGCTTATTGATATGC	GCATCGATGAAGAACGCAGC	2012-03-12	31.11	-95.15	USA	0.1	88	19.0	253	Aquic Glossudalfs	Loblolly Pine, Beautyberry, Yaupon, Sweetgum, Oaks, Wax Myrtle	Cfa, Humid subtropical	OM2	C0	0	O horizon	0.0	NA	NA	4.3	NA	NA
TXC126F	TXC126	LP_TX_	Texas	TXC	Forest Soil	Whole Community DNA	PCR	Amplicon	pyrotag library	454 GS FLX Titanium	European Nucleotide Archive	https://www.ebi.ac.uk/ena/data/search?query=PRJEB12501	ERS1039924	SAMEA3732775	ERX1298261	ERR1226049	ITS	ITS2	ACGCTCGACA	TCCTCCGCTTATTGATATGC	GCATCGATGAAGAACGCAGC	2012-03-12	31.11	-95.15	USA	0.1	88	19.0	253	Aquic Glossudalfs	Loblolly Pine, Beautyberry, Yaupon, Sweetgum, Oaks, Wax Myrtle	Cfa, Humid subtropical	OM2	C0	0	O horizon	0.0	NA	NA	5.2	NA	NA
TXC127F	TXC127	LP_TX_	Texas	TXC	Forest Soil	Whole Community DNA	PCR	Amplicon	pyrotag library	454 GS FLX Titanium	European Nucleotide Archive	https://www.ebi.ac.uk/ena/data/search?query=PRJEB12501	ERS1039925	SAMEA3732776	ERX1298262	ERR1226050	ITS	ITS2	ATAGAGTACT	TCCTCCGCTTATTGATATGC	GCATCGATGAAGAACGCAGC	2012-03-12	31.11	-95.15	USA	0.3	88	19.0	253	Aquic Glossudalfs	Loblolly Pine, Beautyberry, Yaupon, Sweetgum, Oaks, Wax Myrtle	Cfa, Humid subtropical	OM2	C0	1	A horizon	0.0	0.7	0.0	5.0	1.4	15.7
TXC128F	TXC128	LP_TX_	Texas	TXC	Forest Soil	Whole Community DNA	PCR	Amplicon	pyrotag library	454 GS FLX Titanium	European Nucleotide Archive	https://www.ebi.ac.uk/ena/data/search?query=PRJEB12501	ERS1039926	SAMEA3732777	ERX1298263	ERR1226051	ITS	ITS2	AGTACGCTAT	TCCTCCGCTTATTGATATGC	GCATCGATGAAGAACGCAGC	2012-03-12	31.11	-95.15	USA	0.3	88	19.0	253	Aquic Glossudalfs	Loblolly Pine, Beautyberry, Yaupon, Sweetgum, Oaks, Wax Myrtle	Cfa, Humid subtropical	OM2	C0	1	A horizon	0.0	0.7	0.0	5.1	1.4	15.7
TXC129F	TXC129	LP_TX_	Texas	TXC	Forest Soil	Whole Community DNA	PCR	Amplicon	pyrotag library	454 GS FLX Titanium	European Nucleotide Archive	https://www.ebi.ac.uk/ena/data/search?query=PRJEB12501	ERS1039927	SAMEA3732778	ERX1298264	ERR1226052	ITS	ITS2	CTCGCGTGTC	TCCTCCGCTTATTGATATGC	GCATCGATGAAGAACGCAGC	2012-03-12	31.11	-95.15	USA	0.3	88	19.0	253	Aquic Glossudalfs	Loblolly Pine, Beautyberry, Yaupon, Sweetgum, Oaks, Wax Myrtle	Cfa, Humid subtropical	OM2	C0	1	A horizon	0.0	0.7	0.0	5.1	1.4	15.7
TXC130F	TXC130	LP_TX_	Texas	TXC	Forest Soil	Whole Community DNA	PCR	Amplicon	pyrotag library	454 GS FLX Titanium	European Nucleotide Archive	https://www.ebi.ac.uk/ena/data/search?query=PRJEB12501	ERS1039928	SAMEA3732779	ERX1298265	ERR1226053	ITS	ITS2	TCTCTATGCG	TCCTCCGCTTATTGATATGC	GCATCGATGAAGAACGCAGC	2012-03-12	31.11	-95.15	USA	0.1	88	19.0	253	Aquic Glossudalfs	Loblolly Pine, Beautyberry, Yaupon, Sweetgum, Oaks, Wax Myrtle	Cfa, Humid subtropical	OM2	C0	1	O horizon	0.0	NA	NA	4.4	NA	NA
TXC132F	TXC132	LP_TX_	Texas	TXC	Forest Soil	Whole Community DNA	PCR	Amplicon	pyrotag library	454 GS FLX Titanium	European Nucleotide Archive	https://www.ebi.ac.uk/ena/data/search?query=PRJEB12501	ERS1039929	SAMEA3732780	ERX1298266	ERR1226054	ITS	ITS2	ACTGTACAGT	TCCTCCGCTTATTGATATGC	GCATCGATGAAGAACGCAGC	2012-03-12	31.11	-95.15	USA	0.1	88	19.0	253	Aquic Glossudalfs	Loblolly Pine, Beautyberry, Yaupon, Sweetgum, Oaks, Wax Myrtle	Cfa, Humid subtropical	OM2	C0	1	O horizon	0.0	NA	NA	4.1	NA	NA
TXC133F	TXC133	LP_TX_	Texas	TXC	Forest Soil	Whole Community DNA	PCR	Amplicon	pyrotag library	454 GS FLX Titanium	European Nucleotide Archive	https://www.ebi.ac.uk/ena/data/search?query=PRJEB12501	ERS1039930	SAMEA3732781	ERX1298267	ERR1226055	ITS	ITS2	TGATACGTCT	TCCTCCGCTTATTGATATGC	GCATCGATGAAGAACGCAGC	2012-03-12	31.11	-95.15	USA	0.3	88	19.0	253	Aquic Glossudalfs	Loblolly Pine, Beautyberry, Yaupon, Sweetgum, Oaks, Wax Myrtle	Cfa, Humid subtropical	OM3	C0	0	A horizon	0.0	0.7	0.0	4.3	1.3	16.4
TXC135F	TXC135	LP_TX_	Texas	TXC	Forest Soil	Whole Community DNA	PCR	Amplicon	pyrotag library	454 GS FLX Titanium	European Nucleotide Archive	https://www.ebi.ac.uk/ena/data/search?query=PRJEB12501	ERS1039931	SAMEA3732782	ERX1298268	ERR1226056	ITS	ITS2	TACAGATCGT	TCCTCCGCTTATTGATATGC	GCATCGATGAAGAACGCAGC	2012-03-12	31.11	-95.15	USA	0.3	88	19.0	253	Aquic Glossudalfs	Loblolly Pine, Beautyberry, Yaupon, Sweetgum, Oaks, Wax Myrtle	Cfa, Humid subtropical	OM3	C0	0	A horizon	0.0	0.7	0.0	4.6	1.3	16.4
TXC137F	TXC137	LP_TX_	Texas	TXC	Forest Soil	Whole Community DNA	PCR	Amplicon	pyrotag library	454 GS FLX Titanium	European Nucleotide Archive	https://www.ebi.ac.uk/ena/data/search?query=PRJEB12501	ERS1039932	SAMEA3732783	ERX1298269	ERR1226057	ITS	ITS2	TACAGATCGT	TCCTCCGCTTATTGATATGC	GCATCGATGAAGAACGCAGC	2012-03-12	31.11	-95.15	USA	0.1	88	19.0	253	Aquic Glossudalfs	Loblolly Pine, Beautyberry, Yaupon, Sweetgum, Oaks, Wax Myrtle	Cfa, Humid subtropical	OM3	C0	0	O horizon	0.0	NA	NA	4.8	NA	NA
TXC138F	TXC138	LP_TX_	Texas	TXC	Forest Soil	Whole Community DNA	PCR	Amplicon	pyrotag library	454 GS FLX Titanium	European Nucleotide Archive	https://www.ebi.ac.uk/ena/data/search?query=PRJEB12501	ERS1039933	SAMEA3732784	ERX1298270	ERR1226058	ITS	ITS2	TACGCTGTCT	TCCTCCGCTTATTGATATGC	GCATCGATGAAGAACGCAGC	2012-03-12	31.11	-95.15	USA	0.1	88	19.0	253	Aquic Glossudalfs	Loblolly Pine, Beautyberry, Yaupon, Sweetgum, Oaks, Wax Myrtle	Cfa, Humid subtropical	OM3	C0	0	O horizon	0.0	NA	NA	4.8	NA	NA
TXC139F	TXC139	LP_TX_	Texas	TXC	Forest Soil	Whole Community DNA	PCR	Amplicon	pyrotag library	454 GS FLX Titanium	European Nucleotide Archive	https://www.ebi.ac.uk/ena/data/search?query=PRJEB12501	ERS1039934	SAMEA3732785	ERX1298271	ERR1226059	ITS	ITS2	TCACGTACTA	TCCTCCGCTTATTGATATGC	GCATCGATGAAGAACGCAGC	2012-03-12	31.11	-95.15	USA	0.3	88	19.0	253	Aquic Glossudalfs	Loblolly Pine, Beautyberry, Yaupon, Sweetgum, Oaks, Wax Myrtle	Cfa, Humid subtropical	OM3	C0	1	A horizon	0.0	0.7	0.0	4.4	1.3	16.4
TXC140F	TXC140	LP_TX_	Texas	TXC	Forest Soil	Whole Community DNA	PCR	Amplicon	pyrotag library	454 GS FLX Titanium	European Nucleotide Archive	https://www.ebi.ac.uk/ena/data/search?query=PRJEB12501	ERS1039935	SAMEA3732786	ERX1298272	ERR1226060	ITS	ITS2	ATAGAGTACT	TCCTCCGCTTATTGATATGC	GCATCGATGAAGAACGCAGC	2012-03-12	31.11	-95.15	USA	0.3	88	19.0	253	Aquic Glossudalfs	Loblolly Pine, Beautyberry, Yaupon, Sweetgum, Oaks, Wax Myrtle	Cfa, Humid subtropical	OM3	C0	1	A horizon	0.0	0.7	0.0	4.5	1.3	16.4
TXC142F	TXC142	LP_TX_	Texas	TXC	Forest Soil	Whole Community DNA	PCR	Amplicon	pyrotag library	454 GS FLX Titanium	European Nucleotide Archive	https://www.ebi.ac.uk/ena/data/search?query=PRJEB12501	ERS1039936	SAMEA3732787	ERX1298273	ERR1226061	ITS	ITS2	CTCGCGTGTC	TCCTCCGCTTATTGATATGC	GCATCGATGAAGAACGCAGC	2012-03-12	31.11	-95.15	USA	0.1	88	19.0	253	Aquic Glossudalfs	Loblolly Pine, Beautyberry, Yaupon, Sweetgum, Oaks, Wax Myrtle	Cfa, Humid subtropical	OM3	C0	1	O horizon	0.0	NA	NA	4.1	NA	NA
TXC143F	TXC143	LP_TX_	Texas	TXC	Forest Soil	Whole Community DNA	PCR	Amplicon	pyrotag library	454 GS FLX Titanium	European Nucleotide Archive	https://www.ebi.ac.uk/ena/data/search?query=PRJEB12501	ERS1039937	SAMEA3732788	ERX1298274	ERR1226062	ITS	ITS2	AGACGCACTC	TCCTCCGCTTATTGATATGC	GCATCGATGAAGAACGCAGC	2012-03-12	31.11	-95.15	USA	0.1	88	19.0	253	Aquic Glossudalfs	Loblolly Pine, Beautyberry, Yaupon, Sweetgum, Oaks, Wax Myrtle	Cfa, Humid subtropical	OM3	C0	1	O horizon	0.0	NA	NA	4.3	NA	NA
TXC144F	TXC144	LP_TX_	Texas	TXC	Forest Soil	Whole Community DNA	PCR	Amplicon	pyrotag library	454 GS FLX Titanium	European Nucleotide Archive	https://www.ebi.ac.uk/ena/data/search?query=PRJEB12501	ERS1039938	SAMEA3732789	ERX1298275	ERR1226063	ITS	ITS2	ATACGACGTA	TCCTCCGCTTATTGATATGC	GCATCGATGAAGAACGCAGC	2012-03-12	31.11	-95.15	USA	0.1	88	19.0	253	Aquic Glossudalfs	Loblolly Pine, Beautyberry, Yaupon, Sweetgum, Oaks, Wax Myrtle	Cfa, Humid subtropical	OM3	C0	1	O horizon	0.0	NA	NA	4.3	NA	NA
TXC146F	TXC146	LP_TX_	Texas	TXC	Forest Soil	Whole Community DNA	PCR	Amplicon	pyrotag library	454 GS FLX Titanium	European Nucleotide Archive	https://www.ebi.ac.uk/ena/data/search?query=PRJEB12501	ERS1039939	SAMEA3732790	ERX1298276	ERR1226064	ITS	ITS2	AGTACGCTAT	TCCTCCGCTTATTGATATGC	GCATCGATGAAGAACGCAGC	2012-03-12	31.11	-95.15	USA	0.3	88	19.0	253	Aquic Glossudalfs	Loblolly Pine, Beautyberry, Yaupon, Sweetgum, Oaks, Wax Myrtle	Cfa, Humid subtropical	REF	REF	0	A horizon	0.0	NA	NA	0.0	NA	NA
TXC148F	TXC148	LP_TX_	Texas	TXC	Forest Soil	Whole Community DNA	PCR	Amplicon	pyrotag library	454 GS FLX Titanium	European Nucleotide Archive	https://www.ebi.ac.uk/ena/data/search?query=PRJEB12501	ERS1039940	SAMEA3732791	ERX1298277	ERR1226065	ITS	ITS2	ATCAGACACG	TCCTCCGCTTATTGATATGC	GCATCGATGAAGAACGCAGC	2012-03-12	31.11	-95.15	USA	0.1	88	19.0	253	Aquic Glossudalfs	Loblolly Pine, Beautyberry, Yaupon, Sweetgum, Oaks, Wax Myrtle	Cfa, Humid subtropical	REF	REF	0	O horizon	0.0	NA	NA	4.1	NA	NA
TXC149F	TXC149	LP_TX_	Texas	TXC	Forest Soil	Whole Community DNA	PCR	Amplicon	pyrotag library	454 GS FLX Titanium	European Nucleotide Archive	https://www.ebi.ac.uk/ena/data/search?query=PRJEB12501	ERS1039941	SAMEA3732792	ERX1298278	ERR1226066	ITS	ITS2	ACGAGTGCGT	TCCTCCGCTTATTGATATGC	GCATCGATGAAGAACGCAGC	2012-03-12	31.11	-95.15	USA	0.1	88	19.0	253	Aquic Glossudalfs	Loblolly Pine, Beautyberry, Yaupon, Sweetgum, Oaks, Wax Myrtle	Cfa, Humid subtropical	REF	REF	0	O horizon	0.0	NA	NA	4.7	NA	NA
BP241F	BP241	IDF_BC_	British Columbia	BP	Forest Soil	Whole Community DNA	PCR	Amplicon	pyrotag library	454 GS FLX Titanium	European Nucleotide Archive	https://www.ebi.ac.uk/ena/data/search?query=PRJEB12501	ERS1039942	SAMEA3732793	ERX1298279	ERR1226067	ITS	ITS2	NA	TCCTCCGCTTATTGATATGC	GCATCGATGAAGAACGCAGC	2010-06-22	50.93	-120.28	Canada	0.1	1180	2.5	300	Brunisolic Gray Luvisol	Douglas fir, Lodgepole pine	Dfb, Humid Continental warm summer	OM2	C2	0	O horizon	88.0	2.5	0.1	5.5	1.4	22.8
BP242F	BP242	IDF_BC_	British Columbia	BP	Forest Soil	Whole Community DNA	PCR	Amplicon	pyrotag library	454 GS FLX Titanium	European Nucleotide Archive	https://www.ebi.ac.uk/ena/data/search?query=PRJEB12501	ERS1039943	SAMEA3732794	ERX1298280	ERR1226068	ITS	ITS2	NA	TCCTCCGCTTATTGATATGC	GCATCGATGAAGAACGCAGC	2010-06-22	50.93	-120.28	Canada	0.1	1180	2.5	300	Brunisolic Gray Luvisol	Douglas fir, Lodgepole pine	Dfb, Humid Continental warm summer	OM2	C2	0	O horizon	90.0	2.5	0.1	5.5	1.4	22.8
BP243F	BP243	IDF_BC_	British Columbia	BP	Forest Soil	Whole Community DNA	PCR	Amplicon	pyrotag library	454 GS FLX Titanium	European Nucleotide Archive	https://www.ebi.ac.uk/ena/data/search?query=PRJEB12501	ERS1039944	SAMEA3732795	ERX1298281	ERR1226069	ITS	ITS2	NA	TCCTCCGCTTATTGATATGC	GCATCGATGAAGAACGCAGC	2010-06-22	50.93	-120.28	Canada	0.1	1180	2.5	300	Brunisolic Gray Luvisol	Douglas fir, Lodgepole pine	Dfb, Humid Continental warm summer	OM2	C2	0	O horizon	93.0	2.5	0.1	5.5	1.4	22.8
BP244F	BP244	IDF_BC_	British Columbia	BP	Forest Soil	Whole Community DNA	PCR	Amplicon	pyrotag library	454 GS FLX Titanium	European Nucleotide Archive	https://www.ebi.ac.uk/ena/data/search?query=PRJEB12501	ERS1039945	SAMEA3732796	ERX1298282	ERR1226070	ITS	ITS2	NA	TCCTCCGCTTATTGATATGC	GCATCGATGAAGAACGCAGC	2010-06-22	50.93	-120.28	Canada	0.3	1180	2.5	300	Brunisolic Gray Luvisol	Douglas fir, Lodgepole pine	Dfb, Humid Continental warm summer	OM2	C2	0	A horizon	40.0	41.8	1.5	5.8	1.4	27.7
BP245F	BP245	IDF_BC_	British Columbia	BP	Forest Soil	Whole Community DNA	PCR	Amplicon	pyrotag library	454 GS FLX Titanium	European Nucleotide Archive	https://www.ebi.ac.uk/ena/data/search?query=PRJEB12501	ERS1039946	SAMEA3732797	ERX1298283	ERR1226071	ITS	ITS2	NA	TCCTCCGCTTATTGATATGC	GCATCGATGAAGAACGCAGC	2010-06-22	50.93	-120.28	Canada	0.3	1180	2.5	300	Brunisolic Gray Luvisol	Douglas fir, Lodgepole pine	Dfb, Humid Continental warm summer	OM2	C2	0	A horizon	50.0	41.8	1.5	5.8	1.4	27.7
BP246F	BP246	IDF_BC_	British Columbia	BP	Forest Soil	Whole Community DNA	PCR	Amplicon	pyrotag library	454 GS FLX Titanium	European Nucleotide Archive	https://www.ebi.ac.uk/ena/data/search?query=PRJEB12501	ERS1039947	SAMEA3732798	ERX1298284	ERR1226072	ITS	ITS2	NA	TCCTCCGCTTATTGATATGC	GCATCGATGAAGAACGCAGC	2010-06-22	50.93	-120.28	Canada	0.3	1180	2.5	300	Brunisolic Gray Luvisol	Douglas fir, Lodgepole pine	Dfb, Humid Continental warm summer	OM2	C2	0	A horizon	39.0	41.8	1.5	5.8	1.4	27.7
BP250F	BP250	IDF_BC_	British Columbia	BP	Forest Soil	Whole Community DNA	PCR	Amplicon	pyrotag library	454 GS FLX Titanium	European Nucleotide Archive	https://www.ebi.ac.uk/ena/data/search?query=PRJEB12501	ERS1039948	SAMEA3732799	ERX1298285	ERR1226073	ITS	ITS2	NA	TCCTCCGCTTATTGATATGC	GCATCGATGAAGAACGCAGC	2010-06-22	50.93	-120.28	Canada	0.3	1180	2.5	300	Brunisolic Gray Luvisol	Douglas fir, Lodgepole pine	Dfb, Humid Continental warm summer	OM3	C2	0	A horizon	53.0	2.0	0.1	5.6	1.5	22.3
BP251F	BP251	IDF_BC_	British Columbia	BP	Forest Soil	Whole Community DNA	PCR	Amplicon	pyrotag library	454 GS FLX Titanium	European Nucleotide Archive	https://www.ebi.ac.uk/ena/data/search?query=PRJEB12501	ERS1039949	SAMEA3732800	ERX1298286	ERR1226074	ITS	ITS2	NA	TCCTCCGCTTATTGATATGC	GCATCGATGAAGAACGCAGC	2010-06-22	50.93	-120.28	Canada	0.3	1180	2.5	300	Brunisolic Gray Luvisol	Douglas fir, Lodgepole pine	Dfb, Humid Continental warm summer	OM3	C2	0	A horizon	50.0	2.0	0.1	5.6	1.5	22.3
BP252F	BP252	IDF_BC_	British Columbia	BP	Forest Soil	Whole Community DNA	PCR	Amplicon	pyrotag library	454 GS FLX Titanium	European Nucleotide Archive	https://www.ebi.ac.uk/ena/data/search?query=PRJEB12501	ERS1039950	SAMEA3732801	ERX1298287	ERR1226075	ITS	ITS2	NA	TCCTCCGCTTATTGATATGC	GCATCGATGAAGAACGCAGC	2010-06-22	50.93	-120.28	Canada	0.3	1180	2.5	300	Brunisolic Gray Luvisol	Douglas fir, Lodgepole pine	Dfb, Humid Continental warm summer	OM3	C2	0	A horizon	38.0	2.0	0.1	5.6	1.5	22.3
BP256F	BP256	IDF_BC_	British Columbia	BP	Forest Soil	Whole Community DNA	PCR	Amplicon	pyrotag library	454 GS FLX Titanium	European Nucleotide Archive	https://www.ebi.ac.uk/ena/data/search?query=PRJEB12501	ERS1039951	SAMEA3732802	ERX1298288	ERR1226076	ITS	ITS2	NA	TCCTCCGCTTATTGATATGC	GCATCGATGAAGAACGCAGC	2010-06-22	50.93	-120.28	Canada	0.3	1180	2.5	300	Brunisolic Gray Luvisol	Douglas fir, Lodgepole pine	Dfb, Humid Continental warm summer	OM3	C1	0	A horizon	45.0	2.6	0.1	5.7	1.5	23.6
BP257F	BP257	IDF_BC_	British Columbia	BP	Forest Soil	Whole Community DNA	PCR	Amplicon	pyrotag library	454 GS FLX Titanium	European Nucleotide Archive	https://www.ebi.ac.uk/ena/data/search?query=PRJEB12501	ERS1039952	SAMEA3732803	ERX1298289	ERR1226077	ITS	ITS2	NA	TCCTCCGCTTATTGATATGC	GCATCGATGAAGAACGCAGC	2010-06-22	50.93	-120.28	Canada	0.3	1180	2.5	300	Brunisolic Gray Luvisol	Douglas fir, Lodgepole pine	Dfb, Humid Continental warm summer	OM3	C1	0	A horizon	55.0	2.6	0.1	5.7	1.5	23.6
BP258F	BP258	IDF_BC_	British Columbia	BP	Forest Soil	Whole Community DNA	PCR	Amplicon	pyrotag library	454 GS FLX Titanium	European Nucleotide Archive	https://www.ebi.ac.uk/ena/data/search?query=PRJEB12501	ERS1039953	SAMEA3732804	ERX1298290	ERR1226078	ITS	ITS2	NA	TCCTCCGCTTATTGATATGC	GCATCGATGAAGAACGCAGC	2010-06-22	50.93	-120.28	Canada	0.3	1180	2.5	300	Brunisolic Gray Luvisol	Douglas fir, Lodgepole pine	Dfb, Humid Continental warm summer	OM3	C1	0	A horizon	46.0	2.6	0.1	5.7	1.5	23.6
BP259F	BP259	IDF_BC_	British Columbia	BP	Forest Soil	Whole Community DNA	PCR	Amplicon	pyrotag library	454 GS FLX Titanium	European Nucleotide Archive	https://www.ebi.ac.uk/ena/data/search?query=PRJEB12501	ERS1039954	SAMEA3732805	ERX1298291	ERR1226079	ITS	ITS2	NA	TCCTCCGCTTATTGATATGC	GCATCGATGAAGAACGCAGC	2010-06-22	50.93	-120.28	Canada	0.1	1180	2.5	300	Brunisolic Gray Luvisol	Douglas fir, Lodgepole pine	Dfb, Humid Continental warm summer	OM1	C0	0	O horizon	88.0	38.7	1.1	5.6	1.0	35.2
BP260F	BP260	IDF_BC_	British Columbia	BP	Forest Soil	Whole Community DNA	PCR	Amplicon	pyrotag library	454 GS FLX Titanium	European Nucleotide Archive	https://www.ebi.ac.uk/ena/data/search?query=PRJEB12501	ERS1039955	SAMEA3732806	ERX1298292	ERR1226080	ITS	ITS2	NA	TCCTCCGCTTATTGATATGC	GCATCGATGAAGAACGCAGC	2010-06-22	50.93	-120.28	Canada	0.1	1180	2.5	300	Brunisolic Gray Luvisol	Douglas fir, Lodgepole pine	Dfb, Humid Continental warm summer	OM1	C0	0	O horizon	88.0	38.7	1.1	5.6	1.0	35.2
BP261F	BP261	IDF_BC_	British Columbia	BP	Forest Soil	Whole Community DNA	PCR	Amplicon	pyrotag library	454 GS FLX Titanium	European Nucleotide Archive	https://www.ebi.ac.uk/ena/data/search?query=PRJEB12501	ERS1039956	SAMEA3732807	ERX1298293	ERR1226081	ITS	ITS2	NA	TCCTCCGCTTATTGATATGC	GCATCGATGAAGAACGCAGC	2010-06-22	50.93	-120.28	Canada	0.1	1180	2.5	300	Brunisolic Gray Luvisol	Douglas fir, Lodgepole pine	Dfb, Humid Continental warm summer	OM1	C0	0	O horizon	83.0	38.7	1.1	5.6	1.0	35.2
BP262F	BP262	IDF_BC_	British Columbia	BP	Forest Soil	Whole Community DNA	PCR	Amplicon	pyrotag library	454 GS FLX Titanium	European Nucleotide Archive	https://www.ebi.ac.uk/ena/data/search?query=PRJEB12501	ERS1039957	SAMEA3732808	ERX1298294	ERR1226082	ITS	ITS2	NA	TCCTCCGCTTATTGATATGC	GCATCGATGAAGAACGCAGC	2010-06-22	50.93	-120.28	Canada	0.3	1180	2.5	300	Brunisolic Gray Luvisol	Douglas fir, Lodgepole pine	Dfb, Humid Continental warm summer	OM1	C0	0	A horizon	43.0	3.0	0.1	5.4	1.0	27.1
BP263F	BP263	IDF_BC_	British Columbia	BP	Forest Soil	Whole Community DNA	PCR	Amplicon	pyrotag library	454 GS FLX Titanium	European Nucleotide Archive	https://www.ebi.ac.uk/ena/data/search?query=PRJEB12501	ERS1039958	SAMEA3732809	ERX1298295	ERR1226083	ITS	ITS2	NA	TCCTCCGCTTATTGATATGC	GCATCGATGAAGAACGCAGC	2010-06-22	50.93	-120.28	Canada	0.3	1180	2.5	300	Brunisolic Gray Luvisol	Douglas fir, Lodgepole pine	Dfb, Humid Continental warm summer	OM1	C0	0	A horizon	48.0	3.0	0.1	5.4	1.0	27.1
BP264F	BP264	IDF_BC_	British Columbia	BP	Forest Soil	Whole Community DNA	PCR	Amplicon	pyrotag library	454 GS FLX Titanium	European Nucleotide Archive	https://www.ebi.ac.uk/ena/data/search?query=PRJEB12501	ERS1039959	SAMEA3732810	ERX1298296	ERR1226084	ITS	ITS2	NA	TCCTCCGCTTATTGATATGC	GCATCGATGAAGAACGCAGC	2010-06-22	50.93	-120.28	Canada	0.3	1180	2.5	300	Brunisolic Gray Luvisol	Douglas fir, Lodgepole pine	Dfb, Humid Continental warm summer	OM1	C0	0	A horizon	47.0	3.0	0.1	5.4	1.0	27.1
BP265F	BP265	IDF_BC_	British Columbia	BP	Forest Soil	Whole Community DNA	PCR	Amplicon	pyrotag library	454 GS FLX Titanium	European Nucleotide Archive	https://www.ebi.ac.uk/ena/data/search?query=PRJEB12501	ERS1039960	SAMEA3732811	ERX1298297	ERR1226085	ITS	ITS2	NA	TCCTCCGCTTATTGATATGC	GCATCGATGAAGAACGCAGC	2010-06-22	50.93	-120.28	Canada	0.1	1180	2.5	300	Brunisolic Gray Luvisol	Douglas fir, Lodgepole pine	Dfb, Humid Continental warm summer	OM2	C0	0	O horizon	79.0	43.2	0.9	5.1	1.1	50.9
BP266F	BP266	IDF_BC_	British Columbia	BP	Forest Soil	Whole Community DNA	PCR	Amplicon	pyrotag library	454 GS FLX Titanium	European Nucleotide Archive	https://www.ebi.ac.uk/ena/data/search?query=PRJEB12501	ERS1039961	SAMEA3732812	ERX1298298	ERR1226086	ITS	ITS2	NA	TCCTCCGCTTATTGATATGC	GCATCGATGAAGAACGCAGC	2010-06-22	50.93	-120.28	Canada	0.1	1180	2.5	300	Brunisolic Gray Luvisol	Douglas fir, Lodgepole pine	Dfb, Humid Continental warm summer	OM2	C0	0	O horizon	83.0	43.2	0.9	5.1	1.1	50.9
BP267F	BP267	IDF_BC_	British Columbia	BP	Forest Soil	Whole Community DNA	PCR	Amplicon	pyrotag library	454 GS FLX Titanium	European Nucleotide Archive	https://www.ebi.ac.uk/ena/data/search?query=PRJEB12501	ERS1039962	SAMEA3732813	ERX1298299	ERR1226087	ITS	ITS2	NA	TCCTCCGCTTATTGATATGC	GCATCGATGAAGAACGCAGC	2010-06-22	50.93	-120.28	Canada	0.1	1180	2.5	300	Brunisolic Gray Luvisol	Douglas fir, Lodgepole pine	Dfb, Humid Continental warm summer	OM2	C0	0	O horizon	79.0	43.2	0.9	5.1	1.1	50.9
BP268F	BP268	IDF_BC_	British Columbia	BP	Forest Soil	Whole Community DNA	PCR	Amplicon	pyrotag library	454 GS FLX Titanium	European Nucleotide Archive	https://www.ebi.ac.uk/ena/data/search?query=PRJEB12501	ERS1039963	SAMEA3732814	ERX1298300	ERR1226088	ITS	ITS2	NA	TCCTCCGCTTATTGATATGC	GCATCGATGAAGAACGCAGC	2010-06-22	50.93	-120.28	Canada	0.3	1180	2.5	300	Brunisolic Gray Luvisol	Douglas fir, Lodgepole pine	Dfb, Humid Continental warm summer	OM2	C0	0	A horizon	44.0	2.8	0.1	5.4	1.1	27.6
BP269F	BP269	IDF_BC_	British Columbia	BP	Forest Soil	Whole Community DNA	PCR	Amplicon	pyrotag library	454 GS FLX Titanium	European Nucleotide Archive	https://www.ebi.ac.uk/ena/data/search?query=PRJEB12501	ERS1039964	SAMEA3732815	ERX1298301	ERR1226089	ITS	ITS2	NA	TCCTCCGCTTATTGATATGC	GCATCGATGAAGAACGCAGC	2010-06-22	50.93	-120.28	Canada	0.3	1180	2.5	300	Brunisolic Gray Luvisol	Douglas fir, Lodgepole pine	Dfb, Humid Continental warm summer	OM2	C0	0	A horizon	47.0	2.8	0.1	5.4	1.1	27.6
BP270F	BP270	IDF_BC_	British Columbia	BP	Forest Soil	Whole Community DNA	PCR	Amplicon	pyrotag library	454 GS FLX Titanium	European Nucleotide Archive	https://www.ebi.ac.uk/ena/data/search?query=PRJEB12501	ERS1039965	SAMEA3732816	ERX1298302	ERR1226090	ITS	ITS2	NA	TCCTCCGCTTATTGATATGC	GCATCGATGAAGAACGCAGC	2010-06-22	50.93	-120.28	Canada	0.3	1180	2.5	300	Brunisolic Gray Luvisol	Douglas fir, Lodgepole pine	Dfb, Humid Continental warm summer	OM2	C0	0	A horizon	51.0	2.8	0.1	5.4	1.1	27.6
BP271F	BP271	IDF_BC_	British Columbia	BP	Forest Soil	Whole Community DNA	PCR	Amplicon	pyrotag library	454 GS FLX Titanium	European Nucleotide Archive	https://www.ebi.ac.uk/ena/data/search?query=PRJEB12501	ERS1039966	SAMEA3732817	ERX1298303	ERR1226091	ITS	ITS2	NA	TCCTCCGCTTATTGATATGC	GCATCGATGAAGAACGCAGC	2010-06-22	50.93	-120.28	Canada	0.1	1180	2.5	300	Brunisolic Gray Luvisol	Douglas fir, Lodgepole pine	Dfb, Humid Continental warm summer	OM2	C1	0	O horizon	89.0	38.6	1.0	5.3	1.3	37.5
BP272F	BP272	IDF_BC_	British Columbia	BP	Forest Soil	Whole Community DNA	PCR	Amplicon	pyrotag library	454 GS FLX Titanium	European Nucleotide Archive	https://www.ebi.ac.uk/ena/data/search?query=PRJEB12501	ERS1039967	SAMEA3732818	ERX1298304	ERR1226092	ITS	ITS2	NA	TCCTCCGCTTATTGATATGC	GCATCGATGAAGAACGCAGC	2010-06-22	50.93	-120.28	Canada	0.1	1180	2.5	300	Brunisolic Gray Luvisol	Douglas fir, Lodgepole pine	Dfb, Humid Continental warm summer	OM2	C1	0	O horizon	83.0	38.6	1.0	5.3	1.3	37.5
BP273F	BP273	IDF_BC_	British Columbia	BP	Forest Soil	Whole Community DNA	PCR	Amplicon	pyrotag library	454 GS FLX Titanium	European Nucleotide Archive	https://www.ebi.ac.uk/ena/data/search?query=PRJEB12501	ERS1039968	SAMEA3732819	ERX1298305	ERR1226093	ITS	ITS2	NA	TCCTCCGCTTATTGATATGC	GCATCGATGAAGAACGCAGC	2010-06-22	50.93	-120.28	Canada	0.1	1180	2.5	300	Brunisolic Gray Luvisol	Douglas fir, Lodgepole pine	Dfb, Humid Continental warm summer	OM2	C1	0	O horizon	88.0	38.6	1.0	5.3	1.3	37.5
BP274F	BP274	IDF_BC_	British Columbia	BP	Forest Soil	Whole Community DNA	PCR	Amplicon	pyrotag library	454 GS FLX Titanium	European Nucleotide Archive	https://www.ebi.ac.uk/ena/data/search?query=PRJEB12501	ERS1039969	SAMEA3732820	ERX1298306	ERR1226094	ITS	ITS2	NA	TCCTCCGCTTATTGATATGC	GCATCGATGAAGAACGCAGC	2010-06-22	50.93	-120.28	Canada	0.3	1180	2.5	300	Brunisolic Gray Luvisol	Douglas fir, Lodgepole pine	Dfb, Humid Continental warm summer	OM2	C1	0	A horizon	43.0	2.7	0.1	5.5	1.3	24.2
BP275F	BP275	IDF_BC_	British Columbia	BP	Forest Soil	Whole Community DNA	PCR	Amplicon	pyrotag library	454 GS FLX Titanium	European Nucleotide Archive	https://www.ebi.ac.uk/ena/data/search?query=PRJEB12501	ERS1039970	SAMEA3732821	ERX1298307	ERR1226095	ITS	ITS2	NA	TCCTCCGCTTATTGATATGC	GCATCGATGAAGAACGCAGC	2010-06-22	50.93	-120.28	Canada	0.3	1180	2.5	300	Brunisolic Gray Luvisol	Douglas fir, Lodgepole pine	Dfb, Humid Continental warm summer	OM2	C1	0	A horizon	50.0	2.7	0.1	5.5	1.3	24.2
BP276F	BP276	IDF_BC_	British Columbia	BP	Forest Soil	Whole Community DNA	PCR	Amplicon	pyrotag library	454 GS FLX Titanium	European Nucleotide Archive	https://www.ebi.ac.uk/ena/data/search?query=PRJEB12501	ERS1039971	SAMEA3732822	ERX1298308	ERR1226096	ITS	ITS2	NA	TCCTCCGCTTATTGATATGC	GCATCGATGAAGAACGCAGC	2010-06-22	50.93	-120.28	Canada	0.3	1180	2.5	300	Brunisolic Gray Luvisol	Douglas fir, Lodgepole pine	Dfb, Humid Continental warm summer	OM2	C1	0	A horizon	44.0	2.7	0.1	5.5	1.3	24.2
BP277F	BP277	IDF_BC_	British Columbia	BP	Forest Soil	Whole Community DNA	PCR	Amplicon	pyrotag library	454 GS FLX Titanium	European Nucleotide Archive	https://www.ebi.ac.uk/ena/data/search?query=PRJEB12501	ERS1039972	SAMEA3732823	ERX1298309	ERR1226097	ITS	ITS2	NA	TCCTCCGCTTATTGATATGC	GCATCGATGAAGAACGCAGC	2010-06-22	50.93	-120.28	Canada	0.1	1180	2.5	300	Brunisolic Gray Luvisol	Douglas fir, Lodgepole pine	Dfb, Humid Continental warm summer	OM1	C1	0	O horizon	87.0	33.4	0.9	5.2	1.2	38.4
BP278F	BP278	IDF_BC_	British Columbia	BP	Forest Soil	Whole Community DNA	PCR	Amplicon	pyrotag library	454 GS FLX Titanium	European Nucleotide Archive	https://www.ebi.ac.uk/ena/data/search?query=PRJEB12501	ERS1039973	SAMEA3732824	ERX1298310	ERR1226098	ITS	ITS2	NA	TCCTCCGCTTATTGATATGC	GCATCGATGAAGAACGCAGC	2010-06-22	50.93	-120.28	Canada	0.1	1180	2.5	300	Brunisolic Gray Luvisol	Douglas fir, Lodgepole pine	Dfb, Humid Continental warm summer	OM1	C1	0	O horizon	86.0	33.4	0.9	5.2	1.2	38.4
BP279F	BP279	IDF_BC_	British Columbia	BP	Forest Soil	Whole Community DNA	PCR	Amplicon	pyrotag library	454 GS FLX Titanium	European Nucleotide Archive	https://www.ebi.ac.uk/ena/data/search?query=PRJEB12501	ERS1039974	SAMEA3732825	ERX1298311	ERR1226099	ITS	ITS2	NA	TCCTCCGCTTATTGATATGC	GCATCGATGAAGAACGCAGC	2010-06-22	50.93	-120.28	Canada	0.1	1180	2.5	300	Brunisolic Gray Luvisol	Douglas fir, Lodgepole pine	Dfb, Humid Continental warm summer	OM1	C1	0	O horizon	75.0	33.4	0.9	5.2	1.2	38.4
BP280F	BP280	IDF_BC_	British Columbia	BP	Forest Soil	Whole Community DNA	PCR	Amplicon	pyrotag library	454 GS FLX Titanium	European Nucleotide Archive	https://www.ebi.ac.uk/ena/data/search?query=PRJEB12501	ERS1039975	SAMEA3732826	ERX1298312	ERR1226100	ITS	ITS2	NA	TCCTCCGCTTATTGATATGC	GCATCGATGAAGAACGCAGC	2010-06-22	50.93	-120.28	Canada	0.3	1180	2.5	300	Brunisolic Gray Luvisol	Douglas fir, Lodgepole pine	Dfb, Humid Continental warm summer	OM1	C1	0	A horizon	53.0	2.1	0.1	5.3	1.2	22.9
BP281F	BP281	IDF_BC_	British Columbia	BP	Forest Soil	Whole Community DNA	PCR	Amplicon	pyrotag library	454 GS FLX Titanium	European Nucleotide Archive	https://www.ebi.ac.uk/ena/data/search?query=PRJEB12501	ERS1039976	SAMEA3732827	ERX1298313	ERR1226101	ITS	ITS2	NA	TCCTCCGCTTATTGATATGC	GCATCGATGAAGAACGCAGC	2010-06-22	50.93	-120.28	Canada	0.3	1180	2.5	300	Brunisolic Gray Luvisol	Douglas fir, Lodgepole pine	Dfb, Humid Continental warm summer	OM1	C1	0	A horizon	51.0	2.1	0.1	5.3	1.2	22.9
BP282F	BP282	IDF_BC_	British Columbia	BP	Forest Soil	Whole Community DNA	PCR	Amplicon	pyrotag library	454 GS FLX Titanium	European Nucleotide Archive	https://www.ebi.ac.uk/ena/data/search?query=PRJEB12501	ERS1039977	SAMEA3732828	ERX1298314	ERR1226102	ITS	ITS2	NA	TCCTCCGCTTATTGATATGC	GCATCGATGAAGAACGCAGC	2010-06-22	50.93	-120.28	Canada	0.3	1180	2.5	300	Brunisolic Gray Luvisol	Douglas fir, Lodgepole pine	Dfb, Humid Continental warm summer	OM1	C1	0	A horizon	45.0	2.1	0.1	5.3	1.2	22.9
BP286F	BP286	IDF_BC_	British Columbia	BP	Forest Soil	Whole Community DNA	PCR	Amplicon	pyrotag library	454 GS FLX Titanium	European Nucleotide Archive	https://www.ebi.ac.uk/ena/data/search?query=PRJEB12501	ERS1039978	SAMEA3732829	ERX1298315	ERR1226103	ITS	ITS2	NA	TCCTCCGCTTATTGATATGC	GCATCGATGAAGAACGCAGC	2010-06-22	50.93	-120.28	Canada	0.3	1180	2.5	300	Brunisolic Gray Luvisol	Douglas fir, Lodgepole pine	Dfb, Humid Continental warm summer	OM3	C0	0	A horizon	42.0	2.1	0.1	5.7	1.3	23.3
BP287F	BP287	IDF_BC_	British Columbia	BP	Forest Soil	Whole Community DNA	PCR	Amplicon	pyrotag library	454 GS FLX Titanium	European Nucleotide Archive	https://www.ebi.ac.uk/ena/data/search?query=PRJEB12501	ERS1039979	SAMEA3732830	ERX1298316	ERR1226104	ITS	ITS2	NA	TCCTCCGCTTATTGATATGC	GCATCGATGAAGAACGCAGC	2010-06-22	50.93	-120.28	Canada	0.3	1180	2.5	300	Brunisolic Gray Luvisol	Douglas fir, Lodgepole pine	Dfb, Humid Continental warm summer	OM3	C0	0	A horizon	48.0	2.1	0.1	5.7	1.3	23.3
BP288F	BP288	IDF_BC_	British Columbia	BP	Forest Soil	Whole Community DNA	PCR	Amplicon	pyrotag library	454 GS FLX Titanium	European Nucleotide Archive	https://www.ebi.ac.uk/ena/data/search?query=PRJEB12501	ERS1039980	SAMEA3732831	ERX1298317	ERR1226105	ITS	ITS2	NA	TCCTCCGCTTATTGATATGC	GCATCGATGAAGAACGCAGC	2010-06-22	50.93	-120.28	Canada	0.3	1180	2.5	300	Brunisolic Gray Luvisol	Douglas fir, Lodgepole pine	Dfb, Humid Continental warm summer	OM3	C0	0	A horizon	47.0	2.1	0.1	5.7	1.3	23.3
BP289F	BP289	IDF_BC_	British Columbia	BP	Forest Soil	Whole Community DNA	PCR	Amplicon	pyrotag library	454 GS FLX Titanium	European Nucleotide Archive	https://www.ebi.ac.uk/ena/data/search?query=PRJEB12501	ERS1039981	SAMEA3732832	ERX1298318	ERR1226106	ITS	ITS2	NA	TCCTCCGCTTATTGATATGC	GCATCGATGAAGAACGCAGC	2010-06-22	50.93	-120.28	Canada	0.1	1180	2.5	300	Brunisolic Gray Luvisol	Douglas fir, Lodgepole pine	Dfb, Humid Continental warm summer	OM1	C2	0	O horizon	80.0	37.6	0.9	5.1	1.4	41.4
BP290F	BP290	IDF_BC_	British Columbia	BP	Forest Soil	Whole Community DNA	PCR	Amplicon	pyrotag library	454 GS FLX Titanium	European Nucleotide Archive	https://www.ebi.ac.uk/ena/data/search?query=PRJEB12501	ERS1039982	SAMEA3732833	ERX1298319	ERR1226107	ITS	ITS2	NA	TCCTCCGCTTATTGATATGC	GCATCGATGAAGAACGCAGC	2010-06-22	50.93	-120.28	Canada	0.1	1180	2.5	300	Brunisolic Gray Luvisol	Douglas fir, Lodgepole pine	Dfb, Humid Continental warm summer	OM1	C2	0	O horizon	87.0	37.6	0.9	5.1	1.4	41.4
BP291F	BP291	IDF_BC_	British Columbia	BP	Forest Soil	Whole Community DNA	PCR	Amplicon	pyrotag library	454 GS FLX Titanium	European Nucleotide Archive	https://www.ebi.ac.uk/ena/data/search?query=PRJEB12501	ERS1039983	SAMEA3732834	ERX1298320	ERR1226108	ITS	ITS2	NA	TCCTCCGCTTATTGATATGC	GCATCGATGAAGAACGCAGC	2010-06-22	50.93	-120.28	Canada	0.1	1180	2.5	300	Brunisolic Gray Luvisol	Douglas fir, Lodgepole pine	Dfb, Humid Continental warm summer	OM1	C2	0	O horizon	80.0	37.6	0.9	5.1	1.4	41.4
BP292F	BP292	IDF_BC_	British Columbia	BP	Forest Soil	Whole Community DNA	PCR	Amplicon	pyrotag library	454 GS FLX Titanium	European Nucleotide Archive	https://www.ebi.ac.uk/ena/data/search?query=PRJEB12501	ERS1039984	SAMEA3732835	ERX1298321	ERR1226109	ITS	ITS2	NA	TCCTCCGCTTATTGATATGC	GCATCGATGAAGAACGCAGC	2010-06-22	50.93	-120.28	Canada	0.3	1180	2.5	300	Brunisolic Gray Luvisol	Douglas fir, Lodgepole pine	Dfb, Humid Continental warm summer	OM1	C2	0	A horizon	47.0	3.6	0.2	5.7	1.4	23.7
BP293F	BP293	IDF_BC_	British Columbia	BP	Forest Soil	Whole Community DNA	PCR	Amplicon	pyrotag library	454 GS FLX Titanium	European Nucleotide Archive	https://www.ebi.ac.uk/ena/data/search?query=PRJEB12501	ERS1039985	SAMEA3732836	ERX1298322	ERR1226110	ITS	ITS2	NA	TCCTCCGCTTATTGATATGC	GCATCGATGAAGAACGCAGC	2010-06-22	50.93	-120.28	Canada	0.3	1180	2.5	300	Brunisolic Gray Luvisol	Douglas fir, Lodgepole pine	Dfb, Humid Continental warm summer	OM1	C2	0	A horizon	49.0	3.6	0.2	5.7	1.4	23.7
BP294F	BP294	IDF_BC_	British Columbia	BP	Forest Soil	Whole Community DNA	PCR	Amplicon	pyrotag library	454 GS FLX Titanium	European Nucleotide Archive	https://www.ebi.ac.uk/ena/data/search?query=PRJEB12501	ERS1039986	SAMEA3732837	ERX1298323	ERR1226111	ITS	ITS2	NA	TCCTCCGCTTATTGATATGC	GCATCGATGAAGAACGCAGC	2010-06-22	50.93	-120.28	Canada	0.3	1180	2.5	300	Brunisolic Gray Luvisol	Douglas fir, Lodgepole pine	Dfb, Humid Continental warm summer	OM1	C2	0	A horizon	42.0	3.6	0.2	5.7	1.4	23.7
BP295F	BP295	IDF_BC_	British Columbia	BP	Forest Soil	Whole Community DNA	PCR	Amplicon	pyrotag library	454 GS FLX Titanium	European Nucleotide Archive	https://www.ebi.ac.uk/ena/data/search?query=PRJEB12501	ERS1039987	SAMEA3732838	ERX1298324	ERR1226112	ITS	ITS2	NA	TCCTCCGCTTATTGATATGC	GCATCGATGAAGAACGCAGC	2010-06-22	50.93	-120.28	Canada	0.1	1180	2.5	300	Brunisolic Gray Luvisol	Douglas fir, Lodgepole pine	Dfb, Humid Continental warm summer	REF	REF	0	O horizon	74.0	44.4	1.3	5.4	NA	35.5
BP296F	BP296	IDF_BC_	British Columbia	BP	Forest Soil	Whole Community DNA	PCR	Amplicon	pyrotag library	454 GS FLX Titanium	European Nucleotide Archive	https://www.ebi.ac.uk/ena/data/search?query=PRJEB12501	ERS1039988	SAMEA3732839	ERX1298325	ERR1226113	ITS	ITS2	NA	TCCTCCGCTTATTGATATGC	GCATCGATGAAGAACGCAGC	2010-06-22	50.93	-120.28	Canada	0.1	1180	2.5	300	Brunisolic Gray Luvisol	Douglas fir, Lodgepole pine	Dfb, Humid Continental warm summer	REF	REF	0	O horizon	88.0	44.4	1.3	5.4	NA	35.5
BP297F	BP297	IDF_BC_	British Columbia	BP	Forest Soil	Whole Community DNA	PCR	Amplicon	pyrotag library	454 GS FLX Titanium	European Nucleotide Archive	https://www.ebi.ac.uk/ena/data/search?query=PRJEB12501	ERS1039989	SAMEA3732840	ERX1298326	ERR1226114	ITS	ITS2	NA	TCCTCCGCTTATTGATATGC	GCATCGATGAAGAACGCAGC	2010-06-22	50.93	-120.28	Canada	0.1	1180	2.5	300	Brunisolic Gray Luvisol	Douglas fir, Lodgepole pine	Dfb, Humid Continental warm summer	REF	REF	0	O horizon	84.0	44.4	1.3	5.4	NA	35.5
BP298F	BP298	IDF_BC_	British Columbia	BP	Forest Soil	Whole Community DNA	PCR	Amplicon	pyrotag library	454 GS FLX Titanium	European Nucleotide Archive	https://www.ebi.ac.uk/ena/data/search?query=PRJEB12501	ERS1039990	SAMEA3732841	ERX1298327	ERR1226115	ITS	ITS2	NA	TCCTCCGCTTATTGATATGC	GCATCGATGAAGAACGCAGC	2010-06-22	50.93	-120.28	Canada	0.3	1180	2.5	300	Brunisolic Gray Luvisol	Douglas fir, Lodgepole pine	Dfb, Humid Continental warm summer	REF	REF	0	A horizon	46.0	2.4	0.1	5.6	NA	24.2
BP299F	BP299	IDF_BC_	British Columbia	BP	Forest Soil	Whole Community DNA	PCR	Amplicon	pyrotag library	454 GS FLX Titanium	European Nucleotide Archive	https://www.ebi.ac.uk/ena/data/search?query=PRJEB12501	ERS1039991	SAMEA3732842	ERX1298328	ERR1226116	ITS	ITS2	NA	TCCTCCGCTTATTGATATGC	GCATCGATGAAGAACGCAGC	2010-06-22	50.93	-120.28	Canada	0.3	1180	2.5	300	Brunisolic Gray Luvisol	Douglas fir, Lodgepole pine	Dfb, Humid Continental warm summer	REF	REF	0	A horizon	44.0	2.4	0.1	5.6	NA	24.2
BP300F	BP300	IDF_BC_	British Columbia	BP	Forest Soil	Whole Community DNA	PCR	Amplicon	pyrotag library	454 GS FLX Titanium	European Nucleotide Archive	https://www.ebi.ac.uk/ena/data/search?query=PRJEB12501	ERS1039992	SAMEA3732843	ERX1298329	ERR1226117	ITS	ITS2	NA	TCCTCCGCTTATTGATATGC	GCATCGATGAAGAACGCAGC	2010-06-22	50.93	-120.28	Canada	0.3	1180	2.5	300	Brunisolic Gray Luvisol	Douglas fir, Lodgepole pine	Dfb, Humid Continental warm summer	REF	REF	0	A horizon	45.0	2.4	0.1	5.6	NA	24.2
DC184F	DC184	IDF_BC_	British Columbia	DC	Forest Soil	Whole Community DNA	PCR	Amplicon	pyrotag library	454 GS FLX Titanium	European Nucleotide Archive	https://www.ebi.ac.uk/ena/data/search?query=PRJEB12501	ERS1039993	SAMEA3732844	ERX1298330	ERR1226118	ITS	ITS2	NA	TCCTCCGCTTATTGATATGC	GCATCGATGAAGAACGCAGC	2010-06-25	50.85	-120.42	Canada	0.3	1150	2.5	300	Brunisolic Gray Luvisol	Douglas fir, Subalpine fir, Lodgepole pine	Dfb, Humid Continental warm summer	OM3	C0	0	A horizon	20.0	1.7	0.1	5.9	1.5	18.4
DC185F	DC185	IDF_BC_	British Columbia	DC	Forest Soil	Whole Community DNA	PCR	Amplicon	pyrotag library	454 GS FLX Titanium	European Nucleotide Archive	https://www.ebi.ac.uk/ena/data/search?query=PRJEB12501	ERS1039994	SAMEA3732845	ERX1298331	ERR1226119	ITS	ITS2	NA	TCCTCCGCTTATTGATATGC	GCATCGATGAAGAACGCAGC	2010-06-25	50.85	-120.42	Canada	0.3	1150	2.5	300	Brunisolic Gray Luvisol	Douglas fir, Subalpine fir, Lodgepole pine	Dfb, Humid Continental warm summer	OM3	C0	0	A horizon	26.0	1.7	0.1	5.9	1.5	18.4
DC186F	DC186	IDF_BC_	British Columbia	DC	Forest Soil	Whole Community DNA	PCR	Amplicon	pyrotag library	454 GS FLX Titanium	European Nucleotide Archive	https://www.ebi.ac.uk/ena/data/search?query=PRJEB12501	ERS1039995	SAMEA3732846	ERX1298332	ERR1226120	ITS	ITS2	NA	TCCTCCGCTTATTGATATGC	GCATCGATGAAGAACGCAGC	2010-06-25	50.85	-120.42	Canada	0.3	1150	2.5	300	Brunisolic Gray Luvisol	Douglas fir, Subalpine fir, Lodgepole pine	Dfb, Humid Continental warm summer	OM3	C0	0	A horizon	20.0	1.7	0.1	5.9	1.5	18.4
DC187F	DC187	IDF_BC_	British Columbia	DC	Forest Soil	Whole Community DNA	PCR	Amplicon	pyrotag library	454 GS FLX Titanium	European Nucleotide Archive	https://www.ebi.ac.uk/ena/data/search?query=PRJEB12501	ERS1039996	SAMEA3732847	ERX1298333	ERR1226121	ITS	ITS2	NA	TCCTCCGCTTATTGATATGC	GCATCGATGAAGAACGCAGC	2010-06-25	50.85	-120.42	Canada	0.1	1150	2.5	300	Brunisolic Gray Luvisol	Douglas fir, Subalpine fir, Lodgepole pine	Dfb, Humid Continental warm summer	OM2	C0	0	O horizon	58.0	32.9	1.3	6.0	1.3	25.1
DC188F	DC188	IDF_BC_	British Columbia	DC	Forest Soil	Whole Community DNA	PCR	Amplicon	pyrotag library	454 GS FLX Titanium	European Nucleotide Archive	https://www.ebi.ac.uk/ena/data/search?query=PRJEB12501	ERS1039997	SAMEA3732848	ERX1298334	ERR1226122	ITS	ITS2	NA	TCCTCCGCTTATTGATATGC	GCATCGATGAAGAACGCAGC	2010-06-25	50.85	-120.42	Canada	0.1	1150	2.5	300	Brunisolic Gray Luvisol	Douglas fir, Subalpine fir, Lodgepole pine	Dfb, Humid Continental warm summer	OM2	C0	0	O horizon	63.0	32.9	1.3	6.0	1.3	25.1
DC189F	DC189	IDF_BC_	British Columbia	DC	Forest Soil	Whole Community DNA	PCR	Amplicon	pyrotag library	454 GS FLX Titanium	European Nucleotide Archive	https://www.ebi.ac.uk/ena/data/search?query=PRJEB12501	ERS1039998	SAMEA3732849	ERX1298335	ERR1226123	ITS	ITS2	NA	TCCTCCGCTTATTGATATGC	GCATCGATGAAGAACGCAGC	2010-06-25	50.85	-120.42	Canada	0.1	1150	2.5	300	Brunisolic Gray Luvisol	Douglas fir, Subalpine fir, Lodgepole pine	Dfb, Humid Continental warm summer	OM2	C0	0	O horizon	56.0	32.9	1.3	6.0	1.3	25.1
DC190F	DC190	IDF_BC_	British Columbia	DC	Forest Soil	Whole Community DNA	PCR	Amplicon	pyrotag library	454 GS FLX Titanium	European Nucleotide Archive	https://www.ebi.ac.uk/ena/data/search?query=PRJEB12501	ERS1039999	SAMEA3732850	ERX1298336	ERR1226124	ITS	ITS2	NA	TCCTCCGCTTATTGATATGC	GCATCGATGAAGAACGCAGC	2010-06-25	50.85	-120.42	Canada	0.3	1150	2.5	300	Brunisolic Gray Luvisol	Douglas fir, Subalpine fir, Lodgepole pine	Dfb, Humid Continental warm summer	OM2	C0	0	A horizon	21.0	3.3	0.2	5.6	1.3	20.5
DC191F	DC191	IDF_BC_	British Columbia	DC	Forest Soil	Whole Community DNA	PCR	Amplicon	pyrotag library	454 GS FLX Titanium	European Nucleotide Archive	https://www.ebi.ac.uk/ena/data/search?query=PRJEB12501	ERS1040000	SAMEA3732851	ERX1298337	ERR1226125	ITS	ITS2	NA	TCCTCCGCTTATTGATATGC	GCATCGATGAAGAACGCAGC	2010-06-25	50.85	-120.42	Canada	0.3	1150	2.5	300	Brunisolic Gray Luvisol	Douglas fir, Subalpine fir, Lodgepole pine	Dfb, Humid Continental warm summer	OM2	C0	0	A horizon	23.0	3.3	0.2	5.6	1.3	20.5
DC192F	DC192	IDF_BC_	British Columbia	DC	Forest Soil	Whole Community DNA	PCR	Amplicon	pyrotag library	454 GS FLX Titanium	European Nucleotide Archive	https://www.ebi.ac.uk/ena/data/search?query=PRJEB12501	ERS1040001	SAMEA3732852	ERX1298338	ERR1226126	ITS	ITS2	NA	TCCTCCGCTTATTGATATGC	GCATCGATGAAGAACGCAGC	2010-06-25	50.85	-120.42	Canada	0.3	1150	2.5	300	Brunisolic Gray Luvisol	Douglas fir, Subalpine fir, Lodgepole pine	Dfb, Humid Continental warm summer	OM2	C0	0	A horizon	25.0	3.3	0.2	5.6	1.3	20.5
DC193F	DC193	IDF_BC_	British Columbia	DC	Forest Soil	Whole Community DNA	PCR	Amplicon	pyrotag library	454 GS FLX Titanium	European Nucleotide Archive	https://www.ebi.ac.uk/ena/data/search?query=PRJEB12501	ERS1040002	SAMEA3732853	ERX1298339	ERR1226127	ITS	ITS2	NA	TCCTCCGCTTATTGATATGC	GCATCGATGAAGAACGCAGC	2010-06-25	50.85	-120.42	Canada	0.1	1150	2.5	300	Brunisolic Gray Luvisol	Douglas fir, Subalpine fir, Lodgepole pine	Dfb, Humid Continental warm summer	OM2	C1	0	O horizon	61.0	32.9	1.2	5.8	1.5	28.4
DC194F	DC194	IDF_BC_	British Columbia	DC	Forest Soil	Whole Community DNA	PCR	Amplicon	pyrotag library	454 GS FLX Titanium	European Nucleotide Archive	https://www.ebi.ac.uk/ena/data/search?query=PRJEB12501	ERS1040003	SAMEA3732854	ERX1298340	ERR1226128	ITS	ITS2	NA	TCCTCCGCTTATTGATATGC	GCATCGATGAAGAACGCAGC	2010-06-25	50.85	-120.42	Canada	0.1	1150	2.5	300	Brunisolic Gray Luvisol	Douglas fir, Subalpine fir, Lodgepole pine	Dfb, Humid Continental warm summer	OM2	C1	0	O horizon	63.0	32.9	1.2	5.8	1.5	28.4
DC195F	DC195	IDF_BC_	British Columbia	DC	Forest Soil	Whole Community DNA	PCR	Amplicon	pyrotag library	454 GS FLX Titanium	European Nucleotide Archive	https://www.ebi.ac.uk/ena/data/search?query=PRJEB12501	ERS1040004	SAMEA3732855	ERX1298341	ERR1226129	ITS	ITS2	NA	TCCTCCGCTTATTGATATGC	GCATCGATGAAGAACGCAGC	2010-06-25	50.85	-120.42	Canada	0.1	1150	2.5	300	Brunisolic Gray Luvisol	Douglas fir, Subalpine fir, Lodgepole pine	Dfb, Humid Continental warm summer	OM2	C1	0	O horizon	57.0	32.9	1.2	5.8	1.5	28.4
DC196F	DC196	IDF_BC_	British Columbia	DC	Forest Soil	Whole Community DNA	PCR	Amplicon	pyrotag library	454 GS FLX Titanium	European Nucleotide Archive	https://www.ebi.ac.uk/ena/data/search?query=PRJEB12501	ERS1040005	SAMEA3732856	ERX1298342	ERR1226130	ITS	ITS2	NA	TCCTCCGCTTATTGATATGC	GCATCGATGAAGAACGCAGC	2010-06-25	50.85	-120.42	Canada	0.3	1150	2.5	300	Brunisolic Gray Luvisol	Douglas fir, Subalpine fir, Lodgepole pine	Dfb, Humid Continental warm summer	OM2	C1	0	A horizon	29.0	2.1	0.1	5.6	1.5	20.5
DC197F	DC197	IDF_BC_	British Columbia	DC	Forest Soil	Whole Community DNA	PCR	Amplicon	pyrotag library	454 GS FLX Titanium	European Nucleotide Archive	https://www.ebi.ac.uk/ena/data/search?query=PRJEB12501	ERS1040006	SAMEA3732857	ERX1298343	ERR1226131	ITS	ITS2	NA	TCCTCCGCTTATTGATATGC	GCATCGATGAAGAACGCAGC	2010-06-25	50.85	-120.42	Canada	0.3	1150	2.5	300	Brunisolic Gray Luvisol	Douglas fir, Subalpine fir, Lodgepole pine	Dfb, Humid Continental warm summer	OM2	C1	0	A horizon	27.0	2.1	0.1	5.6	1.5	20.5
DC198F	DC198	IDF_BC_	British Columbia	DC	Forest Soil	Whole Community DNA	PCR	Amplicon	pyrotag library	454 GS FLX Titanium	European Nucleotide Archive	https://www.ebi.ac.uk/ena/data/search?query=PRJEB12501	ERS1040007	SAMEA3732858	ERX1298344	ERR1226132	ITS	ITS2	NA	TCCTCCGCTTATTGATATGC	GCATCGATGAAGAACGCAGC	2010-06-25	50.85	-120.42	Canada	0.3	1150	2.5	300	Brunisolic Gray Luvisol	Douglas fir, Subalpine fir, Lodgepole pine	Dfb, Humid Continental warm summer	OM2	C1	0	A horizon	21.0	2.1	0.1	5.6	1.5	20.5
DC199F	DC199	IDF_BC_	British Columbia	DC	Forest Soil	Whole Community DNA	PCR	Amplicon	pyrotag library	454 GS FLX Titanium	European Nucleotide Archive	https://www.ebi.ac.uk/ena/data/search?query=PRJEB12501	ERS1040008	SAMEA3732859	ERX1298345	ERR1226133	ITS	ITS2	NA	TCCTCCGCTTATTGATATGC	GCATCGATGAAGAACGCAGC	2010-06-25	50.85	-120.42	Canada	0.1	1150	2.5	300	Brunisolic Gray Luvisol	Douglas fir, Subalpine fir, Lodgepole pine	Dfb, Humid Continental warm summer	OM2	C2	0	O horizon	58.0	30.1	1.0	5.8	1.5	29.3
DC200F	DC200	IDF_BC_	British Columbia	DC	Forest Soil	Whole Community DNA	PCR	Amplicon	pyrotag library	454 GS FLX Titanium	European Nucleotide Archive	https://www.ebi.ac.uk/ena/data/search?query=PRJEB12501	ERS1040009	SAMEA3732860	ERX1298346	ERR1226134	ITS	ITS2	NA	TCCTCCGCTTATTGATATGC	GCATCGATGAAGAACGCAGC	2010-06-25	50.85	-120.42	Canada	0.1	1150	2.5	300	Brunisolic Gray Luvisol	Douglas fir, Subalpine fir, Lodgepole pine	Dfb, Humid Continental warm summer	OM2	C2	0	O horizon	53.0	30.1	1.0	5.8	1.5	29.3
DC201F	DC201	IDF_BC_	British Columbia	DC	Forest Soil	Whole Community DNA	PCR	Amplicon	pyrotag library	454 GS FLX Titanium	European Nucleotide Archive	https://www.ebi.ac.uk/ena/data/search?query=PRJEB12501	ERS1040010	SAMEA3732861	ERX1298347	ERR1226135	ITS	ITS2	NA	TCCTCCGCTTATTGATATGC	GCATCGATGAAGAACGCAGC	2010-06-25	50.85	-120.42	Canada	0.1	1150	2.5	300	Brunisolic Gray Luvisol	Douglas fir, Subalpine fir, Lodgepole pine	Dfb, Humid Continental warm summer	OM2	C2	0	O horizon	59.0	30.1	1.0	5.8	1.5	29.3
DC202F	DC202	IDF_BC_	British Columbia	DC	Forest Soil	Whole Community DNA	PCR	Amplicon	pyrotag library	454 GS FLX Titanium	European Nucleotide Archive	https://www.ebi.ac.uk/ena/data/search?query=PRJEB12501	ERS1040011	SAMEA3732862	ERX1298348	ERR1226136	ITS	ITS2	NA	TCCTCCGCTTATTGATATGC	GCATCGATGAAGAACGCAGC	2010-06-25	50.85	-120.42	Canada	0.3	1150	2.5	300	Brunisolic Gray Luvisol	Douglas fir, Subalpine fir, Lodgepole pine	Dfb, Humid Continental warm summer	OM2	C2	0	A horizon	24.0	2.4	0.1	5.8	1.5	18.5
DC203F	DC203	IDF_BC_	British Columbia	DC	Forest Soil	Whole Community DNA	PCR	Amplicon	pyrotag library	454 GS FLX Titanium	European Nucleotide Archive	https://www.ebi.ac.uk/ena/data/search?query=PRJEB12501	ERS1040012	SAMEA3732863	ERX1298349	ERR1226137	ITS	ITS2	NA	TCCTCCGCTTATTGATATGC	GCATCGATGAAGAACGCAGC	2010-06-25	50.85	-120.42	Canada	0.3	1150	2.5	300	Brunisolic Gray Luvisol	Douglas fir, Subalpine fir, Lodgepole pine	Dfb, Humid Continental warm summer	OM2	C2	0	A horizon	33.0	2.4	0.1	5.8	1.5	18.5
DC204F	DC204	IDF_BC_	British Columbia	DC	Forest Soil	Whole Community DNA	PCR	Amplicon	pyrotag library	454 GS FLX Titanium	European Nucleotide Archive	https://www.ebi.ac.uk/ena/data/search?query=PRJEB12501	ERS1040013	SAMEA3732864	ERX1298350	ERR1226138	ITS	ITS2	NA	TCCTCCGCTTATTGATATGC	GCATCGATGAAGAACGCAGC	2010-06-25	50.85	-120.42	Canada	0.3	1150	2.5	300	Brunisolic Gray Luvisol	Douglas fir, Subalpine fir, Lodgepole pine	Dfb, Humid Continental warm summer	OM2	C2	0	A horizon	30.0	2.4	0.1	5.8	1.5	18.5
DC205F	DC205	IDF_BC_	British Columbia	DC	Forest Soil	Whole Community DNA	PCR	Amplicon	pyrotag library	454 GS FLX Titanium	European Nucleotide Archive	https://www.ebi.ac.uk/ena/data/search?query=PRJEB12501	ERS1040014	SAMEA3732865	ERX1298351	ERR1226139	ITS	ITS2	NA	TCCTCCGCTTATTGATATGC	GCATCGATGAAGAACGCAGC	2010-06-25	50.85	-120.42	Canada	0.1	1150	2.5	300	Brunisolic Gray Luvisol	Douglas fir, Subalpine fir, Lodgepole pine	Dfb, Humid Continental warm summer	OM1	C0	0	O horizon	60.0	38.7	1.3	5.6	1.4	29.5
DC206F	DC206	IDF_BC_	British Columbia	DC	Forest Soil	Whole Community DNA	PCR	Amplicon	pyrotag library	454 GS FLX Titanium	European Nucleotide Archive	https://www.ebi.ac.uk/ena/data/search?query=PRJEB12501	ERS1040015	SAMEA3732866	ERX1298352	ERR1226140	ITS	ITS2	NA	TCCTCCGCTTATTGATATGC	GCATCGATGAAGAACGCAGC	2010-06-25	50.85	-120.42	Canada	0.1	1150	2.5	300	Brunisolic Gray Luvisol	Douglas fir, Subalpine fir, Lodgepole pine	Dfb, Humid Continental warm summer	OM1	C0	0	O horizon	60.0	38.7	1.3	5.6	1.4	29.5
DC207F	DC207	IDF_BC_	British Columbia	DC	Forest Soil	Whole Community DNA	PCR	Amplicon	pyrotag library	454 GS FLX Titanium	European Nucleotide Archive	https://www.ebi.ac.uk/ena/data/search?query=PRJEB12501	ERS1040016	SAMEA3732867	ERX1298353	ERR1226141	ITS	ITS2	NA	TCCTCCGCTTATTGATATGC	GCATCGATGAAGAACGCAGC	2010-06-25	50.85	-120.42	Canada	0.1	1150	2.5	300	Brunisolic Gray Luvisol	Douglas fir, Subalpine fir, Lodgepole pine	Dfb, Humid Continental warm summer	OM1	C0	0	O horizon	58.0	38.7	1.3	5.6	1.4	29.5
DC208F	DC208	IDF_BC_	British Columbia	DC	Forest Soil	Whole Community DNA	PCR	Amplicon	pyrotag library	454 GS FLX Titanium	European Nucleotide Archive	https://www.ebi.ac.uk/ena/data/search?query=PRJEB12501	ERS1040017	SAMEA3732868	ERX1298354	ERR1226142	ITS	ITS2	NA	TCCTCCGCTTATTGATATGC	GCATCGATGAAGAACGCAGC	2010-06-25	50.85	-120.42	Canada	0.3	1150	2.5	300	Brunisolic Gray Luvisol	Douglas fir, Subalpine fir, Lodgepole pine	Dfb, Humid Continental warm summer	OM1	C0	0	A horizon	28.0	2.3	0.1	5.2	1.4	20.5
DC209F	DC209	IDF_BC_	British Columbia	DC	Forest Soil	Whole Community DNA	PCR	Amplicon	pyrotag library	454 GS FLX Titanium	European Nucleotide Archive	https://www.ebi.ac.uk/ena/data/search?query=PRJEB12501	ERS1040018	SAMEA3732869	ERX1298355	ERR1226143	ITS	ITS2	NA	TCCTCCGCTTATTGATATGC	GCATCGATGAAGAACGCAGC	2010-06-25	50.85	-120.42	Canada	0.3	1150	2.5	300	Brunisolic Gray Luvisol	Douglas fir, Subalpine fir, Lodgepole pine	Dfb, Humid Continental warm summer	OM1	C0	0	A horizon	26.0	2.3	0.1	5.2	1.4	20.5
DC210F	DC210	IDF_BC_	British Columbia	DC	Forest Soil	Whole Community DNA	PCR	Amplicon	pyrotag library	454 GS FLX Titanium	European Nucleotide Archive	https://www.ebi.ac.uk/ena/data/search?query=PRJEB12501	ERS1040019	SAMEA3732870	ERX1298356	ERR1226144	ITS	ITS2	NA	TCCTCCGCTTATTGATATGC	GCATCGATGAAGAACGCAGC	2010-06-25	50.85	-120.42	Canada	0.3	1150	2.5	300	Brunisolic Gray Luvisol	Douglas fir, Subalpine fir, Lodgepole pine	Dfb, Humid Continental warm summer	OM1	C0	0	A horizon	24.0	2.3	0.1	5.2	1.4	20.5
DC214F	DC214	IDF_BC_	British Columbia	DC	Forest Soil	Whole Community DNA	PCR	Amplicon	pyrotag library	454 GS FLX Titanium	European Nucleotide Archive	https://www.ebi.ac.uk/ena/data/search?query=PRJEB12501	ERS1040020	SAMEA3732871	ERX1298357	ERR1226145	ITS	ITS2	NA	TCCTCCGCTTATTGATATGC	GCATCGATGAAGAACGCAGC	2010-06-25	50.85	-120.42	Canada	0.3	1150	2.5	300	Brunisolic Gray Luvisol	Douglas fir, Subalpine fir, Lodgepole pine	Dfb, Humid Continental warm summer	OM3	C1	0	A horizon	17.0	1.8	0.1	5.7	1.7	19.7
DC215F	DC215	IDF_BC_	British Columbia	DC	Forest Soil	Whole Community DNA	PCR	Amplicon	pyrotag library	454 GS FLX Titanium	European Nucleotide Archive	https://www.ebi.ac.uk/ena/data/search?query=PRJEB12501	ERS1040021	SAMEA3732872	ERX1298358	ERR1226146	ITS	ITS2	NA	TCCTCCGCTTATTGATATGC	GCATCGATGAAGAACGCAGC	2010-06-25	50.85	-120.42	Canada	0.3	1150	2.5	300	Brunisolic Gray Luvisol	Douglas fir, Subalpine fir, Lodgepole pine	Dfb, Humid Continental warm summer	OM3	C1	0	A horizon	18.0	1.8	0.1	5.7	1.7	19.7
DC216F	DC216	IDF_BC_	British Columbia	DC	Forest Soil	Whole Community DNA	PCR	Amplicon	pyrotag library	454 GS FLX Titanium	European Nucleotide Archive	https://www.ebi.ac.uk/ena/data/search?query=PRJEB12501	ERS1040022	SAMEA3732873	ERX1298359	ERR1226147	ITS	ITS2	NA	TCCTCCGCTTATTGATATGC	GCATCGATGAAGAACGCAGC	2010-06-25	50.85	-120.42	Canada	0.3	1150	2.5	300	Brunisolic Gray Luvisol	Douglas fir, Subalpine fir, Lodgepole pine	Dfb, Humid Continental warm summer	OM3	C1	0	A horizon	25.0	1.8	0.1	5.7	1.7	19.7
DC220F	DC220	IDF_BC_	British Columbia	DC	Forest Soil	Whole Community DNA	PCR	Amplicon	pyrotag library	454 GS FLX Titanium	European Nucleotide Archive	https://www.ebi.ac.uk/ena/data/search?query=PRJEB12501	ERS1040023	SAMEA3732874	ERX1298360	ERR1226148	ITS	ITS2	NA	TCCTCCGCTTATTGATATGC	GCATCGATGAAGAACGCAGC	2010-06-25	50.85	-120.42	Canada	0.3	1150	2.5	300	Brunisolic Gray Luvisol	Douglas fir, Subalpine fir, Lodgepole pine	Dfb, Humid Continental warm summer	OM3	C2	0	A horizon	25.0	2.0	0.1	5.8	1.7	18.0
DC221F	DC221	IDF_BC_	British Columbia	DC	Forest Soil	Whole Community DNA	PCR	Amplicon	pyrotag library	454 GS FLX Titanium	European Nucleotide Archive	https://www.ebi.ac.uk/ena/data/search?query=PRJEB12501	ERS1040024	SAMEA3732875	ERX1298361	ERR1226149	ITS	ITS2	NA	TCCTCCGCTTATTGATATGC	GCATCGATGAAGAACGCAGC	2010-06-25	50.85	-120.42	Canada	0.3	1150	2.5	300	Brunisolic Gray Luvisol	Douglas fir, Subalpine fir, Lodgepole pine	Dfb, Humid Continental warm summer	OM3	C2	0	A horizon	25.0	2.0	0.1	5.8	1.7	18.0
DC222F	DC222	IDF_BC_	British Columbia	DC	Forest Soil	Whole Community DNA	PCR	Amplicon	pyrotag library	454 GS FLX Titanium	European Nucleotide Archive	https://www.ebi.ac.uk/ena/data/search?query=PRJEB12501	ERS1040025	SAMEA3732876	ERX1298362	ERR1226150	ITS	ITS2	NA	TCCTCCGCTTATTGATATGC	GCATCGATGAAGAACGCAGC	2010-06-25	50.85	-120.42	Canada	0.3	1150	2.5	300	Brunisolic Gray Luvisol	Douglas fir, Subalpine fir, Lodgepole pine	Dfb, Humid Continental warm summer	OM3	C2	0	A horizon	21.0	2.0	0.1	5.8	1.7	18.0
DC223F	DC223	IDF_BC_	British Columbia	DC	Forest Soil	Whole Community DNA	PCR	Amplicon	pyrotag library	454 GS FLX Titanium	European Nucleotide Archive	https://www.ebi.ac.uk/ena/data/search?query=PRJEB12501	ERS1040026	SAMEA3732877	ERX1298363	ERR1226151	ITS	ITS2	NA	TCCTCCGCTTATTGATATGC	GCATCGATGAAGAACGCAGC	2010-06-25	50.85	-120.42	Canada	0.1	1150	2.5	300	Brunisolic Gray Luvisol	Douglas fir, Subalpine fir, Lodgepole pine	Dfb, Humid Continental warm summer	OM1	C2	0	O horizon	63.0	38.8	1.0	5.5	1.5	39.2
DC224F	DC224	IDF_BC_	British Columbia	DC	Forest Soil	Whole Community DNA	PCR	Amplicon	pyrotag library	454 GS FLX Titanium	European Nucleotide Archive	https://www.ebi.ac.uk/ena/data/search?query=PRJEB12501	ERS1040027	SAMEA3732878	ERX1298364	ERR1226152	ITS	ITS2	NA	TCCTCCGCTTATTGATATGC	GCATCGATGAAGAACGCAGC	2010-06-25	50.85	-120.42	Canada	0.1	1150	2.5	300	Brunisolic Gray Luvisol	Douglas fir, Subalpine fir, Lodgepole pine	Dfb, Humid Continental warm summer	OM1	C2	0	O horizon	57.0	38.8	1.0	5.5	1.5	39.2
DC225F	DC225	IDF_BC_	British Columbia	DC	Forest Soil	Whole Community DNA	PCR	Amplicon	pyrotag library	454 GS FLX Titanium	European Nucleotide Archive	https://www.ebi.ac.uk/ena/data/search?query=PRJEB12501	ERS1040028	SAMEA3732879	ERX1298365	ERR1226153	ITS	ITS2	NA	TCCTCCGCTTATTGATATGC	GCATCGATGAAGAACGCAGC	2010-06-25	50.85	-120.42	Canada	0.1	1150	2.5	300	Brunisolic Gray Luvisol	Douglas fir, Subalpine fir, Lodgepole pine	Dfb, Humid Continental warm summer	OM1	C2	0	O horizon	63.0	38.8	1.0	5.5	1.5	39.2
DC226F	DC226	IDF_BC_	British Columbia	DC	Forest Soil	Whole Community DNA	PCR	Amplicon	pyrotag library	454 GS FLX Titanium	European Nucleotide Archive	https://www.ebi.ac.uk/ena/data/search?query=PRJEB12501	ERS1040029	SAMEA3732880	ERX1298366	ERR1226154	ITS	ITS2	NA	TCCTCCGCTTATTGATATGC	GCATCGATGAAGAACGCAGC	2010-06-25	50.85	-120.42	Canada	0.3	1150	2.5	300	Brunisolic Gray Luvisol	Douglas fir, Subalpine fir, Lodgepole pine	Dfb, Humid Continental warm summer	OM1	C2	0	A horizon	27.0	2.6	0.1	5.5	1.5	21.4
DC227F	DC227	IDF_BC_	British Columbia	DC	Forest Soil	Whole Community DNA	PCR	Amplicon	pyrotag library	454 GS FLX Titanium	European Nucleotide Archive	https://www.ebi.ac.uk/ena/data/search?query=PRJEB12501	ERS1040030	SAMEA3732881	ERX1298367	ERR1226155	ITS	ITS2	NA	TCCTCCGCTTATTGATATGC	GCATCGATGAAGAACGCAGC	2010-06-25	50.85	-120.42	Canada	0.3	1150	2.5	300	Brunisolic Gray Luvisol	Douglas fir, Subalpine fir, Lodgepole pine	Dfb, Humid Continental warm summer	OM1	C2	0	A horizon	26.0	2.6	0.1	5.5	1.5	21.4
DC228F	DC228	IDF_BC_	British Columbia	DC	Forest Soil	Whole Community DNA	PCR	Amplicon	pyrotag library	454 GS FLX Titanium	European Nucleotide Archive	https://www.ebi.ac.uk/ena/data/search?query=PRJEB12501	ERS1040031	SAMEA3732882	ERX1298368	ERR1226156	ITS	ITS2	NA	TCCTCCGCTTATTGATATGC	GCATCGATGAAGAACGCAGC	2010-06-25	50.85	-120.42	Canada	0.3	1150	2.5	300	Brunisolic Gray Luvisol	Douglas fir, Subalpine fir, Lodgepole pine	Dfb, Humid Continental warm summer	OM1	C2	0	A horizon	27.0	2.6	0.1	5.5	1.5	21.4
DC229F	DC229	IDF_BC_	British Columbia	DC	Forest Soil	Whole Community DNA	PCR	Amplicon	pyrotag library	454 GS FLX Titanium	European Nucleotide Archive	https://www.ebi.ac.uk/ena/data/search?query=PRJEB12501	ERS1040032	SAMEA3732883	ERX1298369	ERR1226157	ITS	ITS2	NA	TCCTCCGCTTATTGATATGC	GCATCGATGAAGAACGCAGC	2010-06-25	50.85	-120.42	Canada	0.1	1150	2.5	300	Brunisolic Gray Luvisol	Douglas fir, Subalpine fir, Lodgepole pine	Dfb, Humid Continental warm summer	OM1	C1	0	O horizon	60.0	36.7	1.1	5.9	1.4	33.3
DC230F	DC230	IDF_BC_	British Columbia	DC	Forest Soil	Whole Community DNA	PCR	Amplicon	pyrotag library	454 GS FLX Titanium	European Nucleotide Archive	https://www.ebi.ac.uk/ena/data/search?query=PRJEB12501	ERS1040033	SAMEA3732884	ERX1298370	ERR1226158	ITS	ITS2	NA	TCCTCCGCTTATTGATATGC	GCATCGATGAAGAACGCAGC	2010-06-25	50.85	-120.42	Canada	0.1	1150	2.5	300	Brunisolic Gray Luvisol	Douglas fir, Subalpine fir, Lodgepole pine	Dfb, Humid Continental warm summer	OM1	C1	0	O horizon	60.0	36.7	1.1	5.9	1.4	33.3
DC231F	DC231	IDF_BC_	British Columbia	DC	Forest Soil	Whole Community DNA	PCR	Amplicon	pyrotag library	454 GS FLX Titanium	European Nucleotide Archive	https://www.ebi.ac.uk/ena/data/search?query=PRJEB12501	ERS1040034	SAMEA3732885	ERX1298371	ERR1226159	ITS	ITS2	NA	TCCTCCGCTTATTGATATGC	GCATCGATGAAGAACGCAGC	2010-06-25	50.85	-120.42	Canada	0.1	1150	2.5	300	Brunisolic Gray Luvisol	Douglas fir, Subalpine fir, Lodgepole pine	Dfb, Humid Continental warm summer	OM1	C1	0	O horizon	52.0	36.7	1.1	5.9	1.4	33.3
DC232F	DC232	IDF_BC_	British Columbia	DC	Forest Soil	Whole Community DNA	PCR	Amplicon	pyrotag library	454 GS FLX Titanium	European Nucleotide Archive	https://www.ebi.ac.uk/ena/data/search?query=PRJEB12501	ERS1040035	SAMEA3732886	ERX1298372	ERR1226160	ITS	ITS2	NA	TCCTCCGCTTATTGATATGC	GCATCGATGAAGAACGCAGC	2010-06-25	50.85	-120.42	Canada	0.3	1150	2.5	300	Brunisolic Gray Luvisol	Douglas fir, Subalpine fir, Lodgepole pine	Dfb, Humid Continental warm summer	OM1	C1	0	A horizon	29.0	2.8	0.1	5.9	1.4	19.9
DC233F	DC233	IDF_BC_	British Columbia	DC	Forest Soil	Whole Community DNA	PCR	Amplicon	pyrotag library	454 GS FLX Titanium	European Nucleotide Archive	https://www.ebi.ac.uk/ena/data/search?query=PRJEB12501	ERS1040036	SAMEA3732887	ERX1298373	ERR1226161	ITS	ITS2	NA	TCCTCCGCTTATTGATATGC	GCATCGATGAAGAACGCAGC	2010-06-25	50.85	-120.42	Canada	0.3	1150	2.5	300	Brunisolic Gray Luvisol	Douglas fir, Subalpine fir, Lodgepole pine	Dfb, Humid Continental warm summer	OM1	C1	0	A horizon	29.0	2.8	0.1	5.9	1.4	19.9
DC234F	DC234	IDF_BC_	British Columbia	DC	Forest Soil	Whole Community DNA	PCR	Amplicon	pyrotag library	454 GS FLX Titanium	European Nucleotide Archive	https://www.ebi.ac.uk/ena/data/search?query=PRJEB12501	ERS1040037	SAMEA3732888	ERX1298374	ERR1226162	ITS	ITS2	NA	TCCTCCGCTTATTGATATGC	GCATCGATGAAGAACGCAGC	2010-06-25	50.85	-120.42	Canada	0.3	1150	2.5	300	Brunisolic Gray Luvisol	Douglas fir, Subalpine fir, Lodgepole pine	Dfb, Humid Continental warm summer	OM1	C1	0	A horizon	29.0	2.8	0.1	5.9	1.4	19.9
DC235F	DC235	IDF_BC_	British Columbia	DC	Forest Soil	Whole Community DNA	PCR	Amplicon	pyrotag library	454 GS FLX Titanium	European Nucleotide Archive	https://www.ebi.ac.uk/ena/data/search?query=PRJEB12501	ERS1040038	SAMEA3732889	ERX1298375	ERR1226163	ITS	ITS2	NA	TCCTCCGCTTATTGATATGC	GCATCGATGAAGAACGCAGC	2010-06-25	50.85	-120.42	Canada	0.1	1150	2.5	300	Brunisolic Gray Luvisol	Douglas fir, Subalpine fir, Lodgepole pine	Dfb, Humid Continental warm summer	REF	REF	0	O horizon	63.0	44.3	1.1	5.0	NA	41.4
DC236F	DC236	IDF_BC_	British Columbia	DC	Forest Soil	Whole Community DNA	PCR	Amplicon	pyrotag library	454 GS FLX Titanium	European Nucleotide Archive	https://www.ebi.ac.uk/ena/data/search?query=PRJEB12501	ERS1040039	SAMEA3732890	ERX1298376	ERR1226164	ITS	ITS2	NA	TCCTCCGCTTATTGATATGC	GCATCGATGAAGAACGCAGC	2010-06-25	50.85	-120.42	Canada	0.1	1150	2.5	300	Brunisolic Gray Luvisol	Douglas fir, Subalpine fir, Lodgepole pine	Dfb, Humid Continental warm summer	REF	REF	0	O horizon	64.0	44.3	1.1	5.0	NA	41.4
DC237F	DC237	IDF_BC_	British Columbia	DC	Forest Soil	Whole Community DNA	PCR	Amplicon	pyrotag library	454 GS FLX Titanium	European Nucleotide Archive	https://www.ebi.ac.uk/ena/data/search?query=PRJEB12501	ERS1040040	SAMEA3732891	ERX1298377	ERR1226165	ITS	ITS2	NA	TCCTCCGCTTATTGATATGC	GCATCGATGAAGAACGCAGC	2010-06-25	50.85	-120.42	Canada	0.1	1150	2.5	300	Brunisolic Gray Luvisol	Douglas fir, Subalpine fir, Lodgepole pine	Dfb, Humid Continental warm summer	REF	REF	0	O horizon	60.0	44.3	1.1	5.0	NA	41.4
DC238F	DC238	IDF_BC_	British Columbia	DC	Forest Soil	Whole Community DNA	PCR	Amplicon	pyrotag library	454 GS FLX Titanium	European Nucleotide Archive	https://www.ebi.ac.uk/ena/data/search?query=PRJEB12501	ERS1040041	SAMEA3732892	ERX1298378	ERR1226166	ITS	ITS2	NA	TCCTCCGCTTATTGATATGC	GCATCGATGAAGAACGCAGC	2010-06-25	50.85	-120.42	Canada	0.3	1150	2.5	300	Brunisolic Gray Luvisol	Douglas fir, Subalpine fir, Lodgepole pine	Dfb, Humid Continental warm summer	REF	REF	0	A horizon	25.0	2.3	0.1	5.2	NA	23.4
DC239F	DC239	IDF_BC_	British Columbia	DC	Forest Soil	Whole Community DNA	PCR	Amplicon	pyrotag library	454 GS FLX Titanium	European Nucleotide Archive	https://www.ebi.ac.uk/ena/data/search?query=PRJEB12501	ERS1040042	SAMEA3732893	ERX1298379	ERR1226167	ITS	ITS2	NA	TCCTCCGCTTATTGATATGC	GCATCGATGAAGAACGCAGC	2010-06-25	50.85	-120.42	Canada	0.3	1150	2.5	300	Brunisolic Gray Luvisol	Douglas fir, Subalpine fir, Lodgepole pine	Dfb, Humid Continental warm summer	REF	REF	0	A horizon	27.0	2.3	0.1	5.2	NA	23.4
DC240F	DC240	IDF_BC_	British Columbia	DC	Forest Soil	Whole Community DNA	PCR	Amplicon	pyrotag library	454 GS FLX Titanium	European Nucleotide Archive	https://www.ebi.ac.uk/ena/data/search?query=PRJEB12501	ERS1040043	SAMEA3732894	ERX1298380	ERR1226168	ITS	ITS2	NA	TCCTCCGCTTATTGATATGC	GCATCGATGAAGAACGCAGC	2010-06-25	50.85	-120.42	Canada	0.3	1150	2.5	300	Brunisolic Gray Luvisol	Douglas fir, Subalpine fir, Lodgepole pine	Dfb, Humid Continental warm summer	REF	REF	0	A horizon	34.0	2.3	0.1	5.2	NA	23.4
LL001F	LL001	SBS_BC_	British Columbia	LL	Forest Soil	Whole Community DNA	PCR	Amplicon	pyrotag library	454 GS FLX Titanium	European Nucleotide Archive	https://www.ebi.ac.uk/ena/data/search?query=PRJEB12501	ERS1040044	SAMEA3732895	ERX1298381	ERR1226169	ITS	ITS2	NA	TCCTCCGCTTATTGATATGC	GCATCGATGAAGAACGCAGC	2008-07-09	54.35	-122.61	Canada	0.1	780	3.3	146-193	Orthic Humo-Ferric Podzol, Gleyed Eluviated Dystric Brunisol	Subalpine fir, Douglas fir, Interior Spruce	Dfc, Boreal cool summer	OM1	C0	0	O horizon	42.0	24.3	0.8	4.7	1.4	30.8
LL002F	LL002	SBS_BC_	British Columbia	LL	Forest Soil	Whole Community DNA	PCR	Amplicon	pyrotag library	454 GS FLX Titanium	European Nucleotide Archive	https://www.ebi.ac.uk/ena/data/search?query=PRJEB12501	ERS1040045	SAMEA3732896	ERX1298382	ERR1226170	ITS	ITS2	NA	TCCTCCGCTTATTGATATGC	GCATCGATGAAGAACGCAGC	2008-07-09	54.35	-122.61	Canada	0.1	780	3.3	146-193	Orthic Humo-Ferric Podzol, Gleyed Eluviated Dystric Brunisol	Subalpine fir, Douglas fir, Interior Spruce	Dfc, Boreal cool summer	OM1	C0	0	O horizon	48.0	24.3	0.8	4.7	1.4	30.8
LL003F	LL003	SBS_BC_	British Columbia	LL	Forest Soil	Whole Community DNA	PCR	Amplicon	pyrotag library	454 GS FLX Titanium	European Nucleotide Archive	https://www.ebi.ac.uk/ena/data/search?query=PRJEB12501	ERS1040046	SAMEA3732897	ERX1298383	ERR1226171	ITS	ITS2	NA	TCCTCCGCTTATTGATATGC	GCATCGATGAAGAACGCAGC	2008-07-09	54.35	-122.61	Canada	0.1	780	3.3	146-193	Orthic Humo-Ferric Podzol, Gleyed Eluviated Dystric Brunisol	Subalpine fir, Douglas fir, Interior Spruce	Dfc, Boreal cool summer	OM1	C0	0	O horizon	40.0	24.3	0.8	4.7	1.4	30.8
LL004F	LL004	SBS_BC_	British Columbia	LL	Forest Soil	Whole Community DNA	PCR	Amplicon	pyrotag library	454 GS FLX Titanium	European Nucleotide Archive	https://www.ebi.ac.uk/ena/data/search?query=PRJEB12501	ERS1040047	SAMEA3732898	ERX1298384	ERR1226172	ITS	ITS2	NA	TCCTCCGCTTATTGATATGC	GCATCGATGAAGAACGCAGC	2008-07-09	54.35	-122.61	Canada	0.3	780	3.3	146-193	Orthic Humo-Ferric Podzol, Gleyed Eluviated Dystric Brunisol	Subalpine fir, Douglas fir, Interior Spruce	Dfc, Boreal cool summer	OM1	C0	0	A horizon	22.0	1.6	0.1	4.7	1.4	22.1
LL005F	LL005	SBS_BC_	British Columbia	LL	Forest Soil	Whole Community DNA	PCR	Amplicon	pyrotag library	454 GS FLX Titanium	European Nucleotide Archive	https://www.ebi.ac.uk/ena/data/search?query=PRJEB12501	ERS1040048	SAMEA3732899	ERX1298385	ERR1226173	ITS	ITS2	NA	TCCTCCGCTTATTGATATGC	GCATCGATGAAGAACGCAGC	2008-07-09	54.35	-122.61	Canada	0.3	780	3.3	146-193	Orthic Humo-Ferric Podzol, Gleyed Eluviated Dystric Brunisol	Subalpine fir, Douglas fir, Interior Spruce	Dfc, Boreal cool summer	OM1	C0	0	A horizon	20.0	1.6	0.1	4.7	1.4	22.1
LL006F	LL006	SBS_BC_	British Columbia	LL	Forest Soil	Whole Community DNA	PCR	Amplicon	pyrotag library	454 GS FLX Titanium	European Nucleotide Archive	https://www.ebi.ac.uk/ena/data/search?query=PRJEB12501	ERS1040049	SAMEA3732900	ERX1298386	ERR1226174	ITS	ITS2	NA	TCCTCCGCTTATTGATATGC	GCATCGATGAAGAACGCAGC	2008-07-09	54.35	-122.61	Canada	0.3	780	3.3	146-193	Orthic Humo-Ferric Podzol, Gleyed Eluviated Dystric Brunisol	Subalpine fir, Douglas fir, Interior Spruce	Dfc, Boreal cool summer	OM1	C0	0	A horizon	21.0	1.6	0.1	4.7	1.4	22.1
LL007F	LL007	SBS_BC_	British Columbia	LL	Forest Soil	Whole Community DNA	PCR	Amplicon	pyrotag library	454 GS FLX Titanium	European Nucleotide Archive	https://www.ebi.ac.uk/ena/data/search?query=PRJEB12501	ERS1040050	SAMEA3732901	ERX1298387	ERR1226175	ITS	ITS2	NA	TCCTCCGCTTATTGATATGC	GCATCGATGAAGAACGCAGC	2008-07-09	54.35	-122.61	Canada	0.1	780	3.3	146-193	Orthic Humo-Ferric Podzol, Gleyed Eluviated Dystric Brunisol	Subalpine fir, Douglas fir, Interior Spruce	Dfc, Boreal cool summer	OM2	C0	0	O horizon	48.0	27.6	0.8	4.5	1.6	33.7
LL008F	LL008	SBS_BC_	British Columbia	LL	Forest Soil	Whole Community DNA	PCR	Amplicon	pyrotag library	454 GS FLX Titanium	European Nucleotide Archive	https://www.ebi.ac.uk/ena/data/search?query=PRJEB12501	ERS1040051	SAMEA3732902	ERX1298388	ERR1226176	ITS	ITS2	NA	TCCTCCGCTTATTGATATGC	GCATCGATGAAGAACGCAGC	2008-07-09	54.35	-122.61	Canada	0.1	780	3.3	146-193	Orthic Humo-Ferric Podzol, Gleyed Eluviated Dystric Brunisol	Subalpine fir, Douglas fir, Interior Spruce	Dfc, Boreal cool summer	OM2	C0	0	O horizon	49.0	27.6	0.8	4.5	1.6	33.7
LL009F	LL009	SBS_BC_	British Columbia	LL	Forest Soil	Whole Community DNA	PCR	Amplicon	pyrotag library	454 GS FLX Titanium	European Nucleotide Archive	https://www.ebi.ac.uk/ena/data/search?query=PRJEB12501	ERS1040052	SAMEA3732903	ERX1298389	ERR1226177	ITS	ITS2	NA	TCCTCCGCTTATTGATATGC	GCATCGATGAAGAACGCAGC	2008-07-09	54.35	-122.61	Canada	0.1	780	3.3	146-193	Orthic Humo-Ferric Podzol, Gleyed Eluviated Dystric Brunisol	Subalpine fir, Douglas fir, Interior Spruce	Dfc, Boreal cool summer	OM2	C0	0	O horizon	40.0	27.6	0.8	4.5	1.6	33.7
LL010F	LL010	SBS_BC_	British Columbia	LL	Forest Soil	Whole Community DNA	PCR	Amplicon	pyrotag library	454 GS FLX Titanium	European Nucleotide Archive	https://www.ebi.ac.uk/ena/data/search?query=PRJEB12501	ERS1040053	SAMEA3732904	ERX1298390	ERR1226178	ITS	ITS2	NA	TCCTCCGCTTATTGATATGC	GCATCGATGAAGAACGCAGC	2008-07-09	54.35	-122.61	Canada	0.3	780	3.3	146-193	Orthic Humo-Ferric Podzol, Gleyed Eluviated Dystric Brunisol	Subalpine fir, Douglas fir, Interior Spruce	Dfc, Boreal cool summer	OM2	C0	0	A horizon	17.0	1.2	0.1	4.8	1.6	19.3
LL011F	LL011	SBS_BC_	British Columbia	LL	Forest Soil	Whole Community DNA	PCR	Amplicon	pyrotag library	454 GS FLX Titanium	European Nucleotide Archive	https://www.ebi.ac.uk/ena/data/search?query=PRJEB12501	ERS1040054	SAMEA3732905	ERX1298391	ERR1226179	ITS	ITS2	NA	TCCTCCGCTTATTGATATGC	GCATCGATGAAGAACGCAGC	2008-07-09	54.35	-122.61	Canada	0.3	780	3.3	146-193	Orthic Humo-Ferric Podzol, Gleyed Eluviated Dystric Brunisol	Subalpine fir, Douglas fir, Interior Spruce	Dfc, Boreal cool summer	OM2	C0	0	A horizon	19.0	1.2	0.1	4.8	1.6	19.3
LL012F	LL012	SBS_BC_	British Columbia	LL	Forest Soil	Whole Community DNA	PCR	Amplicon	pyrotag library	454 GS FLX Titanium	European Nucleotide Archive	https://www.ebi.ac.uk/ena/data/search?query=PRJEB12501	ERS1040055	SAMEA3732906	ERX1298392	ERR1226180	ITS	ITS2	NA	TCCTCCGCTTATTGATATGC	GCATCGATGAAGAACGCAGC	2008-07-09	54.35	-122.61	Canada	0.3	780	3.3	146-193	Orthic Humo-Ferric Podzol, Gleyed Eluviated Dystric Brunisol	Subalpine fir, Douglas fir, Interior Spruce	Dfc, Boreal cool summer	OM2	C0	0	A horizon	16.0	1.2	0.1	4.8	1.6	19.3
LL016F	LL016	SBS_BC_	British Columbia	LL	Forest Soil	Whole Community DNA	PCR	Amplicon	pyrotag library	454 GS FLX Titanium	European Nucleotide Archive	https://www.ebi.ac.uk/ena/data/search?query=PRJEB12501	ERS1040056	SAMEA3732907	ERX1298393	ERR1226181	ITS	ITS2	NA	TCCTCCGCTTATTGATATGC	GCATCGATGAAGAACGCAGC	2008-07-09	54.35	-122.61	Canada	0.3	780	3.3	146-193	Orthic Humo-Ferric Podzol, Gleyed Eluviated Dystric Brunisol	Subalpine fir, Douglas fir, Interior Spruce	Dfc, Boreal cool summer	OM3	C0	0	A horizon	23.0	1.7	0.1	5.1	1.3	20.9
LL017F	LL017	SBS_BC_	British Columbia	LL	Forest Soil	Whole Community DNA	PCR	Amplicon	pyrotag library	454 GS FLX Titanium	European Nucleotide Archive	https://www.ebi.ac.uk/ena/data/search?query=PRJEB12501	ERS1040057	SAMEA3732908	ERX1298394	ERR1226182	ITS	ITS2	NA	TCCTCCGCTTATTGATATGC	GCATCGATGAAGAACGCAGC	2008-07-09	54.35	-122.61	Canada	0.3	780	3.3	146-193	Orthic Humo-Ferric Podzol, Gleyed Eluviated Dystric Brunisol	Subalpine fir, Douglas fir, Interior Spruce	Dfc, Boreal cool summer	OM3	C0	0	A horizon	20.0	1.7	0.1	5.1	1.3	20.9
LL018F	LL018	SBS_BC_	British Columbia	LL	Forest Soil	Whole Community DNA	PCR	Amplicon	pyrotag library	454 GS FLX Titanium	European Nucleotide Archive	https://www.ebi.ac.uk/ena/data/search?query=PRJEB12501	ERS1040058	SAMEA3732909	ERX1298395	ERR1226183	ITS	ITS2	NA	TCCTCCGCTTATTGATATGC	GCATCGATGAAGAACGCAGC	2008-07-09	54.35	-122.61	Canada	0.3	780	3.3	146-193	Orthic Humo-Ferric Podzol, Gleyed Eluviated Dystric Brunisol	Subalpine fir, Douglas fir, Interior Spruce	Dfc, Boreal cool summer	OM3	C0	0	A horizon	21.0	1.7	0.1	5.1	1.3	20.9
LL019F	LL019	SBS_BC_	British Columbia	LL	Forest Soil	Whole Community DNA	PCR	Amplicon	pyrotag library	454 GS FLX Titanium	European Nucleotide Archive	https://www.ebi.ac.uk/ena/data/search?query=PRJEB12501	ERS1040059	SAMEA3732910	ERX1298396	ERR1226184	ITS	ITS2	NA	TCCTCCGCTTATTGATATGC	GCATCGATGAAGAACGCAGC	2008-07-09	54.35	-122.61	Canada	0.1	780	3.3	146-193	Orthic Humo-Ferric Podzol, Gleyed Eluviated Dystric Brunisol	Subalpine fir, Douglas fir, Interior Spruce	Dfc, Boreal cool summer	OM1	C1	0	O horizon	61.0	36.0	0.8	4.6	1.5	43.8
LL020F	LL020	SBS_BC_	British Columbia	LL	Forest Soil	Whole Community DNA	PCR	Amplicon	pyrotag library	454 GS FLX Titanium	European Nucleotide Archive	https://www.ebi.ac.uk/ena/data/search?query=PRJEB12501	ERS1040060	SAMEA3732911	ERX1298397	ERR1226185	ITS	ITS2	NA	TCCTCCGCTTATTGATATGC	GCATCGATGAAGAACGCAGC	2008-07-09	54.35	-122.61	Canada	0.1	780	3.3	146-193	Orthic Humo-Ferric Podzol, Gleyed Eluviated Dystric Brunisol	Subalpine fir, Douglas fir, Interior Spruce	Dfc, Boreal cool summer	OM1	C1	0	O horizon	46.0	36.0	0.8	4.6	1.5	43.8
LL021F	LL021	SBS_BC_	British Columbia	LL	Forest Soil	Whole Community DNA	PCR	Amplicon	pyrotag library	454 GS FLX Titanium	European Nucleotide Archive	https://www.ebi.ac.uk/ena/data/search?query=PRJEB12501	ERS1040061	SAMEA3732912	ERX1298398	ERR1226186	ITS	ITS2	NA	TCCTCCGCTTATTGATATGC	GCATCGATGAAGAACGCAGC	2008-07-09	54.35	-122.61	Canada	0.1	780	3.3	146-193	Orthic Humo-Ferric Podzol, Gleyed Eluviated Dystric Brunisol	Subalpine fir, Douglas fir, Interior Spruce	Dfc, Boreal cool summer	OM1	C1	0	O horizon	44.0	36.0	0.8	4.6	1.5	43.8
LL022F	LL022	SBS_BC_	British Columbia	LL	Forest Soil	Whole Community DNA	PCR	Amplicon	pyrotag library	454 GS FLX Titanium	European Nucleotide Archive	https://www.ebi.ac.uk/ena/data/search?query=PRJEB12501	ERS1040062	SAMEA3732913	ERX1298399	ERR1226187	ITS	ITS2	NA	TCCTCCGCTTATTGATATGC	GCATCGATGAAGAACGCAGC	2008-07-09	54.35	-122.61	Canada	0.3	780	3.3	146-193	Orthic Humo-Ferric Podzol, Gleyed Eluviated Dystric Brunisol	Subalpine fir, Douglas fir, Interior Spruce	Dfc, Boreal cool summer	OM1	C1	0	A horizon	17.0	1.2	0.1	4.6	1.5	23.4
LL023F	LL023	SBS_BC_	British Columbia	LL	Forest Soil	Whole Community DNA	PCR	Amplicon	pyrotag library	454 GS FLX Titanium	European Nucleotide Archive	https://www.ebi.ac.uk/ena/data/search?query=PRJEB12501	ERS1040063	SAMEA3732914	ERX1298400	ERR1226188	ITS	ITS2	NA	TCCTCCGCTTATTGATATGC	GCATCGATGAAGAACGCAGC	2008-07-09	54.35	-122.61	Canada	0.3	780	3.3	146-193	Orthic Humo-Ferric Podzol, Gleyed Eluviated Dystric Brunisol	Subalpine fir, Douglas fir, Interior Spruce	Dfc, Boreal cool summer	OM1	C1	0	A horizon	20.0	1.2	0.1	4.6	1.5	23.4
LL024F	LL024	SBS_BC_	British Columbia	LL	Forest Soil	Whole Community DNA	PCR	Amplicon	pyrotag library	454 GS FLX Titanium	European Nucleotide Archive	https://www.ebi.ac.uk/ena/data/search?query=PRJEB12501	ERS1040064	SAMEA3732915	ERX1298401	ERR1226189	ITS	ITS2	NA	TCCTCCGCTTATTGATATGC	GCATCGATGAAGAACGCAGC	2008-07-09	54.35	-122.61	Canada	0.3	780	3.3	146-193	Orthic Humo-Ferric Podzol, Gleyed Eluviated Dystric Brunisol	Subalpine fir, Douglas fir, Interior Spruce	Dfc, Boreal cool summer	OM1	C1	0	A horizon	19.0	1.2	0.1	4.6	1.5	23.4
LL025F	LL025	SBS_BC_	British Columbia	LL	Forest Soil	Whole Community DNA	PCR	Amplicon	pyrotag library	454 GS FLX Titanium	European Nucleotide Archive	https://www.ebi.ac.uk/ena/data/search?query=PRJEB12501	ERS1040065	SAMEA3732916	ERX1298402	ERR1226190	ITS	ITS2	NA	TCCTCCGCTTATTGATATGC	GCATCGATGAAGAACGCAGC	2008-07-09	54.35	-122.61	Canada	0.1	780	3.3	146-193	Orthic Humo-Ferric Podzol, Gleyed Eluviated Dystric Brunisol	Subalpine fir, Douglas fir, Interior Spruce	Dfc, Boreal cool summer	OM2	C1	0	O horizon	52.0	27.5	0.8	4.5	1.6	34.8
LL026F	LL026	SBS_BC_	British Columbia	LL	Forest Soil	Whole Community DNA	PCR	Amplicon	pyrotag library	454 GS FLX Titanium	European Nucleotide Archive	https://www.ebi.ac.uk/ena/data/search?query=PRJEB12501	ERS1040066	SAMEA3732917	ERX1298403	ERR1226191	ITS	ITS2	NA	TCCTCCGCTTATTGATATGC	GCATCGATGAAGAACGCAGC	2008-07-09	54.35	-122.61	Canada	0.1	780	3.3	146-193	Orthic Humo-Ferric Podzol, Gleyed Eluviated Dystric Brunisol	Subalpine fir, Douglas fir, Interior Spruce	Dfc, Boreal cool summer	OM2	C1	0	O horizon	55.0	27.5	0.8	4.5	1.6	34.8
LL027F	LL027	SBS_BC_	British Columbia	LL	Forest Soil	Whole Community DNA	PCR	Amplicon	pyrotag library	454 GS FLX Titanium	European Nucleotide Archive	https://www.ebi.ac.uk/ena/data/search?query=PRJEB12501	ERS1040067	SAMEA3732918	ERX1298404	ERR1226192	ITS	ITS2	NA	TCCTCCGCTTATTGATATGC	GCATCGATGAAGAACGCAGC	2008-07-09	54.35	-122.61	Canada	0.1	780	3.3	146-193	Orthic Humo-Ferric Podzol, Gleyed Eluviated Dystric Brunisol	Subalpine fir, Douglas fir, Interior Spruce	Dfc, Boreal cool summer	OM2	C1	0	O horizon	60.0	27.5	0.8	4.5	1.6	34.8
LL028F	LL028	SBS_BC_	British Columbia	LL	Forest Soil	Whole Community DNA	PCR	Amplicon	pyrotag library	454 GS FLX Titanium	European Nucleotide Archive	https://www.ebi.ac.uk/ena/data/search?query=PRJEB12501	ERS1040068	SAMEA3732919	ERX1298405	ERR1226193	ITS	ITS2	NA	TCCTCCGCTTATTGATATGC	GCATCGATGAAGAACGCAGC	2008-07-09	54.35	-122.61	Canada	0.3	780	3.3	146-193	Orthic Humo-Ferric Podzol, Gleyed Eluviated Dystric Brunisol	Subalpine fir, Douglas fir, Interior Spruce	Dfc, Boreal cool summer	OM2	C1	0	A horizon	20.0	1.5	0.1	4.9	1.6	21.1
LL029F	LL029	SBS_BC_	British Columbia	LL	Forest Soil	Whole Community DNA	PCR	Amplicon	pyrotag library	454 GS FLX Titanium	European Nucleotide Archive	https://www.ebi.ac.uk/ena/data/search?query=PRJEB12501	ERS1040069	SAMEA3732920	ERX1298406	ERR1226194	ITS	ITS2	NA	TCCTCCGCTTATTGATATGC	GCATCGATGAAGAACGCAGC	2008-07-09	54.35	-122.61	Canada	0.3	780	3.3	146-193	Orthic Humo-Ferric Podzol, Gleyed Eluviated Dystric Brunisol	Subalpine fir, Douglas fir, Interior Spruce	Dfc, Boreal cool summer	OM2	C1	0	A horizon	17.0	1.5	0.1	4.9	1.6	21.1
LL030F	LL030	SBS_BC_	British Columbia	LL	Forest Soil	Whole Community DNA	PCR	Amplicon	pyrotag library	454 GS FLX Titanium	European Nucleotide Archive	https://www.ebi.ac.uk/ena/data/search?query=PRJEB12501	ERS1040070	SAMEA3732921	ERX1298407	ERR1226195	ITS	ITS2	NA	TCCTCCGCTTATTGATATGC	GCATCGATGAAGAACGCAGC	2008-07-09	54.35	-122.61	Canada	0.3	780	3.3	146-193	Orthic Humo-Ferric Podzol, Gleyed Eluviated Dystric Brunisol	Subalpine fir, Douglas fir, Interior Spruce	Dfc, Boreal cool summer	OM2	C1	0	A horizon	19.0	1.5	0.1	4.9	1.6	21.1
LL034F	LL034	SBS_BC_	British Columbia	LL	Forest Soil	Whole Community DNA	PCR	Amplicon	pyrotag library	454 GS FLX Titanium	European Nucleotide Archive	https://www.ebi.ac.uk/ena/data/search?query=PRJEB12501	ERS1040071	SAMEA3732922	ERX1298408	ERR1226196	ITS	ITS2	NA	TCCTCCGCTTATTGATATGC	GCATCGATGAAGAACGCAGC	2008-07-09	54.35	-122.61	Canada	0.3	780	3.3	146-193	Orthic Humo-Ferric Podzol, Gleyed Eluviated Dystric Brunisol	Subalpine fir, Douglas fir, Interior Spruce	Dfc, Boreal cool summer	OM3	C1	0	A horizon	22.0	1.3	0.1	4.8	1.4	21.8
LL035F	LL035	SBS_BC_	British Columbia	LL	Forest Soil	Whole Community DNA	PCR	Amplicon	pyrotag library	454 GS FLX Titanium	European Nucleotide Archive	https://www.ebi.ac.uk/ena/data/search?query=PRJEB12501	ERS1040072	SAMEA3732923	ERX1298409	ERR1226197	ITS	ITS2	NA	TCCTCCGCTTATTGATATGC	GCATCGATGAAGAACGCAGC	2008-07-09	54.35	-122.61	Canada	0.3	780	3.3	146-193	Orthic Humo-Ferric Podzol, Gleyed Eluviated Dystric Brunisol	Subalpine fir, Douglas fir, Interior Spruce	Dfc, Boreal cool summer	OM3	C1	0	A horizon	20.0	1.3	0.1	4.8	1.4	21.8
LL036F	LL036	SBS_BC_	British Columbia	LL	Forest Soil	Whole Community DNA	PCR	Amplicon	pyrotag library	454 GS FLX Titanium	European Nucleotide Archive	https://www.ebi.ac.uk/ena/data/search?query=PRJEB12501	ERS1040073	SAMEA3732924	ERX1298410	ERR1226198	ITS	ITS2	NA	TCCTCCGCTTATTGATATGC	GCATCGATGAAGAACGCAGC	2008-07-09	54.35	-122.61	Canada	0.3	780	3.3	146-193	Orthic Humo-Ferric Podzol, Gleyed Eluviated Dystric Brunisol	Subalpine fir, Douglas fir, Interior Spruce	Dfc, Boreal cool summer	OM3	C1	0	A horizon	24.0	1.3	0.1	4.8	1.4	21.8
LL037F	LL037	SBS_BC_	British Columbia	LL	Forest Soil	Whole Community DNA	PCR	Amplicon	pyrotag library	454 GS FLX Titanium	European Nucleotide Archive	https://www.ebi.ac.uk/ena/data/search?query=PRJEB12501	ERS1040074	SAMEA3732925	ERX1298411	ERR1226199	ITS	ITS2	NA	TCCTCCGCTTATTGATATGC	GCATCGATGAAGAACGCAGC	2008-07-09	54.35	-122.61	Canada	0.1	780	3.3	146-193	Orthic Humo-Ferric Podzol, Gleyed Eluviated Dystric Brunisol	Subalpine fir, Douglas fir, Interior Spruce	Dfc, Boreal cool summer	OM1	C2	0	O horizon	60.0	31.3	1.0	4.7	1.4	31.9
LL038F	LL038	SBS_BC_	British Columbia	LL	Forest Soil	Whole Community DNA	PCR	Amplicon	pyrotag library	454 GS FLX Titanium	European Nucleotide Archive	https://www.ebi.ac.uk/ena/data/search?query=PRJEB12501	ERS1040075	SAMEA3732926	ERX1298412	ERR1226200	ITS	ITS2	NA	TCCTCCGCTTATTGATATGC	GCATCGATGAAGAACGCAGC	2008-07-09	54.35	-122.61	Canada	0.1	780	3.3	146-193	Orthic Humo-Ferric Podzol, Gleyed Eluviated Dystric Brunisol	Subalpine fir, Douglas fir, Interior Spruce	Dfc, Boreal cool summer	OM1	C2	0	O horizon	47.0	31.3	1.0	4.7	1.4	31.9
LL039F	LL039	SBS_BC_	British Columbia	LL	Forest Soil	Whole Community DNA	PCR	Amplicon	pyrotag library	454 GS FLX Titanium	European Nucleotide Archive	https://www.ebi.ac.uk/ena/data/search?query=PRJEB12501	ERS1040076	SAMEA3732927	ERX1298413	ERR1226201	ITS	ITS2	NA	TCCTCCGCTTATTGATATGC	GCATCGATGAAGAACGCAGC	2008-07-09	54.35	-122.61	Canada	0.1	780	3.3	146-193	Orthic Humo-Ferric Podzol, Gleyed Eluviated Dystric Brunisol	Subalpine fir, Douglas fir, Interior Spruce	Dfc, Boreal cool summer	OM1	C2	0	O horizon	66.0	31.3	1.0	4.7	1.4	31.9
LL040F	LL040	SBS_BC_	British Columbia	LL	Forest Soil	Whole Community DNA	PCR	Amplicon	pyrotag library	454 GS FLX Titanium	European Nucleotide Archive	https://www.ebi.ac.uk/ena/data/search?query=PRJEB12501	ERS1040077	SAMEA3732928	ERX1298414	ERR1226202	ITS	ITS2	NA	TCCTCCGCTTATTGATATGC	GCATCGATGAAGAACGCAGC	2008-07-09	54.35	-122.61	Canada	0.3	780	3.3	146-193	Orthic Humo-Ferric Podzol, Gleyed Eluviated Dystric Brunisol	Subalpine fir, Douglas fir, Interior Spruce	Dfc, Boreal cool summer	OM1	C2	0	A horizon	22.0	1.9	0.1	5.0	1.4	20.6
LL041F	LL041	SBS_BC_	British Columbia	LL	Forest Soil	Whole Community DNA	PCR	Amplicon	pyrotag library	454 GS FLX Titanium	European Nucleotide Archive	https://www.ebi.ac.uk/ena/data/search?query=PRJEB12501	ERS1040078	SAMEA3732929	ERX1298415	ERR1226203	ITS	ITS2	NA	TCCTCCGCTTATTGATATGC	GCATCGATGAAGAACGCAGC	2008-07-09	54.35	-122.61	Canada	0.3	780	3.3	146-193	Orthic Humo-Ferric Podzol, Gleyed Eluviated Dystric Brunisol	Subalpine fir, Douglas fir, Interior Spruce	Dfc, Boreal cool summer	OM1	C2	0	A horizon	20.0	1.2	0.1	5.0	1.4	16.7
LL042F	LL042	SBS_BC_	British Columbia	LL	Forest Soil	Whole Community DNA	PCR	Amplicon	pyrotag library	454 GS FLX Titanium	European Nucleotide Archive	https://www.ebi.ac.uk/ena/data/search?query=PRJEB12501	ERS1040079	SAMEA3732930	ERX1298416	ERR1226204	ITS	ITS2	NA	TCCTCCGCTTATTGATATGC	GCATCGATGAAGAACGCAGC	2008-07-09	54.35	-122.61	Canada	0.3	780	3.3	146-193	Orthic Humo-Ferric Podzol, Gleyed Eluviated Dystric Brunisol	Subalpine fir, Douglas fir, Interior Spruce	Dfc, Boreal cool summer	OM1	C2	0	A horizon	19.0	1.4	0.1	5.0	1.4	17.5
LL043F	LL043	SBS_BC_	British Columbia	LL	Forest Soil	Whole Community DNA	PCR	Amplicon	pyrotag library	454 GS FLX Titanium	European Nucleotide Archive	https://www.ebi.ac.uk/ena/data/search?query=PRJEB12501	ERS1040080	SAMEA3732931	ERX1298417	ERR1226205	ITS	ITS2	NA	TCCTCCGCTTATTGATATGC	GCATCGATGAAGAACGCAGC	2008-07-09	54.35	-122.61	Canada	0.1	780	3.3	146-193	Orthic Humo-Ferric Podzol, Gleyed Eluviated Dystric Brunisol	Subalpine fir, Douglas fir, Interior Spruce	Dfc, Boreal cool summer	OM2	C2	0	O horizon	42.0	30.2	0.8	4.4	1.5	36.8
LL044F	LL044	SBS_BC_	British Columbia	LL	Forest Soil	Whole Community DNA	PCR	Amplicon	pyrotag library	454 GS FLX Titanium	European Nucleotide Archive	https://www.ebi.ac.uk/ena/data/search?query=PRJEB12501	ERS1040081	SAMEA3732932	ERX1298418	ERR1226206	ITS	ITS2	NA	TCCTCCGCTTATTGATATGC	GCATCGATGAAGAACGCAGC	2008-07-09	54.35	-122.61	Canada	0.1	780	3.3	146-193	Orthic Humo-Ferric Podzol, Gleyed Eluviated Dystric Brunisol	Subalpine fir, Douglas fir, Interior Spruce	Dfc, Boreal cool summer	OM2	C2	0	O horizon	59.0	30.2	0.8	4.4	1.5	36.8
LL045F	LL045	SBS_BC_	British Columbia	LL	Forest Soil	Whole Community DNA	PCR	Amplicon	pyrotag library	454 GS FLX Titanium	European Nucleotide Archive	https://www.ebi.ac.uk/ena/data/search?query=PRJEB12501	ERS1040082	SAMEA3732933	ERX1298419	ERR1226207	ITS	ITS2	NA	TCCTCCGCTTATTGATATGC	GCATCGATGAAGAACGCAGC	2008-07-09	54.35	-122.61	Canada	0.1	780	3.3	146-193	Orthic Humo-Ferric Podzol, Gleyed Eluviated Dystric Brunisol	Subalpine fir, Douglas fir, Interior Spruce	Dfc, Boreal cool summer	OM2	C2	0	O horizon	46.0	30.2	0.8	4.4	1.5	36.8
LL046F	LL046	SBS_BC_	British Columbia	LL	Forest Soil	Whole Community DNA	PCR	Amplicon	pyrotag library	454 GS FLX Titanium	European Nucleotide Archive	https://www.ebi.ac.uk/ena/data/search?query=PRJEB12501	ERS1040083	SAMEA3732934	ERX1298420	ERR1226208	ITS	ITS2	NA	TCCTCCGCTTATTGATATGC	GCATCGATGAAGAACGCAGC	2008-07-09	54.35	-122.61	Canada	0.3	780	3.3	146-193	Orthic Humo-Ferric Podzol, Gleyed Eluviated Dystric Brunisol	Subalpine fir, Douglas fir, Interior Spruce	Dfc, Boreal cool summer	OM2	C2	0	A horizon	22.0	1.6	0.1	5.0	1.5	21.0
LL047F	LL047	SBS_BC_	British Columbia	LL	Forest Soil	Whole Community DNA	PCR	Amplicon	pyrotag library	454 GS FLX Titanium	European Nucleotide Archive	https://www.ebi.ac.uk/ena/data/search?query=PRJEB12501	ERS1040084	SAMEA3732935	ERX1298421	ERR1226209	ITS	ITS2	NA	TCCTCCGCTTATTGATATGC	GCATCGATGAAGAACGCAGC	2008-07-09	54.35	-122.61	Canada	0.3	780	3.3	146-193	Orthic Humo-Ferric Podzol, Gleyed Eluviated Dystric Brunisol	Subalpine fir, Douglas fir, Interior Spruce	Dfc, Boreal cool summer	OM2	C2	0	A horizon	21.0	1.6	0.1	5.0	1.5	21.0
LL048F	LL048	SBS_BC_	British Columbia	LL	Forest Soil	Whole Community DNA	PCR	Amplicon	pyrotag library	454 GS FLX Titanium	European Nucleotide Archive	https://www.ebi.ac.uk/ena/data/search?query=PRJEB12501	ERS1040085	SAMEA3732936	ERX1298422	ERR1226210	ITS	ITS2	NA	TCCTCCGCTTATTGATATGC	GCATCGATGAAGAACGCAGC	2008-07-09	54.35	-122.61	Canada	0.3	780	3.3	146-193	Orthic Humo-Ferric Podzol, Gleyed Eluviated Dystric Brunisol	Subalpine fir, Douglas fir, Interior Spruce	Dfc, Boreal cool summer	OM2	C2	0	A horizon	23.0	1.6	0.1	5.0	1.5	21.0
LL052F	LL052	SBS_BC_	British Columbia	LL	Forest Soil	Whole Community DNA	PCR	Amplicon	pyrotag library	454 GS FLX Titanium	European Nucleotide Archive	https://www.ebi.ac.uk/ena/data/search?query=PRJEB12501	ERS1040086	SAMEA3732937	ERX1298423	ERR1226211	ITS	ITS2	NA	TCCTCCGCTTATTGATATGC	GCATCGATGAAGAACGCAGC	2008-07-09	54.35	-122.61	Canada	0.3	780	3.3	146-193	Orthic Humo-Ferric Podzol, Gleyed Eluviated Dystric Brunisol	Subalpine fir, Douglas fir, Interior Spruce	Dfc, Boreal cool summer	OM3	C2	0	A horizon	18.0	1.4	0.1	5.1	1.5	22.8
LL053F	LL053	SBS_BC_	British Columbia	LL	Forest Soil	Whole Community DNA	PCR	Amplicon	pyrotag library	454 GS FLX Titanium	European Nucleotide Archive	https://www.ebi.ac.uk/ena/data/search?query=PRJEB12501	ERS1040087	SAMEA3732938	ERX1298424	ERR1226212	ITS	ITS2	NA	TCCTCCGCTTATTGATATGC	GCATCGATGAAGAACGCAGC	2008-07-09	54.35	-122.61	Canada	0.3	780	3.3	146-193	Orthic Humo-Ferric Podzol, Gleyed Eluviated Dystric Brunisol	Subalpine fir, Douglas fir, Interior Spruce	Dfc, Boreal cool summer	OM3	C2	0	A horizon	21.0	1.4	0.1	5.1	1.5	22.8
LL054F	LL054	SBS_BC_	British Columbia	LL	Forest Soil	Whole Community DNA	PCR	Amplicon	pyrotag library	454 GS FLX Titanium	European Nucleotide Archive	https://www.ebi.ac.uk/ena/data/search?query=PRJEB12501	ERS1040088	SAMEA3732939	ERX1298425	ERR1226213	ITS	ITS2	NA	TCCTCCGCTTATTGATATGC	GCATCGATGAAGAACGCAGC	2008-07-09	54.35	-122.61	Canada	0.3	780	3.3	146-193	Orthic Humo-Ferric Podzol, Gleyed Eluviated Dystric Brunisol	Subalpine fir, Douglas fir, Interior Spruce	Dfc, Boreal cool summer	OM3	C2	0	A horizon	21.0	1.4	0.1	5.1	1.5	22.8
LL055F	LL055	SBS_BC_	British Columbia	LL	Forest Soil	Whole Community DNA	PCR	Amplicon	pyrotag library	454 GS FLX Titanium	European Nucleotide Archive	https://www.ebi.ac.uk/ena/data/search?query=PRJEB12501	ERS1040089	SAMEA3732940	ERX1298426	ERR1226214	ITS	ITS2	NA	TCCTCCGCTTATTGATATGC	GCATCGATGAAGAACGCAGC	2008-07-09	54.35	-122.61	Canada	0.1	780	3.3	146-193	Orthic Humo-Ferric Podzol, Gleyed Eluviated Dystric Brunisol	Subalpine fir, Douglas fir, Interior Spruce	Dfc, Boreal cool summer	REF	REF	0	O horizon	56.0	37.8	1.0	4.4	NA	36.4
LL056F	LL056	SBS_BC_	British Columbia	LL	Forest Soil	Whole Community DNA	PCR	Amplicon	pyrotag library	454 GS FLX Titanium	European Nucleotide Archive	https://www.ebi.ac.uk/ena/data/search?query=PRJEB12501	ERS1040090	SAMEA3732941	ERX1298427	ERR1226215	ITS	ITS2	NA	TCCTCCGCTTATTGATATGC	GCATCGATGAAGAACGCAGC	2008-07-09	54.35	-122.61	Canada	0.1	780	3.3	146-193	Orthic Humo-Ferric Podzol, Gleyed Eluviated Dystric Brunisol	Subalpine fir, Douglas fir, Interior Spruce	Dfc, Boreal cool summer	REF	REF	0	O horizon	59.0	37.8	1.0	4.4	NA	36.4
LL057F	LL057	SBS_BC_	British Columbia	LL	Forest Soil	Whole Community DNA	PCR	Amplicon	pyrotag library	454 GS FLX Titanium	European Nucleotide Archive	https://www.ebi.ac.uk/ena/data/search?query=PRJEB12501	ERS1040091	SAMEA3732942	ERX1298428	ERR1226216	ITS	ITS2	NA	TCCTCCGCTTATTGATATGC	GCATCGATGAAGAACGCAGC	2008-07-09	54.35	-122.61	Canada	0.1	780	3.3	146-193	Orthic Humo-Ferric Podzol, Gleyed Eluviated Dystric Brunisol	Subalpine fir, Douglas fir, Interior Spruce	Dfc, Boreal cool summer	REF	REF	0	O horizon	56.0	37.8	1.0	4.4	NA	36.4
LL058F	LL058	SBS_BC_	British Columbia	LL	Forest Soil	Whole Community DNA	PCR	Amplicon	pyrotag library	454 GS FLX Titanium	European Nucleotide Archive	https://www.ebi.ac.uk/ena/data/search?query=PRJEB12501	ERS1040092	SAMEA3732943	ERX1298429	ERR1226217	ITS	ITS2	NA	TCCTCCGCTTATTGATATGC	GCATCGATGAAGAACGCAGC	2008-07-09	54.35	-122.61	Canada	0.3	780	3.3	146-193	Orthic Humo-Ferric Podzol, Gleyed Eluviated Dystric Brunisol	Subalpine fir, Douglas fir, Interior Spruce	Dfc, Boreal cool summer	REF	REF	0	A horizon	19.0	1.6	0.1	4.9	NA	22.3
LL059F	LL059	SBS_BC_	British Columbia	LL	Forest Soil	Whole Community DNA	PCR	Amplicon	pyrotag library	454 GS FLX Titanium	European Nucleotide Archive	https://www.ebi.ac.uk/ena/data/search?query=PRJEB12501	ERS1040093	SAMEA3732944	ERX1298430	ERR1226218	ITS	ITS2	NA	TCCTCCGCTTATTGATATGC	GCATCGATGAAGAACGCAGC	2008-07-09	54.35	-122.61	Canada	0.3	780	3.3	146-193	Orthic Humo-Ferric Podzol, Gleyed Eluviated Dystric Brunisol	Subalpine fir, Douglas fir, Interior Spruce	Dfc, Boreal cool summer	REF	REF	0	A horizon	20.0	1.6	0.1	4.9	NA	22.3
LL060F	LL060	SBS_BC_	British Columbia	LL	Forest Soil	Whole Community DNA	PCR	Amplicon	pyrotag library	454 GS FLX Titanium	European Nucleotide Archive	https://www.ebi.ac.uk/ena/data/search?query=PRJEB12501	ERS1040094	SAMEA3732945	ERX1298431	ERR1226219	ITS	ITS2	NA	TCCTCCGCTTATTGATATGC	GCATCGATGAAGAACGCAGC	2008-07-09	54.35	-122.61	Canada	0.3	780	3.3	146-193	Orthic Humo-Ferric Podzol, Gleyed Eluviated Dystric Brunisol	Subalpine fir, Douglas fir, Interior Spruce	Dfc, Boreal cool summer	REF	REF	0	A horizon	22.0	1.6	0.1	4.9	NA	22.3
OC304F	OL304	IDF_BC_	British Columbia	OC	Forest Soil	Whole Community DNA	PCR	Amplicon	pyrotag library	454 GS FLX Titanium	European Nucleotide Archive	https://www.ebi.ac.uk/ena/data/search?query=PRJEB12501	ERS1040095	SAMEA3732946	ERX1298432	ERR1226220	ITS	ITS2	NA	TCCTCCGCTTATTGATATGC	GCATCGATGAAGAACGCAGC	2010-06-26	50.88	-120.35	Canada	0.3	1075	2.5	300	Brunisolic Gray Luvisol	Douglas fir	Dfb, Humid Continental warm summer	OM3	C0	0	A horizon	23.5	NA	NA	0.0	NA	NA
OC305F	OL305	IDF_BC_	British Columbia	OC	Forest Soil	Whole Community DNA	PCR	Amplicon	pyrotag library	454 GS FLX Titanium	European Nucleotide Archive	https://www.ebi.ac.uk/ena/data/search?query=PRJEB12501	ERS1040096	SAMEA3732947	ERX1298433	ERR1226221	ITS	ITS2	NA	TCCTCCGCTTATTGATATGC	GCATCGATGAAGAACGCAGC	2010-06-26	50.88	-120.35	Canada	0.3	1075	2.5	300	Brunisolic Gray Luvisol	Douglas fir	Dfb, Humid Continental warm summer	OM3	C0	0	A horizon	17.3	NA	NA	0.0	NA	NA
OC306F	OL306	IDF_BC_	British Columbia	OC	Forest Soil	Whole Community DNA	PCR	Amplicon	pyrotag library	454 GS FLX Titanium	European Nucleotide Archive	https://www.ebi.ac.uk/ena/data/search?query=PRJEB12501	ERS1040097	SAMEA3732948	ERX1298434	ERR1226222	ITS	ITS2	NA	TCCTCCGCTTATTGATATGC	GCATCGATGAAGAACGCAGC	2010-06-26	50.88	-120.35	Canada	0.3	1075	2.5	300	Brunisolic Gray Luvisol	Douglas fir	Dfb, Humid Continental warm summer	OM3	C0	0	A horizon	22.0	NA	NA	0.0	NA	NA
OC307F	OL307	IDF_BC_	British Columbia	OC	Forest Soil	Whole Community DNA	PCR	Amplicon	pyrotag library	454 GS FLX Titanium	European Nucleotide Archive	https://www.ebi.ac.uk/ena/data/search?query=PRJEB12501	ERS1040098	SAMEA3732949	ERX1298435	ERR1226223	ITS	ITS2	NA	TCCTCCGCTTATTGATATGC	GCATCGATGAAGAACGCAGC	2010-06-26	50.88	-120.35	Canada	0.1	1075	2.5	300	Brunisolic Gray Luvisol	Douglas fir	Dfb, Humid Continental warm summer	OM1	C0	0	O horizon	59.5	NA	NA	0.0	NA	NA
OC308F	OL308	IDF_BC_	British Columbia	OC	Forest Soil	Whole Community DNA	PCR	Amplicon	pyrotag library	454 GS FLX Titanium	European Nucleotide Archive	https://www.ebi.ac.uk/ena/data/search?query=PRJEB12501	ERS1040099	SAMEA3732950	ERX1298436	ERR1226224	ITS	ITS2	NA	TCCTCCGCTTATTGATATGC	GCATCGATGAAGAACGCAGC	2010-06-26	50.88	-120.35	Canada	0.1	1075	2.5	300	Brunisolic Gray Luvisol	Douglas fir	Dfb, Humid Continental warm summer	OM1	C0	0	O horizon	64.7	NA	NA	0.0	NA	NA
OC309F	OL309	IDF_BC_	British Columbia	OC	Forest Soil	Whole Community DNA	PCR	Amplicon	pyrotag library	454 GS FLX Titanium	European Nucleotide Archive	https://www.ebi.ac.uk/ena/data/search?query=PRJEB12501	ERS1040100	SAMEA3732951	ERX1298437	ERR1226225	ITS	ITS2	NA	TCCTCCGCTTATTGATATGC	GCATCGATGAAGAACGCAGC	2010-06-26	50.88	-120.35	Canada	0.1	1075	2.5	300	Brunisolic Gray Luvisol	Douglas fir	Dfb, Humid Continental warm summer	OM1	C0	0	O horizon	61.8	NA	NA	0.0	NA	NA
OC310F	OL310	IDF_BC_	British Columbia	OC	Forest Soil	Whole Community DNA	PCR	Amplicon	pyrotag library	454 GS FLX Titanium	European Nucleotide Archive	https://www.ebi.ac.uk/ena/data/search?query=PRJEB12501	ERS1040101	SAMEA3732952	ERX1298438	ERR1226226	ITS	ITS2	NA	TCCTCCGCTTATTGATATGC	GCATCGATGAAGAACGCAGC	2010-06-26	50.88	-120.35	Canada	0.3	1075	2.5	300	Brunisolic Gray Luvisol	Douglas fir	Dfb, Humid Continental warm summer	OM1	C0	0	A horizon	26.0	NA	NA	0.0	NA	NA
OC311F	OL311	IDF_BC_	British Columbia	OC	Forest Soil	Whole Community DNA	PCR	Amplicon	pyrotag library	454 GS FLX Titanium	European Nucleotide Archive	https://www.ebi.ac.uk/ena/data/search?query=PRJEB12501	ERS1040102	SAMEA3732953	ERX1298439	ERR1226227	ITS	ITS2	NA	TCCTCCGCTTATTGATATGC	GCATCGATGAAGAACGCAGC	2010-06-26	50.88	-120.35	Canada	0.3	1075	2.5	300	Brunisolic Gray Luvisol	Douglas fir	Dfb, Humid Continental warm summer	OM1	C0	0	A horizon	26.6	NA	NA	0.0	NA	NA
OC312F	OL312	IDF_BC_	British Columbia	OC	Forest Soil	Whole Community DNA	PCR	Amplicon	pyrotag library	454 GS FLX Titanium	European Nucleotide Archive	https://www.ebi.ac.uk/ena/data/search?query=PRJEB12501	ERS1040103	SAMEA3732954	ERX1298440	ERR1226228	ITS	ITS2	NA	TCCTCCGCTTATTGATATGC	GCATCGATGAAGAACGCAGC	2010-06-26	50.88	-120.35	Canada	0.3	1075	2.5	300	Brunisolic Gray Luvisol	Douglas fir	Dfb, Humid Continental warm summer	OM1	C0	0	A horizon	28.2	NA	NA	0.0	NA	NA
OC313F	OL313	IDF_BC_	British Columbia	OC	Forest Soil	Whole Community DNA	PCR	Amplicon	pyrotag library	454 GS FLX Titanium	European Nucleotide Archive	https://www.ebi.ac.uk/ena/data/search?query=PRJEB12501	ERS1040104	SAMEA3732955	ERX1298441	ERR1226229	ITS	ITS2	NA	TCCTCCGCTTATTGATATGC	GCATCGATGAAGAACGCAGC	2010-06-26	50.88	-120.35	Canada	0.1	1075	2.5	300	Brunisolic Gray Luvisol	Douglas fir	Dfb, Humid Continental warm summer	OM2	C1	0	O horizon	58.0	NA	NA	0.0	NA	NA
OC314F	OL314	IDF_BC_	British Columbia	OC	Forest Soil	Whole Community DNA	PCR	Amplicon	pyrotag library	454 GS FLX Titanium	European Nucleotide Archive	https://www.ebi.ac.uk/ena/data/search?query=PRJEB12501	ERS1040105	SAMEA3732956	ERX1298442	ERR1226230	ITS	ITS2	NA	TCCTCCGCTTATTGATATGC	GCATCGATGAAGAACGCAGC	2010-06-26	50.88	-120.35	Canada	0.1	1075	2.5	300	Brunisolic Gray Luvisol	Douglas fir	Dfb, Humid Continental warm summer	OM2	C1	0	O horizon	63.5	NA	NA	0.0	NA	NA
OC315F	OL315	IDF_BC_	British Columbia	OC	Forest Soil	Whole Community DNA	PCR	Amplicon	pyrotag library	454 GS FLX Titanium	European Nucleotide Archive	https://www.ebi.ac.uk/ena/data/search?query=PRJEB12501	ERS1040106	SAMEA3732957	ERX1298443	ERR1226231	ITS	ITS2	NA	TCCTCCGCTTATTGATATGC	GCATCGATGAAGAACGCAGC	2010-06-26	50.88	-120.35	Canada	0.1	1075	2.5	300	Brunisolic Gray Luvisol	Douglas fir	Dfb, Humid Continental warm summer	OM2	C1	0	O horizon	57.2	NA	NA	0.0	NA	NA
OC316F	OL316	IDF_BC_	British Columbia	OC	Forest Soil	Whole Community DNA	PCR	Amplicon	pyrotag library	454 GS FLX Titanium	European Nucleotide Archive	https://www.ebi.ac.uk/ena/data/search?query=PRJEB12501	ERS1040107	SAMEA3732958	ERX1298444	ERR1226232	ITS	ITS2	NA	TCCTCCGCTTATTGATATGC	GCATCGATGAAGAACGCAGC	2010-06-26	50.88	-120.35	Canada	0.3	1075	2.5	300	Brunisolic Gray Luvisol	Douglas fir	Dfb, Humid Continental warm summer	OM2	C1	0	A horizon	24.3	NA	NA	0.0	NA	NA
OC317F	OL317	IDF_BC_	British Columbia	OC	Forest Soil	Whole Community DNA	PCR	Amplicon	pyrotag library	454 GS FLX Titanium	European Nucleotide Archive	https://www.ebi.ac.uk/ena/data/search?query=PRJEB12501	ERS1040108	SAMEA3732959	ERX1298445	ERR1226233	ITS	ITS2	NA	TCCTCCGCTTATTGATATGC	GCATCGATGAAGAACGCAGC	2010-06-26	50.88	-120.35	Canada	0.3	1075	2.5	300	Brunisolic Gray Luvisol	Douglas fir	Dfb, Humid Continental warm summer	OM2	C1	0	A horizon	28.6	NA	NA	0.0	NA	NA
OC318F	OL318	IDF_BC_	British Columbia	OC	Forest Soil	Whole Community DNA	PCR	Amplicon	pyrotag library	454 GS FLX Titanium	European Nucleotide Archive	https://www.ebi.ac.uk/ena/data/search?query=PRJEB12501	ERS1040109	SAMEA3732960	ERX1298446	ERR1226234	ITS	ITS2	NA	TCCTCCGCTTATTGATATGC	GCATCGATGAAGAACGCAGC	2010-06-26	50.88	-120.35	Canada	0.3	1075	2.5	300	Brunisolic Gray Luvisol	Douglas fir	Dfb, Humid Continental warm summer	OM2	C1	0	A horizon	21.1	NA	NA	0.0	NA	NA
OC319F	OL319	IDF_BC_	British Columbia	OC	Forest Soil	Whole Community DNA	PCR	Amplicon	pyrotag library	454 GS FLX Titanium	European Nucleotide Archive	https://www.ebi.ac.uk/ena/data/search?query=PRJEB12501	ERS1040110	SAMEA3732961	ERX1298447	ERR1226235	ITS	ITS2	NA	TCCTCCGCTTATTGATATGC	GCATCGATGAAGAACGCAGC	2010-06-26	50.88	-120.35	Canada	0.1	1075	2.5	300	Brunisolic Gray Luvisol	Douglas fir	Dfb, Humid Continental warm summer	OM2	C2	0	O horizon	61.5	NA	NA	0.0	NA	NA
OC320F	OL320	IDF_BC_	British Columbia	OC	Forest Soil	Whole Community DNA	PCR	Amplicon	pyrotag library	454 GS FLX Titanium	European Nucleotide Archive	https://www.ebi.ac.uk/ena/data/search?query=PRJEB12501	ERS1040111	SAMEA3732962	ERX1298448	ERR1226236	ITS	ITS2	NA	TCCTCCGCTTATTGATATGC	GCATCGATGAAGAACGCAGC	2010-06-26	50.88	-120.35	Canada	0.1	1075	2.5	300	Brunisolic Gray Luvisol	Douglas fir	Dfb, Humid Continental warm summer	OM2	C2	0	O horizon	62.7	NA	NA	0.0	NA	NA
OC321F	OL321	IDF_BC_	British Columbia	OC	Forest Soil	Whole Community DNA	PCR	Amplicon	pyrotag library	454 GS FLX Titanium	European Nucleotide Archive	https://www.ebi.ac.uk/ena/data/search?query=PRJEB12501	ERS1040112	SAMEA3732963	ERX1298449	ERR1226237	ITS	ITS2	NA	TCCTCCGCTTATTGATATGC	GCATCGATGAAGAACGCAGC	2010-06-26	50.88	-120.35	Canada	0.1	1075	2.5	300	Brunisolic Gray Luvisol	Douglas fir	Dfb, Humid Continental warm summer	OM2	C2	0	O horizon	57.7	NA	NA	0.0	NA	NA
OC322F	OL322	IDF_BC_	British Columbia	OC	Forest Soil	Whole Community DNA	PCR	Amplicon	pyrotag library	454 GS FLX Titanium	European Nucleotide Archive	https://www.ebi.ac.uk/ena/data/search?query=PRJEB12501	ERS1040113	SAMEA3732964	ERX1298450	ERR1226238	ITS	ITS2	NA	TCCTCCGCTTATTGATATGC	GCATCGATGAAGAACGCAGC	2010-06-26	50.88	-120.35	Canada	0.3	1075	2.5	300	Brunisolic Gray Luvisol	Douglas fir	Dfb, Humid Continental warm summer	OM2	C2	0	A horizon	27.7	NA	NA	0.0	NA	NA
OC323F	OL323	IDF_BC_	British Columbia	OC	Forest Soil	Whole Community DNA	PCR	Amplicon	pyrotag library	454 GS FLX Titanium	European Nucleotide Archive	https://www.ebi.ac.uk/ena/data/search?query=PRJEB12501	ERS1040114	SAMEA3732965	ERX1298451	ERR1226239	ITS	ITS2	NA	TCCTCCGCTTATTGATATGC	GCATCGATGAAGAACGCAGC	2010-06-26	50.88	-120.35	Canada	0.3	1075	2.5	300	Brunisolic Gray Luvisol	Douglas fir	Dfb, Humid Continental warm summer	OM2	C2	0	A horizon	23.4	NA	NA	0.0	NA	NA
OC324F	OL324	IDF_BC_	British Columbia	OC	Forest Soil	Whole Community DNA	PCR	Amplicon	pyrotag library	454 GS FLX Titanium	European Nucleotide Archive	https://www.ebi.ac.uk/ena/data/search?query=PRJEB12501	ERS1040115	SAMEA3732966	ERX1298452	ERR1226240	ITS	ITS2	NA	TCCTCCGCTTATTGATATGC	GCATCGATGAAGAACGCAGC	2010-06-26	50.88	-120.35	Canada	0.3	1075	2.5	300	Brunisolic Gray Luvisol	Douglas fir	Dfb, Humid Continental warm summer	OM2	C2	0	A horizon	23.7	NA	NA	0.0	NA	NA
OC325F	OL325	IDF_BC_	British Columbia	OC	Forest Soil	Whole Community DNA	PCR	Amplicon	pyrotag library	454 GS FLX Titanium	European Nucleotide Archive	https://www.ebi.ac.uk/ena/data/search?query=PRJEB12501	ERS1040116	SAMEA3732967	ERX1298453	ERR1226241	ITS	ITS2	NA	TCCTCCGCTTATTGATATGC	GCATCGATGAAGAACGCAGC	2010-06-26	50.88	-120.35	Canada	0.1	1075	2.5	300	Brunisolic Gray Luvisol	Douglas fir	Dfb, Humid Continental warm summer	OM2	C0	0	O horizon	64.4	NA	NA	0.0	NA	NA
OC326F	OL326	IDF_BC_	British Columbia	OC	Forest Soil	Whole Community DNA	PCR	Amplicon	pyrotag library	454 GS FLX Titanium	European Nucleotide Archive	https://www.ebi.ac.uk/ena/data/search?query=PRJEB12501	ERS1040117	SAMEA3732968	ERX1298454	ERR1226242	ITS	ITS2	NA	TCCTCCGCTTATTGATATGC	GCATCGATGAAGAACGCAGC	2010-06-26	50.88	-120.35	Canada	0.1	1075	2.5	300	Brunisolic Gray Luvisol	Douglas fir	Dfb, Humid Continental warm summer	OM2	C0	0	O horizon	62.2	NA	NA	0.0	NA	NA
OC327F	OL327	IDF_BC_	British Columbia	OC	Forest Soil	Whole Community DNA	PCR	Amplicon	pyrotag library	454 GS FLX Titanium	European Nucleotide Archive	https://www.ebi.ac.uk/ena/data/search?query=PRJEB12501	ERS1040118	SAMEA3732969	ERX1298455	ERR1226243	ITS	ITS2	NA	TCCTCCGCTTATTGATATGC	GCATCGATGAAGAACGCAGC	2010-06-26	50.88	-120.35	Canada	0.1	1075	2.5	300	Brunisolic Gray Luvisol	Douglas fir	Dfb, Humid Continental warm summer	OM2	C0	0	O horizon	53.7	NA	NA	0.0	NA	NA
OC328F	OL328	IDF_BC_	British Columbia	OC	Forest Soil	Whole Community DNA	PCR	Amplicon	pyrotag library	454 GS FLX Titanium	European Nucleotide Archive	https://www.ebi.ac.uk/ena/data/search?query=PRJEB12501	ERS1040119	SAMEA3732970	ERX1298456	ERR1226244	ITS	ITS2	NA	TCCTCCGCTTATTGATATGC	GCATCGATGAAGAACGCAGC	2010-06-26	50.88	-120.35	Canada	0.3	1075	2.5	300	Brunisolic Gray Luvisol	Douglas fir	Dfb, Humid Continental warm summer	OM2	C0	0	A horizon	26.1	NA	NA	0.0	NA	NA
OC329F	OL329	IDF_BC_	British Columbia	OC	Forest Soil	Whole Community DNA	PCR	Amplicon	pyrotag library	454 GS FLX Titanium	European Nucleotide Archive	https://www.ebi.ac.uk/ena/data/search?query=PRJEB12501	ERS1040120	SAMEA3732971	ERX1298457	ERR1226245	ITS	ITS2	NA	TCCTCCGCTTATTGATATGC	GCATCGATGAAGAACGCAGC	2010-06-26	50.88	-120.35	Canada	0.3	1075	2.5	300	Brunisolic Gray Luvisol	Douglas fir	Dfb, Humid Continental warm summer	OM2	C0	0	A horizon	22.3	NA	NA	0.0	NA	NA
OC330F	OL330	IDF_BC_	British Columbia	OC	Forest Soil	Whole Community DNA	PCR	Amplicon	pyrotag library	454 GS FLX Titanium	European Nucleotide Archive	https://www.ebi.ac.uk/ena/data/search?query=PRJEB12501	ERS1040121	SAMEA3732972	ERX1298458	ERR1226246	ITS	ITS2	NA	TCCTCCGCTTATTGATATGC	GCATCGATGAAGAACGCAGC	2010-06-26	50.88	-120.35	Canada	0.3	1075	2.5	300	Brunisolic Gray Luvisol	Douglas fir	Dfb, Humid Continental warm summer	OM2	C0	0	A horizon	23.9	NA	NA	0.0	NA	NA
OC331F	OL331	IDF_BC_	British Columbia	OC	Forest Soil	Whole Community DNA	PCR	Amplicon	pyrotag library	454 GS FLX Titanium	European Nucleotide Archive	https://www.ebi.ac.uk/ena/data/search?query=PRJEB12501	ERS1040122	SAMEA3732973	ERX1298459	ERR1226247	ITS	ITS2	NA	TCCTCCGCTTATTGATATGC	GCATCGATGAAGAACGCAGC	2010-06-26	50.88	-120.35	Canada	0.1	1075	2.5	300	Brunisolic Gray Luvisol	Douglas fir	Dfb, Humid Continental warm summer	OM1	C2	0	O horizon	57.4	NA	NA	0.0	NA	NA
OC332F	OL332	IDF_BC_	British Columbia	OC	Forest Soil	Whole Community DNA	PCR	Amplicon	pyrotag library	454 GS FLX Titanium	European Nucleotide Archive	https://www.ebi.ac.uk/ena/data/search?query=PRJEB12501	ERS1040123	SAMEA3732974	ERX1298460	ERR1226248	ITS	ITS2	NA	TCCTCCGCTTATTGATATGC	GCATCGATGAAGAACGCAGC	2010-06-26	50.88	-120.35	Canada	0.1	1075	2.5	300	Brunisolic Gray Luvisol	Douglas fir	Dfb, Humid Continental warm summer	OM1	C2	0	O horizon	50.5	NA	NA	0.0	NA	NA
OC333F	OL333	IDF_BC_	British Columbia	OC	Forest Soil	Whole Community DNA	PCR	Amplicon	pyrotag library	454 GS FLX Titanium	European Nucleotide Archive	https://www.ebi.ac.uk/ena/data/search?query=PRJEB12501	ERS1040124	SAMEA3732975	ERX1298461	ERR1226249	ITS	ITS2	NA	TCCTCCGCTTATTGATATGC	GCATCGATGAAGAACGCAGC	2010-06-26	50.88	-120.35	Canada	0.1	1075	2.5	300	Brunisolic Gray Luvisol	Douglas fir	Dfb, Humid Continental warm summer	OM1	C2	0	O horizon	60.9	NA	NA	0.0	NA	NA
OC334F	OL334	IDF_BC_	British Columbia	OC	Forest Soil	Whole Community DNA	PCR	Amplicon	pyrotag library	454 GS FLX Titanium	European Nucleotide Archive	https://www.ebi.ac.uk/ena/data/search?query=PRJEB12501	ERS1040125	SAMEA3732976	ERX1298462	ERR1226250	ITS	ITS2	NA	TCCTCCGCTTATTGATATGC	GCATCGATGAAGAACGCAGC	2010-06-26	50.88	-120.35	Canada	0.3	1075	2.5	300	Brunisolic Gray Luvisol	Douglas fir	Dfb, Humid Continental warm summer	OM1	C2	0	A horizon	24.0	NA	NA	0.0	NA	NA
OC335F	OL335	IDF_BC_	British Columbia	OC	Forest Soil	Whole Community DNA	PCR	Amplicon	pyrotag library	454 GS FLX Titanium	European Nucleotide Archive	https://www.ebi.ac.uk/ena/data/search?query=PRJEB12501	ERS1040126	SAMEA3732977	ERX1298463	ERR1226251	ITS	ITS2	NA	TCCTCCGCTTATTGATATGC	GCATCGATGAAGAACGCAGC	2010-06-26	50.88	-120.35	Canada	0.3	1075	2.5	300	Brunisolic Gray Luvisol	Douglas fir	Dfb, Humid Continental warm summer	OM1	C2	0	A horizon	25.6	NA	NA	0.0	NA	NA
OC336F	OL336	IDF_BC_	British Columbia	OC	Forest Soil	Whole Community DNA	PCR	Amplicon	pyrotag library	454 GS FLX Titanium	European Nucleotide Archive	https://www.ebi.ac.uk/ena/data/search?query=PRJEB12501	ERS1040127	SAMEA3732978	ERX1298464	ERR1226252	ITS	ITS2	NA	TCCTCCGCTTATTGATATGC	GCATCGATGAAGAACGCAGC	2010-06-26	50.88	-120.35	Canada	0.3	1075	2.5	300	Brunisolic Gray Luvisol	Douglas fir	Dfb, Humid Continental warm summer	OM1	C2	0	A horizon	25.4	NA	NA	0.0	NA	NA
OC340F	OL340	IDF_BC_	British Columbia	OC	Forest Soil	Whole Community DNA	PCR	Amplicon	pyrotag library	454 GS FLX Titanium	European Nucleotide Archive	https://www.ebi.ac.uk/ena/data/search?query=PRJEB12501	ERS1040128	SAMEA3732979	ERX1298465	ERR1226253	ITS	ITS2	NA	TCCTCCGCTTATTGATATGC	GCATCGATGAAGAACGCAGC	2010-06-26	50.88	-120.35	Canada	0.3	1075	2.5	300	Brunisolic Gray Luvisol	Douglas fir	Dfb, Humid Continental warm summer	OM3	C2	0	A horizon	22.9	NA	NA	0.0	NA	NA
OC341F	OL341	IDF_BC_	British Columbia	OC	Forest Soil	Whole Community DNA	PCR	Amplicon	pyrotag library	454 GS FLX Titanium	European Nucleotide Archive	https://www.ebi.ac.uk/ena/data/search?query=PRJEB12501	ERS1040129	SAMEA3732980	ERX1298466	ERR1226254	ITS	ITS2	NA	TCCTCCGCTTATTGATATGC	GCATCGATGAAGAACGCAGC	2010-06-26	50.88	-120.35	Canada	0.3	1075	2.5	300	Brunisolic Gray Luvisol	Douglas fir	Dfb, Humid Continental warm summer	OM3	C2	0	A horizon	22.1	NA	NA	0.0	NA	NA
OC342F	OL342	IDF_BC_	British Columbia	OC	Forest Soil	Whole Community DNA	PCR	Amplicon	pyrotag library	454 GS FLX Titanium	European Nucleotide Archive	https://www.ebi.ac.uk/ena/data/search?query=PRJEB12501	ERS1040130	SAMEA3732981	ERX1298467	ERR1226255	ITS	ITS2	NA	TCCTCCGCTTATTGATATGC	GCATCGATGAAGAACGCAGC	2010-06-26	50.88	-120.35	Canada	0.3	1075	2.5	300	Brunisolic Gray Luvisol	Douglas fir	Dfb, Humid Continental warm summer	OM3	C2	0	A horizon	20.3	NA	NA	0.0	NA	NA
OC343F	OL343	IDF_BC_	British Columbia	OC	Forest Soil	Whole Community DNA	PCR	Amplicon	pyrotag library	454 GS FLX Titanium	European Nucleotide Archive	https://www.ebi.ac.uk/ena/data/search?query=PRJEB12501	ERS1040131	SAMEA3732982	ERX1298468	ERR1226256	ITS	ITS2	NA	TCCTCCGCTTATTGATATGC	GCATCGATGAAGAACGCAGC	2010-06-26	50.88	-120.35	Canada	0.1	1075	2.5	300	Brunisolic Gray Luvisol	Douglas fir	Dfb, Humid Continental warm summer	OM1	C1	0	O horizon	54.7	NA	NA	0.0	NA	NA
OC344F	OL344	IDF_BC_	British Columbia	OC	Forest Soil	Whole Community DNA	PCR	Amplicon	pyrotag library	454 GS FLX Titanium	European Nucleotide Archive	https://www.ebi.ac.uk/ena/data/search?query=PRJEB12501	ERS1040132	SAMEA3732983	ERX1298469	ERR1226257	ITS	ITS2	NA	TCCTCCGCTTATTGATATGC	GCATCGATGAAGAACGCAGC	2010-06-26	50.88	-120.35	Canada	0.1	1075	2.5	300	Brunisolic Gray Luvisol	Douglas fir	Dfb, Humid Continental warm summer	OM1	C1	0	O horizon	58.7	NA	NA	0.0	NA	NA
OC345F	OL345	IDF_BC_	British Columbia	OC	Forest Soil	Whole Community DNA	PCR	Amplicon	pyrotag library	454 GS FLX Titanium	European Nucleotide Archive	https://www.ebi.ac.uk/ena/data/search?query=PRJEB12501	ERS1040133	SAMEA3732984	ERX1298470	ERR1226258	ITS	ITS2	NA	TCCTCCGCTTATTGATATGC	GCATCGATGAAGAACGCAGC	2010-06-26	50.88	-120.35	Canada	0.1	1075	2.5	300	Brunisolic Gray Luvisol	Douglas fir	Dfb, Humid Continental warm summer	OM1	C1	0	O horizon	57.4	NA	NA	0.0	NA	NA
OC346F	OL346	IDF_BC_	British Columbia	OC	Forest Soil	Whole Community DNA	PCR	Amplicon	pyrotag library	454 GS FLX Titanium	European Nucleotide Archive	https://www.ebi.ac.uk/ena/data/search?query=PRJEB12501	ERS1040134	SAMEA3732985	ERX1298471	ERR1226259	ITS	ITS2	NA	TCCTCCGCTTATTGATATGC	GCATCGATGAAGAACGCAGC	2010-06-26	50.88	-120.35	Canada	0.3	1075	2.5	300	Brunisolic Gray Luvisol	Douglas fir	Dfb, Humid Continental warm summer	OM1	C1	0	A horizon	26.2	NA	NA	0.0	NA	NA
OC347F	OL347	IDF_BC_	British Columbia	OC	Forest Soil	Whole Community DNA	PCR	Amplicon	pyrotag library	454 GS FLX Titanium	European Nucleotide Archive	https://www.ebi.ac.uk/ena/data/search?query=PRJEB12501	ERS1040135	SAMEA3732986	ERX1298472	ERR1226260	ITS	ITS2	NA	TCCTCCGCTTATTGATATGC	GCATCGATGAAGAACGCAGC	2010-06-26	50.88	-120.35	Canada	0.3	1075	2.5	300	Brunisolic Gray Luvisol	Douglas fir	Dfb, Humid Continental warm summer	OM1	C1	0	A horizon	28.0	NA	NA	0.0	NA	NA
OC348F	OL348	IDF_BC_	British Columbia	OC	Forest Soil	Whole Community DNA	PCR	Amplicon	pyrotag library	454 GS FLX Titanium	European Nucleotide Archive	https://www.ebi.ac.uk/ena/data/search?query=PRJEB12501	ERS1040136	SAMEA3732987	ERX1298473	ERR1226261	ITS	ITS2	NA	TCCTCCGCTTATTGATATGC	GCATCGATGAAGAACGCAGC	2010-06-26	50.88	-120.35	Canada	0.3	1075	2.5	300	Brunisolic Gray Luvisol	Douglas fir	Dfb, Humid Continental warm summer	OM1	C1	0	A horizon	26.5	NA	NA	0.0	NA	NA
OC352F	OL352	IDF_BC_	British Columbia	OC	Forest Soil	Whole Community DNA	PCR	Amplicon	pyrotag library	454 GS FLX Titanium	European Nucleotide Archive	https://www.ebi.ac.uk/ena/data/search?query=PRJEB12501	ERS1040137	SAMEA3732988	ERX1298474	ERR1226262	ITS	ITS2	NA	TCCTCCGCTTATTGATATGC	GCATCGATGAAGAACGCAGC	2010-06-26	50.88	-120.35	Canada	0.3	1075	2.5	300	Brunisolic Gray Luvisol	Douglas fir	Dfb, Humid Continental warm summer	OM3	C1	0	A horizon	23.9	NA	NA	0.0	NA	NA
OC353F	OL353	IDF_BC_	British Columbia	OC	Forest Soil	Whole Community DNA	PCR	Amplicon	pyrotag library	454 GS FLX Titanium	European Nucleotide Archive	https://www.ebi.ac.uk/ena/data/search?query=PRJEB12501	ERS1040138	SAMEA3732989	ERX1298475	ERR1226263	ITS	ITS2	NA	TCCTCCGCTTATTGATATGC	GCATCGATGAAGAACGCAGC	2010-06-26	50.88	-120.35	Canada	0.3	1075	2.5	300	Brunisolic Gray Luvisol	Douglas fir	Dfb, Humid Continental warm summer	OM3	C1	0	A horizon	18.4	NA	NA	0.0	NA	NA
OC354F	OL354	IDF_BC_	British Columbia	OC	Forest Soil	Whole Community DNA	PCR	Amplicon	pyrotag library	454 GS FLX Titanium	European Nucleotide Archive	https://www.ebi.ac.uk/ena/data/search?query=PRJEB12501	ERS1040139	SAMEA3732990	ERX1298476	ERR1226264	ITS	ITS2	NA	TCCTCCGCTTATTGATATGC	GCATCGATGAAGAACGCAGC	2010-06-26	50.88	-120.35	Canada	0.3	1075	2.5	300	Brunisolic Gray Luvisol	Douglas fir	Dfb, Humid Continental warm summer	OM3	C1	0	A horizon	18.9	NA	NA	0.0	NA	NA
OC355F	OL355	IDF_BC_	British Columbia	OC	Forest Soil	Whole Community DNA	PCR	Amplicon	pyrotag library	454 GS FLX Titanium	European Nucleotide Archive	https://www.ebi.ac.uk/ena/data/search?query=PRJEB12501	ERS1040140	SAMEA3732991	ERX1298477	ERR1226265	ITS	ITS2	NA	TCCTCCGCTTATTGATATGC	GCATCGATGAAGAACGCAGC	2010-06-26	50.88	-120.35	Canada	0.1	1075	2.5	300	Brunisolic Gray Luvisol	Douglas fir	Dfb, Humid Continental warm summer	REF	REF	0	O horizon	56.5	NA	NA	0.0	NA	NA
OC356F	OL356	IDF_BC_	British Columbia	OC	Forest Soil	Whole Community DNA	PCR	Amplicon	pyrotag library	454 GS FLX Titanium	European Nucleotide Archive	https://www.ebi.ac.uk/ena/data/search?query=PRJEB12501	ERS1040141	SAMEA3732992	ERX1298478	ERR1226266	ITS	ITS2	NA	TCCTCCGCTTATTGATATGC	GCATCGATGAAGAACGCAGC	2010-06-26	50.88	-120.35	Canada	0.1	1075	2.5	300	Brunisolic Gray Luvisol	Douglas fir	Dfb, Humid Continental warm summer	REF	REF	0	O horizon	62.0	NA	NA	0.0	NA	NA
OC357F	OL357	IDF_BC_	British Columbia	OC	Forest Soil	Whole Community DNA	PCR	Amplicon	pyrotag library	454 GS FLX Titanium	European Nucleotide Archive	https://www.ebi.ac.uk/ena/data/search?query=PRJEB12501	ERS1040142	SAMEA3732993	ERX1298479	ERR1226267	ITS	ITS2	NA	TCCTCCGCTTATTGATATGC	GCATCGATGAAGAACGCAGC	2010-06-26	50.88	-120.35	Canada	0.1	1075	2.5	300	Brunisolic Gray Luvisol	Douglas fir	Dfb, Humid Continental warm summer	REF	REF	0	O horizon	62.1	NA	NA	0.0	NA	NA
OC358F	OL358	IDF_BC_	British Columbia	OC	Forest Soil	Whole Community DNA	PCR	Amplicon	pyrotag library	454 GS FLX Titanium	European Nucleotide Archive	https://www.ebi.ac.uk/ena/data/search?query=PRJEB12501	ERS1040143	SAMEA3732994	ERX1298480	ERR1226268	ITS	ITS2	NA	TCCTCCGCTTATTGATATGC	GCATCGATGAAGAACGCAGC	2010-06-26	50.88	-120.35	Canada	0.3	1075	2.5	300	Brunisolic Gray Luvisol	Douglas fir	Dfb, Humid Continental warm summer	REF	REF	0	A horizon	23.0	NA	NA	0.0	NA	NA
OC359F	OL359	IDF_BC_	British Columbia	OC	Forest Soil	Whole Community DNA	PCR	Amplicon	pyrotag library	454 GS FLX Titanium	European Nucleotide Archive	https://www.ebi.ac.uk/ena/data/search?query=PRJEB12501	ERS1040144	SAMEA3732995	ERX1298481	ERR1226269	ITS	ITS2	NA	TCCTCCGCTTATTGATATGC	GCATCGATGAAGAACGCAGC	2010-06-26	50.88	-120.35	Canada	0.3	1075	2.5	300	Brunisolic Gray Luvisol	Douglas fir	Dfb, Humid Continental warm summer	REF	REF	0	A horizon	20.6	NA	NA	0.0	NA	NA
OC360F	OL360	IDF_BC_	British Columbia	OC	Forest Soil	Whole Community DNA	PCR	Amplicon	pyrotag library	454 GS FLX Titanium	European Nucleotide Archive	https://www.ebi.ac.uk/ena/data/search?query=PRJEB12501	ERS1040145	SAMEA3732996	ERX1298482	ERR1226270	ITS	ITS2	NA	TCCTCCGCTTATTGATATGC	GCATCGATGAAGAACGCAGC	2010-06-26	50.88	-120.35	Canada	0.3	1075	2.5	300	Brunisolic Gray Luvisol	Douglas fir	Dfb, Humid Continental warm summer	REF	REF	0	A horizon	20.8	NA	NA	0.0	NA	NA
SL121F	SL121	SBS_BC_	British Columbia	SL	Forest Soil	Whole Community DNA	PCR	Amplicon	pyrotag library	454 GS FLX Titanium	European Nucleotide Archive	https://www.ebi.ac.uk/ena/data/search?query=PRJEB12501	ERS1040146	SAMEA3732997	ERX1298483	ERR1226271	ITS	ITS2	NA	TCCTCCGCTTATTGATATGC	GCATCGATGAAGAACGCAGC	2009-08-14	52.32	-121.92	Canada	0.1	1050	3.8	146-193	Orthic Gray Luvisol	Lodgepole pine, Interior spruce	Dfc, Boreal cool summer	OM1	C0	0	O horizon	63.0	20.7	0.6	5.2	1.6	35.0
SL122F	SL122	SBS_BC_	British Columbia	SL	Forest Soil	Whole Community DNA	PCR	Amplicon	pyrotag library	454 GS FLX Titanium	European Nucleotide Archive	https://www.ebi.ac.uk/ena/data/search?query=PRJEB12501	ERS1040147	SAMEA3732998	ERX1298484	ERR1226272	ITS	ITS2	NA	TCCTCCGCTTATTGATATGC	GCATCGATGAAGAACGCAGC	2009-08-14	52.32	-121.92	Canada	0.1	1050	3.8	146-193	Orthic Gray Luvisol	Lodgepole pine, Interior spruce	Dfc, Boreal cool summer	OM1	C0	0	O horizon	57.0	20.7	0.6	5.2	1.6	35.0
SL123F	SL123	SBS_BC_	British Columbia	SL	Forest Soil	Whole Community DNA	PCR	Amplicon	pyrotag library	454 GS FLX Titanium	European Nucleotide Archive	https://www.ebi.ac.uk/ena/data/search?query=PRJEB12501	ERS1040148	SAMEA3732999	ERX1298485	ERR1226273	ITS	ITS2	NA	TCCTCCGCTTATTGATATGC	GCATCGATGAAGAACGCAGC	2009-08-14	52.32	-121.92	Canada	0.1	1050	3.8	146-193	Orthic Gray Luvisol	Lodgepole pine, Interior spruce	Dfc, Boreal cool summer	OM1	C0	0	O horizon	61.0	20.7	0.6	5.2	1.6	35.0
SL124F	SL124	SBS_BC_	British Columbia	SL	Forest Soil	Whole Community DNA	PCR	Amplicon	pyrotag library	454 GS FLX Titanium	European Nucleotide Archive	https://www.ebi.ac.uk/ena/data/search?query=PRJEB12501	ERS1040149	SAMEA3733000	ERX1298486	ERR1226274	ITS	ITS2	NA	TCCTCCGCTTATTGATATGC	GCATCGATGAAGAACGCAGC	2009-08-14	52.32	-121.92	Canada	0.3	1050	3.8	146-193	Orthic Gray Luvisol	Lodgepole pine, Interior spruce	Dfc, Boreal cool summer	OM1	C0	0	A horizon	30.0	0.9	0.1	5.4	1.6	18.4
SL125F	SL125	SBS_BC_	British Columbia	SL	Forest Soil	Whole Community DNA	PCR	Amplicon	pyrotag library	454 GS FLX Titanium	European Nucleotide Archive	https://www.ebi.ac.uk/ena/data/search?query=PRJEB12501	ERS1040150	SAMEA3733001	ERX1298487	ERR1226275	ITS	ITS2	NA	TCCTCCGCTTATTGATATGC	GCATCGATGAAGAACGCAGC	2009-08-14	52.32	-121.92	Canada	0.3	1050	3.8	146-193	Orthic Gray Luvisol	Lodgepole pine, Interior spruce	Dfc, Boreal cool summer	OM1	C0	0	A horizon	30.0	0.9	0.1	5.4	1.6	18.4
SL126F	SL126	SBS_BC_	British Columbia	SL	Forest Soil	Whole Community DNA	PCR	Amplicon	pyrotag library	454 GS FLX Titanium	European Nucleotide Archive	https://www.ebi.ac.uk/ena/data/search?query=PRJEB12501	ERS1040151	SAMEA3733002	ERX1298488	ERR1226276	ITS	ITS2	NA	TCCTCCGCTTATTGATATGC	GCATCGATGAAGAACGCAGC	2009-08-14	52.32	-121.92	Canada	0.3	1050	3.8	146-193	Orthic Gray Luvisol	Lodgepole pine, Interior spruce	Dfc, Boreal cool summer	OM1	C0	0	A horizon	30.0	0.9	0.1	5.4	1.6	18.4
SL127F	SL127	SBS_BC_	British Columbia	SL	Forest Soil	Whole Community DNA	PCR	Amplicon	pyrotag library	454 GS FLX Titanium	European Nucleotide Archive	https://www.ebi.ac.uk/ena/data/search?query=PRJEB12501	ERS1040152	SAMEA3733003	ERX1298489	ERR1226277	ITS	ITS2	NA	TCCTCCGCTTATTGATATGC	GCATCGATGAAGAACGCAGC	2009-08-14	52.32	-121.92	Canada	0.1	1050	3.8	146-193	Orthic Gray Luvisol	Lodgepole pine, Interior spruce	Dfc, Boreal cool summer	OM1	C1	0	O horizon	60.0	22.3	0.6	5.1	1.6	38.4
SL128F	SL128	SBS_BC_	British Columbia	SL	Forest Soil	Whole Community DNA	PCR	Amplicon	pyrotag library	454 GS FLX Titanium	European Nucleotide Archive	https://www.ebi.ac.uk/ena/data/search?query=PRJEB12501	ERS1040153	SAMEA3733004	ERX1298490	ERR1226278	ITS	ITS2	NA	TCCTCCGCTTATTGATATGC	GCATCGATGAAGAACGCAGC	2009-08-14	52.32	-121.92	Canada	0.1	1050	3.8	146-193	Orthic Gray Luvisol	Lodgepole pine, Interior spruce	Dfc, Boreal cool summer	OM1	C1	0	O horizon	59.0	22.3	0.6	5.1	1.6	38.4
SL129F	SL129	SBS_BC_	British Columbia	SL	Forest Soil	Whole Community DNA	PCR	Amplicon	pyrotag library	454 GS FLX Titanium	European Nucleotide Archive	https://www.ebi.ac.uk/ena/data/search?query=PRJEB12501	ERS1040154	SAMEA3733005	ERX1298491	ERR1226279	ITS	ITS2	NA	TCCTCCGCTTATTGATATGC	GCATCGATGAAGAACGCAGC	2009-08-14	52.32	-121.92	Canada	0.1	1050	3.8	146-193	Orthic Gray Luvisol	Lodgepole pine, Interior spruce	Dfc, Boreal cool summer	OM1	C1	0	O horizon	62.0	22.3	0.6	5.1	1.6	38.4
SL130F	SL130	SBS_BC_	British Columbia	SL	Forest Soil	Whole Community DNA	PCR	Amplicon	pyrotag library	454 GS FLX Titanium	European Nucleotide Archive	https://www.ebi.ac.uk/ena/data/search?query=PRJEB12501	ERS1040155	SAMEA3733006	ERX1298492	ERR1226280	ITS	ITS2	NA	TCCTCCGCTTATTGATATGC	GCATCGATGAAGAACGCAGC	2009-08-14	52.32	-121.92	Canada	0.3	1050	3.8	146-193	Orthic Gray Luvisol	Lodgepole pine, Interior spruce	Dfc, Boreal cool summer	OM1	C1	0	A horizon	32.0	1.0	0.1	5.5	1.6	19.4
SL131F	SL131	SBS_BC_	British Columbia	SL	Forest Soil	Whole Community DNA	PCR	Amplicon	pyrotag library	454 GS FLX Titanium	European Nucleotide Archive	https://www.ebi.ac.uk/ena/data/search?query=PRJEB12501	ERS1040156	SAMEA3733007	ERX1298493	ERR1226281	ITS	ITS2	NA	TCCTCCGCTTATTGATATGC	GCATCGATGAAGAACGCAGC	2009-08-14	52.32	-121.92	Canada	0.3	1050	3.8	146-193	Orthic Gray Luvisol	Lodgepole pine, Interior spruce	Dfc, Boreal cool summer	OM1	C1	0	A horizon	32.0	1.0	0.1	5.5	1.6	19.4
SL132F	SL132	SBS_BC_	British Columbia	SL	Forest Soil	Whole Community DNA	PCR	Amplicon	pyrotag library	454 GS FLX Titanium	European Nucleotide Archive	https://www.ebi.ac.uk/ena/data/search?query=PRJEB12501	ERS1040157	SAMEA3733008	ERX1298494	ERR1226282	ITS	ITS2	NA	TCCTCCGCTTATTGATATGC	GCATCGATGAAGAACGCAGC	2009-08-14	52.32	-121.92	Canada	0.3	1050	3.8	146-193	Orthic Gray Luvisol	Lodgepole pine, Interior spruce	Dfc, Boreal cool summer	OM1	C1	0	A horizon	36.0	1.0	0.1	5.5	1.6	19.4
SL133F	SL133	SBS_BC_	British Columbia	SL	Forest Soil	Whole Community DNA	PCR	Amplicon	pyrotag library	454 GS FLX Titanium	European Nucleotide Archive	https://www.ebi.ac.uk/ena/data/search?query=PRJEB12501	ERS1040158	SAMEA3733009	ERX1298495	ERR1226283	ITS	ITS2	NA	TCCTCCGCTTATTGATATGC	GCATCGATGAAGAACGCAGC	2009-08-14	52.32	-121.92	Canada	0.1	1050	3.8	146-193	Orthic Gray Luvisol	Lodgepole pine, Interior spruce	Dfc, Boreal cool summer	OM1	C2	0	O horizon	50.0	18.8	0.5	5.3	1.8	36.2
SL134F	SL134	SBS_BC_	British Columbia	SL	Forest Soil	Whole Community DNA	PCR	Amplicon	pyrotag library	454 GS FLX Titanium	European Nucleotide Archive	https://www.ebi.ac.uk/ena/data/search?query=PRJEB12501	ERS1040159	SAMEA3733010	ERX1298496	ERR1226284	ITS	ITS2	NA	TCCTCCGCTTATTGATATGC	GCATCGATGAAGAACGCAGC	2009-08-14	52.32	-121.92	Canada	0.1	1050	3.8	146-193	Orthic Gray Luvisol	Lodgepole pine, Interior spruce	Dfc, Boreal cool summer	OM1	C2	0	O horizon	68.0	18.8	0.5	5.3	1.8	36.2
SL135F	SL135	SBS_BC_	British Columbia	SL	Forest Soil	Whole Community DNA	PCR	Amplicon	pyrotag library	454 GS FLX Titanium	European Nucleotide Archive	https://www.ebi.ac.uk/ena/data/search?query=PRJEB12501	ERS1040160	SAMEA3733011	ERX1298497	ERR1226285	ITS	ITS2	NA	TCCTCCGCTTATTGATATGC	GCATCGATGAAGAACGCAGC	2009-08-14	52.32	-121.92	Canada	0.1	1050	3.8	146-193	Orthic Gray Luvisol	Lodgepole pine, Interior spruce	Dfc, Boreal cool summer	OM1	C2	0	O horizon	67.0	18.8	0.5	5.3	1.8	36.2
SL136F	SL136	SBS_BC_	British Columbia	SL	Forest Soil	Whole Community DNA	PCR	Amplicon	pyrotag library	454 GS FLX Titanium	European Nucleotide Archive	https://www.ebi.ac.uk/ena/data/search?query=PRJEB12501	ERS1040161	SAMEA3733012	ERX1298498	ERR1226286	ITS	ITS2	NA	TCCTCCGCTTATTGATATGC	GCATCGATGAAGAACGCAGC	2009-08-14	52.32	-121.92	Canada	0.3	1050	3.8	146-193	Orthic Gray Luvisol	Lodgepole pine, Interior spruce	Dfc, Boreal cool summer	OM1	C2	0	A horizon	31.0	0.8	0.1	5.6	1.8	16.2
SL137F	SL137	SBS_BC_	British Columbia	SL	Forest Soil	Whole Community DNA	PCR	Amplicon	pyrotag library	454 GS FLX Titanium	European Nucleotide Archive	https://www.ebi.ac.uk/ena/data/search?query=PRJEB12501	ERS1040162	SAMEA3733013	ERX1298499	ERR1226287	ITS	ITS2	NA	TCCTCCGCTTATTGATATGC	GCATCGATGAAGAACGCAGC	2009-08-14	52.32	-121.92	Canada	0.3	1050	3.8	146-193	Orthic Gray Luvisol	Lodgepole pine, Interior spruce	Dfc, Boreal cool summer	OM1	C2	0	A horizon	33.0	0.8	0.1	5.6	1.8	16.2
SL138F	SL138	SBS_BC_	British Columbia	SL	Forest Soil	Whole Community DNA	PCR	Amplicon	pyrotag library	454 GS FLX Titanium	European Nucleotide Archive	https://www.ebi.ac.uk/ena/data/search?query=PRJEB12501	ERS1040163	SAMEA3733014	ERX1298500	ERR1226288	ITS	ITS2	NA	TCCTCCGCTTATTGATATGC	GCATCGATGAAGAACGCAGC	2009-08-14	52.32	-121.92	Canada	0.3	1050	3.8	146-193	Orthic Gray Luvisol	Lodgepole pine, Interior spruce	Dfc, Boreal cool summer	OM1	C2	0	A horizon	32.0	0.8	0.1	5.6	1.8	16.2
SL139F	SL139	SBS_BC_	British Columbia	SL	Forest Soil	Whole Community DNA	PCR	Amplicon	pyrotag library	454 GS FLX Titanium	European Nucleotide Archive	https://www.ebi.ac.uk/ena/data/search?query=PRJEB12501	ERS1040164	SAMEA3733015	ERX1298501	ERR1226289	ITS	ITS2	NA	TCCTCCGCTTATTGATATGC	GCATCGATGAAGAACGCAGC	2009-08-14	52.32	-121.92	Canada	0.1	1050	3.8	146-193	Orthic Gray Luvisol	Lodgepole pine, Interior spruce	Dfc, Boreal cool summer	OM2	C0	0	O horizon	56.0	17.5	0.5	5.2	1.7	34.3
SL140F	SL140	SBS_BC_	British Columbia	SL	Forest Soil	Whole Community DNA	PCR	Amplicon	pyrotag library	454 GS FLX Titanium	European Nucleotide Archive	https://www.ebi.ac.uk/ena/data/search?query=PRJEB12501	ERS1040165	SAMEA3733016	ERX1298502	ERR1226290	ITS	ITS2	NA	TCCTCCGCTTATTGATATGC	GCATCGATGAAGAACGCAGC	2009-08-14	52.32	-121.92	Canada	0.1	1050	3.8	146-193	Orthic Gray Luvisol	Lodgepole pine, Interior spruce	Dfc, Boreal cool summer	OM2	C0	0	O horizon	57.0	17.5	0.5	5.2	1.7	34.3
SL141F	SL141	SBS_BC_	British Columbia	SL	Forest Soil	Whole Community DNA	PCR	Amplicon	pyrotag library	454 GS FLX Titanium	European Nucleotide Archive	https://www.ebi.ac.uk/ena/data/search?query=PRJEB12501	ERS1040166	SAMEA3733017	ERX1298503	ERR1226291	ITS	ITS2	NA	TCCTCCGCTTATTGATATGC	GCATCGATGAAGAACGCAGC	2009-08-14	52.32	-121.92	Canada	0.1	1050	3.8	146-193	Orthic Gray Luvisol	Lodgepole pine, Interior spruce	Dfc, Boreal cool summer	OM2	C0	0	O horizon	58.0	17.5	0.5	5.2	1.7	34.3
SL142F	SL142	SBS_BC_	British Columbia	SL	Forest Soil	Whole Community DNA	PCR	Amplicon	pyrotag library	454 GS FLX Titanium	European Nucleotide Archive	https://www.ebi.ac.uk/ena/data/search?query=PRJEB12501	ERS1040167	SAMEA3733018	ERX1298504	ERR1226292	ITS	ITS2	NA	TCCTCCGCTTATTGATATGC	GCATCGATGAAGAACGCAGC	2009-08-14	52.32	-121.92	Canada	0.3	1050	3.8	146-193	Orthic Gray Luvisol	Lodgepole pine, Interior spruce	Dfc, Boreal cool summer	OM2	C0	0	A horizon	33.0	0.9	0.1	5.5	1.7	18.2
SL143F	SL143	SBS_BC_	British Columbia	SL	Forest Soil	Whole Community DNA	PCR	Amplicon	pyrotag library	454 GS FLX Titanium	European Nucleotide Archive	https://www.ebi.ac.uk/ena/data/search?query=PRJEB12501	ERS1040168	SAMEA3733019	ERX1298505	ERR1226293	ITS	ITS2	NA	TCCTCCGCTTATTGATATGC	GCATCGATGAAGAACGCAGC	2009-08-14	52.32	-121.92	Canada	0.3	1050	3.8	146-193	Orthic Gray Luvisol	Lodgepole pine, Interior spruce	Dfc, Boreal cool summer	OM2	C0	0	A horizon	35.0	0.9	0.1	5.5	1.7	18.2
SL144F	SL144	SBS_BC_	British Columbia	SL	Forest Soil	Whole Community DNA	PCR	Amplicon	pyrotag library	454 GS FLX Titanium	European Nucleotide Archive	https://www.ebi.ac.uk/ena/data/search?query=PRJEB12501	ERS1040169	SAMEA3733020	ERX1298506	ERR1226294	ITS	ITS2	NA	TCCTCCGCTTATTGATATGC	GCATCGATGAAGAACGCAGC	2009-08-14	52.32	-121.92	Canada	0.3	1050	3.8	146-193	Orthic Gray Luvisol	Lodgepole pine, Interior spruce	Dfc, Boreal cool summer	OM2	C0	0	A horizon	28.0	0.9	0.1	5.5	1.7	18.2
SL145F	SL145	SBS_BC_	British Columbia	SL	Forest Soil	Whole Community DNA	PCR	Amplicon	pyrotag library	454 GS FLX Titanium	European Nucleotide Archive	https://www.ebi.ac.uk/ena/data/search?query=PRJEB12501	ERS1040170	SAMEA3733021	ERX1298507	ERR1226295	ITS	ITS2	NA	TCCTCCGCTTATTGATATGC	GCATCGATGAAGAACGCAGC	2009-08-14	52.32	-121.92	Canada	0.1	1050	3.8	146-193	Orthic Gray Luvisol	Lodgepole pine, Interior spruce	Dfc, Boreal cool summer	OM2	C1	0	O horizon	55.0	19.9	0.5	5.4	1.7	39.0
SL146F	SL146	SBS_BC_	British Columbia	SL	Forest Soil	Whole Community DNA	PCR	Amplicon	pyrotag library	454 GS FLX Titanium	European Nucleotide Archive	https://www.ebi.ac.uk/ena/data/search?query=PRJEB12501	ERS1040171	SAMEA3733022	ERX1298508	ERR1226296	ITS	ITS2	NA	TCCTCCGCTTATTGATATGC	GCATCGATGAAGAACGCAGC	2009-08-14	52.32	-121.92	Canada	0.1	1050	3.8	146-193	Orthic Gray Luvisol	Lodgepole pine, Interior spruce	Dfc, Boreal cool summer	OM2	C1	0	O horizon	54.0	19.9	0.5	5.4	1.7	39.0
SL147F	SL147	SBS_BC_	British Columbia	SL	Forest Soil	Whole Community DNA	PCR	Amplicon	pyrotag library	454 GS FLX Titanium	European Nucleotide Archive	https://www.ebi.ac.uk/ena/data/search?query=PRJEB12501	ERS1040172	SAMEA3733023	ERX1298509	ERR1226297	ITS	ITS2	NA	TCCTCCGCTTATTGATATGC	GCATCGATGAAGAACGCAGC	2009-08-14	52.32	-121.92	Canada	0.1	1050	3.8	146-193	Orthic Gray Luvisol	Lodgepole pine, Interior spruce	Dfc, Boreal cool summer	OM2	C1	0	O horizon	62.0	19.9	0.5	5.4	1.7	39.0
SL148F	SL148	SBS_BC_	British Columbia	SL	Forest Soil	Whole Community DNA	PCR	Amplicon	pyrotag library	454 GS FLX Titanium	European Nucleotide Archive	https://www.ebi.ac.uk/ena/data/search?query=PRJEB12501	ERS1040173	SAMEA3733024	ERX1298510	ERR1226298	ITS	ITS2	NA	TCCTCCGCTTATTGATATGC	GCATCGATGAAGAACGCAGC	2009-08-14	52.32	-121.92	Canada	0.3	1050	3.8	146-193	Orthic Gray Luvisol	Lodgepole pine, Interior spruce	Dfc, Boreal cool summer	OM2	C1	0	A horizon	33.0	1.4	0.1	5.6	1.7	19.6
SL149F	SL149	SBS_BC_	British Columbia	SL	Forest Soil	Whole Community DNA	PCR	Amplicon	pyrotag library	454 GS FLX Titanium	European Nucleotide Archive	https://www.ebi.ac.uk/ena/data/search?query=PRJEB12501	ERS1040174	SAMEA3733025	ERX1298511	ERR1226299	ITS	ITS2	NA	TCCTCCGCTTATTGATATGC	GCATCGATGAAGAACGCAGC	2009-08-14	52.32	-121.92	Canada	0.3	1050	3.8	146-193	Orthic Gray Luvisol	Lodgepole pine, Interior spruce	Dfc, Boreal cool summer	OM2	C1	0	A horizon	32.0	1.4	0.1	5.6	1.7	19.6
SL150F	SL150	SBS_BC_	British Columbia	SL	Forest Soil	Whole Community DNA	PCR	Amplicon	pyrotag library	454 GS FLX Titanium	European Nucleotide Archive	https://www.ebi.ac.uk/ena/data/search?query=PRJEB12501	ERS1040175	SAMEA3733026	ERX1298512	ERR1226300	ITS	ITS2	NA	TCCTCCGCTTATTGATATGC	GCATCGATGAAGAACGCAGC	2009-08-14	52.32	-121.92	Canada	0.3	1050	3.8	146-193	Orthic Gray Luvisol	Lodgepole pine, Interior spruce	Dfc, Boreal cool summer	OM2	C1	0	A horizon	31.0	1.4	0.1	5.6	1.7	19.6
SL151F	SL151	SBS_BC_	British Columbia	SL	Forest Soil	Whole Community DNA	PCR	Amplicon	pyrotag library	454 GS FLX Titanium	European Nucleotide Archive	https://www.ebi.ac.uk/ena/data/search?query=PRJEB12501	ERS1040176	SAMEA3733027	ERX1298513	ERR1226301	ITS	ITS2	NA	TCCTCCGCTTATTGATATGC	GCATCGATGAAGAACGCAGC	2009-08-14	52.32	-121.92	Canada	0.1	1050	3.8	146-193	Orthic Gray Luvisol	Lodgepole pine, Interior spruce	Dfc, Boreal cool summer	OM2	C2	0	O horizon	51.0	17.9	0.5	5.2	1.7	33.2
SL152F	SL152	SBS_BC_	British Columbia	SL	Forest Soil	Whole Community DNA	PCR	Amplicon	pyrotag library	454 GS FLX Titanium	European Nucleotide Archive	https://www.ebi.ac.uk/ena/data/search?query=PRJEB12501	ERS1040177	SAMEA3733028	ERX1298514	ERR1226302	ITS	ITS2	NA	TCCTCCGCTTATTGATATGC	GCATCGATGAAGAACGCAGC	2009-08-14	52.32	-121.92	Canada	0.1	1050	3.8	146-193	Orthic Gray Luvisol	Lodgepole pine, Interior spruce	Dfc, Boreal cool summer	OM2	C2	0	O horizon	54.0	17.9	0.5	5.2	1.7	33.2
SL153F	SL153	SBS_BC_	British Columbia	SL	Forest Soil	Whole Community DNA	PCR	Amplicon	pyrotag library	454 GS FLX Titanium	European Nucleotide Archive	https://www.ebi.ac.uk/ena/data/search?query=PRJEB12501	ERS1040178	SAMEA3733029	ERX1298515	ERR1226303	ITS	ITS2	NA	TCCTCCGCTTATTGATATGC	GCATCGATGAAGAACGCAGC	2009-08-14	52.32	-121.92	Canada	0.1	1050	3.8	146-193	Orthic Gray Luvisol	Lodgepole pine, Interior spruce	Dfc, Boreal cool summer	OM2	C2	0	O horizon	63.0	17.9	0.5	5.2	1.7	33.2
SL154F	SL154	SBS_BC_	British Columbia	SL	Forest Soil	Whole Community DNA	PCR	Amplicon	pyrotag library	454 GS FLX Titanium	European Nucleotide Archive	https://www.ebi.ac.uk/ena/data/search?query=PRJEB12501	ERS1040179	SAMEA3733030	ERX1298516	ERR1226304	ITS	ITS2	NA	TCCTCCGCTTATTGATATGC	GCATCGATGAAGAACGCAGC	2009-08-14	52.32	-121.92	Canada	0.3	1050	3.8	146-193	Orthic Gray Luvisol	Lodgepole pine, Interior spruce	Dfc, Boreal cool summer	OM2	C2	0	A horizon	28.0	1.1	0.1	5.5	1.7	17.8
SL155F	SL155	SBS_BC_	British Columbia	SL	Forest Soil	Whole Community DNA	PCR	Amplicon	pyrotag library	454 GS FLX Titanium	European Nucleotide Archive	https://www.ebi.ac.uk/ena/data/search?query=PRJEB12501	ERS1040180	SAMEA3733031	ERX1298517	ERR1226305	ITS	ITS2	NA	TCCTCCGCTTATTGATATGC	GCATCGATGAAGAACGCAGC	2009-08-14	52.32	-121.92	Canada	0.3	1050	3.8	146-193	Orthic Gray Luvisol	Lodgepole pine, Interior spruce	Dfc, Boreal cool summer	OM2	C2	0	A horizon	34.0	1.1	0.1	5.5	1.7	17.8
SL156F	SL156	SBS_BC_	British Columbia	SL	Forest Soil	Whole Community DNA	PCR	Amplicon	pyrotag library	454 GS FLX Titanium	European Nucleotide Archive	https://www.ebi.ac.uk/ena/data/search?query=PRJEB12501	ERS1040181	SAMEA3733032	ERX1298518	ERR1226306	ITS	ITS2	NA	TCCTCCGCTTATTGATATGC	GCATCGATGAAGAACGCAGC	2009-08-14	52.32	-121.92	Canada	0.3	1050	3.8	146-193	Orthic Gray Luvisol	Lodgepole pine, Interior spruce	Dfc, Boreal cool summer	OM2	C2	0	A horizon	32.0	1.1	0.1	5.5	1.7	17.8
SL160F	SL160	SBS_BC_	British Columbia	SL	Forest Soil	Whole Community DNA	PCR	Amplicon	pyrotag library	454 GS FLX Titanium	European Nucleotide Archive	https://www.ebi.ac.uk/ena/data/search?query=PRJEB12501	ERS1040182	SAMEA3733033	ERX1298519	ERR1226307	ITS	ITS2	NA	TCCTCCGCTTATTGATATGC	GCATCGATGAAGAACGCAGC	2009-08-14	52.32	-121.92	Canada	0.3	1050	3.8	146-193	Orthic Gray Luvisol	Lodgepole pine, Interior spruce	Dfc, Boreal cool summer	OM3	C0	0	A horizon	33.0	0.9	0.1	5.6	1.7	18.2
SL162F	SL162	SBS_BC_	British Columbia	SL	Forest Soil	Whole Community DNA	PCR	Amplicon	pyrotag library	454 GS FLX Titanium	European Nucleotide Archive	https://www.ebi.ac.uk/ena/data/search?query=PRJEB12501	ERS1040184	SAMEA3733035	ERX1298521	ERR1226309	ITS	ITS2	NA	TCCTCCGCTTATTGATATGC	GCATCGATGAAGAACGCAGC	2009-08-14	52.32	-121.92	Canada	0.3	1050	3.8	146-193	Orthic Gray Luvisol	Lodgepole pine, Interior spruce	Dfc, Boreal cool summer	OM3	C0	0	A horizon	33.0	0.9	0.1	5.6	1.7	18.2
SL166F	SL166	SBS_BC_	British Columbia	SL	Forest Soil	Whole Community DNA	PCR	Amplicon	pyrotag library	454 GS FLX Titanium	European Nucleotide Archive	https://www.ebi.ac.uk/ena/data/search?query=PRJEB12501	ERS1040185	SAMEA3733036	ERX1298522	ERR1226310	ITS	ITS2	NA	TCCTCCGCTTATTGATATGC	GCATCGATGAAGAACGCAGC	2009-08-14	52.32	-121.92	Canada	0.3	1050	3.8	146-193	Orthic Gray Luvisol	Lodgepole pine, Interior spruce	Dfc, Boreal cool summer	OM3	C1	0	A horizon	32.0	1.1	0.1	5.5	1.7	18.2
SL167F	SL167	SBS_BC_	British Columbia	SL	Forest Soil	Whole Community DNA	PCR	Amplicon	pyrotag library	454 GS FLX Titanium	European Nucleotide Archive	https://www.ebi.ac.uk/ena/data/search?query=PRJEB12501	ERS1040186	SAMEA3733037	ERX1298523	ERR1226311	ITS	ITS2	NA	TCCTCCGCTTATTGATATGC	GCATCGATGAAGAACGCAGC	2009-08-14	52.32	-121.92	Canada	0.3	1050	3.8	146-193	Orthic Gray Luvisol	Lodgepole pine, Interior spruce	Dfc, Boreal cool summer	OM3	C1	0	A horizon	32.0	1.1	0.1	5.5	1.7	18.2
SL168F	SL168	SBS_BC_	British Columbia	SL	Forest Soil	Whole Community DNA	PCR	Amplicon	pyrotag library	454 GS FLX Titanium	European Nucleotide Archive	https://www.ebi.ac.uk/ena/data/search?query=PRJEB12501	ERS1040187	SAMEA3733038	ERX1298524	ERR1226312	ITS	ITS2	NA	TCCTCCGCTTATTGATATGC	GCATCGATGAAGAACGCAGC	2009-08-14	52.32	-121.92	Canada	0.3	1050	3.8	146-193	Orthic Gray Luvisol	Lodgepole pine, Interior spruce	Dfc, Boreal cool summer	OM3	C1	0	A horizon	31.0	1.1	0.1	5.5	1.7	18.2
SL172F	SL172	SBS_BC_	British Columbia	SL	Forest Soil	Whole Community DNA	PCR	Amplicon	pyrotag library	454 GS FLX Titanium	European Nucleotide Archive	https://www.ebi.ac.uk/ena/data/search?query=PRJEB12501	ERS1040188	SAMEA3733039	ERX1298525	ERR1226313	ITS	ITS2	NA	TCCTCCGCTTATTGATATGC	GCATCGATGAAGAACGCAGC	2009-08-14	52.32	-121.92	Canada	0.3	1050	3.8	146-193	Orthic Gray Luvisol	Lodgepole pine, Interior spruce	Dfc, Boreal cool summer	OM3	C2	0	A horizon	32.0	0.9	0.1	5.7	1.7	18.0
SL173F	SL173	SBS_BC_	British Columbia	SL	Forest Soil	Whole Community DNA	PCR	Amplicon	pyrotag library	454 GS FLX Titanium	European Nucleotide Archive	https://www.ebi.ac.uk/ena/data/search?query=PRJEB12501	ERS1040189	SAMEA3733040	ERX1298526	ERR1226314	ITS	ITS2	NA	TCCTCCGCTTATTGATATGC	GCATCGATGAAGAACGCAGC	2009-08-14	52.32	-121.92	Canada	0.3	1050	3.8	146-193	Orthic Gray Luvisol	Lodgepole pine, Interior spruce	Dfc, Boreal cool summer	OM3	C2	0	A horizon	30.0	0.9	0.1	5.7	1.7	18.0
SL174F	SL174	SBS_BC_	British Columbia	SL	Forest Soil	Whole Community DNA	PCR	Amplicon	pyrotag library	454 GS FLX Titanium	European Nucleotide Archive	https://www.ebi.ac.uk/ena/data/search?query=PRJEB12501	ERS1040190	SAMEA3733041	ERX1298527	ERR1226315	ITS	ITS2	NA	TCCTCCGCTTATTGATATGC	GCATCGATGAAGAACGCAGC	2009-08-14	52.32	-121.92	Canada	0.3	1050	3.8	146-193	Orthic Gray Luvisol	Lodgepole pine, Interior spruce	Dfc, Boreal cool summer	OM3	C2	0	A horizon	32.0	0.9	0.1	5.7	1.7	18.0
SL175F	SL175	SBS_BC_	British Columbia	SL	Forest Soil	Whole Community DNA	PCR	Amplicon	pyrotag library	454 GS FLX Titanium	European Nucleotide Archive	https://www.ebi.ac.uk/ena/data/search?query=PRJEB12501	ERS1040191	SAMEA3733042	ERX1298528	ERR1226316	ITS	ITS2	NA	TCCTCCGCTTATTGATATGC	GCATCGATGAAGAACGCAGC	2009-08-14	52.32	-121.92	Canada	0.1	1050	3.8	146-193	Orthic Gray Luvisol	Lodgepole pine, Interior spruce	Dfc, Boreal cool summer	REF	REF	0	O horizon	85.0	18.9	0.6	5.4	NA	33.7
SL176F	SL176	SBS_BC_	British Columbia	SL	Forest Soil	Whole Community DNA	PCR	Amplicon	pyrotag library	454 GS FLX Titanium	European Nucleotide Archive	https://www.ebi.ac.uk/ena/data/search?query=PRJEB12501	ERS1040192	SAMEA3733043	ERX1298529	ERR1226317	ITS	ITS2	NA	TCCTCCGCTTATTGATATGC	GCATCGATGAAGAACGCAGC	2009-08-14	52.32	-121.92	Canada	0.1	1050	3.8	146-193	Orthic Gray Luvisol	Lodgepole pine, Interior spruce	Dfc, Boreal cool summer	REF	REF	0	O horizon	73.0	18.9	0.6	5.4	NA	33.7
SL177F	SL177	SBS_BC_	British Columbia	SL	Forest Soil	Whole Community DNA	PCR	Amplicon	pyrotag library	454 GS FLX Titanium	European Nucleotide Archive	https://www.ebi.ac.uk/ena/data/search?query=PRJEB12501	ERS1040193	SAMEA3733044	ERX1298530	ERR1226318	ITS	ITS2	NA	TCCTCCGCTTATTGATATGC	GCATCGATGAAGAACGCAGC	2009-08-14	52.32	-121.92	Canada	0.1	1050	3.8	146-193	Orthic Gray Luvisol	Lodgepole pine, Interior spruce	Dfc, Boreal cool summer	REF	REF	0	O horizon	71.0	18.9	0.6	5.4	NA	33.7
SL178F	SL178	SBS_BC_	British Columbia	SL	Forest Soil	Whole Community DNA	PCR	Amplicon	pyrotag library	454 GS FLX Titanium	European Nucleotide Archive	https://www.ebi.ac.uk/ena/data/search?query=PRJEB12501	ERS1040194	SAMEA3733045	ERX1298531	ERR1226319	ITS	ITS2	NA	TCCTCCGCTTATTGATATGC	GCATCGATGAAGAACGCAGC	2009-08-14	52.32	-121.92	Canada	0.3	1050	3.8	146-193	Orthic Gray Luvisol	Lodgepole pine, Interior spruce	Dfc, Boreal cool summer	REF	REF	0	A horizon	36.0	0.9	0.1	5.8	NA	17.4
SL179F	SL179	SBS_BC_	British Columbia	SL	Forest Soil	Whole Community DNA	PCR	Amplicon	pyrotag library	454 GS FLX Titanium	European Nucleotide Archive	https://www.ebi.ac.uk/ena/data/search?query=PRJEB12501	ERS1040195	SAMEA3733046	ERX1298532	ERR1226320	ITS	ITS2	NA	TCCTCCGCTTATTGATATGC	GCATCGATGAAGAACGCAGC	2009-08-14	52.32	-121.92	Canada	0.3	1050	3.8	146-193	Orthic Gray Luvisol	Lodgepole pine, Interior spruce	Dfc, Boreal cool summer	REF	REF	0	A horizon	36.0	0.9	0.1	5.8	NA	17.4
SL180F	SL180	SBS_BC_	British Columbia	SL	Forest Soil	Whole Community DNA	PCR	Amplicon	pyrotag library	454 GS FLX Titanium	European Nucleotide Archive	https://www.ebi.ac.uk/ena/data/search?query=PRJEB12501	ERS1040196	SAMEA3733047	ERX1298533	ERR1226321	ITS	ITS2	NA	TCCTCCGCTTATTGATATGC	GCATCGATGAAGAACGCAGC	2009-08-14	52.32	-121.92	Canada	0.3	1050	3.8	146-193	Orthic Gray Luvisol	Lodgepole pine, Interior spruce	Dfc, Boreal cool summer	REF	REF	0	A horizon	34.0	0.9	0.1	5.8	NA	17.4
TO061F	TO061	SBS_BC_	British Columbia	TO	Forest Soil	Whole Community DNA	PCR	Amplicon	pyrotag library	454 GS FLX Titanium	European Nucleotide Archive	https://www.ebi.ac.uk/ena/data/search?query=PRJEB12501	ERS1040197	SAMEA3733048	ERX1298534	ERR1226322	ITS	ITS2	NA	TCCTCCGCTTATTGATATGC	GCATCGATGAAGAACGCAGC	2008-07-11	52.32	-126.31	Canada	0.1	1100	1.7	146-193	Orthic Gray Luvisol, Gleyed Gray Luvisol	Lodgepole pine, Subalpine fir, Interior spruce	Dfc, Boreal cool summer	OM1	C0	0	O horizon	58.0	33.5	1.0	5.1	1.5	32.5
TO062F	TO062	SBS_BC_	British Columbia	TO	Forest Soil	Whole Community DNA	PCR	Amplicon	pyrotag library	454 GS FLX Titanium	European Nucleotide Archive	https://www.ebi.ac.uk/ena/data/search?query=PRJEB12501	ERS1040198	SAMEA3733049	ERX1298535	ERR1226323	ITS	ITS2	NA	TCCTCCGCTTATTGATATGC	GCATCGATGAAGAACGCAGC	2008-07-11	52.32	-126.31	Canada	0.1	1100	1.7	146-193	Orthic Gray Luvisol, Gleyed Gray Luvisol	Lodgepole pine, Subalpine fir, Interior spruce	Dfc, Boreal cool summer	OM1	C0	0	O horizon	52.0	33.5	1.0	5.1	1.5	32.5
TO063F	TO063	SBS_BC_	British Columbia	TO	Forest Soil	Whole Community DNA	PCR	Amplicon	pyrotag library	454 GS FLX Titanium	European Nucleotide Archive	https://www.ebi.ac.uk/ena/data/search?query=PRJEB12501	ERS1040199	SAMEA3733050	ERX1298536	ERR1226324	ITS	ITS2	NA	TCCTCCGCTTATTGATATGC	GCATCGATGAAGAACGCAGC	2008-07-11	52.32	-126.31	Canada	0.1	1100	1.7	146-193	Orthic Gray Luvisol, Gleyed Gray Luvisol	Lodgepole pine, Subalpine fir, Interior spruce	Dfc, Boreal cool summer	OM1	C0	0	O horizon	56.0	33.5	1.0	5.1	1.5	32.5
TO064F	TO064	SBS_BC_	British Columbia	TO	Forest Soil	Whole Community DNA	PCR	Amplicon	pyrotag library	454 GS FLX Titanium	European Nucleotide Archive	https://www.ebi.ac.uk/ena/data/search?query=PRJEB12501	ERS1040200	SAMEA3733051	ERX1298537	ERR1226325	ITS	ITS2	NA	TCCTCCGCTTATTGATATGC	GCATCGATGAAGAACGCAGC	2008-07-11	52.32	-126.31	Canada	0.3	1100	1.7	146-193	Orthic Gray Luvisol, Gleyed Gray Luvisol	Lodgepole pine, Subalpine fir, Interior spruce	Dfc, Boreal cool summer	OM1	C0	0	A horizon	20.0	2.2	0.1	5.7	1.5	21.6
TO065F	TO065	SBS_BC_	British Columbia	TO	Forest Soil	Whole Community DNA	PCR	Amplicon	pyrotag library	454 GS FLX Titanium	European Nucleotide Archive	https://www.ebi.ac.uk/ena/data/search?query=PRJEB12501	ERS1040201	SAMEA3733052	ERX1298538	ERR1226326	ITS	ITS2	NA	TCCTCCGCTTATTGATATGC	GCATCGATGAAGAACGCAGC	2008-07-11	52.32	-126.31	Canada	0.3	1100	1.7	146-193	Orthic Gray Luvisol, Gleyed Gray Luvisol	Lodgepole pine, Subalpine fir, Interior spruce	Dfc, Boreal cool summer	OM1	C0	0	A horizon	17.0	2.2	0.1	5.7	1.5	21.6
TO066F	TO066	SBS_BC_	British Columbia	TO	Forest Soil	Whole Community DNA	PCR	Amplicon	pyrotag library	454 GS FLX Titanium	European Nucleotide Archive	https://www.ebi.ac.uk/ena/data/search?query=PRJEB12501	ERS1040202	SAMEA3733053	ERX1298539	ERR1226327	ITS	ITS2	NA	TCCTCCGCTTATTGATATGC	GCATCGATGAAGAACGCAGC	2008-07-11	52.32	-126.31	Canada	0.3	1100	1.7	146-193	Orthic Gray Luvisol, Gleyed Gray Luvisol	Lodgepole pine, Subalpine fir, Interior spruce	Dfc, Boreal cool summer	OM1	C0	0	A horizon	15.0	2.2	0.1	5.7	1.5	21.6
TO067F	TO067	SBS_BC_	British Columbia	TO	Forest Soil	Whole Community DNA	PCR	Amplicon	pyrotag library	454 GS FLX Titanium	European Nucleotide Archive	https://www.ebi.ac.uk/ena/data/search?query=PRJEB12501	ERS1040203	SAMEA3733054	ERX1298540	ERR1226328	ITS	ITS2	NA	TCCTCCGCTTATTGATATGC	GCATCGATGAAGAACGCAGC	2008-07-11	52.32	-126.31	Canada	0.1	1100	1.7	146-193	Orthic Gray Luvisol, Gleyed Gray Luvisol	Lodgepole pine, Subalpine fir, Interior spruce	Dfc, Boreal cool summer	OM2	C0	0	O horizon	58.0	26.4	0.9	4.8	1.5	28.7
TO068F	TO068	SBS_BC_	British Columbia	TO	Forest Soil	Whole Community DNA	PCR	Amplicon	pyrotag library	454 GS FLX Titanium	European Nucleotide Archive	https://www.ebi.ac.uk/ena/data/search?query=PRJEB12501	ERS1040204	SAMEA3733055	ERX1298541	ERR1226329	ITS	ITS2	NA	TCCTCCGCTTATTGATATGC	GCATCGATGAAGAACGCAGC	2008-07-11	52.32	-126.31	Canada	0.1	1100	1.7	146-193	Orthic Gray Luvisol, Gleyed Gray Luvisol	Lodgepole pine, Subalpine fir, Interior spruce	Dfc, Boreal cool summer	OM2	C0	0	O horizon	47.0	26.4	0.9	4.8	1.5	28.7
TO069F	TO069	SBS_BC_	British Columbia	TO	Forest Soil	Whole Community DNA	PCR	Amplicon	pyrotag library	454 GS FLX Titanium	European Nucleotide Archive	https://www.ebi.ac.uk/ena/data/search?query=PRJEB12501	ERS1040205	SAMEA3733056	ERX1298542	ERR1226330	ITS	ITS2	NA	TCCTCCGCTTATTGATATGC	GCATCGATGAAGAACGCAGC	2008-07-11	52.32	-126.31	Canada	0.1	1100	1.7	146-193	Orthic Gray Luvisol, Gleyed Gray Luvisol	Lodgepole pine, Subalpine fir, Interior spruce	Dfc, Boreal cool summer	OM2	C0	0	O horizon	43.0	26.4	0.9	4.8	1.5	28.7
TO070F	TO070	SBS_BC_	British Columbia	TO	Forest Soil	Whole Community DNA	PCR	Amplicon	pyrotag library	454 GS FLX Titanium	European Nucleotide Archive	https://www.ebi.ac.uk/ena/data/search?query=PRJEB12501	ERS1040206	SAMEA3733057	ERX1298543	ERR1226331	ITS	ITS2	NA	TCCTCCGCTTATTGATATGC	GCATCGATGAAGAACGCAGC	2008-07-11	52.32	-126.31	Canada	0.3	1100	1.7	146-193	Orthic Gray Luvisol, Gleyed Gray Luvisol	Lodgepole pine, Subalpine fir, Interior spruce	Dfc, Boreal cool summer	OM2	C0	0	A horizon	25.0	3.3	0.2	5.0	1.5	20.3
TO071F	TO071	SBS_BC_	British Columbia	TO	Forest Soil	Whole Community DNA	PCR	Amplicon	pyrotag library	454 GS FLX Titanium	European Nucleotide Archive	https://www.ebi.ac.uk/ena/data/search?query=PRJEB12501	ERS1040207	SAMEA3733058	ERX1298544	ERR1226332	ITS	ITS2	NA	TCCTCCGCTTATTGATATGC	GCATCGATGAAGAACGCAGC	2008-07-11	52.32	-126.31	Canada	0.3	1100	1.7	146-193	Orthic Gray Luvisol, Gleyed Gray Luvisol	Lodgepole pine, Subalpine fir, Interior spruce	Dfc, Boreal cool summer	OM2	C0	0	A horizon	19.0	3.3	0.2	5.0	1.5	20.3
TO072F	TO072	SBS_BC_	British Columbia	TO	Forest Soil	Whole Community DNA	PCR	Amplicon	pyrotag library	454 GS FLX Titanium	European Nucleotide Archive	https://www.ebi.ac.uk/ena/data/search?query=PRJEB12501	ERS1040208	SAMEA3733059	ERX1298545	ERR1226333	ITS	ITS2	NA	TCCTCCGCTTATTGATATGC	GCATCGATGAAGAACGCAGC	2008-07-11	52.32	-126.31	Canada	0.3	1100	1.7	146-193	Orthic Gray Luvisol, Gleyed Gray Luvisol	Lodgepole pine, Subalpine fir, Interior spruce	Dfc, Boreal cool summer	OM2	C0	0	A horizon	27.0	3.3	0.2	5.0	1.5	20.3
TO076F	TO076	SBS_BC_	British Columbia	TO	Forest Soil	Whole Community DNA	PCR	Amplicon	pyrotag library	454 GS FLX Titanium	European Nucleotide Archive	https://www.ebi.ac.uk/ena/data/search?query=PRJEB12501	ERS1040209	SAMEA3733060	ERX1298546	ERR1226334	ITS	ITS2	NA	TCCTCCGCTTATTGATATGC	GCATCGATGAAGAACGCAGC	2008-07-11	52.32	-126.31	Canada	0.3	1100	1.7	146-193	Orthic Gray Luvisol, Gleyed Gray Luvisol	Lodgepole pine, Subalpine fir, Interior spruce	Dfc, Boreal cool summer	OM3	C0	0	A horizon	20.0	2.2	0.1	5.0	1.6	24.3
TO077F	TO077	SBS_BC_	British Columbia	TO	Forest Soil	Whole Community DNA	PCR	Amplicon	pyrotag library	454 GS FLX Titanium	European Nucleotide Archive	https://www.ebi.ac.uk/ena/data/search?query=PRJEB12501	ERS1040210	SAMEA3733061	ERX1298547	ERR1226335	ITS	ITS2	NA	TCCTCCGCTTATTGATATGC	GCATCGATGAAGAACGCAGC	2008-07-11	52.32	-126.31	Canada	0.3	1100	1.7	146-193	Orthic Gray Luvisol, Gleyed Gray Luvisol	Lodgepole pine, Subalpine fir, Interior spruce	Dfc, Boreal cool summer	OM3	C0	0	A horizon	19.0	2.2	0.1	5.0	1.6	24.3
TO078F	TO078	SBS_BC_	British Columbia	TO	Forest Soil	Whole Community DNA	PCR	Amplicon	pyrotag library	454 GS FLX Titanium	European Nucleotide Archive	https://www.ebi.ac.uk/ena/data/search?query=PRJEB12501	ERS1040211	SAMEA3733062	ERX1298548	ERR1226336	ITS	ITS2	NA	TCCTCCGCTTATTGATATGC	GCATCGATGAAGAACGCAGC	2008-07-11	52.32	-126.31	Canada	0.3	1100	1.7	146-193	Orthic Gray Luvisol, Gleyed Gray Luvisol	Lodgepole pine, Subalpine fir, Interior spruce	Dfc, Boreal cool summer	OM3	C0	0	A horizon	18.0	2.2	0.1	5.0	1.6	24.3
TO079F	TO079	SBS_BC_	British Columbia	TO	Forest Soil	Whole Community DNA	PCR	Amplicon	pyrotag library	454 GS FLX Titanium	European Nucleotide Archive	https://www.ebi.ac.uk/ena/data/search?query=PRJEB12501	ERS1040212	SAMEA3733063	ERX1298549	ERR1226337	ITS	ITS2	NA	TCCTCCGCTTATTGATATGC	GCATCGATGAAGAACGCAGC	2008-07-11	52.32	-126.31	Canada	0.1	1100	1.7	146-193	Orthic Gray Luvisol, Gleyed Gray Luvisol	Lodgepole pine, Subalpine fir, Interior spruce	Dfc, Boreal cool summer	OM1	C1	0	O horizon	52.0	27.6	0.8	4.7	1.8	34.9
TO080F	TO080	SBS_BC_	British Columbia	TO	Forest Soil	Whole Community DNA	PCR	Amplicon	pyrotag library	454 GS FLX Titanium	European Nucleotide Archive	https://www.ebi.ac.uk/ena/data/search?query=PRJEB12501	ERS1040213	SAMEA3733064	ERX1298550	ERR1226338	ITS	ITS2	NA	TCCTCCGCTTATTGATATGC	GCATCGATGAAGAACGCAGC	2008-07-11	52.32	-126.31	Canada	0.1	1100	1.7	146-193	Orthic Gray Luvisol, Gleyed Gray Luvisol	Lodgepole pine, Subalpine fir, Interior spruce	Dfc, Boreal cool summer	OM1	C1	0	O horizon	66.0	27.6	0.8	4.7	1.8	34.9
TO081F	TO081	SBS_BC_	British Columbia	TO	Forest Soil	Whole Community DNA	PCR	Amplicon	pyrotag library	454 GS FLX Titanium	European Nucleotide Archive	https://www.ebi.ac.uk/ena/data/search?query=PRJEB12501	ERS1040214	SAMEA3733065	ERX1298551	ERR1226339	ITS	ITS2	NA	TCCTCCGCTTATTGATATGC	GCATCGATGAAGAACGCAGC	2008-07-11	52.32	-126.31	Canada	0.1	1100	1.7	146-193	Orthic Gray Luvisol, Gleyed Gray Luvisol	Lodgepole pine, Subalpine fir, Interior spruce	Dfc, Boreal cool summer	OM1	C1	0	O horizon	69.0	27.6	0.8	4.7	1.8	34.9
TO082F	TO082	SBS_BC_	British Columbia	TO	Forest Soil	Whole Community DNA	PCR	Amplicon	pyrotag library	454 GS FLX Titanium	European Nucleotide Archive	https://www.ebi.ac.uk/ena/data/search?query=PRJEB12501	ERS1040215	SAMEA3733066	ERX1298552	ERR1226340	ITS	ITS2	NA	TCCTCCGCTTATTGATATGC	GCATCGATGAAGAACGCAGC	2008-07-11	52.32	-126.31	Canada	0.3	1100	1.7	146-193	Orthic Gray Luvisol, Gleyed Gray Luvisol	Lodgepole pine, Subalpine fir, Interior spruce	Dfc, Boreal cool summer	OM1	C1	0	A horizon	30.0	1.9	0.1	4.9	1.8	21.1
TO083F	TO083	SBS_BC_	British Columbia	TO	Forest Soil	Whole Community DNA	PCR	Amplicon	pyrotag library	454 GS FLX Titanium	European Nucleotide Archive	https://www.ebi.ac.uk/ena/data/search?query=PRJEB12501	ERS1040216	SAMEA3733067	ERX1298553	ERR1226341	ITS	ITS2	NA	TCCTCCGCTTATTGATATGC	GCATCGATGAAGAACGCAGC	2008-07-11	52.32	-126.31	Canada	0.3	1100	1.7	146-193	Orthic Gray Luvisol, Gleyed Gray Luvisol	Lodgepole pine, Subalpine fir, Interior spruce	Dfc, Boreal cool summer	OM1	C1	0	A horizon	25.0	1.9	0.1	4.9	1.8	21.1
TO084F	TO084	SBS_BC_	British Columbia	TO	Forest Soil	Whole Community DNA	PCR	Amplicon	pyrotag library	454 GS FLX Titanium	European Nucleotide Archive	https://www.ebi.ac.uk/ena/data/search?query=PRJEB12501	ERS1040217	SAMEA3733068	ERX1298554	ERR1226342	ITS	ITS2	NA	TCCTCCGCTTATTGATATGC	GCATCGATGAAGAACGCAGC	2008-07-11	52.32	-126.31	Canada	0.3	1100	1.7	146-193	Orthic Gray Luvisol, Gleyed Gray Luvisol	Lodgepole pine, Subalpine fir, Interior spruce	Dfc, Boreal cool summer	OM1	C1	0	A horizon	25.0	1.9	0.1	4.9	1.8	21.1
TO085F	TO085	SBS_BC_	British Columbia	TO	Forest Soil	Whole Community DNA	PCR	Amplicon	pyrotag library	454 GS FLX Titanium	European Nucleotide Archive	https://www.ebi.ac.uk/ena/data/search?query=PRJEB12501	ERS1040218	SAMEA3733069	ERX1298555	ERR1226343	ITS	ITS2	NA	TCCTCCGCTTATTGATATGC	GCATCGATGAAGAACGCAGC	2008-07-11	52.32	-126.31	Canada	0.1	1100	1.7	146-193	Orthic Gray Luvisol, Gleyed Gray Luvisol	Lodgepole pine, Subalpine fir, Interior spruce	Dfc, Boreal cool summer	OM2	C1	0	O horizon	68.0	37.1	1.2	4.9	1.7	32.0
TO086F	TO086	SBS_BC_	British Columbia	TO	Forest Soil	Whole Community DNA	PCR	Amplicon	pyrotag library	454 GS FLX Titanium	European Nucleotide Archive	https://www.ebi.ac.uk/ena/data/search?query=PRJEB12501	ERS1040219	SAMEA3733070	ERX1298556	ERR1226344	ITS	ITS2	NA	TCCTCCGCTTATTGATATGC	GCATCGATGAAGAACGCAGC	2008-07-11	52.32	-126.31	Canada	0.1	1100	1.7	146-193	Orthic Gray Luvisol, Gleyed Gray Luvisol	Lodgepole pine, Subalpine fir, Interior spruce	Dfc, Boreal cool summer	OM2	C1	0	O horizon	68.0	37.1	1.2	4.9	1.7	32.0
TO087F	TO087	SBS_BC_	British Columbia	TO	Forest Soil	Whole Community DNA	PCR	Amplicon	pyrotag library	454 GS FLX Titanium	European Nucleotide Archive	https://www.ebi.ac.uk/ena/data/search?query=PRJEB12501	ERS1040220	SAMEA3733071	ERX1298557	ERR1226345	ITS	ITS2	NA	TCCTCCGCTTATTGATATGC	GCATCGATGAAGAACGCAGC	2008-07-11	52.32	-126.31	Canada	0.1	1100	1.7	146-193	Orthic Gray Luvisol, Gleyed Gray Luvisol	Lodgepole pine, Subalpine fir, Interior spruce	Dfc, Boreal cool summer	OM2	C1	0	O horizon	56.0	37.1	1.2	4.9	1.7	32.0
TO088F	TO088	SBS_BC_	British Columbia	TO	Forest Soil	Whole Community DNA	PCR	Amplicon	pyrotag library	454 GS FLX Titanium	European Nucleotide Archive	https://www.ebi.ac.uk/ena/data/search?query=PRJEB12501	ERS1040221	SAMEA3733072	ERX1298558	ERR1226346	ITS	ITS2	NA	TCCTCCGCTTATTGATATGC	GCATCGATGAAGAACGCAGC	2008-07-11	52.32	-126.31	Canada	0.3	1100	1.7	146-193	Orthic Gray Luvisol, Gleyed Gray Luvisol	Lodgepole pine, Subalpine fir, Interior spruce	Dfc, Boreal cool summer	OM2	C1	0	A horizon	18.0	1.9	0.1	4.9	1.7	21.6
TO089F	TO089	SBS_BC_	British Columbia	TO	Forest Soil	Whole Community DNA	PCR	Amplicon	pyrotag library	454 GS FLX Titanium	European Nucleotide Archive	https://www.ebi.ac.uk/ena/data/search?query=PRJEB12501	ERS1040222	SAMEA3733073	ERX1298559	ERR1226347	ITS	ITS2	NA	TCCTCCGCTTATTGATATGC	GCATCGATGAAGAACGCAGC	2008-07-11	52.32	-126.31	Canada	0.3	1100	1.7	146-193	Orthic Gray Luvisol, Gleyed Gray Luvisol	Lodgepole pine, Subalpine fir, Interior spruce	Dfc, Boreal cool summer	OM2	C1	0	A horizon	22.0	1.9	0.1	4.9	1.7	21.6
TO090F	TO090	SBS_BC_	British Columbia	TO	Forest Soil	Whole Community DNA	PCR	Amplicon	pyrotag library	454 GS FLX Titanium	European Nucleotide Archive	https://www.ebi.ac.uk/ena/data/search?query=PRJEB12501	ERS1040223	SAMEA3733074	ERX1298560	ERR1226348	ITS	ITS2	NA	TCCTCCGCTTATTGATATGC	GCATCGATGAAGAACGCAGC	2008-07-11	52.32	-126.31	Canada	0.3	1100	1.7	146-193	Orthic Gray Luvisol, Gleyed Gray Luvisol	Lodgepole pine, Subalpine fir, Interior spruce	Dfc, Boreal cool summer	OM2	C1	0	A horizon	20.0	1.9	0.1	4.9	1.7	21.6
TO094F	TO094	SBS_BC_	British Columbia	TO	Forest Soil	Whole Community DNA	PCR	Amplicon	pyrotag library	454 GS FLX Titanium	European Nucleotide Archive	https://www.ebi.ac.uk/ena/data/search?query=PRJEB12501	ERS1040224	SAMEA3733075	ERX1298561	ERR1226349	ITS	ITS2	NA	TCCTCCGCTTATTGATATGC	GCATCGATGAAGAACGCAGC	2008-07-11	52.32	-126.31	Canada	0.3	1100	1.7	146-193	Orthic Gray Luvisol, Gleyed Gray Luvisol	Lodgepole pine, Subalpine fir, Interior spruce	Dfc, Boreal cool summer	OM3	C1	0	A horizon	16.0	1.7	0.1	5.5	1.7	21.0
TO095F	TO095	SBS_BC_	British Columbia	TO	Forest Soil	Whole Community DNA	PCR	Amplicon	pyrotag library	454 GS FLX Titanium	European Nucleotide Archive	https://www.ebi.ac.uk/ena/data/search?query=PRJEB12501	ERS1040225	SAMEA3733076	ERX1298562	ERR1226350	ITS	ITS2	NA	TCCTCCGCTTATTGATATGC	GCATCGATGAAGAACGCAGC	2008-07-11	52.32	-126.31	Canada	0.3	1100	1.7	146-193	Orthic Gray Luvisol, Gleyed Gray Luvisol	Lodgepole pine, Subalpine fir, Interior spruce	Dfc, Boreal cool summer	OM3	C1	0	A horizon	17.0	1.7	0.1	5.5	1.7	21.0
TO096F	TO096	SBS_BC_	British Columbia	TO	Forest Soil	Whole Community DNA	PCR	Amplicon	pyrotag library	454 GS FLX Titanium	European Nucleotide Archive	https://www.ebi.ac.uk/ena/data/search?query=PRJEB12501	ERS1040226	SAMEA3733077	ERX1298563	ERR1226351	ITS	ITS2	NA	TCCTCCGCTTATTGATATGC	GCATCGATGAAGAACGCAGC	2008-07-11	52.32	-126.31	Canada	0.3	1100	1.7	146-193	Orthic Gray Luvisol, Gleyed Gray Luvisol	Lodgepole pine, Subalpine fir, Interior spruce	Dfc, Boreal cool summer	OM3	C1	0	A horizon	15.0	1.7	0.1	5.5	1.7	21.0
TO097F	TO097	SBS_BC_	British Columbia	TO	Forest Soil	Whole Community DNA	PCR	Amplicon	pyrotag library	454 GS FLX Titanium	European Nucleotide Archive	https://www.ebi.ac.uk/ena/data/search?query=PRJEB12501	ERS1040227	SAMEA3733078	ERX1298564	ERR1226352	ITS	ITS2	NA	TCCTCCGCTTATTGATATGC	GCATCGATGAAGAACGCAGC	2008-07-11	52.32	-126.31	Canada	0.1	1100	1.7	146-193	Orthic Gray Luvisol, Gleyed Gray Luvisol	Lodgepole pine, Subalpine fir, Interior spruce	Dfc, Boreal cool summer	OM1	C2	0	O horizon	60.0	35.6	0.9	5.1	1.5	37.9
TO098F	TO098	SBS_BC_	British Columbia	TO	Forest Soil	Whole Community DNA	PCR	Amplicon	pyrotag library	454 GS FLX Titanium	European Nucleotide Archive	https://www.ebi.ac.uk/ena/data/search?query=PRJEB12501	ERS1040228	SAMEA3733079	ERX1298565	ERR1226353	ITS	ITS2	NA	TCCTCCGCTTATTGATATGC	GCATCGATGAAGAACGCAGC	2008-07-11	52.32	-126.31	Canada	0.1	1100	1.7	146-193	Orthic Gray Luvisol, Gleyed Gray Luvisol	Lodgepole pine, Subalpine fir, Interior spruce	Dfc, Boreal cool summer	OM1	C2	0	O horizon	64.0	35.6	0.9	5.1	1.5	37.9
TO099F	TO099	SBS_BC_	British Columbia	TO	Forest Soil	Whole Community DNA	PCR	Amplicon	pyrotag library	454 GS FLX Titanium	European Nucleotide Archive	https://www.ebi.ac.uk/ena/data/search?query=PRJEB12501	ERS1040229	SAMEA3733080	ERX1298566	ERR1226354	ITS	ITS2	NA	TCCTCCGCTTATTGATATGC	GCATCGATGAAGAACGCAGC	2008-07-11	52.32	-126.31	Canada	0.1	1100	1.7	146-193	Orthic Gray Luvisol, Gleyed Gray Luvisol	Lodgepole pine, Subalpine fir, Interior spruce	Dfc, Boreal cool summer	OM1	C2	0	O horizon	66.0	35.6	0.9	5.1	1.5	37.9
TO100F	TO100	SBS_BC_	British Columbia	TO	Forest Soil	Whole Community DNA	PCR	Amplicon	pyrotag library	454 GS FLX Titanium	European Nucleotide Archive	https://www.ebi.ac.uk/ena/data/search?query=PRJEB12501	ERS1040230	SAMEA3733081	ERX1298567	ERR1226355	ITS	ITS2	NA	TCCTCCGCTTATTGATATGC	GCATCGATGAAGAACGCAGC	2008-07-11	52.32	-126.31	Canada	0.3	1100	1.7	146-193	Orthic Gray Luvisol, Gleyed Gray Luvisol	Lodgepole pine, Subalpine fir, Interior spruce	Dfc, Boreal cool summer	OM1	C2	0	A horizon	21.0	2.3	0.1	5.2	1.5	20.7
TO101F	TO101	SBS_BC_	British Columbia	TO	Forest Soil	Whole Community DNA	PCR	Amplicon	pyrotag library	454 GS FLX Titanium	European Nucleotide Archive	https://www.ebi.ac.uk/ena/data/search?query=PRJEB12501	ERS1040231	SAMEA3733082	ERX1298568	ERR1226356	ITS	ITS2	NA	TCCTCCGCTTATTGATATGC	GCATCGATGAAGAACGCAGC	2008-07-11	52.32	-126.31	Canada	0.3	1100	1.7	146-193	Orthic Gray Luvisol, Gleyed Gray Luvisol	Lodgepole pine, Subalpine fir, Interior spruce	Dfc, Boreal cool summer	OM1	C2	0	A horizon	22.0	2.3	0.1	5.2	1.5	20.7
TO102F	TO102	SBS_BC_	British Columbia	TO	Forest Soil	Whole Community DNA	PCR	Amplicon	pyrotag library	454 GS FLX Titanium	European Nucleotide Archive	https://www.ebi.ac.uk/ena/data/search?query=PRJEB12501	ERS1040232	SAMEA3733083	ERX1298569	ERR1226357	ITS	ITS2	NA	TCCTCCGCTTATTGATATGC	GCATCGATGAAGAACGCAGC	2008-07-11	52.32	-126.31	Canada	0.3	1100	1.7	146-193	Orthic Gray Luvisol, Gleyed Gray Luvisol	Lodgepole pine, Subalpine fir, Interior spruce	Dfc, Boreal cool summer	OM1	C2	0	A horizon	17.0	2.3	0.1	5.2	1.5	20.7
TO103F	TO103	SBS_BC_	British Columbia	TO	Forest Soil	Whole Community DNA	PCR	Amplicon	pyrotag library	454 GS FLX Titanium	European Nucleotide Archive	https://www.ebi.ac.uk/ena/data/search?query=PRJEB12501	ERS1040233	SAMEA3733084	ERX1298570	ERR1226358	ITS	ITS2	NA	TCCTCCGCTTATTGATATGC	GCATCGATGAAGAACGCAGC	2008-07-11	52.32	-126.31	Canada	0.1	1100	1.7	146-193	Orthic Gray Luvisol, Gleyed Gray Luvisol	Lodgepole pine, Subalpine fir, Interior spruce	Dfc, Boreal cool summer	OM2	C2	0	O horizon	86.0	30.6	0.9	5.1	1.8	34.8
TO104F	TO104	SBS_BC_	British Columbia	TO	Forest Soil	Whole Community DNA	PCR	Amplicon	pyrotag library	454 GS FLX Titanium	European Nucleotide Archive	https://www.ebi.ac.uk/ena/data/search?query=PRJEB12501	ERS1040234	SAMEA3733085	ERX1298571	ERR1226359	ITS	ITS2	NA	TCCTCCGCTTATTGATATGC	GCATCGATGAAGAACGCAGC	2008-07-11	52.32	-126.31	Canada	0.1	1100	1.7	146-193	Orthic Gray Luvisol, Gleyed Gray Luvisol	Lodgepole pine, Subalpine fir, Interior spruce	Dfc, Boreal cool summer	OM2	C2	0	O horizon	74.0	30.6	0.9	5.1	1.8	34.8
TO105F	TO105	SBS_BC_	British Columbia	TO	Forest Soil	Whole Community DNA	PCR	Amplicon	pyrotag library	454 GS FLX Titanium	European Nucleotide Archive	https://www.ebi.ac.uk/ena/data/search?query=PRJEB12501	ERS1040235	SAMEA3733086	ERX1298572	ERR1226360	ITS	ITS2	NA	TCCTCCGCTTATTGATATGC	GCATCGATGAAGAACGCAGC	2008-07-11	52.32	-126.31	Canada	0.1	1100	1.7	146-193	Orthic Gray Luvisol, Gleyed Gray Luvisol	Lodgepole pine, Subalpine fir, Interior spruce	Dfc, Boreal cool summer	OM2	C2	0	O horizon	64.0	30.6	0.9	5.1	1.8	34.8
TO106F	TO106	SBS_BC_	British Columbia	TO	Forest Soil	Whole Community DNA	PCR	Amplicon	pyrotag library	454 GS FLX Titanium	European Nucleotide Archive	https://www.ebi.ac.uk/ena/data/search?query=PRJEB12501	ERS1040236	SAMEA3733087	ERX1298573	ERR1226361	ITS	ITS2	NA	TCCTCCGCTTATTGATATGC	GCATCGATGAAGAACGCAGC	2008-07-11	52.32	-126.31	Canada	0.3	1100	1.7	146-193	Orthic Gray Luvisol, Gleyed Gray Luvisol	Lodgepole pine, Subalpine fir, Interior spruce	Dfc, Boreal cool summer	OM2	C2	0	A horizon	18.0	3.1	0.2	5.1	1.8	19.6
TO107F	TO107	SBS_BC_	British Columbia	TO	Forest Soil	Whole Community DNA	PCR	Amplicon	pyrotag library	454 GS FLX Titanium	European Nucleotide Archive	https://www.ebi.ac.uk/ena/data/search?query=PRJEB12501	ERS1040237	SAMEA3733088	ERX1298574	ERR1226362	ITS	ITS2	NA	TCCTCCGCTTATTGATATGC	GCATCGATGAAGAACGCAGC	2008-07-11	52.32	-126.31	Canada	0.3	1100	1.7	146-193	Orthic Gray Luvisol, Gleyed Gray Luvisol	Lodgepole pine, Subalpine fir, Interior spruce	Dfc, Boreal cool summer	OM2	C2	0	A horizon	27.0	3.1	0.2	5.1	1.8	19.6
TO108F	TO108	SBS_BC_	British Columbia	TO	Forest Soil	Whole Community DNA	PCR	Amplicon	pyrotag library	454 GS FLX Titanium	European Nucleotide Archive	https://www.ebi.ac.uk/ena/data/search?query=PRJEB12501	ERS1040238	SAMEA3733089	ERX1298575	ERR1226363	ITS	ITS2	NA	TCCTCCGCTTATTGATATGC	GCATCGATGAAGAACGCAGC	2008-07-11	52.32	-126.31	Canada	0.3	1100	1.7	146-193	Orthic Gray Luvisol, Gleyed Gray Luvisol	Lodgepole pine, Subalpine fir, Interior spruce	Dfc, Boreal cool summer	OM2	C2	0	A horizon	18.0	3.1	0.2	5.1	1.8	19.6
TO112F	TO112	SBS_BC_	British Columbia	TO	Forest Soil	Whole Community DNA	PCR	Amplicon	pyrotag library	454 GS FLX Titanium	European Nucleotide Archive	https://www.ebi.ac.uk/ena/data/search?query=PRJEB12501	ERS1040239	SAMEA3733090	ERX1298576	ERR1226364	ITS	ITS2	NA	TCCTCCGCTTATTGATATGC	GCATCGATGAAGAACGCAGC	2008-07-11	52.32	-126.31	Canada	0.3	1100	1.7	146-193	Orthic Gray Luvisol, Gleyed Gray Luvisol	Lodgepole pine, Subalpine fir, Interior spruce	Dfc, Boreal cool summer	OM3	C2	0	A horizon	20.0	1.8	0.1	5.7	1.6	19.5
TO113F	TO113	SBS_BC_	British Columbia	TO	Forest Soil	Whole Community DNA	PCR	Amplicon	pyrotag library	454 GS FLX Titanium	European Nucleotide Archive	https://www.ebi.ac.uk/ena/data/search?query=PRJEB12501	ERS1040240	SAMEA3733091	ERX1298577	ERR1226365	ITS	ITS2	NA	TCCTCCGCTTATTGATATGC	GCATCGATGAAGAACGCAGC	2008-07-11	52.32	-126.31	Canada	0.3	1100	1.7	146-193	Orthic Gray Luvisol, Gleyed Gray Luvisol	Lodgepole pine, Subalpine fir, Interior spruce	Dfc, Boreal cool summer	OM3	C2	0	A horizon	16.0	1.8	0.1	5.7	1.6	19.5
TO114F	TO114	SBS_BC_	British Columbia	TO	Forest Soil	Whole Community DNA	PCR	Amplicon	pyrotag library	454 GS FLX Titanium	European Nucleotide Archive	https://www.ebi.ac.uk/ena/data/search?query=PRJEB12501	ERS1040241	SAMEA3733092	ERX1298578	ERR1226366	ITS	ITS2	NA	TCCTCCGCTTATTGATATGC	GCATCGATGAAGAACGCAGC	2008-07-11	52.32	-126.31	Canada	0.3	1100	1.7	146-193	Orthic Gray Luvisol, Gleyed Gray Luvisol	Lodgepole pine, Subalpine fir, Interior spruce	Dfc, Boreal cool summer	OM3	C2	0	A horizon	18.0	1.8	0.1	5.7	1.6	19.5
TO115F	TO115	SBS_BC_	British Columbia	TO	Forest Soil	Whole Community DNA	PCR	Amplicon	pyrotag library	454 GS FLX Titanium	European Nucleotide Archive	https://www.ebi.ac.uk/ena/data/search?query=PRJEB12501	ERS1040242	SAMEA3733093	ERX1298579	ERR1226367	ITS	ITS2	NA	TCCTCCGCTTATTGATATGC	GCATCGATGAAGAACGCAGC	2008-07-11	52.32	-126.31	Canada	0.1	1100	1.7	146-193	Orthic Gray Luvisol, Gleyed Gray Luvisol	Lodgepole pine, Subalpine fir, Interior spruce	Dfc, Boreal cool summer	REF	REF	0	O horizon	79.0	44.8	1.1	4.4	NA	42.3
TO116F	TO116	SBS_BC_	British Columbia	TO	Forest Soil	Whole Community DNA	PCR	Amplicon	pyrotag library	454 GS FLX Titanium	European Nucleotide Archive	https://www.ebi.ac.uk/ena/data/search?query=PRJEB12501	ERS1040243	SAMEA3733094	ERX1298580	ERR1226368	ITS	ITS2	NA	TCCTCCGCTTATTGATATGC	GCATCGATGAAGAACGCAGC	2008-07-11	52.32	-126.31	Canada	0.1	1100	1.7	146-193	Orthic Gray Luvisol, Gleyed Gray Luvisol	Lodgepole pine, Subalpine fir, Interior spruce	Dfc, Boreal cool summer	REF	REF	0	O horizon	76.0	44.8	1.1	4.4	NA	42.3
TO117F	TO117	SBS_BC_	British Columbia	TO	Forest Soil	Whole Community DNA	PCR	Amplicon	pyrotag library	454 GS FLX Titanium	European Nucleotide Archive	https://www.ebi.ac.uk/ena/data/search?query=PRJEB12501	ERS1040244	SAMEA3733095	ERX1298581	ERR1226369	ITS	ITS2	NA	TCCTCCGCTTATTGATATGC	GCATCGATGAAGAACGCAGC	2008-07-11	52.32	-126.31	Canada	0.1	1100	1.7	146-193	Orthic Gray Luvisol, Gleyed Gray Luvisol	Lodgepole pine, Subalpine fir, Interior spruce	Dfc, Boreal cool summer	REF	REF	0	O horizon	72.0	44.8	1.1	4.4	NA	42.3
TO118F	TO118	SBS_BC_	British Columbia	TO	Forest Soil	Whole Community DNA	PCR	Amplicon	pyrotag library	454 GS FLX Titanium	European Nucleotide Archive	https://www.ebi.ac.uk/ena/data/search?query=PRJEB12501	ERS1040245	SAMEA3733096	ERX1298582	ERR1226370	ITS	ITS2	NA	TCCTCCGCTTATTGATATGC	GCATCGATGAAGAACGCAGC	2008-07-11	52.32	-126.31	Canada	0.3	1100	1.7	146-193	Orthic Gray Luvisol, Gleyed Gray Luvisol	Lodgepole pine, Subalpine fir, Interior spruce	Dfc, Boreal cool summer	REF	REF	0	A horizon	20.0	1.1	0.1	5.0	NA	22.2
TO119F	TO119	SBS_BC_	British Columbia	TO	Forest Soil	Whole Community DNA	PCR	Amplicon	pyrotag library	454 GS FLX Titanium	European Nucleotide Archive	https://www.ebi.ac.uk/ena/data/search?query=PRJEB12501	ERS1040246	SAMEA3733097	ERX1298583	ERR1226371	ITS	ITS2	NA	TCCTCCGCTTATTGATATGC	GCATCGATGAAGAACGCAGC	2008-07-11	52.32	-126.31	Canada	0.3	1100	1.7	146-193	Orthic Gray Luvisol, Gleyed Gray Luvisol	Lodgepole pine, Subalpine fir, Interior spruce	Dfc, Boreal cool summer	REF	REF	0	A horizon	19.0	1.1	0.1	5.0	NA	22.2
TO120F	TO120	SBS_BC_	British Columbia	TO	Forest Soil	Whole Community DNA	PCR	Amplicon	pyrotag library	454 GS FLX Titanium	European Nucleotide Archive	https://www.ebi.ac.uk/ena/data/search?query=PRJEB12501	ERS1040247	SAMEA3733098	ERX1298584	ERR1226372	ITS	ITS2	NA	TCCTCCGCTTATTGATATGC	GCATCGATGAAGAACGCAGC	2008-07-11	52.32	-126.31	Canada	0.3	1100	1.7	146-193	Orthic Gray Luvisol, Gleyed Gray Luvisol	Lodgepole pine, Subalpine fir, Interior spruce	Dfc, Boreal cool summer	REF	REF	0	A horizon	25.0	1.1	0.1	5.0	NA	22.2
BP241B	BP241	IDF_BC_	British Columbia	BP	Forest Soil	Whole Community DNA	PCR	Amplicon	pyrotag library	454 GS FLX Titanium	European Nucleotide Archive	https://www.ebi.ac.uk/ena/data/search?query=PRJEB12501	ERS1040248	SAMEA3733099	ERX1298585	ERR1226373	16S rRNA	V1-V3	NA	AGAGTTTGATCMTGGCTCAG	GWATTACCGCGGCKGCTG	2010-06-22	50.93	-120.28	Canada	0.1	1180	2.5	300	Brunisolic Gray Luvisol	Douglas fir, Lodgepole pine	Dfb, Humid Continental warm summer	OM2	C2	0	O horizon	88.0	2.5	0.1	5.5	1.4	22.8
BP242B	BP242	IDF_BC_	British Columbia	BP	Forest Soil	Whole Community DNA	PCR	Amplicon	pyrotag library	454 GS FLX Titanium	European Nucleotide Archive	https://www.ebi.ac.uk/ena/data/search?query=PRJEB12501	ERS1040249	SAMEA3733100	ERX1298586	ERR1226374	16S rRNA	V1-V3	NA	AGAGTTTGATCMTGGCTCAG	GWATTACCGCGGCKGCTG	2010-06-22	50.93	-120.28	Canada	0.1	1180	2.5	300	Brunisolic Gray Luvisol	Douglas fir, Lodgepole pine	Dfb, Humid Continental warm summer	OM2	C2	0	O horizon	90.0	2.5	0.1	5.5	1.4	22.8
BP243B	BP243	IDF_BC_	British Columbia	BP	Forest Soil	Whole Community DNA	PCR	Amplicon	pyrotag library	454 GS FLX Titanium	European Nucleotide Archive	https://www.ebi.ac.uk/ena/data/search?query=PRJEB12501	ERS1040250	SAMEA3733101	ERX1298587	ERR1226375	16S rRNA	V1-V3	NA	AGAGTTTGATCMTGGCTCAG	GWATTACCGCGGCKGCTG	2010-06-22	50.93	-120.28	Canada	0.1	1180	2.5	300	Brunisolic Gray Luvisol	Douglas fir, Lodgepole pine	Dfb, Humid Continental warm summer	OM2	C2	0	O horizon	93.0	2.5	0.1	5.5	1.4	22.8
BP244B	BP244	IDF_BC_	British Columbia	BP	Forest Soil	Whole Community DNA	PCR	Amplicon	pyrotag library	454 GS FLX Titanium	European Nucleotide Archive	https://www.ebi.ac.uk/ena/data/search?query=PRJEB12501	ERS1040251	SAMEA3733102	ERX1298588	ERR1226376	16S rRNA	V1-V3	NA	AGAGTTTGATCMTGGCTCAG	GWATTACCGCGGCKGCTG	2010-06-22	50.93	-120.28	Canada	0.3	1180	2.5	300	Brunisolic Gray Luvisol	Douglas fir, Lodgepole pine	Dfb, Humid Continental warm summer	OM2	C2	0	A horizon	40.0	41.8	1.5	5.8	1.4	27.7
BP245B	BP245	IDF_BC_	British Columbia	BP	Forest Soil	Whole Community DNA	PCR	Amplicon	pyrotag library	454 GS FLX Titanium	European Nucleotide Archive	https://www.ebi.ac.uk/ena/data/search?query=PRJEB12501	ERS1040252	SAMEA3733103	ERX1298589	ERR1226377	16S rRNA	V1-V3	NA	AGAGTTTGATCMTGGCTCAG	GWATTACCGCGGCKGCTG	2010-06-22	50.93	-120.28	Canada	0.3	1180	2.5	300	Brunisolic Gray Luvisol	Douglas fir, Lodgepole pine	Dfb, Humid Continental warm summer	OM2	C2	0	A horizon	50.0	41.8	1.5	5.8	1.4	27.7
BP246B	BP246	IDF_BC_	British Columbia	BP	Forest Soil	Whole Community DNA	PCR	Amplicon	pyrotag library	454 GS FLX Titanium	European Nucleotide Archive	https://www.ebi.ac.uk/ena/data/search?query=PRJEB12501	ERS1040253	SAMEA3733104	ERX1298590	ERR1226378	16S rRNA	V1-V3	NA	AGAGTTTGATCMTGGCTCAG	GWATTACCGCGGCKGCTG	2010-06-22	50.93	-120.28	Canada	0.3	1180	2.5	300	Brunisolic Gray Luvisol	Douglas fir, Lodgepole pine	Dfb, Humid Continental warm summer	OM2	C2	0	A horizon	39.0	41.8	1.5	5.8	1.4	27.7
BP250B	BP250	IDF_BC_	British Columbia	BP	Forest Soil	Whole Community DNA	PCR	Amplicon	pyrotag library	454 GS FLX Titanium	European Nucleotide Archive	https://www.ebi.ac.uk/ena/data/search?query=PRJEB12501	ERS1040254	SAMEA3733105	ERX1298591	ERR1226379	16S rRNA	V1-V3	NA	AGAGTTTGATCMTGGCTCAG	GWATTACCGCGGCKGCTG	2010-06-22	50.93	-120.28	Canada	0.3	1180	2.5	300	Brunisolic Gray Luvisol	Douglas fir, Lodgepole pine	Dfb, Humid Continental warm summer	OM3	C2	0	A horizon	53.0	2.0	0.1	5.6	1.5	22.3
BP251B	BP251	IDF_BC_	British Columbia	BP	Forest Soil	Whole Community DNA	PCR	Amplicon	pyrotag library	454 GS FLX Titanium	European Nucleotide Archive	https://www.ebi.ac.uk/ena/data/search?query=PRJEB12501	ERS1040255	SAMEA3733106	ERX1298592	ERR1226380	16S rRNA	V1-V3	NA	AGAGTTTGATCMTGGCTCAG	GWATTACCGCGGCKGCTG	2010-06-22	50.93	-120.28	Canada	0.3	1180	2.5	300	Brunisolic Gray Luvisol	Douglas fir, Lodgepole pine	Dfb, Humid Continental warm summer	OM3	C2	0	A horizon	50.0	2.0	0.1	5.6	1.5	22.3
BP252B	BP252	IDF_BC_	British Columbia	BP	Forest Soil	Whole Community DNA	PCR	Amplicon	pyrotag library	454 GS FLX Titanium	European Nucleotide Archive	https://www.ebi.ac.uk/ena/data/search?query=PRJEB12501	ERS1040256	SAMEA3733107	ERX1298593	ERR1226381	16S rRNA	V1-V3	NA	AGAGTTTGATCMTGGCTCAG	GWATTACCGCGGCKGCTG	2010-06-22	50.93	-120.28	Canada	0.3	1180	2.5	300	Brunisolic Gray Luvisol	Douglas fir, Lodgepole pine	Dfb, Humid Continental warm summer	OM3	C2	0	A horizon	38.0	2.0	0.1	5.6	1.5	22.3
BP256B	BP256	IDF_BC_	British Columbia	BP	Forest Soil	Whole Community DNA	PCR	Amplicon	pyrotag library	454 GS FLX Titanium	European Nucleotide Archive	https://www.ebi.ac.uk/ena/data/search?query=PRJEB12501	ERS1040257	SAMEA3733108	ERX1298594	ERR1226382	16S rRNA	V1-V3	NA	AGAGTTTGATCMTGGCTCAG	GWATTACCGCGGCKGCTG	2010-06-22	50.93	-120.28	Canada	0.3	1180	2.5	300	Brunisolic Gray Luvisol	Douglas fir, Lodgepole pine	Dfb, Humid Continental warm summer	OM3	C1	0	A horizon	45.0	2.6	0.1	5.7	1.5	23.6
BP257B	BP257	IDF_BC_	British Columbia	BP	Forest Soil	Whole Community DNA	PCR	Amplicon	pyrotag library	454 GS FLX Titanium	European Nucleotide Archive	https://www.ebi.ac.uk/ena/data/search?query=PRJEB12501	ERS1040258	SAMEA3733109	ERX1298595	ERR1226383	16S rRNA	V1-V3	NA	AGAGTTTGATCMTGGCTCAG	GWATTACCGCGGCKGCTG	2010-06-22	50.93	-120.28	Canada	0.3	1180	2.5	300	Brunisolic Gray Luvisol	Douglas fir, Lodgepole pine	Dfb, Humid Continental warm summer	OM3	C1	0	A horizon	55.0	2.6	0.1	5.7	1.5	23.6
BP258B	BP258	IDF_BC_	British Columbia	BP	Forest Soil	Whole Community DNA	PCR	Amplicon	pyrotag library	454 GS FLX Titanium	European Nucleotide Archive	https://www.ebi.ac.uk/ena/data/search?query=PRJEB12501	ERS1040259	SAMEA3733110	ERX1298596	ERR1226384	16S rRNA	V1-V3	NA	AGAGTTTGATCMTGGCTCAG	GWATTACCGCGGCKGCTG	2010-06-22	50.93	-120.28	Canada	0.3	1180	2.5	300	Brunisolic Gray Luvisol	Douglas fir, Lodgepole pine	Dfb, Humid Continental warm summer	OM3	C1	0	A horizon	46.0	2.6	0.1	5.7	1.5	23.6
BP259B	BP259	IDF_BC_	British Columbia	BP	Forest Soil	Whole Community DNA	PCR	Amplicon	pyrotag library	454 GS FLX Titanium	European Nucleotide Archive	https://www.ebi.ac.uk/ena/data/search?query=PRJEB12501	ERS1040260	SAMEA3733111	ERX1298597	ERR1226385	16S rRNA	V1-V3	NA	AGAGTTTGATCMTGGCTCAG	GWATTACCGCGGCKGCTG	2010-06-22	50.93	-120.28	Canada	0.1	1180	2.5	300	Brunisolic Gray Luvisol	Douglas fir, Lodgepole pine	Dfb, Humid Continental warm summer	OM1	C0	0	O horizon	88.0	38.7	1.1	5.6	1.0	35.2
BP260B	BP260	IDF_BC_	British Columbia	BP	Forest Soil	Whole Community DNA	PCR	Amplicon	pyrotag library	454 GS FLX Titanium	European Nucleotide Archive	https://www.ebi.ac.uk/ena/data/search?query=PRJEB12501	ERS1040261	SAMEA3733112	ERX1298598	ERR1226386	16S rRNA	V1-V3	NA	AGAGTTTGATCMTGGCTCAG	GWATTACCGCGGCKGCTG	2010-06-22	50.93	-120.28	Canada	0.1	1180	2.5	300	Brunisolic Gray Luvisol	Douglas fir, Lodgepole pine	Dfb, Humid Continental warm summer	OM1	C0	0	O horizon	88.0	38.7	1.1	5.6	1.0	35.2
BP261B	BP261	IDF_BC_	British Columbia	BP	Forest Soil	Whole Community DNA	PCR	Amplicon	pyrotag library	454 GS FLX Titanium	European Nucleotide Archive	https://www.ebi.ac.uk/ena/data/search?query=PRJEB12501	ERS1040262	SAMEA3733113	ERX1298599	ERR1226387	16S rRNA	V1-V3	NA	AGAGTTTGATCMTGGCTCAG	GWATTACCGCGGCKGCTG	2010-06-22	50.93	-120.28	Canada	0.1	1180	2.5	300	Brunisolic Gray Luvisol	Douglas fir, Lodgepole pine	Dfb, Humid Continental warm summer	OM1	C0	0	O horizon	83.0	38.7	1.1	5.6	1.0	35.2
BP262B	BP262	IDF_BC_	British Columbia	BP	Forest Soil	Whole Community DNA	PCR	Amplicon	pyrotag library	454 GS FLX Titanium	European Nucleotide Archive	https://www.ebi.ac.uk/ena/data/search?query=PRJEB12501	ERS1040263	SAMEA3733114	ERX1298600	ERR1226388	16S rRNA	V1-V3	NA	AGAGTTTGATCMTGGCTCAG	GWATTACCGCGGCKGCTG	2010-06-22	50.93	-120.28	Canada	0.3	1180	2.5	300	Brunisolic Gray Luvisol	Douglas fir, Lodgepole pine	Dfb, Humid Continental warm summer	OM1	C0	0	A horizon	43.0	3.0	0.1	5.4	1.0	27.1
BP263B	BP263	IDF_BC_	British Columbia	BP	Forest Soil	Whole Community DNA	PCR	Amplicon	pyrotag library	454 GS FLX Titanium	European Nucleotide Archive	https://www.ebi.ac.uk/ena/data/search?query=PRJEB12501	ERS1040264	SAMEA3733115	ERX1298601	ERR1226389	16S rRNA	V1-V3	NA	AGAGTTTGATCMTGGCTCAG	GWATTACCGCGGCKGCTG	2010-06-22	50.93	-120.28	Canada	0.3	1180	2.5	300	Brunisolic Gray Luvisol	Douglas fir, Lodgepole pine	Dfb, Humid Continental warm summer	OM1	C0	0	A horizon	48.0	3.0	0.1	5.4	1.0	27.1
BP264B	BP264	IDF_BC_	British Columbia	BP	Forest Soil	Whole Community DNA	PCR	Amplicon	pyrotag library	454 GS FLX Titanium	European Nucleotide Archive	https://www.ebi.ac.uk/ena/data/search?query=PRJEB12501	ERS1040265	SAMEA3733116	ERX1298602	ERR1226390	16S rRNA	V1-V3	NA	AGAGTTTGATCMTGGCTCAG	GWATTACCGCGGCKGCTG	2010-06-22	50.93	-120.28	Canada	0.3	1180	2.5	300	Brunisolic Gray Luvisol	Douglas fir, Lodgepole pine	Dfb, Humid Continental warm summer	OM1	C0	0	A horizon	47.0	3.0	0.1	5.4	1.0	27.1
BP265B	BP265	IDF_BC_	British Columbia	BP	Forest Soil	Whole Community DNA	PCR	Amplicon	pyrotag library	454 GS FLX Titanium	European Nucleotide Archive	https://www.ebi.ac.uk/ena/data/search?query=PRJEB12501	ERS1040266	SAMEA3733117	ERX1298603	ERR1226391	16S rRNA	V1-V3	NA	AGAGTTTGATCMTGGCTCAG	GWATTACCGCGGCKGCTG	2010-06-22	50.93	-120.28	Canada	0.1	1180	2.5	300	Brunisolic Gray Luvisol	Douglas fir, Lodgepole pine	Dfb, Humid Continental warm summer	OM2	C0	0	O horizon	79.0	43.2	0.9	5.1	1.1	50.9
BP266B	BP266	IDF_BC_	British Columbia	BP	Forest Soil	Whole Community DNA	PCR	Amplicon	pyrotag library	454 GS FLX Titanium	European Nucleotide Archive	https://www.ebi.ac.uk/ena/data/search?query=PRJEB12501	ERS1040267	SAMEA3733118	ERX1298604	ERR1226392	16S rRNA	V1-V3	NA	AGAGTTTGATCMTGGCTCAG	GWATTACCGCGGCKGCTG	2010-06-22	50.93	-120.28	Canada	0.1	1180	2.5	300	Brunisolic Gray Luvisol	Douglas fir, Lodgepole pine	Dfb, Humid Continental warm summer	OM2	C0	0	O horizon	83.0	43.2	0.9	5.1	1.1	50.9
BP267B	BP267	IDF_BC_	British Columbia	BP	Forest Soil	Whole Community DNA	PCR	Amplicon	pyrotag library	454 GS FLX Titanium	European Nucleotide Archive	https://www.ebi.ac.uk/ena/data/search?query=PRJEB12501	ERS1040268	SAMEA3733119	ERX1298605	ERR1226393	16S rRNA	V1-V3	NA	AGAGTTTGATCMTGGCTCAG	GWATTACCGCGGCKGCTG	2010-06-22	50.93	-120.28	Canada	0.1	1180	2.5	300	Brunisolic Gray Luvisol	Douglas fir, Lodgepole pine	Dfb, Humid Continental warm summer	OM2	C0	0	O horizon	79.0	43.2	0.9	5.1	1.1	50.9
BP268B	BP268	IDF_BC_	British Columbia	BP	Forest Soil	Whole Community DNA	PCR	Amplicon	pyrotag library	454 GS FLX Titanium	European Nucleotide Archive	https://www.ebi.ac.uk/ena/data/search?query=PRJEB12501	ERS1040269	SAMEA3733120	ERX1298606	ERR1226394	16S rRNA	V1-V3	NA	AGAGTTTGATCMTGGCTCAG	GWATTACCGCGGCKGCTG	2010-06-22	50.93	-120.28	Canada	0.3	1180	2.5	300	Brunisolic Gray Luvisol	Douglas fir, Lodgepole pine	Dfb, Humid Continental warm summer	OM2	C0	0	A horizon	44.0	2.8	0.1	5.4	1.1	27.6
BP269B	BP269	IDF_BC_	British Columbia	BP	Forest Soil	Whole Community DNA	PCR	Amplicon	pyrotag library	454 GS FLX Titanium	European Nucleotide Archive	https://www.ebi.ac.uk/ena/data/search?query=PRJEB12501	ERS1040270	SAMEA3733121	ERX1298607	ERR1226395	16S rRNA	V1-V3	NA	AGAGTTTGATCMTGGCTCAG	GWATTACCGCGGCKGCTG	2010-06-22	50.93	-120.28	Canada	0.3	1180	2.5	300	Brunisolic Gray Luvisol	Douglas fir, Lodgepole pine	Dfb, Humid Continental warm summer	OM2	C0	0	A horizon	47.0	2.8	0.1	5.4	1.1	27.6
BP270B	BP270	IDF_BC_	British Columbia	BP	Forest Soil	Whole Community DNA	PCR	Amplicon	pyrotag library	454 GS FLX Titanium	European Nucleotide Archive	https://www.ebi.ac.uk/ena/data/search?query=PRJEB12501	ERS1040271	SAMEA3733122	ERX1298608	ERR1226396	16S rRNA	V1-V3	NA	AGAGTTTGATCMTGGCTCAG	GWATTACCGCGGCKGCTG	2010-06-22	50.93	-120.28	Canada	0.3	1180	2.5	300	Brunisolic Gray Luvisol	Douglas fir, Lodgepole pine	Dfb, Humid Continental warm summer	OM2	C0	0	A horizon	51.0	2.8	0.1	5.4	1.1	27.6
BP271B	BP271	IDF_BC_	British Columbia	BP	Forest Soil	Whole Community DNA	PCR	Amplicon	pyrotag library	454 GS FLX Titanium	European Nucleotide Archive	https://www.ebi.ac.uk/ena/data/search?query=PRJEB12501	ERS1040272	SAMEA3733123	ERX1298609	ERR1226397	16S rRNA	V1-V3	NA	AGAGTTTGATCMTGGCTCAG	GWATTACCGCGGCKGCTG	2010-06-22	50.93	-120.28	Canada	0.1	1180	2.5	300	Brunisolic Gray Luvisol	Douglas fir, Lodgepole pine	Dfb, Humid Continental warm summer	OM2	C1	0	O horizon	89.0	38.6	1.0	5.3	1.3	37.5
BP272B	BP272	IDF_BC_	British Columbia	BP	Forest Soil	Whole Community DNA	PCR	Amplicon	pyrotag library	454 GS FLX Titanium	European Nucleotide Archive	https://www.ebi.ac.uk/ena/data/search?query=PRJEB12501	ERS1040273	SAMEA3733124	ERX1298610	ERR1226398	16S rRNA	V1-V3	NA	AGAGTTTGATCMTGGCTCAG	GWATTACCGCGGCKGCTG	2010-06-22	50.93	-120.28	Canada	0.1	1180	2.5	300	Brunisolic Gray Luvisol	Douglas fir, Lodgepole pine	Dfb, Humid Continental warm summer	OM2	C1	0	O horizon	83.0	38.6	1.0	5.3	1.3	37.5
BP273B	BP273	IDF_BC_	British Columbia	BP	Forest Soil	Whole Community DNA	PCR	Amplicon	pyrotag library	454 GS FLX Titanium	European Nucleotide Archive	https://www.ebi.ac.uk/ena/data/search?query=PRJEB12501	ERS1040274	SAMEA3733125	ERX1298611	ERR1226399	16S rRNA	V1-V3	NA	AGAGTTTGATCMTGGCTCAG	GWATTACCGCGGCKGCTG	2010-06-22	50.93	-120.28	Canada	0.1	1180	2.5	300	Brunisolic Gray Luvisol	Douglas fir, Lodgepole pine	Dfb, Humid Continental warm summer	OM2	C1	0	O horizon	88.0	38.6	1.0	5.3	1.3	37.5
BP274B	BP274	IDF_BC_	British Columbia	BP	Forest Soil	Whole Community DNA	PCR	Amplicon	pyrotag library	454 GS FLX Titanium	European Nucleotide Archive	https://www.ebi.ac.uk/ena/data/search?query=PRJEB12501	ERS1040275	SAMEA3733126	ERX1298612	ERR1226400	16S rRNA	V1-V3	NA	AGAGTTTGATCMTGGCTCAG	GWATTACCGCGGCKGCTG	2010-06-22	50.93	-120.28	Canada	0.3	1180	2.5	300	Brunisolic Gray Luvisol	Douglas fir, Lodgepole pine	Dfb, Humid Continental warm summer	OM2	C1	0	A horizon	43.0	2.7	0.1	5.5	1.3	24.2
BP275B	BP275	IDF_BC_	British Columbia	BP	Forest Soil	Whole Community DNA	PCR	Amplicon	pyrotag library	454 GS FLX Titanium	European Nucleotide Archive	https://www.ebi.ac.uk/ena/data/search?query=PRJEB12501	ERS1040276	SAMEA3733127	ERX1298613	ERR1226401	16S rRNA	V1-V3	NA	AGAGTTTGATCMTGGCTCAG	GWATTACCGCGGCKGCTG	2010-06-22	50.93	-120.28	Canada	0.3	1180	2.5	300	Brunisolic Gray Luvisol	Douglas fir, Lodgepole pine	Dfb, Humid Continental warm summer	OM2	C1	0	A horizon	50.0	2.7	0.1	5.5	1.3	24.2
BP276B	BP276	IDF_BC_	British Columbia	BP	Forest Soil	Whole Community DNA	PCR	Amplicon	pyrotag library	454 GS FLX Titanium	European Nucleotide Archive	https://www.ebi.ac.uk/ena/data/search?query=PRJEB12501	ERS1040277	SAMEA3733128	ERX1298614	ERR1226402	16S rRNA	V1-V3	NA	AGAGTTTGATCMTGGCTCAG	GWATTACCGCGGCKGCTG	2010-06-22	50.93	-120.28	Canada	0.3	1180	2.5	300	Brunisolic Gray Luvisol	Douglas fir, Lodgepole pine	Dfb, Humid Continental warm summer	OM2	C1	0	A horizon	44.0	2.7	0.1	5.5	1.3	24.2
BP277B	BP277	IDF_BC_	British Columbia	BP	Forest Soil	Whole Community DNA	PCR	Amplicon	pyrotag library	454 GS FLX Titanium	European Nucleotide Archive	https://www.ebi.ac.uk/ena/data/search?query=PRJEB12501	ERS1040278	SAMEA3733129	ERX1298615	ERR1226403	16S rRNA	V1-V3	NA	AGAGTTTGATCMTGGCTCAG	GWATTACCGCGGCKGCTG	2010-06-22	50.93	-120.28	Canada	0.1	1180	2.5	300	Brunisolic Gray Luvisol	Douglas fir, Lodgepole pine	Dfb, Humid Continental warm summer	OM1	C1	0	O horizon	87.0	33.4	0.9	5.2	1.2	38.4
BP278B	BP278	IDF_BC_	British Columbia	BP	Forest Soil	Whole Community DNA	PCR	Amplicon	pyrotag library	454 GS FLX Titanium	European Nucleotide Archive	https://www.ebi.ac.uk/ena/data/search?query=PRJEB12501	ERS1040279	SAMEA3733130	ERX1298616	ERR1226404	16S rRNA	V1-V3	NA	AGAGTTTGATCMTGGCTCAG	GWATTACCGCGGCKGCTG	2010-06-22	50.93	-120.28	Canada	0.1	1180	2.5	300	Brunisolic Gray Luvisol	Douglas fir, Lodgepole pine	Dfb, Humid Continental warm summer	OM1	C1	0	O horizon	86.0	33.4	0.9	5.2	1.2	38.4
BP279B	BP279	IDF_BC_	British Columbia	BP	Forest Soil	Whole Community DNA	PCR	Amplicon	pyrotag library	454 GS FLX Titanium	European Nucleotide Archive	https://www.ebi.ac.uk/ena/data/search?query=PRJEB12501	ERS1040280	SAMEA3733131	ERX1298617	ERR1226405	16S rRNA	V1-V3	NA	AGAGTTTGATCMTGGCTCAG	GWATTACCGCGGCKGCTG	2010-06-22	50.93	-120.28	Canada	0.1	1180	2.5	300	Brunisolic Gray Luvisol	Douglas fir, Lodgepole pine	Dfb, Humid Continental warm summer	OM1	C1	0	O horizon	75.0	33.4	0.9	5.2	1.2	38.4
BP280B	BP280	IDF_BC_	British Columbia	BP	Forest Soil	Whole Community DNA	PCR	Amplicon	pyrotag library	454 GS FLX Titanium	European Nucleotide Archive	https://www.ebi.ac.uk/ena/data/search?query=PRJEB12501	ERS1040281	SAMEA3733132	ERX1298618	ERR1226406	16S rRNA	V1-V3	NA	AGAGTTTGATCMTGGCTCAG	GWATTACCGCGGCKGCTG	2010-06-22	50.93	-120.28	Canada	0.3	1180	2.5	300	Brunisolic Gray Luvisol	Douglas fir, Lodgepole pine	Dfb, Humid Continental warm summer	OM1	C1	0	A horizon	53.0	2.1	0.1	5.3	1.2	22.9
BP281B	BP281	IDF_BC_	British Columbia	BP	Forest Soil	Whole Community DNA	PCR	Amplicon	pyrotag library	454 GS FLX Titanium	European Nucleotide Archive	https://www.ebi.ac.uk/ena/data/search?query=PRJEB12501	ERS1040282	SAMEA3733133	ERX1298619	ERR1226407	16S rRNA	V1-V3	NA	AGAGTTTGATCMTGGCTCAG	GWATTACCGCGGCKGCTG	2010-06-22	50.93	-120.28	Canada	0.3	1180	2.5	300	Brunisolic Gray Luvisol	Douglas fir, Lodgepole pine	Dfb, Humid Continental warm summer	OM1	C1	0	A horizon	51.0	2.1	0.1	5.3	1.2	22.9
BP282B	BP282	IDF_BC_	British Columbia	BP	Forest Soil	Whole Community DNA	PCR	Amplicon	pyrotag library	454 GS FLX Titanium	European Nucleotide Archive	https://www.ebi.ac.uk/ena/data/search?query=PRJEB12501	ERS1040283	SAMEA3733134	ERX1298620	ERR1226408	16S rRNA	V1-V3	NA	AGAGTTTGATCMTGGCTCAG	GWATTACCGCGGCKGCTG	2010-06-22	50.93	-120.28	Canada	0.3	1180	2.5	300	Brunisolic Gray Luvisol	Douglas fir, Lodgepole pine	Dfb, Humid Continental warm summer	OM1	C1	0	A horizon	45.0	2.1	0.1	5.3	1.2	22.9
BP286B	BP286	IDF_BC_	British Columbia	BP	Forest Soil	Whole Community DNA	PCR	Amplicon	pyrotag library	454 GS FLX Titanium	European Nucleotide Archive	https://www.ebi.ac.uk/ena/data/search?query=PRJEB12501	ERS1040284	SAMEA3733135	ERX1298621	ERR1226409	16S rRNA	V1-V3	NA	AGAGTTTGATCMTGGCTCAG	GWATTACCGCGGCKGCTG	2010-06-22	50.93	-120.28	Canada	0.3	1180	2.5	300	Brunisolic Gray Luvisol	Douglas fir, Lodgepole pine	Dfb, Humid Continental warm summer	OM3	C0	0	A horizon	42.0	2.1	0.1	5.7	1.3	23.3
BP287B	BP287	IDF_BC_	British Columbia	BP	Forest Soil	Whole Community DNA	PCR	Amplicon	pyrotag library	454 GS FLX Titanium	European Nucleotide Archive	https://www.ebi.ac.uk/ena/data/search?query=PRJEB12501	ERS1040285	SAMEA3733136	ERX1298622	ERR1226410	16S rRNA	V1-V3	NA	AGAGTTTGATCMTGGCTCAG	GWATTACCGCGGCKGCTG	2010-06-22	50.93	-120.28	Canada	0.3	1180	2.5	300	Brunisolic Gray Luvisol	Douglas fir, Lodgepole pine	Dfb, Humid Continental warm summer	OM3	C0	0	A horizon	48.0	2.1	0.1	5.7	1.3	23.3
BP288B	BP288	IDF_BC_	British Columbia	BP	Forest Soil	Whole Community DNA	PCR	Amplicon	pyrotag library	454 GS FLX Titanium	European Nucleotide Archive	https://www.ebi.ac.uk/ena/data/search?query=PRJEB12501	ERS1040286	SAMEA3733137	ERX1298623	ERR1226411	16S rRNA	V1-V3	NA	AGAGTTTGATCMTGGCTCAG	GWATTACCGCGGCKGCTG	2010-06-22	50.93	-120.28	Canada	0.3	1180	2.5	300	Brunisolic Gray Luvisol	Douglas fir, Lodgepole pine	Dfb, Humid Continental warm summer	OM3	C0	0	A horizon	47.0	2.1	0.1	5.7	1.3	23.3
BP289B	BP289	IDF_BC_	British Columbia	BP	Forest Soil	Whole Community DNA	PCR	Amplicon	pyrotag library	454 GS FLX Titanium	European Nucleotide Archive	https://www.ebi.ac.uk/ena/data/search?query=PRJEB12501	ERS1040287	SAMEA3733138	ERX1298624	ERR1226412	16S rRNA	V1-V3	NA	AGAGTTTGATCMTGGCTCAG	GWATTACCGCGGCKGCTG	2010-06-22	50.93	-120.28	Canada	0.1	1180	2.5	300	Brunisolic Gray Luvisol	Douglas fir, Lodgepole pine	Dfb, Humid Continental warm summer	OM1	C2	0	O horizon	80.0	37.6	0.9	5.1	1.4	41.4
BP290B	BP290	IDF_BC_	British Columbia	BP	Forest Soil	Whole Community DNA	PCR	Amplicon	pyrotag library	454 GS FLX Titanium	European Nucleotide Archive	https://www.ebi.ac.uk/ena/data/search?query=PRJEB12501	ERS1040288	SAMEA3733139	ERX1298625	ERR1226413	16S rRNA	V1-V3	NA	AGAGTTTGATCMTGGCTCAG	GWATTACCGCGGCKGCTG	2010-06-22	50.93	-120.28	Canada	0.1	1180	2.5	300	Brunisolic Gray Luvisol	Douglas fir, Lodgepole pine	Dfb, Humid Continental warm summer	OM1	C2	0	O horizon	87.0	37.6	0.9	5.1	1.4	41.4
BP291B	BP291	IDF_BC_	British Columbia	BP	Forest Soil	Whole Community DNA	PCR	Amplicon	pyrotag library	454 GS FLX Titanium	European Nucleotide Archive	https://www.ebi.ac.uk/ena/data/search?query=PRJEB12501	ERS1040289	SAMEA3733140	ERX1298626	ERR1226414	16S rRNA	V1-V3	NA	AGAGTTTGATCMTGGCTCAG	GWATTACCGCGGCKGCTG	2010-06-22	50.93	-120.28	Canada	0.1	1180	2.5	300	Brunisolic Gray Luvisol	Douglas fir, Lodgepole pine	Dfb, Humid Continental warm summer	OM1	C2	0	O horizon	80.0	37.6	0.9	5.1	1.4	41.4
BP292B	BP292	IDF_BC_	British Columbia	BP	Forest Soil	Whole Community DNA	PCR	Amplicon	pyrotag library	454 GS FLX Titanium	European Nucleotide Archive	https://www.ebi.ac.uk/ena/data/search?query=PRJEB12501	ERS1040290	SAMEA3733141	ERX1298627	ERR1226415	16S rRNA	V1-V3	NA	AGAGTTTGATCMTGGCTCAG	GWATTACCGCGGCKGCTG	2010-06-22	50.93	-120.28	Canada	0.3	1180	2.5	300	Brunisolic Gray Luvisol	Douglas fir, Lodgepole pine	Dfb, Humid Continental warm summer	OM1	C2	0	A horizon	47.0	3.6	0.2	5.7	1.4	23.7
BP293B	BP293	IDF_BC_	British Columbia	BP	Forest Soil	Whole Community DNA	PCR	Amplicon	pyrotag library	454 GS FLX Titanium	European Nucleotide Archive	https://www.ebi.ac.uk/ena/data/search?query=PRJEB12501	ERS1040291	SAMEA3733142	ERX1298628	ERR1226416	16S rRNA	V1-V3	NA	AGAGTTTGATCMTGGCTCAG	GWATTACCGCGGCKGCTG	2010-06-22	50.93	-120.28	Canada	0.3	1180	2.5	300	Brunisolic Gray Luvisol	Douglas fir, Lodgepole pine	Dfb, Humid Continental warm summer	OM1	C2	0	A horizon	49.0	3.6	0.2	5.7	1.4	23.7
BP294B	BP294	IDF_BC_	British Columbia	BP	Forest Soil	Whole Community DNA	PCR	Amplicon	pyrotag library	454 GS FLX Titanium	European Nucleotide Archive	https://www.ebi.ac.uk/ena/data/search?query=PRJEB12501	ERS1040292	SAMEA3733143	ERX1298629	ERR1226417	16S rRNA	V1-V3	NA	AGAGTTTGATCMTGGCTCAG	GWATTACCGCGGCKGCTG	2010-06-22	50.93	-120.28	Canada	0.3	1180	2.5	300	Brunisolic Gray Luvisol	Douglas fir, Lodgepole pine	Dfb, Humid Continental warm summer	OM1	C2	0	A horizon	42.0	3.6	0.2	5.7	1.4	23.7
BP295B	BP295	IDF_BC_	British Columbia	BP	Forest Soil	Whole Community DNA	PCR	Amplicon	pyrotag library	454 GS FLX Titanium	European Nucleotide Archive	https://www.ebi.ac.uk/ena/data/search?query=PRJEB12501	ERS1040293	SAMEA3733144	ERX1298630	ERR1226418	16S rRNA	V1-V3	NA	AGAGTTTGATCMTGGCTCAG	GWATTACCGCGGCKGCTG	2010-06-22	50.93	-120.28	Canada	0.1	1180	2.5	300	Brunisolic Gray Luvisol	Douglas fir, Lodgepole pine	Dfb, Humid Continental warm summer	REF	REF	0	O horizon	74.0	44.4	1.3	5.4	NA	35.5
BP296B	BP296	IDF_BC_	British Columbia	BP	Forest Soil	Whole Community DNA	PCR	Amplicon	pyrotag library	454 GS FLX Titanium	European Nucleotide Archive	https://www.ebi.ac.uk/ena/data/search?query=PRJEB12501	ERS1040294	SAMEA3733145	ERX1298631	ERR1226419	16S rRNA	V1-V3	NA	AGAGTTTGATCMTGGCTCAG	GWATTACCGCGGCKGCTG	2010-06-22	50.93	-120.28	Canada	0.1	1180	2.5	300	Brunisolic Gray Luvisol	Douglas fir, Lodgepole pine	Dfb, Humid Continental warm summer	REF	REF	0	O horizon	88.0	44.4	1.3	5.4	NA	35.5
BP297B	BP297	IDF_BC_	British Columbia	BP	Forest Soil	Whole Community DNA	PCR	Amplicon	pyrotag library	454 GS FLX Titanium	European Nucleotide Archive	https://www.ebi.ac.uk/ena/data/search?query=PRJEB12501	ERS1040295	SAMEA3733146	ERX1298632	ERR1226420	16S rRNA	V1-V3	NA	AGAGTTTGATCMTGGCTCAG	GWATTACCGCGGCKGCTG	2010-06-22	50.93	-120.28	Canada	0.1	1180	2.5	300	Brunisolic Gray Luvisol	Douglas fir, Lodgepole pine	Dfb, Humid Continental warm summer	REF	REF	0	O horizon	84.0	44.4	1.3	5.4	NA	35.5
BP298B	BP298	IDF_BC_	British Columbia	BP	Forest Soil	Whole Community DNA	PCR	Amplicon	pyrotag library	454 GS FLX Titanium	European Nucleotide Archive	https://www.ebi.ac.uk/ena/data/search?query=PRJEB12501	ERS1040296	SAMEA3733147	ERX1298633	ERR1226421	16S rRNA	V1-V3	NA	AGAGTTTGATCMTGGCTCAG	GWATTACCGCGGCKGCTG	2010-06-22	50.93	-120.28	Canada	0.3	1180	2.5	300	Brunisolic Gray Luvisol	Douglas fir, Lodgepole pine	Dfb, Humid Continental warm summer	REF	REF	0	A horizon	46.0	2.4	0.1	5.6	NA	24.2
BP299B	BP299	IDF_BC_	British Columbia	BP	Forest Soil	Whole Community DNA	PCR	Amplicon	pyrotag library	454 GS FLX Titanium	European Nucleotide Archive	https://www.ebi.ac.uk/ena/data/search?query=PRJEB12501	ERS1040297	SAMEA3733148	ERX1298634	ERR1226422	16S rRNA	V1-V3	NA	AGAGTTTGATCMTGGCTCAG	GWATTACCGCGGCKGCTG	2010-06-22	50.93	-120.28	Canada	0.3	1180	2.5	300	Brunisolic Gray Luvisol	Douglas fir, Lodgepole pine	Dfb, Humid Continental warm summer	REF	REF	0	A horizon	44.0	2.4	0.1	5.6	NA	24.2
BP300B	BP300	IDF_BC_	British Columbia	BP	Forest Soil	Whole Community DNA	PCR	Amplicon	pyrotag library	454 GS FLX Titanium	European Nucleotide Archive	https://www.ebi.ac.uk/ena/data/search?query=PRJEB12501	ERS1040298	SAMEA3733149	ERX1298635	ERR1226423	16S rRNA	V1-V3	NA	AGAGTTTGATCMTGGCTCAG	GWATTACCGCGGCKGCTG	2010-06-22	50.93	-120.28	Canada	0.3	1180	2.5	300	Brunisolic Gray Luvisol	Douglas fir, Lodgepole pine	Dfb, Humid Continental warm summer	REF	REF	0	A horizon	45.0	2.4	0.1	5.6	NA	24.2
DC184B	DC184	IDF_BC_	British Columbia	DC	Forest Soil	Whole Community DNA	PCR	Amplicon	pyrotag library	454 GS FLX Titanium	European Nucleotide Archive	https://www.ebi.ac.uk/ena/data/search?query=PRJEB12501	ERS1040299	SAMEA3733150	ERX1298636	ERR1226424	16S rRNA	V1-V3	NA	AGAGTTTGATCMTGGCTCAG	GWATTACCGCGGCKGCTG	2010-06-25	50.85	-120.42	Canada	0.3	1150	2.5	300	Brunisolic Gray Luvisol	Douglas fir, Subalpine fir, Lodgepole pine	Dfb, Humid Continental warm summer	OM3	C0	0	A horizon	20.0	1.7	0.1	5.9	1.5	18.4
DC185B	DC185	IDF_BC_	British Columbia	DC	Forest Soil	Whole Community DNA	PCR	Amplicon	pyrotag library	454 GS FLX Titanium	European Nucleotide Archive	https://www.ebi.ac.uk/ena/data/search?query=PRJEB12501	ERS1040300	SAMEA3733151	ERX1298637	ERR1226425	16S rRNA	V1-V3	NA	AGAGTTTGATCMTGGCTCAG	GWATTACCGCGGCKGCTG	2010-06-25	50.85	-120.42	Canada	0.3	1150	2.5	300	Brunisolic Gray Luvisol	Douglas fir, Subalpine fir, Lodgepole pine	Dfb, Humid Continental warm summer	OM3	C0	0	A horizon	26.0	1.7	0.1	5.9	1.5	18.4
DC186B	DC186	IDF_BC_	British Columbia	DC	Forest Soil	Whole Community DNA	PCR	Amplicon	pyrotag library	454 GS FLX Titanium	European Nucleotide Archive	https://www.ebi.ac.uk/ena/data/search?query=PRJEB12501	ERS1040301	SAMEA3733152	ERX1298638	ERR1226426	16S rRNA	V1-V3	NA	AGAGTTTGATCMTGGCTCAG	GWATTACCGCGGCKGCTG	2010-06-25	50.85	-120.42	Canada	0.3	1150	2.5	300	Brunisolic Gray Luvisol	Douglas fir, Subalpine fir, Lodgepole pine	Dfb, Humid Continental warm summer	OM3	C0	0	A horizon	20.0	1.7	0.1	5.9	1.5	18.4
DC187B	DC187	IDF_BC_	British Columbia	DC	Forest Soil	Whole Community DNA	PCR	Amplicon	pyrotag library	454 GS FLX Titanium	European Nucleotide Archive	https://www.ebi.ac.uk/ena/data/search?query=PRJEB12501	ERS1040302	SAMEA3733153	ERX1298639	ERR1226427	16S rRNA	V1-V3	NA	AGAGTTTGATCMTGGCTCAG	GWATTACCGCGGCKGCTG	2010-06-25	50.85	-120.42	Canada	0.1	1150	2.5	300	Brunisolic Gray Luvisol	Douglas fir, Subalpine fir, Lodgepole pine	Dfb, Humid Continental warm summer	OM2	C0	0	O horizon	58.0	32.9	1.3	6.0	1.3	25.1
DC188B	DC188	IDF_BC_	British Columbia	DC	Forest Soil	Whole Community DNA	PCR	Amplicon	pyrotag library	454 GS FLX Titanium	European Nucleotide Archive	https://www.ebi.ac.uk/ena/data/search?query=PRJEB12501	ERS1040303	SAMEA3733154	ERX1298640	ERR1226428	16S rRNA	V1-V3	NA	AGAGTTTGATCMTGGCTCAG	GWATTACCGCGGCKGCTG	2010-06-25	50.85	-120.42	Canada	0.1	1150	2.5	300	Brunisolic Gray Luvisol	Douglas fir, Subalpine fir, Lodgepole pine	Dfb, Humid Continental warm summer	OM2	C0	0	O horizon	63.0	32.9	1.3	6.0	1.3	25.1
DC189B	DC189	IDF_BC_	British Columbia	DC	Forest Soil	Whole Community DNA	PCR	Amplicon	pyrotag library	454 GS FLX Titanium	European Nucleotide Archive	https://www.ebi.ac.uk/ena/data/search?query=PRJEB12501	ERS1040304	SAMEA3733155	ERX1298641	ERR1226429	16S rRNA	V1-V3	NA	AGAGTTTGATCMTGGCTCAG	GWATTACCGCGGCKGCTG	2010-06-25	50.85	-120.42	Canada	0.1	1150	2.5	300	Brunisolic Gray Luvisol	Douglas fir, Subalpine fir, Lodgepole pine	Dfb, Humid Continental warm summer	OM2	C0	0	O horizon	56.0	32.9	1.3	6.0	1.3	25.1
DC190B	DC190	IDF_BC_	British Columbia	DC	Forest Soil	Whole Community DNA	PCR	Amplicon	pyrotag library	454 GS FLX Titanium	European Nucleotide Archive	https://www.ebi.ac.uk/ena/data/search?query=PRJEB12501	ERS1040305	SAMEA3733156	ERX1298642	ERR1226430	16S rRNA	V1-V3	NA	AGAGTTTGATCMTGGCTCAG	GWATTACCGCGGCKGCTG	2010-06-25	50.85	-120.42	Canada	0.3	1150	2.5	300	Brunisolic Gray Luvisol	Douglas fir, Subalpine fir, Lodgepole pine	Dfb, Humid Continental warm summer	OM2	C0	0	A horizon	21.0	3.3	0.2	5.6	1.3	20.5
DC191B	DC191	IDF_BC_	British Columbia	DC	Forest Soil	Whole Community DNA	PCR	Amplicon	pyrotag library	454 GS FLX Titanium	European Nucleotide Archive	https://www.ebi.ac.uk/ena/data/search?query=PRJEB12501	ERS1040306	SAMEA3733157	ERX1298643	ERR1226431	16S rRNA	V1-V3	NA	AGAGTTTGATCMTGGCTCAG	GWATTACCGCGGCKGCTG	2010-06-25	50.85	-120.42	Canada	0.3	1150	2.5	300	Brunisolic Gray Luvisol	Douglas fir, Subalpine fir, Lodgepole pine	Dfb, Humid Continental warm summer	OM2	C0	0	A horizon	23.0	3.3	0.2	5.6	1.3	20.5
DC192B	DC192	IDF_BC_	British Columbia	DC	Forest Soil	Whole Community DNA	PCR	Amplicon	pyrotag library	454 GS FLX Titanium	European Nucleotide Archive	https://www.ebi.ac.uk/ena/data/search?query=PRJEB12501	ERS1040307	SAMEA3733158	ERX1298644	ERR1226432	16S rRNA	V1-V3	NA	AGAGTTTGATCMTGGCTCAG	GWATTACCGCGGCKGCTG	2010-06-25	50.85	-120.42	Canada	0.3	1150	2.5	300	Brunisolic Gray Luvisol	Douglas fir, Subalpine fir, Lodgepole pine	Dfb, Humid Continental warm summer	OM2	C0	0	A horizon	25.0	3.3	0.2	5.6	1.3	20.5
DC193B	DC193	IDF_BC_	British Columbia	DC	Forest Soil	Whole Community DNA	PCR	Amplicon	pyrotag library	454 GS FLX Titanium	European Nucleotide Archive	https://www.ebi.ac.uk/ena/data/search?query=PRJEB12501	ERS1040308	SAMEA3733159	ERX1298645	ERR1226433	16S rRNA	V1-V3	NA	AGAGTTTGATCMTGGCTCAG	GWATTACCGCGGCKGCTG	2010-06-25	50.85	-120.42	Canada	0.1	1150	2.5	300	Brunisolic Gray Luvisol	Douglas fir, Subalpine fir, Lodgepole pine	Dfb, Humid Continental warm summer	OM2	C1	0	O horizon	61.0	32.9	1.2	5.8	1.5	28.4
DC194B	DC194	IDF_BC_	British Columbia	DC	Forest Soil	Whole Community DNA	PCR	Amplicon	pyrotag library	454 GS FLX Titanium	European Nucleotide Archive	https://www.ebi.ac.uk/ena/data/search?query=PRJEB12501	ERS1040309	SAMEA3733160	ERX1298646	ERR1226434	16S rRNA	V1-V3	NA	AGAGTTTGATCMTGGCTCAG	GWATTACCGCGGCKGCTG	2010-06-25	50.85	-120.42	Canada	0.1	1150	2.5	300	Brunisolic Gray Luvisol	Douglas fir, Subalpine fir, Lodgepole pine	Dfb, Humid Continental warm summer	OM2	C1	0	O horizon	63.0	32.9	1.2	5.8	1.5	28.4
DC195B	DC195	IDF_BC_	British Columbia	DC	Forest Soil	Whole Community DNA	PCR	Amplicon	pyrotag library	454 GS FLX Titanium	European Nucleotide Archive	https://www.ebi.ac.uk/ena/data/search?query=PRJEB12501	ERS1040310	SAMEA3733161	ERX1298647	ERR1226435	16S rRNA	V1-V3	NA	AGAGTTTGATCMTGGCTCAG	GWATTACCGCGGCKGCTG	2010-06-25	50.85	-120.42	Canada	0.1	1150	2.5	300	Brunisolic Gray Luvisol	Douglas fir, Subalpine fir, Lodgepole pine	Dfb, Humid Continental warm summer	OM2	C1	0	O horizon	57.0	32.9	1.2	5.8	1.5	28.4
DC196B	DC196	IDF_BC_	British Columbia	DC	Forest Soil	Whole Community DNA	PCR	Amplicon	pyrotag library	454 GS FLX Titanium	European Nucleotide Archive	https://www.ebi.ac.uk/ena/data/search?query=PRJEB12501	ERS1040311	SAMEA3733162	ERX1298648	ERR1226436	16S rRNA	V1-V3	NA	AGAGTTTGATCMTGGCTCAG	GWATTACCGCGGCKGCTG	2010-06-25	50.85	-120.42	Canada	0.3	1150	2.5	300	Brunisolic Gray Luvisol	Douglas fir, Subalpine fir, Lodgepole pine	Dfb, Humid Continental warm summer	OM2	C1	0	A horizon	29.0	2.1	0.1	5.6	1.5	20.5
DC197B	DC197	IDF_BC_	British Columbia	DC	Forest Soil	Whole Community DNA	PCR	Amplicon	pyrotag library	454 GS FLX Titanium	European Nucleotide Archive	https://www.ebi.ac.uk/ena/data/search?query=PRJEB12501	ERS1040312	SAMEA3733163	ERX1298649	ERR1226437	16S rRNA	V1-V3	NA	AGAGTTTGATCMTGGCTCAG	GWATTACCGCGGCKGCTG	2010-06-25	50.85	-120.42	Canada	0.3	1150	2.5	300	Brunisolic Gray Luvisol	Douglas fir, Subalpine fir, Lodgepole pine	Dfb, Humid Continental warm summer	OM2	C1	0	A horizon	27.0	2.1	0.1	5.6	1.5	20.5
DC198B	DC198	IDF_BC_	British Columbia	DC	Forest Soil	Whole Community DNA	PCR	Amplicon	pyrotag library	454 GS FLX Titanium	European Nucleotide Archive	https://www.ebi.ac.uk/ena/data/search?query=PRJEB12501	ERS1040313	SAMEA3733164	ERX1298650	ERR1226438	16S rRNA	V1-V3	NA	AGAGTTTGATCMTGGCTCAG	GWATTACCGCGGCKGCTG	2010-06-25	50.85	-120.42	Canada	0.3	1150	2.5	300	Brunisolic Gray Luvisol	Douglas fir, Subalpine fir, Lodgepole pine	Dfb, Humid Continental warm summer	OM2	C1	0	A horizon	21.0	2.1	0.1	5.6	1.5	20.5
DC199B	DC199	IDF_BC_	British Columbia	DC	Forest Soil	Whole Community DNA	PCR	Amplicon	pyrotag library	454 GS FLX Titanium	European Nucleotide Archive	https://www.ebi.ac.uk/ena/data/search?query=PRJEB12501	ERS1040314	SAMEA3733165	ERX1298651	ERR1226439	16S rRNA	V1-V3	NA	AGAGTTTGATCMTGGCTCAG	GWATTACCGCGGCKGCTG	2010-06-25	50.85	-120.42	Canada	0.1	1150	2.5	300	Brunisolic Gray Luvisol	Douglas fir, Subalpine fir, Lodgepole pine	Dfb, Humid Continental warm summer	OM2	C2	0	O horizon	58.0	30.1	1.0	5.8	1.5	29.3
DC200B	DC200	IDF_BC_	British Columbia	DC	Forest Soil	Whole Community DNA	PCR	Amplicon	pyrotag library	454 GS FLX Titanium	European Nucleotide Archive	https://www.ebi.ac.uk/ena/data/search?query=PRJEB12501	ERS1040315	SAMEA3733166	ERX1298652	ERR1226440	16S rRNA	V1-V3	NA	AGAGTTTGATCMTGGCTCAG	GWATTACCGCGGCKGCTG	2010-06-25	50.85	-120.42	Canada	0.1	1150	2.5	300	Brunisolic Gray Luvisol	Douglas fir, Subalpine fir, Lodgepole pine	Dfb, Humid Continental warm summer	OM2	C2	0	O horizon	53.0	30.1	1.0	5.8	1.5	29.3
DC201B	DC201	IDF_BC_	British Columbia	DC	Forest Soil	Whole Community DNA	PCR	Amplicon	pyrotag library	454 GS FLX Titanium	European Nucleotide Archive	https://www.ebi.ac.uk/ena/data/search?query=PRJEB12501	ERS1040316	SAMEA3733167	ERX1298653	ERR1226441	16S rRNA	V1-V3	NA	AGAGTTTGATCMTGGCTCAG	GWATTACCGCGGCKGCTG	2010-06-25	50.85	-120.42	Canada	0.1	1150	2.5	300	Brunisolic Gray Luvisol	Douglas fir, Subalpine fir, Lodgepole pine	Dfb, Humid Continental warm summer	OM2	C2	0	O horizon	59.0	30.1	1.0	5.8	1.5	29.3
DC202B	DC202	IDF_BC_	British Columbia	DC	Forest Soil	Whole Community DNA	PCR	Amplicon	pyrotag library	454 GS FLX Titanium	European Nucleotide Archive	https://www.ebi.ac.uk/ena/data/search?query=PRJEB12501	ERS1040317	SAMEA3733168	ERX1298654	ERR1226442	16S rRNA	V1-V3	NA	AGAGTTTGATCMTGGCTCAG	GWATTACCGCGGCKGCTG	2010-06-25	50.85	-120.42	Canada	0.3	1150	2.5	300	Brunisolic Gray Luvisol	Douglas fir, Subalpine fir, Lodgepole pine	Dfb, Humid Continental warm summer	OM2	C2	0	A horizon	24.0	2.4	0.1	5.8	1.5	18.5
DC203B	DC203	IDF_BC_	British Columbia	DC	Forest Soil	Whole Community DNA	PCR	Amplicon	pyrotag library	454 GS FLX Titanium	European Nucleotide Archive	https://www.ebi.ac.uk/ena/data/search?query=PRJEB12501	ERS1040318	SAMEA3733169	ERX1298655	ERR1226443	16S rRNA	V1-V3	NA	AGAGTTTGATCMTGGCTCAG	GWATTACCGCGGCKGCTG	2010-06-25	50.85	-120.42	Canada	0.3	1150	2.5	300	Brunisolic Gray Luvisol	Douglas fir, Subalpine fir, Lodgepole pine	Dfb, Humid Continental warm summer	OM2	C2	0	A horizon	33.0	2.4	0.1	5.8	1.5	18.5
DC204B	DC204	IDF_BC_	British Columbia	DC	Forest Soil	Whole Community DNA	PCR	Amplicon	pyrotag library	454 GS FLX Titanium	European Nucleotide Archive	https://www.ebi.ac.uk/ena/data/search?query=PRJEB12501	ERS1040319	SAMEA3733170	ERX1298656	ERR1226444	16S rRNA	V1-V3	NA	AGAGTTTGATCMTGGCTCAG	GWATTACCGCGGCKGCTG	2010-06-25	50.85	-120.42	Canada	0.3	1150	2.5	300	Brunisolic Gray Luvisol	Douglas fir, Subalpine fir, Lodgepole pine	Dfb, Humid Continental warm summer	OM2	C2	0	A horizon	30.0	2.4	0.1	5.8	1.5	18.5
DC205B	DC205	IDF_BC_	British Columbia	DC	Forest Soil	Whole Community DNA	PCR	Amplicon	pyrotag library	454 GS FLX Titanium	European Nucleotide Archive	https://www.ebi.ac.uk/ena/data/search?query=PRJEB12501	ERS1040320	SAMEA3733171	ERX1298657	ERR1226445	16S rRNA	V1-V3	NA	AGAGTTTGATCMTGGCTCAG	GWATTACCGCGGCKGCTG	2010-06-25	50.85	-120.42	Canada	0.1	1150	2.5	300	Brunisolic Gray Luvisol	Douglas fir, Subalpine fir, Lodgepole pine	Dfb, Humid Continental warm summer	OM1	C0	0	O horizon	60.0	38.7	1.3	5.6	1.4	29.5
DC206B	DC206	IDF_BC_	British Columbia	DC	Forest Soil	Whole Community DNA	PCR	Amplicon	pyrotag library	454 GS FLX Titanium	European Nucleotide Archive	https://www.ebi.ac.uk/ena/data/search?query=PRJEB12501	ERS1040321	SAMEA3733172	ERX1298658	ERR1226446	16S rRNA	V1-V3	NA	AGAGTTTGATCMTGGCTCAG	GWATTACCGCGGCKGCTG	2010-06-25	50.85	-120.42	Canada	0.1	1150	2.5	300	Brunisolic Gray Luvisol	Douglas fir, Subalpine fir, Lodgepole pine	Dfb, Humid Continental warm summer	OM1	C0	0	O horizon	60.0	38.7	1.3	5.6	1.4	29.5
DC207B	DC207	IDF_BC_	British Columbia	DC	Forest Soil	Whole Community DNA	PCR	Amplicon	pyrotag library	454 GS FLX Titanium	European Nucleotide Archive	https://www.ebi.ac.uk/ena/data/search?query=PRJEB12501	ERS1040322	SAMEA3733173	ERX1298659	ERR1226447	16S rRNA	V1-V3	NA	AGAGTTTGATCMTGGCTCAG	GWATTACCGCGGCKGCTG	2010-06-25	50.85	-120.42	Canada	0.1	1150	2.5	300	Brunisolic Gray Luvisol	Douglas fir, Subalpine fir, Lodgepole pine	Dfb, Humid Continental warm summer	OM1	C0	0	O horizon	58.0	38.7	1.3	5.6	1.4	29.5
DC208B	DC208	IDF_BC_	British Columbia	DC	Forest Soil	Whole Community DNA	PCR	Amplicon	pyrotag library	454 GS FLX Titanium	European Nucleotide Archive	https://www.ebi.ac.uk/ena/data/search?query=PRJEB12501	ERS1040323	SAMEA3733174	ERX1298660	ERR1226448	16S rRNA	V1-V3	NA	AGAGTTTGATCMTGGCTCAG	GWATTACCGCGGCKGCTG	2010-06-25	50.85	-120.42	Canada	0.3	1150	2.5	300	Brunisolic Gray Luvisol	Douglas fir, Subalpine fir, Lodgepole pine	Dfb, Humid Continental warm summer	OM1	C0	0	A horizon	28.0	2.3	0.1	5.2	1.4	20.5
DC209B	DC209	IDF_BC_	British Columbia	DC	Forest Soil	Whole Community DNA	PCR	Amplicon	pyrotag library	454 GS FLX Titanium	European Nucleotide Archive	https://www.ebi.ac.uk/ena/data/search?query=PRJEB12501	ERS1040324	SAMEA3733175	ERX1298661	ERR1226449	16S rRNA	V1-V3	NA	AGAGTTTGATCMTGGCTCAG	GWATTACCGCGGCKGCTG	2010-06-25	50.85	-120.42	Canada	0.3	1150	2.5	300	Brunisolic Gray Luvisol	Douglas fir, Subalpine fir, Lodgepole pine	Dfb, Humid Continental warm summer	OM1	C0	0	A horizon	26.0	2.3	0.1	5.2	1.4	20.5
DC210B	DC210	IDF_BC_	British Columbia	DC	Forest Soil	Whole Community DNA	PCR	Amplicon	pyrotag library	454 GS FLX Titanium	European Nucleotide Archive	https://www.ebi.ac.uk/ena/data/search?query=PRJEB12501	ERS1040325	SAMEA3733176	ERX1298662	ERR1226450	16S rRNA	V1-V3	NA	AGAGTTTGATCMTGGCTCAG	GWATTACCGCGGCKGCTG	2010-06-25	50.85	-120.42	Canada	0.3	1150	2.5	300	Brunisolic Gray Luvisol	Douglas fir, Subalpine fir, Lodgepole pine	Dfb, Humid Continental warm summer	OM1	C0	0	A horizon	24.0	2.3	0.1	5.2	1.4	20.5
DC214B	DC214	IDF_BC_	British Columbia	DC	Forest Soil	Whole Community DNA	PCR	Amplicon	pyrotag library	454 GS FLX Titanium	European Nucleotide Archive	https://www.ebi.ac.uk/ena/data/search?query=PRJEB12501	ERS1040326	SAMEA3733177	ERX1298663	ERR1226451	16S rRNA	V1-V3	NA	AGAGTTTGATCMTGGCTCAG	GWATTACCGCGGCKGCTG	2010-06-25	50.85	-120.42	Canada	0.3	1150	2.5	300	Brunisolic Gray Luvisol	Douglas fir, Subalpine fir, Lodgepole pine	Dfb, Humid Continental warm summer	OM3	C1	0	A horizon	17.0	1.8	0.1	5.7	1.7	19.7
DC215B	DC215	IDF_BC_	British Columbia	DC	Forest Soil	Whole Community DNA	PCR	Amplicon	pyrotag library	454 GS FLX Titanium	European Nucleotide Archive	https://www.ebi.ac.uk/ena/data/search?query=PRJEB12501	ERS1040327	SAMEA3733178	ERX1298664	ERR1226452	16S rRNA	V1-V3	NA	AGAGTTTGATCMTGGCTCAG	GWATTACCGCGGCKGCTG	2010-06-25	50.85	-120.42	Canada	0.3	1150	2.5	300	Brunisolic Gray Luvisol	Douglas fir, Subalpine fir, Lodgepole pine	Dfb, Humid Continental warm summer	OM3	C1	0	A horizon	18.0	1.8	0.1	5.7	1.7	19.7
DC216B	DC216	IDF_BC_	British Columbia	DC	Forest Soil	Whole Community DNA	PCR	Amplicon	pyrotag library	454 GS FLX Titanium	European Nucleotide Archive	https://www.ebi.ac.uk/ena/data/search?query=PRJEB12501	ERS1040328	SAMEA3733179	ERX1298665	ERR1226453	16S rRNA	V1-V3	NA	AGAGTTTGATCMTGGCTCAG	GWATTACCGCGGCKGCTG	2010-06-25	50.85	-120.42	Canada	0.3	1150	2.5	300	Brunisolic Gray Luvisol	Douglas fir, Subalpine fir, Lodgepole pine	Dfb, Humid Continental warm summer	OM3	C1	0	A horizon	25.0	1.8	0.1	5.7	1.7	19.7
DC220B	DC220	IDF_BC_	British Columbia	DC	Forest Soil	Whole Community DNA	PCR	Amplicon	pyrotag library	454 GS FLX Titanium	European Nucleotide Archive	https://www.ebi.ac.uk/ena/data/search?query=PRJEB12501	ERS1040329	SAMEA3733180	ERX1298666	ERR1226454	16S rRNA	V1-V3	NA	AGAGTTTGATCMTGGCTCAG	GWATTACCGCGGCKGCTG	2010-06-25	50.85	-120.42	Canada	0.3	1150	2.5	300	Brunisolic Gray Luvisol	Douglas fir, Subalpine fir, Lodgepole pine	Dfb, Humid Continental warm summer	OM3	C2	0	A horizon	25.0	2.0	0.1	5.8	1.7	18.0
DC222B	DC222	IDF_BC_	British Columbia	DC	Forest Soil	Whole Community DNA	PCR	Amplicon	pyrotag library	454 GS FLX Titanium	European Nucleotide Archive	https://www.ebi.ac.uk/ena/data/search?query=PRJEB12501	ERS1040330	SAMEA3733181	ERX1298667	ERR1226455	16S rRNA	V1-V3	NA	AGAGTTTGATCMTGGCTCAG	GWATTACCGCGGCKGCTG	2010-06-25	50.85	-120.42	Canada	0.3	1150	2.5	300	Brunisolic Gray Luvisol	Douglas fir, Subalpine fir, Lodgepole pine	Dfb, Humid Continental warm summer	OM3	C2	0	A horizon	21.0	2.0	0.1	5.8	1.7	18.0
DC223B	DC223	IDF_BC_	British Columbia	DC	Forest Soil	Whole Community DNA	PCR	Amplicon	pyrotag library	454 GS FLX Titanium	European Nucleotide Archive	https://www.ebi.ac.uk/ena/data/search?query=PRJEB12501	ERS1040331	SAMEA3733182	ERX1298668	ERR1226456	16S rRNA	V1-V3	NA	AGAGTTTGATCMTGGCTCAG	GWATTACCGCGGCKGCTG	2010-06-25	50.85	-120.42	Canada	0.1	1150	2.5	300	Brunisolic Gray Luvisol	Douglas fir, Subalpine fir, Lodgepole pine	Dfb, Humid Continental warm summer	OM1	C2	0	O horizon	63.0	38.8	1.0	5.5	1.5	39.2
DC224B	DC224	IDF_BC_	British Columbia	DC	Forest Soil	Whole Community DNA	PCR	Amplicon	pyrotag library	454 GS FLX Titanium	European Nucleotide Archive	https://www.ebi.ac.uk/ena/data/search?query=PRJEB12501	ERS1040332	SAMEA3733183	ERX1298669	ERR1226457	16S rRNA	V1-V3	NA	AGAGTTTGATCMTGGCTCAG	GWATTACCGCGGCKGCTG	2010-06-25	50.85	-120.42	Canada	0.1	1150	2.5	300	Brunisolic Gray Luvisol	Douglas fir, Subalpine fir, Lodgepole pine	Dfb, Humid Continental warm summer	OM1	C2	0	O horizon	57.0	38.8	1.0	5.5	1.5	39.2
DC225B	DC225	IDF_BC_	British Columbia	DC	Forest Soil	Whole Community DNA	PCR	Amplicon	pyrotag library	454 GS FLX Titanium	European Nucleotide Archive	https://www.ebi.ac.uk/ena/data/search?query=PRJEB12501	ERS1040333	SAMEA3733184	ERX1298670	ERR1226458	16S rRNA	V1-V3	NA	AGAGTTTGATCMTGGCTCAG	GWATTACCGCGGCKGCTG	2010-06-25	50.85	-120.42	Canada	0.1	1150	2.5	300	Brunisolic Gray Luvisol	Douglas fir, Subalpine fir, Lodgepole pine	Dfb, Humid Continental warm summer	OM1	C2	0	O horizon	63.0	38.8	1.0	5.5	1.5	39.2
DC226B	DC226	IDF_BC_	British Columbia	DC	Forest Soil	Whole Community DNA	PCR	Amplicon	pyrotag library	454 GS FLX Titanium	European Nucleotide Archive	https://www.ebi.ac.uk/ena/data/search?query=PRJEB12501	ERS1040334	SAMEA3733185	ERX1298671	ERR1226459	16S rRNA	V1-V3	NA	AGAGTTTGATCMTGGCTCAG	GWATTACCGCGGCKGCTG	2010-06-25	50.85	-120.42	Canada	0.3	1150	2.5	300	Brunisolic Gray Luvisol	Douglas fir, Subalpine fir, Lodgepole pine	Dfb, Humid Continental warm summer	OM1	C2	0	A horizon	27.0	2.6	0.1	5.5	1.5	21.4
DC227B	DC227	IDF_BC_	British Columbia	DC	Forest Soil	Whole Community DNA	PCR	Amplicon	pyrotag library	454 GS FLX Titanium	European Nucleotide Archive	https://www.ebi.ac.uk/ena/data/search?query=PRJEB12501	ERS1040335	SAMEA3733186	ERX1298672	ERR1226460	16S rRNA	V1-V3	NA	AGAGTTTGATCMTGGCTCAG	GWATTACCGCGGCKGCTG	2010-06-25	50.85	-120.42	Canada	0.3	1150	2.5	300	Brunisolic Gray Luvisol	Douglas fir, Subalpine fir, Lodgepole pine	Dfb, Humid Continental warm summer	OM1	C2	0	A horizon	26.0	2.6	0.1	5.5	1.5	21.4
DC228B	DC228	IDF_BC_	British Columbia	DC	Forest Soil	Whole Community DNA	PCR	Amplicon	pyrotag library	454 GS FLX Titanium	European Nucleotide Archive	https://www.ebi.ac.uk/ena/data/search?query=PRJEB12501	ERS1040336	SAMEA3733187	ERX1298673	ERR1226461	16S rRNA	V1-V3	NA	AGAGTTTGATCMTGGCTCAG	GWATTACCGCGGCKGCTG	2010-06-25	50.85	-120.42	Canada	0.3	1150	2.5	300	Brunisolic Gray Luvisol	Douglas fir, Subalpine fir, Lodgepole pine	Dfb, Humid Continental warm summer	OM1	C2	0	A horizon	27.0	2.6	0.1	5.5	1.5	21.4
DC229B	DC229	IDF_BC_	British Columbia	DC	Forest Soil	Whole Community DNA	PCR	Amplicon	pyrotag library	454 GS FLX Titanium	European Nucleotide Archive	https://www.ebi.ac.uk/ena/data/search?query=PRJEB12501	ERS1040337	SAMEA3733188	ERX1298674	ERR1226462	16S rRNA	V1-V3	NA	AGAGTTTGATCMTGGCTCAG	GWATTACCGCGGCKGCTG	2010-06-25	50.85	-120.42	Canada	0.1	1150	2.5	300	Brunisolic Gray Luvisol	Douglas fir, Subalpine fir, Lodgepole pine	Dfb, Humid Continental warm summer	OM1	C1	0	O horizon	60.0	36.7	1.1	5.9	1.4	33.3
DC230B	DC230	IDF_BC_	British Columbia	DC	Forest Soil	Whole Community DNA	PCR	Amplicon	pyrotag library	454 GS FLX Titanium	European Nucleotide Archive	https://www.ebi.ac.uk/ena/data/search?query=PRJEB12501	ERS1040338	SAMEA3733189	ERX1298675	ERR1226463	16S rRNA	V1-V3	NA	AGAGTTTGATCMTGGCTCAG	GWATTACCGCGGCKGCTG	2010-06-25	50.85	-120.42	Canada	0.1	1150	2.5	300	Brunisolic Gray Luvisol	Douglas fir, Subalpine fir, Lodgepole pine	Dfb, Humid Continental warm summer	OM1	C1	0	O horizon	60.0	36.7	1.1	5.9	1.4	33.3
DC231B	DC231	IDF_BC_	British Columbia	DC	Forest Soil	Whole Community DNA	PCR	Amplicon	pyrotag library	454 GS FLX Titanium	European Nucleotide Archive	https://www.ebi.ac.uk/ena/data/search?query=PRJEB12501	ERS1040339	SAMEA3733190	ERX1298676	ERR1226464	16S rRNA	V1-V3	NA	AGAGTTTGATCMTGGCTCAG	GWATTACCGCGGCKGCTG	2010-06-25	50.85	-120.42	Canada	0.1	1150	2.5	300	Brunisolic Gray Luvisol	Douglas fir, Subalpine fir, Lodgepole pine	Dfb, Humid Continental warm summer	OM1	C1	0	O horizon	52.0	36.7	1.1	5.9	1.4	33.3
DC232B	DC232	IDF_BC_	British Columbia	DC	Forest Soil	Whole Community DNA	PCR	Amplicon	pyrotag library	454 GS FLX Titanium	European Nucleotide Archive	https://www.ebi.ac.uk/ena/data/search?query=PRJEB12501	ERS1040340	SAMEA3733191	ERX1298677	ERR1226465	16S rRNA	V1-V3	NA	AGAGTTTGATCMTGGCTCAG	GWATTACCGCGGCKGCTG	2010-06-25	50.85	-120.42	Canada	0.3	1150	2.5	300	Brunisolic Gray Luvisol	Douglas fir, Subalpine fir, Lodgepole pine	Dfb, Humid Continental warm summer	OM1	C1	0	A horizon	29.0	2.8	0.1	5.9	1.4	19.9
DC233B	DC233	IDF_BC_	British Columbia	DC	Forest Soil	Whole Community DNA	PCR	Amplicon	pyrotag library	454 GS FLX Titanium	European Nucleotide Archive	https://www.ebi.ac.uk/ena/data/search?query=PRJEB12501	ERS1040341	SAMEA3733192	ERX1298678	ERR1226466	16S rRNA	V1-V3	NA	AGAGTTTGATCMTGGCTCAG	GWATTACCGCGGCKGCTG	2010-06-25	50.85	-120.42	Canada	0.3	1150	2.5	300	Brunisolic Gray Luvisol	Douglas fir, Subalpine fir, Lodgepole pine	Dfb, Humid Continental warm summer	OM1	C1	0	A horizon	29.0	2.8	0.1	5.9	1.4	19.9
DC234B	DC234	IDF_BC_	British Columbia	DC	Forest Soil	Whole Community DNA	PCR	Amplicon	pyrotag library	454 GS FLX Titanium	European Nucleotide Archive	https://www.ebi.ac.uk/ena/data/search?query=PRJEB12501	ERS1040342	SAMEA3733193	ERX1298679	ERR1226467	16S rRNA	V1-V3	NA	AGAGTTTGATCMTGGCTCAG	GWATTACCGCGGCKGCTG	2010-06-25	50.85	-120.42	Canada	0.3	1150	2.5	300	Brunisolic Gray Luvisol	Douglas fir, Subalpine fir, Lodgepole pine	Dfb, Humid Continental warm summer	OM1	C1	0	A horizon	29.0	2.8	0.1	5.9	1.4	19.9
DC235B	DC235	IDF_BC_	British Columbia	DC	Forest Soil	Whole Community DNA	PCR	Amplicon	pyrotag library	454 GS FLX Titanium	European Nucleotide Archive	https://www.ebi.ac.uk/ena/data/search?query=PRJEB12501	ERS1040343	SAMEA3733194	ERX1298680	ERR1226468	16S rRNA	V1-V3	NA	AGAGTTTGATCMTGGCTCAG	GWATTACCGCGGCKGCTG	2010-06-25	50.85	-120.42	Canada	0.1	1150	2.5	300	Brunisolic Gray Luvisol	Douglas fir, Subalpine fir, Lodgepole pine	Dfb, Humid Continental warm summer	REF	REF	0	O horizon	63.0	44.3	1.1	5.0	NA	41.4
DC236B	DC236	IDF_BC_	British Columbia	DC	Forest Soil	Whole Community DNA	PCR	Amplicon	pyrotag library	454 GS FLX Titanium	European Nucleotide Archive	https://www.ebi.ac.uk/ena/data/search?query=PRJEB12501	ERS1040344	SAMEA3733195	ERX1298681	ERR1226469	16S rRNA	V1-V3	NA	AGAGTTTGATCMTGGCTCAG	GWATTACCGCGGCKGCTG	2010-06-25	50.85	-120.42	Canada	0.1	1150	2.5	300	Brunisolic Gray Luvisol	Douglas fir, Subalpine fir, Lodgepole pine	Dfb, Humid Continental warm summer	REF	REF	0	O horizon	64.0	44.3	1.1	5.0	NA	41.4
DC237B	DC237	IDF_BC_	British Columbia	DC	Forest Soil	Whole Community DNA	PCR	Amplicon	pyrotag library	454 GS FLX Titanium	European Nucleotide Archive	https://www.ebi.ac.uk/ena/data/search?query=PRJEB12501	ERS1040345	SAMEA3733196	ERX1298682	ERR1226470	16S rRNA	V1-V3	NA	AGAGTTTGATCMTGGCTCAG	GWATTACCGCGGCKGCTG	2010-06-25	50.85	-120.42	Canada	0.1	1150	2.5	300	Brunisolic Gray Luvisol	Douglas fir, Subalpine fir, Lodgepole pine	Dfb, Humid Continental warm summer	REF	REF	0	O horizon	60.0	44.3	1.1	5.0	NA	41.4
DC238B	DC238	IDF_BC_	British Columbia	DC	Forest Soil	Whole Community DNA	PCR	Amplicon	pyrotag library	454 GS FLX Titanium	European Nucleotide Archive	https://www.ebi.ac.uk/ena/data/search?query=PRJEB12501	ERS1040346	SAMEA3733197	ERX1298683	ERR1226471	16S rRNA	V1-V3	NA	AGAGTTTGATCMTGGCTCAG	GWATTACCGCGGCKGCTG	2010-06-25	50.85	-120.42	Canada	0.3	1150	2.5	300	Brunisolic Gray Luvisol	Douglas fir, Subalpine fir, Lodgepole pine	Dfb, Humid Continental warm summer	REF	REF	0	A horizon	25.0	2.3	0.1	5.2	NA	23.4
DC239B	DC239	IDF_BC_	British Columbia	DC	Forest Soil	Whole Community DNA	PCR	Amplicon	pyrotag library	454 GS FLX Titanium	European Nucleotide Archive	https://www.ebi.ac.uk/ena/data/search?query=PRJEB12501	ERS1040347	SAMEA3733198	ERX1298684	ERR1226472	16S rRNA	V1-V3	NA	AGAGTTTGATCMTGGCTCAG	GWATTACCGCGGCKGCTG	2010-06-25	50.85	-120.42	Canada	0.3	1150	2.5	300	Brunisolic Gray Luvisol	Douglas fir, Subalpine fir, Lodgepole pine	Dfb, Humid Continental warm summer	REF	REF	0	A horizon	27.0	2.3	0.1	5.2	NA	23.4
DC240B	DC240	IDF_BC_	British Columbia	DC	Forest Soil	Whole Community DNA	PCR	Amplicon	pyrotag library	454 GS FLX Titanium	European Nucleotide Archive	https://www.ebi.ac.uk/ena/data/search?query=PRJEB12501	ERS1040348	SAMEA3733199	ERX1298685	ERR1226473	16S rRNA	V1-V3	NA	AGAGTTTGATCMTGGCTCAG	GWATTACCGCGGCKGCTG	2010-06-25	50.85	-120.42	Canada	0.3	1150	2.5	300	Brunisolic Gray Luvisol	Douglas fir, Subalpine fir, Lodgepole pine	Dfb, Humid Continental warm summer	REF	REF	0	A horizon	34.0	2.3	0.1	5.2	NA	23.4
LL001B	LL001	SBS_BC_	British Columbia	LL	Forest Soil	Whole Community DNA	PCR	Amplicon	pyrotag library	454 GS FLX Titanium	European Nucleotide Archive	https://www.ebi.ac.uk/ena/data/search?query=PRJEB12501	ERS1040349	SAMEA3733200	ERX1298686	ERR1226474	16S rRNA	V1-V3	NA	AGAGTTTGATCMTGGCTCAG	GWATTACCGCGGCKGCTG	2008-07-09	54.35	-122.61	Canada	0.1	780	3.3	146-193	Orthic Humo-Ferric Podzol, Gleyed Eluviated Dystric Brunisol	Subalpine fir, Douglas fir, Interior Spruce	Dfc, Boreal cool summer	OM1	C0	0	O horizon	42.0	24.3	0.8	4.7	1.4	30.8
LL002B	LL002	SBS_BC_	British Columbia	LL	Forest Soil	Whole Community DNA	PCR	Amplicon	pyrotag library	454 GS FLX Titanium	European Nucleotide Archive	https://www.ebi.ac.uk/ena/data/search?query=PRJEB12501	ERS1040350	SAMEA3733201	ERX1298687	ERR1226475	16S rRNA	V1-V3	NA	AGAGTTTGATCMTGGCTCAG	GWATTACCGCGGCKGCTG	2008-07-09	54.35	-122.61	Canada	0.1	780	3.3	146-193	Orthic Humo-Ferric Podzol, Gleyed Eluviated Dystric Brunisol	Subalpine fir, Douglas fir, Interior Spruce	Dfc, Boreal cool summer	OM1	C0	0	O horizon	48.0	24.3	0.8	4.7	1.4	30.8
LL003B	LL003	SBS_BC_	British Columbia	LL	Forest Soil	Whole Community DNA	PCR	Amplicon	pyrotag library	454 GS FLX Titanium	European Nucleotide Archive	https://www.ebi.ac.uk/ena/data/search?query=PRJEB12501	ERS1040351	SAMEA3733202	ERX1298688	ERR1226476	16S rRNA	V1-V3	NA	AGAGTTTGATCMTGGCTCAG	GWATTACCGCGGCKGCTG	2008-07-09	54.35	-122.61	Canada	0.1	780	3.3	146-193	Orthic Humo-Ferric Podzol, Gleyed Eluviated Dystric Brunisol	Subalpine fir, Douglas fir, Interior Spruce	Dfc, Boreal cool summer	OM1	C0	0	O horizon	40.0	24.3	0.8	4.7	1.4	30.8
LL004B	LL004	SBS_BC_	British Columbia	LL	Forest Soil	Whole Community DNA	PCR	Amplicon	pyrotag library	454 GS FLX Titanium	European Nucleotide Archive	https://www.ebi.ac.uk/ena/data/search?query=PRJEB12501	ERS1040352	SAMEA3733203	ERX1298689	ERR1226477	16S rRNA	V1-V3	NA	AGAGTTTGATCMTGGCTCAG	GWATTACCGCGGCKGCTG	2008-07-09	54.35	-122.61	Canada	0.3	780	3.3	146-193	Orthic Humo-Ferric Podzol, Gleyed Eluviated Dystric Brunisol	Subalpine fir, Douglas fir, Interior Spruce	Dfc, Boreal cool summer	OM1	C0	0	A horizon	22.0	1.6	0.1	4.7	1.4	22.1
LL005B	LL005	SBS_BC_	British Columbia	LL	Forest Soil	Whole Community DNA	PCR	Amplicon	pyrotag library	454 GS FLX Titanium	European Nucleotide Archive	https://www.ebi.ac.uk/ena/data/search?query=PRJEB12501	ERS1040353	SAMEA3733204	ERX1298690	ERR1226478	16S rRNA	V1-V3	NA	AGAGTTTGATCMTGGCTCAG	GWATTACCGCGGCKGCTG	2008-07-09	54.35	-122.61	Canada	0.3	780	3.3	146-193	Orthic Humo-Ferric Podzol, Gleyed Eluviated Dystric Brunisol	Subalpine fir, Douglas fir, Interior Spruce	Dfc, Boreal cool summer	OM1	C0	0	A horizon	20.0	1.6	0.1	4.7	1.4	22.1
LL006B	LL006	SBS_BC_	British Columbia	LL	Forest Soil	Whole Community DNA	PCR	Amplicon	pyrotag library	454 GS FLX Titanium	European Nucleotide Archive	https://www.ebi.ac.uk/ena/data/search?query=PRJEB12501	ERS1040354	SAMEA3733205	ERX1298691	ERR1226479	16S rRNA	V1-V3	NA	AGAGTTTGATCMTGGCTCAG	GWATTACCGCGGCKGCTG	2008-07-09	54.35	-122.61	Canada	0.3	780	3.3	146-193	Orthic Humo-Ferric Podzol, Gleyed Eluviated Dystric Brunisol	Subalpine fir, Douglas fir, Interior Spruce	Dfc, Boreal cool summer	OM1	C0	0	A horizon	21.0	1.6	0.1	4.7	1.4	22.1
LL007B	LL007	SBS_BC_	British Columbia	LL	Forest Soil	Whole Community DNA	PCR	Amplicon	pyrotag library	454 GS FLX Titanium	European Nucleotide Archive	https://www.ebi.ac.uk/ena/data/search?query=PRJEB12501	ERS1040355	SAMEA3733206	ERX1298692	ERR1226480	16S rRNA	V1-V3	NA	AGAGTTTGATCMTGGCTCAG	GWATTACCGCGGCKGCTG	2008-07-09	54.35	-122.61	Canada	0.1	780	3.3	146-193	Orthic Humo-Ferric Podzol, Gleyed Eluviated Dystric Brunisol	Subalpine fir, Douglas fir, Interior Spruce	Dfc, Boreal cool summer	OM2	C0	0	O horizon	48.0	27.6	0.8	4.5	1.6	33.7
LL008B	LL008	SBS_BC_	British Columbia	LL	Forest Soil	Whole Community DNA	PCR	Amplicon	pyrotag library	454 GS FLX Titanium	European Nucleotide Archive	https://www.ebi.ac.uk/ena/data/search?query=PRJEB12501	ERS1040356	SAMEA3733207	ERX1298693	ERR1226481	16S rRNA	V1-V3	NA	AGAGTTTGATCMTGGCTCAG	GWATTACCGCGGCKGCTG	2008-07-09	54.35	-122.61	Canada	0.1	780	3.3	146-193	Orthic Humo-Ferric Podzol, Gleyed Eluviated Dystric Brunisol	Subalpine fir, Douglas fir, Interior Spruce	Dfc, Boreal cool summer	OM2	C0	0	O horizon	49.0	27.6	0.8	4.5	1.6	33.7
LL009B	LL009	SBS_BC_	British Columbia	LL	Forest Soil	Whole Community DNA	PCR	Amplicon	pyrotag library	454 GS FLX Titanium	European Nucleotide Archive	https://www.ebi.ac.uk/ena/data/search?query=PRJEB12501	ERS1040357	SAMEA3733208	ERX1298694	ERR1226482	16S rRNA	V1-V3	NA	AGAGTTTGATCMTGGCTCAG	GWATTACCGCGGCKGCTG	2008-07-09	54.35	-122.61	Canada	0.1	780	3.3	146-193	Orthic Humo-Ferric Podzol, Gleyed Eluviated Dystric Brunisol	Subalpine fir, Douglas fir, Interior Spruce	Dfc, Boreal cool summer	OM2	C0	0	O horizon	40.0	27.6	0.8	4.5	1.6	33.7
LL010B	LL010	SBS_BC_	British Columbia	LL	Forest Soil	Whole Community DNA	PCR	Amplicon	pyrotag library	454 GS FLX Titanium	European Nucleotide Archive	https://www.ebi.ac.uk/ena/data/search?query=PRJEB12501	ERS1040358	SAMEA3733209	ERX1298695	ERR1226483	16S rRNA	V1-V3	NA	AGAGTTTGATCMTGGCTCAG	GWATTACCGCGGCKGCTG	2008-07-09	54.35	-122.61	Canada	0.3	780	3.3	146-193	Orthic Humo-Ferric Podzol, Gleyed Eluviated Dystric Brunisol	Subalpine fir, Douglas fir, Interior Spruce	Dfc, Boreal cool summer	OM2	C0	0	A horizon	17.0	1.2	0.1	4.8	1.6	19.3
LL011B	LL011	SBS_BC_	British Columbia	LL	Forest Soil	Whole Community DNA	PCR	Amplicon	pyrotag library	454 GS FLX Titanium	European Nucleotide Archive	https://www.ebi.ac.uk/ena/data/search?query=PRJEB12501	ERS1040359	SAMEA3733210	ERX1298696	ERR1226484	16S rRNA	V1-V3	NA	AGAGTTTGATCMTGGCTCAG	GWATTACCGCGGCKGCTG	2008-07-09	54.35	-122.61	Canada	0.3	780	3.3	146-193	Orthic Humo-Ferric Podzol, Gleyed Eluviated Dystric Brunisol	Subalpine fir, Douglas fir, Interior Spruce	Dfc, Boreal cool summer	OM2	C0	0	A horizon	19.0	1.2	0.1	4.8	1.6	19.3
LL012B	LL012	SBS_BC_	British Columbia	LL	Forest Soil	Whole Community DNA	PCR	Amplicon	pyrotag library	454 GS FLX Titanium	European Nucleotide Archive	https://www.ebi.ac.uk/ena/data/search?query=PRJEB12501	ERS1040360	SAMEA3733211	ERX1298697	ERR1226485	16S rRNA	V1-V3	NA	AGAGTTTGATCMTGGCTCAG	GWATTACCGCGGCKGCTG	2008-07-09	54.35	-122.61	Canada	0.3	780	3.3	146-193	Orthic Humo-Ferric Podzol, Gleyed Eluviated Dystric Brunisol	Subalpine fir, Douglas fir, Interior Spruce	Dfc, Boreal cool summer	OM2	C0	0	A horizon	16.0	1.2	0.1	4.8	1.6	19.3
LL016B	LL016	SBS_BC_	British Columbia	LL	Forest Soil	Whole Community DNA	PCR	Amplicon	pyrotag library	454 GS FLX Titanium	European Nucleotide Archive	https://www.ebi.ac.uk/ena/data/search?query=PRJEB12501	ERS1040361	SAMEA3733212	ERX1298698	ERR1226486	16S rRNA	V1-V3	NA	AGAGTTTGATCMTGGCTCAG	GWATTACCGCGGCKGCTG	2008-07-09	54.35	-122.61	Canada	0.3	780	3.3	146-193	Orthic Humo-Ferric Podzol, Gleyed Eluviated Dystric Brunisol	Subalpine fir, Douglas fir, Interior Spruce	Dfc, Boreal cool summer	OM3	C0	0	A horizon	23.0	1.7	0.1	5.1	1.3	20.9
LL017B	LL017	SBS_BC_	British Columbia	LL	Forest Soil	Whole Community DNA	PCR	Amplicon	pyrotag library	454 GS FLX Titanium	European Nucleotide Archive	https://www.ebi.ac.uk/ena/data/search?query=PRJEB12501	ERS1040362	SAMEA3733213	ERX1298699	ERR1226487	16S rRNA	V1-V3	NA	AGAGTTTGATCMTGGCTCAG	GWATTACCGCGGCKGCTG	2008-07-09	54.35	-122.61	Canada	0.3	780	3.3	146-193	Orthic Humo-Ferric Podzol, Gleyed Eluviated Dystric Brunisol	Subalpine fir, Douglas fir, Interior Spruce	Dfc, Boreal cool summer	OM3	C0	0	A horizon	20.0	1.7	0.1	5.1	1.3	20.9
LL018B	LL018	SBS_BC_	British Columbia	LL	Forest Soil	Whole Community DNA	PCR	Amplicon	pyrotag library	454 GS FLX Titanium	European Nucleotide Archive	https://www.ebi.ac.uk/ena/data/search?query=PRJEB12501	ERS1040363	SAMEA3733214	ERX1298700	ERR1226488	16S rRNA	V1-V3	NA	AGAGTTTGATCMTGGCTCAG	GWATTACCGCGGCKGCTG	2008-07-09	54.35	-122.61	Canada	0.3	780	3.3	146-193	Orthic Humo-Ferric Podzol, Gleyed Eluviated Dystric Brunisol	Subalpine fir, Douglas fir, Interior Spruce	Dfc, Boreal cool summer	OM3	C0	0	A horizon	21.0	1.7	0.1	5.1	1.3	20.9
LL019B	LL019	SBS_BC_	British Columbia	LL	Forest Soil	Whole Community DNA	PCR	Amplicon	pyrotag library	454 GS FLX Titanium	European Nucleotide Archive	https://www.ebi.ac.uk/ena/data/search?query=PRJEB12501	ERS1040364	SAMEA3733215	ERX1298701	ERR1226489	16S rRNA	V1-V3	NA	AGAGTTTGATCMTGGCTCAG	GWATTACCGCGGCKGCTG	2008-07-09	54.35	-122.61	Canada	0.1	780	3.3	146-193	Orthic Humo-Ferric Podzol, Gleyed Eluviated Dystric Brunisol	Subalpine fir, Douglas fir, Interior Spruce	Dfc, Boreal cool summer	OM1	C1	0	O horizon	61.0	36.0	0.8	4.6	1.5	43.8
LL020B	LL020	SBS_BC_	British Columbia	LL	Forest Soil	Whole Community DNA	PCR	Amplicon	pyrotag library	454 GS FLX Titanium	European Nucleotide Archive	https://www.ebi.ac.uk/ena/data/search?query=PRJEB12501	ERS1040365	SAMEA3733216	ERX1298702	ERR1226490	16S rRNA	V1-V3	NA	AGAGTTTGATCMTGGCTCAG	GWATTACCGCGGCKGCTG	2008-07-09	54.35	-122.61	Canada	0.1	780	3.3	146-193	Orthic Humo-Ferric Podzol, Gleyed Eluviated Dystric Brunisol	Subalpine fir, Douglas fir, Interior Spruce	Dfc, Boreal cool summer	OM1	C1	0	O horizon	46.0	36.0	0.8	4.6	1.5	43.8
LL021B	LL021	SBS_BC_	British Columbia	LL	Forest Soil	Whole Community DNA	PCR	Amplicon	pyrotag library	454 GS FLX Titanium	European Nucleotide Archive	https://www.ebi.ac.uk/ena/data/search?query=PRJEB12501	ERS1040366	SAMEA3733217	ERX1298703	ERR1226491	16S rRNA	V1-V3	NA	AGAGTTTGATCMTGGCTCAG	GWATTACCGCGGCKGCTG	2008-07-09	54.35	-122.61	Canada	0.1	780	3.3	146-193	Orthic Humo-Ferric Podzol, Gleyed Eluviated Dystric Brunisol	Subalpine fir, Douglas fir, Interior Spruce	Dfc, Boreal cool summer	OM1	C1	0	O horizon	44.0	36.0	0.8	4.6	1.5	43.8
LL022B	LL022	SBS_BC_	British Columbia	LL	Forest Soil	Whole Community DNA	PCR	Amplicon	pyrotag library	454 GS FLX Titanium	European Nucleotide Archive	https://www.ebi.ac.uk/ena/data/search?query=PRJEB12501	ERS1040367	SAMEA3733218	ERX1298704	ERR1226492	16S rRNA	V1-V3	NA	AGAGTTTGATCMTGGCTCAG	GWATTACCGCGGCKGCTG	2008-07-09	54.35	-122.61	Canada	0.3	780	3.3	146-193	Orthic Humo-Ferric Podzol, Gleyed Eluviated Dystric Brunisol	Subalpine fir, Douglas fir, Interior Spruce	Dfc, Boreal cool summer	OM1	C1	0	A horizon	17.0	1.2	0.1	4.6	1.5	23.4
LL023B	LL023	SBS_BC_	British Columbia	LL	Forest Soil	Whole Community DNA	PCR	Amplicon	pyrotag library	454 GS FLX Titanium	European Nucleotide Archive	https://www.ebi.ac.uk/ena/data/search?query=PRJEB12501	ERS1040368	SAMEA3733219	ERX1298705	ERR1226493	16S rRNA	V1-V3	NA	AGAGTTTGATCMTGGCTCAG	GWATTACCGCGGCKGCTG	2008-07-09	54.35	-122.61	Canada	0.3	780	3.3	146-193	Orthic Humo-Ferric Podzol, Gleyed Eluviated Dystric Brunisol	Subalpine fir, Douglas fir, Interior Spruce	Dfc, Boreal cool summer	OM1	C1	0	A horizon	20.0	1.2	0.1	4.6	1.5	23.4
LL024B	LL024	SBS_BC_	British Columbia	LL	Forest Soil	Whole Community DNA	PCR	Amplicon	pyrotag library	454 GS FLX Titanium	European Nucleotide Archive	https://www.ebi.ac.uk/ena/data/search?query=PRJEB12501	ERS1040369	SAMEA3733220	ERX1298706	ERR1226494	16S rRNA	V1-V3	NA	AGAGTTTGATCMTGGCTCAG	GWATTACCGCGGCKGCTG	2008-07-09	54.35	-122.61	Canada	0.3	780	3.3	146-193	Orthic Humo-Ferric Podzol, Gleyed Eluviated Dystric Brunisol	Subalpine fir, Douglas fir, Interior Spruce	Dfc, Boreal cool summer	OM1	C1	0	A horizon	19.0	1.2	0.1	4.6	1.5	23.4
LL025B	LL025	SBS_BC_	British Columbia	LL	Forest Soil	Whole Community DNA	PCR	Amplicon	pyrotag library	454 GS FLX Titanium	European Nucleotide Archive	https://www.ebi.ac.uk/ena/data/search?query=PRJEB12501	ERS1040370	SAMEA3733221	ERX1298707	ERR1226495	16S rRNA	V1-V3	NA	AGAGTTTGATCMTGGCTCAG	GWATTACCGCGGCKGCTG	2008-07-09	54.35	-122.61	Canada	0.1	780	3.3	146-193	Orthic Humo-Ferric Podzol, Gleyed Eluviated Dystric Brunisol	Subalpine fir, Douglas fir, Interior Spruce	Dfc, Boreal cool summer	OM2	C1	0	O horizon	52.0	27.5	0.8	4.5	1.6	34.8
LL026B	LL026	SBS_BC_	British Columbia	LL	Forest Soil	Whole Community DNA	PCR	Amplicon	pyrotag library	454 GS FLX Titanium	European Nucleotide Archive	https://www.ebi.ac.uk/ena/data/search?query=PRJEB12501	ERS1040371	SAMEA3733222	ERX1298708	ERR1226496	16S rRNA	V1-V3	NA	AGAGTTTGATCMTGGCTCAG	GWATTACCGCGGCKGCTG	2008-07-09	54.35	-122.61	Canada	0.1	780	3.3	146-193	Orthic Humo-Ferric Podzol, Gleyed Eluviated Dystric Brunisol	Subalpine fir, Douglas fir, Interior Spruce	Dfc, Boreal cool summer	OM2	C1	0	O horizon	55.0	27.5	0.8	4.5	1.6	34.8
LL027B	LL027	SBS_BC_	British Columbia	LL	Forest Soil	Whole Community DNA	PCR	Amplicon	pyrotag library	454 GS FLX Titanium	European Nucleotide Archive	https://www.ebi.ac.uk/ena/data/search?query=PRJEB12501	ERS1040372	SAMEA3733223	ERX1298709	ERR1226497	16S rRNA	V1-V3	NA	AGAGTTTGATCMTGGCTCAG	GWATTACCGCGGCKGCTG	2008-07-09	54.35	-122.61	Canada	0.1	780	3.3	146-193	Orthic Humo-Ferric Podzol, Gleyed Eluviated Dystric Brunisol	Subalpine fir, Douglas fir, Interior Spruce	Dfc, Boreal cool summer	OM2	C1	0	O horizon	60.0	27.5	0.8	4.5	1.6	34.8
LL028B	LL028	SBS_BC_	British Columbia	LL	Forest Soil	Whole Community DNA	PCR	Amplicon	pyrotag library	454 GS FLX Titanium	European Nucleotide Archive	https://www.ebi.ac.uk/ena/data/search?query=PRJEB12501	ERS1040373	SAMEA3733224	ERX1298710	ERR1226498	16S rRNA	V1-V3	NA	AGAGTTTGATCMTGGCTCAG	GWATTACCGCGGCKGCTG	2008-07-09	54.35	-122.61	Canada	0.3	780	3.3	146-193	Orthic Humo-Ferric Podzol, Gleyed Eluviated Dystric Brunisol	Subalpine fir, Douglas fir, Interior Spruce	Dfc, Boreal cool summer	OM2	C1	0	A horizon	20.0	1.5	0.1	4.9	1.6	21.1
LL029B	LL029	SBS_BC_	British Columbia	LL	Forest Soil	Whole Community DNA	PCR	Amplicon	pyrotag library	454 GS FLX Titanium	European Nucleotide Archive	https://www.ebi.ac.uk/ena/data/search?query=PRJEB12501	ERS1040374	SAMEA3733225	ERX1298711	ERR1226499	16S rRNA	V1-V3	NA	AGAGTTTGATCMTGGCTCAG	GWATTACCGCGGCKGCTG	2008-07-09	54.35	-122.61	Canada	0.3	780	3.3	146-193	Orthic Humo-Ferric Podzol, Gleyed Eluviated Dystric Brunisol	Subalpine fir, Douglas fir, Interior Spruce	Dfc, Boreal cool summer	OM2	C1	0	A horizon	17.0	1.5	0.1	4.9	1.6	21.1
LL030B	LL030	SBS_BC_	British Columbia	LL	Forest Soil	Whole Community DNA	PCR	Amplicon	pyrotag library	454 GS FLX Titanium	European Nucleotide Archive	https://www.ebi.ac.uk/ena/data/search?query=PRJEB12501	ERS1040375	SAMEA3733226	ERX1298712	ERR1226500	16S rRNA	V1-V3	NA	AGAGTTTGATCMTGGCTCAG	GWATTACCGCGGCKGCTG	2008-07-09	54.35	-122.61	Canada	0.3	780	3.3	146-193	Orthic Humo-Ferric Podzol, Gleyed Eluviated Dystric Brunisol	Subalpine fir, Douglas fir, Interior Spruce	Dfc, Boreal cool summer	OM2	C1	0	A horizon	19.0	1.5	0.1	4.9	1.6	21.1
LL034B	LL034	SBS_BC_	British Columbia	LL	Forest Soil	Whole Community DNA	PCR	Amplicon	pyrotag library	454 GS FLX Titanium	European Nucleotide Archive	https://www.ebi.ac.uk/ena/data/search?query=PRJEB12501	ERS1040376	SAMEA3733227	ERX1298713	ERR1226501	16S rRNA	V1-V3	NA	AGAGTTTGATCMTGGCTCAG	GWATTACCGCGGCKGCTG	2008-07-09	54.35	-122.61	Canada	0.3	780	3.3	146-193	Orthic Humo-Ferric Podzol, Gleyed Eluviated Dystric Brunisol	Subalpine fir, Douglas fir, Interior Spruce	Dfc, Boreal cool summer	OM3	C1	0	A horizon	22.0	1.3	0.1	4.8	1.4	21.8
LL035B	LL035	SBS_BC_	British Columbia	LL	Forest Soil	Whole Community DNA	PCR	Amplicon	pyrotag library	454 GS FLX Titanium	European Nucleotide Archive	https://www.ebi.ac.uk/ena/data/search?query=PRJEB12501	ERS1040377	SAMEA3733228	ERX1298714	ERR1226502	16S rRNA	V1-V3	NA	AGAGTTTGATCMTGGCTCAG	GWATTACCGCGGCKGCTG	2008-07-09	54.35	-122.61	Canada	0.3	780	3.3	146-193	Orthic Humo-Ferric Podzol, Gleyed Eluviated Dystric Brunisol	Subalpine fir, Douglas fir, Interior Spruce	Dfc, Boreal cool summer	OM3	C1	0	A horizon	20.0	1.3	0.1	4.8	1.4	21.8
LL036B	LL036	SBS_BC_	British Columbia	LL	Forest Soil	Whole Community DNA	PCR	Amplicon	pyrotag library	454 GS FLX Titanium	European Nucleotide Archive	https://www.ebi.ac.uk/ena/data/search?query=PRJEB12501	ERS1040378	SAMEA3733229	ERX1298715	ERR1226503	16S rRNA	V1-V3	NA	AGAGTTTGATCMTGGCTCAG	GWATTACCGCGGCKGCTG	2008-07-09	54.35	-122.61	Canada	0.3	780	3.3	146-193	Orthic Humo-Ferric Podzol, Gleyed Eluviated Dystric Brunisol	Subalpine fir, Douglas fir, Interior Spruce	Dfc, Boreal cool summer	OM3	C1	0	A horizon	24.0	1.3	0.1	4.8	1.4	21.8
LL037B	LL037	SBS_BC_	British Columbia	LL	Forest Soil	Whole Community DNA	PCR	Amplicon	pyrotag library	454 GS FLX Titanium	European Nucleotide Archive	https://www.ebi.ac.uk/ena/data/search?query=PRJEB12501	ERS1040379	SAMEA3733230	ERX1298716	ERR1226504	16S rRNA	V1-V3	NA	AGAGTTTGATCMTGGCTCAG	GWATTACCGCGGCKGCTG	2008-07-09	54.35	-122.61	Canada	0.1	780	3.3	146-193	Orthic Humo-Ferric Podzol, Gleyed Eluviated Dystric Brunisol	Subalpine fir, Douglas fir, Interior Spruce	Dfc, Boreal cool summer	OM1	C2	0	O horizon	60.0	31.3	1.0	4.7	1.4	31.9
LL038B	LL038	SBS_BC_	British Columbia	LL	Forest Soil	Whole Community DNA	PCR	Amplicon	pyrotag library	454 GS FLX Titanium	European Nucleotide Archive	https://www.ebi.ac.uk/ena/data/search?query=PRJEB12501	ERS1040380	SAMEA3733231	ERX1298717	ERR1226505	16S rRNA	V1-V3	NA	AGAGTTTGATCMTGGCTCAG	GWATTACCGCGGCKGCTG	2008-07-09	54.35	-122.61	Canada	0.1	780	3.3	146-193	Orthic Humo-Ferric Podzol, Gleyed Eluviated Dystric Brunisol	Subalpine fir, Douglas fir, Interior Spruce	Dfc, Boreal cool summer	OM1	C2	0	O horizon	47.0	31.3	1.0	4.7	1.4	31.9
LL039B	LL039	SBS_BC_	British Columbia	LL	Forest Soil	Whole Community DNA	PCR	Amplicon	pyrotag library	454 GS FLX Titanium	European Nucleotide Archive	https://www.ebi.ac.uk/ena/data/search?query=PRJEB12501	ERS1040381	SAMEA3733232	ERX1298718	ERR1226506	16S rRNA	V1-V3	NA	AGAGTTTGATCMTGGCTCAG	GWATTACCGCGGCKGCTG	2008-07-09	54.35	-122.61	Canada	0.1	780	3.3	146-193	Orthic Humo-Ferric Podzol, Gleyed Eluviated Dystric Brunisol	Subalpine fir, Douglas fir, Interior Spruce	Dfc, Boreal cool summer	OM1	C2	0	O horizon	66.0	31.3	1.0	4.7	1.4	31.9
LL040B	LL040	SBS_BC_	British Columbia	LL	Forest Soil	Whole Community DNA	PCR	Amplicon	pyrotag library	454 GS FLX Titanium	European Nucleotide Archive	https://www.ebi.ac.uk/ena/data/search?query=PRJEB12501	ERS1040382	SAMEA3733233	ERX1298719	ERR1226507	16S rRNA	V1-V3	NA	AGAGTTTGATCMTGGCTCAG	GWATTACCGCGGCKGCTG	2008-07-09	54.35	-122.61	Canada	0.3	780	3.3	146-193	Orthic Humo-Ferric Podzol, Gleyed Eluviated Dystric Brunisol	Subalpine fir, Douglas fir, Interior Spruce	Dfc, Boreal cool summer	OM1	C2	0	A horizon	22.0	1.9	0.1	5.0	1.4	20.6
LL041B	LL041	SBS_BC_	British Columbia	LL	Forest Soil	Whole Community DNA	PCR	Amplicon	pyrotag library	454 GS FLX Titanium	European Nucleotide Archive	https://www.ebi.ac.uk/ena/data/search?query=PRJEB12501	ERS1040383	SAMEA3733234	ERX1298720	ERR1226508	16S rRNA	V1-V3	NA	AGAGTTTGATCMTGGCTCAG	GWATTACCGCGGCKGCTG	2008-07-09	54.35	-122.61	Canada	0.3	780	3.3	146-193	Orthic Humo-Ferric Podzol, Gleyed Eluviated Dystric Brunisol	Subalpine fir, Douglas fir, Interior Spruce	Dfc, Boreal cool summer	OM1	C2	0	A horizon	20.0	1.2	0.1	5.0	1.4	16.7
LL042B	LL042	SBS_BC_	British Columbia	LL	Forest Soil	Whole Community DNA	PCR	Amplicon	pyrotag library	454 GS FLX Titanium	European Nucleotide Archive	https://www.ebi.ac.uk/ena/data/search?query=PRJEB12501	ERS1040384	SAMEA3733235	ERX1298721	ERR1226509	16S rRNA	V1-V3	NA	AGAGTTTGATCMTGGCTCAG	GWATTACCGCGGCKGCTG	2008-07-09	54.35	-122.61	Canada	0.3	780	3.3	146-193	Orthic Humo-Ferric Podzol, Gleyed Eluviated Dystric Brunisol	Subalpine fir, Douglas fir, Interior Spruce	Dfc, Boreal cool summer	OM1	C2	0	A horizon	19.0	1.4	0.1	5.0	1.4	17.5
LL043B	LL043	SBS_BC_	British Columbia	LL	Forest Soil	Whole Community DNA	PCR	Amplicon	pyrotag library	454 GS FLX Titanium	European Nucleotide Archive	https://www.ebi.ac.uk/ena/data/search?query=PRJEB12501	ERS1040385	SAMEA3733236	ERX1298722	ERR1226510	16S rRNA	V1-V3	NA	AGAGTTTGATCMTGGCTCAG	GWATTACCGCGGCKGCTG	2008-07-09	54.35	-122.61	Canada	0.1	780	3.3	146-193	Orthic Humo-Ferric Podzol, Gleyed Eluviated Dystric Brunisol	Subalpine fir, Douglas fir, Interior Spruce	Dfc, Boreal cool summer	OM2	C2	0	O horizon	42.0	30.2	0.8	4.4	1.5	36.8
LL044B	LL044	SBS_BC_	British Columbia	LL	Forest Soil	Whole Community DNA	PCR	Amplicon	pyrotag library	454 GS FLX Titanium	European Nucleotide Archive	https://www.ebi.ac.uk/ena/data/search?query=PRJEB12501	ERS1040386	SAMEA3733237	ERX1298723	ERR1226511	16S rRNA	V1-V3	NA	AGAGTTTGATCMTGGCTCAG	GWATTACCGCGGCKGCTG	2008-07-09	54.35	-122.61	Canada	0.1	780	3.3	146-193	Orthic Humo-Ferric Podzol, Gleyed Eluviated Dystric Brunisol	Subalpine fir, Douglas fir, Interior Spruce	Dfc, Boreal cool summer	OM2	C2	0	O horizon	59.0	30.2	0.8	4.4	1.5	36.8
LL045B	LL045	SBS_BC_	British Columbia	LL	Forest Soil	Whole Community DNA	PCR	Amplicon	pyrotag library	454 GS FLX Titanium	European Nucleotide Archive	https://www.ebi.ac.uk/ena/data/search?query=PRJEB12501	ERS1040387	SAMEA3733238	ERX1298724	ERR1226512	16S rRNA	V1-V3	NA	AGAGTTTGATCMTGGCTCAG	GWATTACCGCGGCKGCTG	2008-07-09	54.35	-122.61	Canada	0.1	780	3.3	146-193	Orthic Humo-Ferric Podzol, Gleyed Eluviated Dystric Brunisol	Subalpine fir, Douglas fir, Interior Spruce	Dfc, Boreal cool summer	OM2	C2	0	O horizon	46.0	30.2	0.8	4.4	1.5	36.8
LL046B	LL046	SBS_BC_	British Columbia	LL	Forest Soil	Whole Community DNA	PCR	Amplicon	pyrotag library	454 GS FLX Titanium	European Nucleotide Archive	https://www.ebi.ac.uk/ena/data/search?query=PRJEB12501	ERS1040388	SAMEA3733239	ERX1298725	ERR1226513	16S rRNA	V1-V3	NA	AGAGTTTGATCMTGGCTCAG	GWATTACCGCGGCKGCTG	2008-07-09	54.35	-122.61	Canada	0.3	780	3.3	146-193	Orthic Humo-Ferric Podzol, Gleyed Eluviated Dystric Brunisol	Subalpine fir, Douglas fir, Interior Spruce	Dfc, Boreal cool summer	OM2	C2	0	A horizon	22.0	1.6	0.1	5.0	1.5	21.0
LL047B	LL047	SBS_BC_	British Columbia	LL	Forest Soil	Whole Community DNA	PCR	Amplicon	pyrotag library	454 GS FLX Titanium	European Nucleotide Archive	https://www.ebi.ac.uk/ena/data/search?query=PRJEB12501	ERS1040389	SAMEA3733240	ERX1298726	ERR1226514	16S rRNA	V1-V3	NA	AGAGTTTGATCMTGGCTCAG	GWATTACCGCGGCKGCTG	2008-07-09	54.35	-122.61	Canada	0.3	780	3.3	146-193	Orthic Humo-Ferric Podzol, Gleyed Eluviated Dystric Brunisol	Subalpine fir, Douglas fir, Interior Spruce	Dfc, Boreal cool summer	OM2	C2	0	A horizon	21.0	1.6	0.1	5.0	1.5	21.0
LL048B	LL048	SBS_BC_	British Columbia	LL	Forest Soil	Whole Community DNA	PCR	Amplicon	pyrotag library	454 GS FLX Titanium	European Nucleotide Archive	https://www.ebi.ac.uk/ena/data/search?query=PRJEB12501	ERS1040390	SAMEA3733241	ERX1298727	ERR1226515	16S rRNA	V1-V3	NA	AGAGTTTGATCMTGGCTCAG	GWATTACCGCGGCKGCTG	2008-07-09	54.35	-122.61	Canada	0.3	780	3.3	146-193	Orthic Humo-Ferric Podzol, Gleyed Eluviated Dystric Brunisol	Subalpine fir, Douglas fir, Interior Spruce	Dfc, Boreal cool summer	OM2	C2	0	A horizon	23.0	1.6	0.1	5.0	1.5	21.0
LL052B	LL052	SBS_BC_	British Columbia	LL	Forest Soil	Whole Community DNA	PCR	Amplicon	pyrotag library	454 GS FLX Titanium	European Nucleotide Archive	https://www.ebi.ac.uk/ena/data/search?query=PRJEB12501	ERS1040391	SAMEA3733242	ERX1298728	ERR1226516	16S rRNA	V1-V3	NA	AGAGTTTGATCMTGGCTCAG	GWATTACCGCGGCKGCTG	2008-07-09	54.35	-122.61	Canada	0.3	780	3.3	146-193	Orthic Humo-Ferric Podzol, Gleyed Eluviated Dystric Brunisol	Subalpine fir, Douglas fir, Interior Spruce	Dfc, Boreal cool summer	OM3	C2	0	A horizon	18.0	1.4	0.1	5.1	1.5	22.8
LL053B	LL053	SBS_BC_	British Columbia	LL	Forest Soil	Whole Community DNA	PCR	Amplicon	pyrotag library	454 GS FLX Titanium	European Nucleotide Archive	https://www.ebi.ac.uk/ena/data/search?query=PRJEB12501	ERS1040392	SAMEA3733243	ERX1298729	ERR1226517	16S rRNA	V1-V3	NA	AGAGTTTGATCMTGGCTCAG	GWATTACCGCGGCKGCTG	2008-07-09	54.35	-122.61	Canada	0.3	780	3.3	146-193	Orthic Humo-Ferric Podzol, Gleyed Eluviated Dystric Brunisol	Subalpine fir, Douglas fir, Interior Spruce	Dfc, Boreal cool summer	OM3	C2	0	A horizon	21.0	1.4	0.1	5.1	1.5	22.8
LL054B	LL054	SBS_BC_	British Columbia	LL	Forest Soil	Whole Community DNA	PCR	Amplicon	pyrotag library	454 GS FLX Titanium	European Nucleotide Archive	https://www.ebi.ac.uk/ena/data/search?query=PRJEB12501	ERS1040393	SAMEA3733244	ERX1298730	ERR1226518	16S rRNA	V1-V3	NA	AGAGTTTGATCMTGGCTCAG	GWATTACCGCGGCKGCTG	2008-07-09	54.35	-122.61	Canada	0.3	780	3.3	146-193	Orthic Humo-Ferric Podzol, Gleyed Eluviated Dystric Brunisol	Subalpine fir, Douglas fir, Interior Spruce	Dfc, Boreal cool summer	OM3	C2	0	A horizon	21.0	1.4	0.1	5.1	1.5	22.8
LL055B	LL055	SBS_BC_	British Columbia	LL	Forest Soil	Whole Community DNA	PCR	Amplicon	pyrotag library	454 GS FLX Titanium	European Nucleotide Archive	https://www.ebi.ac.uk/ena/data/search?query=PRJEB12501	ERS1040394	SAMEA3733245	ERX1298731	ERR1226519	16S rRNA	V1-V3	NA	AGAGTTTGATCMTGGCTCAG	GWATTACCGCGGCKGCTG	2008-07-09	54.35	-122.61	Canada	0.1	780	3.3	146-193	Orthic Humo-Ferric Podzol, Gleyed Eluviated Dystric Brunisol	Subalpine fir, Douglas fir, Interior Spruce	Dfc, Boreal cool summer	REF	REF	0	O horizon	56.0	37.8	1.0	4.4	NA	36.4
LL056B	LL056	SBS_BC_	British Columbia	LL	Forest Soil	Whole Community DNA	PCR	Amplicon	pyrotag library	454 GS FLX Titanium	European Nucleotide Archive	https://www.ebi.ac.uk/ena/data/search?query=PRJEB12501	ERS1040395	SAMEA3733246	ERX1298732	ERR1226520	16S rRNA	V1-V3	NA	AGAGTTTGATCMTGGCTCAG	GWATTACCGCGGCKGCTG	2008-07-09	54.35	-122.61	Canada	0.1	780	3.3	146-193	Orthic Humo-Ferric Podzol, Gleyed Eluviated Dystric Brunisol	Subalpine fir, Douglas fir, Interior Spruce	Dfc, Boreal cool summer	REF	REF	0	O horizon	59.0	37.8	1.0	4.4	NA	36.4
LL057B	LL057	SBS_BC_	British Columbia	LL	Forest Soil	Whole Community DNA	PCR	Amplicon	pyrotag library	454 GS FLX Titanium	European Nucleotide Archive	https://www.ebi.ac.uk/ena/data/search?query=PRJEB12501	ERS1040396	SAMEA3733247	ERX1298733	ERR1226521	16S rRNA	V1-V3	NA	AGAGTTTGATCMTGGCTCAG	GWATTACCGCGGCKGCTG	2008-07-09	54.35	-122.61	Canada	0.1	780	3.3	146-193	Orthic Humo-Ferric Podzol, Gleyed Eluviated Dystric Brunisol	Subalpine fir, Douglas fir, Interior Spruce	Dfc, Boreal cool summer	REF	REF	0	O horizon	56.0	37.8	1.0	4.4	NA	36.4
LL058B	LL058	SBS_BC_	British Columbia	LL	Forest Soil	Whole Community DNA	PCR	Amplicon	pyrotag library	454 GS FLX Titanium	European Nucleotide Archive	https://www.ebi.ac.uk/ena/data/search?query=PRJEB12501	ERS1040397	SAMEA3733248	ERX1298734	ERR1226522	16S rRNA	V1-V3	NA	AGAGTTTGATCMTGGCTCAG	GWATTACCGCGGCKGCTG	2008-07-09	54.35	-122.61	Canada	0.3	780	3.3	146-193	Orthic Humo-Ferric Podzol, Gleyed Eluviated Dystric Brunisol	Subalpine fir, Douglas fir, Interior Spruce	Dfc, Boreal cool summer	REF	REF	0	A horizon	19.0	1.6	0.1	4.9	NA	22.3
LL059B	LL059	SBS_BC_	British Columbia	LL	Forest Soil	Whole Community DNA	PCR	Amplicon	pyrotag library	454 GS FLX Titanium	European Nucleotide Archive	https://www.ebi.ac.uk/ena/data/search?query=PRJEB12501	ERS1040398	SAMEA3733249	ERX1298735	ERR1226523	16S rRNA	V1-V3	NA	AGAGTTTGATCMTGGCTCAG	GWATTACCGCGGCKGCTG	2008-07-09	54.35	-122.61	Canada	0.3	780	3.3	146-193	Orthic Humo-Ferric Podzol, Gleyed Eluviated Dystric Brunisol	Subalpine fir, Douglas fir, Interior Spruce	Dfc, Boreal cool summer	REF	REF	0	A horizon	20.0	1.6	0.1	4.9	NA	22.3
LL060B	LL060	SBS_BC_	British Columbia	LL	Forest Soil	Whole Community DNA	PCR	Amplicon	pyrotag library	454 GS FLX Titanium	European Nucleotide Archive	https://www.ebi.ac.uk/ena/data/search?query=PRJEB12501	ERS1040399	SAMEA3733250	ERX1298736	ERR1226524	16S rRNA	V1-V3	NA	AGAGTTTGATCMTGGCTCAG	GWATTACCGCGGCKGCTG	2008-07-09	54.35	-122.61	Canada	0.3	780	3.3	146-193	Orthic Humo-Ferric Podzol, Gleyed Eluviated Dystric Brunisol	Subalpine fir, Douglas fir, Interior Spruce	Dfc, Boreal cool summer	REF	REF	0	A horizon	22.0	1.6	0.1	4.9	NA	22.3
OC304B	OL304	IDF_BC_	British Columbia	OC	Forest Soil	Whole Community DNA	PCR	Amplicon	pyrotag library	454 GS FLX Titanium	European Nucleotide Archive	https://www.ebi.ac.uk/ena/data/search?query=PRJEB12501	ERS1040400	SAMEA3733251	ERX1298737	ERR1226525	16S rRNA	V1-V3	NA	AGAGTTTGATCMTGGCTCAG	GWATTACCGCGGCKGCTG	2010-06-26	50.88	-120.35	Canada	0.3	1075	2.5	300	Brunisolic Gray Luvisol	Douglas fir	Dfb, Humid Continental warm summer	OM3	C0	0	A horizon	23.5	NA	NA	0.0	NA	NA
OC305B	OL305	IDF_BC_	British Columbia	OC	Forest Soil	Whole Community DNA	PCR	Amplicon	pyrotag library	454 GS FLX Titanium	European Nucleotide Archive	https://www.ebi.ac.uk/ena/data/search?query=PRJEB12501	ERS1040401	SAMEA3733252	ERX1298738	ERR1226526	16S rRNA	V1-V3	NA	AGAGTTTGATCMTGGCTCAG	GWATTACCGCGGCKGCTG	2010-06-26	50.88	-120.35	Canada	0.3	1075	2.5	300	Brunisolic Gray Luvisol	Douglas fir	Dfb, Humid Continental warm summer	OM3	C0	0	A horizon	17.3	NA	NA	0.0	NA	NA
OC306B	OL306	IDF_BC_	British Columbia	OC	Forest Soil	Whole Community DNA	PCR	Amplicon	pyrotag library	454 GS FLX Titanium	European Nucleotide Archive	https://www.ebi.ac.uk/ena/data/search?query=PRJEB12501	ERS1040402	SAMEA3733253	ERX1298739	ERR1226527	16S rRNA	V1-V3	NA	AGAGTTTGATCMTGGCTCAG	GWATTACCGCGGCKGCTG	2010-06-26	50.88	-120.35	Canada	0.3	1075	2.5	300	Brunisolic Gray Luvisol	Douglas fir	Dfb, Humid Continental warm summer	OM3	C0	0	A horizon	22.0	NA	NA	0.0	NA	NA
OC307B	OL307	IDF_BC_	British Columbia	OC	Forest Soil	Whole Community DNA	PCR	Amplicon	pyrotag library	454 GS FLX Titanium	European Nucleotide Archive	https://www.ebi.ac.uk/ena/data/search?query=PRJEB12501	ERS1040403	SAMEA3733254	ERX1298740	ERR1226528	16S rRNA	V1-V3	NA	AGAGTTTGATCMTGGCTCAG	GWATTACCGCGGCKGCTG	2010-06-26	50.88	-120.35	Canada	0.1	1075	2.5	300	Brunisolic Gray Luvisol	Douglas fir	Dfb, Humid Continental warm summer	OM1	C0	0	O horizon	59.5	NA	NA	0.0	NA	NA
OC308B	OL308	IDF_BC_	British Columbia	OC	Forest Soil	Whole Community DNA	PCR	Amplicon	pyrotag library	454 GS FLX Titanium	European Nucleotide Archive	https://www.ebi.ac.uk/ena/data/search?query=PRJEB12501	ERS1040404	SAMEA3733255	ERX1298741	ERR1226529	16S rRNA	V1-V3	NA	AGAGTTTGATCMTGGCTCAG	GWATTACCGCGGCKGCTG	2010-06-26	50.88	-120.35	Canada	0.1	1075	2.5	300	Brunisolic Gray Luvisol	Douglas fir	Dfb, Humid Continental warm summer	OM1	C0	0	O horizon	64.7	NA	NA	0.0	NA	NA
OC309B	OL309	IDF_BC_	British Columbia	OC	Forest Soil	Whole Community DNA	PCR	Amplicon	pyrotag library	454 GS FLX Titanium	European Nucleotide Archive	https://www.ebi.ac.uk/ena/data/search?query=PRJEB12501	ERS1040405	SAMEA3733256	ERX1298742	ERR1226530	16S rRNA	V1-V3	NA	AGAGTTTGATCMTGGCTCAG	GWATTACCGCGGCKGCTG	2010-06-26	50.88	-120.35	Canada	0.1	1075	2.5	300	Brunisolic Gray Luvisol	Douglas fir	Dfb, Humid Continental warm summer	OM1	C0	0	O horizon	61.8	NA	NA	0.0	NA	NA
OC310B	OL310	IDF_BC_	British Columbia	OC	Forest Soil	Whole Community DNA	PCR	Amplicon	pyrotag library	454 GS FLX Titanium	European Nucleotide Archive	https://www.ebi.ac.uk/ena/data/search?query=PRJEB12501	ERS1040406	SAMEA3733257	ERX1298743	ERR1226531	16S rRNA	V1-V3	NA	AGAGTTTGATCMTGGCTCAG	GWATTACCGCGGCKGCTG	2010-06-26	50.88	-120.35	Canada	0.3	1075	2.5	300	Brunisolic Gray Luvisol	Douglas fir	Dfb, Humid Continental warm summer	OM1	C0	0	A horizon	26.0	NA	NA	0.0	NA	NA
OC311B	OL311	IDF_BC_	British Columbia	OC	Forest Soil	Whole Community DNA	PCR	Amplicon	pyrotag library	454 GS FLX Titanium	European Nucleotide Archive	https://www.ebi.ac.uk/ena/data/search?query=PRJEB12501	ERS1040407	SAMEA3733258	ERX1298744	ERR1226532	16S rRNA	V1-V3	NA	AGAGTTTGATCMTGGCTCAG	GWATTACCGCGGCKGCTG	2010-06-26	50.88	-120.35	Canada	0.3	1075	2.5	300	Brunisolic Gray Luvisol	Douglas fir	Dfb, Humid Continental warm summer	OM1	C0	0	A horizon	26.6	NA	NA	0.0	NA	NA
OC312B	OL312	IDF_BC_	British Columbia	OC	Forest Soil	Whole Community DNA	PCR	Amplicon	pyrotag library	454 GS FLX Titanium	European Nucleotide Archive	https://www.ebi.ac.uk/ena/data/search?query=PRJEB12501	ERS1040408	SAMEA3733259	ERX1298745	ERR1226533	16S rRNA	V1-V3	NA	AGAGTTTGATCMTGGCTCAG	GWATTACCGCGGCKGCTG	2010-06-26	50.88	-120.35	Canada	0.3	1075	2.5	300	Brunisolic Gray Luvisol	Douglas fir	Dfb, Humid Continental warm summer	OM1	C0	0	A horizon	28.2	NA	NA	0.0	NA	NA
OC313B	OL313	IDF_BC_	British Columbia	OC	Forest Soil	Whole Community DNA	PCR	Amplicon	pyrotag library	454 GS FLX Titanium	European Nucleotide Archive	https://www.ebi.ac.uk/ena/data/search?query=PRJEB12501	ERS1040409	SAMEA3733260	ERX1298746	ERR1226534	16S rRNA	V1-V3	NA	AGAGTTTGATCMTGGCTCAG	GWATTACCGCGGCKGCTG	2010-06-26	50.88	-120.35	Canada	0.1	1075	2.5	300	Brunisolic Gray Luvisol	Douglas fir	Dfb, Humid Continental warm summer	OM2	C1	0	O horizon	58.0	NA	NA	0.0	NA	NA
OC314B	OL314	IDF_BC_	British Columbia	OC	Forest Soil	Whole Community DNA	PCR	Amplicon	pyrotag library	454 GS FLX Titanium	European Nucleotide Archive	https://www.ebi.ac.uk/ena/data/search?query=PRJEB12501	ERS1040410	SAMEA3733261	ERX1298747	ERR1226535	16S rRNA	V1-V3	NA	AGAGTTTGATCMTGGCTCAG	GWATTACCGCGGCKGCTG	2010-06-26	50.88	-120.35	Canada	0.1	1075	2.5	300	Brunisolic Gray Luvisol	Douglas fir	Dfb, Humid Continental warm summer	OM2	C1	0	O horizon	63.5	NA	NA	0.0	NA	NA
OC315B	OL315	IDF_BC_	British Columbia	OC	Forest Soil	Whole Community DNA	PCR	Amplicon	pyrotag library	454 GS FLX Titanium	European Nucleotide Archive	https://www.ebi.ac.uk/ena/data/search?query=PRJEB12501	ERS1040411	SAMEA3733262	ERX1298748	ERR1226536	16S rRNA	V1-V3	NA	AGAGTTTGATCMTGGCTCAG	GWATTACCGCGGCKGCTG	2010-06-26	50.88	-120.35	Canada	0.1	1075	2.5	300	Brunisolic Gray Luvisol	Douglas fir	Dfb, Humid Continental warm summer	OM2	C1	0	O horizon	57.2	NA	NA	0.0	NA	NA
OC316B	OL316	IDF_BC_	British Columbia	OC	Forest Soil	Whole Community DNA	PCR	Amplicon	pyrotag library	454 GS FLX Titanium	European Nucleotide Archive	https://www.ebi.ac.uk/ena/data/search?query=PRJEB12501	ERS1040412	SAMEA3733263	ERX1298749	ERR1226537	16S rRNA	V1-V3	NA	AGAGTTTGATCMTGGCTCAG	GWATTACCGCGGCKGCTG	2010-06-26	50.88	-120.35	Canada	0.3	1075	2.5	300	Brunisolic Gray Luvisol	Douglas fir	Dfb, Humid Continental warm summer	OM2	C1	0	A horizon	24.3	NA	NA	0.0	NA	NA
OC317B	OL317	IDF_BC_	British Columbia	OC	Forest Soil	Whole Community DNA	PCR	Amplicon	pyrotag library	454 GS FLX Titanium	European Nucleotide Archive	https://www.ebi.ac.uk/ena/data/search?query=PRJEB12501	ERS1040413	SAMEA3733264	ERX1298750	ERR1226538	16S rRNA	V1-V3	NA	AGAGTTTGATCMTGGCTCAG	GWATTACCGCGGCKGCTG	2010-06-26	50.88	-120.35	Canada	0.3	1075	2.5	300	Brunisolic Gray Luvisol	Douglas fir	Dfb, Humid Continental warm summer	OM2	C1	0	A horizon	28.6	NA	NA	0.0	NA	NA
OC318B	OL318	IDF_BC_	British Columbia	OC	Forest Soil	Whole Community DNA	PCR	Amplicon	pyrotag library	454 GS FLX Titanium	European Nucleotide Archive	https://www.ebi.ac.uk/ena/data/search?query=PRJEB12501	ERS1040414	SAMEA3733265	ERX1298751	ERR1226539	16S rRNA	V1-V3	NA	AGAGTTTGATCMTGGCTCAG	GWATTACCGCGGCKGCTG	2010-06-26	50.88	-120.35	Canada	0.3	1075	2.5	300	Brunisolic Gray Luvisol	Douglas fir	Dfb, Humid Continental warm summer	OM2	C1	0	A horizon	21.1	NA	NA	0.0	NA	NA
OC319B	OL319	IDF_BC_	British Columbia	OC	Forest Soil	Whole Community DNA	PCR	Amplicon	pyrotag library	454 GS FLX Titanium	European Nucleotide Archive	https://www.ebi.ac.uk/ena/data/search?query=PRJEB12501	ERS1040415	SAMEA3733266	ERX1298752	ERR1226540	16S rRNA	V1-V3	NA	AGAGTTTGATCMTGGCTCAG	GWATTACCGCGGCKGCTG	2010-06-26	50.88	-120.35	Canada	0.1	1075	2.5	300	Brunisolic Gray Luvisol	Douglas fir	Dfb, Humid Continental warm summer	OM2	C2	0	O horizon	61.5	NA	NA	0.0	NA	NA
OC320B	OL320	IDF_BC_	British Columbia	OC	Forest Soil	Whole Community DNA	PCR	Amplicon	pyrotag library	454 GS FLX Titanium	European Nucleotide Archive	https://www.ebi.ac.uk/ena/data/search?query=PRJEB12501	ERS1040416	SAMEA3733267	ERX1298753	ERR1226541	16S rRNA	V1-V3	NA	AGAGTTTGATCMTGGCTCAG	GWATTACCGCGGCKGCTG	2010-06-26	50.88	-120.35	Canada	0.1	1075	2.5	300	Brunisolic Gray Luvisol	Douglas fir	Dfb, Humid Continental warm summer	OM2	C2	0	O horizon	62.7	NA	NA	0.0	NA	NA
OC321B	OL321	IDF_BC_	British Columbia	OC	Forest Soil	Whole Community DNA	PCR	Amplicon	pyrotag library	454 GS FLX Titanium	European Nucleotide Archive	https://www.ebi.ac.uk/ena/data/search?query=PRJEB12501	ERS1040417	SAMEA3733268	ERX1298754	ERR1226542	16S rRNA	V1-V3	NA	AGAGTTTGATCMTGGCTCAG	GWATTACCGCGGCKGCTG	2010-06-26	50.88	-120.35	Canada	0.1	1075	2.5	300	Brunisolic Gray Luvisol	Douglas fir	Dfb, Humid Continental warm summer	OM2	C2	0	O horizon	57.7	NA	NA	0.0	NA	NA
OC322B	OL322	IDF_BC_	British Columbia	OC	Forest Soil	Whole Community DNA	PCR	Amplicon	pyrotag library	454 GS FLX Titanium	European Nucleotide Archive	https://www.ebi.ac.uk/ena/data/search?query=PRJEB12501	ERS1040418	SAMEA3733269	ERX1298755	ERR1226543	16S rRNA	V1-V3	NA	AGAGTTTGATCMTGGCTCAG	GWATTACCGCGGCKGCTG	2010-06-26	50.88	-120.35	Canada	0.3	1075	2.5	300	Brunisolic Gray Luvisol	Douglas fir	Dfb, Humid Continental warm summer	OM2	C2	0	A horizon	27.7	NA	NA	0.0	NA	NA
OC323B	OL323	IDF_BC_	British Columbia	OC	Forest Soil	Whole Community DNA	PCR	Amplicon	pyrotag library	454 GS FLX Titanium	European Nucleotide Archive	https://www.ebi.ac.uk/ena/data/search?query=PRJEB12501	ERS1040419	SAMEA3733270	ERX1298756	ERR1226544	16S rRNA	V1-V3	NA	AGAGTTTGATCMTGGCTCAG	GWATTACCGCGGCKGCTG	2010-06-26	50.88	-120.35	Canada	0.3	1075	2.5	300	Brunisolic Gray Luvisol	Douglas fir	Dfb, Humid Continental warm summer	OM2	C2	0	A horizon	23.4	NA	NA	0.0	NA	NA
OC324B	OL324	IDF_BC_	British Columbia	OC	Forest Soil	Whole Community DNA	PCR	Amplicon	pyrotag library	454 GS FLX Titanium	European Nucleotide Archive	https://www.ebi.ac.uk/ena/data/search?query=PRJEB12501	ERS1040420	SAMEA3733271	ERX1298757	ERR1226545	16S rRNA	V1-V3	NA	AGAGTTTGATCMTGGCTCAG	GWATTACCGCGGCKGCTG	2010-06-26	50.88	-120.35	Canada	0.3	1075	2.5	300	Brunisolic Gray Luvisol	Douglas fir	Dfb, Humid Continental warm summer	OM2	C2	0	A horizon	23.7	NA	NA	0.0	NA	NA
OC325B	OL325	IDF_BC_	British Columbia	OC	Forest Soil	Whole Community DNA	PCR	Amplicon	pyrotag library	454 GS FLX Titanium	European Nucleotide Archive	https://www.ebi.ac.uk/ena/data/search?query=PRJEB12501	ERS1040421	SAMEA3733272	ERX1298758	ERR1226546	16S rRNA	V1-V3	NA	AGAGTTTGATCMTGGCTCAG	GWATTACCGCGGCKGCTG	2010-06-26	50.88	-120.35	Canada	0.1	1075	2.5	300	Brunisolic Gray Luvisol	Douglas fir	Dfb, Humid Continental warm summer	OM2	C0	0	O horizon	64.4	NA	NA	0.0	NA	NA
OC326B	OL326	IDF_BC_	British Columbia	OC	Forest Soil	Whole Community DNA	PCR	Amplicon	pyrotag library	454 GS FLX Titanium	European Nucleotide Archive	https://www.ebi.ac.uk/ena/data/search?query=PRJEB12501	ERS1040422	SAMEA3733273	ERX1298759	ERR1226547	16S rRNA	V1-V3	NA	AGAGTTTGATCMTGGCTCAG	GWATTACCGCGGCKGCTG	2010-06-26	50.88	-120.35	Canada	0.1	1075	2.5	300	Brunisolic Gray Luvisol	Douglas fir	Dfb, Humid Continental warm summer	OM2	C0	0	O horizon	62.2	NA	NA	0.0	NA	NA
OC327B	OL327	IDF_BC_	British Columbia	OC	Forest Soil	Whole Community DNA	PCR	Amplicon	pyrotag library	454 GS FLX Titanium	European Nucleotide Archive	https://www.ebi.ac.uk/ena/data/search?query=PRJEB12501	ERS1040423	SAMEA3733274	ERX1298760	ERR1226548	16S rRNA	V1-V3	NA	AGAGTTTGATCMTGGCTCAG	GWATTACCGCGGCKGCTG	2010-06-26	50.88	-120.35	Canada	0.1	1075	2.5	300	Brunisolic Gray Luvisol	Douglas fir	Dfb, Humid Continental warm summer	OM2	C0	0	O horizon	53.7	NA	NA	0.0	NA	NA
OC328B	OL328	IDF_BC_	British Columbia	OC	Forest Soil	Whole Community DNA	PCR	Amplicon	pyrotag library	454 GS FLX Titanium	European Nucleotide Archive	https://www.ebi.ac.uk/ena/data/search?query=PRJEB12501	ERS1040424	SAMEA3733275	ERX1298761	ERR1226549	16S rRNA	V1-V3	NA	AGAGTTTGATCMTGGCTCAG	GWATTACCGCGGCKGCTG	2010-06-26	50.88	-120.35	Canada	0.3	1075	2.5	300	Brunisolic Gray Luvisol	Douglas fir	Dfb, Humid Continental warm summer	OM2	C0	0	A horizon	26.1	NA	NA	0.0	NA	NA
OC329B	OL329	IDF_BC_	British Columbia	OC	Forest Soil	Whole Community DNA	PCR	Amplicon	pyrotag library	454 GS FLX Titanium	European Nucleotide Archive	https://www.ebi.ac.uk/ena/data/search?query=PRJEB12501	ERS1040425	SAMEA3733276	ERX1298762	ERR1226550	16S rRNA	V1-V3	NA	AGAGTTTGATCMTGGCTCAG	GWATTACCGCGGCKGCTG	2010-06-26	50.88	-120.35	Canada	0.3	1075	2.5	300	Brunisolic Gray Luvisol	Douglas fir	Dfb, Humid Continental warm summer	OM2	C0	0	A horizon	22.3	NA	NA	0.0	NA	NA
OC330B	OL330	IDF_BC_	British Columbia	OC	Forest Soil	Whole Community DNA	PCR	Amplicon	pyrotag library	454 GS FLX Titanium	European Nucleotide Archive	https://www.ebi.ac.uk/ena/data/search?query=PRJEB12501	ERS1040426	SAMEA3733277	ERX1298763	ERR1226551	16S rRNA	V1-V3	NA	AGAGTTTGATCMTGGCTCAG	GWATTACCGCGGCKGCTG	2010-06-26	50.88	-120.35	Canada	0.3	1075	2.5	300	Brunisolic Gray Luvisol	Douglas fir	Dfb, Humid Continental warm summer	OM2	C0	0	A horizon	23.9	NA	NA	0.0	NA	NA
OC331B	OL331	IDF_BC_	British Columbia	OC	Forest Soil	Whole Community DNA	PCR	Amplicon	pyrotag library	454 GS FLX Titanium	European Nucleotide Archive	https://www.ebi.ac.uk/ena/data/search?query=PRJEB12501	ERS1040427	SAMEA3733278	ERX1298764	ERR1226552	16S rRNA	V1-V3	NA	AGAGTTTGATCMTGGCTCAG	GWATTACCGCGGCKGCTG	2010-06-26	50.88	-120.35	Canada	0.1	1075	2.5	300	Brunisolic Gray Luvisol	Douglas fir	Dfb, Humid Continental warm summer	OM1	C2	0	O horizon	57.4	NA	NA	0.0	NA	NA
OC332B	OL332	IDF_BC_	British Columbia	OC	Forest Soil	Whole Community DNA	PCR	Amplicon	pyrotag library	454 GS FLX Titanium	European Nucleotide Archive	https://www.ebi.ac.uk/ena/data/search?query=PRJEB12501	ERS1040428	SAMEA3733279	ERX1298765	ERR1226553	16S rRNA	V1-V3	NA	AGAGTTTGATCMTGGCTCAG	GWATTACCGCGGCKGCTG	2010-06-26	50.88	-120.35	Canada	0.1	1075	2.5	300	Brunisolic Gray Luvisol	Douglas fir	Dfb, Humid Continental warm summer	OM1	C2	0	O horizon	50.5	NA	NA	0.0	NA	NA
OC333B	OL333	IDF_BC_	British Columbia	OC	Forest Soil	Whole Community DNA	PCR	Amplicon	pyrotag library	454 GS FLX Titanium	European Nucleotide Archive	https://www.ebi.ac.uk/ena/data/search?query=PRJEB12501	ERS1040429	SAMEA3733280	ERX1298766	ERR1226554	16S rRNA	V1-V3	NA	AGAGTTTGATCMTGGCTCAG	GWATTACCGCGGCKGCTG	2010-06-26	50.88	-120.35	Canada	0.1	1075	2.5	300	Brunisolic Gray Luvisol	Douglas fir	Dfb, Humid Continental warm summer	OM1	C2	0	O horizon	60.9	NA	NA	0.0	NA	NA
OC334B	OL334	IDF_BC_	British Columbia	OC	Forest Soil	Whole Community DNA	PCR	Amplicon	pyrotag library	454 GS FLX Titanium	European Nucleotide Archive	https://www.ebi.ac.uk/ena/data/search?query=PRJEB12501	ERS1040430	SAMEA3733281	ERX1298767	ERR1226555	16S rRNA	V1-V3	NA	AGAGTTTGATCMTGGCTCAG	GWATTACCGCGGCKGCTG	2010-06-26	50.88	-120.35	Canada	0.3	1075	2.5	300	Brunisolic Gray Luvisol	Douglas fir	Dfb, Humid Continental warm summer	OM1	C2	0	A horizon	24.0	NA	NA	0.0	NA	NA
OC335B	OL335	IDF_BC_	British Columbia	OC	Forest Soil	Whole Community DNA	PCR	Amplicon	pyrotag library	454 GS FLX Titanium	European Nucleotide Archive	https://www.ebi.ac.uk/ena/data/search?query=PRJEB12501	ERS1040431	SAMEA3733282	ERX1298768	ERR1226556	16S rRNA	V1-V3	NA	AGAGTTTGATCMTGGCTCAG	GWATTACCGCGGCKGCTG	2010-06-26	50.88	-120.35	Canada	0.3	1075	2.5	300	Brunisolic Gray Luvisol	Douglas fir	Dfb, Humid Continental warm summer	OM1	C2	0	A horizon	25.6	NA	NA	0.0	NA	NA
OC336B	OL336	IDF_BC_	British Columbia	OC	Forest Soil	Whole Community DNA	PCR	Amplicon	pyrotag library	454 GS FLX Titanium	European Nucleotide Archive	https://www.ebi.ac.uk/ena/data/search?query=PRJEB12501	ERS1040432	SAMEA3733283	ERX1298769	ERR1226557	16S rRNA	V1-V3	NA	AGAGTTTGATCMTGGCTCAG	GWATTACCGCGGCKGCTG	2010-06-26	50.88	-120.35	Canada	0.3	1075	2.5	300	Brunisolic Gray Luvisol	Douglas fir	Dfb, Humid Continental warm summer	OM1	C2	0	A horizon	25.4	NA	NA	0.0	NA	NA
OC340B	OL340	IDF_BC_	British Columbia	OC	Forest Soil	Whole Community DNA	PCR	Amplicon	pyrotag library	454 GS FLX Titanium	European Nucleotide Archive	https://www.ebi.ac.uk/ena/data/search?query=PRJEB12501	ERS1040433	SAMEA3733284	ERX1298770	ERR1226558	16S rRNA	V1-V3	NA	AGAGTTTGATCMTGGCTCAG	GWATTACCGCGGCKGCTG	2010-06-26	50.88	-120.35	Canada	0.3	1075	2.5	300	Brunisolic Gray Luvisol	Douglas fir	Dfb, Humid Continental warm summer	OM3	C2	0	A horizon	22.9	NA	NA	0.0	NA	NA
OC341B	OL341	IDF_BC_	British Columbia	OC	Forest Soil	Whole Community DNA	PCR	Amplicon	pyrotag library	454 GS FLX Titanium	European Nucleotide Archive	https://www.ebi.ac.uk/ena/data/search?query=PRJEB12501	ERS1040434	SAMEA3733285	ERX1298771	ERR1226559	16S rRNA	V1-V3	NA	AGAGTTTGATCMTGGCTCAG	GWATTACCGCGGCKGCTG	2010-06-26	50.88	-120.35	Canada	0.3	1075	2.5	300	Brunisolic Gray Luvisol	Douglas fir	Dfb, Humid Continental warm summer	OM3	C2	0	A horizon	22.1	NA	NA	0.0	NA	NA
OC342B	OL342	IDF_BC_	British Columbia	OC	Forest Soil	Whole Community DNA	PCR	Amplicon	pyrotag library	454 GS FLX Titanium	European Nucleotide Archive	https://www.ebi.ac.uk/ena/data/search?query=PRJEB12501	ERS1040435	SAMEA3733286	ERX1298772	ERR1226560	16S rRNA	V1-V3	NA	AGAGTTTGATCMTGGCTCAG	GWATTACCGCGGCKGCTG	2010-06-26	50.88	-120.35	Canada	0.3	1075	2.5	300	Brunisolic Gray Luvisol	Douglas fir	Dfb, Humid Continental warm summer	OM3	C2	0	A horizon	20.3	NA	NA	0.0	NA	NA
OC343B	OL343	IDF_BC_	British Columbia	OC	Forest Soil	Whole Community DNA	PCR	Amplicon	pyrotag library	454 GS FLX Titanium	European Nucleotide Archive	https://www.ebi.ac.uk/ena/data/search?query=PRJEB12501	ERS1040436	SAMEA3733287	ERX1298773	ERR1226561	16S rRNA	V1-V3	NA	AGAGTTTGATCMTGGCTCAG	GWATTACCGCGGCKGCTG	2010-06-26	50.88	-120.35	Canada	0.1	1075	2.5	300	Brunisolic Gray Luvisol	Douglas fir	Dfb, Humid Continental warm summer	OM1	C1	0	O horizon	54.7	NA	NA	0.0	NA	NA
OC344B	OL344	IDF_BC_	British Columbia	OC	Forest Soil	Whole Community DNA	PCR	Amplicon	pyrotag library	454 GS FLX Titanium	European Nucleotide Archive	https://www.ebi.ac.uk/ena/data/search?query=PRJEB12501	ERS1040437	SAMEA3733288	ERX1298774	ERR1226562	16S rRNA	V1-V3	NA	AGAGTTTGATCMTGGCTCAG	GWATTACCGCGGCKGCTG	2010-06-26	50.88	-120.35	Canada	0.1	1075	2.5	300	Brunisolic Gray Luvisol	Douglas fir	Dfb, Humid Continental warm summer	OM1	C1	0	O horizon	58.7	NA	NA	0.0	NA	NA
OC345B	OL345	IDF_BC_	British Columbia	OC	Forest Soil	Whole Community DNA	PCR	Amplicon	pyrotag library	454 GS FLX Titanium	European Nucleotide Archive	https://www.ebi.ac.uk/ena/data/search?query=PRJEB12501	ERS1040438	SAMEA3733289	ERX1298775	ERR1226563	16S rRNA	V1-V3	NA	AGAGTTTGATCMTGGCTCAG	GWATTACCGCGGCKGCTG	2010-06-26	50.88	-120.35	Canada	0.1	1075	2.5	300	Brunisolic Gray Luvisol	Douglas fir	Dfb, Humid Continental warm summer	OM1	C1	0	O horizon	57.4	NA	NA	0.0	NA	NA
OC346B	OL346	IDF_BC_	British Columbia	OC	Forest Soil	Whole Community DNA	PCR	Amplicon	pyrotag library	454 GS FLX Titanium	European Nucleotide Archive	https://www.ebi.ac.uk/ena/data/search?query=PRJEB12501	ERS1040439	SAMEA3733290	ERX1298776	ERR1226564	16S rRNA	V1-V3	NA	AGAGTTTGATCMTGGCTCAG	GWATTACCGCGGCKGCTG	2010-06-26	50.88	-120.35	Canada	0.3	1075	2.5	300	Brunisolic Gray Luvisol	Douglas fir	Dfb, Humid Continental warm summer	OM1	C1	0	A horizon	26.2	NA	NA	0.0	NA	NA
OC347B	OL347	IDF_BC_	British Columbia	OC	Forest Soil	Whole Community DNA	PCR	Amplicon	pyrotag library	454 GS FLX Titanium	European Nucleotide Archive	https://www.ebi.ac.uk/ena/data/search?query=PRJEB12501	ERS1040440	SAMEA3733291	ERX1298777	ERR1226565	16S rRNA	V1-V3	NA	AGAGTTTGATCMTGGCTCAG	GWATTACCGCGGCKGCTG	2010-06-26	50.88	-120.35	Canada	0.3	1075	2.5	300	Brunisolic Gray Luvisol	Douglas fir	Dfb, Humid Continental warm summer	OM1	C1	0	A horizon	28.0	NA	NA	0.0	NA	NA
OC348B	OL348	IDF_BC_	British Columbia	OC	Forest Soil	Whole Community DNA	PCR	Amplicon	pyrotag library	454 GS FLX Titanium	European Nucleotide Archive	https://www.ebi.ac.uk/ena/data/search?query=PRJEB12501	ERS1040441	SAMEA3733292	ERX1298778	ERR1226566	16S rRNA	V1-V3	NA	AGAGTTTGATCMTGGCTCAG	GWATTACCGCGGCKGCTG	2010-06-26	50.88	-120.35	Canada	0.3	1075	2.5	300	Brunisolic Gray Luvisol	Douglas fir	Dfb, Humid Continental warm summer	OM1	C1	0	A horizon	26.5	NA	NA	0.0	NA	NA
OC352B	OL352	IDF_BC_	British Columbia	OC	Forest Soil	Whole Community DNA	PCR	Amplicon	pyrotag library	454 GS FLX Titanium	European Nucleotide Archive	https://www.ebi.ac.uk/ena/data/search?query=PRJEB12501	ERS1040442	SAMEA3733293	ERX1298779	ERR1226567	16S rRNA	V1-V3	NA	AGAGTTTGATCMTGGCTCAG	GWATTACCGCGGCKGCTG	2010-06-26	50.88	-120.35	Canada	0.3	1075	2.5	300	Brunisolic Gray Luvisol	Douglas fir	Dfb, Humid Continental warm summer	OM3	C1	0	A horizon	23.9	NA	NA	0.0	NA	NA
OC353B	OL353	IDF_BC_	British Columbia	OC	Forest Soil	Whole Community DNA	PCR	Amplicon	pyrotag library	454 GS FLX Titanium	European Nucleotide Archive	https://www.ebi.ac.uk/ena/data/search?query=PRJEB12501	ERS1040443	SAMEA3733294	ERX1298780	ERR1226568	16S rRNA	V1-V3	NA	AGAGTTTGATCMTGGCTCAG	GWATTACCGCGGCKGCTG	2010-06-26	50.88	-120.35	Canada	0.3	1075	2.5	300	Brunisolic Gray Luvisol	Douglas fir	Dfb, Humid Continental warm summer	OM3	C1	0	A horizon	18.4	NA	NA	0.0	NA	NA
OC354B	OL354	IDF_BC_	British Columbia	OC	Forest Soil	Whole Community DNA	PCR	Amplicon	pyrotag library	454 GS FLX Titanium	European Nucleotide Archive	https://www.ebi.ac.uk/ena/data/search?query=PRJEB12501	ERS1040444	SAMEA3733295	ERX1298781	ERR1226569	16S rRNA	V1-V3	NA	AGAGTTTGATCMTGGCTCAG	GWATTACCGCGGCKGCTG	2010-06-26	50.88	-120.35	Canada	0.3	1075	2.5	300	Brunisolic Gray Luvisol	Douglas fir	Dfb, Humid Continental warm summer	OM3	C1	0	A horizon	18.9	NA	NA	0.0	NA	NA
OC355B	OL355	IDF_BC_	British Columbia	OC	Forest Soil	Whole Community DNA	PCR	Amplicon	pyrotag library	454 GS FLX Titanium	European Nucleotide Archive	https://www.ebi.ac.uk/ena/data/search?query=PRJEB12501	ERS1040445	SAMEA3733296	ERX1298782	ERR1226570	16S rRNA	V1-V3	NA	AGAGTTTGATCMTGGCTCAG	GWATTACCGCGGCKGCTG	2010-06-26	50.88	-120.35	Canada	0.1	1075	2.5	300	Brunisolic Gray Luvisol	Douglas fir	Dfb, Humid Continental warm summer	REF	REF	0	O horizon	56.5	NA	NA	0.0	NA	NA
OC356B	OL356	IDF_BC_	British Columbia	OC	Forest Soil	Whole Community DNA	PCR	Amplicon	pyrotag library	454 GS FLX Titanium	European Nucleotide Archive	https://www.ebi.ac.uk/ena/data/search?query=PRJEB12501	ERS1040446	SAMEA3733297	ERX1298783	ERR1226571	16S rRNA	V1-V3	NA	AGAGTTTGATCMTGGCTCAG	GWATTACCGCGGCKGCTG	2010-06-26	50.88	-120.35	Canada	0.1	1075	2.5	300	Brunisolic Gray Luvisol	Douglas fir	Dfb, Humid Continental warm summer	REF	REF	0	O horizon	62.0	NA	NA	0.0	NA	NA
OC357B	OL357	IDF_BC_	British Columbia	OC	Forest Soil	Whole Community DNA	PCR	Amplicon	pyrotag library	454 GS FLX Titanium	European Nucleotide Archive	https://www.ebi.ac.uk/ena/data/search?query=PRJEB12501	ERS1040447	SAMEA3733298	ERX1298784	ERR1226572	16S rRNA	V1-V3	NA	AGAGTTTGATCMTGGCTCAG	GWATTACCGCGGCKGCTG	2010-06-26	50.88	-120.35	Canada	0.1	1075	2.5	300	Brunisolic Gray Luvisol	Douglas fir	Dfb, Humid Continental warm summer	REF	REF	0	O horizon	62.1	NA	NA	0.0	NA	NA
OC358B	OL358	IDF_BC_	British Columbia	OC	Forest Soil	Whole Community DNA	PCR	Amplicon	pyrotag library	454 GS FLX Titanium	European Nucleotide Archive	https://www.ebi.ac.uk/ena/data/search?query=PRJEB12501	ERS1040448	SAMEA3733299	ERX1298785	ERR1226573	16S rRNA	V1-V3	NA	AGAGTTTGATCMTGGCTCAG	GWATTACCGCGGCKGCTG	2010-06-26	50.88	-120.35	Canada	0.3	1075	2.5	300	Brunisolic Gray Luvisol	Douglas fir	Dfb, Humid Continental warm summer	REF	REF	0	A horizon	23.0	NA	NA	0.0	NA	NA
OC359B	OL359	IDF_BC_	British Columbia	OC	Forest Soil	Whole Community DNA	PCR	Amplicon	pyrotag library	454 GS FLX Titanium	European Nucleotide Archive	https://www.ebi.ac.uk/ena/data/search?query=PRJEB12501	ERS1040449	SAMEA3733300	ERX1298786	ERR1226574	16S rRNA	V1-V3	NA	AGAGTTTGATCMTGGCTCAG	GWATTACCGCGGCKGCTG	2010-06-26	50.88	-120.35	Canada	0.3	1075	2.5	300	Brunisolic Gray Luvisol	Douglas fir	Dfb, Humid Continental warm summer	REF	REF	0	A horizon	20.6	NA	NA	0.0	NA	NA
OC360B	OL360	IDF_BC_	British Columbia	OC	Forest Soil	Whole Community DNA	PCR	Amplicon	pyrotag library	454 GS FLX Titanium	European Nucleotide Archive	https://www.ebi.ac.uk/ena/data/search?query=PRJEB12501	ERS1040450	SAMEA3733301	ERX1298787	ERR1226575	16S rRNA	V1-V3	NA	AGAGTTTGATCMTGGCTCAG	GWATTACCGCGGCKGCTG	2010-06-26	50.88	-120.35	Canada	0.3	1075	2.5	300	Brunisolic Gray Luvisol	Douglas fir	Dfb, Humid Continental warm summer	REF	REF	0	A horizon	20.8	NA	NA	0.0	NA	NA
SL121B	SL121	SBS_BC_	British Columbia	SL	Forest Soil	Whole Community DNA	PCR	Amplicon	pyrotag library	454 GS FLX Titanium	European Nucleotide Archive	https://www.ebi.ac.uk/ena/data/search?query=PRJEB12501	ERS1040451	SAMEA3733302	ERX1298788	ERR1226576	16S rRNA	V1-V3	NA	AGAGTTTGATCMTGGCTCAG	GWATTACCGCGGCKGCTG	2009-08-14	52.32	-121.92	Canada	0.1	1050	3.8	146-193	Orthic Gray Luvisol	Lodgepole pine, Interior spruce	Dfc, Boreal cool summer	OM1	C0	0	O horizon	63.0	20.7	0.6	5.2	1.6	35.0
SL122B	SL122	SBS_BC_	British Columbia	SL	Forest Soil	Whole Community DNA	PCR	Amplicon	pyrotag library	454 GS FLX Titanium	European Nucleotide Archive	https://www.ebi.ac.uk/ena/data/search?query=PRJEB12501	ERS1040452	SAMEA3733303	ERX1298789	ERR1226577	16S rRNA	V1-V3	NA	AGAGTTTGATCMTGGCTCAG	GWATTACCGCGGCKGCTG	2009-08-14	52.32	-121.92	Canada	0.1	1050	3.8	146-193	Orthic Gray Luvisol	Lodgepole pine, Interior spruce	Dfc, Boreal cool summer	OM1	C0	0	O horizon	57.0	20.7	0.6	5.2	1.6	35.0
SL123B	SL123	SBS_BC_	British Columbia	SL	Forest Soil	Whole Community DNA	PCR	Amplicon	pyrotag library	454 GS FLX Titanium	European Nucleotide Archive	https://www.ebi.ac.uk/ena/data/search?query=PRJEB12501	ERS1040453	SAMEA3733304	ERX1298790	ERR1226578	16S rRNA	V1-V3	NA	AGAGTTTGATCMTGGCTCAG	GWATTACCGCGGCKGCTG	2009-08-14	52.32	-121.92	Canada	0.1	1050	3.8	146-193	Orthic Gray Luvisol	Lodgepole pine, Interior spruce	Dfc, Boreal cool summer	OM1	C0	0	O horizon	61.0	20.7	0.6	5.2	1.6	35.0
SL124B	SL124	SBS_BC_	British Columbia	SL	Forest Soil	Whole Community DNA	PCR	Amplicon	pyrotag library	454 GS FLX Titanium	European Nucleotide Archive	https://www.ebi.ac.uk/ena/data/search?query=PRJEB12501	ERS1040454	SAMEA3733305	ERX1298791	ERR1226579	16S rRNA	V1-V3	NA	AGAGTTTGATCMTGGCTCAG	GWATTACCGCGGCKGCTG	2009-08-14	52.32	-121.92	Canada	0.3	1050	3.8	146-193	Orthic Gray Luvisol	Lodgepole pine, Interior spruce	Dfc, Boreal cool summer	OM1	C0	0	A horizon	30.0	0.9	0.1	5.4	1.6	18.4
SL125B	SL125	SBS_BC_	British Columbia	SL	Forest Soil	Whole Community DNA	PCR	Amplicon	pyrotag library	454 GS FLX Titanium	European Nucleotide Archive	https://www.ebi.ac.uk/ena/data/search?query=PRJEB12501	ERS1040455	SAMEA3733306	ERX1298792	ERR1226580	16S rRNA	V1-V3	NA	AGAGTTTGATCMTGGCTCAG	GWATTACCGCGGCKGCTG	2009-08-14	52.32	-121.92	Canada	0.3	1050	3.8	146-193	Orthic Gray Luvisol	Lodgepole pine, Interior spruce	Dfc, Boreal cool summer	OM1	C0	0	A horizon	30.0	0.9	0.1	5.4	1.6	18.4
SL126B	SL126	SBS_BC_	British Columbia	SL	Forest Soil	Whole Community DNA	PCR	Amplicon	pyrotag library	454 GS FLX Titanium	European Nucleotide Archive	https://www.ebi.ac.uk/ena/data/search?query=PRJEB12501	ERS1040456	SAMEA3733307	ERX1298793	ERR1226581	16S rRNA	V1-V3	NA	AGAGTTTGATCMTGGCTCAG	GWATTACCGCGGCKGCTG	2009-08-14	52.32	-121.92	Canada	0.3	1050	3.8	146-193	Orthic Gray Luvisol	Lodgepole pine, Interior spruce	Dfc, Boreal cool summer	OM1	C0	0	A horizon	30.0	0.9	0.1	5.4	1.6	18.4
SL127B	SL127	SBS_BC_	British Columbia	SL	Forest Soil	Whole Community DNA	PCR	Amplicon	pyrotag library	454 GS FLX Titanium	European Nucleotide Archive	https://www.ebi.ac.uk/ena/data/search?query=PRJEB12501	ERS1040457	SAMEA3733308	ERX1298794	ERR1226582	16S rRNA	V1-V3	NA	AGAGTTTGATCMTGGCTCAG	GWATTACCGCGGCKGCTG	2009-08-14	52.32	-121.92	Canada	0.1	1050	3.8	146-193	Orthic Gray Luvisol	Lodgepole pine, Interior spruce	Dfc, Boreal cool summer	OM1	C1	0	O horizon	60.0	22.3	0.6	5.1	1.6	38.4
SL128B	SL128	SBS_BC_	British Columbia	SL	Forest Soil	Whole Community DNA	PCR	Amplicon	pyrotag library	454 GS FLX Titanium	European Nucleotide Archive	https://www.ebi.ac.uk/ena/data/search?query=PRJEB12501	ERS1040458	SAMEA3733309	ERX1298795	ERR1226583	16S rRNA	V1-V3	NA	AGAGTTTGATCMTGGCTCAG	GWATTACCGCGGCKGCTG	2009-08-14	52.32	-121.92	Canada	0.1	1050	3.8	146-193	Orthic Gray Luvisol	Lodgepole pine, Interior spruce	Dfc, Boreal cool summer	OM1	C1	0	O horizon	59.0	22.3	0.6	5.1	1.6	38.4
SL129B	SL129	SBS_BC_	British Columbia	SL	Forest Soil	Whole Community DNA	PCR	Amplicon	pyrotag library	454 GS FLX Titanium	European Nucleotide Archive	https://www.ebi.ac.uk/ena/data/search?query=PRJEB12501	ERS1040459	SAMEA3733310	ERX1298796	ERR1226584	16S rRNA	V1-V3	NA	AGAGTTTGATCMTGGCTCAG	GWATTACCGCGGCKGCTG	2009-08-14	52.32	-121.92	Canada	0.1	1050	3.8	146-193	Orthic Gray Luvisol	Lodgepole pine, Interior spruce	Dfc, Boreal cool summer	OM1	C1	0	O horizon	62.0	22.3	0.6	5.1	1.6	38.4
SL130B	SL130	SBS_BC_	British Columbia	SL	Forest Soil	Whole Community DNA	PCR	Amplicon	pyrotag library	454 GS FLX Titanium	European Nucleotide Archive	https://www.ebi.ac.uk/ena/data/search?query=PRJEB12501	ERS1040460	SAMEA3733311	ERX1298797	ERR1226585	16S rRNA	V1-V3	NA	AGAGTTTGATCMTGGCTCAG	GWATTACCGCGGCKGCTG	2009-08-14	52.32	-121.92	Canada	0.3	1050	3.8	146-193	Orthic Gray Luvisol	Lodgepole pine, Interior spruce	Dfc, Boreal cool summer	OM1	C1	0	A horizon	32.0	1.0	0.1	5.5	1.6	19.4
SL131B	SL131	SBS_BC_	British Columbia	SL	Forest Soil	Whole Community DNA	PCR	Amplicon	pyrotag library	454 GS FLX Titanium	European Nucleotide Archive	https://www.ebi.ac.uk/ena/data/search?query=PRJEB12501	ERS1040461	SAMEA3733312	ERX1298798	ERR1226586	16S rRNA	V1-V3	NA	AGAGTTTGATCMTGGCTCAG	GWATTACCGCGGCKGCTG	2009-08-14	52.32	-121.92	Canada	0.3	1050	3.8	146-193	Orthic Gray Luvisol	Lodgepole pine, Interior spruce	Dfc, Boreal cool summer	OM1	C1	0	A horizon	32.0	1.0	0.1	5.5	1.6	19.4
SL132B	SL132	SBS_BC_	British Columbia	SL	Forest Soil	Whole Community DNA	PCR	Amplicon	pyrotag library	454 GS FLX Titanium	European Nucleotide Archive	https://www.ebi.ac.uk/ena/data/search?query=PRJEB12501	ERS1040462	SAMEA3733313	ERX1298799	ERR1226587	16S rRNA	V1-V3	NA	AGAGTTTGATCMTGGCTCAG	GWATTACCGCGGCKGCTG	2009-08-14	52.32	-121.92	Canada	0.3	1050	3.8	146-193	Orthic Gray Luvisol	Lodgepole pine, Interior spruce	Dfc, Boreal cool summer	OM1	C1	0	A horizon	36.0	1.0	0.1	5.5	1.6	19.4
SL133B	SL133	SBS_BC_	British Columbia	SL	Forest Soil	Whole Community DNA	PCR	Amplicon	pyrotag library	454 GS FLX Titanium	European Nucleotide Archive	https://www.ebi.ac.uk/ena/data/search?query=PRJEB12501	ERS1040463	SAMEA3733314	ERX1298800	ERR1226588	16S rRNA	V1-V3	NA	AGAGTTTGATCMTGGCTCAG	GWATTACCGCGGCKGCTG	2009-08-14	52.32	-121.92	Canada	0.1	1050	3.8	146-193	Orthic Gray Luvisol	Lodgepole pine, Interior spruce	Dfc, Boreal cool summer	OM1	C2	0	O horizon	50.0	18.8	0.5	5.3	1.8	36.2
SL134B	SL134	SBS_BC_	British Columbia	SL	Forest Soil	Whole Community DNA	PCR	Amplicon	pyrotag library	454 GS FLX Titanium	European Nucleotide Archive	https://www.ebi.ac.uk/ena/data/search?query=PRJEB12501	ERS1040464	SAMEA3733315	ERX1298801	ERR1226589	16S rRNA	V1-V3	NA	AGAGTTTGATCMTGGCTCAG	GWATTACCGCGGCKGCTG	2009-08-14	52.32	-121.92	Canada	0.1	1050	3.8	146-193	Orthic Gray Luvisol	Lodgepole pine, Interior spruce	Dfc, Boreal cool summer	OM1	C2	0	O horizon	68.0	18.8	0.5	5.3	1.8	36.2
SL135B	SL135	SBS_BC_	British Columbia	SL	Forest Soil	Whole Community DNA	PCR	Amplicon	pyrotag library	454 GS FLX Titanium	European Nucleotide Archive	https://www.ebi.ac.uk/ena/data/search?query=PRJEB12501	ERS1040465	SAMEA3733316	ERX1298802	ERR1226590	16S rRNA	V1-V3	NA	AGAGTTTGATCMTGGCTCAG	GWATTACCGCGGCKGCTG	2009-08-14	52.32	-121.92	Canada	0.1	1050	3.8	146-193	Orthic Gray Luvisol	Lodgepole pine, Interior spruce	Dfc, Boreal cool summer	OM1	C2	0	O horizon	67.0	18.8	0.5	5.3	1.8	36.2
SL136B	SL136	SBS_BC_	British Columbia	SL	Forest Soil	Whole Community DNA	PCR	Amplicon	pyrotag library	454 GS FLX Titanium	European Nucleotide Archive	https://www.ebi.ac.uk/ena/data/search?query=PRJEB12501	ERS1040466	SAMEA3733317	ERX1298803	ERR1226591	16S rRNA	V1-V3	NA	AGAGTTTGATCMTGGCTCAG	GWATTACCGCGGCKGCTG	2009-08-14	52.32	-121.92	Canada	0.3	1050	3.8	146-193	Orthic Gray Luvisol	Lodgepole pine, Interior spruce	Dfc, Boreal cool summer	OM1	C2	0	A horizon	31.0	0.8	0.1	5.6	1.8	16.2
SL137B	SL137	SBS_BC_	British Columbia	SL	Forest Soil	Whole Community DNA	PCR	Amplicon	pyrotag library	454 GS FLX Titanium	European Nucleotide Archive	https://www.ebi.ac.uk/ena/data/search?query=PRJEB12501	ERS1040467	SAMEA3733318	ERX1298804	ERR1226592	16S rRNA	V1-V3	NA	AGAGTTTGATCMTGGCTCAG	GWATTACCGCGGCKGCTG	2009-08-14	52.32	-121.92	Canada	0.3	1050	3.8	146-193	Orthic Gray Luvisol	Lodgepole pine, Interior spruce	Dfc, Boreal cool summer	OM1	C2	0	A horizon	33.0	0.8	0.1	5.6	1.8	16.2
SL138B	SL138	SBS_BC_	British Columbia	SL	Forest Soil	Whole Community DNA	PCR	Amplicon	pyrotag library	454 GS FLX Titanium	European Nucleotide Archive	https://www.ebi.ac.uk/ena/data/search?query=PRJEB12501	ERS1040468	SAMEA3733319	ERX1298805	ERR1226593	16S rRNA	V1-V3	NA	AGAGTTTGATCMTGGCTCAG	GWATTACCGCGGCKGCTG	2009-08-14	52.32	-121.92	Canada	0.3	1050	3.8	146-193	Orthic Gray Luvisol	Lodgepole pine, Interior spruce	Dfc, Boreal cool summer	OM1	C2	0	A horizon	32.0	0.8	0.1	5.6	1.8	16.2
SL139B	SL139	SBS_BC_	British Columbia	SL	Forest Soil	Whole Community DNA	PCR	Amplicon	pyrotag library	454 GS FLX Titanium	European Nucleotide Archive	https://www.ebi.ac.uk/ena/data/search?query=PRJEB12501	ERS1040469	SAMEA3733320	ERX1298806	ERR1226594	16S rRNA	V1-V3	NA	AGAGTTTGATCMTGGCTCAG	GWATTACCGCGGCKGCTG	2009-08-14	52.32	-121.92	Canada	0.1	1050	3.8	146-193	Orthic Gray Luvisol	Lodgepole pine, Interior spruce	Dfc, Boreal cool summer	OM2	C0	0	O horizon	56.0	17.5	0.5	5.2	1.7	34.3
SL140B	SL140	SBS_BC_	British Columbia	SL	Forest Soil	Whole Community DNA	PCR	Amplicon	pyrotag library	454 GS FLX Titanium	European Nucleotide Archive	https://www.ebi.ac.uk/ena/data/search?query=PRJEB12501	ERS1040470	SAMEA3733321	ERX1298807	ERR1226595	16S rRNA	V1-V3	NA	AGAGTTTGATCMTGGCTCAG	GWATTACCGCGGCKGCTG	2009-08-14	52.32	-121.92	Canada	0.1	1050	3.8	146-193	Orthic Gray Luvisol	Lodgepole pine, Interior spruce	Dfc, Boreal cool summer	OM2	C0	0	O horizon	57.0	17.5	0.5	5.2	1.7	34.3
SL141B	SL141	SBS_BC_	British Columbia	SL	Forest Soil	Whole Community DNA	PCR	Amplicon	pyrotag library	454 GS FLX Titanium	European Nucleotide Archive	https://www.ebi.ac.uk/ena/data/search?query=PRJEB12501	ERS1040471	SAMEA3733322	ERX1298808	ERR1226596	16S rRNA	V1-V3	NA	AGAGTTTGATCMTGGCTCAG	GWATTACCGCGGCKGCTG	2009-08-14	52.32	-121.92	Canada	0.1	1050	3.8	146-193	Orthic Gray Luvisol	Lodgepole pine, Interior spruce	Dfc, Boreal cool summer	OM2	C0	0	O horizon	58.0	17.5	0.5	5.2	1.7	34.3
SL142B	SL142	SBS_BC_	British Columbia	SL	Forest Soil	Whole Community DNA	PCR	Amplicon	pyrotag library	454 GS FLX Titanium	European Nucleotide Archive	https://www.ebi.ac.uk/ena/data/search?query=PRJEB12501	ERS1040472	SAMEA3733323	ERX1298809	ERR1226597	16S rRNA	V1-V3	NA	AGAGTTTGATCMTGGCTCAG	GWATTACCGCGGCKGCTG	2009-08-14	52.32	-121.92	Canada	0.3	1050	3.8	146-193	Orthic Gray Luvisol	Lodgepole pine, Interior spruce	Dfc, Boreal cool summer	OM2	C0	0	A horizon	33.0	0.9	0.1	5.5	1.7	18.2
SL143B	SL143	SBS_BC_	British Columbia	SL	Forest Soil	Whole Community DNA	PCR	Amplicon	pyrotag library	454 GS FLX Titanium	European Nucleotide Archive	https://www.ebi.ac.uk/ena/data/search?query=PRJEB12501	ERS1040473	SAMEA3733324	ERX1298810	ERR1226598	16S rRNA	V1-V3	NA	AGAGTTTGATCMTGGCTCAG	GWATTACCGCGGCKGCTG	2009-08-14	52.32	-121.92	Canada	0.3	1050	3.8	146-193	Orthic Gray Luvisol	Lodgepole pine, Interior spruce	Dfc, Boreal cool summer	OM2	C0	0	A horizon	35.0	0.9	0.1	5.5	1.7	18.2
SL144B	SL144	SBS_BC_	British Columbia	SL	Forest Soil	Whole Community DNA	PCR	Amplicon	pyrotag library	454 GS FLX Titanium	European Nucleotide Archive	https://www.ebi.ac.uk/ena/data/search?query=PRJEB12501	ERS1040474	SAMEA3733325	ERX1298811	ERR1226599	16S rRNA	V1-V3	NA	AGAGTTTGATCMTGGCTCAG	GWATTACCGCGGCKGCTG	2009-08-14	52.32	-121.92	Canada	0.3	1050	3.8	146-193	Orthic Gray Luvisol	Lodgepole pine, Interior spruce	Dfc, Boreal cool summer	OM2	C0	0	A horizon	28.0	0.9	0.1	5.5	1.7	18.2
SL145B	SL145	SBS_BC_	British Columbia	SL	Forest Soil	Whole Community DNA	PCR	Amplicon	pyrotag library	454 GS FLX Titanium	European Nucleotide Archive	https://www.ebi.ac.uk/ena/data/search?query=PRJEB12501	ERS1040475	SAMEA3733326	ERX1298812	ERR1226600	16S rRNA	V1-V3	NA	AGAGTTTGATCMTGGCTCAG	GWATTACCGCGGCKGCTG	2009-08-14	52.32	-121.92	Canada	0.1	1050	3.8	146-193	Orthic Gray Luvisol	Lodgepole pine, Interior spruce	Dfc, Boreal cool summer	OM2	C1	0	O horizon	55.0	19.9	0.5	5.4	1.7	39.0
SL146B	SL146	SBS_BC_	British Columbia	SL	Forest Soil	Whole Community DNA	PCR	Amplicon	pyrotag library	454 GS FLX Titanium	European Nucleotide Archive	https://www.ebi.ac.uk/ena/data/search?query=PRJEB12501	ERS1040476	SAMEA3733327	ERX1298813	ERR1226601	16S rRNA	V1-V3	NA	AGAGTTTGATCMTGGCTCAG	GWATTACCGCGGCKGCTG	2009-08-14	52.32	-121.92	Canada	0.1	1050	3.8	146-193	Orthic Gray Luvisol	Lodgepole pine, Interior spruce	Dfc, Boreal cool summer	OM2	C1	0	O horizon	54.0	19.9	0.5	5.4	1.7	39.0
SL147B	SL147	SBS_BC_	British Columbia	SL	Forest Soil	Whole Community DNA	PCR	Amplicon	pyrotag library	454 GS FLX Titanium	European Nucleotide Archive	https://www.ebi.ac.uk/ena/data/search?query=PRJEB12501	ERS1040477	SAMEA3733328	ERX1298814	ERR1226602	16S rRNA	V1-V3	NA	AGAGTTTGATCMTGGCTCAG	GWATTACCGCGGCKGCTG	2009-08-14	52.32	-121.92	Canada	0.1	1050	3.8	146-193	Orthic Gray Luvisol	Lodgepole pine, Interior spruce	Dfc, Boreal cool summer	OM2	C1	0	O horizon	62.0	19.9	0.5	5.4	1.7	39.0
SL148B	SL148	SBS_BC_	British Columbia	SL	Forest Soil	Whole Community DNA	PCR	Amplicon	pyrotag library	454 GS FLX Titanium	European Nucleotide Archive	https://www.ebi.ac.uk/ena/data/search?query=PRJEB12501	ERS1040478	SAMEA3733329	ERX1298815	ERR1226603	16S rRNA	V1-V3	NA	AGAGTTTGATCMTGGCTCAG	GWATTACCGCGGCKGCTG	2009-08-14	52.32	-121.92	Canada	0.3	1050	3.8	146-193	Orthic Gray Luvisol	Lodgepole pine, Interior spruce	Dfc, Boreal cool summer	OM2	C1	0	A horizon	33.0	1.4	0.1	5.6	1.7	19.6
SL149B	SL149	SBS_BC_	British Columbia	SL	Forest Soil	Whole Community DNA	PCR	Amplicon	pyrotag library	454 GS FLX Titanium	European Nucleotide Archive	https://www.ebi.ac.uk/ena/data/search?query=PRJEB12501	ERS1040479	SAMEA3733330	ERX1298816	ERR1226604	16S rRNA	V1-V3	NA	AGAGTTTGATCMTGGCTCAG	GWATTACCGCGGCKGCTG	2009-08-14	52.32	-121.92	Canada	0.3	1050	3.8	146-193	Orthic Gray Luvisol	Lodgepole pine, Interior spruce	Dfc, Boreal cool summer	OM2	C1	0	A horizon	32.0	1.4	0.1	5.6	1.7	19.6
SL150B	SL150	SBS_BC_	British Columbia	SL	Forest Soil	Whole Community DNA	PCR	Amplicon	pyrotag library	454 GS FLX Titanium	European Nucleotide Archive	https://www.ebi.ac.uk/ena/data/search?query=PRJEB12501	ERS1040480	SAMEA3733331	ERX1298817	ERR1226605	16S rRNA	V1-V3	NA	AGAGTTTGATCMTGGCTCAG	GWATTACCGCGGCKGCTG	2009-08-14	52.32	-121.92	Canada	0.3	1050	3.8	146-193	Orthic Gray Luvisol	Lodgepole pine, Interior spruce	Dfc, Boreal cool summer	OM2	C1	0	A horizon	31.0	1.4	0.1	5.6	1.7	19.6
SL152B	SL152	SBS_BC_	British Columbia	SL	Forest Soil	Whole Community DNA	PCR	Amplicon	pyrotag library	454 GS FLX Titanium	European Nucleotide Archive	https://www.ebi.ac.uk/ena/data/search?query=PRJEB12501	ERS1040481	SAMEA3733332	ERX1298818	ERR1226606	16S rRNA	V1-V3	NA	AGAGTTTGATCMTGGCTCAG	GWATTACCGCGGCKGCTG	2009-08-14	52.32	-121.92	Canada	0.1	1050	3.8	146-193	Orthic Gray Luvisol	Lodgepole pine, Interior spruce	Dfc, Boreal cool summer	OM2	C2	0	O horizon	54.0	17.9	0.5	5.2	1.7	33.2
SL153B	SL153	SBS_BC_	British Columbia	SL	Forest Soil	Whole Community DNA	PCR	Amplicon	pyrotag library	454 GS FLX Titanium	European Nucleotide Archive	https://www.ebi.ac.uk/ena/data/search?query=PRJEB12501	ERS1040482	SAMEA3733333	ERX1298819	ERR1226607	16S rRNA	V1-V3	NA	AGAGTTTGATCMTGGCTCAG	GWATTACCGCGGCKGCTG	2009-08-14	52.32	-121.92	Canada	0.1	1050	3.8	146-193	Orthic Gray Luvisol	Lodgepole pine, Interior spruce	Dfc, Boreal cool summer	OM2	C2	0	O horizon	63.0	17.9	0.5	5.2	1.7	33.2
SL154B	SL154	SBS_BC_	British Columbia	SL	Forest Soil	Whole Community DNA	PCR	Amplicon	pyrotag library	454 GS FLX Titanium	European Nucleotide Archive	https://www.ebi.ac.uk/ena/data/search?query=PRJEB12501	ERS1040483	SAMEA3733334	ERX1298820	ERR1226608	16S rRNA	V1-V3	NA	AGAGTTTGATCMTGGCTCAG	GWATTACCGCGGCKGCTG	2009-08-14	52.32	-121.92	Canada	0.3	1050	3.8	146-193	Orthic Gray Luvisol	Lodgepole pine, Interior spruce	Dfc, Boreal cool summer	OM2	C2	0	A horizon	28.0	1.1	0.1	5.5	1.7	17.8
SL155B	SL155	SBS_BC_	British Columbia	SL	Forest Soil	Whole Community DNA	PCR	Amplicon	pyrotag library	454 GS FLX Titanium	European Nucleotide Archive	https://www.ebi.ac.uk/ena/data/search?query=PRJEB12501	ERS1040484	SAMEA3733335	ERX1298821	ERR1226609	16S rRNA	V1-V3	NA	AGAGTTTGATCMTGGCTCAG	GWATTACCGCGGCKGCTG	2009-08-14	52.32	-121.92	Canada	0.3	1050	3.8	146-193	Orthic Gray Luvisol	Lodgepole pine, Interior spruce	Dfc, Boreal cool summer	OM2	C2	0	A horizon	34.0	1.1	0.1	5.5	1.7	17.8
SL156B	SL156	SBS_BC_	British Columbia	SL	Forest Soil	Whole Community DNA	PCR	Amplicon	pyrotag library	454 GS FLX Titanium	European Nucleotide Archive	https://www.ebi.ac.uk/ena/data/search?query=PRJEB12501	ERS1040485	SAMEA3733336	ERX1298822	ERR1226610	16S rRNA	V1-V3	NA	AGAGTTTGATCMTGGCTCAG	GWATTACCGCGGCKGCTG	2009-08-14	52.32	-121.92	Canada	0.3	1050	3.8	146-193	Orthic Gray Luvisol	Lodgepole pine, Interior spruce	Dfc, Boreal cool summer	OM2	C2	0	A horizon	32.0	1.1	0.1	5.5	1.7	17.8
SL160B	SL160	SBS_BC_	British Columbia	SL	Forest Soil	Whole Community DNA	PCR	Amplicon	pyrotag library	454 GS FLX Titanium	European Nucleotide Archive	https://www.ebi.ac.uk/ena/data/search?query=PRJEB12501	ERS1040486	SAMEA3733337	ERX1298823	ERR1226611	16S rRNA	V1-V3	NA	AGAGTTTGATCMTGGCTCAG	GWATTACCGCGGCKGCTG	2009-08-14	52.32	-121.92	Canada	0.3	1050	3.8	146-193	Orthic Gray Luvisol	Lodgepole pine, Interior spruce	Dfc, Boreal cool summer	OM3	C0	0	A horizon	33.0	0.9	0.1	5.6	1.7	18.2
SL161B	SL161	SBS_BC_	British Columbia	SL	Forest Soil	Whole Community DNA	PCR	Amplicon	pyrotag library	454 GS FLX Titanium	European Nucleotide Archive	https://www.ebi.ac.uk/ena/data/search?query=PRJEB12501	ERS1040487	SAMEA3733338	ERX1298824	ERR1226612	16S rRNA	V1-V3	NA	AGAGTTTGATCMTGGCTCAG	GWATTACCGCGGCKGCTG	2009-08-14	52.32	-121.92	Canada	0.3	1050	3.8	146-193	Orthic Gray Luvisol	Lodgepole pine, Interior spruce	Dfc, Boreal cool summer	OM3	C0	0	A horizon	33.0	0.9	0.1	5.6	1.7	18.2
SL162B	SL162	SBS_BC_	British Columbia	SL	Forest Soil	Whole Community DNA	PCR	Amplicon	pyrotag library	454 GS FLX Titanium	European Nucleotide Archive	https://www.ebi.ac.uk/ena/data/search?query=PRJEB12501	ERS1040488	SAMEA3733339	ERX1298825	ERR1226613	16S rRNA	V1-V3	NA	AGAGTTTGATCMTGGCTCAG	GWATTACCGCGGCKGCTG	2009-08-14	52.32	-121.92	Canada	0.3	1050	3.8	146-193	Orthic Gray Luvisol	Lodgepole pine, Interior spruce	Dfc, Boreal cool summer	OM3	C0	0	A horizon	33.0	0.9	0.1	5.6	1.7	18.2
SL166B	SL166	SBS_BC_	British Columbia	SL	Forest Soil	Whole Community DNA	PCR	Amplicon	pyrotag library	454 GS FLX Titanium	European Nucleotide Archive	https://www.ebi.ac.uk/ena/data/search?query=PRJEB12501	ERS1040489	SAMEA3733340	ERX1298826	ERR1226614	16S rRNA	V1-V3	NA	AGAGTTTGATCMTGGCTCAG	GWATTACCGCGGCKGCTG	2009-08-14	52.32	-121.92	Canada	0.3	1050	3.8	146-193	Orthic Gray Luvisol	Lodgepole pine, Interior spruce	Dfc, Boreal cool summer	OM3	C1	0	A horizon	32.0	1.1	0.1	5.5	1.7	18.2
SL167B	SL167	SBS_BC_	British Columbia	SL	Forest Soil	Whole Community DNA	PCR	Amplicon	pyrotag library	454 GS FLX Titanium	European Nucleotide Archive	https://www.ebi.ac.uk/ena/data/search?query=PRJEB12501	ERS1040490	SAMEA3733341	ERX1298827	ERR1226615	16S rRNA	V1-V3	NA	AGAGTTTGATCMTGGCTCAG	GWATTACCGCGGCKGCTG	2009-08-14	52.32	-121.92	Canada	0.3	1050	3.8	146-193	Orthic Gray Luvisol	Lodgepole pine, Interior spruce	Dfc, Boreal cool summer	OM3	C1	0	A horizon	32.0	1.1	0.1	5.5	1.7	18.2
SL168B	SL168	SBS_BC_	British Columbia	SL	Forest Soil	Whole Community DNA	PCR	Amplicon	pyrotag library	454 GS FLX Titanium	European Nucleotide Archive	https://www.ebi.ac.uk/ena/data/search?query=PRJEB12501	ERS1040491	SAMEA3733342	ERX1298828	ERR1226616	16S rRNA	V1-V3	NA	AGAGTTTGATCMTGGCTCAG	GWATTACCGCGGCKGCTG	2009-08-14	52.32	-121.92	Canada	0.3	1050	3.8	146-193	Orthic Gray Luvisol	Lodgepole pine, Interior spruce	Dfc, Boreal cool summer	OM3	C1	0	A horizon	31.0	1.1	0.1	5.5	1.7	18.2
SL172B	SL172	SBS_BC_	British Columbia	SL	Forest Soil	Whole Community DNA	PCR	Amplicon	pyrotag library	454 GS FLX Titanium	European Nucleotide Archive	https://www.ebi.ac.uk/ena/data/search?query=PRJEB12501	ERS1040492	SAMEA3733343	ERX1298829	ERR1226617	16S rRNA	V1-V3	NA	AGAGTTTGATCMTGGCTCAG	GWATTACCGCGGCKGCTG	2009-08-14	52.32	-121.92	Canada	0.3	1050	3.8	146-193	Orthic Gray Luvisol	Lodgepole pine, Interior spruce	Dfc, Boreal cool summer	OM3	C2	0	A horizon	32.0	0.9	0.1	5.7	1.7	18.0
SL173B	SL173	SBS_BC_	British Columbia	SL	Forest Soil	Whole Community DNA	PCR	Amplicon	pyrotag library	454 GS FLX Titanium	European Nucleotide Archive	https://www.ebi.ac.uk/ena/data/search?query=PRJEB12501	ERS1040493	SAMEA3733344	ERX1298830	ERR1226618	16S rRNA	V1-V3	NA	AGAGTTTGATCMTGGCTCAG	GWATTACCGCGGCKGCTG	2009-08-14	52.32	-121.92	Canada	0.3	1050	3.8	146-193	Orthic Gray Luvisol	Lodgepole pine, Interior spruce	Dfc, Boreal cool summer	OM3	C2	0	A horizon	30.0	0.9	0.1	5.7	1.7	18.0
SL174B	SL174	SBS_BC_	British Columbia	SL	Forest Soil	Whole Community DNA	PCR	Amplicon	pyrotag library	454 GS FLX Titanium	European Nucleotide Archive	https://www.ebi.ac.uk/ena/data/search?query=PRJEB12501	ERS1040494	SAMEA3733345	ERX1298831	ERR1226619	16S rRNA	V1-V3	NA	AGAGTTTGATCMTGGCTCAG	GWATTACCGCGGCKGCTG	2009-08-14	52.32	-121.92	Canada	0.3	1050	3.8	146-193	Orthic Gray Luvisol	Lodgepole pine, Interior spruce	Dfc, Boreal cool summer	OM3	C2	0	A horizon	32.0	0.9	0.1	5.7	1.7	18.0
SL175B	SL175	SBS_BC_	British Columbia	SL	Forest Soil	Whole Community DNA	PCR	Amplicon	pyrotag library	454 GS FLX Titanium	European Nucleotide Archive	https://www.ebi.ac.uk/ena/data/search?query=PRJEB12501	ERS1040495	SAMEA3733346	ERX1298832	ERR1226620	16S rRNA	V1-V3	NA	AGAGTTTGATCMTGGCTCAG	GWATTACCGCGGCKGCTG	2009-08-14	52.32	-121.92	Canada	0.1	1050	3.8	146-193	Orthic Gray Luvisol	Lodgepole pine, Interior spruce	Dfc, Boreal cool summer	REF	REF	0	O horizon	85.0	18.9	0.6	5.4	NA	33.7
SL176B	SL176	SBS_BC_	British Columbia	SL	Forest Soil	Whole Community DNA	PCR	Amplicon	pyrotag library	454 GS FLX Titanium	European Nucleotide Archive	https://www.ebi.ac.uk/ena/data/search?query=PRJEB12501	ERS1040496	SAMEA3733347	ERX1298833	ERR1226621	16S rRNA	V1-V3	NA	AGAGTTTGATCMTGGCTCAG	GWATTACCGCGGCKGCTG	2009-08-14	52.32	-121.92	Canada	0.1	1050	3.8	146-193	Orthic Gray Luvisol	Lodgepole pine, Interior spruce	Dfc, Boreal cool summer	REF	REF	0	O horizon	73.0	18.9	0.6	5.4	NA	33.7
SL177B	SL177	SBS_BC_	British Columbia	SL	Forest Soil	Whole Community DNA	PCR	Amplicon	pyrotag library	454 GS FLX Titanium	European Nucleotide Archive	https://www.ebi.ac.uk/ena/data/search?query=PRJEB12501	ERS1040497	SAMEA3733348	ERX1298834	ERR1226622	16S rRNA	V1-V3	NA	AGAGTTTGATCMTGGCTCAG	GWATTACCGCGGCKGCTG	2009-08-14	52.32	-121.92	Canada	0.1	1050	3.8	146-193	Orthic Gray Luvisol	Lodgepole pine, Interior spruce	Dfc, Boreal cool summer	REF	REF	0	O horizon	71.0	18.9	0.6	5.4	NA	33.7
SL178B	SL178	SBS_BC_	British Columbia	SL	Forest Soil	Whole Community DNA	PCR	Amplicon	pyrotag library	454 GS FLX Titanium	European Nucleotide Archive	https://www.ebi.ac.uk/ena/data/search?query=PRJEB12501	ERS1040498	SAMEA3733349	ERX1298835	ERR1226623	16S rRNA	V1-V3	NA	AGAGTTTGATCMTGGCTCAG	GWATTACCGCGGCKGCTG	2009-08-14	52.32	-121.92	Canada	0.3	1050	3.8	146-193	Orthic Gray Luvisol	Lodgepole pine, Interior spruce	Dfc, Boreal cool summer	REF	REF	0	A horizon	36.0	0.9	0.1	5.8	NA	17.4
SL179B	SL179	SBS_BC_	British Columbia	SL	Forest Soil	Whole Community DNA	PCR	Amplicon	pyrotag library	454 GS FLX Titanium	European Nucleotide Archive	https://www.ebi.ac.uk/ena/data/search?query=PRJEB12501	ERS1040499	SAMEA3733350	ERX1298836	ERR1226624	16S rRNA	V1-V3	NA	AGAGTTTGATCMTGGCTCAG	GWATTACCGCGGCKGCTG	2009-08-14	52.32	-121.92	Canada	0.3	1050	3.8	146-193	Orthic Gray Luvisol	Lodgepole pine, Interior spruce	Dfc, Boreal cool summer	REF	REF	0	A horizon	36.0	0.9	0.1	5.8	NA	17.4
SL180B	SL180	SBS_BC_	British Columbia	SL	Forest Soil	Whole Community DNA	PCR	Amplicon	pyrotag library	454 GS FLX Titanium	European Nucleotide Archive	https://www.ebi.ac.uk/ena/data/search?query=PRJEB12501	ERS1040500	SAMEA3733351	ERX1298837	ERR1226625	16S rRNA	V1-V3	NA	AGAGTTTGATCMTGGCTCAG	GWATTACCGCGGCKGCTG	2009-08-14	52.32	-121.92	Canada	0.3	1050	3.8	146-193	Orthic Gray Luvisol	Lodgepole pine, Interior spruce	Dfc, Boreal cool summer	REF	REF	0	A horizon	34.0	0.9	0.1	5.8	NA	17.4
TO061B	TO061	SBS_BC_	British Columbia	TO	Forest Soil	Whole Community DNA	PCR	Amplicon	pyrotag library	454 GS FLX Titanium	European Nucleotide Archive	https://www.ebi.ac.uk/ena/data/search?query=PRJEB12501	ERS1040501	SAMEA3733352	ERX1298838	ERR1226626	16S rRNA	V1-V3	NA	AGAGTTTGATCMTGGCTCAG	GWATTACCGCGGCKGCTG	2008-07-11	52.32	-126.31	Canada	0.1	1100	1.7	146-193	Orthic Gray Luvisol, Gleyed Gray Luvisol	Lodgepole pine, Subalpine fir, Interior spruce	Dfc, Boreal cool summer	OM1	C0	0	O horizon	58.0	33.5	1.0	5.1	1.5	32.5
TO062B	TO062	SBS_BC_	British Columbia	TO	Forest Soil	Whole Community DNA	PCR	Amplicon	pyrotag library	454 GS FLX Titanium	European Nucleotide Archive	https://www.ebi.ac.uk/ena/data/search?query=PRJEB12501	ERS1040502	SAMEA3733353	ERX1298839	ERR1226627	16S rRNA	V1-V3	NA	AGAGTTTGATCMTGGCTCAG	GWATTACCGCGGCKGCTG	2008-07-11	52.32	-126.31	Canada	0.1	1100	1.7	146-193	Orthic Gray Luvisol, Gleyed Gray Luvisol	Lodgepole pine, Subalpine fir, Interior spruce	Dfc, Boreal cool summer	OM1	C0	0	O horizon	52.0	33.5	1.0	5.1	1.5	32.5
TO063B	TO063	SBS_BC_	British Columbia	TO	Forest Soil	Whole Community DNA	PCR	Amplicon	pyrotag library	454 GS FLX Titanium	European Nucleotide Archive	https://www.ebi.ac.uk/ena/data/search?query=PRJEB12501	ERS1040503	SAMEA3733354	ERX1298840	ERR1226628	16S rRNA	V1-V3	NA	AGAGTTTGATCMTGGCTCAG	GWATTACCGCGGCKGCTG	2008-07-11	52.32	-126.31	Canada	0.1	1100	1.7	146-193	Orthic Gray Luvisol, Gleyed Gray Luvisol	Lodgepole pine, Subalpine fir, Interior spruce	Dfc, Boreal cool summer	OM1	C0	0	O horizon	56.0	33.5	1.0	5.1	1.5	32.5
TO064B	TO064	SBS_BC_	British Columbia	TO	Forest Soil	Whole Community DNA	PCR	Amplicon	pyrotag library	454 GS FLX Titanium	European Nucleotide Archive	https://www.ebi.ac.uk/ena/data/search?query=PRJEB12501	ERS1040504	SAMEA3733355	ERX1298841	ERR1226629	16S rRNA	V1-V3	NA	AGAGTTTGATCMTGGCTCAG	GWATTACCGCGGCKGCTG	2008-07-11	52.32	-126.31	Canada	0.3	1100	1.7	146-193	Orthic Gray Luvisol, Gleyed Gray Luvisol	Lodgepole pine, Subalpine fir, Interior spruce	Dfc, Boreal cool summer	OM1	C0	0	A horizon	20.0	2.2	0.1	5.7	1.5	21.6
TO065B	TO065	SBS_BC_	British Columbia	TO	Forest Soil	Whole Community DNA	PCR	Amplicon	pyrotag library	454 GS FLX Titanium	European Nucleotide Archive	https://www.ebi.ac.uk/ena/data/search?query=PRJEB12501	ERS1040505	SAMEA3733356	ERX1298842	ERR1226630	16S rRNA	V1-V3	NA	AGAGTTTGATCMTGGCTCAG	GWATTACCGCGGCKGCTG	2008-07-11	52.32	-126.31	Canada	0.3	1100	1.7	146-193	Orthic Gray Luvisol, Gleyed Gray Luvisol	Lodgepole pine, Subalpine fir, Interior spruce	Dfc, Boreal cool summer	OM1	C0	0	A horizon	17.0	2.2	0.1	5.7	1.5	21.6
TO066B	TO066	SBS_BC_	British Columbia	TO	Forest Soil	Whole Community DNA	PCR	Amplicon	pyrotag library	454 GS FLX Titanium	European Nucleotide Archive	https://www.ebi.ac.uk/ena/data/search?query=PRJEB12501	ERS1040506	SAMEA3733357	ERX1298843	ERR1226631	16S rRNA	V1-V3	NA	AGAGTTTGATCMTGGCTCAG	GWATTACCGCGGCKGCTG	2008-07-11	52.32	-126.31	Canada	0.3	1100	1.7	146-193	Orthic Gray Luvisol, Gleyed Gray Luvisol	Lodgepole pine, Subalpine fir, Interior spruce	Dfc, Boreal cool summer	OM1	C0	0	A horizon	15.0	2.2	0.1	5.7	1.5	21.6
TO067B	TO067	SBS_BC_	British Columbia	TO	Forest Soil	Whole Community DNA	PCR	Amplicon	pyrotag library	454 GS FLX Titanium	European Nucleotide Archive	https://www.ebi.ac.uk/ena/data/search?query=PRJEB12501	ERS1040507	SAMEA3733358	ERX1298844	ERR1226632	16S rRNA	V1-V3	NA	AGAGTTTGATCMTGGCTCAG	GWATTACCGCGGCKGCTG	2008-07-11	52.32	-126.31	Canada	0.1	1100	1.7	146-193	Orthic Gray Luvisol, Gleyed Gray Luvisol	Lodgepole pine, Subalpine fir, Interior spruce	Dfc, Boreal cool summer	OM2	C0	0	O horizon	58.0	26.4	0.9	4.8	1.5	28.7
TO068B	TO068	SBS_BC_	British Columbia	TO	Forest Soil	Whole Community DNA	PCR	Amplicon	pyrotag library	454 GS FLX Titanium	European Nucleotide Archive	https://www.ebi.ac.uk/ena/data/search?query=PRJEB12501	ERS1040508	SAMEA3733359	ERX1298845	ERR1226633	16S rRNA	V1-V3	NA	AGAGTTTGATCMTGGCTCAG	GWATTACCGCGGCKGCTG	2008-07-11	52.32	-126.31	Canada	0.1	1100	1.7	146-193	Orthic Gray Luvisol, Gleyed Gray Luvisol	Lodgepole pine, Subalpine fir, Interior spruce	Dfc, Boreal cool summer	OM2	C0	0	O horizon	47.0	26.4	0.9	4.8	1.5	28.7
TO069B	TO069	SBS_BC_	British Columbia	TO	Forest Soil	Whole Community DNA	PCR	Amplicon	pyrotag library	454 GS FLX Titanium	European Nucleotide Archive	https://www.ebi.ac.uk/ena/data/search?query=PRJEB12501	ERS1040509	SAMEA3733360	ERX1298846	ERR1226634	16S rRNA	V1-V3	NA	AGAGTTTGATCMTGGCTCAG	GWATTACCGCGGCKGCTG	2008-07-11	52.32	-126.31	Canada	0.1	1100	1.7	146-193	Orthic Gray Luvisol, Gleyed Gray Luvisol	Lodgepole pine, Subalpine fir, Interior spruce	Dfc, Boreal cool summer	OM2	C0	0	O horizon	43.0	26.4	0.9	4.8	1.5	28.7
TO070B	TO070	SBS_BC_	British Columbia	TO	Forest Soil	Whole Community DNA	PCR	Amplicon	pyrotag library	454 GS FLX Titanium	European Nucleotide Archive	https://www.ebi.ac.uk/ena/data/search?query=PRJEB12501	ERS1040510	SAMEA3733361	ERX1298847	ERR1226635	16S rRNA	V1-V3	NA	AGAGTTTGATCMTGGCTCAG	GWATTACCGCGGCKGCTG	2008-07-11	52.32	-126.31	Canada	0.3	1100	1.7	146-193	Orthic Gray Luvisol, Gleyed Gray Luvisol	Lodgepole pine, Subalpine fir, Interior spruce	Dfc, Boreal cool summer	OM2	C0	0	A horizon	25.0	3.3	0.2	5.0	1.5	20.3
TO071B	TO071	SBS_BC_	British Columbia	TO	Forest Soil	Whole Community DNA	PCR	Amplicon	pyrotag library	454 GS FLX Titanium	European Nucleotide Archive	https://www.ebi.ac.uk/ena/data/search?query=PRJEB12501	ERS1040511	SAMEA3733362	ERX1298848	ERR1226636	16S rRNA	V1-V3	NA	AGAGTTTGATCMTGGCTCAG	GWATTACCGCGGCKGCTG	2008-07-11	52.32	-126.31	Canada	0.3	1100	1.7	146-193	Orthic Gray Luvisol, Gleyed Gray Luvisol	Lodgepole pine, Subalpine fir, Interior spruce	Dfc, Boreal cool summer	OM2	C0	0	A horizon	19.0	3.3	0.2	5.0	1.5	20.3
TO072B	TO072	SBS_BC_	British Columbia	TO	Forest Soil	Whole Community DNA	PCR	Amplicon	pyrotag library	454 GS FLX Titanium	European Nucleotide Archive	https://www.ebi.ac.uk/ena/data/search?query=PRJEB12501	ERS1040512	SAMEA3733363	ERX1298849	ERR1226637	16S rRNA	V1-V3	NA	AGAGTTTGATCMTGGCTCAG	GWATTACCGCGGCKGCTG	2008-07-11	52.32	-126.31	Canada	0.3	1100	1.7	146-193	Orthic Gray Luvisol, Gleyed Gray Luvisol	Lodgepole pine, Subalpine fir, Interior spruce	Dfc, Boreal cool summer	OM2	C0	0	A horizon	27.0	3.3	0.2	5.0	1.5	20.3
TO076B	TO076	SBS_BC_	British Columbia	TO	Forest Soil	Whole Community DNA	PCR	Amplicon	pyrotag library	454 GS FLX Titanium	European Nucleotide Archive	https://www.ebi.ac.uk/ena/data/search?query=PRJEB12501	ERS1040513	SAMEA3733364	ERX1298850	ERR1226638	16S rRNA	V1-V3	NA	AGAGTTTGATCMTGGCTCAG	GWATTACCGCGGCKGCTG	2008-07-11	52.32	-126.31	Canada	0.3	1100	1.7	146-193	Orthic Gray Luvisol, Gleyed Gray Luvisol	Lodgepole pine, Subalpine fir, Interior spruce	Dfc, Boreal cool summer	OM3	C0	0	A horizon	20.0	2.2	0.1	5.0	1.6	24.3
TO077B	TO077	SBS_BC_	British Columbia	TO	Forest Soil	Whole Community DNA	PCR	Amplicon	pyrotag library	454 GS FLX Titanium	European Nucleotide Archive	https://www.ebi.ac.uk/ena/data/search?query=PRJEB12501	ERS1040514	SAMEA3733365	ERX1298851	ERR1226639	16S rRNA	V1-V3	NA	AGAGTTTGATCMTGGCTCAG	GWATTACCGCGGCKGCTG	2008-07-11	52.32	-126.31	Canada	0.3	1100	1.7	146-193	Orthic Gray Luvisol, Gleyed Gray Luvisol	Lodgepole pine, Subalpine fir, Interior spruce	Dfc, Boreal cool summer	OM3	C0	0	A horizon	19.0	2.2	0.1	5.0	1.6	24.3
TO078B	TO078	SBS_BC_	British Columbia	TO	Forest Soil	Whole Community DNA	PCR	Amplicon	pyrotag library	454 GS FLX Titanium	European Nucleotide Archive	https://www.ebi.ac.uk/ena/data/search?query=PRJEB12501	ERS1040515	SAMEA3733366	ERX1298852	ERR1226640	16S rRNA	V1-V3	NA	AGAGTTTGATCMTGGCTCAG	GWATTACCGCGGCKGCTG	2008-07-11	52.32	-126.31	Canada	0.3	1100	1.7	146-193	Orthic Gray Luvisol, Gleyed Gray Luvisol	Lodgepole pine, Subalpine fir, Interior spruce	Dfc, Boreal cool summer	OM3	C0	0	A horizon	18.0	2.2	0.1	5.0	1.6	24.3
TO079B	TO079	SBS_BC_	British Columbia	TO	Forest Soil	Whole Community DNA	PCR	Amplicon	pyrotag library	454 GS FLX Titanium	European Nucleotide Archive	https://www.ebi.ac.uk/ena/data/search?query=PRJEB12501	ERS1040516	SAMEA3733367	ERX1298853	ERR1226641	16S rRNA	V1-V3	NA	AGAGTTTGATCMTGGCTCAG	GWATTACCGCGGCKGCTG	2008-07-11	52.32	-126.31	Canada	0.1	1100	1.7	146-193	Orthic Gray Luvisol, Gleyed Gray Luvisol	Lodgepole pine, Subalpine fir, Interior spruce	Dfc, Boreal cool summer	OM1	C1	0	O horizon	52.0	27.6	0.8	4.7	1.8	34.9
TO080B	TO080	SBS_BC_	British Columbia	TO	Forest Soil	Whole Community DNA	PCR	Amplicon	pyrotag library	454 GS FLX Titanium	European Nucleotide Archive	https://www.ebi.ac.uk/ena/data/search?query=PRJEB12501	ERS1040517	SAMEA3733368	ERX1298854	ERR1226642	16S rRNA	V1-V3	NA	AGAGTTTGATCMTGGCTCAG	GWATTACCGCGGCKGCTG	2008-07-11	52.32	-126.31	Canada	0.1	1100	1.7	146-193	Orthic Gray Luvisol, Gleyed Gray Luvisol	Lodgepole pine, Subalpine fir, Interior spruce	Dfc, Boreal cool summer	OM1	C1	0	O horizon	66.0	27.6	0.8	4.7	1.8	34.9
TO081B	TO081	SBS_BC_	British Columbia	TO	Forest Soil	Whole Community DNA	PCR	Amplicon	pyrotag library	454 GS FLX Titanium	European Nucleotide Archive	https://www.ebi.ac.uk/ena/data/search?query=PRJEB12501	ERS1040518	SAMEA3733369	ERX1298855	ERR1226643	16S rRNA	V1-V3	NA	AGAGTTTGATCMTGGCTCAG	GWATTACCGCGGCKGCTG	2008-07-11	52.32	-126.31	Canada	0.1	1100	1.7	146-193	Orthic Gray Luvisol, Gleyed Gray Luvisol	Lodgepole pine, Subalpine fir, Interior spruce	Dfc, Boreal cool summer	OM1	C1	0	O horizon	69.0	27.6	0.8	4.7	1.8	34.9
TO082B	TO082	SBS_BC_	British Columbia	TO	Forest Soil	Whole Community DNA	PCR	Amplicon	pyrotag library	454 GS FLX Titanium	European Nucleotide Archive	https://www.ebi.ac.uk/ena/data/search?query=PRJEB12501	ERS1040519	SAMEA3733370	ERX1298856	ERR1226644	16S rRNA	V1-V3	NA	AGAGTTTGATCMTGGCTCAG	GWATTACCGCGGCKGCTG	2008-07-11	52.32	-126.31	Canada	0.3	1100	1.7	146-193	Orthic Gray Luvisol, Gleyed Gray Luvisol	Lodgepole pine, Subalpine fir, Interior spruce	Dfc, Boreal cool summer	OM1	C1	0	A horizon	30.0	1.9	0.1	4.9	1.8	21.1
TO083B	TO083	SBS_BC_	British Columbia	TO	Forest Soil	Whole Community DNA	PCR	Amplicon	pyrotag library	454 GS FLX Titanium	European Nucleotide Archive	https://www.ebi.ac.uk/ena/data/search?query=PRJEB12501	ERS1040520	SAMEA3733371	ERX1298857	ERR1226645	16S rRNA	V1-V3	NA	AGAGTTTGATCMTGGCTCAG	GWATTACCGCGGCKGCTG	2008-07-11	52.32	-126.31	Canada	0.3	1100	1.7	146-193	Orthic Gray Luvisol, Gleyed Gray Luvisol	Lodgepole pine, Subalpine fir, Interior spruce	Dfc, Boreal cool summer	OM1	C1	0	A horizon	25.0	1.9	0.1	4.9	1.8	21.1
TO084B	TO084	SBS_BC_	British Columbia	TO	Forest Soil	Whole Community DNA	PCR	Amplicon	pyrotag library	454 GS FLX Titanium	European Nucleotide Archive	https://www.ebi.ac.uk/ena/data/search?query=PRJEB12501	ERS1040521	SAMEA3733372	ERX1298858	ERR1226646	16S rRNA	V1-V3	NA	AGAGTTTGATCMTGGCTCAG	GWATTACCGCGGCKGCTG	2008-07-11	52.32	-126.31	Canada	0.3	1100	1.7	146-193	Orthic Gray Luvisol, Gleyed Gray Luvisol	Lodgepole pine, Subalpine fir, Interior spruce	Dfc, Boreal cool summer	OM1	C1	0	A horizon	25.0	1.9	0.1	4.9	1.8	21.1
TO085B	TO085	SBS_BC_	British Columbia	TO	Forest Soil	Whole Community DNA	PCR	Amplicon	pyrotag library	454 GS FLX Titanium	European Nucleotide Archive	https://www.ebi.ac.uk/ena/data/search?query=PRJEB12501	ERS1040522	SAMEA3733373	ERX1298859	ERR1226647	16S rRNA	V1-V3	NA	AGAGTTTGATCMTGGCTCAG	GWATTACCGCGGCKGCTG	2008-07-11	52.32	-126.31	Canada	0.1	1100	1.7	146-193	Orthic Gray Luvisol, Gleyed Gray Luvisol	Lodgepole pine, Subalpine fir, Interior spruce	Dfc, Boreal cool summer	OM2	C1	0	O horizon	68.0	37.1	1.2	4.9	1.7	32.0
TO086B	TO086	SBS_BC_	British Columbia	TO	Forest Soil	Whole Community DNA	PCR	Amplicon	pyrotag library	454 GS FLX Titanium	European Nucleotide Archive	https://www.ebi.ac.uk/ena/data/search?query=PRJEB12501	ERS1040523	SAMEA3733374	ERX1298860	ERR1226648	16S rRNA	V1-V3	NA	AGAGTTTGATCMTGGCTCAG	GWATTACCGCGGCKGCTG	2008-07-11	52.32	-126.31	Canada	0.1	1100	1.7	146-193	Orthic Gray Luvisol, Gleyed Gray Luvisol	Lodgepole pine, Subalpine fir, Interior spruce	Dfc, Boreal cool summer	OM2	C1	0	O horizon	68.0	37.1	1.2	4.9	1.7	32.0
TO087B	TO087	SBS_BC_	British Columbia	TO	Forest Soil	Whole Community DNA	PCR	Amplicon	pyrotag library	454 GS FLX Titanium	European Nucleotide Archive	https://www.ebi.ac.uk/ena/data/search?query=PRJEB12501	ERS1040524	SAMEA3733375	ERX1298861	ERR1226649	16S rRNA	V1-V3	NA	AGAGTTTGATCMTGGCTCAG	GWATTACCGCGGCKGCTG	2008-07-11	52.32	-126.31	Canada	0.1	1100	1.7	146-193	Orthic Gray Luvisol, Gleyed Gray Luvisol	Lodgepole pine, Subalpine fir, Interior spruce	Dfc, Boreal cool summer	OM2	C1	0	O horizon	56.0	37.1	1.2	4.9	1.7	32.0
TO088B	TO088	SBS_BC_	British Columbia	TO	Forest Soil	Whole Community DNA	PCR	Amplicon	pyrotag library	454 GS FLX Titanium	European Nucleotide Archive	https://www.ebi.ac.uk/ena/data/search?query=PRJEB12501	ERS1040525	SAMEA3733376	ERX1298862	ERR1226650	16S rRNA	V1-V3	NA	AGAGTTTGATCMTGGCTCAG	GWATTACCGCGGCKGCTG	2008-07-11	52.32	-126.31	Canada	0.3	1100	1.7	146-193	Orthic Gray Luvisol, Gleyed Gray Luvisol	Lodgepole pine, Subalpine fir, Interior spruce	Dfc, Boreal cool summer	OM2	C1	0	A horizon	18.0	1.9	0.1	4.9	1.7	21.6
TO089B	TO089	SBS_BC_	British Columbia	TO	Forest Soil	Whole Community DNA	PCR	Amplicon	pyrotag library	454 GS FLX Titanium	European Nucleotide Archive	https://www.ebi.ac.uk/ena/data/search?query=PRJEB12501	ERS1040526	SAMEA3733377	ERX1298863	ERR1226651	16S rRNA	V1-V3	NA	AGAGTTTGATCMTGGCTCAG	GWATTACCGCGGCKGCTG	2008-07-11	52.32	-126.31	Canada	0.3	1100	1.7	146-193	Orthic Gray Luvisol, Gleyed Gray Luvisol	Lodgepole pine, Subalpine fir, Interior spruce	Dfc, Boreal cool summer	OM2	C1	0	A horizon	22.0	1.9	0.1	4.9	1.7	21.6
TO090B	TO090	SBS_BC_	British Columbia	TO	Forest Soil	Whole Community DNA	PCR	Amplicon	pyrotag library	454 GS FLX Titanium	European Nucleotide Archive	https://www.ebi.ac.uk/ena/data/search?query=PRJEB12501	ERS1040527	SAMEA3733378	ERX1298864	ERR1226652	16S rRNA	V1-V3	NA	AGAGTTTGATCMTGGCTCAG	GWATTACCGCGGCKGCTG	2008-07-11	52.32	-126.31	Canada	0.3	1100	1.7	146-193	Orthic Gray Luvisol, Gleyed Gray Luvisol	Lodgepole pine, Subalpine fir, Interior spruce	Dfc, Boreal cool summer	OM2	C1	0	A horizon	20.0	1.9	0.1	4.9	1.7	21.6
TO094B	TO094	SBS_BC_	British Columbia	TO	Forest Soil	Whole Community DNA	PCR	Amplicon	pyrotag library	454 GS FLX Titanium	European Nucleotide Archive	https://www.ebi.ac.uk/ena/data/search?query=PRJEB12501	ERS1040528	SAMEA3733379	ERX1298865	ERR1226653	16S rRNA	V1-V3	NA	AGAGTTTGATCMTGGCTCAG	GWATTACCGCGGCKGCTG	2008-07-11	52.32	-126.31	Canada	0.3	1100	1.7	146-193	Orthic Gray Luvisol, Gleyed Gray Luvisol	Lodgepole pine, Subalpine fir, Interior spruce	Dfc, Boreal cool summer	OM3	C1	0	A horizon	16.0	1.7	0.1	5.5	1.7	21.0
TO095B	TO095	SBS_BC_	British Columbia	TO	Forest Soil	Whole Community DNA	PCR	Amplicon	pyrotag library	454 GS FLX Titanium	European Nucleotide Archive	https://www.ebi.ac.uk/ena/data/search?query=PRJEB12501	ERS1040529	SAMEA3733380	ERX1298866	ERR1226654	16S rRNA	V1-V3	NA	AGAGTTTGATCMTGGCTCAG	GWATTACCGCGGCKGCTG	2008-07-11	52.32	-126.31	Canada	0.3	1100	1.7	146-193	Orthic Gray Luvisol, Gleyed Gray Luvisol	Lodgepole pine, Subalpine fir, Interior spruce	Dfc, Boreal cool summer	OM3	C1	0	A horizon	17.0	1.7	0.1	5.5	1.7	21.0
TO096B	TO096	SBS_BC_	British Columbia	TO	Forest Soil	Whole Community DNA	PCR	Amplicon	pyrotag library	454 GS FLX Titanium	European Nucleotide Archive	https://www.ebi.ac.uk/ena/data/search?query=PRJEB12501	ERS1040530	SAMEA3733381	ERX1298867	ERR1226655	16S rRNA	V1-V3	NA	AGAGTTTGATCMTGGCTCAG	GWATTACCGCGGCKGCTG	2008-07-11	52.32	-126.31	Canada	0.3	1100	1.7	146-193	Orthic Gray Luvisol, Gleyed Gray Luvisol	Lodgepole pine, Subalpine fir, Interior spruce	Dfc, Boreal cool summer	OM3	C1	0	A horizon	15.0	1.7	0.1	5.5	1.7	21.0
TO097B	TO097	SBS_BC_	British Columbia	TO	Forest Soil	Whole Community DNA	PCR	Amplicon	pyrotag library	454 GS FLX Titanium	European Nucleotide Archive	https://www.ebi.ac.uk/ena/data/search?query=PRJEB12501	ERS1040531	SAMEA3733382	ERX1298868	ERR1226656	16S rRNA	V1-V3	NA	AGAGTTTGATCMTGGCTCAG	GWATTACCGCGGCKGCTG	2008-07-11	52.32	-126.31	Canada	0.1	1100	1.7	146-193	Orthic Gray Luvisol, Gleyed Gray Luvisol	Lodgepole pine, Subalpine fir, Interior spruce	Dfc, Boreal cool summer	OM1	C2	0	O horizon	60.0	35.6	0.9	5.1	1.5	37.9
TO098B	TO098	SBS_BC_	British Columbia	TO	Forest Soil	Whole Community DNA	PCR	Amplicon	pyrotag library	454 GS FLX Titanium	European Nucleotide Archive	https://www.ebi.ac.uk/ena/data/search?query=PRJEB12501	ERS1040532	SAMEA3733383	ERX1298869	ERR1226657	16S rRNA	V1-V3	NA	AGAGTTTGATCMTGGCTCAG	GWATTACCGCGGCKGCTG	2008-07-11	52.32	-126.31	Canada	0.1	1100	1.7	146-193	Orthic Gray Luvisol, Gleyed Gray Luvisol	Lodgepole pine, Subalpine fir, Interior spruce	Dfc, Boreal cool summer	OM1	C2	0	O horizon	64.0	35.6	0.9	5.1	1.5	37.9
TO099B	TO099	SBS_BC_	British Columbia	TO	Forest Soil	Whole Community DNA	PCR	Amplicon	pyrotag library	454 GS FLX Titanium	European Nucleotide Archive	https://www.ebi.ac.uk/ena/data/search?query=PRJEB12501	ERS1040533	SAMEA3733384	ERX1298870	ERR1226658	16S rRNA	V1-V3	NA	AGAGTTTGATCMTGGCTCAG	GWATTACCGCGGCKGCTG	2008-07-11	52.32	-126.31	Canada	0.1	1100	1.7	146-193	Orthic Gray Luvisol, Gleyed Gray Luvisol	Lodgepole pine, Subalpine fir, Interior spruce	Dfc, Boreal cool summer	OM1	C2	0	O horizon	66.0	35.6	0.9	5.1	1.5	37.9
TO100B	TO100	SBS_BC_	British Columbia	TO	Forest Soil	Whole Community DNA	PCR	Amplicon	pyrotag library	454 GS FLX Titanium	European Nucleotide Archive	https://www.ebi.ac.uk/ena/data/search?query=PRJEB12501	ERS1040534	SAMEA3733385	ERX1298871	ERR1226659	16S rRNA	V1-V3	NA	AGAGTTTGATCMTGGCTCAG	GWATTACCGCGGCKGCTG	2008-07-11	52.32	-126.31	Canada	0.3	1100	1.7	146-193	Orthic Gray Luvisol, Gleyed Gray Luvisol	Lodgepole pine, Subalpine fir, Interior spruce	Dfc, Boreal cool summer	OM1	C2	0	A horizon	21.0	2.3	0.1	5.2	1.5	20.7
TO101B	TO101	SBS_BC_	British Columbia	TO	Forest Soil	Whole Community DNA	PCR	Amplicon	pyrotag library	454 GS FLX Titanium	European Nucleotide Archive	https://www.ebi.ac.uk/ena/data/search?query=PRJEB12501	ERS1040535	SAMEA3733386	ERX1298872	ERR1226660	16S rRNA	V1-V3	NA	AGAGTTTGATCMTGGCTCAG	GWATTACCGCGGCKGCTG	2008-07-11	52.32	-126.31	Canada	0.3	1100	1.7	146-193	Orthic Gray Luvisol, Gleyed Gray Luvisol	Lodgepole pine, Subalpine fir, Interior spruce	Dfc, Boreal cool summer	OM1	C2	0	A horizon	22.0	2.3	0.1	5.2	1.5	20.7
TO102B	TO102	SBS_BC_	British Columbia	TO	Forest Soil	Whole Community DNA	PCR	Amplicon	pyrotag library	454 GS FLX Titanium	European Nucleotide Archive	https://www.ebi.ac.uk/ena/data/search?query=PRJEB12501	ERS1040536	SAMEA3733387	ERX1298873	ERR1226661	16S rRNA	V1-V3	NA	AGAGTTTGATCMTGGCTCAG	GWATTACCGCGGCKGCTG	2008-07-11	52.32	-126.31	Canada	0.3	1100	1.7	146-193	Orthic Gray Luvisol, Gleyed Gray Luvisol	Lodgepole pine, Subalpine fir, Interior spruce	Dfc, Boreal cool summer	OM1	C2	0	A horizon	17.0	2.3	0.1	5.2	1.5	20.7
TO103B	TO103	SBS_BC_	British Columbia	TO	Forest Soil	Whole Community DNA	PCR	Amplicon	pyrotag library	454 GS FLX Titanium	European Nucleotide Archive	https://www.ebi.ac.uk/ena/data/search?query=PRJEB12501	ERS1040537	SAMEA3733388	ERX1298874	ERR1226662	16S rRNA	V1-V3	NA	AGAGTTTGATCMTGGCTCAG	GWATTACCGCGGCKGCTG	2008-07-11	52.32	-126.31	Canada	0.1	1100	1.7	146-193	Orthic Gray Luvisol, Gleyed Gray Luvisol	Lodgepole pine, Subalpine fir, Interior spruce	Dfc, Boreal cool summer	OM2	C2	0	O horizon	86.0	30.6	0.9	5.1	1.8	34.8
TO104B	TO104	SBS_BC_	British Columbia	TO	Forest Soil	Whole Community DNA	PCR	Amplicon	pyrotag library	454 GS FLX Titanium	European Nucleotide Archive	https://www.ebi.ac.uk/ena/data/search?query=PRJEB12501	ERS1040538	SAMEA3733389	ERX1298875	ERR1226663	16S rRNA	V1-V3	NA	AGAGTTTGATCMTGGCTCAG	GWATTACCGCGGCKGCTG	2008-07-11	52.32	-126.31	Canada	0.1	1100	1.7	146-193	Orthic Gray Luvisol, Gleyed Gray Luvisol	Lodgepole pine, Subalpine fir, Interior spruce	Dfc, Boreal cool summer	OM2	C2	0	O horizon	74.0	30.6	0.9	5.1	1.8	34.8
TO105B	TO105	SBS_BC_	British Columbia	TO	Forest Soil	Whole Community DNA	PCR	Amplicon	pyrotag library	454 GS FLX Titanium	European Nucleotide Archive	https://www.ebi.ac.uk/ena/data/search?query=PRJEB12501	ERS1040539	SAMEA3733390	ERX1298876	ERR1226664	16S rRNA	V1-V3	NA	AGAGTTTGATCMTGGCTCAG	GWATTACCGCGGCKGCTG	2008-07-11	52.32	-126.31	Canada	0.1	1100	1.7	146-193	Orthic Gray Luvisol, Gleyed Gray Luvisol	Lodgepole pine, Subalpine fir, Interior spruce	Dfc, Boreal cool summer	OM2	C2	0	O horizon	64.0	30.6	0.9	5.1	1.8	34.8
TO106B	TO106	SBS_BC_	British Columbia	TO	Forest Soil	Whole Community DNA	PCR	Amplicon	pyrotag library	454 GS FLX Titanium	European Nucleotide Archive	https://www.ebi.ac.uk/ena/data/search?query=PRJEB12501	ERS1040540	SAMEA3733391	ERX1298877	ERR1226665	16S rRNA	V1-V3	NA	AGAGTTTGATCMTGGCTCAG	GWATTACCGCGGCKGCTG	2008-07-11	52.32	-126.31	Canada	0.3	1100	1.7	146-193	Orthic Gray Luvisol, Gleyed Gray Luvisol	Lodgepole pine, Subalpine fir, Interior spruce	Dfc, Boreal cool summer	OM2	C2	0	A horizon	18.0	3.1	0.2	5.1	1.8	19.6
TO107B	TO107	SBS_BC_	British Columbia	TO	Forest Soil	Whole Community DNA	PCR	Amplicon	pyrotag library	454 GS FLX Titanium	European Nucleotide Archive	https://www.ebi.ac.uk/ena/data/search?query=PRJEB12501	ERS1040541	SAMEA3733392	ERX1298878	ERR1226666	16S rRNA	V1-V3	NA	AGAGTTTGATCMTGGCTCAG	GWATTACCGCGGCKGCTG	2008-07-11	52.32	-126.31	Canada	0.3	1100	1.7	146-193	Orthic Gray Luvisol, Gleyed Gray Luvisol	Lodgepole pine, Subalpine fir, Interior spruce	Dfc, Boreal cool summer	OM2	C2	0	A horizon	27.0	3.1	0.2	5.1	1.8	19.6
TO108B	TO108	SBS_BC_	British Columbia	TO	Forest Soil	Whole Community DNA	PCR	Amplicon	pyrotag library	454 GS FLX Titanium	European Nucleotide Archive	https://www.ebi.ac.uk/ena/data/search?query=PRJEB12501	ERS1040542	SAMEA3733393	ERX1298879	ERR1226667	16S rRNA	V1-V3	NA	AGAGTTTGATCMTGGCTCAG	GWATTACCGCGGCKGCTG	2008-07-11	52.32	-126.31	Canada	0.3	1100	1.7	146-193	Orthic Gray Luvisol, Gleyed Gray Luvisol	Lodgepole pine, Subalpine fir, Interior spruce	Dfc, Boreal cool summer	OM2	C2	0	A horizon	18.0	3.1	0.2	5.1	1.8	19.6
TO112B	TO112	SBS_BC_	British Columbia	TO	Forest Soil	Whole Community DNA	PCR	Amplicon	pyrotag library	454 GS FLX Titanium	European Nucleotide Archive	https://www.ebi.ac.uk/ena/data/search?query=PRJEB12501	ERS1040543	SAMEA3733394	ERX1298880	ERR1226668	16S rRNA	V1-V3	NA	AGAGTTTGATCMTGGCTCAG	GWATTACCGCGGCKGCTG	2008-07-11	52.32	-126.31	Canada	0.3	1100	1.7	146-193	Orthic Gray Luvisol, Gleyed Gray Luvisol	Lodgepole pine, Subalpine fir, Interior spruce	Dfc, Boreal cool summer	OM3	C2	0	A horizon	20.0	1.8	0.1	5.7	1.6	19.5
TO113B	TO113	SBS_BC_	British Columbia	TO	Forest Soil	Whole Community DNA	PCR	Amplicon	pyrotag library	454 GS FLX Titanium	European Nucleotide Archive	https://www.ebi.ac.uk/ena/data/search?query=PRJEB12501	ERS1040544	SAMEA3733395	ERX1298881	ERR1226669	16S rRNA	V1-V3	NA	AGAGTTTGATCMTGGCTCAG	GWATTACCGCGGCKGCTG	2008-07-11	52.32	-126.31	Canada	0.3	1100	1.7	146-193	Orthic Gray Luvisol, Gleyed Gray Luvisol	Lodgepole pine, Subalpine fir, Interior spruce	Dfc, Boreal cool summer	OM3	C2	0	A horizon	16.0	1.8	0.1	5.7	1.6	19.5
TO114B	TO114	SBS_BC_	British Columbia	TO	Forest Soil	Whole Community DNA	PCR	Amplicon	pyrotag library	454 GS FLX Titanium	European Nucleotide Archive	https://www.ebi.ac.uk/ena/data/search?query=PRJEB12501	ERS1040545	SAMEA3733396	ERX1298882	ERR1226670	16S rRNA	V1-V3	NA	AGAGTTTGATCMTGGCTCAG	GWATTACCGCGGCKGCTG	2008-07-11	52.32	-126.31	Canada	0.3	1100	1.7	146-193	Orthic Gray Luvisol, Gleyed Gray Luvisol	Lodgepole pine, Subalpine fir, Interior spruce	Dfc, Boreal cool summer	OM3	C2	0	A horizon	18.0	1.8	0.1	5.7	1.6	19.5
TO115B	TO115	SBS_BC_	British Columbia	TO	Forest Soil	Whole Community DNA	PCR	Amplicon	pyrotag library	454 GS FLX Titanium	European Nucleotide Archive	https://www.ebi.ac.uk/ena/data/search?query=PRJEB12501	ERS1040546	SAMEA3733397	ERX1298883	ERR1226671	16S rRNA	V1-V3	NA	AGAGTTTGATCMTGGCTCAG	GWATTACCGCGGCKGCTG	2008-07-11	52.32	-126.31	Canada	0.1	1100	1.7	146-193	Orthic Gray Luvisol, Gleyed Gray Luvisol	Lodgepole pine, Subalpine fir, Interior spruce	Dfc, Boreal cool summer	REF	REF	0	O horizon	79.0	44.8	1.1	4.4	NA	42.3
TO116B	TO116	SBS_BC_	British Columbia	TO	Forest Soil	Whole Community DNA	PCR	Amplicon	pyrotag library	454 GS FLX Titanium	European Nucleotide Archive	https://www.ebi.ac.uk/ena/data/search?query=PRJEB12501	ERS1040547	SAMEA3733398	ERX1298884	ERR1226672	16S rRNA	V1-V3	NA	AGAGTTTGATCMTGGCTCAG	GWATTACCGCGGCKGCTG	2008-07-11	52.32	-126.31	Canada	0.1	1100	1.7	146-193	Orthic Gray Luvisol, Gleyed Gray Luvisol	Lodgepole pine, Subalpine fir, Interior spruce	Dfc, Boreal cool summer	REF	REF	0	O horizon	76.0	44.8	1.1	4.4	NA	42.3
TO117B	TO117	SBS_BC_	British Columbia	TO	Forest Soil	Whole Community DNA	PCR	Amplicon	pyrotag library	454 GS FLX Titanium	European Nucleotide Archive	https://www.ebi.ac.uk/ena/data/search?query=PRJEB12501	ERS1040548	SAMEA3733399	ERX1298885	ERR1226673	16S rRNA	V1-V3	NA	AGAGTTTGATCMTGGCTCAG	GWATTACCGCGGCKGCTG	2008-07-11	52.32	-126.31	Canada	0.1	1100	1.7	146-193	Orthic Gray Luvisol, Gleyed Gray Luvisol	Lodgepole pine, Subalpine fir, Interior spruce	Dfc, Boreal cool summer	REF	REF	0	O horizon	72.0	44.8	1.1	4.4	NA	42.3
TO118B	TO118	SBS_BC_	British Columbia	TO	Forest Soil	Whole Community DNA	PCR	Amplicon	pyrotag library	454 GS FLX Titanium	European Nucleotide Archive	https://www.ebi.ac.uk/ena/data/search?query=PRJEB12501	ERS1040549	SAMEA3733400	ERX1298886	ERR1226674	16S rRNA	V1-V3	NA	AGAGTTTGATCMTGGCTCAG	GWATTACCGCGGCKGCTG	2008-07-11	52.32	-126.31	Canada	0.3	1100	1.7	146-193	Orthic Gray Luvisol, Gleyed Gray Luvisol	Lodgepole pine, Subalpine fir, Interior spruce	Dfc, Boreal cool summer	REF	REF	0	A horizon	20.0	1.1	0.1	5.0	NA	22.2
TO119B	TO119	SBS_BC_	British Columbia	TO	Forest Soil	Whole Community DNA	PCR	Amplicon	pyrotag library	454 GS FLX Titanium	European Nucleotide Archive	https://www.ebi.ac.uk/ena/data/search?query=PRJEB12501	ERS1040550	SAMEA3733401	ERX1298887	ERR1226675	16S rRNA	V1-V3	NA	AGAGTTTGATCMTGGCTCAG	GWATTACCGCGGCKGCTG	2008-07-11	52.32	-126.31	Canada	0.3	1100	1.7	146-193	Orthic Gray Luvisol, Gleyed Gray Luvisol	Lodgepole pine, Subalpine fir, Interior spruce	Dfc, Boreal cool summer	REF	REF	0	A horizon	19.0	1.1	0.1	5.0	NA	22.2
TO120B	TO120	SBS_BC_	British Columbia	TO	Forest Soil	Whole Community DNA	PCR	Amplicon	pyrotag library	454 GS FLX Titanium	European Nucleotide Archive	https://www.ebi.ac.uk/ena/data/search?query=PRJEB12501	ERS1040551	SAMEA3733402	ERX1298888	ERR1226676	16S rRNA	V1-V3	NA	AGAGTTTGATCMTGGCTCAG	GWATTACCGCGGCKGCTG	2008-07-11	52.32	-126.31	Canada	0.3	1100	1.7	146-193	Orthic Gray Luvisol, Gleyed Gray Luvisol	Lodgepole pine, Subalpine fir, Interior spruce	Dfc, Boreal cool summer	REF	REF	0	A horizon	25.0	1.1	0.1	5.0	NA	22.2
A7001B	A7001	BS_ON_	Ontario	A7	Forest Soil	Whole Community DNA	PCR	Amplicon	pyrotag library	454 GS FLX Titanium	European Nucleotide Archive	https://www.ebi.ac.uk/ena/data/search?query=PRJEB8599	ERS662612	SAMEA3261616	ERX708859	ERR765586	16S rRNA	V1-V3	ACGAGTGCGT	AGAGTTTGATCMTGGCTCAG	GWATTACCGCGGCKGCTG	2011-07-03	49.07	-89.41	Canada	0.1	445	2.4	266	Orthic Dystric Brunisol	Black Spruce	Dfb, Humid Continental warm summer	OM2	C0	0	O horizon	48.1	42.5	1.1	5.4	0.2	37.9
A7002B	A7002	BS_ON_	Ontario	A7	Forest Soil	Whole Community DNA	PCR	Amplicon	pyrotag library	454 GS FLX Titanium	European Nucleotide Archive	https://www.ebi.ac.uk/ena/data/search?query=PRJEB8599	ERS662613	SAMEA3261617	ERX708860	ERR765587	16S rRNA	V1-V3	TCACGTACTA	AGAGTTTGATCMTGGCTCAG	GWATTACCGCGGCKGCTG	2011-07-03	49.07	-89.41	Canada	0.3	445	2.4	266	Orthic Dystric Brunisol	Black Spruce	Dfb, Humid Continental warm summer	OM2	C0	0	A horizon	18.4	0.6	0.0	5.7	1.3	16.3
A7003B	A7003	BS_ON_	Ontario	A7	Forest Soil	Whole Community DNA	PCR	Amplicon	pyrotag library	454 GS FLX Titanium	European Nucleotide Archive	https://www.ebi.ac.uk/ena/data/search?query=PRJEB8599	ERS662614	SAMEA3261618	ERX708861	ERR765588	16S rRNA	V1-V3	TGACGTATGT	AGAGTTTGATCMTGGCTCAG	GWATTACCGCGGCKGCTG	2011-07-03	49.07	-89.41	Canada	0.1	445	2.4	266	Orthic Dystric Brunisol	Black Spruce	Dfb, Humid Continental warm summer	OM3	C0	0	O horizon	60.8	31.2	0.8	5.5	0.2	37.5
A7004B	A7004	BS_ON_	Ontario	A7	Forest Soil	Whole Community DNA	PCR	Amplicon	pyrotag library	454 GS FLX Titanium	European Nucleotide Archive	https://www.ebi.ac.uk/ena/data/search?query=PRJEB8599	ERS662615	SAMEA3261619	ERX708862	ERR765589	16S rRNA	V1-V3	TAGTGTAGAT	AGAGTTTGATCMTGGCTCAG	GWATTACCGCGGCKGCTG	2011-07-03	49.07	-89.41	Canada	0.3	445	2.4	266	Orthic Dystric Brunisol	Black Spruce	Dfb, Humid Continental warm summer	OM3	C0	0	A horizon	16.4	1.2	0.1	6.0	1.0	20.1
A7008B	A7008	BS_ON_	Ontario	A7	Forest Soil	Whole Community DNA	PCR	Amplicon	pyrotag library	454 GS FLX Titanium	European Nucleotide Archive	https://www.ebi.ac.uk/ena/data/search?query=PRJEB8599	ERS662616	SAMEA3261620	ERX708863	ERR765590	16S rRNA	V1-V3	TGTGAGTAGT	AGAGTTTGATCMTGGCTCAG	GWATTACCGCGGCKGCTG	2011-07-03	49.07	-89.41	Canada	0.3	445	2.4	266	Orthic Dystric Brunisol	Black Spruce	Dfb, Humid Continental warm summer	OM1	C0	0	A horizon	16.4	2.4	0.1	5.5	1.2	23.9
A7009B	A7009	BS_ON_	Ontario	A7	Forest Soil	Whole Community DNA	PCR	Amplicon	pyrotag library	454 GS FLX Titanium	European Nucleotide Archive	https://www.ebi.ac.uk/ena/data/search?query=PRJEB8599	ERS662617	SAMEA3261621	ERX708864	ERR765591	16S rRNA	V1-V3	AGACTATACT	AGAGTTTGATCMTGGCTCAG	GWATTACCGCGGCKGCTG	2011-07-03	49.07	-89.41	Canada	0.1	445	2.4	266	Orthic Dystric Brunisol	Black Spruce	Dfb, Humid Continental warm summer	OM2	C0	0	O horizon	29.7	34.9	0.9	4.4	0.2	39.8
A7010B	A7010	BS_ON_	Ontario	A7	Forest Soil	Whole Community DNA	PCR	Amplicon	pyrotag library	454 GS FLX Titanium	European Nucleotide Archive	https://www.ebi.ac.uk/ena/data/search?query=PRJEB8599	ERS662618	SAMEA3261622	ERX708865	ERR765592	16S rRNA	V1-V3	TGTGAGTAGT	AGAGTTTGATCMTGGCTCAG	GWATTACCGCGGCKGCTG	2011-07-03	49.07	-89.41	Canada	0.3	445	2.4	266	Orthic Dystric Brunisol	Black Spruce	Dfb, Humid Continental warm summer	OM2	C0	0	A horizon	13.1	1.1	0.1	5.6	1.0	19.8
A7011B	A7011	BS_ON_	Ontario	A7	Forest Soil	Whole Community DNA	PCR	Amplicon	pyrotag library	454 GS FLX Titanium	European Nucleotide Archive	https://www.ebi.ac.uk/ena/data/search?query=PRJEB8599	ERS662619	SAMEA3261623	ERX708866	ERR765593	16S rRNA	V1-V3	TGATACGTCT	AGAGTTTGATCMTGGCTCAG	GWATTACCGCGGCKGCTG	2011-07-03	49.07	-89.41	Canada	0.1	445	2.4	266	Orthic Dystric Brunisol	Black Spruce	Dfb, Humid Continental warm summer	OM3	C0	0	O horizon	33.6	33.8	0.9	5.1	0.2	38.1
A7012B	A7012	BS_ON_	Ontario	A7	Forest Soil	Whole Community DNA	PCR	Amplicon	pyrotag library	454 GS FLX Titanium	European Nucleotide Archive	https://www.ebi.ac.uk/ena/data/search?query=PRJEB8599	ERS662620	SAMEA3261624	ERX708867	ERR765594	16S rRNA	V1-V3	TCTCTATGCG	AGAGTTTGATCMTGGCTCAG	GWATTACCGCGGCKGCTG	2011-07-03	49.07	-89.41	Canada	0.3	445	2.4	266	Orthic Dystric Brunisol	Black Spruce	Dfb, Humid Continental warm summer	OM3	C0	0	A horizon	18.9	1.4	0.1	5.7	1.0	21.5
A7015B	A7015	BS_ON_	Ontario	A7	Forest Soil	Whole Community DNA	PCR	Amplicon	pyrotag library	454 GS FLX Titanium	European Nucleotide Archive	https://www.ebi.ac.uk/ena/data/search?query=PRJEB8599	ERS662621	SAMEA3261625	ERX708868	ERR765595	16S rRNA	V1-V3	ACTGTACAGT	AGAGTTTGATCMTGGCTCAG	GWATTACCGCGGCKGCTG	2011-07-03	49.07	-89.41	Canada	0.1	445	2.4	266	Orthic Dystric Brunisol	Black Spruce	Dfb, Humid Continental warm summer	OM1	C0	0	O horizon	37.0	37.3	1.0	5.1	0.2	39.3
A7016B	A7016	BS_ON_	Ontario	A7	Forest Soil	Whole Community DNA	PCR	Amplicon	pyrotag library	454 GS FLX Titanium	European Nucleotide Archive	https://www.ebi.ac.uk/ena/data/search?query=PRJEB8599	ERS662622	SAMEA3261626	ERX708869	ERR765596	16S rRNA	V1-V3	ATAGAGTACT	AGAGTTTGATCMTGGCTCAG	GWATTACCGCGGCKGCTG	2011-07-03	49.07	-89.41	Canada	0.3	445	2.4	266	Orthic Dystric Brunisol	Black Spruce	Dfb, Humid Continental warm summer	OM1	C0	0	A horizon	25.4	2.5	0.1	5.5	1.1	21.3
A7019B	A7019	BS_ON_	Ontario	A7	Forest Soil	Whole Community DNA	PCR	Amplicon	pyrotag library	454 GS FLX Titanium	European Nucleotide Archive	https://www.ebi.ac.uk/ena/data/search?query=PRJEB8599	ERS662623	SAMEA3261627	ERX708870	ERR765597	16S rRNA	V1-V3	CGAGAGATAC	AGAGTTTGATCMTGGCTCAG	GWATTACCGCGGCKGCTG	2011-07-03	49.07	-89.41	Canada	0.1	445	2.4	266	Orthic Dystric Brunisol	Black Spruce	Dfb, Humid Continental warm summer	OM2	C0	0	O horizon	50.0	46.9	1.2	4.3	0.2	40.1
A7020B	A7020	BS_ON_	Ontario	A7	Forest Soil	Whole Community DNA	PCR	Amplicon	pyrotag library	454 GS FLX Titanium	European Nucleotide Archive	https://www.ebi.ac.uk/ena/data/search?query=PRJEB8599	ERS662624	SAMEA3261628	ERX708871	ERR765598	16S rRNA	V1-V3	ACAGTATATA	AGAGTTTGATCMTGGCTCAG	GWATTACCGCGGCKGCTG	2011-07-03	49.07	-89.41	Canada	0.3	445	2.4	266	Orthic Dystric Brunisol	Black Spruce	Dfb, Humid Continental warm summer	OM2	C0	0	A horizon	20.4	2.0	0.1	5.5	1.3	21.6
A7021B	A7021	BS_ON_	Ontario	A7	Forest Soil	Whole Community DNA	PCR	Amplicon	pyrotag library	454 GS FLX Titanium	European Nucleotide Archive	https://www.ebi.ac.uk/ena/data/search?query=PRJEB8599	ERS662625	SAMEA3261629	ERX708872	ERR765599	16S rRNA	V1-V3	CAGTAGACGT	AGAGTTTGATCMTGGCTCAG	GWATTACCGCGGCKGCTG	2011-07-03	49.07	-89.41	Canada	0.1	445	2.4	266	Orthic Dystric Brunisol	Black Spruce	Dfb, Humid Continental warm summer	OM3	C0	0	O horizon	45.1	40.5	1.2	4.5	0.2	33.7
A7022B	A7022	BS_ON_	Ontario	A7	Forest Soil	Whole Community DNA	PCR	Amplicon	pyrotag library	454 GS FLX Titanium	European Nucleotide Archive	https://www.ebi.ac.uk/ena/data/search?query=PRJEB8599	ERS662626	SAMEA3261630	ERX708873	ERR765600	16S rRNA	V1-V3	TACACGTGAT	AGAGTTTGATCMTGGCTCAG	GWATTACCGCGGCKGCTG	2011-07-03	49.07	-89.41	Canada	0.3	445	2.4	266	Orthic Dystric Brunisol	Black Spruce	Dfb, Humid Continental warm summer	OM3	C0	0	A horizon	17.1	1.2	0.1	5.6	1.4	24.2
A7023B	A7023	BS_ON_	Ontario	A7	Forest Soil	Whole Community DNA	PCR	Amplicon	pyrotag library	454 GS FLX Titanium	European Nucleotide Archive	https://www.ebi.ac.uk/ena/data/search?query=PRJEB8599	ERS662627	SAMEA3261631	ERX708874	ERR765601	16S rRNA	V1-V3	TACAGATCGT	AGAGTTTGATCMTGGCTCAG	GWATTACCGCGGCKGCTG	2011-07-03	49.07	-89.41	Canada	0.1	445	2.4	266	Orthic Dystric Brunisol	Black Spruce	Dfb, Humid Continental warm summer	OM1	C0	0	O horizon	63.6	44.9	1.1	4.5	0.2	42.1
A7024B	A7024	BS_ON_	Ontario	A7	Forest Soil	Whole Community DNA	PCR	Amplicon	pyrotag library	454 GS FLX Titanium	European Nucleotide Archive	https://www.ebi.ac.uk/ena/data/search?query=PRJEB8599	ERS662628	SAMEA3261632	ERX708875	ERR765602	16S rRNA	V1-V3	AGCGTCGTCT	AGAGTTTGATCMTGGCTCAG	GWATTACCGCGGCKGCTG	2011-07-03	49.07	-89.41	Canada	0.3	445	2.4	266	Orthic Dystric Brunisol	Black Spruce	Dfb, Humid Continental warm summer	OM1	C0	0	A horizon	26.4	1.1	0.0	5.8	1.5	27.3
A7025B	A7025	BS_ON_	Ontario	A7	Forest Soil	Whole Community DNA	PCR	Amplicon	pyrotag library	454 GS FLX Titanium	European Nucleotide Archive	https://www.ebi.ac.uk/ena/data/search?query=PRJEB8599	ERS662629	SAMEA3261633	ERX708876	ERR765603	16S rRNA	V1-V3	ACGCGATCGA	AGAGTTTGATCMTGGCTCAG	GWATTACCGCGGCKGCTG	2011-07-03	49.07	-89.41	Canada	0.1	445	2.4	266	Orthic Dystric Brunisol	Black Spruce	Dfb, Humid Continental warm summer	REF	REF	0	O horizon	56.8	45.0	1.1	4.3	0.1	40.6
A7026B	A7026	BS_ON_	Ontario	A7	Forest Soil	Whole Community DNA	PCR	Amplicon	pyrotag library	454 GS FLX Titanium	European Nucleotide Archive	https://www.ebi.ac.uk/ena/data/search?query=PRJEB8599	ERS662630	SAMEA3261634	ERX708877	ERR765604	16S rRNA	V1-V3	AGCACTGTAG	AGAGTTTGATCMTGGCTCAG	GWATTACCGCGGCKGCTG	2011-07-03	49.07	-89.41	Canada	0.3	445	2.4	266	Orthic Dystric Brunisol	Black Spruce	Dfb, Humid Continental warm summer	REF	REF	0	A horizon	26.3	1.4	0.1	5.3	1.1	21.4
A7027B	A7027	BS_ON_	Ontario	A7	Forest Soil	Whole Community DNA	PCR	Amplicon	pyrotag library	454 GS FLX Titanium	European Nucleotide Archive	https://www.ebi.ac.uk/ena/data/search?query=PRJEB8599	ERS662631	SAMEA3261635	ERX708878	ERR765605	16S rRNA	V1-V3	CGTGTCTCTA	AGAGTTTGATCMTGGCTCAG	GWATTACCGCGGCKGCTG	2011-07-03	49.07	-89.41	Canada	0.1	445	2.4	266	Orthic Dystric Brunisol	Black Spruce	Dfb, Humid Continental warm summer	REF	REF	0	O horizon	57.6	44.7	1.0	4.4	0.1	44.4
A7028B	A7028	BS_ON_	Ontario	A7	Forest Soil	Whole Community DNA	PCR	Amplicon	pyrotag library	454 GS FLX Titanium	European Nucleotide Archive	https://www.ebi.ac.uk/ena/data/search?query=PRJEB8599	ERS662632	SAMEA3261636	ERX708879	ERR765606	16S rRNA	V1-V3	TCTCTATGCG	AGAGTTTGATCMTGGCTCAG	GWATTACCGCGGCKGCTG	2011-07-03	49.07	-89.41	Canada	0.3	445	2.4	266	Orthic Dystric Brunisol	Black Spruce	Dfb, Humid Continental warm summer	REF	REF	0	A horizon	25.4	2.1	0.1	5.3	0.9	24.3
A7029B	A7029	BS_ON_	Ontario	A7	Forest Soil	Whole Community DNA	PCR	Amplicon	pyrotag library	454 GS FLX Titanium	European Nucleotide Archive	https://www.ebi.ac.uk/ena/data/search?query=PRJEB8599	ERS662633	SAMEA3261637	ERX708880	ERR765607	16S rRNA	V1-V3	AGACTATACT	AGAGTTTGATCMTGGCTCAG	GWATTACCGCGGCKGCTG	2011-07-03	49.07	-89.41	Canada	0.1	445	2.4	266	Orthic Dystric Brunisol	Black Spruce	Dfb, Humid Continental warm summer	REF	REF	0	O horizon	58.1	40.7	1.0	4.2	0.2	40.2
A7030B	A7030	BS_ON_	Ontario	A7	Forest Soil	Whole Community DNA	PCR	Amplicon	pyrotag library	454 GS FLX Titanium	European Nucleotide Archive	https://www.ebi.ac.uk/ena/data/search?query=PRJEB8599	ERS662634	SAMEA3261638	ERX708881	ERR765608	16S rRNA	V1-V3	ATAGAGTACT	AGAGTTTGATCMTGGCTCAG	GWATTACCGCGGCKGCTG	2011-07-03	49.07	-89.41	Canada	0.3	445	2.4	266	Orthic Dystric Brunisol	Black Spruce	Dfb, Humid Continental warm summer	REF	REF	0	A horizon	16.0	2.4	0.1	5.2	0.9	20.3
A8031B	A8031	BS_ON_	Ontario	A8	Forest Soil	Whole Community DNA	PCR	Amplicon	pyrotag library	454 GS FLX Titanium	European Nucleotide Archive	https://www.ebi.ac.uk/ena/data/search?query=PRJEB8599	ERS662635	SAMEA3261639	ERX708882	ERR765609	16S rRNA	V1-V3	ACGAGTGCGT	AGAGTTTGATCMTGGCTCAG	GWATTACCGCGGCKGCTG	2011-07-04	49.08	-89.38	Canada	0.1	450	1.8	266	Orthic Dystric Brunisol	Black Spruce	Dfb, Humid Continental warm summer	OM1	C0	0	O horizon	53.8	41.4	1.2	4.6	0.2	35.4
A8032B	A8032	BS_ON_	Ontario	A8	Forest Soil	Whole Community DNA	PCR	Amplicon	pyrotag library	454 GS FLX Titanium	European Nucleotide Archive	https://www.ebi.ac.uk/ena/data/search?query=PRJEB8599	ERS662636	SAMEA3261640	ERX708883	ERR765610	16S rRNA	V1-V3	TGTGAGTAGT	AGAGTTTGATCMTGGCTCAG	GWATTACCGCGGCKGCTG	2011-07-04	49.08	-89.38	Canada	0.3	450	1.8	266	Orthic Dystric Brunisol	Black Spruce	Dfb, Humid Continental warm summer	OM1	C0	0	A horizon	22.8	4.5	0.2	4.7	1.0	24.5
A8035B	A8035	BS_ON_	Ontario	A8	Forest Soil	Whole Community DNA	PCR	Amplicon	pyrotag library	454 GS FLX Titanium	European Nucleotide Archive	https://www.ebi.ac.uk/ena/data/search?query=PRJEB8599	ERS662637	SAMEA3261641	ERX708884	ERR765611	16S rRNA	V1-V3	TCTATACTAT	AGAGTTTGATCMTGGCTCAG	GWATTACCGCGGCKGCTG	2011-07-04	49.08	-89.38	Canada	0.1	450	1.8	266	Orthic Dystric Brunisol	Black Spruce	Dfb, Humid Continental warm summer	OM3	C0	0	O horizon	50.6	32.3	0.8	5.1	0.2	38.4
A8036B	A8036	BS_ON_	Ontario	A8	Forest Soil	Whole Community DNA	PCR	Amplicon	pyrotag library	454 GS FLX Titanium	European Nucleotide Archive	https://www.ebi.ac.uk/ena/data/search?query=PRJEB8599	ERS662638	SAMEA3261642	ERX708885	ERR765612	16S rRNA	V1-V3	CACGCTACGT	AGAGTTTGATCMTGGCTCAG	GWATTACCGCGGCKGCTG	2011-07-04	49.08	-89.38	Canada	0.3	450	1.8	266	Orthic Dystric Brunisol	Black Spruce	Dfb, Humid Continental warm summer	OM3	C0	0	A horizon	13.5	0.8	0.0	5.6	1.7	21.6
A8038B	A8038	BS_ON_	Ontario	A8	Forest Soil	Whole Community DNA	PCR	Amplicon	pyrotag library	454 GS FLX Titanium	European Nucleotide Archive	https://www.ebi.ac.uk/ena/data/search?query=PRJEB8599	ERS662640	SAMEA3261644	ERX708887	ERR765614	16S rRNA	V1-V3	AGACTATACT	AGAGTTTGATCMTGGCTCAG	GWATTACCGCGGCKGCTG	2011-07-04	49.08	-89.38	Canada	0.3	450	1.8	266	Orthic Dystric Brunisol	Black Spruce	Dfb, Humid Continental warm summer	OM2	C0	0	A horizon	19.5	1.3	0.1	5.0	1.3	20.8
A8039B	A8039	BS_ON_	Ontario	A8	Forest Soil	Whole Community DNA	PCR	Amplicon	pyrotag library	454 GS FLX Titanium	European Nucleotide Archive	https://www.ebi.ac.uk/ena/data/search?query=PRJEB8599	ERS662641	SAMEA3261645	ERX708888	ERR765615	16S rRNA	V1-V3	ACAGTATATA	AGAGTTTGATCMTGGCTCAG	GWATTACCGCGGCKGCTG	2011-07-04	49.08	-89.38	Canada	0.1	450	1.8	266	Orthic Dystric Brunisol	Black Spruce	Dfb, Humid Continental warm summer	OM2	C0	0	O horizon	70.0	43.8	1.3	4.8	0.3	34.2
A8040B	A8040	BS_ON_	Ontario	A8	Forest Soil	Whole Community DNA	PCR	Amplicon	pyrotag library	454 GS FLX Titanium	European Nucleotide Archive	https://www.ebi.ac.uk/ena/data/search?query=PRJEB8599	ERS662642	SAMEA3261646	ERX708889	ERR765616	16S rRNA	V1-V3	ACTACTATGT	AGAGTTTGATCMTGGCTCAG	GWATTACCGCGGCKGCTG	2011-07-04	49.08	-89.38	Canada	0.3	450	1.8	266	Orthic Dystric Brunisol	Black Spruce	Dfb, Humid Continental warm summer	OM2	C0	0	A horizon	25.5	1.6	0.1	5.4	1.3	21.6
A8041B	A8041	BS_ON_	Ontario	A8	Forest Soil	Whole Community DNA	PCR	Amplicon	pyrotag library	454 GS FLX Titanium	European Nucleotide Archive	https://www.ebi.ac.uk/ena/data/search?query=PRJEB8599	ERS662643	SAMEA3261647	ERX708890	ERR765617	16S rRNA	V1-V3	ACGCTCGACA	AGAGTTTGATCMTGGCTCAG	GWATTACCGCGGCKGCTG	2011-07-04	49.08	-89.38	Canada	0.1	450	1.8	266	Orthic Dystric Brunisol	Black Spruce	Dfb, Humid Continental warm summer	OM3	C0	0	O horizon	43.2	42.0	1.2	5.0	0.1	35.4
A8042B	A8042	BS_ON_	Ontario	A8	Forest Soil	Whole Community DNA	PCR	Amplicon	pyrotag library	454 GS FLX Titanium	European Nucleotide Archive	https://www.ebi.ac.uk/ena/data/search?query=PRJEB8599	ERS662644	SAMEA3261648	ERX708891	ERR765618	16S rRNA	V1-V3	CGTCTAGTAC	AGAGTTTGATCMTGGCTCAG	GWATTACCGCGGCKGCTG	2011-07-04	49.08	-89.38	Canada	0.3	450	1.8	266	Orthic Dystric Brunisol	Black Spruce	Dfb, Humid Continental warm summer	OM3	C0	0	A horizon	18.1	2.1	0.1	5.2	1.6	20.9
A8045B	A8045	BS_ON_	Ontario	A8	Forest Soil	Whole Community DNA	PCR	Amplicon	pyrotag library	454 GS FLX Titanium	European Nucleotide Archive	https://www.ebi.ac.uk/ena/data/search?query=PRJEB8599	ERS662645	SAMEA3261649	ERX708892	ERR765619	16S rRNA	V1-V3	AGCACTGTAG	AGAGTTTGATCMTGGCTCAG	GWATTACCGCGGCKGCTG	2011-07-04	49.08	-89.38	Canada	0.1	450	1.8	266	Orthic Dystric Brunisol	Black Spruce	Dfb, Humid Continental warm summer	OM1	C0	0	O horizon	36.2	41.6	1.2	4.7	0.2	34.2
A8046B	A8046	BS_ON_	Ontario	A8	Forest Soil	Whole Community DNA	PCR	Amplicon	pyrotag library	454 GS FLX Titanium	European Nucleotide Archive	https://www.ebi.ac.uk/ena/data/search?query=PRJEB8599	ERS662646	SAMEA3261650	ERX708893	ERR765620	16S rRNA	V1-V3	TCTACGTAGC	AGAGTTTGATCMTGGCTCAG	GWATTACCGCGGCKGCTG	2011-07-04	49.08	-89.38	Canada	0.3	450	1.8	266	Orthic Dystric Brunisol	Black Spruce	Dfb, Humid Continental warm summer	OM1	C0	0	A horizon	24.0	2.1	0.1	5.3	1.1	21.7
A8047B	A8047	BS_ON_	Ontario	A8	Forest Soil	Whole Community DNA	PCR	Amplicon	pyrotag library	454 GS FLX Titanium	European Nucleotide Archive	https://www.ebi.ac.uk/ena/data/search?query=PRJEB8599	ERS662647	SAMEA3261651	ERX708894	ERR765621	16S rRNA	V1-V3	AGACGCACTC	AGAGTTTGATCMTGGCTCAG	GWATTACCGCGGCKGCTG	2011-07-04	49.08	-89.38	Canada	0.1	450	1.8	266	Orthic Dystric Brunisol	Black Spruce	Dfb, Humid Continental warm summer	OM2	C0	0	O horizon	52.5	41.7	1.2	4.5	0.3	35.5
A8048B	A8048	BS_ON_	Ontario	A8	Forest Soil	Whole Community DNA	PCR	Amplicon	pyrotag library	454 GS FLX Titanium	European Nucleotide Archive	https://www.ebi.ac.uk/ena/data/search?query=PRJEB8599	ERS662648	SAMEA3261652	ERX708895	ERR765622	16S rRNA	V1-V3	CGTGTCTCTA	AGAGTTTGATCMTGGCTCAG	GWATTACCGCGGCKGCTG	2011-07-04	49.08	-89.38	Canada	0.3	450	1.8	266	Orthic Dystric Brunisol	Black Spruce	Dfb, Humid Continental warm summer	OM2	C0	0	A horizon	21.1	0.9	0.0	5.4	1.7	23.6
A8049B	A8049	BS_ON_	Ontario	A8	Forest Soil	Whole Community DNA	PCR	Amplicon	pyrotag library	454 GS FLX Titanium	European Nucleotide Archive	https://www.ebi.ac.uk/ena/data/search?query=PRJEB8599	ERS662649	SAMEA3261653	ERX708896	ERR765623	16S rRNA	V1-V3	TCTAGCGACT	AGAGTTTGATCMTGGCTCAG	GWATTACCGCGGCKGCTG	2011-07-04	49.08	-89.38	Canada	0.1	450	1.8	266	Orthic Dystric Brunisol	Black Spruce	Dfb, Humid Continental warm summer	OM3	C0	0	O horizon	47.8	30.4	0.8	4.8	0.2	39.4
A8050B	A8050	BS_ON_	Ontario	A8	Forest Soil	Whole Community DNA	PCR	Amplicon	pyrotag library	454 GS FLX Titanium	European Nucleotide Archive	https://www.ebi.ac.uk/ena/data/search?query=PRJEB8599	ERS662650	SAMEA3261654	ERX708897	ERR765624	16S rRNA	V1-V3	TACACGTGAT	AGAGTTTGATCMTGGCTCAG	GWATTACCGCGGCKGCTG	2011-07-04	49.08	-89.38	Canada	0.3	450	1.8	266	Orthic Dystric Brunisol	Black Spruce	Dfb, Humid Continental warm summer	OM3	C0	0	A horizon	21.5	1.3	0.1	5.7	1.7	20.3
A8053B	A8053	BS_ON_	Ontario	A8	Forest Soil	Whole Community DNA	PCR	Amplicon	pyrotag library	454 GS FLX Titanium	European Nucleotide Archive	https://www.ebi.ac.uk/ena/data/search?query=PRJEB8599	ERS662651	SAMEA3261655	ERX708898	ERR765625	16S rRNA	V1-V3	TCACGTACTA	AGAGTTTGATCMTGGCTCAG	GWATTACCGCGGCKGCTG	2011-07-04	49.08	-89.38	Canada	0.1	450	1.8	266	Orthic Dystric Brunisol	Black Spruce	Dfb, Humid Continental warm summer	OM1	C0	0	O horizon	52.1	37.3	0.8	4.5	0.2	47.4
A8054B	A8054	BS_ON_	Ontario	A8	Forest Soil	Whole Community DNA	PCR	Amplicon	pyrotag library	454 GS FLX Titanium	European Nucleotide Archive	https://www.ebi.ac.uk/ena/data/search?query=PRJEB8599	ERS662652	SAMEA3261656	ERX708899	ERR765626	16S rRNA	V1-V3	CTCGCGTGTC	AGAGTTTGATCMTGGCTCAG	GWATTACCGCGGCKGCTG	2011-07-04	49.08	-89.38	Canada	0.3	450	1.8	266	Orthic Dystric Brunisol	Black Spruce	Dfb, Humid Continental warm summer	OM1	C0	0	A horizon	23.4	1.7	0.1	5.4	1.3	23.7
A8055B	A8055	BS_ON_	Ontario	A8	Forest Soil	Whole Community DNA	PCR	Amplicon	pyrotag library	454 GS FLX Titanium	European Nucleotide Archive	https://www.ebi.ac.uk/ena/data/search?query=PRJEB8599	ERS662653	SAMEA3261657	ERX708900	ERR765627	16S rRNA	V1-V3	TGTGAGTAGT	AGAGTTTGATCMTGGCTCAG	GWATTACCGCGGCKGCTG	2011-07-04	49.08	-89.38	Canada	0.1	450	1.8	266	Orthic Dystric Brunisol	Black Spruce	Dfb, Humid Continental warm summer	REF	REF	0	O horizon	73.3	45.8	1.1	4.1	0.2	41.8
A8056B	A8056	BS_ON_	Ontario	A8	Forest Soil	Whole Community DNA	PCR	Amplicon	pyrotag library	454 GS FLX Titanium	European Nucleotide Archive	https://www.ebi.ac.uk/ena/data/search?query=PRJEB8599	ERS662654	SAMEA3261658	ERX708901	ERR765628	16S rRNA	V1-V3	CATAGTAGTG	AGAGTTTGATCMTGGCTCAG	GWATTACCGCGGCKGCTG	2011-07-04	49.08	-89.38	Canada	0.3	450	1.8	266	Orthic Dystric Brunisol	Black Spruce	Dfb, Humid Continental warm summer	REF	REF	0	A horizon	26.0	2.5	0.1	5.2	1.0	23.1
A8057B	A8057	BS_ON_	Ontario	A8	Forest Soil	Whole Community DNA	PCR	Amplicon	pyrotag library	454 GS FLX Titanium	European Nucleotide Archive	https://www.ebi.ac.uk/ena/data/search?query=PRJEB8599	ERS662655	SAMEA3261659	ERX708902	ERR765629	16S rRNA	V1-V3	TGACGTATGT	AGAGTTTGATCMTGGCTCAG	GWATTACCGCGGCKGCTG	2011-07-04	49.08	-89.38	Canada	0.1	450	1.8	266	Orthic Dystric Brunisol	Black Spruce	Dfb, Humid Continental warm summer	REF	REF	0	O horizon	75.7	44.3	1.0	4.7	0.1	43.8
A8058B	A8058	BS_ON_	Ontario	A8	Forest Soil	Whole Community DNA	PCR	Amplicon	pyrotag library	454 GS FLX Titanium	European Nucleotide Archive	https://www.ebi.ac.uk/ena/data/search?query=PRJEB8599	ERS662656	SAMEA3261660	ERX708903	ERR765630	16S rRNA	V1-V3	ACTACTATGT	AGAGTTTGATCMTGGCTCAG	GWATTACCGCGGCKGCTG	2011-07-04	49.08	-89.38	Canada	0.3	450	1.8	266	Orthic Dystric Brunisol	Black Spruce	Dfb, Humid Continental warm summer	REF	REF	0	A horizon	27.5	3.3	0.1	4.9	0.8	27.3
A8059B	A8059	BS_ON_	Ontario	A8	Forest Soil	Whole Community DNA	PCR	Amplicon	pyrotag library	454 GS FLX Titanium	European Nucleotide Archive	https://www.ebi.ac.uk/ena/data/search?query=PRJEB8599	ERS662657	SAMEA3261661	ERX708904	ERR765631	16S rRNA	V1-V3	ATAGAGTACT	AGAGTTTGATCMTGGCTCAG	GWATTACCGCGGCKGCTG	2011-07-04	49.08	-89.38	Canada	0.1	450	1.8	266	Orthic Dystric Brunisol	Black Spruce	Dfb, Humid Continental warm summer	REF	REF	0	O horizon	73.2	45.7	1.1	4.2	0.1	43.5
A8060B	A8060	BS_ON_	Ontario	A8	Forest Soil	Whole Community DNA	PCR	Amplicon	pyrotag library	454 GS FLX Titanium	European Nucleotide Archive	https://www.ebi.ac.uk/ena/data/search?query=PRJEB8599	ERS662658	SAMEA3261662	ERX708905	ERR765632	16S rRNA	V1-V3	TACACGTGAT	AGAGTTTGATCMTGGCTCAG	GWATTACCGCGGCKGCTG	2011-07-04	49.08	-89.38	Canada	0.3	450	1.8	266	Orthic Dystric Brunisol	Black Spruce	Dfb, Humid Continental warm summer	REF	REF	0	A horizon	28.8	1.7	0.1	5.2	1.0	18.4
A9061B	A9061	BS_ON_	Ontario	A9	Forest Soil	Whole Community DNA	PCR	Amplicon	pyrotag library	454 GS FLX Titanium	European Nucleotide Archive	https://www.ebi.ac.uk/ena/data/search?query=PRJEB8599	ERS662659	SAMEA3261663	ERX708906	ERR765633	16S rRNA	V1-V3	ACTAGCAGTA	AGAGTTTGATCMTGGCTCAG	GWATTACCGCGGCKGCTG	2011-07-05	49.07	-89.39	Canada	0.1	442	1.5	266	Gleyed Dystric Brunisol	Black Spruce	Dfb, Humid Continental warm summer	OM1	C0	0	O horizon	59.8	39.6	1.1	5.2	0.2	34.6
A9062B	A9062	BS_ON_	Ontario	A9	Forest Soil	Whole Community DNA	PCR	Amplicon	pyrotag library	454 GS FLX Titanium	European Nucleotide Archive	https://www.ebi.ac.uk/ena/data/search?query=PRJEB8599	ERS662660	SAMEA3261664	ERX708907	ERR765634	16S rRNA	V1-V3	ATCAGACACG	AGAGTTTGATCMTGGCTCAG	GWATTACCGCGGCKGCTG	2011-07-05	49.07	-89.39	Canada	0.3	442	1.5	266	Gleyed Dystric Brunisol	Black Spruce	Dfb, Humid Continental warm summer	OM1	C0	0	A horizon	20.3	3.3	0.1	5.4	0.9	23.5
A9063B	A9063	BS_ON_	Ontario	A9	Forest Soil	Whole Community DNA	PCR	Amplicon	pyrotag library	454 GS FLX Titanium	European Nucleotide Archive	https://www.ebi.ac.uk/ena/data/search?query=PRJEB8599	ERS662661	SAMEA3261665	ERX708908	ERR765635	16S rRNA	V1-V3	ATACGACGTA	AGAGTTTGATCMTGGCTCAG	GWATTACCGCGGCKGCTG	2011-07-05	49.07	-89.39	Canada	0.1	442	1.5	266	Gleyed Dystric Brunisol	Black Spruce	Dfb, Humid Continental warm summer	OM2	C0	0	O horizon	59.2	35.3	1.1	5.4	0.2	31.9
A9064B	A9064	BS_ON_	Ontario	A9	Forest Soil	Whole Community DNA	PCR	Amplicon	pyrotag library	454 GS FLX Titanium	European Nucleotide Archive	https://www.ebi.ac.uk/ena/data/search?query=PRJEB8599	ERS662662	SAMEA3261666	ERX708909	ERR765636	16S rRNA	V1-V3	AGTACGCTAT	AGAGTTTGATCMTGGCTCAG	GWATTACCGCGGCKGCTG	2011-07-05	49.07	-89.39	Canada	0.3	442	1.5	266	Gleyed Dystric Brunisol	Black Spruce	Dfb, Humid Continental warm summer	OM2	C0	0	A horizon	24.2	2.2	0.1	5.6	1.2	21.2
A9067B	A9067	BS_ON_	Ontario	A9	Forest Soil	Whole Community DNA	PCR	Amplicon	pyrotag library	454 GS FLX Titanium	European Nucleotide Archive	https://www.ebi.ac.uk/ena/data/search?query=PRJEB8599	ERS662663	SAMEA3261667	ERX708910	ERR765637	16S rRNA	V1-V3	CACGCTACGT	AGAGTTTGATCMTGGCTCAG	GWATTACCGCGGCKGCTG	2011-07-05	49.07	-89.39	Canada	0.1	442	1.5	266	Gleyed Dystric Brunisol	Black Spruce	Dfb, Humid Continental warm summer	OM3	C0	0	O horizon	53.0	32.6	0.9	5.4	0.2	37.2
A9068B	A9068	BS_ON_	Ontario	A9	Forest Soil	Whole Community DNA	PCR	Amplicon	pyrotag library	454 GS FLX Titanium	European Nucleotide Archive	https://www.ebi.ac.uk/ena/data/search?query=PRJEB8599	ERS662664	SAMEA3261668	ERX708911	ERR765638	16S rRNA	V1-V3	TACACGTGAT	AGAGTTTGATCMTGGCTCAG	GWATTACCGCGGCKGCTG	2011-07-05	49.07	-89.39	Canada	0.3	442	1.5	266	Gleyed Dystric Brunisol	Black Spruce	Dfb, Humid Continental warm summer	OM3	C0	0	A horizon	22.8	2.3	0.1	5.8	1.2	18.3
A9069B	A9069	BS_ON_	Ontario	A9	Forest Soil	Whole Community DNA	PCR	Amplicon	pyrotag library	454 GS FLX Titanium	European Nucleotide Archive	https://www.ebi.ac.uk/ena/data/search?query=PRJEB8599	ERS662665	SAMEA3261669	ERX708912	ERR765639	16S rRNA	V1-V3	ACAGTATATA	AGAGTTTGATCMTGGCTCAG	GWATTACCGCGGCKGCTG	2011-07-05	49.07	-89.39	Canada	0.1	442	1.5	266	Gleyed Dystric Brunisol	Black Spruce	Dfb, Humid Continental warm summer	OM2	C0	0	O horizon	42.5	45.5	1.3	4.9	0.3	36.5
A9070B	A9070	BS_ON_	Ontario	A9	Forest Soil	Whole Community DNA	PCR	Amplicon	pyrotag library	454 GS FLX Titanium	European Nucleotide Archive	https://www.ebi.ac.uk/ena/data/search?query=PRJEB8599	ERS662666	SAMEA3261670	ERX708913	ERR765640	16S rRNA	V1-V3	TCTCTATGCG	AGAGTTTGATCMTGGCTCAG	GWATTACCGCGGCKGCTG	2011-07-05	49.07	-89.39	Canada	0.3	442	1.5	266	Gleyed Dystric Brunisol	Black Spruce	Dfb, Humid Continental warm summer	OM2	C0	0	A horizon	20.2	0.7	0.0	5.8	1.2	17.5
A9073B	A9073	BS_ON_	Ontario	A9	Forest Soil	Whole Community DNA	PCR	Amplicon	pyrotag library	454 GS FLX Titanium	European Nucleotide Archive	https://www.ebi.ac.uk/ena/data/search?query=PRJEB8599	ERS662667	SAMEA3261671	ERX708914	ERR765641	16S rRNA	V1-V3	TACGCTGTCT	AGAGTTTGATCMTGGCTCAG	GWATTACCGCGGCKGCTG	2011-07-05	49.07	-89.39	Canada	0.1	442	1.5	266	Gleyed Dystric Brunisol	Black Spruce	Dfb, Humid Continental warm summer	OM2	C0	0	O horizon	64.4	45.1	1.1	5.0	0.2	40.2
A9074B	A9074	BS_ON_	Ontario	A9	Forest Soil	Whole Community DNA	PCR	Amplicon	pyrotag library	454 GS FLX Titanium	European Nucleotide Archive	https://www.ebi.ac.uk/ena/data/search?query=PRJEB8599	ERS662668	SAMEA3261672	ERX708915	ERR765642	16S rRNA	V1-V3	TCGATCACGT	AGAGTTTGATCMTGGCTCAG	GWATTACCGCGGCKGCTG	2011-07-05	49.07	-89.39	Canada	0.3	442	1.5	266	Gleyed Dystric Brunisol	Black Spruce	Dfb, Humid Continental warm summer	OM2	C0	0	A horizon	32.1	2.8	0.1	5.3	1.2	24.4
A9075B	A9075	BS_ON_	Ontario	A9	Forest Soil	Whole Community DNA	PCR	Amplicon	pyrotag library	454 GS FLX Titanium	European Nucleotide Archive	https://www.ebi.ac.uk/ena/data/search?query=PRJEB8599	ERS662669	SAMEA3261673	ERX708916	ERR765643	16S rRNA	V1-V3	ACGCGATCGA	AGAGTTTGATCMTGGCTCAG	GWATTACCGCGGCKGCTG	2011-07-05	49.07	-89.39	Canada	0.1	442	1.5	266	Gleyed Dystric Brunisol	Black Spruce	Dfb, Humid Continental warm summer	OM1	C0	0	O horizon	47.0	42.7	0.9	4.9	0.2	47.4
A9076B	A9076	BS_ON_	Ontario	A9	Forest Soil	Whole Community DNA	PCR	Amplicon	pyrotag library	454 GS FLX Titanium	European Nucleotide Archive	https://www.ebi.ac.uk/ena/data/search?query=PRJEB8599	ERS662670	SAMEA3261674	ERX708917	ERR765644	16S rRNA	V1-V3	CACGCTACGT	AGAGTTTGATCMTGGCTCAG	GWATTACCGCGGCKGCTG	2011-07-05	49.07	-89.39	Canada	0.3	442	1.5	266	Gleyed Dystric Brunisol	Black Spruce	Dfb, Humid Continental warm summer	OM1	C0	0	A horizon	20.8	1.5	0.1	5.6	1.2	19.3
A9077B	A9077	BS_ON_	Ontario	A9	Forest Soil	Whole Community DNA	PCR	Amplicon	pyrotag library	454 GS FLX Titanium	European Nucleotide Archive	https://www.ebi.ac.uk/ena/data/search?query=PRJEB8599	ERS662671	SAMEA3261675	ERX708918	ERR765645	16S rRNA	V1-V3	TAGTGTAGAT	AGAGTTTGATCMTGGCTCAG	GWATTACCGCGGCKGCTG	2011-07-05	49.07	-89.39	Canada	0.1	442	1.5	266	Gleyed Dystric Brunisol	Black Spruce	Dfb, Humid Continental warm summer	OM3	C0	0	O horizon	41.7	27.7	0.7	5.8	0.2	40.1
A9078B	A9078	BS_ON_	Ontario	A9	Forest Soil	Whole Community DNA	PCR	Amplicon	pyrotag library	454 GS FLX Titanium	European Nucleotide Archive	https://www.ebi.ac.uk/ena/data/search?query=PRJEB8599	ERS662672	SAMEA3261676	ERX708919	ERR765646	16S rRNA	V1-V3	AGCACTGTAG	AGAGTTTGATCMTGGCTCAG	GWATTACCGCGGCKGCTG	2011-07-05	49.07	-89.39	Canada	0.3	442	1.5	266	Gleyed Dystric Brunisol	Black Spruce	Dfb, Humid Continental warm summer	OM3	C0	0	A horizon	14.9	0.7	0.0	6.0	1.3	18.6
A9081B	A9081	BS_ON_	Ontario	A9	Forest Soil	Whole Community DNA	PCR	Amplicon	pyrotag library	454 GS FLX Titanium	European Nucleotide Archive	https://www.ebi.ac.uk/ena/data/search?query=PRJEB8599	ERS662673	SAMEA3261677	ERX708920	ERR765647	16S rRNA	V1-V3	TCTCTATGCG	AGAGTTTGATCMTGGCTCAG	GWATTACCGCGGCKGCTG	2011-07-05	49.07	-89.39	Canada	0.1	442	1.5	266	Gleyed Dystric Brunisol	Black Spruce	Dfb, Humid Continental warm summer	OM3	C0	0	O horizon	76.4	13.4	0.3	5.3	0.2	46.2
A9082B	A9082	BS_ON_	Ontario	A9	Forest Soil	Whole Community DNA	PCR	Amplicon	pyrotag library	454 GS FLX Titanium	European Nucleotide Archive	https://www.ebi.ac.uk/ena/data/search?query=PRJEB8599	ERS662674	SAMEA3261678	ERX708921	ERR765648	16S rRNA	V1-V3	AGACGCACTC	AGAGTTTGATCMTGGCTCAG	GWATTACCGCGGCKGCTG	2011-07-05	49.07	-89.39	Canada	0.3	442	1.5	266	Gleyed Dystric Brunisol	Black Spruce	Dfb, Humid Continental warm summer	OM3	C0	0	A horizon	18.6	1.6	0.1	6.1	1.1	19.9
A9083B	A9083	BS_ON_	Ontario	A9	Forest Soil	Whole Community DNA	PCR	Amplicon	pyrotag library	454 GS FLX Titanium	European Nucleotide Archive	https://www.ebi.ac.uk/ena/data/search?query=PRJEB8599	ERS662675	SAMEA3261679	ERX708922	ERR765649	16S rRNA	V1-V3	ACGAGTGCGT	AGAGTTTGATCMTGGCTCAG	GWATTACCGCGGCKGCTG	2011-07-05	49.07	-89.39	Canada	0.1	442	1.5	266	Gleyed Dystric Brunisol	Black Spruce	Dfb, Humid Continental warm summer	OM1	C0	0	O horizon	69.0	29.0	0.9	5.4	0.2	30.9
A9084B	A9084	BS_ON_	Ontario	A9	Forest Soil	Whole Community DNA	PCR	Amplicon	pyrotag library	454 GS FLX Titanium	European Nucleotide Archive	https://www.ebi.ac.uk/ena/data/search?query=PRJEB8599	ERS662676	SAMEA3261680	ERX708923	ERR765650	16S rRNA	V1-V3	ACAGTATATA	AGAGTTTGATCMTGGCTCAG	GWATTACCGCGGCKGCTG	2011-07-05	49.07	-89.39	Canada	0.3	442	1.5	266	Gleyed Dystric Brunisol	Black Spruce	Dfb, Humid Continental warm summer	OM1	C0	0	A horizon	25.6	2.8	0.1	5.4	1.2	27.8
A9085B	A9085	BS_ON_	Ontario	A9	Forest Soil	Whole Community DNA	PCR	Amplicon	pyrotag library	454 GS FLX Titanium	European Nucleotide Archive	https://www.ebi.ac.uk/ena/data/search?query=PRJEB8599	ERS662677	SAMEA3261681	ERX708924	ERR765651	16S rRNA	V1-V3	CGTCTAGTAC	AGAGTTTGATCMTGGCTCAG	GWATTACCGCGGCKGCTG	2011-07-05	49.07	-89.39	Canada	0.1	442	1.5	266	Gleyed Dystric Brunisol	Black Spruce	Dfb, Humid Continental warm summer	REF	REF	0	O horizon	64.4	43.9	1.2	4.8	0.1	35.7
A9086B	A9086	BS_ON_	Ontario	A9	Forest Soil	Whole Community DNA	PCR	Amplicon	pyrotag library	454 GS FLX Titanium	European Nucleotide Archive	https://www.ebi.ac.uk/ena/data/search?query=PRJEB8599	ERS662678	SAMEA3261682	ERX708925	ERR765652	16S rRNA	V1-V3	AGTACGCTAT	AGAGTTTGATCMTGGCTCAG	GWATTACCGCGGCKGCTG	2011-07-05	49.07	-89.39	Canada	0.3	442	1.5	266	Gleyed Dystric Brunisol	Black Spruce	Dfb, Humid Continental warm summer	REF	REF	0	A horizon	28.2	1.9	0.1	5.4	1.2	18.9
A9087B	A9087	BS_ON_	Ontario	A9	Forest Soil	Whole Community DNA	PCR	Amplicon	pyrotag library	454 GS FLX Titanium	European Nucleotide Archive	https://www.ebi.ac.uk/ena/data/search?query=PRJEB8599	ERS662679	SAMEA3261683	ERX708926	ERR765653	16S rRNA	V1-V3	ATATCGCGAG	AGAGTTTGATCMTGGCTCAG	GWATTACCGCGGCKGCTG	2011-07-05	49.07	-89.39	Canada	0.1	442	1.5	266	Gleyed Dystric Brunisol	Black Spruce	Dfb, Humid Continental warm summer	REF	REF	0	O horizon	57.4	45.5	1.1	4.8	0.1	39.9
A9088B	A9088	BS_ON_	Ontario	A9	Forest Soil	Whole Community DNA	PCR	Amplicon	pyrotag library	454 GS FLX Titanium	European Nucleotide Archive	https://www.ebi.ac.uk/ena/data/search?query=PRJEB8599	ERS662680	SAMEA3261684	ERX708927	ERR765654	16S rRNA	V1-V3	AGCGTCGTCT	AGAGTTTGATCMTGGCTCAG	GWATTACCGCGGCKGCTG	2011-07-05	49.07	-89.39	Canada	0.3	442	1.5	266	Gleyed Dystric Brunisol	Black Spruce	Dfb, Humid Continental warm summer	REF	REF	0	A horizon	30.4	1.4	0.1	5.9	1.4	20.1
A9089B	A9089	BS_ON_	Ontario	A9	Forest Soil	Whole Community DNA	PCR	Amplicon	pyrotag library	454 GS FLX Titanium	European Nucleotide Archive	https://www.ebi.ac.uk/ena/data/search?query=PRJEB8599	ERS662681	SAMEA3261685	ERX708928	ERR765655	16S rRNA	V1-V3	TACACACACT	AGAGTTTGATCMTGGCTCAG	GWATTACCGCGGCKGCTG	2011-07-05	49.07	-89.39	Canada	0.1	442	1.5	266	Gleyed Dystric Brunisol	Black Spruce	Dfb, Humid Continental warm summer	REF	REF	0	O horizon	71.0	44.2	1.0	4.7	0.1	43.1
A9090B	A9090	BS_ON_	Ontario	A9	Forest Soil	Whole Community DNA	PCR	Amplicon	pyrotag library	454 GS FLX Titanium	European Nucleotide Archive	https://www.ebi.ac.uk/ena/data/search?query=PRJEB8599	ERS662682	SAMEA3261686	ERX708929	ERR765656	16S rRNA	V1-V3	ATATCGCGAG	AGAGTTTGATCMTGGCTCAG	GWATTACCGCGGCKGCTG	2011-07-05	49.07	-89.39	Canada	0.3	442	1.5	266	Gleyed Dystric Brunisol	Black Spruce	Dfb, Humid Continental warm summer	REF	REF	0	A horizon	25.0	1.2	0.1	5.6	1.5	22.3
BL025B	BL025	PP_CA_	California	BL	Forest Soil	Whole Community DNA	PCR	Amplicon	pyrotag library	454 GS FLX Titanium	European Nucleotide Archive	https://www.ebi.ac.uk/ena/data/search?query=PRJEB8599	ERS662701	SAMEA3261705	ERX708948	ERR765675	16S rRNA	V1-V3	ATATCGCGAG	AGAGTTTGATCMTGGCTCAG	GWATTACCGCGGCKGCTG	2011-09-16	38.88	-120.64	USA	0.1	1350	11.2	55	Mesic Ultic Haploxeralfs	Ponderosa pine, sugar pine, white fir, giant sequoia	Csa, Mediterranean hot summer	OM1	C0	0	O horizon	18.4	NA	NA	4.8	NA	NA
BL026B	BL026	PP_CA_	California	BL	Forest Soil	Whole Community DNA	PCR	Amplicon	pyrotag library	454 GS FLX Titanium	European Nucleotide Archive	https://www.ebi.ac.uk/ena/data/search?query=PRJEB8599	ERS662702	SAMEA3261706	ERX708949	ERR765676	16S rRNA	V1-V3	ACGCGAGTAT	AGAGTTTGATCMTGGCTCAG	GWATTACCGCGGCKGCTG	2011-09-16	38.88	-120.64	USA	0.3	1350	11.2	55	Mesic Ultic Haploxeralfs	Ponderosa pine, sugar pine, white fir, giant sequoia	Csa, Mediterranean hot summer	OM1	C0	0	A horizon	22.0	5.5	0.3	5.7	NA	19.8
BL027B	BL027	PP_CA_	California	BL	Forest Soil	Whole Community DNA	PCR	Amplicon	pyrotag library	454 GS FLX Titanium	European Nucleotide Archive	https://www.ebi.ac.uk/ena/data/search?query=PRJEB8599	ERS662703	SAMEA3261707	ERX708950	ERR765677	16S rRNA	V1-V3	TCTACGTAGC	AGAGTTTGATCMTGGCTCAG	GWATTACCGCGGCKGCTG	2011-09-16	38.88	-120.64	USA	0.1	1350	11.2	55	Mesic Ultic Haploxeralfs	Ponderosa pine, sugar pine, white fir, giant sequoia	Csa, Mediterranean hot summer	OM1	C0	0	O horizon	16.7	NA	NA	4.9	NA	NA
BL028B	BL028	PP_CA_	California	BL	Forest Soil	Whole Community DNA	PCR	Amplicon	pyrotag library	454 GS FLX Titanium	European Nucleotide Archive	https://www.ebi.ac.uk/ena/data/search?query=PRJEB8599	ERS662704	SAMEA3261708	ERX708951	ERR765678	16S rRNA	V1-V3	ATAGAGTACT	AGAGTTTGATCMTGGCTCAG	GWATTACCGCGGCKGCTG	2011-09-16	38.88	-120.64	USA	0.3	1350	11.2	55	Mesic Ultic Haploxeralfs	Ponderosa pine, sugar pine, white fir, giant sequoia	Csa, Mediterranean hot summer	OM1	C0	0	A horizon	21.0	5.5	0.3	5.9	NA	19.8
BL029B	BL029	PP_CA_	California	BL	Forest Soil	Whole Community DNA	PCR	Amplicon	pyrotag library	454 GS FLX Titanium	European Nucleotide Archive	https://www.ebi.ac.uk/ena/data/search?query=PRJEB8599	ERS662705	SAMEA3261709	ERX708952	ERR765679	16S rRNA	V1-V3	ACTACTATGT	AGAGTTTGATCMTGGCTCAG	GWATTACCGCGGCKGCTG	2011-09-16	38.88	-120.64	USA	0.1	1350	11.2	55	Mesic Ultic Haploxeralfs	Ponderosa pine, sugar pine, white fir, giant sequoia	Csa, Mediterranean hot summer	OM1	C0	0	O horizon	18.9	NA	NA	4.7	NA	NA
BL030B	BL030	PP_CA_	California	BL	Forest Soil	Whole Community DNA	PCR	Amplicon	pyrotag library	454 GS FLX Titanium	European Nucleotide Archive	https://www.ebi.ac.uk/ena/data/search?query=PRJEB8599	ERS662706	SAMEA3261710	ERX708953	ERR765680	16S rRNA	V1-V3	CGTGTCTCTA	AGAGTTTGATCMTGGCTCAG	GWATTACCGCGGCKGCTG	2011-09-16	38.88	-120.64	USA	0.3	1350	11.2	55	Mesic Ultic Haploxeralfs	Ponderosa pine, sugar pine, white fir, giant sequoia	Csa, Mediterranean hot summer	OM1	C0	0	A horizon	23.7	5.5	0.3	5.9	NA	19.8
BL031B	BL031	PP_CA_	California	BL	Forest Soil	Whole Community DNA	PCR	Amplicon	pyrotag library	454 GS FLX Titanium	European Nucleotide Archive	https://www.ebi.ac.uk/ena/data/search?query=PRJEB8599	ERS662707	SAMEA3261711	ERX708954	ERR765681	16S rRNA	V1-V3	TCGATCACGT	AGAGTTTGATCMTGGCTCAG	GWATTACCGCGGCKGCTG	2011-09-16	38.88	-120.64	USA	0.1	1350	11.2	55	Mesic Ultic Haploxeralfs	Ponderosa pine, sugar pine, white fir, giant sequoia	Csa, Mediterranean hot summer	OM2	C0	0	O horizon	28.6	NA	NA	5.7	NA	NA
BL032B	BL032	PP_CA_	California	BL	Forest Soil	Whole Community DNA	PCR	Amplicon	pyrotag library	454 GS FLX Titanium	European Nucleotide Archive	https://www.ebi.ac.uk/ena/data/search?query=PRJEB8599	ERS662708	SAMEA3261712	ERX708955	ERR765682	16S rRNA	V1-V3	ATCAGACACG	AGAGTTTGATCMTGGCTCAG	GWATTACCGCGGCKGCTG	2011-09-16	38.88	-120.64	USA	0.3	1350	11.2	55	Mesic Ultic Haploxeralfs	Ponderosa pine, sugar pine, white fir, giant sequoia	Csa, Mediterranean hot summer	OM2	C0	0	A horizon	21.0	5.4	0.3	5.8	NA	20.6
BL033B	BL033	PP_CA_	California	BL	Forest Soil	Whole Community DNA	PCR	Amplicon	pyrotag library	454 GS FLX Titanium	European Nucleotide Archive	https://www.ebi.ac.uk/ena/data/search?query=PRJEB8599	ERS662709	SAMEA3261713	ERX708956	ERR765683	16S rRNA	V1-V3	CAGTAGACGT	AGAGTTTGATCMTGGCTCAG	GWATTACCGCGGCKGCTG	2011-09-16	38.88	-120.64	USA	0.1	1350	11.2	55	Mesic Ultic Haploxeralfs	Ponderosa pine, sugar pine, white fir, giant sequoia	Csa, Mediterranean hot summer	OM2	C0	0	O horizon	21.7	NA	NA	5.2	NA	NA
BL034B	BL034	PP_CA_	California	BL	Forest Soil	Whole Community DNA	PCR	Amplicon	pyrotag library	454 GS FLX Titanium	European Nucleotide Archive	https://www.ebi.ac.uk/ena/data/search?query=PRJEB8599	ERS662710	SAMEA3261714	ERX708957	ERR765684	16S rRNA	V1-V3	CTCGCGTGTC	AGAGTTTGATCMTGGCTCAG	GWATTACCGCGGCKGCTG	2011-09-16	38.88	-120.64	USA	0.3	1350	11.2	55	Mesic Ultic Haploxeralfs	Ponderosa pine, sugar pine, white fir, giant sequoia	Csa, Mediterranean hot summer	OM2	C0	0	A horizon	20.5	5.4	0.3	5.3	NA	20.6
BL035B	BL035	PP_CA_	California	BL	Forest Soil	Whole Community DNA	PCR	Amplicon	pyrotag library	454 GS FLX Titanium	European Nucleotide Archive	https://www.ebi.ac.uk/ena/data/search?query=PRJEB8599	ERS662711	SAMEA3261715	ERX708958	ERR765685	16S rRNA	V1-V3	ACGCTCGACA	AGAGTTTGATCMTGGCTCAG	GWATTACCGCGGCKGCTG	2011-09-16	38.88	-120.64	USA	0.1	1350	11.2	55	Mesic Ultic Haploxeralfs	Ponderosa pine, sugar pine, white fir, giant sequoia	Csa, Mediterranean hot summer	OM2	C0	0	O horizon	16.4	NA	NA	5.1	NA	NA
BL036B	BL036	PP_CA_	California	BL	Forest Soil	Whole Community DNA	PCR	Amplicon	pyrotag library	454 GS FLX Titanium	European Nucleotide Archive	https://www.ebi.ac.uk/ena/data/search?query=PRJEB8599	ERS662712	SAMEA3261716	ERX708959	ERR765686	16S rRNA	V1-V3	TCTATACTAT	AGAGTTTGATCMTGGCTCAG	GWATTACCGCGGCKGCTG	2011-09-16	38.88	-120.64	USA	0.3	1350	11.2	55	Mesic Ultic Haploxeralfs	Ponderosa pine, sugar pine, white fir, giant sequoia	Csa, Mediterranean hot summer	OM2	C0	0	A horizon	22.3	5.4	0.3	5.7	NA	20.6
BL037B	BL037	PP_CA_	California	BL	Forest Soil	Whole Community DNA	PCR	Amplicon	pyrotag library	454 GS FLX Titanium	European Nucleotide Archive	https://www.ebi.ac.uk/ena/data/search?query=PRJEB8599	ERS662713	SAMEA3261717	ERX708960	ERR765687	16S rRNA	V1-V3	ACAGTATATA	AGAGTTTGATCMTGGCTCAG	GWATTACCGCGGCKGCTG	2011-09-16	38.88	-120.64	USA	0.1	1350	11.2	55	Mesic Ultic Haploxeralfs	Ponderosa pine, sugar pine, white fir, giant sequoia	Csa, Mediterranean hot summer	OM3	C0	0	O horizon	21.1	NA	NA	5.2	NA	NA
BL038B	BL038	PP_CA_	California	BL	Forest Soil	Whole Community DNA	PCR	Amplicon	pyrotag library	454 GS FLX Titanium	European Nucleotide Archive	https://www.ebi.ac.uk/ena/data/search?query=PRJEB8599	ERS662714	SAMEA3261718	ERX708961	ERR765688	16S rRNA	V1-V3	CTCGCGTGTC	AGAGTTTGATCMTGGCTCAG	GWATTACCGCGGCKGCTG	2011-09-16	38.88	-120.64	USA	0.3	1350	11.2	55	Mesic Ultic Haploxeralfs	Ponderosa pine, sugar pine, white fir, giant sequoia	Csa, Mediterranean hot summer	OM3	C0	0	A horizon	21.7	5.3	0.3	5.4	NA	18.9
BL039B	BL039	PP_CA_	California	BL	Forest Soil	Whole Community DNA	PCR	Amplicon	pyrotag library	454 GS FLX Titanium	European Nucleotide Archive	https://www.ebi.ac.uk/ena/data/search?query=PRJEB8599	ERS662715	SAMEA3261719	ERX708962	ERR765689	16S rRNA	V1-V3	TGATACGTCT	AGAGTTTGATCMTGGCTCAG	GWATTACCGCGGCKGCTG	2011-09-16	38.88	-120.64	USA	0.1	1350	11.2	55	Mesic Ultic Haploxeralfs	Ponderosa pine, sugar pine, white fir, giant sequoia	Csa, Mediterranean hot summer	OM3	C0	0	O horizon	24.4	NA	NA	5.3	NA	NA
BL040B	BL040	PP_CA_	California	BL	Forest Soil	Whole Community DNA	PCR	Amplicon	pyrotag library	454 GS FLX Titanium	European Nucleotide Archive	https://www.ebi.ac.uk/ena/data/search?query=PRJEB8599	ERS662716	SAMEA3261720	ERX708963	ERR765690	16S rRNA	V1-V3	TCTAGCGACT	AGAGTTTGATCMTGGCTCAG	GWATTACCGCGGCKGCTG	2011-09-16	38.88	-120.64	USA	0.3	1350	11.2	55	Mesic Ultic Haploxeralfs	Ponderosa pine, sugar pine, white fir, giant sequoia	Csa, Mediterranean hot summer	OM3	C0	0	A horizon	22.2	5.3	0.3	5.9	NA	18.9
BL041B	BL041	PP_CA_	California	BL	Forest Soil	Whole Community DNA	PCR	Amplicon	pyrotag library	454 GS FLX Titanium	European Nucleotide Archive	https://www.ebi.ac.uk/ena/data/search?query=PRJEB8599	ERS662717	SAMEA3261721	ERX708964	ERR765691	16S rRNA	V1-V3	ACGCTCGACA	AGAGTTTGATCMTGGCTCAG	GWATTACCGCGGCKGCTG	2011-09-16	38.88	-120.64	USA	0.1	1350	11.2	55	Mesic Ultic Haploxeralfs	Ponderosa pine, sugar pine, white fir, giant sequoia	Csa, Mediterranean hot summer	OM3	C0	0	O horizon	23.2	NA	NA	5.1	NA	NA
BL042B	BL042	PP_CA_	California	BL	Forest Soil	Whole Community DNA	PCR	Amplicon	pyrotag library	454 GS FLX Titanium	European Nucleotide Archive	https://www.ebi.ac.uk/ena/data/search?query=PRJEB8599	ERS662718	SAMEA3261722	ERX708965	ERR765692	16S rRNA	V1-V3	TCGCACTAGT	AGAGTTTGATCMTGGCTCAG	GWATTACCGCGGCKGCTG	2011-09-16	38.88	-120.64	USA	0.3	1350	11.2	55	Mesic Ultic Haploxeralfs	Ponderosa pine, sugar pine, white fir, giant sequoia	Csa, Mediterranean hot summer	OM3	C0	0	A horizon	21.5	5.3	0.3	5.4	NA	18.9
BL043B	BL043	PP_CA_	California	BL	Forest Soil	Whole Community DNA	PCR	Amplicon	pyrotag library	454 GS FLX Titanium	European Nucleotide Archive	https://www.ebi.ac.uk/ena/data/search?query=PRJEB8599	ERS662719	SAMEA3261723	ERX708966	ERR765693	16S rRNA	V1-V3	TACACACACT	AGAGTTTGATCMTGGCTCAG	GWATTACCGCGGCKGCTG	2011-09-16	38.88	-120.64	USA	0.1	1350	11.2	55	Mesic Ultic Haploxeralfs	Ponderosa pine, sugar pine, white fir, giant sequoia	Csa, Mediterranean hot summer	REF	REF	0	O horizon	27.0	NA	NA	4.5	NA	NA
BL044B	BL044	PP_CA_	California	BL	Forest Soil	Whole Community DNA	PCR	Amplicon	pyrotag library	454 GS FLX Titanium	European Nucleotide Archive	https://www.ebi.ac.uk/ena/data/search?query=PRJEB8599	ERS662720	SAMEA3261724	ERX708967	ERR765694	16S rRNA	V1-V3	ATCAGACACG	AGAGTTTGATCMTGGCTCAG	GWATTACCGCGGCKGCTG	2011-09-16	38.88	-120.64	USA	0.3	1350	11.2	55	Mesic Ultic Haploxeralfs	Ponderosa pine, sugar pine, white fir, giant sequoia	Csa, Mediterranean hot summer	REF	REF	0	A horizon	17.8	6.3	0.3	5.5	NA	20.3
BL045B	BL045	PP_CA_	California	BL	Forest Soil	Whole Community DNA	PCR	Amplicon	pyrotag library	454 GS FLX Titanium	European Nucleotide Archive	https://www.ebi.ac.uk/ena/data/search?query=PRJEB8599	ERS662721	SAMEA3261725	ERX708968	ERR765695	16S rRNA	V1-V3	TCGCACTAGT	AGAGTTTGATCMTGGCTCAG	GWATTACCGCGGCKGCTG	2011-09-16	38.88	-120.64	USA	0.1	1350	11.2	55	Mesic Ultic Haploxeralfs	Ponderosa pine, sugar pine, white fir, giant sequoia	Csa, Mediterranean hot summer	REF	REF	0	O horizon	25.8	NA	NA	5.3	NA	NA
BL046B	BL046	PP_CA_	California	BL	Forest Soil	Whole Community DNA	PCR	Amplicon	pyrotag library	454 GS FLX Titanium	European Nucleotide Archive	https://www.ebi.ac.uk/ena/data/search?query=PRJEB8599	ERS662722	SAMEA3261726	ERX708969	ERR765696	16S rRNA	V1-V3	TCGATCACGT	AGAGTTTGATCMTGGCTCAG	GWATTACCGCGGCKGCTG	2011-09-16	38.88	-120.64	USA	0.3	1350	11.2	55	Mesic Ultic Haploxeralfs	Ponderosa pine, sugar pine, white fir, giant sequoia	Csa, Mediterranean hot summer	REF	REF	0	A horizon	17.5	6.3	0.3	5.5	NA	20.3
BL047B	BL047	PP_CA_	California	BL	Forest Soil	Whole Community DNA	PCR	Amplicon	pyrotag library	454 GS FLX Titanium	European Nucleotide Archive	https://www.ebi.ac.uk/ena/data/search?query=PRJEB8599	ERS662723	SAMEA3261727	ERX708970	ERR765697	16S rRNA	V1-V3	CACGCTACGT	AGAGTTTGATCMTGGCTCAG	GWATTACCGCGGCKGCTG	2011-09-16	38.88	-120.64	USA	0.1	1350	11.2	55	Mesic Ultic Haploxeralfs	Ponderosa pine, sugar pine, white fir, giant sequoia	Csa, Mediterranean hot summer	REF	REF	0	O horizon	29.8	NA	NA	4.9	NA	NA
BL048B	BL048	PP_CA_	California	BL	Forest Soil	Whole Community DNA	PCR	Amplicon	pyrotag library	454 GS FLX Titanium	European Nucleotide Archive	https://www.ebi.ac.uk/ena/data/search?query=PRJEB8599	ERS662724	SAMEA3261728	ERX708971	ERR765698	16S rRNA	V1-V3	CGAGAGATAC	AGAGTTTGATCMTGGCTCAG	GWATTACCGCGGCKGCTG	2011-09-16	38.88	-120.64	USA	0.3	1350	11.2	55	Mesic Ultic Haploxeralfs	Ponderosa pine, sugar pine, white fir, giant sequoia	Csa, Mediterranean hot summer	REF	REF	0	A horizon	15.3	6.3	0.3	6.0	NA	20.3
BR049B	BR049	PP_CA_	California	BR	Forest Soil	Whole Community DNA	PCR	Amplicon	pyrotag library	454 GS FLX Titanium	European Nucleotide Archive	https://www.ebi.ac.uk/ena/data/search?query=PRJEB8599	ERS662725	SAMEA3261729	ERX708972	ERR765699	16S rRNA	V1-V3	TCTCTATGCG	AGAGTTTGATCMTGGCTCAG	GWATTACCGCGGCKGCTG	2011-06-22	39.55	-121.04	USA	0.1	1135	11.2	55	Mesic Ultic Haploxeralfs	Ponderosa pine, sugar pine, white fir, giant sequoia	Csa, Mediterranean hot summer	OM1	C0	0	O horizon	54.2	41.6	1.6	5.4	NA	25.5
BR050B	BR050	PP_CA_	California	BR	Forest Soil	Whole Community DNA	PCR	Amplicon	pyrotag library	454 GS FLX Titanium	European Nucleotide Archive	https://www.ebi.ac.uk/ena/data/search?query=PRJEB8599	ERS662726	SAMEA3261730	ERX708973	ERR765700	16S rRNA	V1-V3	CGAGAGATAC	AGAGTTTGATCMTGGCTCAG	GWATTACCGCGGCKGCTG	2011-06-22	39.55	-121.04	USA	0.3	1135	11.2	55	Mesic Ultic Haploxeralfs	Ponderosa pine, sugar pine, white fir, giant sequoia	Csa, Mediterranean hot summer	OM1	C0	0	A horizon	35.0	8.5	0.3	5.9	NA	25.3
BR051B	BR051	PP_CA_	California	BR	Forest Soil	Whole Community DNA	PCR	Amplicon	pyrotag library	454 GS FLX Titanium	European Nucleotide Archive	https://www.ebi.ac.uk/ena/data/search?query=PRJEB8599	ERS662727	SAMEA3261731	ERX708974	ERR765701	16S rRNA	V1-V3	ATATCGCGAG	AGAGTTTGATCMTGGCTCAG	GWATTACCGCGGCKGCTG	2011-06-22	39.55	-121.04	USA	0.1	1135	11.2	55	Mesic Ultic Haploxeralfs	Ponderosa pine, sugar pine, white fir, giant sequoia	Csa, Mediterranean hot summer	OM1	C0	0	O horizon	55.1	38.6	1.4	4.3	NA	27.0
BR052B	BR052	PP_CA_	California	BR	Forest Soil	Whole Community DNA	PCR	Amplicon	pyrotag library	454 GS FLX Titanium	European Nucleotide Archive	https://www.ebi.ac.uk/ena/data/search?query=PRJEB8599	ERS662728	SAMEA3261732	ERX708975	ERR765702	16S rRNA	V1-V3	CGTGTCTCTA	AGAGTTTGATCMTGGCTCAG	GWATTACCGCGGCKGCTG	2011-06-22	39.55	-121.04	USA	0.3	1135	11.2	55	Mesic Ultic Haploxeralfs	Ponderosa pine, sugar pine, white fir, giant sequoia	Csa, Mediterranean hot summer	OM1	C0	0	A horizon	33.9	7.8	0.3	5.2	NA	25.1
BR053B	BR053	PP_CA_	California	BR	Forest Soil	Whole Community DNA	PCR	Amplicon	pyrotag library	454 GS FLX Titanium	European Nucleotide Archive	https://www.ebi.ac.uk/ena/data/search?query=PRJEB8599	ERS662729	SAMEA3261733	ERX708976	ERR765703	16S rRNA	V1-V3	AGTACGCTAT	AGAGTTTGATCMTGGCTCAG	GWATTACCGCGGCKGCTG	2011-06-22	39.55	-121.04	USA	0.1	1135	11.2	55	Mesic Ultic Haploxeralfs	Ponderosa pine, sugar pine, white fir, giant sequoia	Csa, Mediterranean hot summer	OM1	C0	0	O horizon	58.0	39.0	1.3	4.4	NA	30.4
BR054B	BR054	PP_CA_	California	BR	Forest Soil	Whole Community DNA	PCR	Amplicon	pyrotag library	454 GS FLX Titanium	European Nucleotide Archive	https://www.ebi.ac.uk/ena/data/search?query=PRJEB8599	ERS662730	SAMEA3261734	ERX708977	ERR765704	16S rRNA	V1-V3	TCGATCACGT	AGAGTTTGATCMTGGCTCAG	GWATTACCGCGGCKGCTG	2011-06-22	39.55	-121.04	USA	0.3	1135	11.2	55	Mesic Ultic Haploxeralfs	Ponderosa pine, sugar pine, white fir, giant sequoia	Csa, Mediterranean hot summer	OM1	C0	0	A horizon	31.7	6.2	0.2	5.3	NA	24.9
BR055B	BR055	PP_CA_	California	BR	Forest Soil	Whole Community DNA	PCR	Amplicon	pyrotag library	454 GS FLX Titanium	European Nucleotide Archive	https://www.ebi.ac.uk/ena/data/search?query=PRJEB8599	ERS662731	SAMEA3261735	ERX708978	ERR765705	16S rRNA	V1-V3	TGATACGTCT	AGAGTTTGATCMTGGCTCAG	GWATTACCGCGGCKGCTG	2011-06-22	39.55	-121.04	USA	0.1	1135	11.2	55	Mesic Ultic Haploxeralfs	Ponderosa pine, sugar pine, white fir, giant sequoia	Csa, Mediterranean hot summer	OM2	C0	0	O horizon	50.0	26.4	1.1	5.7	NA	23.6
BR056B	BR056	PP_CA_	California	BR	Forest Soil	Whole Community DNA	PCR	Amplicon	pyrotag library	454 GS FLX Titanium	European Nucleotide Archive	https://www.ebi.ac.uk/ena/data/search?query=PRJEB8599	ERS662732	SAMEA3261736	ERX708979	ERR765706	16S rRNA	V1-V3	ATATCGCGAG	AGAGTTTGATCMTGGCTCAG	GWATTACCGCGGCKGCTG	2011-06-22	39.55	-121.04	USA	0.3	1135	11.2	55	Mesic Ultic Haploxeralfs	Ponderosa pine, sugar pine, white fir, giant sequoia	Csa, Mediterranean hot summer	OM2	C0	0	A horizon	35.6	5.7	0.3	6.1	NA	20.9
BR057B	BR057	PP_CA_	California	BR	Forest Soil	Whole Community DNA	PCR	Amplicon	pyrotag library	454 GS FLX Titanium	European Nucleotide Archive	https://www.ebi.ac.uk/ena/data/search?query=PRJEB8599	ERS662733	SAMEA3261737	ERX708980	ERR765707	16S rRNA	V1-V3	ACGCTCGACA	AGAGTTTGATCMTGGCTCAG	GWATTACCGCGGCKGCTG	2011-06-22	39.55	-121.04	USA	0.1	1135	11.2	55	Mesic Ultic Haploxeralfs	Ponderosa pine, sugar pine, white fir, giant sequoia	Csa, Mediterranean hot summer	OM2	C0	0	O horizon	50.6	35.1	1.2	5.0	NA	28.1
BR058B	BR058	PP_CA_	California	BR	Forest Soil	Whole Community DNA	PCR	Amplicon	pyrotag library	454 GS FLX Titanium	European Nucleotide Archive	https://www.ebi.ac.uk/ena/data/search?query=PRJEB8599	ERS662734	SAMEA3261738	ERX708981	ERR765708	16S rRNA	V1-V3	CATAGTAGTG	AGAGTTTGATCMTGGCTCAG	GWATTACCGCGGCKGCTG	2011-06-22	39.55	-121.04	USA	0.3	1135	11.2	55	Mesic Ultic Haploxeralfs	Ponderosa pine, sugar pine, white fir, giant sequoia	Csa, Mediterranean hot summer	OM2	C0	0	A horizon	35.4	5.7	0.3	6.3	NA	20.9
BR059B	BR059	PP_CA_	California	BR	Forest Soil	Whole Community DNA	PCR	Amplicon	pyrotag library	454 GS FLX Titanium	European Nucleotide Archive	https://www.ebi.ac.uk/ena/data/search?query=PRJEB8599	ERS662735	SAMEA3261739	ERX708982	ERR765709	16S rRNA	V1-V3	TACGCTGTCT	AGAGTTTGATCMTGGCTCAG	GWATTACCGCGGCKGCTG	2011-06-22	39.55	-121.04	USA	0.1	1135	11.2	55	Mesic Ultic Haploxeralfs	Ponderosa pine, sugar pine, white fir, giant sequoia	Csa, Mediterranean hot summer	OM2	C0	0	O horizon	45.5	27.8	1.2	5.9	NA	24.0
BR060B	BR060	PP_CA_	California	BR	Forest Soil	Whole Community DNA	PCR	Amplicon	pyrotag library	454 GS FLX Titanium	European Nucleotide Archive	https://www.ebi.ac.uk/ena/data/search?query=PRJEB8599	ERS662736	SAMEA3261740	ERX708983	ERR765710	16S rRNA	V1-V3	TCTCTATGCG	AGAGTTTGATCMTGGCTCAG	GWATTACCGCGGCKGCTG	2011-06-22	39.55	-121.04	USA	0.3	1135	11.2	55	Mesic Ultic Haploxeralfs	Ponderosa pine, sugar pine, white fir, giant sequoia	Csa, Mediterranean hot summer	OM2	C0	0	A horizon	37.0	5.7	0.3	6.1	NA	20.9
BR061B	BR061	PP_CA_	California	BR	Forest Soil	Whole Community DNA	PCR	Amplicon	pyrotag library	454 GS FLX Titanium	European Nucleotide Archive	https://www.ebi.ac.uk/ena/data/search?query=PRJEB8599	ERS662737	SAMEA3261741	ERX708984	ERR765711	16S rRNA	V1-V3	TGACGTATGT	AGAGTTTGATCMTGGCTCAG	GWATTACCGCGGCKGCTG	2011-06-22	39.55	-121.04	USA	0.1	1135	11.2	55	Mesic Ultic Haploxeralfs	Ponderosa pine, sugar pine, white fir, giant sequoia	Csa, Mediterranean hot summer	OM3	C0	0	O horizon	40.0	16.4	0.6	5.8	NA	27.1
BR062B	BR062	PP_CA_	California	BR	Forest Soil	Whole Community DNA	PCR	Amplicon	pyrotag library	454 GS FLX Titanium	European Nucleotide Archive	https://www.ebi.ac.uk/ena/data/search?query=PRJEB8599	ERS662738	SAMEA3261742	ERX708985	ERR765712	16S rRNA	V1-V3	CTCGCGTGTC	AGAGTTTGATCMTGGCTCAG	GWATTACCGCGGCKGCTG	2011-06-22	39.55	-121.04	USA	0.3	1135	11.2	55	Mesic Ultic Haploxeralfs	Ponderosa pine, sugar pine, white fir, giant sequoia	Csa, Mediterranean hot summer	OM3	C0	0	A horizon	33.6	6.7	0.3	5.5	NA	20.7
BR063B	BR063	PP_CA_	California	BR	Forest Soil	Whole Community DNA	PCR	Amplicon	pyrotag library	454 GS FLX Titanium	European Nucleotide Archive	https://www.ebi.ac.uk/ena/data/search?query=PRJEB8599	ERS662739	SAMEA3261743	ERX708986	ERR765713	16S rRNA	V1-V3	CGACGTGACT	AGAGTTTGATCMTGGCTCAG	GWATTACCGCGGCKGCTG	2011-06-22	39.55	-121.04	USA	0.1	1135	11.2	55	Mesic Ultic Haploxeralfs	Ponderosa pine, sugar pine, white fir, giant sequoia	Csa, Mediterranean hot summer	OM3	C0	0	O horizon	50.0	19.2	0.7	4.8	NA	26.8
BR064B	BR064	PP_CA_	California	BR	Forest Soil	Whole Community DNA	PCR	Amplicon	pyrotag library	454 GS FLX Titanium	European Nucleotide Archive	https://www.ebi.ac.uk/ena/data/search?query=PRJEB8599	ERS662740	SAMEA3261744	ERX708987	ERR765714	16S rRNA	V1-V3	ATCAGACACG	AGAGTTTGATCMTGGCTCAG	GWATTACCGCGGCKGCTG	2011-06-22	39.55	-121.04	USA	0.3	1135	11.2	55	Mesic Ultic Haploxeralfs	Ponderosa pine, sugar pine, white fir, giant sequoia	Csa, Mediterranean hot summer	OM3	C0	0	A horizon	33.4	5.9	0.3	5.4	NA	19.1
BR065B	BR065	PP_CA_	California	BR	Forest Soil	Whole Community DNA	PCR	Amplicon	pyrotag library	454 GS FLX Titanium	European Nucleotide Archive	https://www.ebi.ac.uk/ena/data/search?query=PRJEB8599	ERS662741	SAMEA3261745	ERX708988	ERR765715	16S rRNA	V1-V3	AGCGTCGTCT	AGAGTTTGATCMTGGCTCAG	GWATTACCGCGGCKGCTG	2011-06-22	39.55	-121.04	USA	0.1	1135	11.2	55	Mesic Ultic Haploxeralfs	Ponderosa pine, sugar pine, white fir, giant sequoia	Csa, Mediterranean hot summer	OM3	C0	0	O horizon	50.8	27.1	1.1	5.6	NA	25.1
BR066B	BR066	PP_CA_	California	BR	Forest Soil	Whole Community DNA	PCR	Amplicon	pyrotag library	454 GS FLX Titanium	European Nucleotide Archive	https://www.ebi.ac.uk/ena/data/search?query=PRJEB8599	ERS662742	SAMEA3261746	ERX708989	ERR765716	16S rRNA	V1-V3	AGTACGCTAT	AGAGTTTGATCMTGGCTCAG	GWATTACCGCGGCKGCTG	2011-06-22	39.55	-121.04	USA	0.3	1135	11.2	55	Mesic Ultic Haploxeralfs	Ponderosa pine, sugar pine, white fir, giant sequoia	Csa, Mediterranean hot summer	OM3	C0	0	A horizon	34.7	6.1	0.3	5.8	NA	19.1
BR067B	BR067	PP_CA_	California	BR	Forest Soil	Whole Community DNA	PCR	Amplicon	pyrotag library	454 GS FLX Titanium	European Nucleotide Archive	https://www.ebi.ac.uk/ena/data/search?query=PRJEB8599	ERS662743	SAMEA3261747	ERX708990	ERR765717	16S rRNA	V1-V3	TCTCTATGCG	AGAGTTTGATCMTGGCTCAG	GWATTACCGCGGCKGCTG	2011-06-22	39.55	-121.04	USA	0.1	1135	11.2	55	Mesic Ultic Haploxeralfs	Ponderosa pine, sugar pine, white fir, giant sequoia	Csa, Mediterranean hot summer	REF	REF	0	O horizon	60.7	39.2	1.2	5.0	NA	31.6
BR068B	BR068	PP_CA_	California	BR	Forest Soil	Whole Community DNA	PCR	Amplicon	pyrotag library	454 GS FLX Titanium	European Nucleotide Archive	https://www.ebi.ac.uk/ena/data/search?query=PRJEB8599	ERS662744	SAMEA3261748	ERX708991	ERR765718	16S rRNA	V1-V3	ACGCGAGTAT	AGAGTTTGATCMTGGCTCAG	GWATTACCGCGGCKGCTG	2011-06-22	39.55	-121.04	USA	0.3	1135	11.2	55	Mesic Ultic Haploxeralfs	Ponderosa pine, sugar pine, white fir, giant sequoia	Csa, Mediterranean hot summer	REF	REF	0	A horizon	27.5	6.7	0.3	6.0	NA	23.4
BR069B	BR069	PP_CA_	California	BR	Forest Soil	Whole Community DNA	PCR	Amplicon	pyrotag library	454 GS FLX Titanium	European Nucleotide Archive	https://www.ebi.ac.uk/ena/data/search?query=PRJEB8599	ERS662745	SAMEA3261749	ERX708992	ERR765719	16S rRNA	V1-V3	TACGCTGTCT	AGAGTTTGATCMTGGCTCAG	GWATTACCGCGGCKGCTG	2011-06-22	39.55	-121.04	USA	0.1	1135	11.2	55	Mesic Ultic Haploxeralfs	Ponderosa pine, sugar pine, white fir, giant sequoia	Csa, Mediterranean hot summer	REF	REF	0	O horizon	47.8	33.3	1.4	6.3	NA	23.7
BR070B	BR070	PP_CA_	California	BR	Forest Soil	Whole Community DNA	PCR	Amplicon	pyrotag library	454 GS FLX Titanium	European Nucleotide Archive	https://www.ebi.ac.uk/ena/data/search?query=PRJEB8599	ERS662746	SAMEA3261750	ERX708993	ERR765720	16S rRNA	V1-V3	ACTACTATGT	AGAGTTTGATCMTGGCTCAG	GWATTACCGCGGCKGCTG	2011-06-22	39.55	-121.04	USA	0.3	1135	11.2	55	Mesic Ultic Haploxeralfs	Ponderosa pine, sugar pine, white fir, giant sequoia	Csa, Mediterranean hot summer	REF	REF	0	A horizon	35.5	8.0	0.4	6.7	NA	20.0
BR071B	BR071	PP_CA_	California	BR	Forest Soil	Whole Community DNA	PCR	Amplicon	pyrotag library	454 GS FLX Titanium	European Nucleotide Archive	https://www.ebi.ac.uk/ena/data/search?query=PRJEB8599	ERS662747	SAMEA3261751	ERX708994	ERR765721	16S rRNA	V1-V3	ACTAGCAGTA	AGAGTTTGATCMTGGCTCAG	GWATTACCGCGGCKGCTG	2011-06-22	39.55	-121.04	USA	0.1	1135	11.2	55	Mesic Ultic Haploxeralfs	Ponderosa pine, sugar pine, white fir, giant sequoia	Csa, Mediterranean hot summer	REF	REF	0	O horizon	58.9	42.8	1.4	5.6	NA	30.7
BR072B	BR072	PP_CA_	California	BR	Forest Soil	Whole Community DNA	PCR	Amplicon	pyrotag library	454 GS FLX Titanium	European Nucleotide Archive	https://www.ebi.ac.uk/ena/data/search?query=PRJEB8599	ERS662748	SAMEA3261752	ERX708995	ERR765722	16S rRNA	V1-V3	ACGCTCGACA	AGAGTTTGATCMTGGCTCAG	GWATTACCGCGGCKGCTG	2011-06-22	39.55	-121.04	USA	0.3	1135	11.2	55	Mesic Ultic Haploxeralfs	Ponderosa pine, sugar pine, white fir, giant sequoia	Csa, Mediterranean hot summer	REF	REF	0	A horizon	31.1	7.0	0.3	6.3	NA	22.4
JE085B	JE085	JP_ON_	Ontario	JE	Forest Soil	Whole Community DNA	PCR	Amplicon	pyrotag library	454 GS FLX Titanium	European Nucleotide Archive	https://www.ebi.ac.uk/ena/data/search?query=PRJEB8599	ERS662749	SAMEA3261753	ERX708996	ERR765723	16S rRNA	V1-V3	CGAGAGATAC	AGAGTTTGATCMTGGCTCAG	GWATTACCGCGGCKGCTG	2011-08-03	46.75	-82.25	Canada	0.1	490	2.8	242	NA	Jack Pine, Balsam fir, White birch	Dfb, Humid Continental cool summer	OM1	C0	1	O horizon	33.7	41.5	1.3	3.7	5.7	34.6
JE086B	JE086	JP_ON_	Ontario	JE	Forest Soil	Whole Community DNA	PCR	Amplicon	pyrotag library	454 GS FLX Titanium	European Nucleotide Archive	https://www.ebi.ac.uk/ena/data/search?query=PRJEB8599	ERS662750	SAMEA3261754	ERX708997	ERR765724	16S rRNA	V1-V3	TCTAGCGACT	AGAGTTTGATCMTGGCTCAG	GWATTACCGCGGCKGCTG	2011-08-03	46.75	-82.25	Canada	0.3	490	2.8	242	NA	Jack Pine, Balsam fir, White birch	Dfb, Humid Continental cool summer	OM1	C0	1	A horizon	6.1	2.6	0.2	5.0	5.7	15.9
JE087B	JE087	JP_ON_	Ontario	JE	Forest Soil	Whole Community DNA	PCR	Amplicon	pyrotag library	454 GS FLX Titanium	European Nucleotide Archive	https://www.ebi.ac.uk/ena/data/search?query=PRJEB8599	ERS662751	SAMEA3261755	ERX708998	ERR765725	16S rRNA	V1-V3	TCGCACTAGT	AGAGTTTGATCMTGGCTCAG	GWATTACCGCGGCKGCTG	2011-08-03	46.75	-82.25	Canada	0.1	490	2.8	242	NA	Jack Pine, Balsam fir, White birch	Dfb, Humid Continental cool summer	OM1	C0	0	O horizon	33.7	41.5	1.3	3.7	5.7	34.6
JE088B	JE088	JP_ON_	Ontario	JE	Forest Soil	Whole Community DNA	PCR	Amplicon	pyrotag library	454 GS FLX Titanium	European Nucleotide Archive	https://www.ebi.ac.uk/ena/data/search?query=PRJEB8599	ERS662752	SAMEA3261756	ERX708999	ERR765726	16S rRNA	V1-V3	CGAGAGATAC	AGAGTTTGATCMTGGCTCAG	GWATTACCGCGGCKGCTG	2011-08-03	46.75	-82.25	Canada	0.3	490	2.8	242	NA	Jack Pine, Balsam fir, White birch	Dfb, Humid Continental cool summer	OM1	C0	0	A horizon	9.6	2.6	0.2	5.0	5.7	15.9
JE090B	JE090	JP_ON_	Ontario	JE	Forest Soil	Whole Community DNA	PCR	Amplicon	pyrotag library	454 GS FLX Titanium	European Nucleotide Archive	https://www.ebi.ac.uk/ena/data/search?query=PRJEB8599	ERS662753	SAMEA3261757	ERX709000	ERR765727	16S rRNA	V1-V3	TACAGATCGT	AGAGTTTGATCMTGGCTCAG	GWATTACCGCGGCKGCTG	2011-08-03	46.75	-82.25	Canada	0.3	490	2.8	242	NA	Jack Pine, Balsam fir, White birch	Dfb, Humid Continental cool summer	OM3	C0	1	A horizon	8.0	3.0	0.2	5.0	5.7	16.4
JE092B	JE092	JP_ON_	Ontario	JE	Forest Soil	Whole Community DNA	PCR	Amplicon	pyrotag library	454 GS FLX Titanium	European Nucleotide Archive	https://www.ebi.ac.uk/ena/data/search?query=PRJEB8599	ERS662754	SAMEA3261758	ERX709001	ERR765728	16S rRNA	V1-V3	TACGCTGTCT	AGAGTTTGATCMTGGCTCAG	GWATTACCGCGGCKGCTG	2011-08-03	46.75	-82.25	Canada	0.3	490	2.8	242	NA	Jack Pine, Balsam fir, White birch	Dfb, Humid Continental cool summer	OM3	C0	0	A horizon	8.0	3.0	0.2	5.0	5.7	16.4
JE093B	JE093	JP_ON_	Ontario	JE	Forest Soil	Whole Community DNA	PCR	Amplicon	pyrotag library	454 GS FLX Titanium	European Nucleotide Archive	https://www.ebi.ac.uk/ena/data/search?query=PRJEB8599	ERS662755	SAMEA3261759	ERX709002	ERR765729	16S rRNA	V1-V3	ATACGACGTA	AGAGTTTGATCMTGGCTCAG	GWATTACCGCGGCKGCTG	2011-08-03	46.75	-82.25	Canada	0.1	490	2.8	242	NA	Jack Pine, Balsam fir, White birch	Dfb, Humid Continental cool summer	OM2	C0	1	O horizon	20.0	40.5	1.3	3.7	5.7	32.3
JE094B	JE094	JP_ON_	Ontario	JE	Forest Soil	Whole Community DNA	PCR	Amplicon	pyrotag library	454 GS FLX Titanium	European Nucleotide Archive	https://www.ebi.ac.uk/ena/data/search?query=PRJEB8599	ERS662756	SAMEA3261760	ERX709003	ERR765730	16S rRNA	V1-V3	ACGCGATCGA	AGAGTTTGATCMTGGCTCAG	GWATTACCGCGGCKGCTG	2011-08-03	46.75	-82.25	Canada	0.3	490	2.8	242	NA	Jack Pine, Balsam fir, White birch	Dfb, Humid Continental cool summer	OM2	C0	1	A horizon	12.9	2.9	0.2	5.0	5.7	15.9
JE095B	JE095	JP_ON_	Ontario	JE	Forest Soil	Whole Community DNA	PCR	Amplicon	pyrotag library	454 GS FLX Titanium	European Nucleotide Archive	https://www.ebi.ac.uk/ena/data/search?query=PRJEB8599	ERS662757	SAMEA3261761	ERX709004	ERR765731	16S rRNA	V1-V3	ACTGTACAGT	AGAGTTTGATCMTGGCTCAG	GWATTACCGCGGCKGCTG	2011-08-03	46.75	-82.25	Canada	0.1	490	2.8	242	NA	Jack Pine, Balsam fir, White birch	Dfb, Humid Continental cool summer	OM2	C0	0	O horizon	32.0	40.5	1.3	3.7	5.7	32.3
JE096B	JE096	JP_ON_	Ontario	JE	Forest Soil	Whole Community DNA	PCR	Amplicon	pyrotag library	454 GS FLX Titanium	European Nucleotide Archive	https://www.ebi.ac.uk/ena/data/search?query=PRJEB8599	ERS662758	SAMEA3261762	ERX709005	ERR765732	16S rRNA	V1-V3	AGCACTGTAG	AGAGTTTGATCMTGGCTCAG	GWATTACCGCGGCKGCTG	2011-08-03	46.75	-82.25	Canada	0.3	490	2.8	242	NA	Jack Pine, Balsam fir, White birch	Dfb, Humid Continental cool summer	OM2	C0	0	A horizon	12.5	2.9	0.2	5.0	5.7	15.9
JE098B	JE098	JP_ON_	Ontario	JE	Forest Soil	Whole Community DNA	PCR	Amplicon	pyrotag library	454 GS FLX Titanium	European Nucleotide Archive	https://www.ebi.ac.uk/ena/data/search?query=PRJEB8599	ERS662759	SAMEA3261763	ERX709006	ERR765733	16S rRNA	V1-V3	AGCGTCGTCT	AGAGTTTGATCMTGGCTCAG	GWATTACCGCGGCKGCTG	2011-08-03	46.75	-82.25	Canada	0.3	490	2.8	242	NA	Jack Pine, Balsam fir, White birch	Dfb, Humid Continental cool summer	OM3	C0	1	A horizon	10.9	1.9	0.1	5.2	5.7	13.6
JE100B	JE100	JP_ON_	Ontario	JE	Forest Soil	Whole Community DNA	PCR	Amplicon	pyrotag library	454 GS FLX Titanium	European Nucleotide Archive	https://www.ebi.ac.uk/ena/data/search?query=PRJEB8599	ERS662760	SAMEA3261764	ERX709007	ERR765734	16S rRNA	V1-V3	CTCGCGTGTC	AGAGTTTGATCMTGGCTCAG	GWATTACCGCGGCKGCTG	2011-08-03	46.75	-82.25	Canada	0.3	490	2.8	242	NA	Jack Pine, Balsam fir, White birch	Dfb, Humid Continental cool summer	OM3	C0	0	A horizon	10.6	1.9	0.1	5.2	5.7	13.6
JE101B	JE101	JP_ON_	Ontario	JE	Forest Soil	Whole Community DNA	PCR	Amplicon	pyrotag library	454 GS FLX Titanium	European Nucleotide Archive	https://www.ebi.ac.uk/ena/data/search?query=PRJEB8599	ERS662761	SAMEA3261765	ERX709008	ERR765735	16S rRNA	V1-V3	TCTACGTAGC	AGAGTTTGATCMTGGCTCAG	GWATTACCGCGGCKGCTG	2011-08-03	46.75	-82.25	Canada	0.1	490	2.8	242	NA	Jack Pine, Balsam fir, White birch	Dfb, Humid Continental cool summer	OM2	C0	1	O horizon	31.1	38.5	1.2	3.6	5.7	32.7
JE102B	JE102	JP_ON_	Ontario	JE	Forest Soil	Whole Community DNA	PCR	Amplicon	pyrotag library	454 GS FLX Titanium	European Nucleotide Archive	https://www.ebi.ac.uk/ena/data/search?query=PRJEB8599	ERS662762	SAMEA3261766	ERX709009	ERR765736	16S rRNA	V1-V3	CGACGTGACT	AGAGTTTGATCMTGGCTCAG	GWATTACCGCGGCKGCTG	2011-08-03	46.75	-82.25	Canada	0.3	490	2.8	242	NA	Jack Pine, Balsam fir, White birch	Dfb, Humid Continental cool summer	OM2	C0	1	A horizon	7.0	2.1	0.2	5.1	5.7	13.5
JE103B	JE103	JP_ON_	Ontario	JE	Forest Soil	Whole Community DNA	PCR	Amplicon	pyrotag library	454 GS FLX Titanium	European Nucleotide Archive	https://www.ebi.ac.uk/ena/data/search?query=PRJEB8599	ERS662763	SAMEA3261767	ERX709010	ERR765737	16S rRNA	V1-V3	ACGAGTGCGT	AGAGTTTGATCMTGGCTCAG	GWATTACCGCGGCKGCTG	2011-08-03	46.75	-82.25	Canada	0.1	490	2.8	242	NA	Jack Pine, Balsam fir, White birch	Dfb, Humid Continental cool summer	OM2	C0	0	O horizon	24.5	38.5	1.2	3.6	5.7	32.7
JE104B	JE104	JP_ON_	Ontario	JE	Forest Soil	Whole Community DNA	PCR	Amplicon	pyrotag library	454 GS FLX Titanium	European Nucleotide Archive	https://www.ebi.ac.uk/ena/data/search?query=PRJEB8599	ERS662764	SAMEA3261768	ERX709011	ERR765738	16S rRNA	V1-V3	CATAGTAGTG	AGAGTTTGATCMTGGCTCAG	GWATTACCGCGGCKGCTG	2011-08-03	46.75	-82.25	Canada	0.3	490	2.8	242	NA	Jack Pine, Balsam fir, White birch	Dfb, Humid Continental cool summer	OM2	C0	0	A horizon	12.9	2.1	0.2	5.1	5.7	13.5
JE105B	JE105	JP_ON_	Ontario	JE	Forest Soil	Whole Community DNA	PCR	Amplicon	pyrotag library	454 GS FLX Titanium	European Nucleotide Archive	https://www.ebi.ac.uk/ena/data/search?query=PRJEB8599	ERS662765	SAMEA3261769	ERX709012	ERR765739	16S rRNA	V1-V3	TACAGATCGT	AGAGTTTGATCMTGGCTCAG	GWATTACCGCGGCKGCTG	2011-08-03	46.75	-82.25	Canada	0.1	490	2.8	242	NA	Jack Pine, Balsam fir, White birch	Dfb, Humid Continental cool summer	OM1	C0	1	O horizon	27.2	41.4	1.3	3.8	5.7	33.8
JE106B	JE106	JP_ON_	Ontario	JE	Forest Soil	Whole Community DNA	PCR	Amplicon	pyrotag library	454 GS FLX Titanium	European Nucleotide Archive	https://www.ebi.ac.uk/ena/data/search?query=PRJEB8599	ERS662766	SAMEA3261770	ERX709013	ERR765740	16S rRNA	V1-V3	ATATCGCGAG	AGAGTTTGATCMTGGCTCAG	GWATTACCGCGGCKGCTG	2011-08-03	46.75	-82.25	Canada	0.3	490	2.8	242	NA	Jack Pine, Balsam fir, White birch	Dfb, Humid Continental cool summer	OM1	C0	1	A horizon	7.1	2.4	0.2	5.1	5.7	13.9
JE107B	JE107	JP_ON_	Ontario	JE	Forest Soil	Whole Community DNA	PCR	Amplicon	pyrotag library	454 GS FLX Titanium	European Nucleotide Archive	https://www.ebi.ac.uk/ena/data/search?query=PRJEB8599	ERS662767	SAMEA3261771	ERX709014	ERR765741	16S rRNA	V1-V3	ATAGAGTACT	AGAGTTTGATCMTGGCTCAG	GWATTACCGCGGCKGCTG	2011-08-03	46.75	-82.25	Canada	0.1	490	2.8	242	NA	Jack Pine, Balsam fir, White birch	Dfb, Humid Continental cool summer	OM1	C0	0	O horizon	24.0	41.4	1.3	3.8	5.7	33.8
JE108B	JE108	JP_ON_	Ontario	JE	Forest Soil	Whole Community DNA	PCR	Amplicon	pyrotag library	454 GS FLX Titanium	European Nucleotide Archive	https://www.ebi.ac.uk/ena/data/search?query=PRJEB8599	ERS662768	SAMEA3261772	ERX709015	ERR765742	16S rRNA	V1-V3	TCTCTATGCG	AGAGTTTGATCMTGGCTCAG	GWATTACCGCGGCKGCTG	2011-08-03	46.75	-82.25	Canada	0.3	490	2.8	242	NA	Jack Pine, Balsam fir, White birch	Dfb, Humid Continental cool summer	OM1	C0	0	A horizon	12.0	2.4	0.2	5.1	5.7	13.9
JE110B	JE110	JP_ON_	Ontario	JE	Forest Soil	Whole Community DNA	PCR	Amplicon	pyrotag library	454 GS FLX Titanium	European Nucleotide Archive	https://www.ebi.ac.uk/ena/data/search?query=PRJEB8599	ERS662769	SAMEA3261773	ERX709016	ERR765743	16S rRNA	V1-V3	CAGTAGACGT	AGAGTTTGATCMTGGCTCAG	GWATTACCGCGGCKGCTG	2011-08-03	46.75	-82.25	Canada	0.3	490	2.8	242	NA	Jack Pine, Balsam fir, White birch	Dfb, Humid Continental cool summer	OM3	C0	1	A horizon	13.9	1.9	0.1	5.2	5.7	13.9
JE112B	JE112	JP_ON_	Ontario	JE	Forest Soil	Whole Community DNA	PCR	Amplicon	pyrotag library	454 GS FLX Titanium	European Nucleotide Archive	https://www.ebi.ac.uk/ena/data/search?query=PRJEB8599	ERS662770	SAMEA3261774	ERX709017	ERR765744	16S rRNA	V1-V3	ACGCGATCGA	AGAGTTTGATCMTGGCTCAG	GWATTACCGCGGCKGCTG	2011-08-03	46.75	-82.25	Canada	0.3	490	2.8	242	NA	Jack Pine, Balsam fir, White birch	Dfb, Humid Continental cool summer	OM3	C0	0	A horizon	14.0	1.9	0.1	5.2	5.7	13.9
JE113B	JE113	JP_ON_	Ontario	JE	Forest Soil	Whole Community DNA	PCR	Amplicon	pyrotag library	454 GS FLX Titanium	European Nucleotide Archive	https://www.ebi.ac.uk/ena/data/search?query=PRJEB8599	ERS662771	SAMEA3261775	ERX709018	ERR765745	16S rRNA	V1-V3	TCACGTACTA	AGAGTTTGATCMTGGCTCAG	GWATTACCGCGGCKGCTG	2011-08-03	46.75	-82.25	Canada	0.1	490	2.8	242	NA	Jack Pine, Balsam fir, White birch	Dfb, Humid Continental cool summer	OM2	C0	1	O horizon	49.5	41.6	1.4	3.7	5.7	31.6
JE114B	JE114	JP_ON_	Ontario	JE	Forest Soil	Whole Community DNA	PCR	Amplicon	pyrotag library	454 GS FLX Titanium	European Nucleotide Archive	https://www.ebi.ac.uk/ena/data/search?query=PRJEB8599	ERS662772	SAMEA3261776	ERX709019	ERR765746	16S rRNA	V1-V3	TACACACACT	AGAGTTTGATCMTGGCTCAG	GWATTACCGCGGCKGCTG	2011-08-03	46.75	-82.25	Canada	0.3	490	2.8	242	NA	Jack Pine, Balsam fir, White birch	Dfb, Humid Continental cool summer	OM2	C0	1	A horizon	11.0	2.6	0.2	5.0	5.7	16.1
JE115B	JE115	JP_ON_	Ontario	JE	Forest Soil	Whole Community DNA	PCR	Amplicon	pyrotag library	454 GS FLX Titanium	European Nucleotide Archive	https://www.ebi.ac.uk/ena/data/search?query=PRJEB8599	ERS662773	SAMEA3261777	ERX709020	ERR765747	16S rRNA	V1-V3	AGCGTCGTCT	AGAGTTTGATCMTGGCTCAG	GWATTACCGCGGCKGCTG	2011-08-03	46.75	-82.25	Canada	0.1	490	2.8	242	NA	Jack Pine, Balsam fir, White birch	Dfb, Humid Continental cool summer	OM2	C0	0	O horizon	55.9	41.6	1.4	3.7	5.7	31.6
JE116B	JE116	JP_ON_	Ontario	JE	Forest Soil	Whole Community DNA	PCR	Amplicon	pyrotag library	454 GS FLX Titanium	European Nucleotide Archive	https://www.ebi.ac.uk/ena/data/search?query=PRJEB8599	ERS662774	SAMEA3261778	ERX709021	ERR765748	16S rRNA	V1-V3	ACGCGATCGA	AGAGTTTGATCMTGGCTCAG	GWATTACCGCGGCKGCTG	2011-08-03	46.75	-82.25	Canada	0.3	490	2.8	242	NA	Jack Pine, Balsam fir, White birch	Dfb, Humid Continental cool summer	OM2	C0	0	A horizon	16.2	2.6	0.2	5.0	5.7	16.1
JE117B	JE117	JP_ON_	Ontario	JE	Forest Soil	Whole Community DNA	PCR	Amplicon	pyrotag library	454 GS FLX Titanium	European Nucleotide Archive	https://www.ebi.ac.uk/ena/data/search?query=PRJEB8599	ERS662775	SAMEA3261779	ERX709022	ERR765749	16S rRNA	V1-V3	ACTACTATGT	AGAGTTTGATCMTGGCTCAG	GWATTACCGCGGCKGCTG	2011-08-03	46.75	-82.25	Canada	0.1	490	2.8	242	NA	Jack Pine, Balsam fir, White birch	Dfb, Humid Continental cool summer	OM1	C0	1	O horizon	42.1	39.6	1.3	3.8	5.7	33.5
JE118B	JE118	JP_ON_	Ontario	JE	Forest Soil	Whole Community DNA	PCR	Amplicon	pyrotag library	454 GS FLX Titanium	European Nucleotide Archive	https://www.ebi.ac.uk/ena/data/search?query=PRJEB8599	ERS662776	SAMEA3261780	ERX709023	ERR765750	16S rRNA	V1-V3	CGACGTGACT	AGAGTTTGATCMTGGCTCAG	GWATTACCGCGGCKGCTG	2011-08-03	46.75	-82.25	Canada	0.3	490	2.8	242	NA	Jack Pine, Balsam fir, White birch	Dfb, Humid Continental cool summer	OM1	C0	1	A horizon	21.4	2.5	0.2	4.9	5.7	15.3
JE119B	JE119	JP_ON_	Ontario	JE	Forest Soil	Whole Community DNA	PCR	Amplicon	pyrotag library	454 GS FLX Titanium	European Nucleotide Archive	https://www.ebi.ac.uk/ena/data/search?query=PRJEB8599	ERS662777	SAMEA3261781	ERX709024	ERR765751	16S rRNA	V1-V3	TACACACACT	AGAGTTTGATCMTGGCTCAG	GWATTACCGCGGCKGCTG	2011-08-03	46.75	-82.25	Canada	0.1	490	2.8	242	NA	Jack Pine, Balsam fir, White birch	Dfb, Humid Continental cool summer	OM1	C0	0	O horizon	47.6	39.6	1.3	3.8	5.7	33.5
JE120B	JE120	JP_ON_	Ontario	JE	Forest Soil	Whole Community DNA	PCR	Amplicon	pyrotag library	454 GS FLX Titanium	European Nucleotide Archive	https://www.ebi.ac.uk/ena/data/search?query=PRJEB8599	ERS662778	SAMEA3261782	ERX709025	ERR765752	16S rRNA	V1-V3	CGTCTAGTAC	AGAGTTTGATCMTGGCTCAG	GWATTACCGCGGCKGCTG	2011-08-03	46.75	-82.25	Canada	0.3	490	2.8	242	NA	Jack Pine, Balsam fir, White birch	Dfb, Humid Continental cool summer	OM1	C0	0	A horizon	9.8	2.5	0.2	4.9	5.7	15.3
JE121B	JE121	JP_ON_	Ontario	JE	Forest Soil	Whole Community DNA	PCR	Amplicon	pyrotag library	454 GS FLX Titanium	European Nucleotide Archive	https://www.ebi.ac.uk/ena/data/search?query=PRJEB8599	ERS662779	SAMEA3261783	ERX709026	ERR765753	16S rRNA	V1-V3	ATCAGACACG	AGAGTTTGATCMTGGCTCAG	GWATTACCGCGGCKGCTG	2011-08-03	46.75	-82.25	Canada	0.1	490	2.8	242	NA	Jack Pine, Balsam fir, White birch	Dfb, Humid Continental cool summer	REF	REF	0	O horizon	68.9	43.2	1.4	3.6	5.7	30.8
JE122B	JE122	JP_ON_	Ontario	JE	Forest Soil	Whole Community DNA	PCR	Amplicon	pyrotag library	454 GS FLX Titanium	European Nucleotide Archive	https://www.ebi.ac.uk/ena/data/search?query=PRJEB8599	ERS662780	SAMEA3261784	ERX709027	ERR765754	16S rRNA	V1-V3	AGCACTGTAG	AGAGTTTGATCMTGGCTCAG	GWATTACCGCGGCKGCTG	2011-08-03	46.75	-82.25	Canada	0.3	490	2.8	242	NA	Jack Pine, Balsam fir, White birch	Dfb, Humid Continental cool summer	REF	REF	0	A horizon	14.6	2.4	0.2	4.9	5.7	16.4
JE123B	JE123	JP_ON_	Ontario	JE	Forest Soil	Whole Community DNA	PCR	Amplicon	pyrotag library	454 GS FLX Titanium	European Nucleotide Archive	https://www.ebi.ac.uk/ena/data/search?query=PRJEB8599	ERS662781	SAMEA3261785	ERX709028	ERR765755	16S rRNA	V1-V3	CGTGTCTCTA	AGAGTTTGATCMTGGCTCAG	GWATTACCGCGGCKGCTG	2011-08-03	46.75	-82.25	Canada	0.1	490	2.8	242	NA	Jack Pine, Balsam fir, White birch	Dfb, Humid Continental cool summer	REF	REF	0	O horizon	47.1	38.1	1.2	3.7	5.7	30.8
JE124B	JE124	JP_ON_	Ontario	JE	Forest Soil	Whole Community DNA	PCR	Amplicon	pyrotag library	454 GS FLX Titanium	European Nucleotide Archive	https://www.ebi.ac.uk/ena/data/search?query=PRJEB8599	ERS662782	SAMEA3261786	ERX709029	ERR765756	16S rRNA	V1-V3	CATAGTAGTG	AGAGTTTGATCMTGGCTCAG	GWATTACCGCGGCKGCTG	2011-08-03	46.75	-82.25	Canada	0.3	490	2.8	242	NA	Jack Pine, Balsam fir, White birch	Dfb, Humid Continental cool summer	REF	REF	0	A horizon	15.0	2.8	0.2	4.9	5.7	17.8
JE125B	JE125	JP_ON_	Ontario	JE	Forest Soil	Whole Community DNA	PCR	Amplicon	pyrotag library	454 GS FLX Titanium	European Nucleotide Archive	https://www.ebi.ac.uk/ena/data/search?query=PRJEB8599	ERS662783	SAMEA3261787	ERX709030	ERR765757	16S rRNA	V1-V3	AGCGTCGTCT	AGAGTTTGATCMTGGCTCAG	GWATTACCGCGGCKGCTG	2011-08-03	46.75	-82.25	Canada	0.1	490	2.8	242	NA	Jack Pine, Balsam fir, White birch	Dfb, Humid Continental cool summer	REF	REF	0	O horizon	73.7	38.9	1.3	3.6	5.7	29.8
JE126B	JE126	JP_ON_	Ontario	JE	Forest Soil	Whole Community DNA	PCR	Amplicon	pyrotag library	454 GS FLX Titanium	European Nucleotide Archive	https://www.ebi.ac.uk/ena/data/search?query=PRJEB8599	ERS662784	SAMEA3261788	ERX709031	ERR765758	16S rRNA	V1-V3	CGTCTAGTAC	AGAGTTTGATCMTGGCTCAG	GWATTACCGCGGCKGCTG	2011-08-03	46.75	-82.25	Canada	0.3	490	2.8	242	NA	Jack Pine, Balsam fir, White birch	Dfb, Humid Continental cool summer	REF	REF	0	A horizon	22.3	1.7	0.1	4.8	5.7	16.4
JS043B	JS043	JP_ON_	Ontario	JS	Forest Soil	Whole Community DNA	PCR	Amplicon	pyrotag library	454 GS FLX Titanium	European Nucleotide Archive	https://www.ebi.ac.uk/ena/data/search?query=PRJEB8599	ERS662785	SAMEA3261789	ERX709032	ERR765759	16S rRNA	V1-V3	TCGATCACGT	AGAGTTTGATCMTGGCTCAG	GWATTACCGCGGCKGCTG	2011-08-04	47.57	-82.85	Canada	0.1	426	1.7	250	Orthic Dystric Brunisol	Jack Pine, Black Spruce	Dfb, Humid Continental cool summer	OM1	C0	1	O horizon	42.6	44.5	1.1	4.0	7.9	39.6
JS044B	JS044	JP_ON_	Ontario	JS	Forest Soil	Whole Community DNA	PCR	Amplicon	pyrotag library	454 GS FLX Titanium	European Nucleotide Archive	https://www.ebi.ac.uk/ena/data/search?query=PRJEB8599	ERS662786	SAMEA3261790	ERX709033	ERR765760	16S rRNA	V1-V3	TACACGTGAT	AGAGTTTGATCMTGGCTCAG	GWATTACCGCGGCKGCTG	2011-08-04	47.57	-82.85	Canada	0.3	426	1.7	250	Orthic Dystric Brunisol	Jack Pine, Black Spruce	Dfb, Humid Continental cool summer	OM1	C0	1	A horizon	7.6	0.8	0.0	5.2	7.9	27.1
JS045B	JS045	JP_ON_	Ontario	JS	Forest Soil	Whole Community DNA	PCR	Amplicon	pyrotag library	454 GS FLX Titanium	European Nucleotide Archive	https://www.ebi.ac.uk/ena/data/search?query=PRJEB8599	ERS662787	SAMEA3261791	ERX709034	ERR765761	16S rRNA	V1-V3	AGTACGCTAT	AGAGTTTGATCMTGGCTCAG	GWATTACCGCGGCKGCTG	2011-08-04	47.57	-82.85	Canada	0.1	426	1.7	250	Orthic Dystric Brunisol	Jack Pine, Black Spruce	Dfb, Humid Continental cool summer	OM1	C0	0	O horizon	34.0	44.5	1.1	4.0	7.9	39.6
JS046B	JS046	JP_ON_	Ontario	JS	Forest Soil	Whole Community DNA	PCR	Amplicon	pyrotag library	454 GS FLX Titanium	European Nucleotide Archive	https://www.ebi.ac.uk/ena/data/search?query=PRJEB8599	ERS662788	SAMEA3261792	ERX709035	ERR765762	16S rRNA	V1-V3	ACTAGCAGTA	AGAGTTTGATCMTGGCTCAG	GWATTACCGCGGCKGCTG	2011-08-04	47.57	-82.85	Canada	0.3	426	1.7	250	Orthic Dystric Brunisol	Jack Pine, Black Spruce	Dfb, Humid Continental cool summer	OM1	C0	0	A horizon	4.0	0.8	0.0	5.2	7.9	27.1
JS048B	JS048	JP_ON_	Ontario	JS	Forest Soil	Whole Community DNA	PCR	Amplicon	pyrotag library	454 GS FLX Titanium	European Nucleotide Archive	https://www.ebi.ac.uk/ena/data/search?query=PRJEB8599	ERS662789	SAMEA3261793	ERX709036	ERR765763	16S rRNA	V1-V3	TCGCACTAGT	AGAGTTTGATCMTGGCTCAG	GWATTACCGCGGCKGCTG	2011-08-04	47.57	-82.85	Canada	0.3	426	1.7	250	Orthic Dystric Brunisol	Jack Pine, Black Spruce	Dfb, Humid Continental cool summer	OM3	C0	1	A horizon	17.0	0.4	0.0	5.5	7.9	26.6
JS050B	JS050	JP_ON_	Ontario	JS	Forest Soil	Whole Community DNA	PCR	Amplicon	pyrotag library	454 GS FLX Titanium	European Nucleotide Archive	https://www.ebi.ac.uk/ena/data/search?query=PRJEB8599	ERS662790	SAMEA3261794	ERX709037	ERR765764	16S rRNA	V1-V3	ACGCGAGTAT	AGAGTTTGATCMTGGCTCAG	GWATTACCGCGGCKGCTG	2011-08-04	47.57	-82.85	Canada	0.3	426	1.7	250	Orthic Dystric Brunisol	Jack Pine, Black Spruce	Dfb, Humid Continental cool summer	OM3	C0	0	A horizon	3.0	0.4	0.0	5.5	7.9	26.6
JS051B	JS051	JP_ON_	Ontario	JS	Forest Soil	Whole Community DNA	PCR	Amplicon	pyrotag library	454 GS FLX Titanium	European Nucleotide Archive	https://www.ebi.ac.uk/ena/data/search?query=PRJEB8599	ERS662791	SAMEA3261795	ERX709038	ERR765765	16S rRNA	V1-V3	ACGCTCGACA	AGAGTTTGATCMTGGCTCAG	GWATTACCGCGGCKGCTG	2011-08-04	47.57	-82.85	Canada	0.1	426	1.7	250	Orthic Dystric Brunisol	Jack Pine, Black Spruce	Dfb, Humid Continental cool summer	OM2	C0	1	O horizon	46.6	43.1	1.1	3.9	7.9	38.9
JS052B	JS052	JP_ON_	Ontario	JS	Forest Soil	Whole Community DNA	PCR	Amplicon	pyrotag library	454 GS FLX Titanium	European Nucleotide Archive	https://www.ebi.ac.uk/ena/data/search?query=PRJEB8599	ERS662792	SAMEA3261796	ERX709039	ERR765766	16S rRNA	V1-V3	ACGCGAGTAT	AGAGTTTGATCMTGGCTCAG	GWATTACCGCGGCKGCTG	2011-08-04	47.57	-82.85	Canada	0.3	426	1.7	250	Orthic Dystric Brunisol	Jack Pine, Black Spruce	Dfb, Humid Continental cool summer	OM2	C0	1	A horizon	12.7	0.6	0.0	5.3	7.9	26.0
JS053B	JS053	JP_ON_	Ontario	JS	Forest Soil	Whole Community DNA	PCR	Amplicon	pyrotag library	454 GS FLX Titanium	European Nucleotide Archive	https://www.ebi.ac.uk/ena/data/search?query=PRJEB8599	ERS662793	SAMEA3261797	ERX709040	ERR765767	16S rRNA	V1-V3	CGAGAGATAC	AGAGTTTGATCMTGGCTCAG	GWATTACCGCGGCKGCTG	2011-08-04	47.57	-82.85	Canada	0.1	426	1.7	250	Orthic Dystric Brunisol	Jack Pine, Black Spruce	Dfb, Humid Continental cool summer	OM2	C0	0	O horizon	47.5	43.1	1.1	3.9	7.9	38.9
JS054B	JS054	JP_ON_	Ontario	JS	Forest Soil	Whole Community DNA	PCR	Amplicon	pyrotag library	454 GS FLX Titanium	European Nucleotide Archive	https://www.ebi.ac.uk/ena/data/search?query=PRJEB8599	ERS662794	SAMEA3261798	ERX709041	ERR765768	16S rRNA	V1-V3	CATAGTAGTG	AGAGTTTGATCMTGGCTCAG	GWATTACCGCGGCKGCTG	2011-08-04	47.57	-82.85	Canada	0.3	426	1.7	250	Orthic Dystric Brunisol	Jack Pine, Black Spruce	Dfb, Humid Continental cool summer	OM2	C0	0	A horizon	6.7	0.6	0.0	5.3	7.9	26.0
JS056B	JS056	JP_ON_	Ontario	JS	Forest Soil	Whole Community DNA	PCR	Amplicon	pyrotag library	454 GS FLX Titanium	European Nucleotide Archive	https://www.ebi.ac.uk/ena/data/search?query=PRJEB8599	ERS662795	SAMEA3261799	ERX709042	ERR765769	16S rRNA	V1-V3	TCTATACTAT	AGAGTTTGATCMTGGCTCAG	GWATTACCGCGGCKGCTG	2011-08-04	47.57	-82.85	Canada	0.3	426	1.7	250	Orthic Dystric Brunisol	Jack Pine, Black Spruce	Dfb, Humid Continental cool summer	OM3	C0	1	A horizon	11.8	12.7	0.3	5.1	7.9	31.3
JS058B	JS058	JP_ON_	Ontario	JS	Forest Soil	Whole Community DNA	PCR	Amplicon	pyrotag library	454 GS FLX Titanium	European Nucleotide Archive	https://www.ebi.ac.uk/ena/data/search?query=PRJEB8599	ERS662796	SAMEA3261800	ERX709043	ERR765770	16S rRNA	V1-V3	CGTCTAGTAC	AGAGTTTGATCMTGGCTCAG	GWATTACCGCGGCKGCTG	2011-08-04	47.57	-82.85	Canada	0.3	426	1.7	250	Orthic Dystric Brunisol	Jack Pine, Black Spruce	Dfb, Humid Continental cool summer	OM3	C0	0	A horizon	11.7	12.7	0.3	5.1	7.9	31.3
JS059B	JS059	JP_ON_	Ontario	JS	Forest Soil	Whole Community DNA	PCR	Amplicon	pyrotag library	454 GS FLX Titanium	European Nucleotide Archive	https://www.ebi.ac.uk/ena/data/search?query=PRJEB8599	ERS662797	SAMEA3261801	ERX709044	ERR765771	16S rRNA	V1-V3	CATAGTAGTG	AGAGTTTGATCMTGGCTCAG	GWATTACCGCGGCKGCTG	2011-08-04	47.57	-82.85	Canada	0.1	426	1.7	250	Orthic Dystric Brunisol	Jack Pine, Black Spruce	Dfb, Humid Continental cool summer	OM2	C0	1	O horizon	44.7	42.7	1.0	4.0	7.9	42.6
JS060B	JS060	JP_ON_	Ontario	JS	Forest Soil	Whole Community DNA	PCR	Amplicon	pyrotag library	454 GS FLX Titanium	European Nucleotide Archive	https://www.ebi.ac.uk/ena/data/search?query=PRJEB8599	ERS662798	SAMEA3261802	ERX709045	ERR765772	16S rRNA	V1-V3	CGTGTCTCTA	AGAGTTTGATCMTGGCTCAG	GWATTACCGCGGCKGCTG	2011-08-04	47.57	-82.85	Canada	0.3	426	1.7	250	Orthic Dystric Brunisol	Jack Pine, Black Spruce	Dfb, Humid Continental cool summer	OM2	C0	1	A horizon	17.9	1.2	0.1	5.3	7.9	25.6
JS061B	JS061	JP_ON_	Ontario	JS	Forest Soil	Whole Community DNA	PCR	Amplicon	pyrotag library	454 GS FLX Titanium	European Nucleotide Archive	https://www.ebi.ac.uk/ena/data/search?query=PRJEB8599	ERS662799	SAMEA3261803	ERX709046	ERR765773	16S rRNA	V1-V3	AGCGTCGTCT	AGAGTTTGATCMTGGCTCAG	GWATTACCGCGGCKGCTG	2011-08-04	47.57	-82.85	Canada	0.1	426	1.7	250	Orthic Dystric Brunisol	Jack Pine, Black Spruce	Dfb, Humid Continental cool summer	OM2	C0	0	O horizon	NA	NA	NA	NA	NA	NA
JS062B	JS062	JP_ON_	Ontario	JS	Forest Soil	Whole Community DNA	PCR	Amplicon	pyrotag library	454 GS FLX Titanium	European Nucleotide Archive	https://www.ebi.ac.uk/ena/data/search?query=PRJEB8599	ERS662800	SAMEA3261804	ERX709047	ERR765774	16S rRNA	V1-V3	TACGCTGTCT	AGAGTTTGATCMTGGCTCAG	GWATTACCGCGGCKGCTG	2011-08-04	47.57	-82.85	Canada	0.3	426	1.7	250	Orthic Dystric Brunisol	Jack Pine, Black Spruce	Dfb, Humid Continental cool summer	OM2	C0	0	A horizon	6.9	1.2	0.1	5.3	7.9	25.6
JS063B	JS063	JP_ON_	Ontario	JS	Forest Soil	Whole Community DNA	PCR	Amplicon	pyrotag library	454 GS FLX Titanium	European Nucleotide Archive	https://www.ebi.ac.uk/ena/data/search?query=PRJEB8599	ERS662801	SAMEA3261805	ERX709048	ERR765775	16S rRNA	V1-V3	CGACGTGACT	AGAGTTTGATCMTGGCTCAG	GWATTACCGCGGCKGCTG	2011-08-04	47.57	-82.85	Canada	0.1	426	1.7	250	Orthic Dystric Brunisol	Jack Pine, Black Spruce	Dfb, Humid Continental cool summer	OM1	C0	1	O horizon	28.4	46.5	1.2	4.1	7.9	40.7
JS064B	JS064	JP_ON_	Ontario	JS	Forest Soil	Whole Community DNA	PCR	Amplicon	pyrotag library	454 GS FLX Titanium	European Nucleotide Archive	https://www.ebi.ac.uk/ena/data/search?query=PRJEB8599	ERS662802	SAMEA3261806	ERX709049	ERR765776	16S rRNA	V1-V3	TCACGTACTA	AGAGTTTGATCMTGGCTCAG	GWATTACCGCGGCKGCTG	2011-08-04	47.57	-82.85	Canada	0.3	426	1.7	250	Orthic Dystric Brunisol	Jack Pine, Black Spruce	Dfb, Humid Continental cool summer	OM1	C0	1	A horizon	6.0	1.0	0.0	5.2	7.9	24.1
JS065B	JS065	JP_ON_	Ontario	JS	Forest Soil	Whole Community DNA	PCR	Amplicon	pyrotag library	454 GS FLX Titanium	European Nucleotide Archive	https://www.ebi.ac.uk/ena/data/search?query=PRJEB8599	ERS662803	SAMEA3261807	ERX709050	ERR765777	16S rRNA	V1-V3	TCACGTACTA	AGAGTTTGATCMTGGCTCAG	GWATTACCGCGGCKGCTG	2011-08-04	47.57	-82.85	Canada	0.1	426	1.7	250	Orthic Dystric Brunisol	Jack Pine, Black Spruce	Dfb, Humid Continental cool summer	OM1	C0	0	O horizon	36.5	46.5	1.2	4.1	7.9	40.7
JS066B	JS066	JP_ON_	Ontario	JS	Forest Soil	Whole Community DNA	PCR	Amplicon	pyrotag library	454 GS FLX Titanium	European Nucleotide Archive	https://www.ebi.ac.uk/ena/data/search?query=PRJEB8599	ERS662804	SAMEA3261808	ERX709051	ERR765778	16S rRNA	V1-V3	ACGAGTGCGT	AGAGTTTGATCMTGGCTCAG	GWATTACCGCGGCKGCTG	2011-08-04	47.57	-82.85	Canada	0.3	426	1.7	250	Orthic Dystric Brunisol	Jack Pine, Black Spruce	Dfb, Humid Continental cool summer	OM1	C0	0	A horizon	7.8	1.0	0.0	5.2	7.9	24.1
JS068B	JS068	JP_ON_	Ontario	JS	Forest Soil	Whole Community DNA	PCR	Amplicon	pyrotag library	454 GS FLX Titanium	European Nucleotide Archive	https://www.ebi.ac.uk/ena/data/search?query=PRJEB8599	ERS662805	SAMEA3261809	ERX709052	ERR765779	16S rRNA	V1-V3	AGACGCACTC	AGAGTTTGATCMTGGCTCAG	GWATTACCGCGGCKGCTG	2011-08-04	47.57	-82.85	Canada	0.3	426	1.7	250	Orthic Dystric Brunisol	Jack Pine, Black Spruce	Dfb, Humid Continental cool summer	OM2	C0	1	A horizon	13.9	0.8	0.0	5.2	7.9	26.8
JS072B	JS072	JP_ON_	Ontario	JS	Forest Soil	Whole Community DNA	PCR	Amplicon	pyrotag library	454 GS FLX Titanium	European Nucleotide Archive	https://www.ebi.ac.uk/ena/data/search?query=PRJEB8599	ERS662806	SAMEA3261810	ERX709053	ERR765780	16S rRNA	V1-V3	TGATACGTCT	AGAGTTTGATCMTGGCTCAG	GWATTACCGCGGCKGCTG	2011-08-04	47.57	-82.85	Canada	0.3	426	1.7	250	Orthic Dystric Brunisol	Jack Pine, Black Spruce	Dfb, Humid Continental cool summer	OM3	C0	1	A horizon	9.1	0.4	0.0	5.3	7.9	24.1
JS074B	JS074	JP_ON_	Ontario	JS	Forest Soil	Whole Community DNA	PCR	Amplicon	pyrotag library	454 GS FLX Titanium	European Nucleotide Archive	https://www.ebi.ac.uk/ena/data/search?query=PRJEB8599	ERS662807	SAMEA3261811	ERX709054	ERR765781	16S rRNA	V1-V3	CGTGTCTCTA	AGAGTTTGATCMTGGCTCAG	GWATTACCGCGGCKGCTG	2011-08-04	47.57	-82.85	Canada	0.3	426	1.7	250	Orthic Dystric Brunisol	Jack Pine, Black Spruce	Dfb, Humid Continental cool summer	OM3	C0	0	A horizon	5.9	0.4	0.0	5.3	7.9	24.1
JS075B	JS075	JP_ON_	Ontario	JS	Forest Soil	Whole Community DNA	PCR	Amplicon	pyrotag library	454 GS FLX Titanium	European Nucleotide Archive	https://www.ebi.ac.uk/ena/data/search?query=PRJEB8599	ERS662808	SAMEA3261812	ERX709055	ERR765782	16S rRNA	V1-V3	TACACGTGAT	AGAGTTTGATCMTGGCTCAG	GWATTACCGCGGCKGCTG	2011-08-04	47.57	-82.85	Canada	0.1	426	1.7	250	Orthic Dystric Brunisol	Jack Pine, Black Spruce	Dfb, Humid Continental cool summer	OM1	C0	1	O horizon	32.4	43.4	1.1	3.9	7.9	40.0
JS076B	JS076	JP_ON_	Ontario	JS	Forest Soil	Whole Community DNA	PCR	Amplicon	pyrotag library	454 GS FLX Titanium	European Nucleotide Archive	https://www.ebi.ac.uk/ena/data/search?query=PRJEB8599	ERS662809	SAMEA3261813	ERX709056	ERR765783	16S rRNA	V1-V3	CACGCTACGT	AGAGTTTGATCMTGGCTCAG	GWATTACCGCGGCKGCTG	2011-08-04	47.57	-82.85	Canada	0.3	426	1.7	250	Orthic Dystric Brunisol	Jack Pine, Black Spruce	Dfb, Humid Continental cool summer	OM1	C0	1	A horizon	5.9	0.8	0.0	5.3	7.9	23.0
JS077B	JS077	JP_ON_	Ontario	JS	Forest Soil	Whole Community DNA	PCR	Amplicon	pyrotag library	454 GS FLX Titanium	European Nucleotide Archive	https://www.ebi.ac.uk/ena/data/search?query=PRJEB8599	ERS662810	SAMEA3261814	ERX709057	ERR765784	16S rRNA	V1-V3	TCACGTACTA	AGAGTTTGATCMTGGCTCAG	GWATTACCGCGGCKGCTG	2011-08-04	47.57	-82.85	Canada	0.1	426	1.7	250	Orthic Dystric Brunisol	Jack Pine, Black Spruce	Dfb, Humid Continental cool summer	OM1	C0	0	O horizon	33.7	43.4	1.1	3.9	7.9	40.0
JS078B	JS078	JP_ON_	Ontario	JS	Forest Soil	Whole Community DNA	PCR	Amplicon	pyrotag library	454 GS FLX Titanium	European Nucleotide Archive	https://www.ebi.ac.uk/ena/data/search?query=PRJEB8599	ERS662811	SAMEA3261815	ERX709058	ERR765785	16S rRNA	V1-V3	CAGTAGACGT	AGAGTTTGATCMTGGCTCAG	GWATTACCGCGGCKGCTG	2011-08-04	47.57	-82.85	Canada	0.3	426	1.7	250	Orthic Dystric Brunisol	Jack Pine, Black Spruce	Dfb, Humid Continental cool summer	OM1	C0	0	A horizon	4.1	0.8	0.0	5.3	7.9	23.0
JS079B	JS079	JP_ON_	Ontario	JS	Forest Soil	Whole Community DNA	PCR	Amplicon	pyrotag library	454 GS FLX Titanium	European Nucleotide Archive	https://www.ebi.ac.uk/ena/data/search?query=PRJEB8599	ERS662812	SAMEA3261816	ERX709059	ERR765786	16S rRNA	V1-V3	ACTGTACAGT	AGAGTTTGATCMTGGCTCAG	GWATTACCGCGGCKGCTG	2011-08-04	47.57	-82.85	Canada	0.1	426	1.7	250	Orthic Dystric Brunisol	Jack Pine, Black Spruce	Dfb, Humid Continental cool summer	REF	REF	0	O horizon	46.5	45.7	1.3	3.7	7.9	36.6
JS080B	JS080	JP_ON_	Ontario	JS	Forest Soil	Whole Community DNA	PCR	Amplicon	pyrotag library	454 GS FLX Titanium	European Nucleotide Archive	https://www.ebi.ac.uk/ena/data/search?query=PRJEB8599	ERS662813	SAMEA3261817	ERX709060	ERR765787	16S rRNA	V1-V3	CGTCTAGTAC	AGAGTTTGATCMTGGCTCAG	GWATTACCGCGGCKGCTG	2011-08-04	47.57	-82.85	Canada	0.3	426	1.7	250	Orthic Dystric Brunisol	Jack Pine, Black Spruce	Dfb, Humid Continental cool summer	REF	REF	0	A horizon	15.7	1.0	0.1	5.2	7.9	16.4
JS082B	JS082	JP_ON_	Ontario	JS	Forest Soil	Whole Community DNA	PCR	Amplicon	pyrotag library	454 GS FLX Titanium	European Nucleotide Archive	https://www.ebi.ac.uk/ena/data/search?query=PRJEB8599	ERS662814	SAMEA3261818	ERX709061	ERR765788	16S rRNA	V1-V3	AGTACGCTAT	AGAGTTTGATCMTGGCTCAG	GWATTACCGCGGCKGCTG	2011-08-04	47.57	-82.85	Canada	0.3	426	1.7	250	Orthic Dystric Brunisol	Jack Pine, Black Spruce	Dfb, Humid Continental cool summer	REF	REF	0	A horizon	17.1	1.0	0.0	5.1	7.8	23.8
JS083B	JS083	JP_ON_	Ontario	JS	Forest Soil	Whole Community DNA	PCR	Amplicon	pyrotag library	454 GS FLX Titanium	European Nucleotide Archive	https://www.ebi.ac.uk/ena/data/search?query=PRJEB8599	ERS662815	SAMEA3261819	ERX709062	ERR765789	16S rRNA	V1-V3	AGACGCACTC	AGAGTTTGATCMTGGCTCAG	GWATTACCGCGGCKGCTG	2011-08-04	47.57	-82.85	Canada	0.1	426	1.7	250	Orthic Dystric Brunisol	Jack Pine, Black Spruce	Dfb, Humid Continental cool summer	REF	REF	0	O horizon	49.1	44.6	1.2	3.6	7.8	35.9
JS084B	JS084	JP_ON_	Ontario	JS	Forest Soil	Whole Community DNA	PCR	Amplicon	pyrotag library	454 GS FLX Titanium	European Nucleotide Archive	https://www.ebi.ac.uk/ena/data/search?query=PRJEB8599	ERS662816	SAMEA3261820	ERX709063	ERR765790	16S rRNA	V1-V3	TACGCTGTCT	AGAGTTTGATCMTGGCTCAG	GWATTACCGCGGCKGCTG	2011-08-04	47.57	-82.85	Canada	0.3	426	1.7	250	Orthic Dystric Brunisol	Jack Pine, Black Spruce	Dfb, Humid Continental cool summer	REF	REF	0	A horizon	12.1	1.2	0.1	5.0	7.8	23.2
JW002B	JW002	JP_ON_	Ontario	JW	Forest Soil	Whole Community DNA	PCR	Amplicon	pyrotag library	454 GS FLX Titanium	European Nucleotide Archive	https://www.ebi.ac.uk/ena/data/search?query=PRJEB8599	ERS662817	SAMEA3261821	ERX709064	ERR765791	16S rRNA	V1-V3	CTCGCGTGTC	AGAGTTTGATCMTGGCTCAG	GWATTACCGCGGCKGCTG	2011-07-07	46.42	-83.37	Canada	0.3	228	4.4	248	Orthic Humo-Ferric Podzol	Jack Pine, Black Spruce, Red Pine	Dfb, Humid Continental cool summer	OM3	C0	1	A horizon	12.0	2.2	0.1	5.4	7.2	17.8
JW003B	JW003	JP_ON_	Ontario	JW	Forest Soil	Whole Community DNA	PCR	Amplicon	pyrotag library	454 GS FLX Titanium	European Nucleotide Archive	https://www.ebi.ac.uk/ena/data/search?query=PRJEB8599	ERS662818	SAMEA3261822	ERX709065	ERR765792	16S rRNA	V1-V3	TCTACGTAGC	AGAGTTTGATCMTGGCTCAG	GWATTACCGCGGCKGCTG	2011-07-07	46.42	-83.37	Canada	0.1	228	4.4	248	Orthic Humo-Ferric Podzol	Jack Pine, Black Spruce, Red Pine	Dfb, Humid Continental cool summer	OM3	C0	0	O horizon	37.0	38.3	1.1	4.1	7.2	37.3
JW004B	JW004	JP_ON_	Ontario	JW	Forest Soil	Whole Community DNA	PCR	Amplicon	pyrotag library	454 GS FLX Titanium	European Nucleotide Archive	https://www.ebi.ac.uk/ena/data/search?query=PRJEB8599	ERS662819	SAMEA3261823	ERX709066	ERR765793	16S rRNA	V1-V3	ACGCGAGTAT	AGAGTTTGATCMTGGCTCAG	GWATTACCGCGGCKGCTG	2011-07-07	46.42	-83.37	Canada	0.3	228	4.4	248	Orthic Humo-Ferric Podzol	Jack Pine, Black Spruce, Red Pine	Dfb, Humid Continental cool summer	OM3	C0	0	A horizon	11.0	2.2	0.1	5.4	7.2	17.8
JW005B	JW005	JP_ON_	Ontario	JW	Forest Soil	Whole Community DNA	PCR	Amplicon	pyrotag library	454 GS FLX Titanium	European Nucleotide Archive	https://www.ebi.ac.uk/ena/data/search?query=PRJEB8599	ERS662820	SAMEA3261824	ERX709067	ERR765794	16S rRNA	V1-V3	ACGCGAGTAT	AGAGTTTGATCMTGGCTCAG	GWATTACCGCGGCKGCTG	2011-07-07	46.42	-83.37	Canada	0.1	228	4.4	248	Orthic Humo-Ferric Podzol	Jack Pine, Black Spruce, Red Pine	Dfb, Humid Continental cool summer	OM1	C0	1	O horizon	38.1	36.4	1.1	4.2	6.3	33.0
JW006B	JW006	JP_ON_	Ontario	JW	Forest Soil	Whole Community DNA	PCR	Amplicon	pyrotag library	454 GS FLX Titanium	European Nucleotide Archive	https://www.ebi.ac.uk/ena/data/search?query=PRJEB8599	ERS662821	SAMEA3261825	ERX709068	ERR765795	16S rRNA	V1-V3	ACTGTACAGT	AGAGTTTGATCMTGGCTCAG	GWATTACCGCGGCKGCTG	2011-07-07	46.42	-83.37	Canada	0.3	228	4.4	248	Orthic Humo-Ferric Podzol	Jack Pine, Black Spruce, Red Pine	Dfb, Humid Continental cool summer	OM1	C0	1	A horizon	15.7	2.7	0.2	5.3	6.3	16.0
JW007B	JW007	JP_ON_	Ontario	JW	Forest Soil	Whole Community DNA	PCR	Amplicon	pyrotag library	454 GS FLX Titanium	European Nucleotide Archive	https://www.ebi.ac.uk/ena/data/search?query=PRJEB8599	ERS662822	SAMEA3261826	ERX709069	ERR765796	16S rRNA	V1-V3	TCTACGTAGC	AGAGTTTGATCMTGGCTCAG	GWATTACCGCGGCKGCTG	2011-07-07	46.42	-83.37	Canada	0.1	228	4.4	248	Orthic Humo-Ferric Podzol	Jack Pine, Black Spruce, Red Pine	Dfb, Humid Continental cool summer	OM1	C0	0	O horizon	40.0	36.4	1.1	4.2	6.3	33.0
JW008B	JW008	JP_ON_	Ontario	JW	Forest Soil	Whole Community DNA	PCR	Amplicon	pyrotag library	454 GS FLX Titanium	European Nucleotide Archive	https://www.ebi.ac.uk/ena/data/search?query=PRJEB8599	ERS662823	SAMEA3261827	ERX709070	ERR765797	16S rRNA	V1-V3	AGTACGCTAT	AGAGTTTGATCMTGGCTCAG	GWATTACCGCGGCKGCTG	2011-07-07	46.42	-83.37	Canada	0.3	228	4.4	248	Orthic Humo-Ferric Podzol	Jack Pine, Black Spruce, Red Pine	Dfb, Humid Continental cool summer	OM1	C0	0	A horizon	9.8	2.7	0.2	5.3	6.3	16.0
JW010B	JW010	JP_ON_	Ontario	JW	Forest Soil	Whole Community DNA	PCR	Amplicon	pyrotag library	454 GS FLX Titanium	European Nucleotide Archive	https://www.ebi.ac.uk/ena/data/search?query=PRJEB8599	ERS662824	SAMEA3261828	ERX709071	ERR765798	16S rRNA	V1-V3	CGTCTAGTAC	AGAGTTTGATCMTGGCTCAG	GWATTACCGCGGCKGCTG	2011-07-07	46.42	-83.37	Canada	0.3	228	4.4	248	Orthic Humo-Ferric Podzol	Jack Pine, Black Spruce, Red Pine	Dfb, Humid Continental cool summer	OM3	C0	1	A horizon	17.6	1.5	0.1	5.5	8.9	15.6
JW012B	JW012	JP_ON_	Ontario	JW	Forest Soil	Whole Community DNA	PCR	Amplicon	pyrotag library	454 GS FLX Titanium	European Nucleotide Archive	https://www.ebi.ac.uk/ena/data/search?query=PRJEB8599	ERS662825	SAMEA3261829	ERX709072	ERR765799	16S rRNA	V1-V3	CGAGAGATAC	AGAGTTTGATCMTGGCTCAG	GWATTACCGCGGCKGCTG	2011-07-07	46.42	-83.37	Canada	0.3	228	4.4	248	Orthic Humo-Ferric Podzol	Jack Pine, Black Spruce, Red Pine	Dfb, Humid Continental cool summer	OM3	C0	0	A horizon	13.3	1.5	0.1	5.5	8.9	15.6
JW013B	JW013	JP_ON_	Ontario	JW	Forest Soil	Whole Community DNA	PCR	Amplicon	pyrotag library	454 GS FLX Titanium	European Nucleotide Archive	https://www.ebi.ac.uk/ena/data/search?query=PRJEB8599	ERS662826	SAMEA3261830	ERX709073	ERR765800	16S rRNA	V1-V3	TAGTGTAGAT	AGAGTTTGATCMTGGCTCAG	GWATTACCGCGGCKGCTG	2011-07-07	46.42	-83.37	Canada	0.1	228	4.4	248	Orthic Humo-Ferric Podzol	Jack Pine, Black Spruce, Red Pine	Dfb, Humid Continental cool summer	REF	REF	0	O horizon	31.0	38.1	1.1	4.1	8.9	36.6
JW014B	JW014	JP_ON_	Ontario	JW	Forest Soil	Whole Community DNA	PCR	Amplicon	pyrotag library	454 GS FLX Titanium	European Nucleotide Archive	https://www.ebi.ac.uk/ena/data/search?query=PRJEB8599	ERS662827	SAMEA3261831	ERX709074	ERR765801	16S rRNA	V1-V3	TCTACGTAGC	AGAGTTTGATCMTGGCTCAG	GWATTACCGCGGCKGCTG	2011-07-07	46.42	-83.37	Canada	0.3	228	4.4	248	Orthic Humo-Ferric Podzol	Jack Pine, Black Spruce, Red Pine	Dfb, Humid Continental cool summer	REF	REF	0	A horizon	16.0	1.5	0.1	5.5	8.9	15.6
JW015B	JW015	JP_ON_	Ontario	JW	Forest Soil	Whole Community DNA	PCR	Amplicon	pyrotag library	454 GS FLX Titanium	European Nucleotide Archive	https://www.ebi.ac.uk/ena/data/search?query=PRJEB8599	ERS662828	SAMEA3261832	ERX709075	ERR765802	16S rRNA	V1-V3	TCGCACTAGT	AGAGTTTGATCMTGGCTCAG	GWATTACCGCGGCKGCTG	2011-07-07	46.42	-83.37	Canada	0.1	228	4.4	248	Orthic Humo-Ferric Podzol	Jack Pine, Black Spruce, Red Pine	Dfb, Humid Continental cool summer	OM2	C0	1	O horizon	29.7	36.9	1.2	4.2	9.8	30.8
JW016B	JW016	JP_ON_	Ontario	JW	Forest Soil	Whole Community DNA	PCR	Amplicon	pyrotag library	454 GS FLX Titanium	European Nucleotide Archive	https://www.ebi.ac.uk/ena/data/search?query=PRJEB8599	ERS662829	SAMEA3261833	ERX709076	ERR765803	16S rRNA	V1-V3	ACAGTATATA	AGAGTTTGATCMTGGCTCAG	GWATTACCGCGGCKGCTG	2011-07-07	46.42	-83.37	Canada	0.3	228	4.4	248	Orthic Humo-Ferric Podzol	Jack Pine, Black Spruce, Red Pine	Dfb, Humid Continental cool summer	OM2	C0	1	A horizon	15.2	2.6	0.2	5.4	9.8	16.2
JW017B	JW017	JP_ON_	Ontario	JW	Forest Soil	Whole Community DNA	PCR	Amplicon	pyrotag library	454 GS FLX Titanium	European Nucleotide Archive	https://www.ebi.ac.uk/ena/data/search?query=PRJEB8599	ERS662830	SAMEA3261834	ERX709077	ERR765804	16S rRNA	V1-V3	ACTACTATGT	AGAGTTTGATCMTGGCTCAG	GWATTACCGCGGCKGCTG	2011-07-07	46.42	-83.37	Canada	0.1	228	4.4	248	Orthic Humo-Ferric Podzol	Jack Pine, Black Spruce, Red Pine	Dfb, Humid Continental cool summer	OM2	C0	0	O horizon	46.7	36.9	1.2	4.2	9.8	30.8
JW018B	JW018	JP_ON_	Ontario	JW	Forest Soil	Whole Community DNA	PCR	Amplicon	pyrotag library	454 GS FLX Titanium	European Nucleotide Archive	https://www.ebi.ac.uk/ena/data/search?query=PRJEB8599	ERS662831	SAMEA3261835	ERX709078	ERR765805	16S rRNA	V1-V3	TCTAGCGACT	AGAGTTTGATCMTGGCTCAG	GWATTACCGCGGCKGCTG	2011-07-07	46.42	-83.37	Canada	0.3	228	4.4	248	Orthic Humo-Ferric Podzol	Jack Pine, Black Spruce, Red Pine	Dfb, Humid Continental cool summer	OM2	C0	0	A horizon	14.7	2.6	0.2	5.4	9.8	16.2
JW019B	JW019	JP_ON_	Ontario	JW	Forest Soil	Whole Community DNA	PCR	Amplicon	pyrotag library	454 GS FLX Titanium	European Nucleotide Archive	https://www.ebi.ac.uk/ena/data/search?query=PRJEB8599	ERS662832	SAMEA3261836	ERX709079	ERR765806	16S rRNA	V1-V3	TCTATACTAT	AGAGTTTGATCMTGGCTCAG	GWATTACCGCGGCKGCTG	2011-07-07	46.42	-83.37	Canada	0.1	228	4.4	248	Orthic Humo-Ferric Podzol	Jack Pine, Black Spruce, Red Pine	Dfb, Humid Continental cool summer	REF	REF	0	O horizon	31.4	36.9	1.2	4.2	9.8	30.8
JW020B	JW020	JP_ON_	Ontario	JW	Forest Soil	Whole Community DNA	PCR	Amplicon	pyrotag library	454 GS FLX Titanium	European Nucleotide Archive	https://www.ebi.ac.uk/ena/data/search?query=PRJEB8599	ERS662833	SAMEA3261837	ERX709080	ERR765807	16S rRNA	V1-V3	CATAGTAGTG	AGAGTTTGATCMTGGCTCAG	GWATTACCGCGGCKGCTG	2011-07-07	46.42	-83.37	Canada	0.3	228	4.4	248	Orthic Humo-Ferric Podzol	Jack Pine, Black Spruce, Red Pine	Dfb, Humid Continental cool summer	REF	REF	0	A horizon	12.0	2.6	0.2	5.4	9.8	16.2
JW021B	JW021	JP_ON_	Ontario	JW	Forest Soil	Whole Community DNA	PCR	Amplicon	pyrotag library	454 GS FLX Titanium	European Nucleotide Archive	https://www.ebi.ac.uk/ena/data/search?query=PRJEB8599	ERS662834	SAMEA3261838	ERX709081	ERR765808	16S rRNA	V1-V3	ACTAGCAGTA	AGAGTTTGATCMTGGCTCAG	GWATTACCGCGGCKGCTG	2011-07-07	46.42	-83.37	Canada	0.1	228	4.4	248	Orthic Humo-Ferric Podzol	Jack Pine, Black Spruce, Red Pine	Dfb, Humid Continental cool summer	OM1	C0	1	O horizon	43.0	34.6	1.1	4.1	7.7	30.1
JW022B	JW022	JP_ON_	Ontario	JW	Forest Soil	Whole Community DNA	PCR	Amplicon	pyrotag library	454 GS FLX Titanium	European Nucleotide Archive	https://www.ebi.ac.uk/ena/data/search?query=PRJEB8599	ERS662835	SAMEA3261839	ERX709082	ERR765809	16S rRNA	V1-V3	ATACGACGTA	AGAGTTTGATCMTGGCTCAG	GWATTACCGCGGCKGCTG	2011-07-07	46.42	-83.37	Canada	0.3	228	4.4	248	Orthic Humo-Ferric Podzol	Jack Pine, Black Spruce, Red Pine	Dfb, Humid Continental cool summer	OM1	C0	1	A horizon	13.7	3.1	0.2	5.3	7.7	14.8
JW023B	JW023	JP_ON_	Ontario	JW	Forest Soil	Whole Community DNA	PCR	Amplicon	pyrotag library	454 GS FLX Titanium	European Nucleotide Archive	https://www.ebi.ac.uk/ena/data/search?query=PRJEB8599	ERS662836	SAMEA3261840	ERX709083	ERR765810	16S rRNA	V1-V3	ATCAGACACG	AGAGTTTGATCMTGGCTCAG	GWATTACCGCGGCKGCTG	2011-07-07	46.42	-83.37	Canada	0.1	228	4.4	248	Orthic Humo-Ferric Podzol	Jack Pine, Black Spruce, Red Pine	Dfb, Humid Continental cool summer	OM1	C0	0	O horizon	46.7	34.6	1.1	4.1	7.7	30.1
JW024B	JW024	JP_ON_	Ontario	JW	Forest Soil	Whole Community DNA	PCR	Amplicon	pyrotag library	454 GS FLX Titanium	European Nucleotide Archive	https://www.ebi.ac.uk/ena/data/search?query=PRJEB8599	ERS662837	SAMEA3261841	ERX709084	ERR765811	16S rRNA	V1-V3	ATATCGCGAG	AGAGTTTGATCMTGGCTCAG	GWATTACCGCGGCKGCTG	2011-07-07	46.42	-83.37	Canada	0.3	228	4.4	248	Orthic Humo-Ferric Podzol	Jack Pine, Black Spruce, Red Pine	Dfb, Humid Continental cool summer	OM1	C0	0	A horizon	12.2	3.1	0.2	5.3	7.7	14.8
JW026B	JW026	JP_ON_	Ontario	JW	Forest Soil	Whole Community DNA	PCR	Amplicon	pyrotag library	454 GS FLX Titanium	European Nucleotide Archive	https://www.ebi.ac.uk/ena/data/search?query=PRJEB8599	ERS662838	SAMEA3261842	ERX709085	ERR765812	16S rRNA	V1-V3	TCTATACTAT	AGAGTTTGATCMTGGCTCAG	GWATTACCGCGGCKGCTG	2011-07-07	46.42	-83.37	Canada	0.3	228	4.4	248	Orthic Humo-Ferric Podzol	Jack Pine, Black Spruce, Red Pine	Dfb, Humid Continental cool summer	REF	REF	0	A horizon	14.0	3.1	0.2	4.1	7.7	14.8
JW027B	JW027	JP_ON_	Ontario	JW	Forest Soil	Whole Community DNA	PCR	Amplicon	pyrotag library	454 GS FLX Titanium	European Nucleotide Archive	https://www.ebi.ac.uk/ena/data/search?query=PRJEB8599	ERS662839	SAMEA3261843	ERX709086	ERR765813	16S rRNA	V1-V3	ATAGAGTACT	AGAGTTTGATCMTGGCTCAG	GWATTACCGCGGCKGCTG	2011-07-07	46.42	-83.37	Canada	0.1	228	4.4	248	Orthic Humo-Ferric Podzol	Jack Pine, Black Spruce, Red Pine	Dfb, Humid Continental cool summer	OM1	C0	1	O horizon	33.7	33.8	1.0	4.4	6.3	32.8
JW028B	JW028	JP_ON_	Ontario	JW	Forest Soil	Whole Community DNA	PCR	Amplicon	pyrotag library	454 GS FLX Titanium	European Nucleotide Archive	https://www.ebi.ac.uk/ena/data/search?query=PRJEB8599	ERS662840	SAMEA3261844	ERX709087	ERR765814	16S rRNA	V1-V3	TGACGTATGT	AGAGTTTGATCMTGGCTCAG	GWATTACCGCGGCKGCTG	2011-07-07	46.42	-83.37	Canada	0.3	228	4.4	248	Orthic Humo-Ferric Podzol	Jack Pine, Black Spruce, Red Pine	Dfb, Humid Continental cool summer	OM1	C0	1	A horizon	13.0	3.3	0.3	5.3	6.3	13.6
JW029B	JW029	JP_ON_	Ontario	JW	Forest Soil	Whole Community DNA	PCR	Amplicon	pyrotag library	454 GS FLX Titanium	European Nucleotide Archive	https://www.ebi.ac.uk/ena/data/search?query=PRJEB8599	ERS662841	SAMEA3261845	ERX709088	ERR765815	16S rRNA	V1-V3	CAGTAGACGT	AGAGTTTGATCMTGGCTCAG	GWATTACCGCGGCKGCTG	2011-07-07	46.42	-83.37	Canada	0.1	228	4.4	248	Orthic Humo-Ferric Podzol	Jack Pine, Black Spruce, Red Pine	Dfb, Humid Continental cool summer	OM1	C0	0	O horizon	57.0	33.8	1.0	4.4	6.3	32.8
JW030B	JW030	JP_ON_	Ontario	JW	Forest Soil	Whole Community DNA	PCR	Amplicon	pyrotag library	454 GS FLX Titanium	European Nucleotide Archive	https://www.ebi.ac.uk/ena/data/search?query=PRJEB8599	ERS662842	SAMEA3261846	ERX709089	ERR765816	16S rRNA	V1-V3	ACGCGAGTAT	AGAGTTTGATCMTGGCTCAG	GWATTACCGCGGCKGCTG	2011-07-07	46.42	-83.37	Canada	0.3	228	4.4	248	Orthic Humo-Ferric Podzol	Jack Pine, Black Spruce, Red Pine	Dfb, Humid Continental cool summer	OM1	C0	0	A horizon	13.7	3.3	0.3	5.3	6.3	13.6
JW031B	JW031	JP_ON_	Ontario	JW	Forest Soil	Whole Community DNA	PCR	Amplicon	pyrotag library	454 GS FLX Titanium	European Nucleotide Archive	https://www.ebi.ac.uk/ena/data/search?query=PRJEB8599	ERS662843	SAMEA3261847	ERX709090	ERR765817	16S rRNA	V1-V3	TAGTGTAGAT	AGAGTTTGATCMTGGCTCAG	GWATTACCGCGGCKGCTG	2011-07-07	46.42	-83.37	Canada	0.1	228	4.4	248	Orthic Humo-Ferric Podzol	Jack Pine, Black Spruce, Red Pine	Dfb, Humid Continental cool summer	OM2	C0	1	O horizon	40.4	37.6	1.1	4.0	7.7	33.6
JW032B	JW032	JP_ON_	Ontario	JW	Forest Soil	Whole Community DNA	PCR	Amplicon	pyrotag library	454 GS FLX Titanium	European Nucleotide Archive	https://www.ebi.ac.uk/ena/data/search?query=PRJEB8599	ERS662844	SAMEA3261848	ERX709091	ERR765818	16S rRNA	V1-V3	TGATACGTCT	AGAGTTTGATCMTGGCTCAG	GWATTACCGCGGCKGCTG	2011-07-07	46.42	-83.37	Canada	0.3	228	4.4	248	Orthic Humo-Ferric Podzol	Jack Pine, Black Spruce, Red Pine	Dfb, Humid Continental cool summer	OM2	C0	1	A horizon	16.7	3.0	0.4	5.1	7.7	13.9
JW033B	JW033	JP_ON_	Ontario	JW	Forest Soil	Whole Community DNA	PCR	Amplicon	pyrotag library	454 GS FLX Titanium	European Nucleotide Archive	https://www.ebi.ac.uk/ena/data/search?query=PRJEB8599	ERS662845	SAMEA3261849	ERX709092	ERR765819	16S rRNA	V1-V3	TGATACGTCT	AGAGTTTGATCMTGGCTCAG	GWATTACCGCGGCKGCTG	2011-07-07	46.42	-83.37	Canada	0.1	228	4.4	248	Orthic Humo-Ferric Podzol	Jack Pine, Black Spruce, Red Pine	Dfb, Humid Continental cool summer	OM2	C0	0	O horizon	60.4	37.6	1.1	4.0	7.7	33.6
JW034B	JW034	JP_ON_	Ontario	JW	Forest Soil	Whole Community DNA	PCR	Amplicon	pyrotag library	454 GS FLX Titanium	European Nucleotide Archive	https://www.ebi.ac.uk/ena/data/search?query=PRJEB8599	ERS662846	SAMEA3261850	ERX709093	ERR765820	16S rRNA	V1-V3	TCACGTACTA	AGAGTTTGATCMTGGCTCAG	GWATTACCGCGGCKGCTG	2011-07-07	46.42	-83.37	Canada	0.3	228	4.4	248	Orthic Humo-Ferric Podzol	Jack Pine, Black Spruce, Red Pine	Dfb, Humid Continental cool summer	OM2	C0	0	A horizon	16.3	3.0	0.4	5.1	7.7	13.9
JW035B	JW035	JP_ON_	Ontario	JW	Forest Soil	Whole Community DNA	PCR	Amplicon	pyrotag library	454 GS FLX Titanium	European Nucleotide Archive	https://www.ebi.ac.uk/ena/data/search?query=PRJEB8599	ERS662847	SAMEA3261851	ERX709094	ERR765821	16S rRNA	V1-V3	AGACGCACTC	AGAGTTTGATCMTGGCTCAG	GWATTACCGCGGCKGCTG	2011-07-07	46.42	-83.37	Canada	0.1	228	4.4	248	Orthic Humo-Ferric Podzol	Jack Pine, Black Spruce, Red Pine	Dfb, Humid Continental cool summer	OM2	C0	1	O horizon	46.5	35.2	1.1	4.3	9.7	32.5
JW036B	JW036	JP_ON_	Ontario	JW	Forest Soil	Whole Community DNA	PCR	Amplicon	pyrotag library	454 GS FLX Titanium	European Nucleotide Archive	https://www.ebi.ac.uk/ena/data/search?query=PRJEB8599	ERS662848	SAMEA3261852	ERX709095	ERR765822	16S rRNA	V1-V3	ATACGACGTA	AGAGTTTGATCMTGGCTCAG	GWATTACCGCGGCKGCTG	2011-07-07	46.42	-83.37	Canada	0.3	228	4.4	248	Orthic Humo-Ferric Podzol	Jack Pine, Black Spruce, Red Pine	Dfb, Humid Continental cool summer	OM2	C0	1	A horizon	12.7	2.7	0.5	5.4	9.7	11.1
JW037B	JW037	JP_ON_	Ontario	JW	Forest Soil	Whole Community DNA	PCR	Amplicon	pyrotag library	454 GS FLX Titanium	European Nucleotide Archive	https://www.ebi.ac.uk/ena/data/search?query=PRJEB8599	ERS662849	SAMEA3261853	ERX709096	ERR765823	16S rRNA	V1-V3	TGTGAGTAGT	AGAGTTTGATCMTGGCTCAG	GWATTACCGCGGCKGCTG	2011-07-07	46.42	-83.37	Canada	0.1	228	4.4	248	Orthic Humo-Ferric Podzol	Jack Pine, Black Spruce, Red Pine	Dfb, Humid Continental cool summer	OM2	C0	0	O horizon	47.5	35.2	1.1	4.3	9.7	32.5
JW038B	JW038	JP_ON_	Ontario	JW	Forest Soil	Whole Community DNA	PCR	Amplicon	pyrotag library	454 GS FLX Titanium	European Nucleotide Archive	https://www.ebi.ac.uk/ena/data/search?query=PRJEB8599	ERS662850	SAMEA3261854	ERX709097	ERR765824	16S rRNA	V1-V3	AGCGTCGTCT	AGAGTTTGATCMTGGCTCAG	GWATTACCGCGGCKGCTG	2011-07-07	46.42	-83.37	Canada	0.3	228	4.4	248	Orthic Humo-Ferric Podzol	Jack Pine, Black Spruce, Red Pine	Dfb, Humid Continental cool summer	OM2	C0	0	A horizon	12.9	2.7	0.5	5.4	9.7	11.1
JW040B	JW040	JP_ON_	Ontario	JW	Forest Soil	Whole Community DNA	PCR	Amplicon	pyrotag library	454 GS FLX Titanium	European Nucleotide Archive	https://www.ebi.ac.uk/ena/data/search?query=PRJEB8599	ERS662851	SAMEA3261855	ERX709098	ERR765825	16S rRNA	V1-V3	TACAGATCGT	AGAGTTTGATCMTGGCTCAG	GWATTACCGCGGCKGCTG	2011-07-07	46.42	-83.37	Canada	0.3	228	4.4	248	Orthic Humo-Ferric Podzol	Jack Pine, Black Spruce, Red Pine	Dfb, Humid Continental cool summer	OM3	C0	1	A horizon	19.0	1.3	0.6	5.4	9.0	9.8
JW042B	JW042	JP_ON_	Ontario	JW	Forest Soil	Whole Community DNA	PCR	Amplicon	pyrotag library	454 GS FLX Titanium	European Nucleotide Archive	https://www.ebi.ac.uk/ena/data/search?query=PRJEB8599	ERS662852	SAMEA3261856	ERX709099	ERR765826	16S rRNA	V1-V3	TACACACACT	AGAGTTTGATCMTGGCTCAG	GWATTACCGCGGCKGCTG	2011-07-07	46.42	-83.37	Canada	0.3	228	4.4	248	Orthic Humo-Ferric Podzol	Jack Pine, Black Spruce, Red Pine	Dfb, Humid Continental cool summer	OM3	C0	0	A horizon	14.3	1.3	0.6	5.4	9.0	9.8
LH001B	LH001	PP_CA_	California	LH	Forest Soil	Whole Community DNA	PCR	Amplicon	pyrotag library	454 GS FLX Titanium	European Nucleotide Archive	https://www.ebi.ac.uk/ena/data/search?query=PRJEB8599	ERS662853	SAMEA3261857	ERX709100	ERR765827	16S rRNA	V1-V3	ATACGACGTA	AGAGTTTGATCMTGGCTCAG	GWATTACCGCGGCKGCTG	2011-09-16	39.26	-120.78	USA	0.1	1268	11.2	55	Mesic Ultic Haploxeralfs	Ponderosa pine, sugar pine, white fir, giant sequoia	Csa, Mediterranean hot summer	OM1	C0	0	O horizon	14.4	44.8	1.0	3.7	NA	44.4
LH002B	LH002	PP_CA_	California	LH	Forest Soil	Whole Community DNA	PCR	Amplicon	pyrotag library	454 GS FLX Titanium	European Nucleotide Archive	https://www.ebi.ac.uk/ena/data/search?query=PRJEB8599	ERS662854	SAMEA3261858	ERX709101	ERR765828	16S rRNA	V1-V3	AGACTATACT	AGAGTTTGATCMTGGCTCAG	GWATTACCGCGGCKGCTG	2011-09-16	39.26	-120.78	USA	0.3	1268	11.2	55	Mesic Ultic Haploxeralfs	Ponderosa pine, sugar pine, white fir, giant sequoia	Csa, Mediterranean hot summer	OM1	C0	0	A horizon	18.5	4.8	0.2	5.2	NA	29.6
LH003B	LH003	PP_CA_	California	LH	Forest Soil	Whole Community DNA	PCR	Amplicon	pyrotag library	454 GS FLX Titanium	European Nucleotide Archive	https://www.ebi.ac.uk/ena/data/search?query=PRJEB8599	ERS662855	SAMEA3261859	ERX709102	ERR765829	16S rRNA	V1-V3	TAGTGTAGAT	AGAGTTTGATCMTGGCTCAG	GWATTACCGCGGCKGCTG	2011-09-16	39.26	-120.78	USA	0.1	1268	11.2	55	Mesic Ultic Haploxeralfs	Ponderosa pine, sugar pine, white fir, giant sequoia	Csa, Mediterranean hot summer	OM1	C0	0	O horizon	17.2	43.3	0.9	3.6	NA	46.4
LH004B	LH004	PP_CA_	California	LH	Forest Soil	Whole Community DNA	PCR	Amplicon	pyrotag library	454 GS FLX Titanium	European Nucleotide Archive	https://www.ebi.ac.uk/ena/data/search?query=PRJEB8599	ERS662856	SAMEA3261860	ERX709103	ERR765830	16S rRNA	V1-V3	CGACGTGACT	AGAGTTTGATCMTGGCTCAG	GWATTACCGCGGCKGCTG	2011-09-16	39.26	-120.78	USA	0.3	1268	11.2	55	Mesic Ultic Haploxeralfs	Ponderosa pine, sugar pine, white fir, giant sequoia	Csa, Mediterranean hot summer	OM1	C0	0	A horizon	34.1	6.5	0.2	5.3	NA	32.8
LH005B	LH005	PP_CA_	California	LH	Forest Soil	Whole Community DNA	PCR	Amplicon	pyrotag library	454 GS FLX Titanium	European Nucleotide Archive	https://www.ebi.ac.uk/ena/data/search?query=PRJEB8599	ERS662857	SAMEA3261861	ERX709104	ERR765831	16S rRNA	V1-V3	AGACTATACT	AGAGTTTGATCMTGGCTCAG	GWATTACCGCGGCKGCTG	2011-09-16	39.26	-120.78	USA	0.1	1268	11.2	55	Mesic Ultic Haploxeralfs	Ponderosa pine, sugar pine, white fir, giant sequoia	Csa, Mediterranean hot summer	OM1	C0	0	O horizon	14.7	40.4	1.1	3.9	NA	35.4
LH006B	LH006	PP_CA_	California	LH	Forest Soil	Whole Community DNA	PCR	Amplicon	pyrotag library	454 GS FLX Titanium	European Nucleotide Archive	https://www.ebi.ac.uk/ena/data/search?query=PRJEB8599	ERS662858	SAMEA3261862	ERX709105	ERR765832	16S rRNA	V1-V3	CATAGTAGTG	AGAGTTTGATCMTGGCTCAG	GWATTACCGCGGCKGCTG	2011-09-16	39.26	-120.78	USA	0.3	1268	11.2	55	Mesic Ultic Haploxeralfs	Ponderosa pine, sugar pine, white fir, giant sequoia	Csa, Mediterranean hot summer	OM1	C0	0	A horizon	19.0	4.8	0.2	5.5	NA	25.2
LH007B	LH007	PP_CA_	California	LH	Forest Soil	Whole Community DNA	PCR	Amplicon	pyrotag library	454 GS FLX Titanium	European Nucleotide Archive	https://www.ebi.ac.uk/ena/data/search?query=PRJEB8599	ERS662859	SAMEA3261863	ERX709106	ERR765833	16S rRNA	V1-V3	AGACTATACT	AGAGTTTGATCMTGGCTCAG	GWATTACCGCGGCKGCTG	2011-09-16	39.26	-120.78	USA	0.1	1268	11.2	55	Mesic Ultic Haploxeralfs	Ponderosa pine, sugar pine, white fir, giant sequoia	Csa, Mediterranean hot summer	OM2	C0	0	O horizon	10.3	25.8	0.8	4.6	NA	34.2
LH008B	LH008	PP_CA_	California	LH	Forest Soil	Whole Community DNA	PCR	Amplicon	pyrotag library	454 GS FLX Titanium	European Nucleotide Archive	https://www.ebi.ac.uk/ena/data/search?query=PRJEB8599	ERS662860	SAMEA3261864	ERX709107	ERR765834	16S rRNA	V1-V3	ACTAGCAGTA	AGAGTTTGATCMTGGCTCAG	GWATTACCGCGGCKGCTG	2011-09-16	39.26	-120.78	USA	0.3	1268	11.2	55	Mesic Ultic Haploxeralfs	Ponderosa pine, sugar pine, white fir, giant sequoia	Csa, Mediterranean hot summer	OM2	C0	0	A horizon	17.1	3.6	0.2	6.0	NA	22.3
LH009B	LH009	PP_CA_	California	LH	Forest Soil	Whole Community DNA	PCR	Amplicon	pyrotag library	454 GS FLX Titanium	European Nucleotide Archive	https://www.ebi.ac.uk/ena/data/search?query=PRJEB8599	ERS662861	SAMEA3261865	ERX709108	ERR765835	16S rRNA	V1-V3	CACGCTACGT	AGAGTTTGATCMTGGCTCAG	GWATTACCGCGGCKGCTG	2011-09-16	39.26	-120.78	USA	0.1	1268	11.2	55	Mesic Ultic Haploxeralfs	Ponderosa pine, sugar pine, white fir, giant sequoia	Csa, Mediterranean hot summer	OM2	C0	0	O horizon	33.7	33.8	1.0	4.5	NA	33.6
LH010B	LH010	PP_CA_	California	LH	Forest Soil	Whole Community DNA	PCR	Amplicon	pyrotag library	454 GS FLX Titanium	European Nucleotide Archive	https://www.ebi.ac.uk/ena/data/search?query=PRJEB8599	ERS662862	SAMEA3261866	ERX709109	ERR765836	16S rRNA	V1-V3	ATACGACGTA	AGAGTTTGATCMTGGCTCAG	GWATTACCGCGGCKGCTG	2011-09-16	39.26	-120.78	USA	0.3	1268	11.2	55	Mesic Ultic Haploxeralfs	Ponderosa pine, sugar pine, white fir, giant sequoia	Csa, Mediterranean hot summer	OM2	C0	0	A horizon	18.3	3.6	0.2	5.9	NA	22.3
LH011B	LH011	PP_CA_	California	LH	Forest Soil	Whole Community DNA	PCR	Amplicon	pyrotag library	454 GS FLX Titanium	European Nucleotide Archive	https://www.ebi.ac.uk/ena/data/search?query=PRJEB8599	ERS662863	SAMEA3261867	ERX709110	ERR765837	16S rRNA	V1-V3	AGCACTGTAG	AGAGTTTGATCMTGGCTCAG	GWATTACCGCGGCKGCTG	2011-09-16	39.26	-120.78	USA	0.1	1268	11.2	55	Mesic Ultic Haploxeralfs	Ponderosa pine, sugar pine, white fir, giant sequoia	Csa, Mediterranean hot summer	OM2	C0	0	O horizon	6.7	29.1	0.8	4.2	NA	34.8
LH012B	LH012	PP_CA_	California	LH	Forest Soil	Whole Community DNA	PCR	Amplicon	pyrotag library	454 GS FLX Titanium	European Nucleotide Archive	https://www.ebi.ac.uk/ena/data/search?query=PRJEB8599	ERS662864	SAMEA3261868	ERX709111	ERR765838	16S rRNA	V1-V3	CGACGTGACT	AGAGTTTGATCMTGGCTCAG	GWATTACCGCGGCKGCTG	2011-09-16	39.26	-120.78	USA	0.3	1268	11.2	55	Mesic Ultic Haploxeralfs	Ponderosa pine, sugar pine, white fir, giant sequoia	Csa, Mediterranean hot summer	OM2	C0	0	A horizon	17.7	3.6	0.2	5.7	NA	22.3
LH013B	LH013	PP_CA_	California	LH	Forest Soil	Whole Community DNA	PCR	Amplicon	pyrotag library	454 GS FLX Titanium	European Nucleotide Archive	https://www.ebi.ac.uk/ena/data/search?query=PRJEB8599	ERS662865	SAMEA3261869	ERX709112	ERR765839	16S rRNA	V1-V3	TCTAGCGACT	AGAGTTTGATCMTGGCTCAG	GWATTACCGCGGCKGCTG	2011-09-16	39.26	-120.78	USA	0.1	1268	11.2	55	Mesic Ultic Haploxeralfs	Ponderosa pine, sugar pine, white fir, giant sequoia	Csa, Mediterranean hot summer	OM3	C0	0	O horizon	14.1	36.4	1.1	5.1	NA	34.1
LH014B	LH014	PP_CA_	California	LH	Forest Soil	Whole Community DNA	PCR	Amplicon	pyrotag library	454 GS FLX Titanium	European Nucleotide Archive	https://www.ebi.ac.uk/ena/data/search?query=PRJEB8599	ERS662866	SAMEA3261870	ERX709113	ERR765840	16S rRNA	V1-V3	ACTGTACAGT	AGAGTTTGATCMTGGCTCAG	GWATTACCGCGGCKGCTG	2011-09-16	39.26	-120.78	USA	0.3	1268	11.2	55	Mesic Ultic Haploxeralfs	Ponderosa pine, sugar pine, white fir, giant sequoia	Csa, Mediterranean hot summer	OM3	C0	0	A horizon	17.1	3.9	0.2	5.0	NA	21.6
LH015B	LH015	PP_CA_	California	LH	Forest Soil	Whole Community DNA	PCR	Amplicon	pyrotag library	454 GS FLX Titanium	European Nucleotide Archive	https://www.ebi.ac.uk/ena/data/search?query=PRJEB8599	ERS662867	SAMEA3261871	ERX709114	ERR765841	16S rRNA	V1-V3	ACGAGTGCGT	AGAGTTTGATCMTGGCTCAG	GWATTACCGCGGCKGCTG	2011-09-16	39.26	-120.78	USA	0.1	1268	11.2	55	Mesic Ultic Haploxeralfs	Ponderosa pine, sugar pine, white fir, giant sequoia	Csa, Mediterranean hot summer	OM3	C0	0	O horizon	14.0	NA	NA	5.8	NA	NA
LH016B	LH016	PP_CA_	California	LH	Forest Soil	Whole Community DNA	PCR	Amplicon	pyrotag library	454 GS FLX Titanium	European Nucleotide Archive	https://www.ebi.ac.uk/ena/data/search?query=PRJEB8599	ERS662868	SAMEA3261872	ERX709115	ERR765842	16S rRNA	V1-V3	TACAGATCGT	AGAGTTTGATCMTGGCTCAG	GWATTACCGCGGCKGCTG	2011-09-16	39.26	-120.78	USA	0.3	1268	11.2	55	Mesic Ultic Haploxeralfs	Ponderosa pine, sugar pine, white fir, giant sequoia	Csa, Mediterranean hot summer	OM3	C0	0	A horizon	17.1	4.3	0.2	6.3	NA	19.2
LH017B	LH017	PP_CA_	California	LH	Forest Soil	Whole Community DNA	PCR	Amplicon	pyrotag library	454 GS FLX Titanium	European Nucleotide Archive	https://www.ebi.ac.uk/ena/data/search?query=PRJEB8599	ERS662869	SAMEA3261873	ERX709116	ERR765843	16S rRNA	V1-V3	ACGCGATCGA	AGAGTTTGATCMTGGCTCAG	GWATTACCGCGGCKGCTG	2011-09-16	39.26	-120.78	USA	0.1	1268	11.2	55	Mesic Ultic Haploxeralfs	Ponderosa pine, sugar pine, white fir, giant sequoia	Csa, Mediterranean hot summer	OM3	C0	0	O horizon	14.0	39.5	0.9	5.0	NA	42.1
LH018B	LH018	PP_CA_	California	LH	Forest Soil	Whole Community DNA	PCR	Amplicon	pyrotag library	454 GS FLX Titanium	European Nucleotide Archive	https://www.ebi.ac.uk/ena/data/search?query=PRJEB8599	ERS662870	SAMEA3261874	ERX709117	ERR765844	16S rRNA	V1-V3	TGATACGTCT	AGAGTTTGATCMTGGCTCAG	GWATTACCGCGGCKGCTG	2011-09-16	39.26	-120.78	USA	0.3	1268	11.2	55	Mesic Ultic Haploxeralfs	Ponderosa pine, sugar pine, white fir, giant sequoia	Csa, Mediterranean hot summer	OM3	C0	0	A horizon	16.4	3.6	0.2	6.0	NA	20.3
LH019B	LH019	PP_CA_	California	LH	Forest Soil	Whole Community DNA	PCR	Amplicon	pyrotag library	454 GS FLX Titanium	European Nucleotide Archive	https://www.ebi.ac.uk/ena/data/search?query=PRJEB8599	ERS662871	SAMEA3261875	ERX709118	ERR765845	16S rRNA	V1-V3	TCTACGTAGC	AGAGTTTGATCMTGGCTCAG	GWATTACCGCGGCKGCTG	2011-09-16	39.26	-120.78	USA	0.1	1268	11.2	55	Mesic Ultic Haploxeralfs	Ponderosa pine, sugar pine, white fir, giant sequoia	Csa, Mediterranean hot summer	REF	REF	0	O horizon	21.7	39.7	1.3	5.6	NA	31.0
LH020B	LH020	PP_CA_	California	LH	Forest Soil	Whole Community DNA	PCR	Amplicon	pyrotag library	454 GS FLX Titanium	European Nucleotide Archive	https://www.ebi.ac.uk/ena/data/search?query=PRJEB8599	ERS662872	SAMEA3261876	ERX709119	ERR765846	16S rRNA	V1-V3	ACTAGCAGTA	AGAGTTTGATCMTGGCTCAG	GWATTACCGCGGCKGCTG	2011-09-16	39.26	-120.78	USA	0.3	1268	11.2	55	Mesic Ultic Haploxeralfs	Ponderosa pine, sugar pine, white fir, giant sequoia	Csa, Mediterranean hot summer	REF	REF	0	A horizon	20.1	4.1	0.2	6.6	NA	19.1
LH021B	LH021	PP_CA_	California	LH	Forest Soil	Whole Community DNA	PCR	Amplicon	pyrotag library	454 GS FLX Titanium	European Nucleotide Archive	https://www.ebi.ac.uk/ena/data/search?query=PRJEB8599	ERS662873	SAMEA3261877	ERX709120	ERR765847	16S rRNA	V1-V3	ACGCTCGACA	AGAGTTTGATCMTGGCTCAG	GWATTACCGCGGCKGCTG	2011-09-16	39.26	-120.78	USA	0.1	1268	11.2	55	Mesic Ultic Haploxeralfs	Ponderosa pine, sugar pine, white fir, giant sequoia	Csa, Mediterranean hot summer	REF	REF	0	O horizon	13.8	31.3	0.9	4.9	NA	35.1
LH022B	LH022	PP_CA_	California	LH	Forest Soil	Whole Community DNA	PCR	Amplicon	pyrotag library	454 GS FLX Titanium	European Nucleotide Archive	https://www.ebi.ac.uk/ena/data/search?query=PRJEB8599	ERS662874	SAMEA3261878	ERX709121	ERR765848	16S rRNA	V1-V3	AGACGCACTC	AGAGTTTGATCMTGGCTCAG	GWATTACCGCGGCKGCTG	2011-09-16	39.26	-120.78	USA	0.3	1268	11.2	55	Mesic Ultic Haploxeralfs	Ponderosa pine, sugar pine, white fir, giant sequoia	Csa, Mediterranean hot summer	REF	REF	0	A horizon	18.7	4.6	0.2	6.1	NA	21.6
LH023B	LH023	PP_CA_	California	LH	Forest Soil	Whole Community DNA	PCR	Amplicon	pyrotag library	454 GS FLX Titanium	European Nucleotide Archive	https://www.ebi.ac.uk/ena/data/search?query=PRJEB8599	ERS662875	SAMEA3261879	ERX709122	ERR765849	16S rRNA	V1-V3	ACGAGTGCGT	AGAGTTTGATCMTGGCTCAG	GWATTACCGCGGCKGCTG	2011-09-16	39.26	-120.78	USA	0.1	1268	11.2	55	Mesic Ultic Haploxeralfs	Ponderosa pine, sugar pine, white fir, giant sequoia	Csa, Mediterranean hot summer	REF	REF	0	O horizon	13.0	43.0	1.3	5.7	NA	32.0
LH024B	LH024	PP_CA_	California	LH	Forest Soil	Whole Community DNA	PCR	Amplicon	pyrotag library	454 GS FLX Titanium	European Nucleotide Archive	https://www.ebi.ac.uk/ena/data/search?query=PRJEB8599	ERS662876	SAMEA3261880	ERX709123	ERR765850	16S rRNA	V1-V3	TAGTGTAGAT	AGAGTTTGATCMTGGCTCAG	GWATTACCGCGGCKGCTG	2011-09-16	39.26	-120.78	USA	0.3	1268	11.2	55	Mesic Ultic Haploxeralfs	Ponderosa pine, sugar pine, white fir, giant sequoia	Csa, Mediterranean hot summer	REF	REF	0	A horizon	18.9	5.6	0.3	6.5	NA	22.5
TXA001B	TXA001	LP_TX_	Texas	TXA	Forest Soil	Whole Community DNA	PCR	Amplicon	pyrotag library	454 GS FLX Titanium	European Nucleotide Archive	https://www.ebi.ac.uk/ena/data/search?query=PRJEB8599	ERS662877	SAMEA3261881	ERX709124	ERR765851	16S rRNA	V1-V3	CGTGTCTCTA	AGAGTTTGATCMTGGCTCAG	GWATTACCGCGGCKGCTG	2012-03-12	31.11	-95.15	USA	0.3	88	19.0	253	Aquic Glossudalfs	Loblolly Pine, Beautyberry, Yaupon, Sweetgum, Oaks, Wax Myrtle	Cfa, Humid subtropical	OM1	C0	0	A horizon	12.2	1.1	0.1	5.0	1.3	18.5
TXA002B	TXA002	LP_TX_	Texas	TXA	Forest Soil	Whole Community DNA	PCR	Amplicon	pyrotag library	454 GS FLX Titanium	European Nucleotide Archive	https://www.ebi.ac.uk/ena/data/search?query=PRJEB8599	ERS662878	SAMEA3261882	ERX709125	ERR765852	16S rRNA	V1-V3	TGACGTATGT	AGAGTTTGATCMTGGCTCAG	GWATTACCGCGGCKGCTG	2012-03-12	31.11	-95.15	USA	0.3	88	19.0	253	Aquic Glossudalfs	Loblolly Pine, Beautyberry, Yaupon, Sweetgum, Oaks, Wax Myrtle	Cfa, Humid subtropical	OM1	C0	0	A horizon	13.9	1.1	0.1	4.7	1.3	18.5
TXA003B	TXA003	LP_TX_	Texas	TXA	Forest Soil	Whole Community DNA	PCR	Amplicon	pyrotag library	454 GS FLX Titanium	European Nucleotide Archive	https://www.ebi.ac.uk/ena/data/search?query=PRJEB8599	ERS662879	SAMEA3261883	ERX709126	ERR765853	16S rRNA	V1-V3	ATATCGCGAG	AGAGTTTGATCMTGGCTCAG	GWATTACCGCGGCKGCTG	2012-03-12	31.11	-95.15	USA	0.3	88	19.0	253	Aquic Glossudalfs	Loblolly Pine, Beautyberry, Yaupon, Sweetgum, Oaks, Wax Myrtle	Cfa, Humid subtropical	OM1	C0	0	A horizon	13.8	1.1	0.1	4.6	1.3	18.5
TXA004B	TXA004	LP_TX_	Texas	TXA	Forest Soil	Whole Community DNA	PCR	Amplicon	pyrotag library	454 GS FLX Titanium	European Nucleotide Archive	https://www.ebi.ac.uk/ena/data/search?query=PRJEB8599	ERS662880	SAMEA3261884	ERX709127	ERR765854	16S rRNA	V1-V3	TCGCACTAGT	AGAGTTTGATCMTGGCTCAG	GWATTACCGCGGCKGCTG	2012-03-12	31.11	-95.15	USA	0.1	88	19.0	253	Aquic Glossudalfs	Loblolly Pine, Beautyberry, Yaupon, Sweetgum, Oaks, Wax Myrtle	Cfa, Humid subtropical	OM1	C0	0	O horizon	25.5	12.6	0.5	4.7	NA	26.7
TXA005B	TXA005	LP_TX_	Texas	TXA	Forest Soil	Whole Community DNA	PCR	Amplicon	pyrotag library	454 GS FLX Titanium	European Nucleotide Archive	https://www.ebi.ac.uk/ena/data/search?query=PRJEB8599	ERS662881	SAMEA3261885	ERX709128	ERR765855	16S rRNA	V1-V3	TACAGATCGT	AGAGTTTGATCMTGGCTCAG	GWATTACCGCGGCKGCTG	2012-03-12	31.11	-95.15	USA	0.1	88	19.0	253	Aquic Glossudalfs	Loblolly Pine, Beautyberry, Yaupon, Sweetgum, Oaks, Wax Myrtle	Cfa, Humid subtropical	OM1	C0	0	O horizon	27.8	11.5	0.4	4.7	NA	28.7
TXA006B	TXA006	LP_TX_	Texas	TXA	Forest Soil	Whole Community DNA	PCR	Amplicon	pyrotag library	454 GS FLX Titanium	European Nucleotide Archive	https://www.ebi.ac.uk/ena/data/search?query=PRJEB8599	ERS662882	SAMEA3261886	ERX709129	ERR765856	16S rRNA	V1-V3	ACAGTATATA	AGAGTTTGATCMTGGCTCAG	GWATTACCGCGGCKGCTG	2012-03-12	31.11	-95.15	USA	0.1	88	19.0	253	Aquic Glossudalfs	Loblolly Pine, Beautyberry, Yaupon, Sweetgum, Oaks, Wax Myrtle	Cfa, Humid subtropical	OM1	C0	0	O horizon	19.0	8.8	0.4	5.0	NA	23.7
TXA007B	TXA007	LP_TX_	Texas	TXA	Forest Soil	Whole Community DNA	PCR	Amplicon	pyrotag library	454 GS FLX Titanium	European Nucleotide Archive	https://www.ebi.ac.uk/ena/data/search?query=PRJEB8599	ERS662883	SAMEA3261887	ERX709130	ERR765857	16S rRNA	V1-V3	TCTATACTAT	AGAGTTTGATCMTGGCTCAG	GWATTACCGCGGCKGCTG	2012-03-12	31.11	-95.15	USA	0.3	88	19.0	253	Aquic Glossudalfs	Loblolly Pine, Beautyberry, Yaupon, Sweetgum, Oaks, Wax Myrtle	Cfa, Humid subtropical	OM1	C0	1	A horizon	11.6	1.1	0.1	4.7	1.3	18.5
TXA008B	TXA008	LP_TX_	Texas	TXA	Forest Soil	Whole Community DNA	PCR	Amplicon	pyrotag library	454 GS FLX Titanium	European Nucleotide Archive	https://www.ebi.ac.uk/ena/data/search?query=PRJEB8599	ERS662884	SAMEA3261888	ERX709131	ERR765858	16S rRNA	V1-V3	AGACTATACT	AGAGTTTGATCMTGGCTCAG	GWATTACCGCGGCKGCTG	2012-03-12	31.11	-95.15	USA	0.3	88	19.0	253	Aquic Glossudalfs	Loblolly Pine, Beautyberry, Yaupon, Sweetgum, Oaks, Wax Myrtle	Cfa, Humid subtropical	OM1	C0	1	A horizon	23.1	1.1	0.1	4.8	1.3	18.5
TXA009B	TXA009	LP_TX_	Texas	TXA	Forest Soil	Whole Community DNA	PCR	Amplicon	pyrotag library	454 GS FLX Titanium	European Nucleotide Archive	https://www.ebi.ac.uk/ena/data/search?query=PRJEB8599	ERS662885	SAMEA3261889	ERX709132	ERR765859	16S rRNA	V1-V3	CGAGAGATAC	AGAGTTTGATCMTGGCTCAG	GWATTACCGCGGCKGCTG	2012-03-12	31.11	-95.15	USA	0.3	88	19.0	253	Aquic Glossudalfs	Loblolly Pine, Beautyberry, Yaupon, Sweetgum, Oaks, Wax Myrtle	Cfa, Humid subtropical	OM1	C0	1	A horizon	12.6	1.1	0.1	5.1	1.3	18.5
TXA010B	TXA010	LP_TX_	Texas	TXA	Forest Soil	Whole Community DNA	PCR	Amplicon	pyrotag library	454 GS FLX Titanium	European Nucleotide Archive	https://www.ebi.ac.uk/ena/data/search?query=PRJEB8599	ERS662886	SAMEA3261890	ERX709133	ERR765860	16S rRNA	V1-V3	ATAGAGTACT	AGAGTTTGATCMTGGCTCAG	GWATTACCGCGGCKGCTG	2012-03-12	31.11	-95.15	USA	0.1	88	19.0	253	Aquic Glossudalfs	Loblolly Pine, Beautyberry, Yaupon, Sweetgum, Oaks, Wax Myrtle	Cfa, Humid subtropical	OM1	C0	1	O horizon	31.9	NA	NA	4.8	NA	NA
TXA011B	TXA011	LP_TX_	Texas	TXA	Forest Soil	Whole Community DNA	PCR	Amplicon	pyrotag library	454 GS FLX Titanium	European Nucleotide Archive	https://www.ebi.ac.uk/ena/data/search?query=PRJEB8599	ERS662887	SAMEA3261891	ERX709134	ERR765861	16S rRNA	V1-V3	TCTATACTAT	AGAGTTTGATCMTGGCTCAG	GWATTACCGCGGCKGCTG	2012-03-12	31.11	-95.15	USA	0.1	88	19.0	253	Aquic Glossudalfs	Loblolly Pine, Beautyberry, Yaupon, Sweetgum, Oaks, Wax Myrtle	Cfa, Humid subtropical	OM1	C0	1	O horizon	31.0	NA	NA	4.3	NA	NA
TXA012B	TXA012	LP_TX_	Texas	TXA	Forest Soil	Whole Community DNA	PCR	Amplicon	pyrotag library	454 GS FLX Titanium	European Nucleotide Archive	https://www.ebi.ac.uk/ena/data/search?query=PRJEB8599	ERS662888	SAMEA3261892	ERX709135	ERR765862	16S rRNA	V1-V3	TACACGTGAT	AGAGTTTGATCMTGGCTCAG	GWATTACCGCGGCKGCTG	2012-03-12	31.11	-95.15	USA	0.1	88	19.0	253	Aquic Glossudalfs	Loblolly Pine, Beautyberry, Yaupon, Sweetgum, Oaks, Wax Myrtle	Cfa, Humid subtropical	OM1	C0	1	O horizon	19.7	NA	NA	4.8	NA	NA
TXA013B	TXA013	LP_TX_	Texas	TXA	Forest Soil	Whole Community DNA	PCR	Amplicon	pyrotag library	454 GS FLX Titanium	European Nucleotide Archive	https://www.ebi.ac.uk/ena/data/search?query=PRJEB8599	ERS662889	SAMEA3261893	ERX709136	ERR765863	16S rRNA	V1-V3	ACTAGCAGTA	AGAGTTTGATCMTGGCTCAG	GWATTACCGCGGCKGCTG	2012-03-12	31.11	-95.15	USA	0.3	88	19.0	253	Aquic Glossudalfs	Loblolly Pine, Beautyberry, Yaupon, Sweetgum, Oaks, Wax Myrtle	Cfa, Humid subtropical	OM2	C0	0	A horizon	10.5	1.2	0.1	4.6	1.3	23.9
TXA014B	TXA014	LP_TX_	Texas	TXA	Forest Soil	Whole Community DNA	PCR	Amplicon	pyrotag library	454 GS FLX Titanium	European Nucleotide Archive	https://www.ebi.ac.uk/ena/data/search?query=PRJEB8599	ERS662890	SAMEA3261894	ERX709137	ERR765864	16S rRNA	V1-V3	TGTGAGTAGT	AGAGTTTGATCMTGGCTCAG	GWATTACCGCGGCKGCTG	2012-03-12	31.11	-95.15	USA	0.3	88	19.0	253	Aquic Glossudalfs	Loblolly Pine, Beautyberry, Yaupon, Sweetgum, Oaks, Wax Myrtle	Cfa, Humid subtropical	OM2	C0	0	A horizon	9.9	1.2	0.1	5.0	1.3	23.9
TXA015B	TXA015	LP_TX_	Texas	TXA	Forest Soil	Whole Community DNA	PCR	Amplicon	pyrotag library	454 GS FLX Titanium	European Nucleotide Archive	https://www.ebi.ac.uk/ena/data/search?query=PRJEB8599	ERS662891	SAMEA3261895	ERX709138	ERR765865	16S rRNA	V1-V3	TCACGTACTA	AGAGTTTGATCMTGGCTCAG	GWATTACCGCGGCKGCTG	2012-03-12	31.11	-95.15	USA	0.3	88	19.0	253	Aquic Glossudalfs	Loblolly Pine, Beautyberry, Yaupon, Sweetgum, Oaks, Wax Myrtle	Cfa, Humid subtropical	OM2	C0	0	A horizon	8.9	1.2	0.1	4.3	1.3	23.9
TXA016B	TXA016	LP_TX_	Texas	TXA	Forest Soil	Whole Community DNA	PCR	Amplicon	pyrotag library	454 GS FLX Titanium	European Nucleotide Archive	https://www.ebi.ac.uk/ena/data/search?query=PRJEB8599	ERS662892	SAMEA3261896	ERX709139	ERR765866	16S rRNA	V1-V3	AGACGCACTC	AGAGTTTGATCMTGGCTCAG	GWATTACCGCGGCKGCTG	2012-03-12	31.11	-95.15	USA	0.1	88	19.0	253	Aquic Glossudalfs	Loblolly Pine, Beautyberry, Yaupon, Sweetgum, Oaks, Wax Myrtle	Cfa, Humid subtropical	OM2	C0	0	O horizon	15.0	22.6	0.8	4.5	NA	28.0
TXA017B	TXA017	LP_TX_	Texas	TXA	Forest Soil	Whole Community DNA	PCR	Amplicon	pyrotag library	454 GS FLX Titanium	European Nucleotide Archive	https://www.ebi.ac.uk/ena/data/search?query=PRJEB8599	ERS662893	SAMEA3261897	ERX709140	ERR765867	16S rRNA	V1-V3	TACGCTGTCT	AGAGTTTGATCMTGGCTCAG	GWATTACCGCGGCKGCTG	2012-03-12	31.11	-95.15	USA	0.1	88	19.0	253	Aquic Glossudalfs	Loblolly Pine, Beautyberry, Yaupon, Sweetgum, Oaks, Wax Myrtle	Cfa, Humid subtropical	OM2	C0	0	O horizon	23.3	13.1	0.6	5.7	NA	23.2
TXA018B	TXA018	LP_TX_	Texas	TXA	Forest Soil	Whole Community DNA	PCR	Amplicon	pyrotag library	454 GS FLX Titanium	European Nucleotide Archive	https://www.ebi.ac.uk/ena/data/search?query=PRJEB8599	ERS662894	SAMEA3261898	ERX709141	ERR765868	16S rRNA	V1-V3	TCGCACTAGT	AGAGTTTGATCMTGGCTCAG	GWATTACCGCGGCKGCTG	2012-03-12	31.11	-95.15	USA	0.1	88	19.0	253	Aquic Glossudalfs	Loblolly Pine, Beautyberry, Yaupon, Sweetgum, Oaks, Wax Myrtle	Cfa, Humid subtropical	OM2	C0	0	O horizon	40	NA	NA	5.28	NA	NA
TXA019B	TXA019	LP_TX_	Texas	TXA	Forest Soil	Whole Community DNA	PCR	Amplicon	pyrotag library	454 GS FLX Titanium	European Nucleotide Archive	https://www.ebi.ac.uk/ena/data/search?query=PRJEB8599	ERS662895	SAMEA3261899	ERX709142	ERR765869	16S rRNA	V1-V3	TACACGTGAT	AGAGTTTGATCMTGGCTCAG	GWATTACCGCGGCKGCTG	2012-03-12	31.11	-95.15	USA	0.3	88	19.0	253	Aquic Glossudalfs	Loblolly Pine, Beautyberry, Yaupon, Sweetgum, Oaks, Wax Myrtle	Cfa, Humid subtropical	OM2	C0	1	A horizon	9.9	1.2	0.1	4.6	1.3	23.9
TXA021B	TXA021	LP_TX_	Texas	TXA	Forest Soil	Whole Community DNA	PCR	Amplicon	pyrotag library	454 GS FLX Titanium	European Nucleotide Archive	https://www.ebi.ac.uk/ena/data/search?query=PRJEB8599	ERS662896	SAMEA3261900	ERX709143	ERR765870	16S rRNA	V1-V3	ATATCGCGAG	AGAGTTTGATCMTGGCTCAG	GWATTACCGCGGCKGCTG	2012-03-12	31.11	-95.15	USA	0.3	88	19.0	253	Aquic Glossudalfs	Loblolly Pine, Beautyberry, Yaupon, Sweetgum, Oaks, Wax Myrtle	Cfa, Humid subtropical	OM2	C0	1	A horizon	8.6	1.2	0.1	5.1	1.3	23.9
TXA022B	TXA022	LP_TX_	Texas	TXA	Forest Soil	Whole Community DNA	PCR	Amplicon	pyrotag library	454 GS FLX Titanium	European Nucleotide Archive	https://www.ebi.ac.uk/ena/data/search?query=PRJEB8599	ERS662897	SAMEA3261901	ERX709144	ERR765871	16S rRNA	V1-V3	CGTCTAGTAC	AGAGTTTGATCMTGGCTCAG	GWATTACCGCGGCKGCTG	2012-03-12	31.11	-95.15	USA	0.1	88	19.0	253	Aquic Glossudalfs	Loblolly Pine, Beautyberry, Yaupon, Sweetgum, Oaks, Wax Myrtle	Cfa, Humid subtropical	OM2	C0	1	O horizon	13.4	NA	NA	4.5	NA	NA
TXA023B	TXA023	LP_TX_	Texas	TXA	Forest Soil	Whole Community DNA	PCR	Amplicon	pyrotag library	454 GS FLX Titanium	European Nucleotide Archive	https://www.ebi.ac.uk/ena/data/search?query=PRJEB8599	ERS662898	SAMEA3261902	ERX709145	ERR765872	16S rRNA	V1-V3	CATAGTAGTG	AGAGTTTGATCMTGGCTCAG	GWATTACCGCGGCKGCTG	2012-03-12	31.11	-95.15	USA	0.1	88	19.0	253	Aquic Glossudalfs	Loblolly Pine, Beautyberry, Yaupon, Sweetgum, Oaks, Wax Myrtle	Cfa, Humid subtropical	OM2	C0	1	O horizon	24.6	NA	NA	4.1	NA	NA
TXA024B	TXA024	LP_TX_	Texas	TXA	Forest Soil	Whole Community DNA	PCR	Amplicon	pyrotag library	454 GS FLX Titanium	European Nucleotide Archive	https://www.ebi.ac.uk/ena/data/search?query=PRJEB8599	ERS662899	SAMEA3261903	ERX709146	ERR765873	16S rRNA	V1-V3	TAGTGTAGAT	AGAGTTTGATCMTGGCTCAG	GWATTACCGCGGCKGCTG	2012-03-12	31.11	-95.15	USA	0.1	88	19.0	253	Aquic Glossudalfs	Loblolly Pine, Beautyberry, Yaupon, Sweetgum, Oaks, Wax Myrtle	Cfa, Humid subtropical	OM2	C0	1	O horizon	52.8	NA	NA	4.6	NA	NA
TXA025B	TXA025	LP_TX_	Texas	TXA	Forest Soil	Whole Community DNA	PCR	Amplicon	pyrotag library	454 GS FLX Titanium	European Nucleotide Archive	https://www.ebi.ac.uk/ena/data/search?query=PRJEB8599	ERS662900	SAMEA3261904	ERX709147	ERR765874	16S rRNA	V1-V3	ACGCGAGTAT	AGAGTTTGATCMTGGCTCAG	GWATTACCGCGGCKGCTG	2012-03-12	31.11	-95.15	USA	0.3	88	19.0	253	Aquic Glossudalfs	Loblolly Pine, Beautyberry, Yaupon, Sweetgum, Oaks, Wax Myrtle	Cfa, Humid subtropical	OM3	C0	0	A horizon	8.9	0.8	0.0	4.6	1.3	17.8
TXA026B	TXA026	LP_TX_	Texas	TXA	Forest Soil	Whole Community DNA	PCR	Amplicon	pyrotag library	454 GS FLX Titanium	European Nucleotide Archive	https://www.ebi.ac.uk/ena/data/search?query=PRJEB8599	ERS662901	SAMEA3261905	ERX709148	ERR765875	16S rRNA	V1-V3	TGACGTATGT	AGAGTTTGATCMTGGCTCAG	GWATTACCGCGGCKGCTG	2012-03-12	31.11	-95.15	USA	0.3	88	19.0	253	Aquic Glossudalfs	Loblolly Pine, Beautyberry, Yaupon, Sweetgum, Oaks, Wax Myrtle	Cfa, Humid subtropical	OM3	C0	0	A horizon	8.6	0.8	0.0	4.5	1.3	17.8
TXA027B	TXA027	LP_TX_	Texas	TXA	Forest Soil	Whole Community DNA	PCR	Amplicon	pyrotag library	454 GS FLX Titanium	European Nucleotide Archive	https://www.ebi.ac.uk/ena/data/search?query=PRJEB8599	ERS662902	SAMEA3261906	ERX709149	ERR765876	16S rRNA	V1-V3	TACGCTGTCT	AGAGTTTGATCMTGGCTCAG	GWATTACCGCGGCKGCTG	2012-03-12	31.11	-95.15	USA	0.3	88	19.0	253	Aquic Glossudalfs	Loblolly Pine, Beautyberry, Yaupon, Sweetgum, Oaks, Wax Myrtle	Cfa, Humid subtropical	OM3	C0	0	A horizon	9.5	0.8	0.0	4.4	1.3	17.8
TXA028B	TXA028	LP_TX_	Texas	TXA	Forest Soil	Whole Community DNA	PCR	Amplicon	pyrotag library	454 GS FLX Titanium	European Nucleotide Archive	https://www.ebi.ac.uk/ena/data/search?query=PRJEB8599	ERS662903	SAMEA3261907	ERX709150	ERR765877	16S rRNA	V1-V3	TCTATACTAT	AGAGTTTGATCMTGGCTCAG	GWATTACCGCGGCKGCTG	2012-03-12	31.11	-95.15	USA	0.1	88	19.0	253	Aquic Glossudalfs	Loblolly Pine, Beautyberry, Yaupon, Sweetgum, Oaks, Wax Myrtle	Cfa, Humid subtropical	OM3	C0	0	O horizon	10.6	9.9	0.4	NA	NA	24.4
TXA029B	TXA029	LP_TX_	Texas	TXA	Forest Soil	Whole Community DNA	PCR	Amplicon	pyrotag library	454 GS FLX Titanium	European Nucleotide Archive	https://www.ebi.ac.uk/ena/data/search?query=PRJEB8599	ERS662904	SAMEA3261908	ERX709151	ERR765878	16S rRNA	V1-V3	ACGCGATCGA	AGAGTTTGATCMTGGCTCAG	GWATTACCGCGGCKGCTG	2012-03-12	31.11	-95.15	USA	0.1	88	19.0	253	Aquic Glossudalfs	Loblolly Pine, Beautyberry, Yaupon, Sweetgum, Oaks, Wax Myrtle	Cfa, Humid subtropical	OM3	C0	0	O horizon	26	NA	NA	4.67	NA	NA
TXA030B	TXA030	LP_TX_	Texas	TXA	Forest Soil	Whole Community DNA	PCR	Amplicon	pyrotag library	454 GS FLX Titanium	European Nucleotide Archive	https://www.ebi.ac.uk/ena/data/search?query=PRJEB8599	ERS662905	SAMEA3261909	ERX709152	ERR765879	16S rRNA	V1-V3	ACGCGAGTAT	AGAGTTTGATCMTGGCTCAG	GWATTACCGCGGCKGCTG	2012-03-12	31.11	-95.15	USA	0.1	88	19.0	253	Aquic Glossudalfs	Loblolly Pine, Beautyberry, Yaupon, Sweetgum, Oaks, Wax Myrtle	Cfa, Humid subtropical	OM3	C0	0	O horizon	19.0	13.5	0.5	4.4	NA	29.3
TXA031B	TXA031	LP_TX_	Texas	TXA	Forest Soil	Whole Community DNA	PCR	Amplicon	pyrotag library	454 GS FLX Titanium	European Nucleotide Archive	https://www.ebi.ac.uk/ena/data/search?query=PRJEB8599	ERS662906	SAMEA3261910	ERX709153	ERR765880	16S rRNA	V1-V3	TCGATCACGT	AGAGTTTGATCMTGGCTCAG	GWATTACCGCGGCKGCTG	2012-03-12	31.11	-95.15	USA	0.3	88	19.0	253	Aquic Glossudalfs	Loblolly Pine, Beautyberry, Yaupon, Sweetgum, Oaks, Wax Myrtle	Cfa, Humid subtropical	OM3	C0	1	A horizon	8.6	0.8	0.0	5.3	1.3	17.8
TXA032B	TXA032	LP_TX_	Texas	TXA	Forest Soil	Whole Community DNA	PCR	Amplicon	pyrotag library	454 GS FLX Titanium	European Nucleotide Archive	https://www.ebi.ac.uk/ena/data/search?query=PRJEB8599	ERS662907	SAMEA3261911	ERX709154	ERR765881	16S rRNA	V1-V3	TACACGTGAT	AGAGTTTGATCMTGGCTCAG	GWATTACCGCGGCKGCTG	2012-03-12	31.11	-95.15	USA	0.3	88	19.0	253	Aquic Glossudalfs	Loblolly Pine, Beautyberry, Yaupon, Sweetgum, Oaks, Wax Myrtle	Cfa, Humid subtropical	OM3	C0	1	A horizon	9.9	0.8	0.0	4.6	1.3	17.8
TXA033B	TXA033	LP_TX_	Texas	TXA	Forest Soil	Whole Community DNA	PCR	Amplicon	pyrotag library	454 GS FLX Titanium	European Nucleotide Archive	https://www.ebi.ac.uk/ena/data/search?query=PRJEB8599	ERS662908	SAMEA3261912	ERX709155	ERR765882	16S rRNA	V1-V3	ACGCGAGTAT	AGAGTTTGATCMTGGCTCAG	GWATTACCGCGGCKGCTG	2012-03-12	31.11	-95.15	USA	0.3	88	19.0	253	Aquic Glossudalfs	Loblolly Pine, Beautyberry, Yaupon, Sweetgum, Oaks, Wax Myrtle	Cfa, Humid subtropical	OM3	C0	1	A horizon	11.6	0.8	0.0	4.5	1.3	17.8
TXA034B	TXA034	LP_TX_	Texas	TXA	Forest Soil	Whole Community DNA	PCR	Amplicon	pyrotag library	454 GS FLX Titanium	European Nucleotide Archive	https://www.ebi.ac.uk/ena/data/search?query=PRJEB8599	ERS662909	SAMEA3261913	ERX709156	ERR765883	16S rRNA	V1-V3	AGCGTCGTCT	AGAGTTTGATCMTGGCTCAG	GWATTACCGCGGCKGCTG	2012-03-12	31.11	-95.15	USA	0.1	88	19.0	253	Aquic Glossudalfs	Loblolly Pine, Beautyberry, Yaupon, Sweetgum, Oaks, Wax Myrtle	Cfa, Humid subtropical	OM3	C0	1	O horizon	26.0	NA	NA	4.5	NA	NA
TXA035B	TXA035	LP_TX_	Texas	TXA	Forest Soil	Whole Community DNA	PCR	Amplicon	pyrotag library	454 GS FLX Titanium	European Nucleotide Archive	https://www.ebi.ac.uk/ena/data/search?query=PRJEB8599	ERS662910	SAMEA3261914	ERX709157	ERR765884	16S rRNA	V1-V3	TGACGTATGT	AGAGTTTGATCMTGGCTCAG	GWATTACCGCGGCKGCTG	2012-03-12	31.11	-95.15	USA	0.1	88	19.0	253	Aquic Glossudalfs	Loblolly Pine, Beautyberry, Yaupon, Sweetgum, Oaks, Wax Myrtle	Cfa, Humid subtropical	OM3	C0	1	O horizon	18.2	NA	NA	4.5	NA	NA
TXA036B	TXA036	LP_TX_	Texas	TXA	Forest Soil	Whole Community DNA	PCR	Amplicon	pyrotag library	454 GS FLX Titanium	European Nucleotide Archive	https://www.ebi.ac.uk/ena/data/search?query=PRJEB8599	ERS662911	SAMEA3261915	ERX709158	ERR765885	16S rRNA	V1-V3	ACTACTATGT	AGAGTTTGATCMTGGCTCAG	GWATTACCGCGGCKGCTG	2012-03-12	31.11	-95.15	USA	0.1	88	19.0	253	Aquic Glossudalfs	Loblolly Pine, Beautyberry, Yaupon, Sweetgum, Oaks, Wax Myrtle	Cfa, Humid subtropical	OM3	C0	1	O horizon	16.9	NA	NA	4.5	NA	NA
TXA037B	TXA037	LP_TX_	Texas	TXA	Forest Soil	Whole Community DNA	PCR	Amplicon	pyrotag library	454 GS FLX Titanium	European Nucleotide Archive	https://www.ebi.ac.uk/ena/data/search?query=PRJEB8599	ERS662912	SAMEA3261916	ERX709159	ERR765886	16S rRNA	V1-V3	ACTGTACAGT	AGAGTTTGATCMTGGCTCAG	GWATTACCGCGGCKGCTG	2012-03-12	31.11	-95.15	USA	0.3	88	19.0	253	Aquic Glossudalfs	Loblolly Pine, Beautyberry, Yaupon, Sweetgum, Oaks, Wax Myrtle	Cfa, Humid subtropical	REF	REF	0	A horizon	10.4	NA	NA	NA	NA	NA
TXA038B	TXA038	LP_TX_	Texas	TXA	Forest Soil	Whole Community DNA	PCR	Amplicon	pyrotag library	454 GS FLX Titanium	European Nucleotide Archive	https://www.ebi.ac.uk/ena/data/search?query=PRJEB8599	ERS662913	SAMEA3261917	ERX709160	ERR765887	16S rRNA	V1-V3	TAGTGTAGAT	AGAGTTTGATCMTGGCTCAG	GWATTACCGCGGCKGCTG	2012-03-12	31.11	-95.15	USA	0.3	88	19.0	253	Aquic Glossudalfs	Loblolly Pine, Beautyberry, Yaupon, Sweetgum, Oaks, Wax Myrtle	Cfa, Humid subtropical	REF	REF	0	A horizon	10.8	NA	NA	NA	NA	NA
TXA039B	TXA039	LP_TX_	Texas	TXA	Forest Soil	Whole Community DNA	PCR	Amplicon	pyrotag library	454 GS FLX Titanium	European Nucleotide Archive	https://www.ebi.ac.uk/ena/data/search?query=PRJEB8599	ERS662914	SAMEA3261918	ERX709161	ERR765888	16S rRNA	V1-V3	TCTATACTAT	AGAGTTTGATCMTGGCTCAG	GWATTACCGCGGCKGCTG	2012-03-12	31.11	-95.15	USA	0.3	88	19.0	253	Aquic Glossudalfs	Loblolly Pine, Beautyberry, Yaupon, Sweetgum, Oaks, Wax Myrtle	Cfa, Humid subtropical	REF	REF	0	A horizon	10.9	NA	NA	NA	NA	NA
TXA040B	TXA040	LP_TX_	Texas	TXA	Forest Soil	Whole Community DNA	PCR	Amplicon	pyrotag library	454 GS FLX Titanium	European Nucleotide Archive	https://www.ebi.ac.uk/ena/data/search?query=PRJEB8599	ERS662915	SAMEA3261919	ERX709162	ERR765889	16S rRNA	V1-V3	CGTCTAGTAC	AGAGTTTGATCMTGGCTCAG	GWATTACCGCGGCKGCTG	2012-03-12	31.11	-95.15	USA	0.1	88	19.0	253	Aquic Glossudalfs	Loblolly Pine, Beautyberry, Yaupon, Sweetgum, Oaks, Wax Myrtle	Cfa, Humid subtropical	REF	REF	0	O horizon	18.9	17.6	0.6	4.7	NA	31.0
TXA041B	TXA041	LP_TX_	Texas	TXA	Forest Soil	Whole Community DNA	PCR	Amplicon	pyrotag library	454 GS FLX Titanium	European Nucleotide Archive	https://www.ebi.ac.uk/ena/data/search?query=PRJEB8599	ERS662916	SAMEA3261920	ERX709163	ERR765890	16S rRNA	V1-V3	TACACACACT	AGAGTTTGATCMTGGCTCAG	GWATTACCGCGGCKGCTG	2012-03-12	31.11	-95.15	USA	0.1	88	19.0	253	Aquic Glossudalfs	Loblolly Pine, Beautyberry, Yaupon, Sweetgum, Oaks, Wax Myrtle	Cfa, Humid subtropical	REF	REF	0	O horizon	33.8	21.0	0.7	4.4	NA	28.0
TXA042B	TXA042	LP_TX_	Texas	TXA	Forest Soil	Whole Community DNA	PCR	Amplicon	pyrotag library	454 GS FLX Titanium	European Nucleotide Archive	https://www.ebi.ac.uk/ena/data/search?query=PRJEB8599	ERS662917	SAMEA3261921	ERX709164	ERR765891	16S rRNA	V1-V3	ACTAGCAGTA	AGAGTTTGATCMTGGCTCAG	GWATTACCGCGGCKGCTG	2012-03-12	31.11	-95.15	USA	0.1	88	19.0	253	Aquic Glossudalfs	Loblolly Pine, Beautyberry, Yaupon, Sweetgum, Oaks, Wax Myrtle	Cfa, Humid subtropical	REF	REF	0	O horizon	26.3	16.9	0.6	4.3	NA	27.2
TXB043B	TXB043	LP_TX_	Texas	TXB	Forest Soil	Whole Community DNA	PCR	Amplicon	pyrotag library	454 GS FLX Titanium	European Nucleotide Archive	https://www.ebi.ac.uk/ena/data/search?query=PRJEB8599	ERS662918	SAMEA3261922	ERX709165	ERR765892	16S rRNA	V1-V3	TCTACGTAGC	AGAGTTTGATCMTGGCTCAG	GWATTACCGCGGCKGCTG	2012-03-12	31.11	-95.15	USA	0.3	88	19.0	253	Aquic Glossudalfs	Loblolly Pine, Beautyberry, Yaupon, Sweetgum, Oaks, Wax Myrtle	Cfa, Humid subtropical	OM1	C0	0	A horizon	18.2	0.9	0.1	5.1	1.3	15.5
TXB044B	TXB044	LP_TX_	Texas	TXB	Forest Soil	Whole Community DNA	PCR	Amplicon	pyrotag library	454 GS FLX Titanium	European Nucleotide Archive	https://www.ebi.ac.uk/ena/data/search?query=PRJEB8599	ERS662919	SAMEA3261923	ERX709166	ERR765893	16S rRNA	V1-V3	ACAGTATATA	AGAGTTTGATCMTGGCTCAG	GWATTACCGCGGCKGCTG	2012-03-12	31.11	-95.15	USA	0.3	88	19.0	253	Aquic Glossudalfs	Loblolly Pine, Beautyberry, Yaupon, Sweetgum, Oaks, Wax Myrtle	Cfa, Humid subtropical	OM1	C0	0	A horizon	14.7	0.9	0.1	5.5	1.3	15.5
TXB045B	TXB045	LP_TX_	Texas	TXB	Forest Soil	Whole Community DNA	PCR	Amplicon	pyrotag library	454 GS FLX Titanium	European Nucleotide Archive	https://www.ebi.ac.uk/ena/data/search?query=PRJEB8599	ERS662920	SAMEA3261924	ERX709167	ERR765894	16S rRNA	V1-V3	CGACGTGACT	AGAGTTTGATCMTGGCTCAG	GWATTACCGCGGCKGCTG	2012-03-12	31.11	-95.15	USA	0.3	88	19.0	253	Aquic Glossudalfs	Loblolly Pine, Beautyberry, Yaupon, Sweetgum, Oaks, Wax Myrtle	Cfa, Humid subtropical	OM1	C0	0	A horizon	13.7	0.9	0.1	4.9	1.3	15.5
TXB046B	TXB046	LP_TX_	Texas	TXB	Forest Soil	Whole Community DNA	PCR	Amplicon	pyrotag library	454 GS FLX Titanium	European Nucleotide Archive	https://www.ebi.ac.uk/ena/data/search?query=PRJEB8599	ERS662921	SAMEA3261925	ERX709168	ERR765895	16S rRNA	V1-V3	TCGATCACGT	AGAGTTTGATCMTGGCTCAG	GWATTACCGCGGCKGCTG	2012-03-12	31.11	-95.15	USA	0.1	88	19.0	253	Aquic Glossudalfs	Loblolly Pine, Beautyberry, Yaupon, Sweetgum, Oaks, Wax Myrtle	Cfa, Humid subtropical	OM1	C0	0	O horizon	29.2	12.8	0.5	5.0	NA	28.0
TXB047B	TXB047	LP_TX_	Texas	TXB	Forest Soil	Whole Community DNA	PCR	Amplicon	pyrotag library	454 GS FLX Titanium	European Nucleotide Archive	https://www.ebi.ac.uk/ena/data/search?query=PRJEB8599	ERS662922	SAMEA3261926	ERX709169	ERR765896	16S rRNA	V1-V3	CTCGCGTGTC	AGAGTTTGATCMTGGCTCAG	GWATTACCGCGGCKGCTG	2012-03-12	31.11	-95.15	USA	0.1	88	19.0	253	Aquic Glossudalfs	Loblolly Pine, Beautyberry, Yaupon, Sweetgum, Oaks, Wax Myrtle	Cfa, Humid subtropical	OM1	C0	0	O horizon	32.2	11.3	0.5	4.9	NA	25.0
TXB048B	TXB048	LP_TX_	Texas	TXB	Forest Soil	Whole Community DNA	PCR	Amplicon	pyrotag library	454 GS FLX Titanium	European Nucleotide Archive	https://www.ebi.ac.uk/ena/data/search?query=PRJEB8599	ERS662923	SAMEA3261927	ERX709170	ERR765897	16S rRNA	V1-V3	TGATACGTCT	AGAGTTTGATCMTGGCTCAG	GWATTACCGCGGCKGCTG	2012-03-12	31.11	-95.15	USA	0.1	88	19.0	253	Aquic Glossudalfs	Loblolly Pine, Beautyberry, Yaupon, Sweetgum, Oaks, Wax Myrtle	Cfa, Humid subtropical	OM1	C0	0	O horizon	23.2	9.0	0.4	5.0	NA	25.2
TXB049B	TXB049	LP_TX_	Texas	TXB	Forest Soil	Whole Community DNA	PCR	Amplicon	pyrotag library	454 GS FLX Titanium	European Nucleotide Archive	https://www.ebi.ac.uk/ena/data/search?query=PRJEB8599	ERS662924	SAMEA3261928	ERX709171	ERR765898	16S rRNA	V1-V3	AGCACTGTAG	AGAGTTTGATCMTGGCTCAG	GWATTACCGCGGCKGCTG	2012-03-12	31.11	-95.15	USA	0.3	88	19.0	253	Aquic Glossudalfs	Loblolly Pine, Beautyberry, Yaupon, Sweetgum, Oaks, Wax Myrtle	Cfa, Humid subtropical	OM1	C0	1	A horizon	16.8	0.9	0.1	5.0	1.3	15.5
TXB050B	TXB050	LP_TX_	Texas	TXB	Forest Soil	Whole Community DNA	PCR	Amplicon	pyrotag library	454 GS FLX Titanium	European Nucleotide Archive	https://www.ebi.ac.uk/ena/data/search?query=PRJEB8599	ERS662925	SAMEA3261929	ERX709172	ERR765899	16S rRNA	V1-V3	TCTCTATGCG	AGAGTTTGATCMTGGCTCAG	GWATTACCGCGGCKGCTG	2012-03-12	31.11	-95.15	USA	0.3	88	19.0	253	Aquic Glossudalfs	Loblolly Pine, Beautyberry, Yaupon, Sweetgum, Oaks, Wax Myrtle	Cfa, Humid subtropical	OM1	C0	1	A horizon	12.0	0.9	0.1	4.7	1.3	15.5
TXB051B	TXB051	LP_TX_	Texas	TXB	Forest Soil	Whole Community DNA	PCR	Amplicon	pyrotag library	454 GS FLX Titanium	European Nucleotide Archive	https://www.ebi.ac.uk/ena/data/search?query=PRJEB8599	ERS662926	SAMEA3261930	ERX709173	ERR765900	16S rRNA	V1-V3	TGATACGTCT	AGAGTTTGATCMTGGCTCAG	GWATTACCGCGGCKGCTG	2012-03-12	31.11	-95.15	USA	0.3	88	19.0	253	Aquic Glossudalfs	Loblolly Pine, Beautyberry, Yaupon, Sweetgum, Oaks, Wax Myrtle	Cfa, Humid subtropical	OM1	C0	1	A horizon	12.9	0.9	0.1	4.8	1.3	15.5
TXB052B	TXB052	LP_TX_	Texas	TXB	Forest Soil	Whole Community DNA	PCR	Amplicon	pyrotag library	454 GS FLX Titanium	European Nucleotide Archive	https://www.ebi.ac.uk/ena/data/search?query=PRJEB8599	ERS662927	SAMEA3261931	ERX709174	ERR765901	16S rRNA	V1-V3	TACACACACT	AGAGTTTGATCMTGGCTCAG	GWATTACCGCGGCKGCTG	2012-03-12	31.11	-95.15	USA	0.1	88	19.0	253	Aquic Glossudalfs	Loblolly Pine, Beautyberry, Yaupon, Sweetgum, Oaks, Wax Myrtle	Cfa, Humid subtropical	OM1	C0	1	O horizon	20.0	NA	NA	5.3	NA	NA
TXB053B	TXB053	LP_TX_	Texas	TXB	Forest Soil	Whole Community DNA	PCR	Amplicon	pyrotag library	454 GS FLX Titanium	European Nucleotide Archive	https://www.ebi.ac.uk/ena/data/search?query=PRJEB8599	ERS662928	SAMEA3261932	ERX709175	ERR765902	16S rRNA	V1-V3	TCGCACTAGT	AGAGTTTGATCMTGGCTCAG	GWATTACCGCGGCKGCTG	2012-03-12	31.11	-95.15	USA	0.1	88	19.0	253	Aquic Glossudalfs	Loblolly Pine, Beautyberry, Yaupon, Sweetgum, Oaks, Wax Myrtle	Cfa, Humid subtropical	OM1	C0	1	O horizon	18.8	NA	NA	5.6	NA	NA
TXB054B	TXB054	LP_TX_	Texas	TXB	Forest Soil	Whole Community DNA	PCR	Amplicon	pyrotag library	454 GS FLX Titanium	European Nucleotide Archive	https://www.ebi.ac.uk/ena/data/search?query=PRJEB8599	ERS662929	SAMEA3261933	ERX709176	ERR765903	16S rRNA	V1-V3	AGCGTCGTCT	AGAGTTTGATCMTGGCTCAG	GWATTACCGCGGCKGCTG	2012-03-12	31.11	-95.15	USA	0.1	88	19.0	253	Aquic Glossudalfs	Loblolly Pine, Beautyberry, Yaupon, Sweetgum, Oaks, Wax Myrtle	Cfa, Humid subtropical	OM1	C0	1	O horizon	25.3	NA	NA	4.9	NA	NA
TXB055B	TXB055	LP_TX_	Texas	TXB	Forest Soil	Whole Community DNA	PCR	Amplicon	pyrotag library	454 GS FLX Titanium	European Nucleotide Archive	https://www.ebi.ac.uk/ena/data/search?query=PRJEB8599	ERS662930	SAMEA3261934	ERX709177	ERR765904	16S rRNA	V1-V3	CGTGTCTCTA	AGAGTTTGATCMTGGCTCAG	GWATTACCGCGGCKGCTG	2012-03-12	31.11	-95.15	USA	0.3	88	19.0	253	Aquic Glossudalfs	Loblolly Pine, Beautyberry, Yaupon, Sweetgum, Oaks, Wax Myrtle	Cfa, Humid subtropical	OM2	C0	0	A horizon	20.9	1.0	0.1	5.6	1.2	19.4
TXB056B	TXB056	LP_TX_	Texas	TXB	Forest Soil	Whole Community DNA	PCR	Amplicon	pyrotag library	454 GS FLX Titanium	European Nucleotide Archive	https://www.ebi.ac.uk/ena/data/search?query=PRJEB8599	ERS662931	SAMEA3261935	ERX709178	ERR765905	16S rRNA	V1-V3	TACACGTGAT	AGAGTTTGATCMTGGCTCAG	GWATTACCGCGGCKGCTG	2012-03-12	31.11	-95.15	USA	0.3	88	19.0	253	Aquic Glossudalfs	Loblolly Pine, Beautyberry, Yaupon, Sweetgum, Oaks, Wax Myrtle	Cfa, Humid subtropical	OM2	C0	0	A horizon	31.2	1.0	0.1	5.4	1.2	19.4
TXB057B	TXB057	LP_TX_	Texas	TXB	Forest Soil	Whole Community DNA	PCR	Amplicon	pyrotag library	454 GS FLX Titanium	European Nucleotide Archive	https://www.ebi.ac.uk/ena/data/search?query=PRJEB8599	ERS662932	SAMEA3261936	ERX709179	ERR765906	16S rRNA	V1-V3	TAGTGTAGAT	AGAGTTTGATCMTGGCTCAG	GWATTACCGCGGCKGCTG	2012-03-12	31.11	-95.15	USA	0.3	88	19.0	253	Aquic Glossudalfs	Loblolly Pine, Beautyberry, Yaupon, Sweetgum, Oaks, Wax Myrtle	Cfa, Humid subtropical	OM2	C0	0	A horizon	31.5	1.0	0.1	5.6	1.2	19.4
TXB058B	TXB058	LP_TX_	Texas	TXB	Forest Soil	Whole Community DNA	PCR	Amplicon	pyrotag library	454 GS FLX Titanium	European Nucleotide Archive	https://www.ebi.ac.uk/ena/data/search?query=PRJEB8599	ERS662933	SAMEA3261937	ERX709180	ERR765907	16S rRNA	V1-V3	TCTATACTAT	AGAGTTTGATCMTGGCTCAG	GWATTACCGCGGCKGCTG	2012-03-12	31.11	-95.15	USA	0.1	88	19.0	253	Aquic Glossudalfs	Loblolly Pine, Beautyberry, Yaupon, Sweetgum, Oaks, Wax Myrtle	Cfa, Humid subtropical	OM2	C0	0	O horizon	14.9	9.3	0.4	5.3	NA	23.4
TXB059B	TXB059	LP_TX_	Texas	TXB	Forest Soil	Whole Community DNA	PCR	Amplicon	pyrotag library	454 GS FLX Titanium	European Nucleotide Archive	https://www.ebi.ac.uk/ena/data/search?query=PRJEB8599	ERS662934	SAMEA3261938	ERX709181	ERR765908	16S rRNA	V1-V3	TCGCACTAGT	AGAGTTTGATCMTGGCTCAG	GWATTACCGCGGCKGCTG	2012-03-12	31.11	-95.15	USA	0.1	88	19.0	253	Aquic Glossudalfs	Loblolly Pine, Beautyberry, Yaupon, Sweetgum, Oaks, Wax Myrtle	Cfa, Humid subtropical	OM2	C0	0	O horizon	26.5	12.0	0.4	5.5	NA	27.1
TXB060B	TXB060	LP_TX_	Texas	TXB	Forest Soil	Whole Community DNA	PCR	Amplicon	pyrotag library	454 GS FLX Titanium	European Nucleotide Archive	https://www.ebi.ac.uk/ena/data/search?query=PRJEB8599	ERS662935	SAMEA3261939	ERX709182	ERR765909	16S rRNA	V1-V3	AGCACTGTAG	AGAGTTTGATCMTGGCTCAG	GWATTACCGCGGCKGCTG	2012-03-12	31.11	-95.15	USA	0.1	88	19.0	253	Aquic Glossudalfs	Loblolly Pine, Beautyberry, Yaupon, Sweetgum, Oaks, Wax Myrtle	Cfa, Humid subtropical	OM2	C0	0	O horizon	24.8	8.9	0.3	5.1	NA	26.5
TXB061B	TXB061	LP_TX_	Texas	TXB	Forest Soil	Whole Community DNA	PCR	Amplicon	pyrotag library	454 GS FLX Titanium	European Nucleotide Archive	https://www.ebi.ac.uk/ena/data/search?query=PRJEB8599	ERS662936	SAMEA3261940	ERX709183	ERR765910	16S rRNA	V1-V3	TAGTGTAGAT	AGAGTTTGATCMTGGCTCAG	GWATTACCGCGGCKGCTG	2012-03-12	31.11	-95.15	USA	0.3	88	19.0	253	Aquic Glossudalfs	Loblolly Pine, Beautyberry, Yaupon, Sweetgum, Oaks, Wax Myrtle	Cfa, Humid subtropical	OM2	C0	1	A horizon	15.2	1.0	0.1	5.0	1.2	19.4
TXB062B	TXB062	LP_TX_	Texas	TXB	Forest Soil	Whole Community DNA	PCR	Amplicon	pyrotag library	454 GS FLX Titanium	European Nucleotide Archive	https://www.ebi.ac.uk/ena/data/search?query=PRJEB8599	ERS662937	SAMEA3261941	ERX709184	ERR765911	16S rRNA	V1-V3	TGACGTATGT	AGAGTTTGATCMTGGCTCAG	GWATTACCGCGGCKGCTG	2012-03-12	31.11	-95.15	USA	0.3	88	19.0	253	Aquic Glossudalfs	Loblolly Pine, Beautyberry, Yaupon, Sweetgum, Oaks, Wax Myrtle	Cfa, Humid subtropical	OM2	C0	1	A horizon	22.8	1.0	0.1	4.9	1.2	19.4
TXB063B	TXB063	LP_TX_	Texas	TXB	Forest Soil	Whole Community DNA	PCR	Amplicon	pyrotag library	454 GS FLX Titanium	European Nucleotide Archive	https://www.ebi.ac.uk/ena/data/search?query=PRJEB8599	ERS662938	SAMEA3261942	ERX709185	ERR765912	16S rRNA	V1-V3	TCTACGTAGC	AGAGTTTGATCMTGGCTCAG	GWATTACCGCGGCKGCTG	2012-03-12	31.11	-95.15	USA	0.3	88	19.0	253	Aquic Glossudalfs	Loblolly Pine, Beautyberry, Yaupon, Sweetgum, Oaks, Wax Myrtle	Cfa, Humid subtropical	OM2	C0	1	A horizon	10.2	1.0	0.1	4.8	1.2	19.4
TXB064B	TXB064	LP_TX_	Texas	TXB	Forest Soil	Whole Community DNA	PCR	Amplicon	pyrotag library	454 GS FLX Titanium	European Nucleotide Archive	https://www.ebi.ac.uk/ena/data/search?query=PRJEB8599	ERS662939	SAMEA3261943	ERX709186	ERR765913	16S rRNA	V1-V3	AGACTATACT	AGAGTTTGATCMTGGCTCAG	GWATTACCGCGGCKGCTG	2012-03-12	31.11	-95.15	USA	0.1	88	19.0	253	Aquic Glossudalfs	Loblolly Pine, Beautyberry, Yaupon, Sweetgum, Oaks, Wax Myrtle	Cfa, Humid subtropical	OM2	C0	1	O horizon	21.5	NA	NA	4.4	NA	NA
TXB065B	TXB065	LP_TX_	Texas	TXB	Forest Soil	Whole Community DNA	PCR	Amplicon	pyrotag library	454 GS FLX Titanium	European Nucleotide Archive	https://www.ebi.ac.uk/ena/data/search?query=PRJEB8599	ERS662940	SAMEA3261944	ERX709187	ERR765914	16S rRNA	V1-V3	TAGTGTAGAT	AGAGTTTGATCMTGGCTCAG	GWATTACCGCGGCKGCTG	2012-03-12	31.11	-95.15	USA	0.1	88	19.0	253	Aquic Glossudalfs	Loblolly Pine, Beautyberry, Yaupon, Sweetgum, Oaks, Wax Myrtle	Cfa, Humid subtropical	OM2	C0	1	O horizon	22.8	NA	NA	5.0	NA	NA
TXB066B	TXB066	LP_TX_	Texas	TXB	Forest Soil	Whole Community DNA	PCR	Amplicon	pyrotag library	454 GS FLX Titanium	European Nucleotide Archive	https://www.ebi.ac.uk/ena/data/search?query=PRJEB8599	ERS662941	SAMEA3261945	ERX709188	ERR765915	16S rRNA	V1-V3	ATACGACGTA	AGAGTTTGATCMTGGCTCAG	GWATTACCGCGGCKGCTG	2012-03-12	31.11	-95.15	USA	0.1	88	19.0	253	Aquic Glossudalfs	Loblolly Pine, Beautyberry, Yaupon, Sweetgum, Oaks, Wax Myrtle	Cfa, Humid subtropical	OM2	C0	1	O horizon	22.0	NA	NA	4.7	NA	NA
TXB067B	TXB067	LP_TX_	Texas	TXB	Forest Soil	Whole Community DNA	PCR	Amplicon	pyrotag library	454 GS FLX Titanium	European Nucleotide Archive	https://www.ebi.ac.uk/ena/data/search?query=PRJEB8599	ERS662942	SAMEA3261946	ERX709189	ERR765916	16S rRNA	V1-V3	ACGAGTGCGT	AGAGTTTGATCMTGGCTCAG	GWATTACCGCGGCKGCTG	2012-03-12	31.11	-95.15	USA	0.3	88	19.0	253	Aquic Glossudalfs	Loblolly Pine, Beautyberry, Yaupon, Sweetgum, Oaks, Wax Myrtle	Cfa, Humid subtropical	OM3	C0	0	A horizon	18.3	1.0	0.1	4.5	1.3	20.1
TXB068B	TXB068	LP_TX_	Texas	TXB	Forest Soil	Whole Community DNA	PCR	Amplicon	pyrotag library	454 GS FLX Titanium	European Nucleotide Archive	https://www.ebi.ac.uk/ena/data/search?query=PRJEB8599	ERS662943	SAMEA3261947	ERX709190	ERR765917	16S rRNA	V1-V3	ACGCGATCGA	AGAGTTTGATCMTGGCTCAG	GWATTACCGCGGCKGCTG	2012-03-12	31.11	-95.15	USA	0.3	88	19.0	253	Aquic Glossudalfs	Loblolly Pine, Beautyberry, Yaupon, Sweetgum, Oaks, Wax Myrtle	Cfa, Humid subtropical	OM3	C0	0	A horizon	19.8	1.0	0.1	4.4	1.3	20.1
TXB069B	TXB069	LP_TX_	Texas	TXB	Forest Soil	Whole Community DNA	PCR	Amplicon	pyrotag library	454 GS FLX Titanium	European Nucleotide Archive	https://www.ebi.ac.uk/ena/data/search?query=PRJEB8599	ERS662944	SAMEA3261948	ERX709191	ERR765918	16S rRNA	V1-V3	TGTGAGTAGT	AGAGTTTGATCMTGGCTCAG	GWATTACCGCGGCKGCTG	2012-03-12	31.11	-95.15	USA	0.3	88	19.0	253	Aquic Glossudalfs	Loblolly Pine, Beautyberry, Yaupon, Sweetgum, Oaks, Wax Myrtle	Cfa, Humid subtropical	OM3	C0	0	A horizon	12	0.98	0.05	4.53	1.3	20.07
TXB070B	TXB070	LP_TX_	Texas	TXB	Forest Soil	Whole Community DNA	PCR	Amplicon	pyrotag library	454 GS FLX Titanium	European Nucleotide Archive	https://www.ebi.ac.uk/ena/data/search?query=PRJEB8599	ERS662945	SAMEA3261949	ERX709192	ERR765919	16S rRNA	V1-V3	TACACACACT	AGAGTTTGATCMTGGCTCAG	GWATTACCGCGGCKGCTG	2012-03-12	31.11	-95.15	USA	0.1	88	19.0	253	Aquic Glossudalfs	Loblolly Pine, Beautyberry, Yaupon, Sweetgum, Oaks, Wax Myrtle	Cfa, Humid subtropical	OM3	C0	0	O horizon	29.1	10.1	0.4	4.3	NA	28.1
TXB071B	TXB071	LP_TX_	Texas	TXB	Forest Soil	Whole Community DNA	PCR	Amplicon	pyrotag library	454 GS FLX Titanium	European Nucleotide Archive	https://www.ebi.ac.uk/ena/data/search?query=PRJEB8599	ERS662946	SAMEA3261950	ERX709193	ERR765920	16S rRNA	V1-V3	TCGATCACGT	AGAGTTTGATCMTGGCTCAG	GWATTACCGCGGCKGCTG	2012-03-12	31.11	-95.15	USA	0.1	88	19.0	253	Aquic Glossudalfs	Loblolly Pine, Beautyberry, Yaupon, Sweetgum, Oaks, Wax Myrtle	Cfa, Humid subtropical	OM3	C0	0	O horizon	32.9	15.6	0.5	4.5	NA	31.8
TXB072B	TXB072	LP_TX_	Texas	TXB	Forest Soil	Whole Community DNA	PCR	Amplicon	pyrotag library	454 GS FLX Titanium	European Nucleotide Archive	https://www.ebi.ac.uk/ena/data/search?query=PRJEB8599	ERS662947	SAMEA3261951	ERX709194	ERR765921	16S rRNA	V1-V3	ACTAGCAGTA	AGAGTTTGATCMTGGCTCAG	GWATTACCGCGGCKGCTG	2012-03-12	31.11	-95.15	USA	0.1	88	19.0	253	Aquic Glossudalfs	Loblolly Pine, Beautyberry, Yaupon, Sweetgum, Oaks, Wax Myrtle	Cfa, Humid subtropical	OM3	C0	0	O horizon	33.6	14.9	0.5	4.6	NA	28.9
TXB073B	TXB073	LP_TX_	Texas	TXB	Forest Soil	Whole Community DNA	PCR	Amplicon	pyrotag library	454 GS FLX Titanium	European Nucleotide Archive	https://www.ebi.ac.uk/ena/data/search?query=PRJEB8599	ERS662948	SAMEA3261952	ERX709195	ERR765922	16S rRNA	V1-V3	ATATCGCGAG	AGAGTTTGATCMTGGCTCAG	GWATTACCGCGGCKGCTG	2012-03-12	31.11	-95.15	USA	0.3	88	19.0	253	Aquic Glossudalfs	Loblolly Pine, Beautyberry, Yaupon, Sweetgum, Oaks, Wax Myrtle	Cfa, Humid subtropical	OM3	C0	1	A horizon	17.8	1.0	0.1	5.0	1.3	20.1
TXB074B	TXB074	LP_TX_	Texas	TXB	Forest Soil	Whole Community DNA	PCR	Amplicon	pyrotag library	454 GS FLX Titanium	European Nucleotide Archive	https://www.ebi.ac.uk/ena/data/search?query=PRJEB8599	ERS662949	SAMEA3261953	ERX709196	ERR765923	16S rRNA	V1-V3	ACTAGCAGTA	AGAGTTTGATCMTGGCTCAG	GWATTACCGCGGCKGCTG	2012-03-12	31.11	-95.15	USA	0.3	88	19.0	253	Aquic Glossudalfs	Loblolly Pine, Beautyberry, Yaupon, Sweetgum, Oaks, Wax Myrtle	Cfa, Humid subtropical	OM3	C0	1	A horizon	6	0.98	0.05	4.81	1.3	20.07
TXB075B	TXB075	LP_TX_	Texas	TXB	Forest Soil	Whole Community DNA	PCR	Amplicon	pyrotag library	454 GS FLX Titanium	European Nucleotide Archive	https://www.ebi.ac.uk/ena/data/search?query=PRJEB8599	ERS662950	SAMEA3261954	ERX709197	ERR765924	16S rRNA	V1-V3	ACTAGCAGTA	AGAGTTTGATCMTGGCTCAG	GWATTACCGCGGCKGCTG	2012-03-12	31.11	-95.15	USA	0.3	88	19.0	253	Aquic Glossudalfs	Loblolly Pine, Beautyberry, Yaupon, Sweetgum, Oaks, Wax Myrtle	Cfa, Humid subtropical	OM3	C0	1	A horizon	19.2	1.0	0.1	4.8	1.3	20.1
TXB076B	TXB076	LP_TX_	Texas	TXB	Forest Soil	Whole Community DNA	PCR	Amplicon	pyrotag library	454 GS FLX Titanium	European Nucleotide Archive	https://www.ebi.ac.uk/ena/data/search?query=PRJEB8599	ERS662951	SAMEA3261955	ERX709198	ERR765925	16S rRNA	V1-V3	TCTCTATGCG	AGAGTTTGATCMTGGCTCAG	GWATTACCGCGGCKGCTG	2012-03-12	31.11	-95.15	USA	0.1	88	19.0	253	Aquic Glossudalfs	Loblolly Pine, Beautyberry, Yaupon, Sweetgum, Oaks, Wax Myrtle	Cfa, Humid subtropical	OM3	C0	1	O horizon	25.2	NA	NA	4.3	NA	NA
TXB077B	TXB077	LP_TX_	Texas	TXB	Forest Soil	Whole Community DNA	PCR	Amplicon	pyrotag library	454 GS FLX Titanium	European Nucleotide Archive	https://www.ebi.ac.uk/ena/data/search?query=PRJEB8599	ERS662952	SAMEA3261956	ERX709199	ERR765926	16S rRNA	V1-V3	CGTGTCTCTA	AGAGTTTGATCMTGGCTCAG	GWATTACCGCGGCKGCTG	2012-03-12	31.11	-95.15	USA	0.1	88	19.0	253	Aquic Glossudalfs	Loblolly Pine, Beautyberry, Yaupon, Sweetgum, Oaks, Wax Myrtle	Cfa, Humid subtropical	OM3	C0	1	O horizon	30.2	NA	NA	4.4	NA	NA
TXB078B	TXB078	LP_TX_	Texas	TXB	Forest Soil	Whole Community DNA	PCR	Amplicon	pyrotag library	454 GS FLX Titanium	European Nucleotide Archive	https://www.ebi.ac.uk/ena/data/search?query=PRJEB8599	ERS662953	SAMEA3261957	ERX709200	ERR765927	16S rRNA	V1-V3	ACGAGTGCGT	AGAGTTTGATCMTGGCTCAG	GWATTACCGCGGCKGCTG	2012-03-12	31.11	-95.15	USA	0.1	88	19.0	253	Aquic Glossudalfs	Loblolly Pine, Beautyberry, Yaupon, Sweetgum, Oaks, Wax Myrtle	Cfa, Humid subtropical	OM3	C0	1	O horizon	23.8	NA	NA	4.3	NA	NA
TXB079B	TXB079	LP_TX_	Texas	TXB	Forest Soil	Whole Community DNA	PCR	Amplicon	pyrotag library	454 GS FLX Titanium	European Nucleotide Archive	https://www.ebi.ac.uk/ena/data/search?query=PRJEB8599	ERS662954	SAMEA3261958	ERX709201	ERR765928	16S rRNA	V1-V3	TCGATCACGT	AGAGTTTGATCMTGGCTCAG	GWATTACCGCGGCKGCTG	2012-03-12	31.11	-95.15	USA	0.3	88	19.0	253	Aquic Glossudalfs	Loblolly Pine, Beautyberry, Yaupon, Sweetgum, Oaks, Wax Myrtle	Cfa, Humid subtropical	REF	REF	0	A horizon	16.0	NA	NA	NA	NA	NA
TXB080B	TXB080	LP_TX_	Texas	TXB	Forest Soil	Whole Community DNA	PCR	Amplicon	pyrotag library	454 GS FLX Titanium	European Nucleotide Archive	https://www.ebi.ac.uk/ena/data/search?query=PRJEB8599	ERS662955	SAMEA3261959	ERX709202	ERR765929	16S rRNA	V1-V3	TACACGTGAT	AGAGTTTGATCMTGGCTCAG	GWATTACCGCGGCKGCTG	2012-03-12	31.11	-95.15	USA	0.3	88	19.0	253	Aquic Glossudalfs	Loblolly Pine, Beautyberry, Yaupon, Sweetgum, Oaks, Wax Myrtle	Cfa, Humid subtropical	REF	REF	0	A horizon	17.2	NA	NA	NA	NA	NA
TXB081B	TXB081	LP_TX_	Texas	TXB	Forest Soil	Whole Community DNA	PCR	Amplicon	pyrotag library	454 GS FLX Titanium	European Nucleotide Archive	https://www.ebi.ac.uk/ena/data/search?query=PRJEB8599	ERS662956	SAMEA3261960	ERX709203	ERR765930	16S rRNA	V1-V3	ACGCTCGACA	AGAGTTTGATCMTGGCTCAG	GWATTACCGCGGCKGCTG	2012-03-12	31.11	-95.15	USA	0.3	88	19.0	253	Aquic Glossudalfs	Loblolly Pine, Beautyberry, Yaupon, Sweetgum, Oaks, Wax Myrtle	Cfa, Humid subtropical	REF	REF	0	A horizon	18.2	NA	NA	NA	NA	NA
TXB082B	TXB082	LP_TX_	Texas	TXB	Forest Soil	Whole Community DNA	PCR	Amplicon	pyrotag library	454 GS FLX Titanium	European Nucleotide Archive	https://www.ebi.ac.uk/ena/data/search?query=PRJEB8599	ERS662957	SAMEA3261961	ERX709204	ERR765931	16S rRNA	V1-V3	CACGCTACGT	AGAGTTTGATCMTGGCTCAG	GWATTACCGCGGCKGCTG	2012-03-12	31.11	-95.15	USA	0.1	88	19.0	253	Aquic Glossudalfs	Loblolly Pine, Beautyberry, Yaupon, Sweetgum, Oaks, Wax Myrtle	Cfa, Humid subtropical	REF	REF	0	O horizon	34.6	24.2	0.9	4.5	NA	27.5
TXB083B	TXB083	LP_TX_	Texas	TXB	Forest Soil	Whole Community DNA	PCR	Amplicon	pyrotag library	454 GS FLX Titanium	European Nucleotide Archive	https://www.ebi.ac.uk/ena/data/search?query=PRJEB8599	ERS662958	SAMEA3261962	ERX709205	ERR765932	16S rRNA	V1-V3	AGACTATACT	AGAGTTTGATCMTGGCTCAG	GWATTACCGCGGCKGCTG	2012-03-12	31.11	-95.15	USA	0.1	88	19.0	253	Aquic Glossudalfs	Loblolly Pine, Beautyberry, Yaupon, Sweetgum, Oaks, Wax Myrtle	Cfa, Humid subtropical	REF	REF	0	O horizon	23.2	14.5	0.5	4.4	NA	29.7
TXB084B	TXB084	LP_TX_	Texas	TXB	Forest Soil	Whole Community DNA	PCR	Amplicon	pyrotag library	454 GS FLX Titanium	European Nucleotide Archive	https://www.ebi.ac.uk/ena/data/search?query=PRJEB8599	ERS662959	SAMEA3261963	ERX709206	ERR765933	16S rRNA	V1-V3	TACGCTGTCT	AGAGTTTGATCMTGGCTCAG	GWATTACCGCGGCKGCTG	2012-03-12	31.11	-95.15	USA	0.1	88	19.0	253	Aquic Glossudalfs	Loblolly Pine, Beautyberry, Yaupon, Sweetgum, Oaks, Wax Myrtle	Cfa, Humid subtropical	REF	REF	0	O horizon	22.1	10.4	0.4	4.9	NA	27.7
TXC108B	TXC108	LP_TX_	Texas	TXC	Forest Soil	Whole Community DNA	PCR	Amplicon	pyrotag library	454 GS FLX Titanium	European Nucleotide Archive	https://www.ebi.ac.uk/ena/data/search?query=PRJEB8599	ERS662960	SAMEA3261964	ERX709207	ERR765934	16S rRNA	V1-V3	CGTCTAGTAC	AGAGTTTGATCMTGGCTCAG	GWATTACCGCGGCKGCTG	2012-03-12	31.11	-95.15	USA	0.1	88	19.0	253	Aquic Glossudalfs	Loblolly Pine, Beautyberry, Yaupon, Sweetgum, Oaks, Wax Myrtle	Cfa, Humid subtropical	OM2	C0	1	O horizon	23	NA	NA	4.79	NA	NA
TXC085B	TXC085	LP_TX_	Texas	TXC	Forest Soil	Whole Community DNA	PCR	Amplicon	pyrotag library	454 GS FLX Titanium	European Nucleotide Archive	https://www.ebi.ac.uk/ena/data/search?query=PRJEB8599	ERS662961	SAMEA3261965	ERX709208	ERR765935	16S rRNA	V1-V3	CGACGTGACT	AGAGTTTGATCMTGGCTCAG	GWATTACCGCGGCKGCTG	2012-03-12	31.11	-95.15	USA	0.3	88	19.0	253	Aquic Glossudalfs	Loblolly Pine, Beautyberry, Yaupon, Sweetgum, Oaks, Wax Myrtle	Cfa, Humid subtropical	OM1	C0	0	A horizon	16	0.88	0.05	4.95	1.29	17.41
TXC086B	TXC086	LP_TX_	Texas	TXC	Forest Soil	Whole Community DNA	PCR	Amplicon	pyrotag library	454 GS FLX Titanium	European Nucleotide Archive	https://www.ebi.ac.uk/ena/data/search?query=PRJEB8599	ERS662962	SAMEA3261966	ERX709209	ERR765936	16S rRNA	V1-V3	ACTACTATGT	AGAGTTTGATCMTGGCTCAG	GWATTACCGCGGCKGCTG	2012-03-12	31.11	-95.15	USA	0.3	88	19.0	253	Aquic Glossudalfs	Loblolly Pine, Beautyberry, Yaupon, Sweetgum, Oaks, Wax Myrtle	Cfa, Humid subtropical	OM1	C0	0	A horizon	7	0.88	0.05	4.65	1.29	17.41
TXC087B	TXC087	LP_TX_	Texas	TXC	Forest Soil	Whole Community DNA	PCR	Amplicon	pyrotag library	454 GS FLX Titanium	European Nucleotide Archive	https://www.ebi.ac.uk/ena/data/search?query=PRJEB8599	ERS662963	SAMEA3261967	ERX709210	ERR765937	16S rRNA	V1-V3	CGAGAGATAC	AGAGTTTGATCMTGGCTCAG	GWATTACCGCGGCKGCTG	2012-03-12	31.11	-95.15	USA	0.3	88	19.0	253	Aquic Glossudalfs	Loblolly Pine, Beautyberry, Yaupon, Sweetgum, Oaks, Wax Myrtle	Cfa, Humid subtropical	OM1	C0	0	A horizon	5	0.88	0.05	4.96	1.29	17.41
TXC088B	TXC088	LP_TX_	Texas	TXC	Forest Soil	Whole Community DNA	PCR	Amplicon	pyrotag library	454 GS FLX Titanium	European Nucleotide Archive	https://www.ebi.ac.uk/ena/data/search?query=PRJEB8599	ERS662964	SAMEA3261968	ERX709211	ERR765938	16S rRNA	V1-V3	TCTAGCGACT	AGAGTTTGATCMTGGCTCAG	GWATTACCGCGGCKGCTG	2012-03-12	31.11	-95.15	USA	0.1	88	19.0	253	Aquic Glossudalfs	Loblolly Pine, Beautyberry, Yaupon, Sweetgum, Oaks, Wax Myrtle	Cfa, Humid subtropical	OM1	C0	0	O horizon	9	NA	NA	5.01	NA	NA
TXC089B	TXC089	LP_TX_	Texas	TXC	Forest Soil	Whole Community DNA	PCR	Amplicon	pyrotag library	454 GS FLX Titanium	European Nucleotide Archive	https://www.ebi.ac.uk/ena/data/search?query=PRJEB8599	ERS662965	SAMEA3261969	ERX709212	ERR765939	16S rRNA	V1-V3	TCTAGCGACT	AGAGTTTGATCMTGGCTCAG	GWATTACCGCGGCKGCTG	2012-03-12	31.11	-95.15	USA	0.1	88	19.0	253	Aquic Glossudalfs	Loblolly Pine, Beautyberry, Yaupon, Sweetgum, Oaks, Wax Myrtle	Cfa, Humid subtropical	OM1	C0	0	O horizon	28	NA	NA	5.27	NA	NA
TXC090B	TXC090	LP_TX_	Texas	TXC	Forest Soil	Whole Community DNA	PCR	Amplicon	pyrotag library	454 GS FLX Titanium	European Nucleotide Archive	https://www.ebi.ac.uk/ena/data/search?query=PRJEB8599	ERS662966	SAMEA3261970	ERX709213	ERR765940	16S rRNA	V1-V3	AGCGTCGTCT	AGAGTTTGATCMTGGCTCAG	GWATTACCGCGGCKGCTG	2012-03-12	31.11	-95.15	USA	0.1	88	19.0	253	Aquic Glossudalfs	Loblolly Pine, Beautyberry, Yaupon, Sweetgum, Oaks, Wax Myrtle	Cfa, Humid subtropical	OM1	C0	0	O horizon	26	NA	NA	5.26	NA	NA
TXC091B	TXC091	LP_TX_	Texas	TXC	Forest Soil	Whole Community DNA	PCR	Amplicon	pyrotag library	454 GS FLX Titanium	European Nucleotide Archive	https://www.ebi.ac.uk/ena/data/search?query=PRJEB8599	ERS662967	SAMEA3261971	ERX709214	ERR765941	16S rRNA	V1-V3	CAGTAGACGT	AGAGTTTGATCMTGGCTCAG	GWATTACCGCGGCKGCTG	2012-03-12	31.11	-95.15	USA	0.3	88	19.0	253	Aquic Glossudalfs	Loblolly Pine, Beautyberry, Yaupon, Sweetgum, Oaks, Wax Myrtle	Cfa, Humid subtropical	OM1	C0	1	A horizon	20	0.88	0.05	5.24	1.29	17.41
TXC092B	TXC092	LP_TX_	Texas	TXC	Forest Soil	Whole Community DNA	PCR	Amplicon	pyrotag library	454 GS FLX Titanium	European Nucleotide Archive	https://www.ebi.ac.uk/ena/data/search?query=PRJEB8599	ERS662968	SAMEA3261972	ERX709215	ERR765942	16S rRNA	V1-V3	ACGCGATCGA	AGAGTTTGATCMTGGCTCAG	GWATTACCGCGGCKGCTG	2012-03-12	31.11	-95.15	USA	0.3	88	19.0	253	Aquic Glossudalfs	Loblolly Pine, Beautyberry, Yaupon, Sweetgum, Oaks, Wax Myrtle	Cfa, Humid subtropical	OM1	C0	1	A horizon	2	0.88	0.05	4.93	1.29	17.41
TXC093B	TXC093	LP_TX_	Texas	TXC	Forest Soil	Whole Community DNA	PCR	Amplicon	pyrotag library	454 GS FLX Titanium	European Nucleotide Archive	https://www.ebi.ac.uk/ena/data/search?query=PRJEB8599	ERS662969	SAMEA3261973	ERX709216	ERR765943	16S rRNA	V1-V3	TCACGTACTA	AGAGTTTGATCMTGGCTCAG	GWATTACCGCGGCKGCTG	2012-03-12	31.11	-95.15	USA	0.3	88	19.0	253	Aquic Glossudalfs	Loblolly Pine, Beautyberry, Yaupon, Sweetgum, Oaks, Wax Myrtle	Cfa, Humid subtropical	OM1	C0	1	A horizon	7	0.88	0.05	5.35	1.29	17.41
TXC094B	TXC094	LP_TX_	Texas	TXC	Forest Soil	Whole Community DNA	PCR	Amplicon	pyrotag library	454 GS FLX Titanium	European Nucleotide Archive	https://www.ebi.ac.uk/ena/data/search?query=PRJEB8599	ERS662970	SAMEA3261974	ERX709217	ERR765944	16S rRNA	V1-V3	ACAGTATATA	AGAGTTTGATCMTGGCTCAG	GWATTACCGCGGCKGCTG	2012-03-12	31.11	-95.15	USA	0.1	88	19.0	253	Aquic Glossudalfs	Loblolly Pine, Beautyberry, Yaupon, Sweetgum, Oaks, Wax Myrtle	Cfa, Humid subtropical	OM1	C0	1	O horizon	3	NA	NA	5.04	NA	NA
TXC095B	TXC095	LP_TX_	Texas	TXC	Forest Soil	Whole Community DNA	PCR	Amplicon	pyrotag library	454 GS FLX Titanium	European Nucleotide Archive	https://www.ebi.ac.uk/ena/data/search?query=PRJEB8599	ERS662971	SAMEA3261975	ERX709218	ERR765945	16S rRNA	V1-V3	CAGTAGACGT	AGAGTTTGATCMTGGCTCAG	GWATTACCGCGGCKGCTG	2012-03-12	31.11	-95.15	USA	0.1	88	19.0	253	Aquic Glossudalfs	Loblolly Pine, Beautyberry, Yaupon, Sweetgum, Oaks, Wax Myrtle	Cfa, Humid subtropical	OM1	C0	1	O horizon	18	NA	NA	4.49	NA	NA
TXC096B	TXC096	LP_TX_	Texas	TXC	Forest Soil	Whole Community DNA	PCR	Amplicon	pyrotag library	454 GS FLX Titanium	European Nucleotide Archive	https://www.ebi.ac.uk/ena/data/search?query=PRJEB8599	ERS662972	SAMEA3261976	ERX709219	ERR765946	16S rRNA	V1-V3	TCTACGTAGC	AGAGTTTGATCMTGGCTCAG	GWATTACCGCGGCKGCTG	2012-03-12	31.11	-95.15	USA	0.1	88	19.0	253	Aquic Glossudalfs	Loblolly Pine, Beautyberry, Yaupon, Sweetgum, Oaks, Wax Myrtle	Cfa, Humid subtropical	OM1	C0	1	O horizon	17	NA	NA	5.03	NA	NA
TXC097B	TXC097	LP_TX_	Texas	TXC	Forest Soil	Whole Community DNA	PCR	Amplicon	pyrotag library	454 GS FLX Titanium	European Nucleotide Archive	https://www.ebi.ac.uk/ena/data/search?query=PRJEB8599	ERS662973	SAMEA3261977	ERX709220	ERR765947	16S rRNA	V1-V3	TGTGAGTAGT	AGAGTTTGATCMTGGCTCAG	GWATTACCGCGGCKGCTG	2012-03-12	31.11	-95.15	USA	0.3	88	19.0	253	Aquic Glossudalfs	Loblolly Pine, Beautyberry, Yaupon, Sweetgum, Oaks, Wax Myrtle	Cfa, Humid subtropical	OM2	C0	0	A horizon	16.9	0.9	0.1	5.2	1.3	17.4
TXC098B	TXC098	LP_TX_	Texas	TXC	Forest Soil	Whole Community DNA	PCR	Amplicon	pyrotag library	454 GS FLX Titanium	European Nucleotide Archive	https://www.ebi.ac.uk/ena/data/search?query=PRJEB8599	ERS662974	SAMEA3261978	ERX709221	ERR765948	16S rRNA	V1-V3	TCTAGCGACT	AGAGTTTGATCMTGGCTCAG	GWATTACCGCGGCKGCTG	2012-03-12	31.11	-95.15	USA	0.3	88	19.0	253	Aquic Glossudalfs	Loblolly Pine, Beautyberry, Yaupon, Sweetgum, Oaks, Wax Myrtle	Cfa, Humid subtropical	OM2	C0	0	A horizon	22.4	0.9	0.1	5.4	1.3	17.4
TXC099B	TXC099	LP_TX_	Texas	TXC	Forest Soil	Whole Community DNA	PCR	Amplicon	pyrotag library	454 GS FLX Titanium	European Nucleotide Archive	https://www.ebi.ac.uk/ena/data/search?query=PRJEB8599	ERS662975	SAMEA3261979	ERX709222	ERR765949	16S rRNA	V1-V3	ATCAGACACG	AGAGTTTGATCMTGGCTCAG	GWATTACCGCGGCKGCTG	2012-03-12	31.11	-95.15	USA	0.3	88	19.0	253	Aquic Glossudalfs	Loblolly Pine, Beautyberry, Yaupon, Sweetgum, Oaks, Wax Myrtle	Cfa, Humid subtropical	OM2	C0	0	A horizon	21.4	0.9	0.1	5.9	1.3	17.4
TXC100B	TXC100	LP_TX_	Texas	TXC	Forest Soil	Whole Community DNA	PCR	Amplicon	pyrotag library	454 GS FLX Titanium	European Nucleotide Archive	https://www.ebi.ac.uk/ena/data/search?query=PRJEB8599	ERS662976	SAMEA3261980	ERX709223	ERR765950	16S rRNA	V1-V3	AGCACTGTAG	AGAGTTTGATCMTGGCTCAG	GWATTACCGCGGCKGCTG	2012-03-12	31.11	-95.15	USA	0.1	88	19.0	253	Aquic Glossudalfs	Loblolly Pine, Beautyberry, Yaupon, Sweetgum, Oaks, Wax Myrtle	Cfa, Humid subtropical	OM2	C0	0	O horizon	12.1	6.5	0.3	4.3	NA	24.4
TXC101B	TXC101	LP_TX_	Texas	TXC	Forest Soil	Whole Community DNA	PCR	Amplicon	pyrotag library	454 GS FLX Titanium	European Nucleotide Archive	https://www.ebi.ac.uk/ena/data/search?query=PRJEB8599	ERS662977	SAMEA3261981	ERX709224	ERR765951	16S rRNA	V1-V3	CGACGTGACT	AGAGTTTGATCMTGGCTCAG	GWATTACCGCGGCKGCTG	2012-03-12	31.11	-95.15	USA	0.1	88	19.0	253	Aquic Glossudalfs	Loblolly Pine, Beautyberry, Yaupon, Sweetgum, Oaks, Wax Myrtle	Cfa, Humid subtropical	OM2	C0	0	O horizon	10.6	4.7	0.2	4.8	NA	23.7
TXC102B	TXC102	LP_TX_	Texas	TXC	Forest Soil	Whole Community DNA	PCR	Amplicon	pyrotag library	454 GS FLX Titanium	European Nucleotide Archive	https://www.ebi.ac.uk/ena/data/search?query=PRJEB8599	ERS662978	SAMEA3261982	ERX709225	ERR765952	16S rRNA	V1-V3	AGTACGCTAT	AGAGTTTGATCMTGGCTCAG	GWATTACCGCGGCKGCTG	2012-03-12	31.11	-95.15	USA	0.1	88	19.0	253	Aquic Glossudalfs	Loblolly Pine, Beautyberry, Yaupon, Sweetgum, Oaks, Wax Myrtle	Cfa, Humid subtropical	OM2	C0	0	O horizon	19.2	10.8	0.4	4.6	NA	27.2
TXC103B	TXC103	LP_TX_	Texas	TXC	Forest Soil	Whole Community DNA	PCR	Amplicon	pyrotag library	454 GS FLX Titanium	European Nucleotide Archive	https://www.ebi.ac.uk/ena/data/search?query=PRJEB8599	ERS662979	SAMEA3261983	ERX709226	ERR765953	16S rRNA	V1-V3	CAGTAGACGT	AGAGTTTGATCMTGGCTCAG	GWATTACCGCGGCKGCTG	2012-03-12	31.11	-95.15	USA	0.3	88	19.0	253	Aquic Glossudalfs	Loblolly Pine, Beautyberry, Yaupon, Sweetgum, Oaks, Wax Myrtle	Cfa, Humid subtropical	OM2	C0	1	A horizon	18.1	0.8	0.0	4.4	1.3	15.7
TXC104B	TXC104	LP_TX_	Texas	TXC	Forest Soil	Whole Community DNA	PCR	Amplicon	pyrotag library	454 GS FLX Titanium	European Nucleotide Archive	https://www.ebi.ac.uk/ena/data/search?query=PRJEB8599	ERS662980	SAMEA3261984	ERX709227	ERR765954	16S rRNA	V1-V3	ATACGACGTA	AGAGTTTGATCMTGGCTCAG	GWATTACCGCGGCKGCTG	2012-03-12	31.11	-95.15	USA	0.3	88	19.0	253	Aquic Glossudalfs	Loblolly Pine, Beautyberry, Yaupon, Sweetgum, Oaks, Wax Myrtle	Cfa, Humid subtropical	OM2	C0	1	A horizon	10.9	0.8	0.0	4.5	1.3	15.7
TXC105B	TXC105	LP_TX_	Texas	TXC	Forest Soil	Whole Community DNA	PCR	Amplicon	pyrotag library	454 GS FLX Titanium	European Nucleotide Archive	https://www.ebi.ac.uk/ena/data/search?query=PRJEB8599	ERS662981	SAMEA3261985	ERX709228	ERR765955	16S rRNA	V1-V3	TCGCACTAGT	AGAGTTTGATCMTGGCTCAG	GWATTACCGCGGCKGCTG	2012-03-12	31.11	-95.15	USA	0.3	88	19.0	253	Aquic Glossudalfs	Loblolly Pine, Beautyberry, Yaupon, Sweetgum, Oaks, Wax Myrtle	Cfa, Humid subtropical	OM2	C0	1	A horizon	11.4	0.8	0.0	4.5	1.3	15.7
TXC106B	TXC106	LP_TX_	Texas	TXC	Forest Soil	Whole Community DNA	PCR	Amplicon	pyrotag library	454 GS FLX Titanium	European Nucleotide Archive	https://www.ebi.ac.uk/ena/data/search?query=PRJEB8599	ERS662982	SAMEA3261986	ERX709229	ERR765956	16S rRNA	V1-V3	AGACGCACTC	AGAGTTTGATCMTGGCTCAG	GWATTACCGCGGCKGCTG	2012-03-12	31.11	-95.15	USA	0.1	88	19.0	253	Aquic Glossudalfs	Loblolly Pine, Beautyberry, Yaupon, Sweetgum, Oaks, Wax Myrtle	Cfa, Humid subtropical	OM2	C0	1	O horizon	20.8	NA	NA	4.6	NA	NA
TXC107B	TXC107	LP_TX_	Texas	TXC	Forest Soil	Whole Community DNA	PCR	Amplicon	pyrotag library	454 GS FLX Titanium	European Nucleotide Archive	https://www.ebi.ac.uk/ena/data/search?query=PRJEB8599	ERS662983	SAMEA3261987	ERX709230	ERR765957	16S rRNA	V1-V3	TCGATCACGT	AGAGTTTGATCMTGGCTCAG	GWATTACCGCGGCKGCTG	2012-03-12	31.11	-95.15	USA	0.1	88	19.0	253	Aquic Glossudalfs	Loblolly Pine, Beautyberry, Yaupon, Sweetgum, Oaks, Wax Myrtle	Cfa, Humid subtropical	OM2	C0	1	O horizon	15.3	NA	NA	4.5	NA	NA
TXC109B	TXC109	LP_TX_	Texas	TXC	Forest Soil	Whole Community DNA	PCR	Amplicon	pyrotag library	454 GS FLX Titanium	European Nucleotide Archive	https://www.ebi.ac.uk/ena/data/search?query=PRJEB8599	ERS662984	SAMEA3261988	ERX709231	ERR765958	16S rRNA	V1-V3	ACTACTATGT	AGAGTTTGATCMTGGCTCAG	GWATTACCGCGGCKGCTG	2012-03-12	31.11	-95.15	USA	0.3	88	19.0	253	Aquic Glossudalfs	Loblolly Pine, Beautyberry, Yaupon, Sweetgum, Oaks, Wax Myrtle	Cfa, Humid subtropical	OM1	C0	0	A horizon	30.3	0.8	0.0	4.8	1.3	16.3
TXC110B	TXC110	LP_TX_	Texas	TXC	Forest Soil	Whole Community DNA	PCR	Amplicon	pyrotag library	454 GS FLX Titanium	European Nucleotide Archive	https://www.ebi.ac.uk/ena/data/search?query=PRJEB8599	ERS662985	SAMEA3261989	ERX709232	ERR765959	16S rRNA	V1-V3	TACAGATCGT	AGAGTTTGATCMTGGCTCAG	GWATTACCGCGGCKGCTG	2012-03-12	31.11	-95.15	USA	0.3	88	19.0	253	Aquic Glossudalfs	Loblolly Pine, Beautyberry, Yaupon, Sweetgum, Oaks, Wax Myrtle	Cfa, Humid subtropical	OM1	C0	0	A horizon	11.4	0.8	0.0	4.9	1.3	16.3
TXC111B	TXC111	LP_TX_	Texas	TXC	Forest Soil	Whole Community DNA	PCR	Amplicon	pyrotag library	454 GS FLX Titanium	European Nucleotide Archive	https://www.ebi.ac.uk/ena/data/search?query=PRJEB8599	ERS662986	SAMEA3261990	ERX709233	ERR765960	16S rRNA	V1-V3	CATAGTAGTG	AGAGTTTGATCMTGGCTCAG	GWATTACCGCGGCKGCTG	2012-03-12	31.11	-95.15	USA	0.3	88	19.0	253	Aquic Glossudalfs	Loblolly Pine, Beautyberry, Yaupon, Sweetgum, Oaks, Wax Myrtle	Cfa, Humid subtropical	OM1	C0	0	A horizon	12.1	0.8	0.0	5.0	1.3	16.3
TXC112B	TXC112	LP_TX_	Texas	TXC	Forest Soil	Whole Community DNA	PCR	Amplicon	pyrotag library	454 GS FLX Titanium	European Nucleotide Archive	https://www.ebi.ac.uk/ena/data/search?query=PRJEB8599	ERS662987	SAMEA3261991	ERX709234	ERR765961	16S rRNA	V1-V3	TACAGATCGT	AGAGTTTGATCMTGGCTCAG	GWATTACCGCGGCKGCTG	2012-03-12	31.11	-95.15	USA	0.1	88	19.0	253	Aquic Glossudalfs	Loblolly Pine, Beautyberry, Yaupon, Sweetgum, Oaks, Wax Myrtle	Cfa, Humid subtropical	OM1	C0	0	O horizon	30.1	NA	NA	4.5	NA	NA
TXC113B	TXC113	LP_TX_	Texas	TXC	Forest Soil	Whole Community DNA	PCR	Amplicon	pyrotag library	454 GS FLX Titanium	European Nucleotide Archive	https://www.ebi.ac.uk/ena/data/search?query=PRJEB8599	ERS662988	SAMEA3261992	ERX709235	ERR765962	16S rRNA	V1-V3	TACACACACT	AGAGTTTGATCMTGGCTCAG	GWATTACCGCGGCKGCTG	2012-03-12	31.11	-95.15	USA	0.1	88	19.0	253	Aquic Glossudalfs	Loblolly Pine, Beautyberry, Yaupon, Sweetgum, Oaks, Wax Myrtle	Cfa, Humid subtropical	OM1	C0	0	O horizon	33.1	NA	NA	4.4	NA	NA
TXC114B	TXC114	LP_TX_	Texas	TXC	Forest Soil	Whole Community DNA	PCR	Amplicon	pyrotag library	454 GS FLX Titanium	European Nucleotide Archive	https://www.ebi.ac.uk/ena/data/search?query=PRJEB8599	ERS662989	SAMEA3261993	ERX709236	ERR765963	16S rRNA	V1-V3	ATCAGACACG	AGAGTTTGATCMTGGCTCAG	GWATTACCGCGGCKGCTG	2012-03-12	31.11	-95.15	USA	0.1	88	19.0	253	Aquic Glossudalfs	Loblolly Pine, Beautyberry, Yaupon, Sweetgum, Oaks, Wax Myrtle	Cfa, Humid subtropical	OM1	C0	0	O horizon	32.2	NA	NA	5.3	NA	NA
TXC115B	TXC115	LP_TX_	Texas	TXC	Forest Soil	Whole Community DNA	PCR	Amplicon	pyrotag library	454 GS FLX Titanium	European Nucleotide Archive	https://www.ebi.ac.uk/ena/data/search?query=PRJEB8599	ERS662990	SAMEA3261994	ERX709237	ERR765964	16S rRNA	V1-V3	TACGCTGTCT	AGAGTTTGATCMTGGCTCAG	GWATTACCGCGGCKGCTG	2012-03-12	31.11	-95.15	USA	0.3	88	19.0	253	Aquic Glossudalfs	Loblolly Pine, Beautyberry, Yaupon, Sweetgum, Oaks, Wax Myrtle	Cfa, Humid subtropical	OM1	C0	1	A horizon	26.9	0.8	0.0	5.3	1.3	16.3
TXC116B	TXC116	LP_TX_	Texas	TXC	Forest Soil	Whole Community DNA	PCR	Amplicon	pyrotag library	454 GS FLX Titanium	European Nucleotide Archive	https://www.ebi.ac.uk/ena/data/search?query=PRJEB8599	ERS662991	SAMEA3261995	ERX709238	ERR765965	16S rRNA	V1-V3	ACGCTCGACA	AGAGTTTGATCMTGGCTCAG	GWATTACCGCGGCKGCTG	2012-03-12	31.11	-95.15	USA	0.3	88	19.0	253	Aquic Glossudalfs	Loblolly Pine, Beautyberry, Yaupon, Sweetgum, Oaks, Wax Myrtle	Cfa, Humid subtropical	OM1	C0	1	A horizon	12.2	0.8	0.0	4.7	1.3	16.3
TXC118B	TXC118	LP_TX_	Texas	TXC	Forest Soil	Whole Community DNA	PCR	Amplicon	pyrotag library	454 GS FLX Titanium	European Nucleotide Archive	https://www.ebi.ac.uk/ena/data/search?query=PRJEB8599	ERS662992	SAMEA3261996	ERX709239	ERR765966	16S rRNA	V1-V3	TACACACACT	AGAGTTTGATCMTGGCTCAG	GWATTACCGCGGCKGCTG	2012-03-12	31.11	-95.15	USA	0.1	88	19.0	253	Aquic Glossudalfs	Loblolly Pine, Beautyberry, Yaupon, Sweetgum, Oaks, Wax Myrtle	Cfa, Humid subtropical	OM1	C0	1	O horizon	34.9	NA	NA	4.2	NA	NA
TXC119B	TXC119	LP_TX_	Texas	TXC	Forest Soil	Whole Community DNA	PCR	Amplicon	pyrotag library	454 GS FLX Titanium	European Nucleotide Archive	https://www.ebi.ac.uk/ena/data/search?query=PRJEB8599	ERS662993	SAMEA3261997	ERX709240	ERR765967	16S rRNA	V1-V3	CGAGAGATAC	AGAGTTTGATCMTGGCTCAG	GWATTACCGCGGCKGCTG	2012-03-12	31.11	-95.15	USA	0.1	88	19.0	253	Aquic Glossudalfs	Loblolly Pine, Beautyberry, Yaupon, Sweetgum, Oaks, Wax Myrtle	Cfa, Humid subtropical	OM1	C0	1	O horizon	26.5	NA	NA	4.5	NA	NA
TXC120B	TXC120	LP_TX_	Texas	TXC	Forest Soil	Whole Community DNA	PCR	Amplicon	pyrotag library	454 GS FLX Titanium	European Nucleotide Archive	https://www.ebi.ac.uk/ena/data/search?query=PRJEB8599	ERS662994	SAMEA3261998	ERX709241	ERR765968	16S rRNA	V1-V3	CACGCTACGT	AGAGTTTGATCMTGGCTCAG	GWATTACCGCGGCKGCTG	2012-03-12	31.11	-95.15	USA	0.1	88	19.0	253	Aquic Glossudalfs	Loblolly Pine, Beautyberry, Yaupon, Sweetgum, Oaks, Wax Myrtle	Cfa, Humid subtropical	OM1	C0	1	O horizon	23.9	NA	NA	4.3	NA	NA
TXC121B	TXC121	LP_TX_	Texas	TXC	Forest Soil	Whole Community DNA	PCR	Amplicon	pyrotag library	454 GS FLX Titanium	European Nucleotide Archive	https://www.ebi.ac.uk/ena/data/search?query=PRJEB8599	ERS662995	SAMEA3261999	ERX709242	ERR765969	16S rRNA	V1-V3	ACAGTATATA	AGAGTTTGATCMTGGCTCAG	GWATTACCGCGGCKGCTG	2012-03-12	31.11	-95.15	USA	0.3	88	19.0	253	Aquic Glossudalfs	Loblolly Pine, Beautyberry, Yaupon, Sweetgum, Oaks, Wax Myrtle	Cfa, Humid subtropical	OM2	C0	0	A horizon	4	0.69	0.04	4.75	1.36	15.65
TXC122B	TXC122	LP_TX_	Texas	TXC	Forest Soil	Whole Community DNA	PCR	Amplicon	pyrotag library	454 GS FLX Titanium	European Nucleotide Archive	https://www.ebi.ac.uk/ena/data/search?query=PRJEB8599	ERS662996	SAMEA3262000	ERX709243	ERR765970	16S rRNA	V1-V3	CACGCTACGT	AGAGTTTGATCMTGGCTCAG	GWATTACCGCGGCKGCTG	2012-03-12	31.11	-95.15	USA	0.3	88	19.0	253	Aquic Glossudalfs	Loblolly Pine, Beautyberry, Yaupon, Sweetgum, Oaks, Wax Myrtle	Cfa, Humid subtropical	OM2	C0	0	A horizon	19.4	0.7	0.0	4.7	1.4	15.7
TXC124B	TXC124	LP_TX_	Texas	TXC	Forest Soil	Whole Community DNA	PCR	Amplicon	pyrotag library	454 GS FLX Titanium	European Nucleotide Archive	https://www.ebi.ac.uk/ena/data/search?query=PRJEB8599	ERS662997	SAMEA3262001	ERX709244	ERR765971	16S rRNA	V1-V3	ACTGTACAGT	AGAGTTTGATCMTGGCTCAG	GWATTACCGCGGCKGCTG	2012-03-12	31.11	-95.15	USA	0.1	88	19.0	253	Aquic Glossudalfs	Loblolly Pine, Beautyberry, Yaupon, Sweetgum, Oaks, Wax Myrtle	Cfa, Humid subtropical	OM2	C0	0	O horizon	12.6	NA	NA	4.2	NA	NA
TXC125B	TXC125	LP_TX_	Texas	TXC	Forest Soil	Whole Community DNA	PCR	Amplicon	pyrotag library	454 GS FLX Titanium	European Nucleotide Archive	https://www.ebi.ac.uk/ena/data/search?query=PRJEB8599	ERS662998	SAMEA3262002	ERX709245	ERR765972	16S rRNA	V1-V3	CATAGTAGTG	AGAGTTTGATCMTGGCTCAG	GWATTACCGCGGCKGCTG	2012-03-12	31.11	-95.15	USA	0.1	88	19.0	253	Aquic Glossudalfs	Loblolly Pine, Beautyberry, Yaupon, Sweetgum, Oaks, Wax Myrtle	Cfa, Humid subtropical	OM2	C0	0	O horizon	12.6	NA	NA	4.3	NA	NA
TXC126B	TXC126	LP_TX_	Texas	TXC	Forest Soil	Whole Community DNA	PCR	Amplicon	pyrotag library	454 GS FLX Titanium	European Nucleotide Archive	https://www.ebi.ac.uk/ena/data/search?query=PRJEB8599	ERS662999	SAMEA3262003	ERX709246	ERR765973	16S rRNA	V1-V3	ACGCTCGACA	AGAGTTTGATCMTGGCTCAG	GWATTACCGCGGCKGCTG	2012-03-12	31.11	-95.15	USA	0.1	88	19.0	253	Aquic Glossudalfs	Loblolly Pine, Beautyberry, Yaupon, Sweetgum, Oaks, Wax Myrtle	Cfa, Humid subtropical	OM2	C0	0	O horizon	14.4	NA	NA	5.2	NA	NA
TXC127B	TXC127	LP_TX_	Texas	TXC	Forest Soil	Whole Community DNA	PCR	Amplicon	pyrotag library	454 GS FLX Titanium	European Nucleotide Archive	https://www.ebi.ac.uk/ena/data/search?query=PRJEB8599	ERS663000	SAMEA3262004	ERX709247	ERR765974	16S rRNA	V1-V3	ATAGAGTACT	AGAGTTTGATCMTGGCTCAG	GWATTACCGCGGCKGCTG	2012-03-12	31.11	-95.15	USA	0.3	88	19.0	253	Aquic Glossudalfs	Loblolly Pine, Beautyberry, Yaupon, Sweetgum, Oaks, Wax Myrtle	Cfa, Humid subtropical	OM2	C0	1	A horizon	18.9	0.7	0.0	5.0	1.4	15.7
TXC128B	TXC128	LP_TX_	Texas	TXC	Forest Soil	Whole Community DNA	PCR	Amplicon	pyrotag library	454 GS FLX Titanium	European Nucleotide Archive	https://www.ebi.ac.uk/ena/data/search?query=PRJEB8599	ERS663001	SAMEA3262005	ERX709248	ERR765975	16S rRNA	V1-V3	AGTACGCTAT	AGAGTTTGATCMTGGCTCAG	GWATTACCGCGGCKGCTG	2012-03-12	31.11	-95.15	USA	0.3	88	19.0	253	Aquic Glossudalfs	Loblolly Pine, Beautyberry, Yaupon, Sweetgum, Oaks, Wax Myrtle	Cfa, Humid subtropical	OM2	C0	1	A horizon	20.6	0.7	0.0	5.1	1.4	15.7
TXC129B	TXC129	LP_TX_	Texas	TXC	Forest Soil	Whole Community DNA	PCR	Amplicon	pyrotag library	454 GS FLX Titanium	European Nucleotide Archive	https://www.ebi.ac.uk/ena/data/search?query=PRJEB8599	ERS663002	SAMEA3262006	ERX709249	ERR765976	16S rRNA	V1-V3	CTCGCGTGTC	AGAGTTTGATCMTGGCTCAG	GWATTACCGCGGCKGCTG	2012-03-12	31.11	-95.15	USA	0.3	88	19.0	253	Aquic Glossudalfs	Loblolly Pine, Beautyberry, Yaupon, Sweetgum, Oaks, Wax Myrtle	Cfa, Humid subtropical	OM2	C0	1	A horizon	20.5	0.7	0.0	5.1	1.4	15.7
TXC130B	TXC130	LP_TX_	Texas	TXC	Forest Soil	Whole Community DNA	PCR	Amplicon	pyrotag library	454 GS FLX Titanium	European Nucleotide Archive	https://www.ebi.ac.uk/ena/data/search?query=PRJEB8599	ERS663003	SAMEA3262007	ERX709250	ERR765977	16S rRNA	V1-V3	TCTCTATGCG	AGAGTTTGATCMTGGCTCAG	GWATTACCGCGGCKGCTG	2012-03-12	31.11	-95.15	USA	0.1	88	19.0	253	Aquic Glossudalfs	Loblolly Pine, Beautyberry, Yaupon, Sweetgum, Oaks, Wax Myrtle	Cfa, Humid subtropical	OM2	C0	1	O horizon	15.4	NA	NA	4.4	NA	NA
TXC131B	TXC131	LP_TX_	Texas	TXC	Forest Soil	Whole Community DNA	PCR	Amplicon	pyrotag library	454 GS FLX Titanium	European Nucleotide Archive	https://www.ebi.ac.uk/ena/data/search?query=PRJEB8599	ERS663004	SAMEA3262008	ERX709251	ERR765978	16S rRNA	V1-V3	TCTAGCGACT	AGAGTTTGATCMTGGCTCAG	GWATTACCGCGGCKGCTG	2012-03-12	31.11	-95.15	USA	0.1	88	19.0	253	Aquic Glossudalfs	Loblolly Pine, Beautyberry, Yaupon, Sweetgum, Oaks, Wax Myrtle	Cfa, Humid subtropical	OM2	C0	1	O horizon	19	NA	NA	4.86	NA	NA
TXC132B	TXC132	LP_TX_	Texas	TXC	Forest Soil	Whole Community DNA	PCR	Amplicon	pyrotag library	454 GS FLX Titanium	European Nucleotide Archive	https://www.ebi.ac.uk/ena/data/search?query=PRJEB8599	ERS663005	SAMEA3262009	ERX709252	ERR765979	16S rRNA	V1-V3	ACTGTACAGT	AGAGTTTGATCMTGGCTCAG	GWATTACCGCGGCKGCTG	2012-03-12	31.11	-95.15	USA	0.1	88	19.0	253	Aquic Glossudalfs	Loblolly Pine, Beautyberry, Yaupon, Sweetgum, Oaks, Wax Myrtle	Cfa, Humid subtropical	OM2	C0	1	O horizon	11.7	NA	NA	4.1	NA	NA
TXC133B	TXC133	LP_TX_	Texas	TXC	Forest Soil	Whole Community DNA	PCR	Amplicon	pyrotag library	454 GS FLX Titanium	European Nucleotide Archive	https://www.ebi.ac.uk/ena/data/search?query=PRJEB8599	ERS663006	SAMEA3262010	ERX709253	ERR765980	16S rRNA	V1-V3	TGATACGTCT	AGAGTTTGATCMTGGCTCAG	GWATTACCGCGGCKGCTG	2012-03-12	31.11	-95.15	USA	0.3	88	19.0	253	Aquic Glossudalfs	Loblolly Pine, Beautyberry, Yaupon, Sweetgum, Oaks, Wax Myrtle	Cfa, Humid subtropical	OM3	C0	0	A horizon	18.9	0.7	0.0	4.3	1.3	16.4
TXC134B	TXC134	LP_TX_	Texas	TXC	Forest Soil	Whole Community DNA	PCR	Amplicon	pyrotag library	454 GS FLX Titanium	European Nucleotide Archive	https://www.ebi.ac.uk/ena/data/search?query=PRJEB8599	ERS663007	SAMEA3262011	ERX709254	ERR765981	16S rRNA	V1-V3	ACGCGATCGA	AGAGTTTGATCMTGGCTCAG	GWATTACCGCGGCKGCTG	2012-03-12	31.11	-95.15	USA	0.3	88	19.0	253	Aquic Glossudalfs	Loblolly Pine, Beautyberry, Yaupon, Sweetgum, Oaks, Wax Myrtle	Cfa, Humid subtropical	OM3	C0	0	A horizon	19.8	0.7	0.0	4.4	1.3	16.4
TXC135B	TXC135	LP_TX_	Texas	TXC	Forest Soil	Whole Community DNA	PCR	Amplicon	pyrotag library	454 GS FLX Titanium	European Nucleotide Archive	https://www.ebi.ac.uk/ena/data/search?query=PRJEB8599	ERS663008	SAMEA3262012	ERX709255	ERR765982	16S rRNA	V1-V3	TACAGATCGT	AGAGTTTGATCMTGGCTCAG	GWATTACCGCGGCKGCTG	2012-03-12	31.11	-95.15	USA	0.3	88	19.0	253	Aquic Glossudalfs	Loblolly Pine, Beautyberry, Yaupon, Sweetgum, Oaks, Wax Myrtle	Cfa, Humid subtropical	OM3	C0	0	A horizon	20.9	0.7	0.0	4.6	1.3	16.4
TXC136B	TXC136	LP_TX_	Texas	TXC	Forest Soil	Whole Community DNA	PCR	Amplicon	pyrotag library	454 GS FLX Titanium	European Nucleotide Archive	https://www.ebi.ac.uk/ena/data/search?query=PRJEB8599	ERS663009	SAMEA3262013	ERX709256	ERR765983	16S rRNA	V1-V3	TGACGTATGT	AGAGTTTGATCMTGGCTCAG	GWATTACCGCGGCKGCTG	2012-03-12	31.11	-95.15	USA	0.1	88	19.0	253	Aquic Glossudalfs	Loblolly Pine, Beautyberry, Yaupon, Sweetgum, Oaks, Wax Myrtle	Cfa, Humid subtropical	OM3	C0	0	O horizon	36.3	12.3	0.4	NA	NA	27.4
TXC137B	TXC137	LP_TX_	Texas	TXC	Forest Soil	Whole Community DNA	PCR	Amplicon	pyrotag library	454 GS FLX Titanium	European Nucleotide Archive	https://www.ebi.ac.uk/ena/data/search?query=PRJEB8599	ERS663010	SAMEA3262014	ERX709257	ERR765984	16S rRNA	V1-V3	TACAGATCGT	AGAGTTTGATCMTGGCTCAG	GWATTACCGCGGCKGCTG	2012-03-12	31.11	-95.15	USA	0.1	88	19.0	253	Aquic Glossudalfs	Loblolly Pine, Beautyberry, Yaupon, Sweetgum, Oaks, Wax Myrtle	Cfa, Humid subtropical	OM3	C0	0	O horizon	28.1	9.0	0.3	4.8	NA	27.0
TXC138B	TXC138	LP_TX_	Texas	TXC	Forest Soil	Whole Community DNA	PCR	Amplicon	pyrotag library	454 GS FLX Titanium	European Nucleotide Archive	https://www.ebi.ac.uk/ena/data/search?query=PRJEB8599	ERS663011	SAMEA3262015	ERX709258	ERR765985	16S rRNA	V1-V3	TACGCTGTCT	AGAGTTTGATCMTGGCTCAG	GWATTACCGCGGCKGCTG	2012-03-12	31.11	-95.15	USA	0.1	88	19.0	253	Aquic Glossudalfs	Loblolly Pine, Beautyberry, Yaupon, Sweetgum, Oaks, Wax Myrtle	Cfa, Humid subtropical	OM3	C0	0	O horizon	33.2	12.3	0.5	4.8	NA	27.2
TXC139B	TXC139	LP_TX_	Texas	TXC	Forest Soil	Whole Community DNA	PCR	Amplicon	pyrotag library	454 GS FLX Titanium	European Nucleotide Archive	https://www.ebi.ac.uk/ena/data/search?query=PRJEB8599	ERS663012	SAMEA3262016	ERX709259	ERR765986	16S rRNA	V1-V3	TCACGTACTA	AGAGTTTGATCMTGGCTCAG	GWATTACCGCGGCKGCTG	2012-03-12	31.11	-95.15	USA	0.3	88	19.0	253	Aquic Glossudalfs	Loblolly Pine, Beautyberry, Yaupon, Sweetgum, Oaks, Wax Myrtle	Cfa, Humid subtropical	OM3	C0	1	A horizon	20.3	0.7	0.0	4.4	1.3	16.4
TXC140B	TXC140	LP_TX_	Texas	TXC	Forest Soil	Whole Community DNA	PCR	Amplicon	pyrotag library	454 GS FLX Titanium	European Nucleotide Archive	https://www.ebi.ac.uk/ena/data/search?query=PRJEB8599	ERS663013	SAMEA3262017	ERX709260	ERR765987	16S rRNA	V1-V3	ATAGAGTACT	AGAGTTTGATCMTGGCTCAG	GWATTACCGCGGCKGCTG	2012-03-12	31.11	-95.15	USA	0.3	88	19.0	253	Aquic Glossudalfs	Loblolly Pine, Beautyberry, Yaupon, Sweetgum, Oaks, Wax Myrtle	Cfa, Humid subtropical	OM3	C0	1	A horizon	20.5	0.7	0.0	4.5	1.3	16.4
TXC141B	TXC141	LP_TX_	Texas	TXC	Forest Soil	Whole Community DNA	PCR	Amplicon	pyrotag library	454 GS FLX Titanium	European Nucleotide Archive	https://www.ebi.ac.uk/ena/data/search?query=PRJEB8599	ERS663014	SAMEA3262018	ERX709261	ERR765988	16S rRNA	V1-V3	ACGCGATCGA	AGAGTTTGATCMTGGCTCAG	GWATTACCGCGGCKGCTG	2012-03-12	31.11	-95.15	USA	0.3	88	19.0	253	Aquic Glossudalfs	Loblolly Pine, Beautyberry, Yaupon, Sweetgum, Oaks, Wax Myrtle	Cfa, Humid subtropical	OM3	C0	1	A horizon	21.2	0.7	0.0	4.9	1.3	16.4
TXC142B	TXC142	LP_TX_	Texas	TXC	Forest Soil	Whole Community DNA	PCR	Amplicon	pyrotag library	454 GS FLX Titanium	European Nucleotide Archive	https://www.ebi.ac.uk/ena/data/search?query=PRJEB8599	ERS663015	SAMEA3262019	ERX709262	ERR765989	16S rRNA	V1-V3	CTCGCGTGTC	AGAGTTTGATCMTGGCTCAG	GWATTACCGCGGCKGCTG	2012-03-12	31.11	-95.15	USA	0.1	88	19.0	253	Aquic Glossudalfs	Loblolly Pine, Beautyberry, Yaupon, Sweetgum, Oaks, Wax Myrtle	Cfa, Humid subtropical	OM3	C0	1	O horizon	24.9	NA	NA	4.1	NA	NA
TXC143B	TXC143	LP_TX_	Texas	TXC	Forest Soil	Whole Community DNA	PCR	Amplicon	pyrotag library	454 GS FLX Titanium	European Nucleotide Archive	https://www.ebi.ac.uk/ena/data/search?query=PRJEB8599	ERS663016	SAMEA3262020	ERX709263	ERR765990	16S rRNA	V1-V3	AGACGCACTC	AGAGTTTGATCMTGGCTCAG	GWATTACCGCGGCKGCTG	2012-03-12	31.11	-95.15	USA	0.1	88	19.0	253	Aquic Glossudalfs	Loblolly Pine, Beautyberry, Yaupon, Sweetgum, Oaks, Wax Myrtle	Cfa, Humid subtropical	OM3	C0	1	O horizon	25.3	NA	NA	4.3	NA	NA
TXC144B	TXC144	LP_TX_	Texas	TXC	Forest Soil	Whole Community DNA	PCR	Amplicon	pyrotag library	454 GS FLX Titanium	European Nucleotide Archive	https://www.ebi.ac.uk/ena/data/search?query=PRJEB8599	ERS663017	SAMEA3262021	ERX709264	ERR765991	16S rRNA	V1-V3	ATACGACGTA	AGAGTTTGATCMTGGCTCAG	GWATTACCGCGGCKGCTG	2012-03-12	31.11	-95.15	USA	0.1	88	19.0	253	Aquic Glossudalfs	Loblolly Pine, Beautyberry, Yaupon, Sweetgum, Oaks, Wax Myrtle	Cfa, Humid subtropical	OM3	C0	1	O horizon	33.0	NA	NA	4.3	NA	NA
TXC145B	TXC145	LP_TX_	Texas	TXC	Forest Soil	Whole Community DNA	PCR	Amplicon	pyrotag library	454 GS FLX Titanium	European Nucleotide Archive	https://www.ebi.ac.uk/ena/data/search?query=PRJEB8599	ERS663018	SAMEA3262022	ERX709265	ERR765992	16S rRNA	V1-V3	ACAGTATATA	AGAGTTTGATCMTGGCTCAG	GWATTACCGCGGCKGCTG	2012-03-12	31.11	-95.15	USA	0.3	88	19.0	253	Aquic Glossudalfs	Loblolly Pine, Beautyberry, Yaupon, Sweetgum, Oaks, Wax Myrtle	Cfa, Humid subtropical	REF	REF	0	A horizon	17.4	NA	NA	NA	NA	NA
TXC146B	TXC146	LP_TX_	Texas	TXC	Forest Soil	Whole Community DNA	PCR	Amplicon	pyrotag library	454 GS FLX Titanium	European Nucleotide Archive	https://www.ebi.ac.uk/ena/data/search?query=PRJEB8599	ERS663019	SAMEA3262023	ERX709266	ERR765993	16S rRNA	V1-V3	AGTACGCTAT	AGAGTTTGATCMTGGCTCAG	GWATTACCGCGGCKGCTG	2012-03-12	31.11	-95.15	USA	0.3	88	19.0	253	Aquic Glossudalfs	Loblolly Pine, Beautyberry, Yaupon, Sweetgum, Oaks, Wax Myrtle	Cfa, Humid subtropical	REF	REF	0	A horizon	14.3	NA	NA	NA	NA	NA
TXC147B	TXC147	LP_TX_	Texas	TXC	Forest Soil	Whole Community DNA	PCR	Amplicon	pyrotag library	454 GS FLX Titanium	European Nucleotide Archive	https://www.ebi.ac.uk/ena/data/search?query=PRJEB8599	ERS663020	SAMEA3262024	ERX709267	ERR765994	16S rRNA	V1-V3	TGTGAGTAGT	AGAGTTTGATCMTGGCTCAG	GWATTACCGCGGCKGCTG	2012-03-12	31.11	-95.15	USA	0.3	88	19.0	253	Aquic Glossudalfs	Loblolly Pine, Beautyberry, Yaupon, Sweetgum, Oaks, Wax Myrtle	Cfa, Humid subtropical	REF	REF	0	A horizon	17.0	NA	NA	NA	NA	NA
TXC148B	TXC148	LP_TX_	Texas	TXC	Forest Soil	Whole Community DNA	PCR	Amplicon	pyrotag library	454 GS FLX Titanium	European Nucleotide Archive	https://www.ebi.ac.uk/ena/data/search?query=PRJEB8599	ERS663021	SAMEA3262025	ERX709268	ERR765995	16S rRNA	V1-V3	ATCAGACACG	AGAGTTTGATCMTGGCTCAG	GWATTACCGCGGCKGCTG	2012-03-12	31.11	-95.15	USA	0.1	88	19.0	253	Aquic Glossudalfs	Loblolly Pine, Beautyberry, Yaupon, Sweetgum, Oaks, Wax Myrtle	Cfa, Humid subtropical	REF	REF	0	O horizon	41.1	25.1	0.8	4.1	NA	32.3
TXC149B	TXC149	LP_TX_	Texas	TXC	Forest Soil	Whole Community DNA	PCR	Amplicon	pyrotag library	454 GS FLX Titanium	European Nucleotide Archive	https://www.ebi.ac.uk/ena/data/search?query=PRJEB8599	ERS663022	SAMEA3262026	ERX709269	ERR765996	16S rRNA	V1-V3	ACGAGTGCGT	AGAGTTTGATCMTGGCTCAG	GWATTACCGCGGCKGCTG	2012-03-12	31.11	-95.15	USA	0.1	88	19.0	253	Aquic Glossudalfs	Loblolly Pine, Beautyberry, Yaupon, Sweetgum, Oaks, Wax Myrtle	Cfa, Humid subtropical	REF	REF	0	O horizon	31.2	18.2	0.7	4.7	NA	27.2
TXC150B	TXC150	LP_TX_	Texas	TXC	Forest Soil	Whole Community DNA	PCR	Amplicon	pyrotag library	454 GS FLX Titanium	European Nucleotide Archive	https://www.ebi.ac.uk/ena/data/search?query=PRJEB8599	ERS663023	SAMEA3262027	ERX709270	ERR765997	16S rRNA	V1-V3	TCTAGCGACT	AGAGTTTGATCMTGGCTCAG	GWATTACCGCGGCKGCTG	2012-03-12	31.11	-95.15	USA	0.1	88	19.0	253	Aquic Glossudalfs	Loblolly Pine, Beautyberry, Yaupon, Sweetgum, Oaks, Wax Myrtle	Cfa, Humid subtropical	REF	REF	0	O horizon	36.3	13.8	0.5	4.2	NA	26.8
SL-REF-LFH-1	SL-REF-LFH-1	SBS_BC_	British Columbia	SL	Forest Soil	Whole Community DNA	PCR	Amplicon	pyrotag library	454 GS FLX Titanium	European Nucleotide Archive	https://www.ebi.ac.uk/ena/data/search?query=PRJEB12501	ERS1434280	SAMEA4535101	ERX1790570	ERR1720618	16S rRNA	V6-V8	CGTCGATCTC	YAAAKGAATTGRCGG	ACGGGCGGTGTGTRC	7/15/2007	52.32	-121.92	Canada	0.395	1050	4.1	146–193	Orthic Gray Luvisols	Lodgepole pine	Dfc, Boreal cool summer	REF	C0	0	LFH	NA	14.4	0.4	4.3	NA	27.2
SL-REF-Ahe-1	SL-REF-Ahe-1	SBS_BC_	British Columbia	SL	Forest Soil	Whole Community DNA	PCR	Amplicon	pyrotag library	454 GS FLX Titanium	European Nucleotide Archive	https://www.ebi.ac.uk/ena/data/search?query=PRJEB12501	ERS1434281	SAMEA4535102	ERX1790571	ERR1720619	16S rRNA	V6-V8	CTACGACTGC	YAAAKGAATTGRCGG	ACGGGCGGTGTGTRC	7/15/2007	52.32	-121.92	Canada	0.545	1050	4.1	146–193	Orthic Gray Luvisols	Lodgepole pine	Dfc, Boreal cool summer	REF	C0	0	Ahe	NA	1.8	0.1	4.7	NA	26.8
SL-REF-Ae-1	SL-REF-Ae-1	SBS_BC_	British Columbia	SL	Forest Soil	Whole Community DNA	PCR	Amplicon	pyrotag library	454 GS FLX Titanium	European Nucleotide Archive	https://www.ebi.ac.uk/ena/data/search?query=PRJEB12501	ERS1434282	SAMEA4535103	ERX1790572	ERR1720620	16S rRNA	V6-V8	CTAGTCACTC	YAAAKGAATTGRCGG	ACGGGCGGTGTGTRC	7/15/2007	52.32	-121.92	Canada	0.003	1050	4.1	146–193	Orthic Gray Luvisols	Lodgepole pine	Dfc, Boreal cool summer	REF	C0	0	Ae	NA	1.0	0.1	5.4	NA	36.0
SL-REF-AB-1	SL-REF-AB-1	SBS_BC_	British Columbia	SL	Forest Soil	Whole Community DNA	PCR	Amplicon	pyrotag library	454 GS FLX Titanium	European Nucleotide Archive	https://www.ebi.ac.uk/ena/data/search?query=PRJEB12501	ERS1434283	SAMEA4535104	ERX1790573	ERR1720621	16S rRNA	V6-V8	CTCTACGCTC	YAAAKGAATTGRCGG	ACGGGCGGTGTGTRC	7/15/2007	52.32	-121.92	Canada	0.062	1050	4.1	146–193	Orthic Gray Luvisols	Lodgepole pine	Dfc, Boreal cool summer	REF	C0	0	AB	NA	0.7	0.1	5.9	NA	22.5
SL-REF-Bt-1	SL-REF-Bt-1	SBS_BC_	British Columbia	SL	Forest Soil	Whole Community DNA	PCR	Amplicon	pyrotag library	454 GS FLX Titanium	European Nucleotide Archive	https://www.ebi.ac.uk/ena/data/search?query=PRJEB12501	ERS1434284	SAMEA4535105	ERX1790574	ERR1720622	16S rRNA	V6-V8	TAGCGCGCGC	YAAAKGAATTGRCGG	ACGGGCGGTGTGTRC	7/15/2007	52.32	-121.92	Canada	0.333	1050	4.1	146–193	Orthic Gray Luvisols	Lodgepole pine	Dfc, Boreal cool summer	REF	C0	0	Bt	NA	0.9	0.1	6.7	NA	19.0
SL-REF-LFH-2	SL-REF-LFH-2	SBS_BC_	British Columbia	SL	Forest Soil	Whole Community DNA	PCR	Amplicon	pyrotag library	454 GS FLX Titanium	European Nucleotide Archive	https://www.ebi.ac.uk/ena/data/search?query=PRJEB12501	ERS1434285	SAMEA4535106	ERX1790575	ERR1720623	16S rRNA	V6-V8	TAGCTCTATC	YAAAKGAATTGRCGG	ACGGGCGGTGTGTRC	7/15/2007	52.32	-121.92	Canada	0.528	1050	4.1	146–193	Orthic Gray Luvisols	Lodgepole pine	Dfc, Boreal cool summer	REF	C0	0	LFH	NA	26.6	0.6	4.5	NA	13.4
SL-REF-Ahe-2	SL-REF-Ahe-2	SBS_BC_	British Columbia	SL	Forest Soil	Whole Community DNA	PCR	Amplicon	pyrotag library	454 GS FLX Titanium	European Nucleotide Archive	https://www.ebi.ac.uk/ena/data/search?query=PRJEB12501	ERS1434286	SAMEA4535107	ERX1790576	ERR1720624	16S rRNA	V6-V8	TATAGACATC	YAAAKGAATTGRCGG	ACGGGCGGTGTGTRC	7/15/2007	52.32	-121.92	Canada	0.002	1050	4.1	146–193	Orthic Gray Luvisols	Lodgepole pine	Dfc, Boreal cool summer	REF	C0	0	Ahe	NA	8.7	0.3	5.1	NA	14.8
SL-REF-Ae-2	SL-REF-Ae-2	SBS_BC_	British Columbia	SL	Forest Soil	Whole Community DNA	PCR	Amplicon	pyrotag library	454 GS FLX Titanium	European Nucleotide Archive	https://www.ebi.ac.uk/ena/data/search?query=PRJEB12501	ERS1434287	SAMEA4535108	ERX1790577	ERR1720625	16S rRNA	V6-V8	TATGATACGC	YAAAKGAATTGRCGG	ACGGGCGGTGTGTRC	7/15/2007	52.32	-121.92	Canada	0.067	1050	4.1	146–193	Orthic Gray Luvisols	Lodgepole pine	Dfc, Boreal cool summer	REF	C0	0	Ae	NA	0.7	0.1	6.2	NA	48.4
SL-REF-AB-2	SL-REF-AB-2	SBS_BC_	British Columbia	SL	Forest Soil	Whole Community DNA	PCR	Amplicon	pyrotag library	454 GS FLX Titanium	European Nucleotide Archive	https://www.ebi.ac.uk/ena/data/search?query=PRJEB12501	ERS1434288	SAMEA4535109	ERX1790578	ERR1720626	16S rRNA	V6-V8	TCACTCATAC	YAAAKGAATTGRCGG	ACGGGCGGTGTGTRC	7/15/2007	52.32	-121.92	Canada	0.217	1050	4.1	146–193	Orthic Gray Luvisols	Lodgepole pine	Dfc, Boreal cool summer	REF	C0	0	AB	NA	1.1	0.1	6.3	NA	34.8
SL-REF-Bt-2	SL-REF-Bt-2	SBS_BC_	British Columbia	SL	Forest Soil	Whole Community DNA	PCR	Amplicon	pyrotag library	454 GS FLX Titanium	European Nucleotide Archive	https://www.ebi.ac.uk/ena/data/search?query=PRJEB12501	ERS1434289	SAMEA4535110	ERX1790579	ERR1720627	16S rRNA	V6-V8	TCATCGAGTC	YAAAKGAATTGRCGG	ACGGGCGGTGTGTRC	7/15/2007	52.32	-121.92	Canada	0.597	1050	4.1	146–193	Orthic Gray Luvisols	Lodgepole pine	Dfc, Boreal cool summer	REF	C0	0	Bt	NA	0.7	0.1	6.9	NA	14.6
SL-REF-LFH-3	SL-REF-LFH-3	SBS_BC_	British Columbia	SL	Forest Soil	Whole Community DNA	PCR	Amplicon	pyrotag library	454 GS FLX Titanium	European Nucleotide Archive	https://www.ebi.ac.uk/ena/data/search?query=PRJEB12501	ERS1434290	SAMEA4535111	ERX1790580	ERR1720628	16S rRNA	V6-V8	TCGAGCTCTC	YAAAKGAATTGRCGG	ACGGGCGGTGTGTRC	7/15/2007	52.32	-121.92	Canada	0.002	1050	4.1	146–193	Orthic Gray Luvisols	Lodgepole pine	Dfc, Boreal cool summer	REF	C0	0	LFH	NA	27.5	0.6	5.6	NA	21.2
SL-REF-Ae-3	SL-REF-Ae-3	SBS_BC_	British Columbia	SL	Forest Soil	Whole Community DNA	PCR	Amplicon	pyrotag library	454 GS FLX Titanium	European Nucleotide Archive	https://www.ebi.ac.uk/ena/data/search?query=PRJEB12501	ERS1434291	SAMEA4535112	ERX1790581	ERR1720629	16S rRNA	V6-V8	TCGCAGACAC	YAAAKGAATTGRCGG	ACGGGCGGTGTGTRC	7/15/2007	52.32	-121.92	Canada	0.042	1050	4.1	146–193	Orthic Gray Luvisols	Lodgepole pine	Dfc, Boreal cool summer	REF	C0	0	Ae	NA	1.1	0.1	5.7	NA	14.8
SL-REF-AB-3	SL-REF-AB-3	SBS_BC_	British Columbia	SL	Forest Soil	Whole Community DNA	PCR	Amplicon	pyrotag library	454 GS FLX Titanium	European Nucleotide Archive	https://www.ebi.ac.uk/ena/data/search?query=PRJEB12501	ERS1434292	SAMEA4535113	ERX1790582	ERR1720630	16S rRNA	V6-V8	TCTGTCTCGC	YAAAKGAATTGRCGG	ACGGGCGGTGTGTRC	7/15/2007	52.32	-121.92	Canada	0.272	1050	4.1	146–193	Orthic Gray Luvisols	Lodgepole pine, Interior spruce	Dfc, Boreal cool summer	REF	C0	0	AB	NA	0.6	0.1	5.8	NA	45.0
SL-REF-Bt-3	SL-REF-Bt-3	SBS_BC_	British Columbia	SL	Forest Soil	Whole Community DNA	PCR	Amplicon	pyrotag library	454 GS FLX Titanium	European Nucleotide Archive	https://www.ebi.ac.uk/ena/data/search?query=PRJEB12501	ERS1434293	SAMEA4535114	ERX1790583	ERR1720631	16S rRNA	V6-V8	TGAGTGACGC	YAAAKGAATTGRCGG	ACGGGCGGTGTGTRC	7/15/2007	52.32	-121.92	Canada	0.492	1050	4.1	146–193	Orthic Gray Luvisols	Lodgepole pine, Interior spruce	Dfc, Boreal cool summer	REF	C0	0	Bt	NA	0.6	0.1	6.6	NA	14.0
SL-OM3-LFH-1	SL-OM3-LFH-1	SBS_BC_	British Columbia	SL	Forest Soil	Whole Community DNA	PCR	Amplicon	pyrotag library	454 GS FLX Titanium	European Nucleotide Archive	https://www.ebi.ac.uk/ena/data/search?query=PRJEB12501	ERS1434294	SAMEA4535115	ERX1790584	ERR1720632	16S rRNA	V6-V8	AGCGACTAGC	YAAAKGAATTGRCGG	ACGGGCGGTGTGTRC	7/15/2007	52.32	-121.92	Canada	0.02	1050	4.1	146–193	Orthic Gray Luvisols	Lodgepole pine, Interior spruce	Dfc, Boreal cool summer	OM3	C0	0	LFH	NA	5.7	0.2	5.9	NA	12.0
SL-OM3-Ae-1	SL-OM3-Ae-1	SBS_BC_	British Columbia	SL	Forest Soil	Whole Community DNA	PCR	Amplicon	pyrotag library	454 GS FLX Titanium	European Nucleotide Archive	https://www.ebi.ac.uk/ena/data/search?query=PRJEB12501	ERS1434295	SAMEA4535116	ERX1790585	ERR1720633	16S rRNA	V6-V8	AGTAGTGATC	YAAAKGAATTGRCGG	ACGGGCGGTGTGTRC	7/15/2007	52.32	-121.92	Canada	0.065	1050	4.1	146–193	Orthic Gray Luvisols	Lodgepole pine, Interior spruce	Dfc, Boreal cool summer	OM3	C0	0	Ae	NA	0.3	0.0	6.1	NA	12.2
SL-OM3-AB-1	SL-OM3-AB-1	SBS_BC_	British Columbia	SL	Forest Soil	Whole Community DNA	PCR	Amplicon	pyrotag library	454 GS FLX Titanium	European Nucleotide Archive	https://www.ebi.ac.uk/ena/data/search?query=PRJEB12501	ERS1434296	SAMEA4535117	ERX1790586	ERR1720634	16S rRNA	V6-V8	AGTGTATGTC	YAAAKGAATTGRCGG	ACGGGCGGTGTGTRC	7/15/2007	52.32	-121.92	Canada	0.13	1050	4.1	146–193	Orthic Gray Luvisols	Lodgepole pine, Interior spruce	Dfc, Boreal cool summer	OM3	C0	0	Bt	NA	0.4	0.0	6.5	NA	27.3
SL-OM3-Bt-1	SL-OM3-Bt-1	SBS_BC_	British Columbia	SL	Forest Soil	Whole Community DNA	PCR	Amplicon	pyrotag library	454 GS FLX Titanium	European Nucleotide Archive	https://www.ebi.ac.uk/ena/data/search?query=PRJEB12501	ERS1434297	SAMEA4535118	ERX1790587	ERR1720635	16S rRNA	V6-V8	ATAGATAGAC	YAAAKGAATTGRCGG	ACGGGCGGTGTGTRC	7/15/2007	52.32	-121.92	Canada	0.385	1050	4.1	146–193	Orthic Gray Luvisols	Lodgepole pine, Interior spruce	Dfc, Boreal cool summer	OM3	C0	0	Bt	NA	0.6	0.0	6.9	NA	9.3
SL-OM3-LFH-2	SL-OM3-LFH-2	SBS_BC_	British Columbia	SL	Forest Soil	Whole Community DNA	PCR	Amplicon	pyrotag library	454 GS FLX Titanium	European Nucleotide Archive	https://www.ebi.ac.uk/ena/data/search?query=PRJEB12501	ERS1434298	SAMEA4535119	ERX1790588	ERR1720636	16S rRNA	V6-V8	ATATAGTCGC	YAAAKGAATTGRCGG	ACGGGCGGTGTGTRC	7/15/2007	52.32	-121.92	Canada	0.62	1050	4.1	146–193	Orthic Gray Luvisols	Lodgepole pine, Interior spruce	Dfc, Boreal cool summer	OM3	C0	0	LFH	NA	2.3	0.1	6.2	NA	12.0
SL-OM3-Ae-2	SL-OM3-Ae-2	SBS_BC_	British Columbia	SL	Forest Soil	Whole Community DNA	PCR	Amplicon	pyrotag library	454 GS FLX Titanium	European Nucleotide Archive	https://www.ebi.ac.uk/ena/data/search?query=PRJEB12501	ERS1434299	SAMEA4535120	ERX1790589	ERR1720637	16S rRNA	V6-V8	ATCTACTGAC	YAAAKGAATTGRCGG	ACGGGCGGTGTGTRC	7/15/2007	52.32	-121.92	Canada	0.02	1050	4.1	146–193	Orthic Gray Luvisols	Lodgepole pine, Interior spruce	Dfc, Boreal cool summer	OM3	C0	0	Ae	NA	1.1	0.1	6.1	NA	14.5
SL-OM3-AB-2	SL-OM3-AB-2	SBS_BC_	British Columbia	SL	Forest Soil	Whole Community DNA	PCR	Amplicon	pyrotag library	454 GS FLX Titanium	European Nucleotide Archive	https://www.ebi.ac.uk/ena/data/search?query=PRJEB12501	ERS1434300	SAMEA4535121	ERX1790590	ERR1720638	16S rRNA	V6-V8	CACGTAGATC	YAAAKGAATTGRCGG	ACGGGCGGTGTGTRC	7/15/2007	52.32	-121.92	Canada	0.075	1050	4.1	146–193	Orthic Gray Luvisols	Lodgepole pine, Interior spruce	Dfc, Boreal cool summer	OM3	C0	0	AB	NA	0.4	0.0	6.3	NA	22.6
SL-OM3-Bt-2	SL-OM3-Bt-2	SBS_BC_	British Columbia	SL	Forest Soil	Whole Community DNA	PCR	Amplicon	pyrotag library	454 GS FLX Titanium	European Nucleotide Archive	https://www.ebi.ac.uk/ena/data/search?query=PRJEB12501	ERS1434301	SAMEA4535122	ERX1790591	ERR1720639	16S rRNA	V6-V8	CACGTGTCGC	YAAAKGAATTGRCGG	ACGGGCGGTGTGTRC	7/15/2007	52.32	-121.92	Canada	0.2	1050	4.1	146–193	Orthic Gray Luvisols	Lodgepole pine, Interior spruce	Dfc, Boreal cool summer	OM3	C0	0	Bt	NA	0.7	0.1	6.8	NA	18.8
SL-OM3-LFH-3	SL-OM3-LFH-3	SBS_BC_	British Columbia	SL	Forest Soil	Whole Community DNA	PCR	Amplicon	pyrotag library	454 GS FLX Titanium	European Nucleotide Archive	https://www.ebi.ac.uk/ena/data/search?query=PRJEB12501	ERS1434302	SAMEA4535123	ERX1790592	ERR1720640	16S rRNA	V6-V8	CATACTCTAC	YAAAKGAATTGRCGG	ACGGGCGGTGTGTRC	7/15/2007	52.32	-121.92	Canada	0.27	1050	4.1	146–193	Orthic Gray Luvisols	Lodgepole pine, Interior spruce	Dfc, Boreal cool summer	OM3	C0	0	LFH	NA	5.1	0.2	6.0	NA	12.0
SL-OM3-Ae-3	SL-OM3-Ae-3	SBS_BC_	British Columbia	SL	Forest Soil	Whole Community DNA	PCR	Amplicon	pyrotag library	454 GS FLX Titanium	European Nucleotide Archive	https://www.ebi.ac.uk/ena/data/search?query=PRJEB12501	ERS1434303	SAMEA4535124	ERX1790593	ERR1720641	16S rRNA	V6-V8	CGACACTATC	YAAAKGAATTGRCGG	ACGGGCGGTGTGTRC	7/15/2007	52.32	-121.92	Canada	0.555	1050	4.1	146–193	Orthic Gray Luvisols	Lodgepole pine, Interior spruce	Dfc, Boreal cool summer	OM3	C0	0	Ae	NA	2.1	0.1	5.2	NA	13.0
SL-OM3-AB-3	SL-OM3-AB-3	SBS_BC_	British Columbia	SL	Forest Soil	Whole Community DNA	PCR	Amplicon	pyrotag library	454 GS FLX Titanium	European Nucleotide Archive	https://www.ebi.ac.uk/ena/data/search?query=PRJEB12501	ERS1434304	SAMEA4535125	ERX1790594	ERR1720642	16S rRNA	V6-V8	CGAGACGCGC	YAAAKGAATTGRCGG	ACGGGCGGTGTGTRC	7/15/2007	52.32	-121.92	Canada	0.105	1050	4.1	146–193	Orthic Gray Luvisols	Lodgepole pine, Interior spruce	Dfc, Boreal cool summer	OM3	C0	0	AB	NA	0.9	0.0	5.7	NA	30.0
SL-OM3-Bt-3	SL-OM3-Bt-3	SBS_BC_	British Columbia	SL	Forest Soil	Whole Community DNA	PCR	Amplicon	pyrotag library	454 GS FLX Titanium	European Nucleotide Archive	https://www.ebi.ac.uk/ena/data/search?query=PRJEB12501	ERS1434305	SAMEA4535126	ERX1790595	ERR1720643	16S rRNA	V6-V8	CGTATGCGAC	YAAAKGAATTGRCGG	ACGGGCGGTGTGTRC	7/15/2007	52.32	-121.92	Canada	0.185	1050	4.1	146–193	Orthic Gray Luvisols	Lodgepole pine, Interior spruce	Dfc, Boreal cool summer	OM3	C0	0	Bt	NA	0.6	0.1	6.5	NA	23.3

**Table 3:** Sequencing and sample data for all SIP-hemicellulose and cellulose 16S and ITS amplicon libraries and shotgun metagenomes

**Sample**	**Sample ID**	**Ecozone**	**Region**	**Site**	**Location**	**Environmental Source**	**DNA Source**	**Library Type**	**Common Name**	**Instrument Model**	**Data Repository**	**Study Accession**	**Sample Accession**	**Secondary Sample Accession**	**Experiment Accession**	**Run Accession**	**Target Gene**	**Collection Date**	**Sample weight for DNA extraction (g)**	**Latitude**	**Longitude**	**Country**	**Sampling Depth (m)**	**Elevation (m)**	**Soil Classification**	**Treatment**	**Soil Horizon**	**Replicate**	**Water_content g/g**	**pH**	**Total C (g/kg)**	**Total N (g/kg)**	**Note**
A8-OM0C0-M1	A8056	BS_ON_	Ontario	A8	Fensom	Forest Soil	Whole Community DNA	Shotgun Metagenome	150-bp paired read library	Illumina HiSeq 2500	European Nucleotide Archive	PRJEB8420	ERS1420503	SAMEA4521324	ERX1770962	ERR1700674	WGS	7/4/2011	0.5	49.08	-89.38	Canada	0.2	450	Orthic Dystric Brunisol	OM0	A horizon	1	0.26	5.20	2.54	0.11	
A8-OM0C0-M2	A8058	BS_ON_	Ontario	A8	Fensom	Forest Soil	Whole Community DNA	Shotgun Metagenome	150-bp paired read library	Illumina HiSeq 2500	European Nucleotide Archive	https://www.ebi.ac.uk/ena/data/search?query=PRJEB8420	ERS1420504	SAMEA4521325	ERX1770963	ERR1700675	WGS	7/4/2011	0.5	49.08	-89.38	Canada	0.2	450	Orthic Dystric Brunisol	OM0	A horizon	2	0.28	4.90	3.30	0.12	
A8-OM0C0-M3	A8060	BS_ON_	Ontario	A8	Fensom	Forest Soil	Whole Community DNA	Shotgun Metagenome	150-bp paired read library	Illumina HiSeq 2500	European Nucleotide Archive	https://www.ebi.ac.uk/ena/data/search?query=PRJEB8420	ERS1420505	SAMEA4521326	ERX1770964	ERR1700676	WGS	7/4/2011	0.5	49.08	-89.38	Canada	0.2	450	Orthic Dystric Brunisol	OM0	A horizon	3	0.29	5.20	1.69	0.09	
A8-OM0C0-O1	A8055	BS_ON_	Ontario	A8	Fensom	Forest Soil	Whole Community DNA	Shotgun Metagenome	150-bp paired read library	Illumina HiSeq 2500	European Nucleotide Archive	https://www.ebi.ac.uk/ena/data/search?query=PRJEB8420	ERS1420506	SAMEA4521327	ERX1770965	ERR1700677	WGS	7/4/2011	0.5	49.08	-89.38	Canada	0	450	Orthic Dystric Brunisol	OM0	O Horizon	1	0.73	4.08	45.78	1.10	
A8-OM0C0-O2	A8057	BS_ON_	Ontario	A8	Fensom	Forest Soil	Whole Community DNA	Shotgun Metagenome	150-bp paired read library	Illumina HiSeq 2500	European Nucleotide Archive	https://www.ebi.ac.uk/ena/data/search?query=PRJEB8420	ERS1420507	SAMEA4521328	ERX1770966	ERR1700678	WGS	7/4/2011	0.5	49.08	-89.38	Canada	0	450	Orthic Dystric Brunisol	OM0	O Horizon	2	0.76	4.73	44.34	1.01	
A8-OM0C0-O3	A8059	BS_ON_	Ontario	A8	Fensom	Forest Soil	Whole Community DNA	Shotgun Metagenome	150-bp paired read library	Illumina HiSeq 2500	European Nucleotide Archive	https://www.ebi.ac.uk/ena/data/search?query=PRJEB8420	ERS1420508	SAMEA4521329	ERX1770967	ERR1700679	WGS	7/4/2011	0.5	49.08	-89.38	Canada	0	450	Orthic Dystric Brunisol	OM0	O Horizon	3	0.73	4.15	45.67	1.05	
A8-OM1C0-M1	A8032	BS_ON_	Ontario	A8	Fensom	Forest Soil	Whole Community DNA	Shotgun Metagenome	150-bp paired read library	Illumina HiSeq 2500	European Nucleotide Archive	https://www.ebi.ac.uk/ena/data/search?query=PRJEB8420	ERS1420509	SAMEA4521330	ERX1770968	ERR1700680	WGS	7/4/2011	0.5	49.08	-89.38	Canada	0.2	450	Orthic Dystric Brunisol	OM1	A horizon	1	0.23	4.99	3.21	0.13	
A8-OM1C0-M2	A8046	BS_ON_	Ontario	A8	Fensom	Forest Soil	Whole Community DNA	Shotgun Metagenome	150-bp paired read library	Illumina HiSeq 2500	European Nucleotide Archive	https://www.ebi.ac.uk/ena/data/search?query=PRJEB8420	ERS1420510	SAMEA4521331	ERX1770969	ERR1700681	WGS	7/4/2011	0.5	49.08	-89.38	Canada	0.2	450	Orthic Dystric Brunisol	OM1	A horizon	2	0.24	5.15	1.98	0.09	
A8-OM1C0-M3	A8054	BS_ON_	Ontario	A8	Fensom	Forest Soil	Whole Community DNA	Shotgun Metagenome	150-bp paired read library	Illumina HiSeq 2500	European Nucleotide Archive	https://www.ebi.ac.uk/ena/data/search?query=PRJEB8420	ERS1420511	SAMEA4521332	ERX1770970	ERR1700682	WGS	7/4/2011	0.5	49.08	-89.38	Canada	0.2	450	Orthic Dystric Brunisol	OM1	A horizon	3	0.23	5.03	4.66	0.19	
A8-OM1C0-O1	A8031	BS_ON_	Ontario	A8	Fensom	Forest Soil	Whole Community DNA	Shotgun Metagenome	150-bp paired read library	Illumina HiSeq 2500	European Nucleotide Archive	https://www.ebi.ac.uk/ena/data/search?query=PRJEB8420	ERS1420512	SAMEA4521333	ERX1770971	ERR1700683	WGS	7/4/2011	0.5	49.08	-89.38	Canada	0	450	Orthic Dystric Brunisol	OM1	O Horizon	1	0.54	4.37	43.17	1.14	
A8-OM1C0-O2	A8045	BS_ON_	Ontario	A8	Fensom	Forest Soil	Whole Community DNA	Shotgun Metagenome	150-bp paired read library	Illumina HiSeq 2500	European Nucleotide Archive	https://www.ebi.ac.uk/ena/data/search?query=PRJEB8420	ERS1420513	SAMEA4521334	ERX1770972	ERR1700684	WGS	7/4/2011	0.5	49.08	-89.38	Canada	0	450	Orthic Dystric Brunisol	OM1	O Horizon	2	0.36	4.29	44.40	1.20	
A8-OM1C0-O3	A8053	BS_ON_	Ontario	A8	Fensom	Forest Soil	Whole Community DNA	Shotgun Metagenome	150-bp paired read library	Illumina HiSeq 2500	European Nucleotide Archive	https://www.ebi.ac.uk/ena/data/search?query=PRJEB8420	ERS1420514	SAMEA4521335	ERX1770973	ERR1700685	WGS	7/4/2011	0.5	49.08	-89.38	Canada	0	450	Orthic Dystric Brunisol	OM1	O Horizon	3	0.52	4.44	44.31	1.14	
A8-OM2C0-M1	A8038	BS_ON_	Ontario	A8	Fensom	Forest Soil	Whole Community DNA	Shotgun Metagenome	150-bp paired read library	Illumina HiSeq 2500	European Nucleotide Archive	https://www.ebi.ac.uk/ena/data/search?query=PRJEB8420	ERS1420515	SAMEA4521336	ERX1770974	ERR1700686	WGS	7/4/2011	0.5	49.08	-89.38	Canada	0.2	450	Orthic Dystric Brunisol	OM2	A horizon	1	0.19	5.00	1.98	0.09	
A8-OM2C0-M2	A8040	BS_ON_	Ontario	A8	Fensom	Forest Soil	Whole Community DNA	Shotgun Metagenome	150-bp paired read library	Illumina HiSeq 2500	European Nucleotide Archive	https://www.ebi.ac.uk/ena/data/search?query=PRJEB8420	ERS1420516	SAMEA4521337	ERX1770975	ERR1700687	WGS	7/4/2011	0.5	49.08	-89.38	Canada	0.2	450	Orthic Dystric Brunisol	OM2	A horizon	2	0.25	4.92	4.24	0.18	
A8-OM2C0-M3	A8048	BS_ON_	Ontario	A8	Fensom	Forest Soil	Whole Community DNA	Shotgun Metagenome	150-bp paired read library	Illumina HiSeq 2500	European Nucleotide Archive	https://www.ebi.ac.uk/ena/data/search?query=PRJEB8420	ERS1420517	SAMEA4521338	ERX1770976	ERR1700688	WGS	7/4/2011	0.5	49.08	-89.38	Canada	0.2	450	Orthic Dystric Brunisol	OM2	A horizon	3	0.21	5.02	1.84	0.08	
A8-OM2C0-O1	A8037	BS_ON_	Ontario	A8	Fensom	Forest Soil	Whole Community DNA	Shotgun Metagenome	150-bp paired read library	Illumina HiSeq 2500	European Nucleotide Archive	https://www.ebi.ac.uk/ena/data/search?query=PRJEB8420	ERS1420518	SAMEA4521339	ERX1770977	ERR1700689	WGS	7/4/2011	0.5	49.08	-89.38	Canada	0	450	Orthic Dystric Brunisol	OM2	O Horizon	1	0.46	4.38	39.33	1.05	
A8-OM2C0-O2	A8039	BS_ON_	Ontario	A8	Fensom	Forest Soil	Whole Community DNA	Shotgun Metagenome	150-bp paired read library	Illumina HiSeq 2500	European Nucleotide Archive	https://www.ebi.ac.uk/ena/data/search?query=PRJEB8420	ERS1420519	SAMEA4521340	ERX1770978	ERR1700690	WGS	7/4/2011	0.5	49.08	-89.38	Canada	0	450	Orthic Dystric Brunisol	OM2	O Horizon	2	0.7	4.78	40.33	1.01	
A8-OM2C0-O3	A8047	BS_ON_	Ontario	A8	Fensom	Forest Soil	Whole Community DNA	Shotgun Metagenome	150-bp paired read library	Illumina HiSeq 2500	European Nucleotide Archive	https://www.ebi.ac.uk/ena/data/search?query=PRJEB8420	ERS1420520	SAMEA4521341	ERX1770979	ERR1700691	WGS	7/4/2011	0.5	49.08	-89.38	Canada	0	450	Orthic Dystric Brunisol	OM2	O Horizon	3	0.53	4.27	43.54	1.17	
A8-OM3C0-M1	A8036	BS_ON_	Ontario	A8	Fensom	Forest Soil	Whole Community DNA	Shotgun Metagenome	150-bp paired read library	Illumina HiSeq 2500	European Nucleotide Archive	https://www.ebi.ac.uk/ena/data/search?query=PRJEB8420	ERS1420521	SAMEA4521342	ERX1770980	ERR1700692	WGS	7/4/2011	0.5	49.08	-89.38	Canada	0.2	450	Orthic Dystric Brunisol	OM3	A horizon	1	0.14	5.03	1.99	0.09	
A8-OM3C0-M2	A8042	BS_ON_	Ontario	A8	Fensom	Forest Soil	Whole Community DNA	Shotgun Metagenome	150-bp paired read library	Illumina HiSeq 2500	European Nucleotide Archive	https://www.ebi.ac.uk/ena/data/search?query=PRJEB8420	ERS1420522	SAMEA4521343	ERX1770981	ERR1700693	WGS	7/4/2011	0.5	49.08	-89.38	Canada	0.2	450	Orthic Dystric Brunisol	OM3	A horizon	2	0.18	5.27	3.62	0.13	
A8-OM3C0-M3	A8050	BS_ON_	Ontario	A8	Fensom	Forest Soil	Whole Community DNA	Shotgun Metagenome	150-bp paired read library	Illumina HiSeq 2500	European Nucleotide Archive	https://www.ebi.ac.uk/ena/data/search?query=PRJEB8420	ERS1420523	SAMEA4521344	ERX1770982	ERR1700694	WGS	7/4/2011	0.5	49.08	-89.38	Canada	0.2	450	Orthic Dystric Brunisol	OM3	A horizon	3	0.21	5.33	1.75	0.08	
BL-OM0C0-M1	BL044	PP_CA_	California	BL	Blodgett	Forest Soil	Whole Community DNA	Shotgun Metagenome	150-bp paired read library	Illumina HiSeq 2500	European Nucleotide Archive	https://www.ebi.ac.uk/ena/data/search?query=PRJEB8420	ERS1420524	SAMEA4521345	ERX1770983	ERR1700695	WGS	9/16/2011	0.5	38.88	-120.64	USA	0.2	1350	Mesic Ultic Haploxeralfs	OM0	A horizon	1	0.18	5.48	NA	NA	
BL-OM0C0-M2	BL046	PP_CA_	California	BL	Blodgett	Forest Soil	Whole Community DNA	Shotgun Metagenome	150-bp paired read library	Illumina HiSeq 2500	European Nucleotide Archive	https://www.ebi.ac.uk/ena/data/search?query=PRJEB8420	ERS1420525	SAMEA4521346	ERX1770984	ERR1700696	WGS	9/16/2011	0.5	38.88	-120.64	USA	0.2	1350	Mesic Ultic Haploxeralfs	OM0	A horizon	2	0.18	5.49	NA	NA	
BL-OM0C0-M31	BL048	PP_CA_	California	BL	Blodgett	Forest Soil	Whole Community DNA	Shotgun Metagenome	150-bp paired read library	Illumina HiSeq 2500	European Nucleotide Archive	https://www.ebi.ac.uk/ena/data/search?query=PRJEB8420	ERS1420526	SAMEA4521347	ERX1770985	ERR1700697	WGS	9/16/2011	0.5	38.88	-120.64	USA	0.2	1350	Mesic Ultic Haploxeralfs	OM0	A horizon	31	0.15	5.99	NA	NA	This sample was sequenced twice. This is the first sequence run
BL-OM0C0-M32	BL048	PP_CA_	California	BL	Blodgett	Forest Soil	Whole Community DNA	Shotgun Metagenome	150-bp paired read library	Illumina HiSeq 2500	European Nucleotide Archive	https://www.ebi.ac.uk/ena/data/search?query=PRJEB8420	ERS1420527	SAMEA4521348	ERX1770986	ERR1700698	WGS	9/16/2011	0.5	38.88	-120.64	USA	0.2	1350	Mesic Ultic Haploxeralfs	OM0	A horizon	32	0.15	5.99	NA	NA	This sample was sequenced twice. This is the second sequence run
BL-OM0C0-O1	BL043	PP_CA_	California	BL	Blodgett	Forest Soil	Whole Community DNA	Shotgun Metagenome	150-bp paired read library	Illumina HiSeq 2500	European Nucleotide Archive	https://www.ebi.ac.uk/ena/data/search?query=PRJEB8420	ERS1420528	SAMEA4521349	ERX1770987	ERR1700699	WGS	9/16/2011	0.5	38.88	-120.64	USA	0	1350	Mesic Ultic Haploxeralfs	OM0	O Horizon	1	0.27	4.51	NA	NA	
BL-OM0C0-O2	BL045	PP_CA_	California	BL	Blodgett	Forest Soil	Whole Community DNA	Shotgun Metagenome	150-bp paired read library	Illumina HiSeq 2500	European Nucleotide Archive	https://www.ebi.ac.uk/ena/data/search?query=PRJEB8420	ERS1420529	SAMEA4521350	ERX1770988	ERR1700700	WGS	9/16/2011	0.5	38.88	-120.64	USA	0	1350	Mesic Ultic Haploxeralfs	OM0	O Horizon	2	0.26	5.28	NA	NA	
BL-OM0C0-O3	BL047	PP_CA_	California	BL	Blodgett	Forest Soil	Whole Community DNA	Shotgun Metagenome	150-bp paired read library	Illumina HiSeq 2500	European Nucleotide Archive	https://www.ebi.ac.uk/ena/data/search?query=PRJEB8420	ERS1420530	SAMEA4521351	ERX1770989	ERR1700701	WGS	9/16/2011	0.5	38.88	-120.64	USA	0	1350	Mesic Ultic Haploxeralfs	OM0	O Horizon	3	0.3	4.9	NA	NA	
BL-OM1C0-M1	BL026	PP_CA_	California	BL	Blodgett	Forest Soil	Whole Community DNA	Shotgun Metagenome	150-bp paired read library	Illumina HiSeq 2500	European Nucleotide Archive	https://www.ebi.ac.uk/ena/data/search?query=PRJEB8420	ERS1420531	SAMEA4521352	ERX1770990	ERR1700702	WGS	9/16/2011	0.5	38.88	-120.64	USA	0.2	1350	Mesic Ultic Haploxeralfs	OM1	A horizon	1	0.22	5.74	5.486	0.277	
BL-OM1C0-M2	BL028	PP_CA_	California	BL	Blodgett	Forest Soil	Whole Community DNA	Shotgun Metagenome	150-bp paired read library	Illumina HiSeq 2500	European Nucleotide Archive	https://www.ebi.ac.uk/ena/data/search?query=PRJEB8420	ERS1420532	SAMEA4521353	ERX1770991	ERR1700703	WGS	9/16/2011	0.5	38.88	-120.64	USA	0.2	1350	Mesic Ultic Haploxeralfs	OM1	A horizon	2	0.21	5.87	5.486	0.277	
BL-OM1C0-M3	BL030	PP_CA_	California	BL	Blodgett	Forest Soil	Whole Community DNA	Shotgun Metagenome	150-bp paired read library	Illumina HiSeq 2500	European Nucleotide Archive	https://www.ebi.ac.uk/ena/data/search?query=PRJEB8420	ERS1420533	SAMEA4521354	ERX1770992	ERR1700704	WGS	9/16/2011	0.5	38.88	-120.64	USA	0.2	1350	Mesic Ultic Haploxeralfs	OM1	A horizon	3	0.24	5.89	5.486	0.277	
BL-OM1C0-O1	BL025	PP_CA_	California	BL	Blodgett	Forest Soil	Whole Community DNA	Shotgun Metagenome	150-bp paired read library	Illumina HiSeq 2500	European Nucleotide Archive	https://www.ebi.ac.uk/ena/data/search?query=PRJEB8420	ERS1420534	SAMEA4521355	ERX1770993	ERR1700705	WGS	9/16/2011	0.5	38.88	-120.64	USA	0	1350	Mesic Ultic Haploxeralfs	OM1	O Horizon	1	0.18	4.79	NA	NA	
BL-OM1C0-O2	BL027	PP_CA_	California	BL	Blodgett	Forest Soil	Whole Community DNA	Shotgun Metagenome	150-bp paired read library	Illumina HiSeq 2500	European Nucleotide Archive	https://www.ebi.ac.uk/ena/data/search?query=PRJEB8420	ERS1420535	SAMEA4521356	ERX1770994	ERR1700706	WGS	9/16/2011	0.5	38.88	-120.64	USA	0	1350	Mesic Ultic Haploxeralfs	OM1	O Horizon	2	0.17	4.9	NA	NA	
BL-OM1C0-O3	BL029	PP_CA_	California	BL	Blodgett	Forest Soil	Whole Community DNA	Shotgun Metagenome	150-bp paired read library	Illumina HiSeq 2500	European Nucleotide Archive	https://www.ebi.ac.uk/ena/data/search?query=PRJEB8420	ERS1420536	SAMEA4521357	ERX1770995	ERR1700707	WGS	9/16/2011	0.5	38.88	-120.64	USA	0	1350	Mesic Ultic Haploxeralfs	OM1	O Horizon	3	0.19	4.69	NA	NA	
BL-OM2C0-M1	BL032	PP_CA_	California	BL	Blodgett	Forest Soil	Whole Community DNA	Shotgun Metagenome	150-bp paired read library	Illumina HiSeq 2500	European Nucleotide Archive	https://www.ebi.ac.uk/ena/data/search?query=PRJEB8420	ERS1420537	SAMEA4521358	ERX1770996	ERR1700708	WGS	9/16/2011	0.5	38.88	-120.64	USA	0.2	1350	Mesic Ultic Haploxeralfs	OM2	A horizon	1	0.21	5.78	5.238	0.25	
BL-OM2C0-M21	BL034	PP_CA_	California	BL	Blodgett	Forest Soil	Whole Community DNA	Shotgun Metagenome	150-bp paired read library	Illumina HiSeq 2500	European Nucleotide Archive	https://www.ebi.ac.uk/ena/data/search?query=PRJEB8420	ERS1420538	SAMEA4521359	ERX1770997	ERR1700709	WGS	9/15/2011	0.5	38.88	-120.64	USA	0.2	1350	Mesic Ultic Haploxeralfs	OM2	A horizon	21	0.21	5.3	5.238	0.25	Total C and total N were calculated per plot and not per sample, This sample was sequenced twice. This is the first sequence run
BL-OM2C0-M22	BL034	PP_CA_	California	BL	Blodgett	Forest Soil	Whole Community DNA	Shotgun Metagenome	150-bp paired read library	Illumina HiSeq 2500	European Nucleotide Archive	https://www.ebi.ac.uk/ena/data/search?query=PRJEB8420	ERS1420539	SAMEA4521360	ERX1770998	ERR1700710	WGS	9/15/2011	0.5	38.88	-120.64	USA	0.2	1350	Mesic Ultic Haploxeralfs	OM2	A horizon	22	0.21	5.3	5.238	0.25	Total C and total N were calculated per plot and not per sample, This sample was sequenced twice. This is the second sequence run
BL-OM2C0-M3	BL036	PP_CA_	California	BL	Blodgett	Forest Soil	Whole Community DNA	Shotgun Metagenome	150-bp paired read library	Illumina HiSeq 2500	European Nucleotide Archive	https://www.ebi.ac.uk/ena/data/search?query=PRJEB8420	ERS1420540	SAMEA4521361	ERX1770999	ERR1700711	WGS	9/15/2011	0.5	38.88	-120.64	USA	0.2	1350	Mesic Ultic Haploxeralfs	OM2	A horizon	3	0.22	5.72	5.238	0.25	Total C and total N were calculated per plot and not per sample
BL-OM2C0-O1	BL031	PP_CA_	California	BL	Blodgett	Forest Soil	Whole Community DNA	Shotgun Metagenome	150-bp paired read library	Illumina HiSeq 2500	European Nucleotide Archive	https://www.ebi.ac.uk/ena/data/search?query=PRJEB8420	ERS1420541	SAMEA4521362	ERX1771000	ERR1700712	WGS	9/16/2011	0.5	38.88	-120.64	USA	0	1350	Mesic Ultic Haploxeralfs	OM2	O Horizon	1	0.29	5.73	NA	NA	
BL-OM2C0-O2	BL033	PP_CA_	California	BL	Blodgett	Forest Soil	Whole Community DNA	Shotgun Metagenome	150-bp paired read library	Illumina HiSeq 2500	European Nucleotide Archive	https://www.ebi.ac.uk/ena/data/search?query=PRJEB8420	ERS1420542	SAMEA4521363	ERX1771001	ERR1700713	WGS	9/16/2011	0.5	38.88	-120.64	USA	0	1350	Mesic Ultic Haploxeralfs	OM2	O Horizon	2	0.22	5.24	NA	NA	
BL-OM2C0-O3	BL035	PP_CA_	California	BL	Blodgett	Forest Soil	Whole Community DNA	Shotgun Metagenome	150-bp paired read library	Illumina HiSeq 2500	European Nucleotide Archive	https://www.ebi.ac.uk/ena/data/search?query=PRJEB8420	ERS1420543	SAMEA4521364	ERX1771002	ERR1700714	WGS	9/15/2011	0.5	38.88	-120.64	USA	0	1350	Mesic Ultic Haploxeralfs	OM2	O Horizon	3	0.16	5.09	NA	NA	
BL-OM3C0-M1	BL038	PP_CA_	California	BL	Blodgett	Forest Soil	Whole Community DNA	Shotgun Metagenome	150-bp paired read library	Illumina HiSeq 2500	European Nucleotide Archive	https://www.ebi.ac.uk/ena/data/search?query=PRJEB8420	ERS1420544	SAMEA4521365	ERX1771003	ERR1700715	WGS	9/15/2011	0.5	38.88	-120.64	USA	0.2	1350	Mesic Ultic Haploxeralfs	OM3	A horizon	1	0.22	5.42	5.55	0.291	
BL-OM3C0-M2	BL040	PP_CA_	California	BL	Blodgett	Forest Soil	Whole Community DNA	Shotgun Metagenome	150-bp paired read library	Illumina HiSeq 2500	European Nucleotide Archive	https://www.ebi.ac.uk/ena/data/search?query=PRJEB8420	ERS1420545	SAMEA4521366	ERX1771004	ERR1700716	WGS	9/16/2011	0.5	38.88	-120.64	USA	0.2	1350	Mesic Ultic Haploxeralfs	OM3	A horizon	2	0.22	5.86	5.55	0.291	
BL-OM3C0-M3	BL042	PP_CA_	California	BL	Blodgett	Forest Soil	Whole Community DNA	Shotgun Metagenome	150-bp paired read library	Illumina HiSeq 2500	European Nucleotide Archive	https://www.ebi.ac.uk/ena/data/search?query=PRJEB8420	ERS1420546	SAMEA4521367	ERX1771005	ERR1700717	WGS	9/16/2011	0.5	38.88	-120.64	USA	0.2	1350	Mesic Ultic Haploxeralfs	OM3	A horizon	3	0.21	5.39	5.55	0.291	
JW-OM0C0-M1	JW014	JP_ON_	Ontario	JW	Wells	Forest Soil	Whole Community DNA	Shotgun Metagenome	150-bp paired read library	Illumina HiSeq 2500	European Nucleotide Archive	https://www.ebi.ac.uk/ena/data/search?query=PRJEB8420	ERS1420547	SAMEA4521368	ERX1771006	ERR1700718	WGS	7/7/2011	0.5	46.42	-83.37	Canada	0.2	228	Orthic Humo-Ferric Podzol	OM0	A horizon	1	0.16	5.48	1.52	0.116	
JW-OM0C0-M2	JW020	JP_ON_	Ontario	JW	Wells	Forest Soil	Whole Community DNA	Shotgun Metagenome	150-bp paired read library	Illumina HiSeq 2500	European Nucleotide Archive	https://www.ebi.ac.uk/ena/data/search?query=PRJEB8420	ERS1420548	SAMEA4521369	ERX1771007	ERR1700719	WGS	7/7/2011	0.5	46.42	-83.37	Canada	0.2	228	Orthic Humo-Ferric Podzol	OM0	A horizon	2	0.12	5.42	2.556	0.168	
JW-OM0C0-M3	JW026	JP_ON_	Ontario	JW	Wells	Forest Soil	Whole Community DNA	Shotgun Metagenome	150-bp paired read library	Illumina HiSeq 2500	European Nucleotide Archive	https://www.ebi.ac.uk/ena/data/search?query=PRJEB8420	ERS1420549	SAMEA4521370	ERX1771008	ERR1700720	WGS	7/7/2011	0.5	46.42	-83.37	Canada	0.2	228	Orthic Humo-Ferric Podzol	OM0	A horizon	3	0.14	5.34	3.13	0.221	
JW-OM0C0-O1	JW013	JP_ON_	Ontario	JW	Wells	Forest Soil	Whole Community DNA	Shotgun Metagenome	150-bp paired read library	Illumina HiSeq 2500	European Nucleotide Archive	https://www.ebi.ac.uk/ena/data/search?query=PRJEB8420	ERS1420550	SAMEA4521371	ERX1771009	ERR1700721	WGS	7/7/2011	0.5	46.42	-83.37	Canada	0	228	Orthic Humo-Ferric Podzol	OM0	O Horizon	1	0.31	4.1	38.128	1.066	
JW-OM0C0-O2	JW019	JP_ON_	Ontario	JW	Wells	Forest Soil	Whole Community DNA	Shotgun Metagenome	150-bp paired read library	Illumina HiSeq 2500	European Nucleotide Archive	https://www.ebi.ac.uk/ena/data/search?query=PRJEB8420	ERS1420551	SAMEA4521372	ERX1771010	ERR1700722	WGS	7/7/2011	0.5	46.42	-83.37	Canada	0	228	Orthic Humo-Ferric Podzol	OM0	O Horizon	2	0.31	4.2	36.85	1.199	
JW-OM0C0-O3	JW025	JP_ON_	Ontario	JW	Wells	Forest Soil	Whole Community DNA	Shotgun Metagenome	150-bp paired read library	Illumina HiSeq 2500	European Nucleotide Archive	https://www.ebi.ac.uk/ena/data/search?query=PRJEB8420	ERS1420552	SAMEA4521373	ERX1771011	ERR1700723	WGS	7/7/2011	0.5	46.42	-83.37	Canada	0	228	Orthic Humo-Ferric Podzol	OM0	O Horizon	3	0.4	4.08	34.549	1.139	
JW-OM1C0-M1	JW008	JP_ON_	Ontario	JW	Wells	Forest Soil	Whole Community DNA	Shotgun Metagenome	150-bp paired read library	Illumina HiSeq 2500	European Nucleotide Archive	https://www.ebi.ac.uk/ena/data/search?query=PRJEB8420	ERS1420553	SAMEA4521374	ERX1771012	ERR1700724	WGS	7/7/2011	0.5	46.42	-83.37	Canada	0.2	228	Orthic Humo-Ferric Podzol	OM1	A horizon	1	0.1	5.24	2.711	0.171	
JW-OM1C0-M2	JW024	JP_ON_	Ontario	JW	Wells	Forest Soil	Whole Community DNA	Shotgun Metagenome	150-bp paired read library	Illumina HiSeq 2500	European Nucleotide Archive	https://www.ebi.ac.uk/ena/data/search?query=PRJEB8420	ERS1420554	SAMEA4521375	ERX1771013	ERR1700725	WGS	7/7/2011	0.5	46.42	-83.37	Canada	0.2	228	Orthic Humo-Ferric Podzol	OM1	A horizon	2	0.12	5.34	3.13	0.221	
JW-OM1C0-M3	JW030	JP_ON_	Ontario	JW	Wells	Forest Soil	Whole Community DNA	Shotgun Metagenome	150-bp paired read library	Illumina HiSeq 2500	European Nucleotide Archive	https://www.ebi.ac.uk/ena/data/search?query=PRJEB8420	ERS1420555	SAMEA4521376	ERX1771014	ERR1700726	WGS	7/7/2011	0.5	46.42	-83.37	Canada	0.2	228	Orthic Humo-Ferric Podzol	OM1	A horizon	3	0.14	5.3	3.26	0.293	
JW-OM1C0-O1	JW007	JP_ON_	Ontario	JW	Wells	Forest Soil	Whole Community DNA	Shotgun Metagenome	150-bp paired read library	Illumina HiSeq 2500	European Nucleotide Archive	https://www.ebi.ac.uk/ena/data/search?query=PRJEB8420	ERS1420556	SAMEA4521377	ERX1771015	ERR1700727	WGS	7/7/2011	0.5	46.42	-83.37	Canada	0	228	Orthic Humo-Ferric Podzol	OM1	O Horizon	1	0.4	4.18	36.414	1.114	
JW-OM1C0-O2	JW023	JP_ON_	Ontario	JW	Wells	Forest Soil	Whole Community DNA	Shotgun Metagenome	150-bp paired read library	Illumina HiSeq 2500	European Nucleotide Archive	https://www.ebi.ac.uk/ena/data/search?query=PRJEB8420	ERS1420557	SAMEA4521378	ERX1771016	ERR1700728	WGS	7/7/2011	0.5	46.42	-83.37	Canada	0	228	Orthic Humo-Ferric Podzol	OM1	O Horizon	2	0.47	4.08	34.549	1.139	
JW-OM1C0-O3	JW029	JP_ON_	Ontario	JW	Wells	Forest Soil	Whole Community DNA	Shotgun Metagenome	150-bp paired read library	Illumina HiSeq 2500	European Nucleotide Archive	https://www.ebi.ac.uk/ena/data/search?query=PRJEB8420	ERS1420558	SAMEA4521379	ERX1771017	ERR1700729	WGS	7/7/2011	0.5	46.42	-83.37	Canada	0	228	Orthic Humo-Ferric Podzol	OM1	O Horizon	3	0.57	4.41	33.831	1.027	
JW-OM2C0-M1	JW018	JP_ON_	Ontario	JW	Wells	Forest Soil	Whole Community DNA	Shotgun Metagenome	150-bp paired read library	Illumina HiSeq 2500	European Nucleotide Archive	https://www.ebi.ac.uk/ena/data/search?query=PRJEB8420	ERS1420559	SAMEA4521380	ERX1771018	ERR1700730	WGS	7/7/2011	0.5	46.42	-83.37	Canada	0.2	228	Orthic Humo-Ferric Podzol	OM2	A horizon	1	0.15	5.42	2.556	0.168	
JW-OM2C0-M2	JW034	JP_ON_	Ontario	JW	Wells	Forest Soil	Whole Community DNA	Shotgun Metagenome	150-bp paired read library	Illumina HiSeq 2500	European Nucleotide Archive	https://www.ebi.ac.uk/ena/data/search?query=PRJEB8420	ERS1420560	SAMEA4521381	ERX1771019	ERR1700731	WGS	7/7/2011	0.5	46.42	-83.37	Canada	0.2	228	Orthic Humo-Ferric Podzol	OM2	A horizon	2	0.16	5.13	3.029	0.354	
JW-OM2C0-M3	JW038	JP_ON_	Ontario	JW	Wells	Forest Soil	Whole Community DNA	Shotgun Metagenome	150-bp paired read library	Illumina HiSeq 2500	European Nucleotide Archive	https://www.ebi.ac.uk/ena/data/search?query=PRJEB8420	ERS1420561	SAMEA4521382	ERX1771020	ERR1700732	WGS	7/7/2011	0.5	46.42	-83.37	Canada	0.2	228	Orthic Humo-Ferric Podzol	OM2	A horizon	3	0.13	5.35	2.657	0.476	
JW-OM2C0-O1	JW017	JP_ON_	Ontario	JW	Wells	Forest Soil	Whole Community DNA	Shotgun Metagenome	150-bp paired read library	Illumina HiSeq 2500	European Nucleotide Archive	https://www.ebi.ac.uk/ena/data/search?query=PRJEB8420	ERS1420562	SAMEA4521383	ERX1771021	ERR1700733	WGS	7/7/2011	0.5	46.42	-83.37	Canada	0	228	Orthic Humo-Ferric Podzol	OM2	O Horizon	1	0.47	4.2	36.85	1.199	
JW-OM2C0-O2	JW033	JP_ON_	Ontario	JW	Wells	Forest Soil	Whole Community DNA	Shotgun Metagenome	150-bp paired read library	Illumina HiSeq 2500	European Nucleotide Archive	https://www.ebi.ac.uk/ena/data/search?query=PRJEB8420	ERS1420563	SAMEA4521384	ERX1771022	ERR1700734	WGS	7/7/2011	0.5	46.42	-83.37	Canada	0	228	Orthic Humo-Ferric Podzol	OM2	O Horizon	2	0.6	4	37.617	1.116	
JW-OM2C0-O3	JW037	JP_ON_	Ontario	JW	Wells	Forest Soil	Whole Community DNA	Shotgun Metagenome	150-bp paired read library	Illumina HiSeq 2500	European Nucleotide Archive	https://www.ebi.ac.uk/ena/data/search?query=PRJEB8420	ERS1420564	SAMEA4521385	ERX1771023	ERR1700735	WGS	7/7/2011	0.5	46.42	-83.37	Canada	0	228	Orthic Humo-Ferric Podzol	OM2	O Horizon	3	0.48	4.27	35.24	1.093	
JW-OM3C0-M1	JW004	JP_ON_	Ontario	JW	Wells	Forest Soil	Whole Community DNA	Shotgun Metagenome	150-bp paired read library	Illumina HiSeq 2500	European Nucleotide Archive	https://www.ebi.ac.uk/ena/data/search?query=PRJEB8420	ERS1420565	SAMEA4521386	ERX1771024	ERR1700736	WGS	7/7/2011	0.5	46.42	-83.37	Canada	0.2	228	Orthic Humo-Ferric Podzol	OM3	A horizon	1	0.11	5.39	2.151	0.122	
JW-OM3C0-M2	JW012	JP_ON_	Ontario	JW	Wells	Forest Soil	Whole Community DNA	Shotgun Metagenome	150-bp paired read library	Illumina HiSeq 2500	European Nucleotide Archive	https://www.ebi.ac.uk/ena/data/search?query=PRJEB8420	ERS1420566	SAMEA4521387	ERX1771025	ERR1700737	WGS	7/7/2011	0.5	46.42	-83.37	Canada	0.2	228	Orthic Humo-Ferric Podzol	OM3	A horizon	2	0.13	5.48	1.52	0.116	
JW-OM3C0-M3	JW042	JP_ON_	Ontario	JW	Wells	Forest Soil	Whole Community DNA	Shotgun Metagenome	150-bp paired read library	Illumina HiSeq 2500	European Nucleotide Archive	https://www.ebi.ac.uk/ena/data/search?query=PRJEB8420	ERS1420567	SAMEA4521388	ERX1771026	ERR1700738	WGS	7/7/2011	0.5	46.42	-83.37	Canada	0.2	228	Orthic Humo-Ferric Podzol	OM3	A horizon	3	0.14	5.39	1.284	0.558	
OC-OM0C0-M1	OL364	IDF_BC_	British Columbia	OC	O’Connor Lake	Forest Soil	Whole Community DNA	Shotgun Metagenome	75-bp paired read library	Illumina HiSeq 2000	European Nucleotide Archive	https://www.ebi.ac.uk/ena/data/search?query=PRJEB8420	ERS656878	SAMEA3235149	ERX697631	ERR753910	WGS	6/18/2011	0.5	50.88	-120.35	Canada	0.2	1075	Brunisolic Gray Luvisol	OM0	A horizon	1	0.12	4.98	2.33	0.12	
OC-OM0C0-M2	OL365	IDF_BC_	British Columbia	OC	O’Connor Lake	Forest Soil	Whole Community DNA	Shotgun Metagenome	75-bp paired read library	Illumina HiSeq 2000	European Nucleotide Archive	https://www.ebi.ac.uk/ena/data/search?query=PRJEB8420	ERS656879	SAMEA3235150	ERX697632	ERR753911	WGS	6/18/2011	0.5	50.88	-120.35	Canada	0.2	1075	Brunisolic Gray Luvisol	OM0	A horizon	2	0.13	5.02	1.23	0.08	
OC-OM0C0-M3	OL366	IDF_BC_	British Columbia	OC	O’Connor Lake	Forest Soil	Whole Community DNA	Shotgun Metagenome	75-bp paired read library	Illumina HiSeq 2000	European Nucleotide Archive	https://www.ebi.ac.uk/ena/data/search?query=PRJEB8420	ERS656880	SAMEA3235151	ERX697633	ERR753912	WGS	6/18/2011	0.5	50.88	-120.35	Canada	0.2	1075	Brunisolic Gray Luvisol	OM0	A horizon	3	0.19	5.01	1.96	0.10	
OC-OM0C0-O1	OL361	IDF_BC_	British Columbia	OC	O’Connor Lake	Forest Soil	Whole Community DNA	Shotgun Metagenome	75-bp paired read library	Illumina HiSeq 2000	European Nucleotide Archive	https://www.ebi.ac.uk/ena/data/search?query=PRJEB8420	ERS656881	SAMEA3235152	ERX697634	ERR753913	WGS	6/18/2011	0.5	50.88	-120.35	Canada	0	1075	Brunisolic Gray Luvisol	OM0	O Horizon	1	0.4	5.04	45.47	1.37	
OC-OM0C0-O2	OL362	IDF_BC_	British Columbia	OC	O’Connor Lake	Forest Soil	Whole Community DNA	Shotgun Metagenome	75-bp paired read library	Illumina HiSeq 2000	European Nucleotide Archive	https://www.ebi.ac.uk/ena/data/search?query=PRJEB8420	ERS656882	SAMEA3235153	ERX697635	ERR753914	WGS	6/18/2011	0.5	50.88	-120.35	Canada	0	1075	Brunisolic Gray Luvisol	OM0	O Horizon	2	0.46	5.34	45.11	1.47	
OC-OM0C0-O3	OL363	IDF_BC_	British Columbia	OC	O’Connor Lake	Forest Soil	Whole Community DNA	Shotgun Metagenome	75-bp paired read library	Illumina HiSeq 2000	European Nucleotide Archive	https://www.ebi.ac.uk/ena/data/search?query=PRJEB8420	ERS656883	SAMEA3235154	ERX697636	ERR753915	WGS	6/18/2011	0.5	50.88	-120.35	Canada	0	1075	Brunisolic Gray Luvisol	OM0	O Horizon	3	0.54	4.87	48.55	1.34	
OC-OM1C0-M1	OL370	IDF_BC_	British Columbia	OC	O’Connor Lake	Forest Soil	Whole Community DNA	Shotgun Metagenome	75-bp paired read library	Illumina HiSeq 2000	European Nucleotide Archive	https://www.ebi.ac.uk/ena/data/search?query=PRJEB8420	ERS656884	SAMEA3235155	ERX697637	ERR753916	WGS	6/18/2011	0.5	50.88	-120.35	Canada	0.2	1075	Brunisolic Gray Luvisol	OM1	A horizon	1	0.2	5.16	2.51	0.14	
OC-OM1C0-M2	OL371	IDF_BC_	British Columbia	OC	O’Connor Lake	Forest Soil	Whole Community DNA	Shotgun Metagenome	75-bp paired read library	Illumina HiSeq 2000	European Nucleotide Archive	https://www.ebi.ac.uk/ena/data/search?query=PRJEB8420	ERS656885	SAMEA3235156	ERX697638	ERR753917	WGS	6/18/2011	0.5	50.88	-120.35	Canada	0.2	1075	Brunisolic Gray Luvisol	OM1	A horizon	2	0.2	5.21	1.78	0.11	
OC-OM1C0-M3	OL372	IDF_BC_	British Columbia	OC	O’Connor Lake	Forest Soil	Whole Community DNA	Shotgun Metagenome	75-bp paired read library	Illumina HiSeq 2000	European Nucleotide Archive	https://www.ebi.ac.uk/ena/data/search?query=PRJEB8420	ERS656886	SAMEA3235157	ERX697639	ERR753918	WGS	6/18/2011	0.5	50.88	-120.35	Canada	0.2	1075	Brunisolic Gray Luvisol	OM1	A horizon	3	0.22	5.01	1.79	0.12	
OC-OM1C0-O1	OL367	IDF_BC_	British Columbia	OC	O’Connor Lake	Forest Soil	Whole Community DNA	Shotgun Metagenome	75-bp paired read library	Illumina HiSeq 2000	European Nucleotide Archive	https://www.ebi.ac.uk/ena/data/search?query=PRJEB8420	ERS656887	SAMEA3235158	ERX697640	ERR753919	WGS	6/18/2011	0.5	50.88	-120.35	Canada	0	1075	Brunisolic Gray Luvisol	OM1	O Horizon	1	0.57	5.36	34.99	1.54	
OC-OM1C0-O2	OL368	IDF_BC_	British Columbia	OC	O’Connor Lake	Forest Soil	Whole Community DNA	Shotgun Metagenome	75-bp paired read library	Illumina HiSeq 2000	European Nucleotide Archive	https://www.ebi.ac.uk/ena/data/search?query=PRJEB8420	ERS656888	SAMEA3235159	ERX697641	ERR753920	WGS	6/18/2011	0.5	50.88	-120.35	Canada	0	1075	Brunisolic Gray Luvisol	OM1	O Horizon	2	0.57	4.8	37.14	1.36	
OC-OM1C0-O3	OL369	IDF_BC_	British Columbia	OC	O’Connor Lake	Forest Soil	Whole Community DNA	Shotgun Metagenome	75-bp paired read library	Illumina HiSeq 2000	European Nucleotide Archive	https://www.ebi.ac.uk/ena/data/search?query=PRJEB8420	ERS656889	SAMEA3235160	ERX697642	ERR753921	WGS	6/18/2011	0.5	50.88	-120.35	Canada	0	1075	Brunisolic Gray Luvisol	OM1	O Horizon	3	0.61	5.34	36.59	1.39	
OC-OM2C0-M1	OL376	IDF_BC_	British Columbia	OC	O’Connor Lake	Forest Soil	Whole Community DNA	Shotgun Metagenome	75-bp paired read library	Illumina HiSeq 2000	European Nucleotide Archive	https://www.ebi.ac.uk/ena/data/search?query=PRJEB8420	ERS656890	SAMEA3235161	ERX697643	ERR753922	WGS	6/18/2011	0.5	50.88	-120.35	Canada	0.2	1075	Brunisolic Gray Luvisol	OM2	A horizon	1	0.2	5.02	1.73	0.10	
OC-OM2C0-M2	OL377	IDF_BC_	British Columbia	OC	O’Connor Lake	Forest Soil	Whole Community DNA	Shotgun Metagenome	75-bp paired read library	Illumina HiSeq 2000	European Nucleotide Archive	https://www.ebi.ac.uk/ena/data/search?query=PRJEB8420	ERS656891	SAMEA3235162	ERX697644	ERR753923	WGS	6/18/2011	0.5	50.88	-120.35	Canada	0.2	1075	Brunisolic Gray Luvisol	OM2	A horizon	2	0.21	5.34	1.82	0.11	
OC-OM2C0-M3	OL378	IDF_BC_	British Columbia	OC	O’Connor Lake	Forest Soil	Whole Community DNA	Shotgun Metagenome	75-bp paired read library	Illumina HiSeq 2000	European Nucleotide Archive	https://www.ebi.ac.uk/ena/data/search?query=PRJEB8420	ERS656892	SAMEA3235163	ERX697645	ERR753924	WGS	6/18/2011	0.5	50.88	-120.35	Canada	0.2	1075	Brunisolic Gray Luvisol	OM2	A horizon	3	0.21	5.01	2.00	0.09	
OC-OM2C0-O1	OL373	IDF_BC_	British Columbia	OC	O’Connor Lake	Forest Soil	Whole Community DNA	Shotgun Metagenome	75-bp paired read library	Illumina HiSeq 2000	European Nucleotide Archive	https://www.ebi.ac.uk/ena/data/search?query=PRJEB8420	ERS656893	SAMEA3235164	ERX697646	ERR753925	WGS	6/18/2011	0.5	50.88	-120.35	Canada	0	1075	Brunisolic Gray Luvisol	OM2	O Horizon	1	0.6	5.07	40.12	1.35	
OC-OM2C0-O2	OL374	IDF_BC_	British Columbia	OC	O’Connor Lake	Forest Soil	Whole Community DNA	Shotgun Metagenome	75-bp paired read library	Illumina HiSeq 2000	European Nucleotide Archive	https://www.ebi.ac.uk/ena/data/search?query=PRJEB8420	ERS656894	SAMEA3235165	ERX697647	ERR753926	WGS	6/18/2011	0.5	50.88	-120.35	Canada	0	1075	Brunisolic Gray Luvisol	OM2	O Horizon	2	0.59	5.6	35.37	1.56	
OC-OM2C0-O3	OL375	IDF_BC_	British Columbia	OC	O’Connor Lake	Forest Soil	Whole Community DNA	Shotgun Metagenome	75-bp paired read library	Illumina HiSeq 2000	European Nucleotide Archive	https://www.ebi.ac.uk/ena/data/search?query=PRJEB8420	ERS656895	SAMEA3235166	ERX697648	ERR753927	WGS	6/18/2011	0.5	50.88	-120.35	Canada	0	1075	Brunisolic Gray Luvisol	OM2	O Horizon	3	0.6	5.46	25.73	1.10	
OC-OM3C0-M1	OL379	IDF_BC_	British Columbia	OC	O’Connor Lake	Forest Soil	Whole Community DNA	Shotgun Metagenome	75-bp paired read library	Illumina HiSeq 2000	European Nucleotide Archive	https://www.ebi.ac.uk/ena/data/search?query=PRJEB8420	ERS656896	SAMEA3235167	ERX697649	ERR753928	WGS	6/18/2011	0.5	50.88	-120.35	Canada	0.2	1075	Brunisolic Gray Luvisol	OM3	A horizon	1	0.23	5.16	1.78	0.10	
OC-OM3C0-M2	OL380	IDF_BC_	British Columbia	OC	O’Connor Lake	Forest Soil	Whole Community DNA	Shotgun Metagenome	75-bp paired read library	Illumina HiSeq 2000	European Nucleotide Archive	https://www.ebi.ac.uk/ena/data/search?query=PRJEB8420	ERS656897	SAMEA3235168	ERX697650	ERR753929	WGS	6/18/2011	0.5	50.88	-120.35	Canada	0.2	1075	Brunisolic Gray Luvisol	OM3	A horizon	2	0.21	5.21	1.75	0.11	
OC-OM3C0-M3	OL381	IDF_BC_	British Columbia	OC	O’Connor Lake	Forest Soil	Whole Community DNA	Shotgun Metagenome	75-bp paired read library	Illumina HiSeq 2000	European Nucleotide Archive	https://www.ebi.ac.uk/ena/data/search?query=PRJEB8420	ERS656898	SAMEA3235169	ERX697651	ERR753930	WGS	6/18/2011	0.5	50.88	-120.35	Canada	0.2	1075	Brunisolic Gray Luvisol	OM3	A horizon	3	0.16	5.27	2.83	0.11	
TXA-OM0C0-M1	TXA037	LP_TX_	Texas	TXA	Kurth	Forest Soil	Whole Community DNA	Shotgun Metagenome	150-bp paired read library	Illumina HiSeq 2500	European Nucleotide Archive	https://www.ebi.ac.uk/ena/data/search?query=PRJEB8420	ERS1420568	SAMEA4521389	ERX1771027	ERR1700739	WGS	3/12/2012	0.5	31.11	-95.15	USA	0.2	88	Aquic Glossudalfs	OM0	A horizon	1	0.1	4.47	NA	NA	
TXA-OM0C0-M2	TXA038	LP_TX_	Texas	TXA	Kurth	Forest Soil	Whole Community DNA	Shotgun Metagenome	150-bp paired read library	Illumina HiSeq 2500	European Nucleotide Archive	https://www.ebi.ac.uk/ena/data/search?query=PRJEB8420	ERS1420569	SAMEA4521390	ERX1771028	ERR1700740	WGS	3/12/2012	0.5	31.11	-95.15	USA	0.2	88	Aquic Glossudalfs	OM0	A horizon	2	0.11	4.83	NA	NA	
TXA-OM0C0-M3	TXA039	LP_TX_	Texas	TXA	Kurth	Forest Soil	Whole Community DNA	Shotgun Metagenome	150-bp paired read library	Illumina HiSeq 2500	European Nucleotide Archive	https://www.ebi.ac.uk/ena/data/search?query=PRJEB8420	ERS1420570	SAMEA4521391	ERX1771029	ERR1700741	WGS	3/12/2012	0.5	31.11	-95.15	USA	0.2	88	Aquic Glossudalfs	OM0	A horizon	3	0.11	4.39	NA	NA	
TXA-OM0C0-O1	TXA040	LP_TX_	Texas	TXA	Kurth	Forest Soil	Whole Community DNA	Shotgun Metagenome	150-bp paired read library	Illumina HiSeq 2500	European Nucleotide Archive	https://www.ebi.ac.uk/ena/data/search?query=PRJEB8420	ERS1420571	SAMEA4521392	ERX1771030	ERR1700742	WGS	3/12/2012	0.5	31.11	-95.15	USA	0	88	Aquic Glossudalfs	OM0	O Horizon	1	0.19	4.69	NA	NA	
TXA-OM0C0-O2	TXA041	LP_TX_	Texas	TXA	Kurth	Forest Soil	Whole Community DNA	Shotgun Metagenome	150-bp paired read library	Illumina HiSeq 2500	European Nucleotide Archive	https://www.ebi.ac.uk/ena/data/search?query=PRJEB8420	ERS1420572	SAMEA4521393	ERX1771031	ERR1700743	WGS	3/12/2012	0.5	31.11	-95.15	USA	0	88	Aquic Glossudalfs	OM0	O Horizon	2	0.34	4.4	NA	NA	
TXA-OM0C0-O3	TXA042	LP_TX_	Texas	TXA	Kurth	Forest Soil	Whole Community DNA	Shotgun Metagenome	150-bp paired read library	Illumina HiSeq 2500	European Nucleotide Archive	https://www.ebi.ac.uk/ena/data/search?query=PRJEB8420	ERS1420573	SAMEA4521394	ERX1771032	ERR1700744	WGS	3/12/2012	0.5	31.11	-95.15	USA	0	88	Aquic Glossudalfs	OM0	O Horizon	3	0.26	4.25	NA	NA	
TXA-OM1C0-M1	TXA001	LP_TX_	Texas	TXA	Kurth	Forest Soil	Whole Community DNA	Shotgun Metagenome	150-bp paired read library	Illumina HiSeq 2500	European Nucleotide Archive	https://www.ebi.ac.uk/ena/data/search?query=PRJEB8420	ERS1420574	SAMEA4521395	ERX1771033	ERR1700745	WGS	3/12/2012	0.5	31.11	-95.15	USA	0.2	88	Aquic Glossudalfs	OM1	A horizon	1	0.12	4.95	1.093	0.05	Total C and total N were calculated per plot and not per sample
TXA-OM1C0-M2	TXA002	LP_TX_	Texas	TXA	Kurth	Forest Soil	Whole Community DNA	Shotgun Metagenome	150-bp paired read library	Illumina HiSeq 2500	European Nucleotide Archive	https://www.ebi.ac.uk/ena/data/search?query=PRJEB8420	ERS1420575	SAMEA4521396	ERX1771034	ERR1700746	WGS	3/12/2012	0.5	31.11	-95.15	USA	0.2	88	Aquic Glossudalfs	OM1	A horizon	2	0.14	4.65	1.093	0.05	Total C and total N were calculated per plot and not per sample
TXA-OM1C0-M3	TXA003	LP_TX_	Texas	TXA	Kurth	Forest Soil	Whole Community DNA	Shotgun Metagenome	150-bp paired read library	Illumina HiSeq 2500	European Nucleotide Archive	https://www.ebi.ac.uk/ena/data/search?query=PRJEB8420	ERS1420576	SAMEA4521397	ERX1771035	ERR1700747	WGS	3/12/2012	0.5	31.11	-95.15	USA	0.2	88	Aquic Glossudalfs	OM1	A horizon	3	0.14	4.56	1.093	0.05	Total C and total N were calculated per plot and not per sample
TXA-OM1C0-O1	TXA004	LP_TX_	Texas	TXA	Kurth	Forest Soil	Whole Community DNA	Shotgun Metagenome	150-bp paired read library	Illumina HiSeq 2500	European Nucleotide Archive	https://www.ebi.ac.uk/ena/data/search?query=PRJEB8420	ERS1420577	SAMEA4521398	ERX1771036	ERR1700748	WGS	3/12/2012	0.5	31.11	-95.15	USA	0	88	Aquic Glossudalfs	OM1	O Horizon	1	0.26	4.69	NA	NA	
TXA-OM1C0-O2	TXA005	LP_TX_	Texas	TXA	Kurth	Forest Soil	Whole Community DNA	Shotgun Metagenome	150-bp paired read library	Illumina HiSeq 2500	European Nucleotide Archive	https://www.ebi.ac.uk/ena/data/search?query=PRJEB8420	ERS1420578	SAMEA4521399	ERX1771037	ERR1700749	WGS	3/12/2012	0.5	31.11	-95.15	USA	0	88	Aquic Glossudalfs	OM1	O Horizon	2	0.28	4.69	NA	NA	
TXA-OM1C0-O3	TXA006	LP_TX_	Texas	TXA	Kurth	Forest Soil	Whole Community DNA	Shotgun Metagenome	150-bp paired read library	Illumina HiSeq 2500	European Nucleotide Archive	https://www.ebi.ac.uk/ena/data/search?query=PRJEB8420	ERS1420579	SAMEA4521400	ERX1771038	ERR1700750	WGS	3/12/2012	0.5	31.11	-95.15	USA	0	88	Aquic Glossudalfs	OM1	O Horizon	3	0.19	5.04	NA	NA	
TXA-OM2C0-M1	TXA013	LP_TX_	Texas	TXA	Kurth	Forest Soil	Whole Community DNA	Shotgun Metagenome	150-bp paired read library	Illumina HiSeq 2500	European Nucleotide Archive	https://www.ebi.ac.uk/ena/data/search?query=PRJEB8420	ERS1420580	SAMEA4521401	ERX1771039	ERR1700751	WGS	3/12/2012	0.5	31.11	-95.15	USA	0.2	88	Aquic Glossudalfs	OM2	A horizon	1	0.11	4.56	1.208	0.047	Total C and total N were calculated per plot and not per sample
TXA-OM2C0-M2	TXA014	LP_TX_	Texas	TXA	Kurth	Forest Soil	Whole Community DNA	Shotgun Metagenome	150-bp paired read library	Illumina HiSeq 2500	European Nucleotide Archive	https://www.ebi.ac.uk/ena/data/search?query=PRJEB8420	ERS1420581	SAMEA4521402	ERX1771040	ERR1700752	WGS	3/12/2012	0.5	31.11	-95.15	USA	0.2	88	Aquic Glossudalfs	OM2	A horizon	2	0.1	5.04	1.208	0.047	Total C and total N were calculated per plot and not per sample
TXA-OM2C0-O1	TXA016	LP_TX_	Texas	TXA	Kurth	Forest Soil	Whole Community DNA	Shotgun Metagenome	150-bp paired read library	Illumina HiSeq 2500	European Nucleotide Archive	https://www.ebi.ac.uk/ena/data/search?query=PRJEB8420	ERS1420582	SAMEA4521403	ERX1771041	ERR1700753	WGS	3/12/2012	0.5	31.11	-95.15	USA	0.2	88	Aquic Glossudalfs	OM2	O Horizon	1	0.15	4.49	NA	NA	
TXA-OM2C0-O2	TXA017	LP_TX_	Texas	TXA	Kurth	Forest Soil	Whole Community DNA	Shotgun Metagenome	150-bp paired read library	Illumina HiSeq 2500	European Nucleotide Archive	https://www.ebi.ac.uk/ena/data/search?query=PRJEB8420	ERS1420583	SAMEA4521404	ERX1771042	ERR1700754	WGS	3/12/2012	0.5	31.11	-95.15	USA	0	88	Aquic Glossudalfs	OM2	O Horizon	2	0.23	5.74	NA	NA	
TXA-OM2C0-O3	TXA018	LP_TX_	Texas	TXA	Kurth	Forest Soil	Whole Community DNA	Shotgun Metagenome	150-bp paired read library	Illumina HiSeq 2500	European Nucleotide Archive	https://www.ebi.ac.uk/ena/data/search?query=PRJEB8420	ERS1420584	SAMEA4521405	ERX1771043	ERR1700755	WGS	3/12/2012	0.5	31.11	-95.15	USA	0	88	Aquic Glossudalfs	OM2	O Horizon	3	0.14	5.28	NA	NA	
TXA-OM3C0-M1	TXA025	LP_TX_	Texas	TXA	Kurth	Forest Soil	Whole Community DNA	Shotgun Metagenome	150-bp paired read library	Illumina HiSeq 2500	European Nucleotide Archive	https://www.ebi.ac.uk/ena/data/search?query=PRJEB8420	ERS1420585	SAMEA4521406	ERX1771044	ERR1700756	WGS	3/12/2012	0.5	31.11	-95.15	USA	0.2	88	Aquic Glossudalfs	OM3	A horizon	1	0.09	4.58	0.788	0.038	Total C and total N were calculated per plot and not per sample
TXA-OM3C0-M2	TXA026	LP_TX_	Texas	TXA	Kurth	Forest Soil	Whole Community DNA	Shotgun Metagenome	150-bp paired read library	Illumina HiSeq 2500	European Nucleotide Archive	https://www.ebi.ac.uk/ena/data/search?query=PRJEB8420	ERS1420586	SAMEA4521407	ERX1771045	ERR1700757	WGS	3/12/2012	0.5	31.11	-95.15	USA	0.2	88	Aquic Glossudalfs	OM3	A horizon	2	0.09	4.53	0.788	0.038	Total C and total N were calculated per plot and not per sample
TXA-OM3C0-M3	TXA027	LP_TX_	Texas	TXA	Kurth	Forest Soil	Whole Community DNA	Shotgun Metagenome	150-bp paired read library	Illumina HiSeq 2500	European Nucleotide Archive	https://www.ebi.ac.uk/ena/data/search?query=PRJEB8420	ERS1420587	SAMEA4521408	ERX1771046	ERR1700758	WGS	3/12/2012	0.5	31.11	-95.15	USA	0.2	88	Aquic Glossudalfs	OM3	A horizon	3	0.09	4.4	0.788	0.038	Total C and total N were calculated per plot and not per sample
TXA-OM3C0-O1	TXA028	LP_TX_	Texas	TXA	Kurth	Forest Soil	Whole Community DNA	Shotgun Metagenome	150-bp paired read library	Illumina HiSeq 2500	European Nucleotide Archive	https://www.ebi.ac.uk/ena/data/search?query=PRJEB8420	ERS1420588	SAMEA4521409	ERX1771047	ERR1700759	WGS	3/12/2012	0.5	31.11	-95.15	USA	0	88	Aquic Glossudalfs	OM3	O Horizon	1	0.11	4.68	NA	NA	
TXA-OM3C0-O2	TXA029	LP_TX_	Texas	TXA	Kurth	Forest Soil	Whole Community DNA	Shotgun Metagenome	150-bp paired read library	Illumina HiSeq 2500	European Nucleotide Archive	https://www.ebi.ac.uk/ena/data/search?query=PRJEB8420	ERS1420589	SAMEA4521410	ERX1771048	ERR1700760	WGS	3/12/2012	0.5	31.11	-95.15	USA	0	88	Aquic Glossudalfs	OM3	O Horizon	2	0.1	4.67	NA	NA	
TXA-OM3C0-O3	TXA030	LP_TX_	Texas	TXA	Kurth	Forest Soil	Whole Community DNA	Shotgun Metagenome	150-bp paired read library	Illumina HiSeq 2500	European Nucleotide Archive	https://www.ebi.ac.uk/ena/data/search?query=PRJEB8420	ERS1420590	SAMEA4521411	ERX1771049	ERR1700761	WGS	3/12/2012	0.5	31.11	-95.15	USA	0	88	Aquic Glossudalfs	OM3	O Horizon	3	0.19	4.42	NA	NA	
SL-OM3C0-LFH-1	SL-OM3C0-LFH-1	SBS_BC_	British Columbia	SL	Skulow Lake	Forest Soil	Whole Community DNA	Shotgun Metagenome	100-bp paired read library	Illumina HiSeq X Ten	European Nucleotide Archive	https://www.ebi.ac.uk/ena/data/search?query=PRJEB8420	ERS1458990	SAMEA4559811	ERX1811794	ERR1742252	WGS	7/15/2007	0.5	52.32	-121.92	Canada	0.02	1050	Orthic Gray Luvisol	OM3	LFH	1	NA	5.87	5.73	0.21	
SL-OM3C0-Ae-1	SL-OM3C0-Ae-1	SBS_BC_	British Columbia	SL	Skulow Lake	Forest Soil	Whole Community DNA	Shotgun Metagenome	100-bp paired read library	Illumina HiSeq X Ten	European Nucleotide Archive	https://www.ebi.ac.uk/ena/data/search?query=PRJEB8420	ERS1458991	SAMEA4559812	ERX1811795	ERR1742253	WGS	7/15/2007	0.5	52.32	-121.92	Canada	0.065	1050	Orthic Gray Luvisol	OM3	Ae	1	NA	6.13	0.28	0.03	
SL-OM3C0-AB-1	SL-OM3C0-AB-1	SBS_BC_	British Columbia	SL	Skulow Lake	Forest Soil	Whole Community DNA	Shotgun Metagenome	100-bp paired read library	Illumina HiSeq X Ten	European Nucleotide Archive	https://www.ebi.ac.uk/ena/data/search?query=PRJEB8420	ERS1458992	SAMEA4559813	ERX1811796	ERR1742254	WGS	7/15/2007	0.5	52.32	-121.92	Canada	0.13	1050	Orthic Gray Luvisol	OM3	AB	1	NA	6.5	0.36	0.03	
SL-OM3C0-Bt-1	SL-OM3C0-Bt-1	SBS_BC_	British Columbia	SL	Skulow Lake	Forest Soil	Whole Community DNA	Shotgun Metagenome	100-bp paired read library	Illumina HiSeq X Ten	European Nucleotide Archive	https://www.ebi.ac.uk/ena/data/search?query=PRJEB8420	ERS1458993	SAMEA4559814	ERX1811797	ERR1742255	WGS	7/15/2007	0.5	52.32	-121.92	Canada	0.385	1050	Orthic Gray Luvisol	OM3	Bt	1	NA	6.9	0.58	0.04	
SL-OM3C0-LFH-2	SL-OM3C0-LFH-2	SBS_BC_	British Columbia	SL	Skulow Lake	Forest Soil	Whole Community DNA	Shotgun Metagenome	100-bp paired read library	Illumina HiSeq X Ten	European Nucleotide Archive	https://www.ebi.ac.uk/ena/data/search?query=PRJEB8420	ERS1458994	SAMEA4559815	ERX1811798	ERR1742256	WGS	7/15/2007	0.5	52.32	-121.92	Canada	0.62	1050	Orthic Gray Luvisol	OM3	LFH	2	NA	6.18	2.26	0.1	
SL-OM3C0-Ae-2	SL-OM3C0-Ae-2	SBS_BC_	British Columbia	SL	Skulow Lake	Forest Soil	Whole Community DNA	Shotgun Metagenome	100-bp paired read library	Illumina HiSeq X Ten	European Nucleotide Archive	https://www.ebi.ac.uk/ena/data/search?query=PRJEB8420	ERS1458995	SAMEA4559816	ERX1811799	ERR1742257	WGS	7/15/2007	0.5	52.32	-121.92	Canada	0.02	1050	Orthic Gray Luvisol	OM3	Ae	2	NA	6.05	1.13	0.06	
SL-OM3C0-AB-2	SL-OM3C0-AB-2	SBS_BC_	British Columbia	SL	Skulow Lake	Forest Soil	Whole Community DNA	Shotgun Metagenome	100-bp paired read library	Illumina HiSeq X Ten	European Nucleotide Archive	https://www.ebi.ac.uk/ena/data/search?query=PRJEB8420	ERS1458996	SAMEA4559817	ERX1811800	ERR1742258	WGS	7/15/2007	0.5	52.32	-121.92	Canada	0.075	1050	Orthic Gray Luvisol	OM3	AB	2	NA	6.27	0.36	0.03	
SL-OM3C0-Bt-2	SL-OM3C0-Bt-2	SBS_BC_	British Columbia	SL	Skulow Lake	Forest Soil	Whole Community DNA	Shotgun Metagenome	100-bp paired read library	Illumina HiSeq X Ten	European Nucleotide Archive	https://www.ebi.ac.uk/ena/data/search?query=PRJEB8420	ERS1458997	SAMEA4559818	ERX1811801	ERR1742259	WGS	7/15/2007	0.5	52.32	-121.92	Canada	0.2	1050	Orthic Gray Luvisol	OM3	Bt	2	NA	6.76	0.65	0.05	
SL-OM3C0-LFH-3	SL-OM3C0-LFH-3	SBS_BC_	British Columbia	SL	Skulow Lake	Forest Soil	Whole Community DNA	Shotgun Metagenome	100-bp paired read library	Illumina HiSeq X Ten	European Nucleotide Archive	https://www.ebi.ac.uk/ena/data/search?query=PRJEB8420	ERS1458998	SAMEA4559819	ERX1811802	ERR1742260	WGS	7/15/2007	0.5	52.32	-121.92	Canada	0.27	1050	Orthic Gray Luvisol	OM3	LFH	3	NA	6.03	5.1	0.17	
SL-OM3C0-Ae-3	SL-OM3C0-Ae-3	SBS_BC_	British Columbia	SL	Skulow Lake	Forest Soil	Whole Community DNA	Shotgun Metagenome	100-bp paired read library	Illumina HiSeq X Ten	European Nucleotide Archive	https://www.ebi.ac.uk/ena/data/search?query=PRJEB8420	ERS1458999	SAMEA4559820	ERX1811803	ERR1742261	WGS	7/15/2007	0.5	52.32	-121.92	Canada	0.555	1050	Orthic Gray Luvisol	OM3	Ae	3	NA	5.16	2.1	0.09	
SL-OM3C0-AB-3	SL-OM3C0-AB-3	SBS_BC_	British Columbia	SL	Skulow Lake	Forest Soil	Whole Community DNA	Shotgun Metagenome	100-bp paired read library	Illumina HiSeq X Ten	European Nucleotide Archive	https://www.ebi.ac.uk/ena/data/search?query=PRJEB8420	ERS1459000	SAMEA4559821	ERX1811804	ERR1742262	WGS	7/15/2007	0.5	52.32	-121.92	Canada	0.105	1050	Orthic Gray Luvisol	OM3	AB	3	NA	5.72	0.88	0.04	
SL-OM3C0-Bt-3	SL-OM3C0-Bt-3	SBS_BC_	British Columbia	SL	Skulow Lake	Forest Soil	Whole Community DNA	Shotgun Metagenome	100-bp paired read library	Illumina HiSeq X Ten	European Nucleotide Archive	https://www.ebi.ac.uk/ena/data/search?query=PRJEB8420	ERS1459001	SAMEA4559822	ERX1811805	ERR1742263	WGS	7/15/2007	0.5	52.32	-121.92	Canada	0.185	1050	Orthic Gray Luvisol	OM3	Bt	3	NA	6.45	0.64	0.05	
SL-OM0C0-LFH-1	SL-OM0C0-LFH-1	SBS_BC_	British Columbia	SL	Skulow Lake	Forest Soil	Whole Community DNA	Shotgun Metagenome	100-bp paired read library	Illumina HiSeq X Ten	European Nucleotide Archive	https://www.ebi.ac.uk/ena/data/search?query=PRJEB8420	ERS1459002	SAMEA4559823	ERX1811806	ERR1742264	WGS	7/15/2007	0.5	52.32	-121.92	Canada	0.395	1050	Orthic Gray Luvisol	REF	LFH	1	NA	4.25	14.4	0.4	
SL-OM0C0-Ahe-1	SL-OM0C0-Ahe-1	SBS_BC_	British Columbia	SL	Skulow Lake	Forest Soil	Whole Community DNA	Shotgun Metagenome	100-bp paired read library	Illumina HiSeq X Ten	European Nucleotide Archive	https://www.ebi.ac.uk/ena/data/search?query=PRJEB8420	ERS1459003	SAMEA4559824	ERX1811807	ERR1742265	WGS	7/15/2007	0.5	52.32	-121.92	Canada	0.545	1050	Orthic Gray Luvisol	REF	Ahe	1	NA	4.69	1.8	0.08	
SL-OM0C0-Ae-1	SL-OM0C0-Ae-1	SBS_BC_	British Columbia	SL	Skulow Lake	Forest Soil	Whole Community DNA	Shotgun Metagenome	100-bp paired read library	Illumina HiSeq X Ten	European Nucleotide Archive	https://www.ebi.ac.uk/ena/data/search?query=PRJEB8420	ERS1459004	SAMEA4559825	ERX1811808	ERR1742266	WGS	7/15/2007	0.5	52.32	-121.92	Canada	0.003	1050	Orthic Gray Luvisol	REF	Ae	1	NA	5.42	0.95	0.05	
SL-OM0C0-AB-1	SL-OM0C0-AB-1	SBS_BC_	British Columbia	SL	Skulow Lake	Forest Soil	Whole Community DNA	Shotgun Metagenome	100-bp paired read library	Illumina HiSeq X Ten	European Nucleotide Archive	https://www.ebi.ac.uk/ena/data/search?query=PRJEB8420	ERS1459005	SAMEA4559826	ERX1811809	ERR1742267	WGS	7/15/2007	0.5	52.32	-121.92	Canada	0.062	1050	Orthic Gray Luvisol	REF	AB	1	NA	5.92	0.67	0.05	
SL-OM0C0-Bt-1	SL-OM0C0-Bt-1	SBS_BC_	British Columbia	SL	Skulow Lake	Forest Soil	Whole Community DNA	Shotgun Metagenome	100-bp paired read library	Illumina HiSeq X Ten	European Nucleotide Archive	https://www.ebi.ac.uk/ena/data/search?query=PRJEB8420	ERS1459006	SAMEA4559827	ERX1811810	ERR1742268	WGS	7/15/2007	0.5	52.32	-121.92	Canada	0.333	1050	Orthic Gray Luvisol	REF	Bt	1	NA	6.72	0.89	0.06	
SL-OM0C0-LFH-2	SL-OM0C0-LFH-2	SBS_BC_	British Columbia	SL	Skulow Lake	Forest Soil	Whole Community DNA	Shotgun Metagenome	100-bp paired read library	Illumina HiSeq X Ten	European Nucleotide Archive	https://www.ebi.ac.uk/ena/data/search?query=PRJEB8420	ERS1459007	SAMEA4559828	ERX1811811	ERR1742269	WGS	7/15/2007	0.5	52.32	-121.92	Canada	0.528	1050	Orthic Gray Luvisol	REF	LFH	2	NA	4.5	26.64	0.55	
SL-OM0C0-Ahe-2	SL-OM0C0-Ahe-2	SBS_BC_	British Columbia	SL	Skulow Lake	Forest Soil	Whole Community DNA	Shotgun Metagenome	100-bp paired read library	Illumina HiSeq X Ten	European Nucleotide Archive	https://www.ebi.ac.uk/ena/data/search?query=PRJEB8420	ERS1459008	SAMEA4559829	ERX1811812	ERR1742270	WGS	7/15/2007	0.5	52.32	-121.92	Canada	0.002	1050	Orthic Gray Luvisol	REF	Ahe	2	NA	5.07	8.71	0.25	
SL-OM0C0-Ae-2	SL-OM0C0-Ae-2	SBS_BC_	British Columbia	SL	Skulow Lake	Forest Soil	Whole Community DNA	Shotgun Metagenome	100-bp paired read library	Illumina HiSeq X Ten	European Nucleotide Archive	https://www.ebi.ac.uk/ena/data/search?query=PRJEB8420	ERS1459009	SAMEA4559830	ERX1811813	ERR1742271	WGS	7/15/2007	0.5	52.32	-121.92	Canada	0.067	1050	Orthic Gray Luvisol	REF	Ae	2	NA	6.22	0.73	0.05	
SL-OM0C0-AB-2	SL-OM0C0-AB-2	SBS_BC_	British Columbia	SL	Skulow Lake	Forest Soil	Whole Community DNA	Shotgun Metagenome	100-bp paired read library	Illumina HiSeq X Ten	European Nucleotide Archive	https://www.ebi.ac.uk/ena/data/search?query=PRJEB8420	ERS1459010	SAMEA4559831	ERX1811814	ERR1742272	WGS	7/15/2007	0.5	52.32	-121.92	Canada	0.217	1050	Orthic Gray Luvisol	REF	AB	2	NA	6.34	1.06	0.05	
SL-OM0C0-Bt-2	SL-OM0C0-Bt-2	SBS_BC_	British Columbia	SL	Skulow Lake	Forest Soil	Whole Community DNA	Shotgun Metagenome	100-bp paired read library	Illumina HiSeq X Ten	European Nucleotide Archive	https://www.ebi.ac.uk/ena/data/search?query=PRJEB8420	ERS1459011	SAMEA4559832	ERX1811815	ERR1742273	WGS	7/15/2007	0.5	52.32	-121.92	Canada	0.597	1050	Orthic Gray Luvisol	REF	Bt	2	NA	6.9	0.74	0.05	
SL-OM0C0-LFH-3	SL-OM0C0-LFH-3	SBS_BC_	British Columbia	SL	Skulow Lake	Forest Soil	Whole Community DNA	Shotgun Metagenome	100-bp paired read library	Illumina HiSeq X Ten	European Nucleotide Archive	https://www.ebi.ac.uk/ena/data/search?query=PRJEB8420	ERS1459012	SAMEA4559833	ERX1811816	ERR1742274	WGS	7/15/2007	0.5	52.32	-121.92	Canada	0.002	1050	Orthic Gray Luvisol	REF	LFH	3	NA	5.59	27.46	0.61	
SL-OM0C0-Ae-3	SL-OM0C0-Ae-3	SBS_BC_	British Columbia	SL	Skulow Lake	Forest Soil	Whole Community DNA	Shotgun Metagenome	100-bp paired read library	Illumina HiSeq X Ten	European Nucleotide Archive	https://www.ebi.ac.uk/ena/data/search?query=PRJEB8420	ERS1459013	SAMEA4559834	ERX1811817	ERR1742275	WGS	7/15/2007	0.5	52.32	-121.92	Canada	0.042	1050	Orthic Gray Luvisol	REF	Ae	3	NA	5.7	1.12	0.08	
SL-OM0C0-AB-3	SL-OM0C0-AB-3	SBS_BC_	British Columbia	SL	Skulow Lake	Forest Soil	Whole Community DNA	Shotgun Metagenome	100-bp paired read library	Illumina HiSeq X Ten	European Nucleotide Archive	https://www.ebi.ac.uk/ena/data/search?query=PRJEB8420	ERS1459014	SAMEA4559835	ERX1811818	ERR1742276	WGS	7/15/2007	0.5	52.32	-121.92	Canada	0.272	1050	Orthic Gray Luvisol	REF	AB	3	NA	5.77	0.6	0.05	
SL-OM0C0-Bt-3	SL-OM0C0-Bt-3	SBS_BC_	British Columbia	SL	Skulow Lake	Forest Soil	Whole Community DNA	Shotgun Metagenome	100-bp paired read library	Illumina HiSeq X Ten	European Nucleotide Archive	https://www.ebi.ac.uk/ena/data/search?query=PRJEB8420	ERS1459015	SAMEA4559836	ERX1811819	ERR1742277	WGS	7/15/2007	0.5	52.32	-121.92	Canada	0.492	1050	Orthic Gray Luvisol	REF	Bt	3	NA	6.6	0.61	0.05	

**Table 4:** Sequencing and sample data for all field 16S and ITS amplicon libraries

**SIP Substrate**	**Sample ID**	**SIP Status**	**Sample Alias**	**Ecozone**	**Region**	**Site**	**Environmental Source**	**DNA Source**	**Library Preparation**	**Library Type**	**Common Name**	**Instrument Model**	**Data Repository**	**Study Accession**	**Sample Accession**	**Secondary Sample Accession**	**Experiment Accession**	**Run Accession**	**Target Gene**	**Target Gene Subfragment**	**Barcode**	**Primer**	**Collection Date**	**Sample weight for DNA extraction**	**Latitude**	**Longitude**	**Country**	**Sampling Depth**	**Elevation**	**Mean Annual Temperature (Celsius)**	**Mean Annual Precipitation (mm)**	**Soil Classification**	**Tree Cover**	**Climatic Zone**	**LTSP Treatment**	**Horizon**	**Moisture Content**	**Total Carbon**	**Total Nitrogen**	**pH**	**Soil Bulk Density**	**CN Ratio**
Cellulose	BL025	12C	IIKFCBR01.BL025_12C.bact	PP_CA_	California	BL	Forest Soil	Whole Community DNA	PCR	Amplicon	Pyrotag library	454 GS FLX Titanium	European Nucleotide Archive	https://www.ebi.ac.uk/ena/data/search?query=PRJEB9761	ERS803692	SAMEA3496543	ERX1051796	ERR974810	16S rRNA	27F	TGACGTATGT	AGAGTTTGATCMTGGCTCAG	9/16/2011	0.5	38.88	-120.64	USA	0.1	1350	11.2	55	Mesic Ultic Haploxeralfs	Ponderosa pine, sugar pine, white fir, giant sequoia	Csa, Mediterranean hot summer	OM1	O horizon	18	NA	NA	4.79	NA	NA
Cellulose	BL026	12C	IIKFCBR01.BL026_12C.bact	PP_CA_	California	BL	Forest Soil	Whole Community DNA	PCR	Amplicon	Pyrotag library	454 GS FLX Titanium	European Nucleotide Archive	https://www.ebi.ac.uk/ena/data/search?query=PRJEB9761	ERS803694	SAMEA3496545	ERX1051798	ERR974812	16S rRNA	27F	ACTACTATGT	AGAGTTTGATCMTGGCTCAG	9/16/2011	0.5	38.88	-120.64	USA	0.3	1350	11.2	55	Mesic Ultic Haploxeralfs	Ponderosa pine, sugar pine, white fir, giant sequoia	Csa, Mediterranean hot summer	OM1	A horizon	22	5.47	0.28	5.74	NA	19.78
Cellulose	BL031	12C	IIKFCBR01.BL031_12C.bact	PP_CA_	California	BL	Forest Soil	Whole Community DNA	PCR	Amplicon	Pyrotag library	454 GS FLX Titanium	European Nucleotide Archive	https://www.ebi.ac.uk/ena/data/search?query=PRJEB9761	ERS803696	SAMEA3496547	ERX1051800	ERR974814	16S rRNA	27F	ACGCGATCGA	AGAGTTTGATCMTGGCTCAG	9/16/2011	0.5	38.88	-120.64	USA	0.1	1350	11.2	55	Mesic Ultic Haploxeralfs	Ponderosa pine, sugar pine, white fir, giant sequoia	Csa, Mediterranean hot summer	OM2	O horizon	29	NA	NA	5.73	NA	NA
Cellulose	BL032	12C	IIKFCBR01.BL032_12C.bact	PP_CA_	California	BL	Forest Soil	Whole Community DNA	PCR	Amplicon	Pyrotag library	454 GS FLX Titanium	European Nucleotide Archive	https://www.ebi.ac.uk/ena/data/search?query=PRJEB9761	ERS803698	SAMEA3496549	ERX1051802	ERR974816	16S rRNA	27F	AGACTATACT	AGAGTTTGATCMTGGCTCAG	9/16/2011	0.5	38.88	-120.64	USA	0.3	1350	11.2	55	Mesic Ultic Haploxeralfs	Ponderosa pine, sugar pine, white fir, giant sequoia	Csa, Mediterranean hot summer	OM2	A horizon	21	5.35	0.26	5.78	NA	20.64
Cellulose	BL037	12C	IIKFCBR01.BL037_12C.bact	PP_CA_	California	BL	Forest Soil	Whole Community DNA	PCR	Amplicon	Pyrotag library	454 GS FLX Titanium	European Nucleotide Archive	https://www.ebi.ac.uk/ena/data/search?query=PRJEB9761	ERS803700	SAMEA3496551	ERX1051804	ERR974818	16S rRNA	27F	AGTATACATA	AGAGTTTGATCMTGGCTCAG	9/16/2011	0.5	38.88	-120.64	USA	0.1	1350	11.2	55	Mesic Ultic Haploxeralfs	Ponderosa pine, sugar pine, white fir, giant sequoia	Csa, Mediterranean hot summer	OM3	O horizon	21.1	NA	NA	5.19	NA	NA
Cellulose	BL038	12C	IIKFCBR01.BL038_12C.bact	PP_CA_	California	BL	Forest Soil	Whole Community DNA	PCR	Amplicon	Pyrotag library	454 GS FLX Titanium	European Nucleotide Archive	https://www.ebi.ac.uk/ena/data/search?query=PRJEB9761	ERS803702	SAMEA3496553	ERX1051806	ERR974820	16S rRNA	27F	AGCGTCGTCT	AGAGTTTGATCMTGGCTCAG	9/16/2011	0.5	38.88	-120.64	USA	0.3	1350	11.2	55	Mesic Ultic Haploxeralfs	Ponderosa pine, sugar pine, white fir, giant sequoia	Csa, Mediterranean hot summer	OM3	A horizon	22	5.27	0.28	5.42	NA	18.87
Cellulose	BL043	12C	IIKFCBR01.BL043_12C.bact	PP_CA_	California	BL	Forest Soil	Whole Community DNA	PCR	Amplicon	Pyrotag library	454 GS FLX Titanium	European Nucleotide Archive	https://www.ebi.ac.uk/ena/data/search?query=PRJEB9761	ERS803704	SAMEA3496555	ERX1051808	ERR974822	16S rRNA	27F	AGTGCTACGA	AGAGTTTGATCMTGGCTCAG	9/16/2011	0.5	38.88	-120.64	USA	0.1	1350	11.2	55	Mesic Ultic Haploxeralfs	Ponderosa pine, sugar pine, white fir, giant sequoia	Csa, Mediterranean hot summer	REF	O horizon	27	NA	NA	4.51	NA	NA
Cellulose	BL044	12C	IIKFCBR01.BL044_12C.bact	PP_CA_	California	BL	Forest Soil	Whole Community DNA	PCR	Amplicon	Pyrotag library	454 GS FLX Titanium	European Nucleotide Archive	https://www.ebi.ac.uk/ena/data/search?query=PRJEB9761	ERS803706	SAMEA3496557	ERX1051810	ERR974824	16S rRNA	27F	ATAGAGTACT	AGAGTTTGATCMTGGCTCAG	9/16/2011	0.5	38.88	-120.64	USA	0.3	1350	11.2	55	Mesic Ultic Haploxeralfs	Ponderosa pine, sugar pine, white fir, giant sequoia	Csa, Mediterranean hot summer	REF	A horizon	18	6.3	0.31	5.48	NA	20.32
Cellulose	BR049	12C	IIKFCBR01.BR049_12C.bact	PP_CA_	California	BR	Forest Soil	Whole Community DNA	PCR	Amplicon	Pyrotag library	454 GS FLX Titanium	European Nucleotide Archive	https://www.ebi.ac.uk/ena/data/search?query=PRJEB9761	ERS803708	SAMEA3496559	ERX1051812	ERR974826	16S rRNA	27F	TACACGTGAT	AGAGTTTGATCMTGGCTCAG	9/16/2011	0.5	39.55	-121.04	USA	0.1	1135	11.2	55	Mesic Ultic Haploxeralfs	Ponderosa pine, sugar pine, white fir, giant sequoia	Csa, Mediterranean hot summer	OM1	O horizon	54	NA	NA	5.39	NA	NA
Cellulose	BR050	12C	IIKFCBR01.BR050_12C.bact	PP_CA_	California	BR	Forest Soil	Whole Community DNA	PCR	Amplicon	Pyrotag library	454 GS FLX Titanium	European Nucleotide Archive	https://www.ebi.ac.uk/ena/data/search?query=PRJEB9761	ERS803710	SAMEA3496561	ERX1051814	ERR974828	16S rRNA	27F	TGTACTACTC	AGAGTTTGATCMTGGCTCAG	9/16/2011	0.5	39.55	-121.04	USA	0.3	1135	11.2	55	Mesic Ultic Haploxeralfs	Ponderosa pine, sugar pine, white fir, giant sequoia	Csa, Mediterranean hot summer	OM1	A horizon	35	6.39	0.25	5.87	NA	25.8
Cellulose	BR055	12C	IIKFCBR01.BR055_12C.bact	PP_CA_	California	BR	Forest Soil	Whole Community DNA	PCR	Amplicon	Pyrotag library	454 GS FLX Titanium	European Nucleotide Archive	https://www.ebi.ac.uk/ena/data/search?query=PRJEB9761	ERS803712	SAMEA3496563	ERX1051816	ERR974830	16S rRNA	27F	TACGCTGTCT	AGAGTTTGATCMTGGCTCAG	9/16/2011	0.5	39.55	-121.04	USA	0.1	1135	11.2	55	Mesic Ultic Haploxeralfs	Ponderosa pine, sugar pine, white fir, giant sequoia	Csa, Mediterranean hot summer	OM2	O horizon	50	NA	NA	5.73	NA	NA
Cellulose	BR056	12C	IIKFCBR01.BR056_12C.bact	PP_CA_	California	BR	Forest Soil	Whole Community DNA	PCR	Amplicon	Pyrotag library	454 GS FLX Titanium	European Nucleotide Archive	https://www.ebi.ac.uk/ena/data/search?query=PRJEB9761	ERS803714	SAMEA3496565	ERX1051818	ERR974832	16S rRNA	27F	CGTAGACTAG	AGAGTTTGATCMTGGCTCAG	9/16/2011	0.5	39.55	-121.04	USA	0.3	1135	11.2	55	Mesic Ultic Haploxeralfs	Ponderosa pine, sugar pine, white fir, giant sequoia	Csa, Mediterranean hot summer	OM2	A horizon	36	5.71	0.28	6.1	NA	20.91
Cellulose	BR061	12C	IIKFCBR01.BR061_12C.bact	PP_CA_	California	BR	Forest Soil	Whole Community DNA	PCR	Amplicon	Pyrotag library	454 GS FLX Titanium	European Nucleotide Archive	https://www.ebi.ac.uk/ena/data/search?query=PRJEB9761	ERS803716	SAMEA3496567	ERX1051820	ERR974834	16S rRNA	27F	TCGATCACGT	AGAGTTTGATCMTGGCTCAG	9/16/2011	0.5	39.55	-121.04	USA	0.1	1135	11.2	55	Mesic Ultic Haploxeralfs	Ponderosa pine, sugar pine, white fir, giant sequoia	Csa, Mediterranean hot summer	OM3	O horizon	40	16.4	0.61	5.81	NA	27.1
Cellulose	BR062	12C	IIKFCBR01.BR062_12C.bact	PP_CA_	California	BR	Forest Soil	Whole Community DNA	PCR	Amplicon	Pyrotag library	454 GS FLX Titanium	European Nucleotide Archive	https://www.ebi.ac.uk/ena/data/search?query=PRJEB9761	ERS803718	SAMEA3496569	ERX1051822	ERR974836	16S rRNA	27F	TACTCTCGTG	AGAGTTTGATCMTGGCTCAG	9/16/2011	0.5	39.55	-121.04	USA	0.3	1135	11.2	55	Mesic Ultic Haploxeralfs	Ponderosa pine, sugar pine, white fir, giant sequoia	Csa, Mediterranean hot summer	OM3	A horizon	34	5.2	0.27	5.52	NA	19.2
Cellulose	BR067	12C	IIKFCBR01.BR067_12C.bact	PP_CA_	California	BR	Forest Soil	Whole Community DNA	PCR	Amplicon	Pyrotag library	454 GS FLX Titanium	European Nucleotide Archive	https://www.ebi.ac.uk/ena/data/search?query=PRJEB9761	ERS803720	SAMEA3496571	ERX1051824	ERR974838	16S rRNA	27F	TCTAGCGACT	AGAGTTTGATCMTGGCTCAG	9/16/2011	0.5	39.55	-121.04	USA	0.1	1135	11.2	55	Mesic Ultic Haploxeralfs	Ponderosa pine, sugar pine, white fir, giant sequoia	Csa, Mediterranean hot summer	REF	O horizon	61	NA	NA	4.95	NA	NA
Cellulose	BR068	12C	IIKFCBR01.BR068_12C.bact	PP_CA_	California	BR	Forest Soil	Whole Community DNA	PCR	Amplicon	Pyrotag library	454 GS FLX Titanium	European Nucleotide Archive	https://www.ebi.ac.uk/ena/data/search?query=PRJEB9761	ERS803722	SAMEA3496573	ERX1051826	ERR974840	16S rRNA	27F	TCGTCGCTCG	AGAGTTTGATCMTGGCTCAG	9/16/2011	0.5	39.55	-121.04	USA	0.3	1135	11.2	55	Mesic Ultic Haploxeralfs	Ponderosa pine, sugar pine, white fir, giant sequoia	Csa, Mediterranean hot summer	REF	A horizon	28	5.8	0.25	5.95	NA	23.2
Cellulose	LH001	12C	IIKFCBR01.LH001_12C.bact	PP_CA_	California	LH	Forest Soil	Whole Community DNA	PCR	Amplicon	Pyrotag library	454 GS FLX Titanium	European Nucleotide Archive	https://www.ebi.ac.uk/ena/data/search?query=PRJEB9761	ERS803724	SAMEA3496575	ERX1051828	ERR974842	16S rRNA	27F	CGAGAGATAC	AGAGTTTGATCMTGGCTCAG	9/16/2011	0.5	39.26	-120.78	USA	0.1	1268	11.2	55	Mesic Ultic Haploxeralfs	Ponderosa pine, sugar pine, white fir, giant sequoia	Csa, Mediterranean hot summer	OM1	O horizon	14	NA	NA	3.67	NA	NA
Cellulose	LH002	12C	IIKFCBR01.LH002_12C.bact	PP_CA_	California	LH	Forest Soil	Whole Community DNA	PCR	Amplicon	Pyrotag library	454 GS FLX Titanium	European Nucleotide Archive	https://www.ebi.ac.uk/ena/data/search?query=PRJEB9761	ERS803726	SAMEA3496577	ERX1051830	ERR974844	16S rRNA	27F	CTCGCGTGTC	AGAGTTTGATCMTGGCTCAG	9/16/2011	0.5	39.26	-120.78	USA	0.3	1268	11.2	55	Mesic Ultic Haploxeralfs	Ponderosa pine, sugar pine, white fir, giant sequoia	Csa, Mediterranean hot summer	OM1	A horizon	19	3.08	0.12	5.18	NA	24.79
Cellulose	LH007	12C	IIKFCBR01.LH007_12C.bact	PP_CA_	California	LH	Forest Soil	Whole Community DNA	PCR	Amplicon	Pyrotag library	454 GS FLX Titanium	European Nucleotide Archive	https://www.ebi.ac.uk/ena/data/search?query=PRJEB9761	ERS803728	SAMEA3496579	ERX1051832	ERR974846	16S rRNA	27F	TCACGTACTA	AGAGTTTGATCMTGGCTCAG	9/16/2011	0.5	39.26	-120.78	USA	0.1	1268	11.2	55	Mesic Ultic Haploxeralfs	Ponderosa pine, sugar pine, white fir, giant sequoia	Csa, Mediterranean hot summer	OM2	O horizon	10	NA	NA	4.58	NA	NA
Cellulose	LH008	12C	IIKFCBR01.LH008_12C.bact	PP_CA_	California	LH	Forest Soil	Whole Community DNA	PCR	Amplicon	Pyrotag library	454 GS FLX Titanium	European Nucleotide Archive	https://www.ebi.ac.uk/ena/data/search?query=PRJEB9761	ERS803730	SAMEA3496581	ERX1051834	ERR974848	16S rRNA	27F	TCTCTATGCG	AGAGTTTGATCMTGGCTCAG	9/16/2011	0.5	39.26	-120.78	USA	0.3	1268	11.2	55	Mesic Ultic Haploxeralfs	Ponderosa pine, sugar pine, white fir, giant sequoia	Csa, Mediterranean hot summer	OM2	A horizon	17.1	3.63	0.16	6	NA	22.3
Cellulose	LH013	12C	IIKFCBR01.LH013_12C.bact	PP_CA_	California	LH	Forest Soil	Whole Community DNA	PCR	Amplicon	Pyrotag library	454 GS FLX Titanium	European Nucleotide Archive	https://www.ebi.ac.uk/ena/data/search?query=PRJEB9761	ERS803732	SAMEA3496583	ERX1051836	ERR974850	16S rRNA	27F	CACGCTACGT	AGAGTTTGATCMTGGCTCAG	9/16/2011	0.5	39.26	-120.78	USA	0.1	1268	11.2	55	Mesic Ultic Haploxeralfs	Ponderosa pine, sugar pine, white fir, giant sequoia	Csa, Mediterranean hot summer	OM3	O horizon	14.1	36.4	1.07	5.08	NA	34.1
Cellulose	LH014	12C	IIKFCBR01.LH014_12C.bact	PP_CA_	California	LH	Forest Soil	Whole Community DNA	PCR	Amplicon	Pyrotag library	454 GS FLX Titanium	European Nucleotide Archive	https://www.ebi.ac.uk/ena/data/search?query=PRJEB9761	ERS803734	SAMEA3496585	ERX1051838	ERR974852	16S rRNA	27F	CGCAGTACGA	AGAGTTTGATCMTGGCTCAG	9/16/2011	0.5	39.26	-120.78	USA	0.3	1268	11.2	55	Mesic Ultic Haploxeralfs	Ponderosa pine, sugar pine, white fir, giant sequoia	Csa, Mediterranean hot summer	OM3	A horizon	17	3.3	0.15	4.99	NA	21.55
Cellulose	LH019	12C	IIKFCBR01.LH019_12C.bact	PP_CA_	California	LH	Forest Soil	Whole Community DNA	PCR	Amplicon	Pyrotag library	454 GS FLX Titanium	European Nucleotide Archive	https://www.ebi.ac.uk/ena/data/search?query=PRJEB9761	ERS803736	SAMEA3496587	ERX1051840	ERR974854	16S rRNA	27F	CGACGTGACT	AGAGTTTGATCMTGGCTCAG	9/16/2011	0.5	39.26	-120.78	USA	0.1	1268	11.2	55	Mesic Ultic Haploxeralfs	Ponderosa pine, sugar pine, white fir, giant sequoia	Csa, Mediterranean hot summer	REF	O horizon	22	NA	NA	5.57	NA	NA
Cellulose	LH020	12C	IIKFCBR01.LH020_12C.bact	PP_CA_	California	LH	Forest Soil	Whole Community DNA	PCR	Amplicon	Pyrotag library	454 GS FLX Titanium	European Nucleotide Archive	https://www.ebi.ac.uk/ena/data/search?query=PRJEB9761	ERS803738	SAMEA3496589	ERX1051842	ERR974856	16S rRNA	27F	CGTCTAGTAC	AGAGTTTGATCMTGGCTCAG	9/16/2011	0.5	39.26	-120.78	USA	0.3	1268	11.2	55	Mesic Ultic Haploxeralfs	Ponderosa pine, sugar pine, white fir, giant sequoia	Csa, Mediterranean hot summer	REF	A horizon	20.1	4.14	0.22	6.6	NA	19.14
Cellulose	BL025	12C	IIKFCBR02.BL025_12C.fungi	PP_CA_	California	BL	Forest Soil	Whole Community DNA	PCR	Amplicon	Pyrotag library	454 GS FLX Titanium	European Nucleotide Archive	https://www.ebi.ac.uk/ena/data/search?query=PRJEB9761	ERS803740	SAMEA3496591	ERX1051844	ERR974858	rRNA intergenic spacer analysis	ITS2	ACAGTATATA	TCCTCCGCTTATTGATATGC	9/16/2011	0.5	38.88	-120.64	USA	0.1	1350	11.2	55	Mesic Ultic Haploxeralfs	Ponderosa pine, sugar pine, white fir, giant sequoia	Csa, Mediterranean hot summer	OM1	O horizon	18	NA	NA	4.79	NA	NA
Cellulose	BL026	12C	IIKFCBR02.BL026_12C.fungi	PP_CA_	California	BL	Forest Soil	Whole Community DNA	PCR	Amplicon	Pyrotag library	454 GS FLX Titanium	European Nucleotide Archive	https://www.ebi.ac.uk/ena/data/search?query=PRJEB9761	ERS803742	SAMEA3496593	ERX1051846	ERR974860	rRNA intergenic spacer analysis	ITS2	TAGAGACGAG	TCCTCCGCTTATTGATATGC	9/16/2011	0.5	38.88	-120.64	USA	0.3	1350	11.2	55	Mesic Ultic Haploxeralfs	Ponderosa pine, sugar pine, white fir, giant sequoia	Csa, Mediterranean hot summer	OM1	A horizon	22	5.47	0.28	5.74	NA	19.78
Cellulose	BL031	12C	IIKFCBR02.BL031_12C.fungi	PP_CA_	California	BL	Forest Soil	Whole Community DNA	PCR	Amplicon	Pyrotag library	454 GS FLX Titanium	European Nucleotide Archive	https://www.ebi.ac.uk/ena/data/search?query=PRJEB9761	ERS803744	SAMEA3496595	ERX1051848	ERR974862	rRNA intergenic spacer analysis	ITS2	ACTAGCAGTA	TCCTCCGCTTATTGATATGC	9/16/2011	0.5	38.88	-120.64	USA	0.1	1350	11.2	55	Mesic Ultic Haploxeralfs	Ponderosa pine, sugar pine, white fir, giant sequoia	Csa, Mediterranean hot summer	OM2	O horizon	29	NA	NA	5.73	NA	NA
Cellulose	BL032	12C	IIKFCBR02.BL032_12C.fungi	PP_CA_	California	BL	Forest Soil	Whole Community DNA	PCR	Amplicon	Pyrotag library	454 GS FLX Titanium	European Nucleotide Archive	https://www.ebi.ac.uk/ena/data/search?query=PRJEB9761	ERS803746	SAMEA3496597	ERX1051850	ERR974864	rRNA intergenic spacer analysis	ITS2	ACATACGCGT	TCCTCCGCTTATTGATATGC	9/16/2011	0.5	38.88	-120.64	USA	0.3	1350	11.2	55	Mesic Ultic Haploxeralfs	Ponderosa pine, sugar pine, white fir, giant sequoia	Csa, Mediterranean hot summer	OM2	A horizon	21	5.35	0.26	5.78	NA	20.64
Cellulose	BL037	12C	IIKFCBR02.BL037_12C.fungi	PP_CA_	California	BL	Forest Soil	Whole Community DNA	PCR	Amplicon	Pyrotag library	454 GS FLX Titanium	European Nucleotide Archive	https://www.ebi.ac.uk/ena/data/search?query=PRJEB9761	ERS803748	SAMEA3496599	ERX1051852	ERR974866	rRNA intergenic spacer analysis	ITS2	AGTATACATA	TCCTCCGCTTATTGATATGC	9/16/2011	0.5	38.88	-120.64	USA	0.1	1350	11.2	55	Mesic Ultic Haploxeralfs	Ponderosa pine, sugar pine, white fir, giant sequoia	Csa, Mediterranean hot summer	OM3	O horizon	21.1	NA	NA	5.19	NA	NA
Cellulose	BL038	12C	IIKFCBR02.BL038_12C.fungi	PP_CA_	California	BL	Forest Soil	Whole Community DNA	PCR	Amplicon	Pyrotag library	454 GS FLX Titanium	European Nucleotide Archive	https://www.ebi.ac.uk/ena/data/search?query=PRJEB9761	ERS803750	SAMEA3496601	ERX1051854	ERR974868	rRNA intergenic spacer analysis	ITS2	ACTGTACAGT	TCCTCCGCTTATTGATATGC	9/16/2011	0.5	38.88	-120.64	USA	0.3	1350	11.2	55	Mesic Ultic Haploxeralfs	Ponderosa pine, sugar pine, white fir, giant sequoia	Csa, Mediterranean hot summer	OM3	A horizon	22	5.27	0.28	5.42	NA	18.87
Cellulose	BL043	12C	IIKFCBR02.BL043_12C.fungi	PP_CA_	California	BL	Forest Soil	Whole Community DNA	PCR	Amplicon	Pyrotag library	454 GS FLX Titanium	European Nucleotide Archive	https://www.ebi.ac.uk/ena/data/search?query=PRJEB9761	ERS803752	SAMEA3496603	ERX1051856	ERR974870	rRNA intergenic spacer analysis	ITS2	AGTGCTACGA	TCCTCCGCTTATTGATATGC	9/16/2011	0.5	38.88	-120.64	USA	0.1	1350	11.2	55	Mesic Ultic Haploxeralfs	Ponderosa pine, sugar pine, white fir, giant sequoia	Csa, Mediterranean hot summer	REF	O horizon	27	NA	NA	4.51	NA	NA
Cellulose	BL044	12C	IIKFCBR02.BL044_12C.fungi	PP_CA_	California	BL	Forest Soil	Whole Community DNA	PCR	Amplicon	Pyrotag library	454 GS FLX Titanium	European Nucleotide Archive	https://www.ebi.ac.uk/ena/data/search?query=PRJEB9761	ERS803754	SAMEA3496605	ERX1051858	ERR974872	rRNA intergenic spacer analysis	ITS2	AGCGTCGTCT	TCCTCCGCTTATTGATATGC	9/16/2011	0.5	38.88	-120.64	USA	0.3	1350	11.2	55	Mesic Ultic Haploxeralfs	Ponderosa pine, sugar pine, white fir, giant sequoia	Csa, Mediterranean hot summer	REF	A horizon	18	6.3	0.31	5.48	NA	20.32
Cellulose	BR049	12C	IIKFCBR02.BR049_12C.fungi	PP_CA_	California	BR	Forest Soil	Whole Community DNA	PCR	Amplicon	Pyrotag library	454 GS FLX Titanium	European Nucleotide Archive	https://www.ebi.ac.uk/ena/data/search?query=PRJEB9761	ERS803756	SAMEA3496607	ERX1051860	ERR974874	rRNA intergenic spacer analysis	ITS2	TACGCTGTCT	TCCTCCGCTTATTGATATGC	9/16/2011	0.5	39.55	-121.04	USA	0.1	1135	11.2	55	Mesic Ultic Haploxeralfs	Ponderosa pine, sugar pine, white fir, giant sequoia	Csa, Mediterranean hot summer	OM1	O horizon	54	NA	NA	5.39	NA	NA
Cellulose	BR050	12C	IIKFCBR02.BR050_12C.fungi	PP_CA_	California	BR	Forest Soil	Whole Community DNA	PCR	Amplicon	Pyrotag library	454 GS FLX Titanium	European Nucleotide Archive	https://www.ebi.ac.uk/ena/data/search?query=PRJEB9761	ERS803758	SAMEA3496609	ERX1051862	ERR974876	rRNA intergenic spacer analysis	ITS2	TCACGTACTA	TCCTCCGCTTATTGATATGC	9/16/2011	0.5	39.55	-121.04	USA	0.3	1135	11.2	55	Mesic Ultic Haploxeralfs	Ponderosa pine, sugar pine, white fir, giant sequoia	Csa, Mediterranean hot summer	OM1	A horizon	35	6.39	0.25	5.87	NA	25.8
Cellulose	BR055	12C	IIKFCBR02.BR055_12C.fungi	PP_CA_	California	BR	Forest Soil	Whole Community DNA	PCR	Amplicon	Pyrotag library	454 GS FLX Titanium	European Nucleotide Archive	https://www.ebi.ac.uk/ena/data/search?query=PRJEB9761	ERS803760	SAMEA3496611	ERX1051864	ERR974878	rRNA intergenic spacer analysis	ITS2	TCGATCACGT	TCCTCCGCTTATTGATATGC	9/16/2011	0.5	39.55	-121.04	USA	0.1	1135	11.2	55	Mesic Ultic Haploxeralfs	Ponderosa pine, sugar pine, white fir, giant sequoia	Csa, Mediterranean hot summer	OM2	O horizon	50	NA	NA	5.73	NA	NA
Cellulose	BR056	12C	IIKFCBR02.BR056_12C.fungi	PP_CA_	California	BR	Forest Soil	Whole Community DNA	PCR	Amplicon	Pyrotag library	454 GS FLX Titanium	European Nucleotide Archive	https://www.ebi.ac.uk/ena/data/search?query=PRJEB9761	ERS803762	SAMEA3496613	ERX1051866	ERR974880	rRNA intergenic spacer analysis	ITS2	TCTACGTAGC	TCCTCCGCTTATTGATATGC	9/16/2011	0.5	39.55	-121.04	USA	0.3	1135	11.2	55	Mesic Ultic Haploxeralfs	Ponderosa pine, sugar pine, white fir, giant sequoia	Csa, Mediterranean hot summer	OM2	A horizon	36	5.71	0.28	6.1	NA	20.91
Cellulose	BR061	12C	IIKFCBR02.BR061_12C.fungi	PP_CA_	California	BR	Forest Soil	Whole Community DNA	PCR	Amplicon	Pyrotag library	454 GS FLX Titanium	European Nucleotide Archive	https://www.ebi.ac.uk/ena/data/search?query=PRJEB9761	ERS803764	SAMEA3496615	ERX1051868	ERR974882	rRNA intergenic spacer analysis	ITS2	TCTAGCGACT	TCCTCCGCTTATTGATATGC	9/16/2011	0.5	39.55	-121.04	USA	0.1	1135	11.2	55	Mesic Ultic Haploxeralfs	Ponderosa pine, sugar pine, white fir, giant sequoia	Csa, Mediterranean hot summer	OM3	O horizon	40	16.4	0.61	5.81	NA	27.1
Cellulose	BR062	12C	IIKFCBR02.BR062_12C.fungi	PP_CA_	California	BR	Forest Soil	Whole Community DNA	PCR	Amplicon	Pyrotag library	454 GS FLX Titanium	European Nucleotide Archive	https://www.ebi.ac.uk/ena/data/search?query=PRJEB9761	ERS803766	SAMEA3496617	ERX1051870	ERR974884	rRNA intergenic spacer analysis	ITS2	ACGACTACAG	TCCTCCGCTTATTGATATGC	9/16/2011	0.5	39.55	-121.04	USA	0.3	1135	11.2	55	Mesic Ultic Haploxeralfs	Ponderosa pine, sugar pine, white fir, giant sequoia	Csa, Mediterranean hot summer	OM3	A horizon	34	5.2	0.27	5.52	NA	19.2
Cellulose	BR067	12C	IIKFCBR02.BR067_12C.fungi	PP_CA_	California	BR	Forest Soil	Whole Community DNA	PCR	Amplicon	Pyrotag library	454 GS FLX Titanium	European Nucleotide Archive	https://www.ebi.ac.uk/ena/data/search?query=PRJEB9761	ERS803768	SAMEA3496619	ERX1051872	ERR974886	rRNA intergenic spacer analysis	ITS2	TGACGTATGT	TCCTCCGCTTATTGATATGC	9/16/2011	0.5	39.55	-121.04	USA	0.1	1135	11.2	55	Mesic Ultic Haploxeralfs	Ponderosa pine, sugar pine, white fir, giant sequoia	Csa, Mediterranean hot summer	REF	O horizon	61	NA	NA	4.95	NA	NA
Cellulose	BR068	12C	IIKFCBR02.BR068_12C.fungi	PP_CA_	California	BR	Forest Soil	Whole Community DNA	PCR	Amplicon	Pyrotag library	454 GS FLX Titanium	European Nucleotide Archive	https://www.ebi.ac.uk/ena/data/search?query=PRJEB9761	ERS803770	SAMEA3496621	ERX1051874	ERR974888	rRNA intergenic spacer analysis	ITS2	TACGAGTATG	TCCTCCGCTTATTGATATGC	9/16/2011	0.5	39.55	-121.04	USA	0.3	1135	11.2	55	Mesic Ultic Haploxeralfs	Ponderosa pine, sugar pine, white fir, giant sequoia	Csa, Mediterranean hot summer	REF	A horizon	28	5.8	0.25	5.95	NA	23.2
Cellulose	LH001	12C	IIKFCBR02.LH001_12C.fungi	PP_CA_	California	LH	Forest Soil	Whole Community DNA	PCR	Amplicon	Pyrotag library	454 GS FLX Titanium	European Nucleotide Archive	https://www.ebi.ac.uk/ena/data/search?query=PRJEB9761	ERS803772	SAMEA3496623	ERX1051876	ERR974890	rRNA intergenic spacer analysis	ITS2	ATAGAGTACT	TCCTCCGCTTATTGATATGC	9/16/2011	0.5	39.26	-120.78	USA	0.1	1268	11.2	55	Mesic Ultic Haploxeralfs	Ponderosa pine, sugar pine, white fir, giant sequoia	Csa, Mediterranean hot summer	OM1	O horizon	14	NA	NA	3.67	NA	NA
Cellulose	LH002	12C	IIKFCBR02.LH002_12C.fungi	PP_CA_	California	LH	Forest Soil	Whole Community DNA	PCR	Amplicon	Pyrotag library	454 GS FLX Titanium	European Nucleotide Archive	https://www.ebi.ac.uk/ena/data/search?query=PRJEB9761	ERS803774	SAMEA3496625	ERX1051878	ERR974892	rRNA intergenic spacer analysis	ITS2	CTCGCGTGTC	TCCTCCGCTTATTGATATGC	9/16/2011	0.5	39.26	-120.78	USA	0.3	1268	11.2	55	Mesic Ultic Haploxeralfs	Ponderosa pine, sugar pine, white fir, giant sequoia	Csa, Mediterranean hot summer	OM1	A horizon	19	3.08	0.12	5.18	NA	24.79
Cellulose	LH007	12C	IIKFCBR02.LH007_12C.fungi	PP_CA_	California	LH	Forest Soil	Whole Community DNA	PCR	Amplicon	Pyrotag library	454 GS FLX Titanium	European Nucleotide Archive	https://www.ebi.ac.uk/ena/data/search?query=PRJEB9761	ERS803776	SAMEA3496627	ERX1051880	ERR974894	rRNA intergenic spacer analysis	ITS2	ACGCGAGTAT	TCCTCCGCTTATTGATATGC	9/16/2011	0.5	39.26	-120.78	USA	0.1	1268	11.2	55	Mesic Ultic Haploxeralfs	Ponderosa pine, sugar pine, white fir, giant sequoia	Csa, Mediterranean hot summer	OM2	O horizon	10	NA	NA	4.58	NA	NA
Cellulose	LH008	12C	IIKFCBR02.LH008_12C.fungi	PP_CA_	California	LH	Forest Soil	Whole Community DNA	PCR	Amplicon	Pyrotag library	454 GS FLX Titanium	European Nucleotide Archive	https://www.ebi.ac.uk/ena/data/search?query=PRJEB9761	ERS803778	SAMEA3496629	ERX1051882	ERR974896	rRNA intergenic spacer analysis	ITS2	TCTCTATGCG	TCCTCCGCTTATTGATATGC	9/16/2011	0.5	39.26	-120.78	USA	0.3	1268	11.2	55	Mesic Ultic Haploxeralfs	Ponderosa pine, sugar pine, white fir, giant sequoia	Csa, Mediterranean hot summer	OM2	A horizon	17.1	3.63	0.16	6	NA	22.3
Cellulose	LH014	12C	IIKFCBR02.LH014_12C.fungi	PP_CA_	California	LH	Forest Soil	Whole Community DNA	PCR	Amplicon	Pyrotag library	454 GS FLX Titanium	European Nucleotide Archive	https://www.ebi.ac.uk/ena/data/search?query=PRJEB9761	ERS803781	SAMEA3496632	ERX1051885	ERR974899	rRNA intergenic spacer analysis	ITS2	CGCAGTACGA	TCCTCCGCTTATTGATATGC	9/16/2011	0.5	39.26	-120.78	USA	0.3	1268	11.2	55	Mesic Ultic Haploxeralfs	Ponderosa pine, sugar pine, white fir, giant sequoia	Csa, Mediterranean hot summer	OM3	A horizon	17	3.3	0.15	4.99	NA	21.55
Cellulose	LH019	12C	IIKFCBR02.LH019_12C.fungi	PP_CA_	California	LH	Forest Soil	Whole Community DNA	PCR	Amplicon	Pyrotag library	454 GS FLX Titanium	European Nucleotide Archive	https://www.ebi.ac.uk/ena/data/search?query=PRJEB9761	ERS803783	SAMEA3496634	ERX1051887	ERR974901	rRNA intergenic spacer analysis	ITS2	TACACGTGAT	TCCTCCGCTTATTGATATGC	9/16/2011	0.5	39.26	-120.78	USA	0.1	1268	11.2	55	Mesic Ultic Haploxeralfs	Ponderosa pine, sugar pine, white fir, giant sequoia	Csa, Mediterranean hot summer	REF	O horizon	22	NA	NA	5.57	NA	NA
Cellulose	LH020	12C	IIKFCBR02.LH020_12C.fungi	PP_CA_	California	LH	Forest Soil	Whole Community DNA	PCR	Amplicon	Pyrotag library	454 GS FLX Titanium	European Nucleotide Archive	https://www.ebi.ac.uk/ena/data/search?query=PRJEB9761	ERS803785	SAMEA3496636	ERX1051889	ERR974903	rRNA intergenic spacer analysis	ITS2	CGAGAGATAC	TCCTCCGCTTATTGATATGC	9/16/2011	0.5	39.26	-120.78	USA	0.3	1268	11.2	55	Mesic Ultic Haploxeralfs	Ponderosa pine, sugar pine, white fir, giant sequoia	Csa, Mediterranean hot summer	REF	A horizon	20.1	4.14	0.22	6.6	NA	19.14
Cellulose	LH020;BR068;BL044	12C	C12_Metagenome_12C	PP_CA_	California	Pooled	Forest Soil	Whole Community DNA	Nextera	Shotgun Metagenome	100 bp paired read library	Illumina HiSeq 2500	European Nucleotide Archive	https://www.ebi.ac.uk/ena/data/search?query=PRJEB9761	ERS1099581	SAMEA3912447	ERX1413336	ERR1341754	WGS	NA	TAAGGCGA	NA	9/16/2011	0.5	Pooled	Pooled	USA	0.3	Pooled	11.2	55	Mesic Ultic Haploxeralfs	Ponderosa pine, sugar pine, white fir, giant sequoia	Csa, Mediterranean hot summer	REF	A horizon	Pooled	Pooled	Pooled	Pooled	Pooled	Pooled
Cellulose	BL025	13C	IIKFCBR01.BL025_13C.bact	PP_CA_	California	BL	Forest Soil	Whole Community DNA	PCR	Amplicon	Pyrotag library	454 GS FLX Titanium	European Nucleotide Archive	https://www.ebi.ac.uk/ena/data/search?query=PRJEB9761	ERS803693	SAMEA3496544	ERX1051797	ERR974811	16S rRNA	27F	TCTATACTAT	AGAGTTTGATCMTGGCTCAG	9/16/2011	0.5	38.88	-120.64	USA	0.1	1350	11.2	55	Mesic Ultic Haploxeralfs	Ponderosa pine, sugar pine, white fir, giant sequoia	Csa, Mediterranean hot summer	OM1	O horizon	18	NA	NA	4.79	NA	NA
Cellulose	BL026	13C	IIKFCBR01.BL026_13C.bact	PP_CA_	California	BL	Forest Soil	Whole Community DNA	PCR	Amplicon	Pyrotag library	454 GS FLX Titanium	European Nucleotide Archive	https://www.ebi.ac.uk/ena/data/search?query=PRJEB9761	ERS803695	SAMEA3496546	ERX1051799	ERR974813	16S rRNA	27F	ACATACGCGT	AGAGTTTGATCMTGGCTCAG	9/16/2011	0.5	38.88	-120.64	USA	0.3	1350	11.2	55	Mesic Ultic Haploxeralfs	Ponderosa pine, sugar pine, white fir, giant sequoia	Csa, Mediterranean hot summer	OM1	A horizon	22	5.47	0.28	5.74	NA	19.78
Cellulose	BL031	13C	IIKFCBR01.BL031_13C.bact	PP_CA_	California	BL	Forest Soil	Whole Community DNA	PCR	Amplicon	Pyrotag library	454 GS FLX Titanium	European Nucleotide Archive	https://www.ebi.ac.uk/ena/data/search?query=PRJEB9761	ERS803697	SAMEA3496548	ERX1051801	ERR974815	16S rRNA	27F	ACAGTATATA	AGAGTTTGATCMTGGCTCAG	9/16/2011	0.5	38.88	-120.64	USA	0.1	1350	11.2	55	Mesic Ultic Haploxeralfs	Ponderosa pine, sugar pine, white fir, giant sequoia	Csa, Mediterranean hot summer	OM2	O horizon	29	NA	NA	5.73	NA	NA
Cellulose	BL032	13C	IIKFCBR01.BL032_13C.bact	PP_CA_	California	BL	Forest Soil	Whole Community DNA	PCR	Amplicon	Pyrotag library	454 GS FLX Titanium	European Nucleotide Archive	https://www.ebi.ac.uk/ena/data/search?query=PRJEB9761	ERS803699	SAMEA3496550	ERX1051803	ERR974817	16S rRNA	27F	ACTGTACAGT	AGAGTTTGATCMTGGCTCAG	9/16/2011	0.5	38.88	-120.64	USA	0.3	1350	11.2	55	Mesic Ultic Haploxeralfs	Ponderosa pine, sugar pine, white fir, giant sequoia	Csa, Mediterranean hot summer	OM2	A horizon	21	5.35	0.26	5.78	NA	20.64
Cellulose	BL037	13C	IIKFCBR01.BL037_13C.bact	PP_CA_	California	BL	Forest Soil	Whole Community DNA	PCR	Amplicon	Pyrotag library	454 GS FLX Titanium	European Nucleotide Archive	https://www.ebi.ac.uk/ena/data/search?query=PRJEB9761	ERS803701	SAMEA3496552	ERX1051805	ERR974819	16S rRNA	27F	AGCTCACGTA	AGAGTTTGATCMTGGCTCAG	9/16/2011	0.5	38.88	-120.64	USA	0.1	1350	11.2	55	Mesic Ultic Haploxeralfs	Ponderosa pine, sugar pine, white fir, giant sequoia	Csa, Mediterranean hot summer	OM3	O horizon	21.1	NA	NA	5.19	NA	NA
Cellulose	BL038	13C	IIKFCBR01.BL038_13C.bact	PP_CA_	California	BL	Forest Soil	Whole Community DNA	PCR	Amplicon	Pyrotag library	454 GS FLX Titanium	European Nucleotide Archive	https://www.ebi.ac.uk/ena/data/search?query=PRJEB9761	ERS803703	SAMEA3496554	ERX1051807	ERR974821	16S rRNA	27F	TGTGAGTAGT	AGAGTTTGATCMTGGCTCAG	9/16/2011	0.5	38.88	-120.64	USA	0.3	1350	11.2	55	Mesic Ultic Haploxeralfs	Ponderosa pine, sugar pine, white fir, giant sequoia	Csa, Mediterranean hot summer	OM3	A horizon	22	5.27	0.28	5.42	NA	18.87
Cellulose	BL043	13C	IIKFCBR01.BL043_13C.bact	PP_CA_	California	BL	Forest Soil	Whole Community DNA	PCR	Amplicon	Pyrotag library	454 GS FLX Titanium	European Nucleotide Archive	https://www.ebi.ac.uk/ena/data/search?query=PRJEB9761	ERS803705	SAMEA3496556	ERX1051809	ERR974823	16S rRNA	27F	AGTCGAGAGA	AGAGTTTGATCMTGGCTCAG	9/16/2011	0.5	38.88	-120.64	USA	0.1	1350	11.2	55	Mesic Ultic Haploxeralfs	Ponderosa pine, sugar pine, white fir, giant sequoia	Csa, Mediterranean hot summer	REF	O horizon	27	NA	NA	4.51	NA	NA
Cellulose	BL044	13C	IIKFCBR01.BL044_13C.bact	PP_CA_	California	BL	Forest Soil	Whole Community DNA	PCR	Amplicon	Pyrotag library	454 GS FLX Titanium	European Nucleotide Archive	https://www.ebi.ac.uk/ena/data/search?query=PRJEB9761	ERS803707	SAMEA3496558	ERX1051811	ERR974825	16S rRNA	27F	AGTACGCTAT	AGAGTTTGATCMTGGCTCAG	9/16/2011	0.5	38.88	-120.64	USA	0.3	1350	11.2	55	Mesic Ultic Haploxeralfs	Ponderosa pine, sugar pine, white fir, giant sequoia	Csa, Mediterranean hot summer	REF	A horizon	18	6.3	0.31	5.48	NA	20.32
Cellulose	BR049	13C	IIKFCBR01.BR049_13C.bact	PP_CA_	California	BR	Forest Soil	Whole Community DNA	PCR	Amplicon	Pyrotag library	454 GS FLX Titanium	European Nucleotide Archive	https://www.ebi.ac.uk/ena/data/search?query=PRJEB9761	ERS803709	SAMEA3496560	ERX1051813	ERR974827	16S rRNA	27F	TACACACACT	AGAGTTTGATCMTGGCTCAG	9/16/2011	0.5	39.55	-121.04	USA	0.1	1135	11.2	55	Mesic Ultic Haploxeralfs	Ponderosa pine, sugar pine, white fir, giant sequoia	Csa, Mediterranean hot summer	OM1	O horizon	54	NA	NA	5.39	NA	NA
Cellulose	BR050	13C	IIKFCBR01.BR050_13C.bact	PP_CA_	California	BR	Forest Soil	Whole Community DNA	PCR	Amplicon	Pyrotag library	454 GS FLX Titanium	European Nucleotide Archive	https://www.ebi.ac.uk/ena/data/search?query=PRJEB9761	ERS803711	SAMEA3496562	ERX1051815	ERR974829	16S rRNA	27F	TCTACGTAGC	AGAGTTTGATCMTGGCTCAG	9/16/2011	0.5	39.55	-121.04	USA	0.3	1135	11.2	55	Mesic Ultic Haploxeralfs	Ponderosa pine, sugar pine, white fir, giant sequoia	Csa, Mediterranean hot summer	OM1	A horizon	35	6.39	0.25	5.87	NA	25.8
Cellulose	BR055	13C	IIKFCBR01.BR055_13C.bact	PP_CA_	California	BR	Forest Soil	Whole Community DNA	PCR	Amplicon	Pyrotag library	454 GS FLX Titanium	European Nucleotide Archive	https://www.ebi.ac.uk/ena/data/search?query=PRJEB9761	ERS803713	SAMEA3496564	ERX1051817	ERR974831	16S rRNA	27F	TACAGATCGT	AGAGTTTGATCMTGGCTCAG	9/16/2011	0.5	39.55	-121.04	USA	0.1	1135	11.2	55	Mesic Ultic Haploxeralfs	Ponderosa pine, sugar pine, white fir, giant sequoia	Csa, Mediterranean hot summer	OM2	O horizon	50	NA	NA	5.73	NA	NA
Cellulose	BR056	13C	IIKFCBR01.BR056_13C.bact	PP_CA_	California	BR	Forest Soil	Whole Community DNA	PCR	Amplicon	Pyrotag library	454 GS FLX Titanium	European Nucleotide Archive	https://www.ebi.ac.uk/ena/data/search?query=PRJEB9761	ERS803715	SAMEA3496566	ERX1051819	ERR974833	16S rRNA	27F	ACGACTACAG	AGAGTTTGATCMTGGCTCAG	9/16/2011	0.5	39.55	-121.04	USA	0.3	1135	11.2	55	Mesic Ultic Haploxeralfs	Ponderosa pine, sugar pine, white fir, giant sequoia	Csa, Mediterranean hot summer	OM2	A horizon	36	5.71	0.28	6.1	NA	20.91
Cellulose	BR061	13C	IIKFCBR01.BR061_13C.bact	PP_CA_	California	BR	Forest Soil	Whole Community DNA	PCR	Amplicon	Pyrotag library	454 GS FLX Titanium	European Nucleotide Archive	https://www.ebi.ac.uk/ena/data/search?query=PRJEB9761	ERS803717	SAMEA3496568	ERX1051821	ERR974835	16S rRNA	27F	TAGTGTAGAT	AGAGTTTGATCMTGGCTCAG	9/16/2011	0.5	39.55	-121.04	USA	0.1	1135	11.2	55	Mesic Ultic Haploxeralfs	Ponderosa pine, sugar pine, white fir, giant sequoia	Csa, Mediterranean hot summer	OM3	O horizon	40	16.4	0.61	5.81	NA	27.1
Cellulose	BR062	13C	IIKFCBR01.BR062_13C.bact	PP_CA_	California	BR	Forest Soil	Whole Community DNA	PCR	Amplicon	Pyrotag library	454 GS FLX Titanium	European Nucleotide Archive	https://www.ebi.ac.uk/ena/data/search?query=PRJEB9761	ERS803719	SAMEA3496570	ERX1051823	ERR974837	16S rRNA	27F	TACGAGTATG	AGAGTTTGATCMTGGCTCAG	9/16/2011	0.5	39.55	-121.04	USA	0.3	1135	11.2	55	Mesic Ultic Haploxeralfs	Ponderosa pine, sugar pine, white fir, giant sequoia	Csa, Mediterranean hot summer	OM3	A horizon	34	5.2	0.27	5.52	NA	19.2
Cellulose	BR067	13C	IIKFCBR01.BR067_13C.bact	PP_CA_	California	BR	Forest Soil	Whole Community DNA	PCR	Amplicon	Pyrotag library	454 GS FLX Titanium	European Nucleotide Archive	https://www.ebi.ac.uk/ena/data/search?query=PRJEB9761	ERS803721	SAMEA3496572	ERX1051825	ERR974839	16S rRNA	27F	TCGCACTAGT	AGAGTTTGATCMTGGCTCAG	9/16/2011	0.5	39.55	-121.04	USA	0.1	1135	11.2	55	Mesic Ultic Haploxeralfs	Ponderosa pine, sugar pine, white fir, giant sequoia	Csa, Mediterranean hot summer	REF	O horizon	61	NA	NA	4.95	NA	NA
Cellulose	BR068	13C	IIKFCBR01.BR068_13C.bact	PP_CA_	California	BR	Forest Soil	Whole Community DNA	PCR	Amplicon	Pyrotag library	454 GS FLX Titanium	European Nucleotide Archive	https://www.ebi.ac.uk/ena/data/search?query=PRJEB9761	ERS803723	SAMEA3496574	ERX1051827	ERR974841	16S rRNA	27F	TAGAGACGAG	AGAGTTTGATCMTGGCTCAG	9/16/2011	0.5	39.55	-121.04	USA	0.3	1135	11.2	55	Mesic Ultic Haploxeralfs	Ponderosa pine, sugar pine, white fir, giant sequoia	Csa, Mediterranean hot summer	REF	A horizon	28	5.8	0.25	5.95	NA	23.2
Cellulose	LH001	13C	IIKFCBR01.LH001_13C.bact	PP_CA_	California	LH	Forest Soil	Whole Community DNA	PCR	Amplicon	Pyrotag library	454 GS FLX Titanium	European Nucleotide Archive	https://www.ebi.ac.uk/ena/data/search?query=PRJEB9761	ERS803725	SAMEA3496576	ERX1051829	ERR974843	16S rRNA	27F	ATACGACGTA	AGAGTTTGATCMTGGCTCAG	9/16/2011	0.5	39.26	-120.78	USA	0.1	1268	11.2	55	Mesic Ultic Haploxeralfs	Ponderosa pine, sugar pine, white fir, giant sequoia	Csa, Mediterranean hot summer	OM1	O horizon	14	NA	NA	3.67	NA	NA
Cellulose	LH002	13C	IIKFCBR01.LH002_13C.bact	PP_CA_	California	LH	Forest Soil	Whole Community DNA	PCR	Amplicon	Pyrotag library	454 GS FLX Titanium	European Nucleotide Archive	https://www.ebi.ac.uk/ena/data/search?query=PRJEB9761	ERS803727	SAMEA3496578	ERX1051831	ERR974845	16S rRNA	27F	CGTGTCTCTA	AGAGTTTGATCMTGGCTCAG	9/16/2011	0.5	39.26	-120.78	USA	0.3	1268	11.2	55	Mesic Ultic Haploxeralfs	Ponderosa pine, sugar pine, white fir, giant sequoia	Csa, Mediterranean hot summer	OM1	A horizon	19	3.08	0.12	5.18	NA	24.79
Cellulose	LH007	13C	IIKFCBR01.LH007_13C.bact	PP_CA_	California	LH	Forest Soil	Whole Community DNA	PCR	Amplicon	Pyrotag library	454 GS FLX Titanium	European Nucleotide Archive	https://www.ebi.ac.uk/ena/data/search?query=PRJEB9761	ERS803729	SAMEA3496580	ERX1051833	ERR974847	16S rRNA	27F	ACGCGAGTAT	AGAGTTTGATCMTGGCTCAG	9/16/2011	0.5	39.26	-120.78	USA	0.1	1268	11.2	55	Mesic Ultic Haploxeralfs	Ponderosa pine, sugar pine, white fir, giant sequoia	Csa, Mediterranean hot summer	OM2	O horizon	10	NA	NA	4.58	NA	NA
Cellulose	LH008	13C	IIKFCBR01.LH008_13C.bact	PP_CA_	California	LH	Forest Soil	Whole Community DNA	PCR	Amplicon	Pyrotag library	454 GS FLX Titanium	European Nucleotide Archive	https://www.ebi.ac.uk/ena/data/search?query=PRJEB9761	ERS803731	SAMEA3496582	ERX1051835	ERR974849	16S rRNA	27F	CGATCGTATA	AGAGTTTGATCMTGGCTCAG	9/16/2011	0.5	39.26	-120.78	USA	0.3	1268	11.2	55	Mesic Ultic Haploxeralfs	Ponderosa pine, sugar pine, white fir, giant sequoia	Csa, Mediterranean hot summer	OM2	A horizon	17.1	3.63	0.16	6	NA	22.3
Cellulose	LH013	13C	IIKFCBR01.LH013_13C.bact	PP_CA_	California	LH	Forest Soil	Whole Community DNA	PCR	Amplicon	Pyrotag library	454 GS FLX Titanium	European Nucleotide Archive	https://www.ebi.ac.uk/ena/data/search?query=PRJEB9761	ERS803733	SAMEA3496584	ERX1051837	ERR974851	16S rRNA	27F	ACTAGCAGTA	AGAGTTTGATCMTGGCTCAG	9/16/2011	0.5	39.26	-120.78	USA	0.1	1268	11.2	55	Mesic Ultic Haploxeralfs	Ponderosa pine, sugar pine, white fir, giant sequoia	Csa, Mediterranean hot summer	OM3	O horizon	14.1	36.4	1.07	5.08	NA	34.1
Cellulose	LH014	13C	IIKFCBR01.LH014_13C.bact	PP_CA_	California	LH	Forest Soil	Whole Community DNA	PCR	Amplicon	Pyrotag library	454 GS FLX Titanium	European Nucleotide Archive	https://www.ebi.ac.uk/ena/data/search?query=PRJEB9761	ERS803735	SAMEA3496586	ERX1051839	ERR974853	16S rRNA	27F	TGATACGTCT	AGAGTTTGATCMTGGCTCAG	9/16/2011	0.5	39.26	-120.78	USA	0.3	1268	11.2	55	Mesic Ultic Haploxeralfs	Ponderosa pine, sugar pine, white fir, giant sequoia	Csa, Mediterranean hot summer	OM3	A horizon	17	3.3	0.15	4.99	NA	21.55
Cellulose	LH019	13C	IIKFCBR01.LH019_13C.bact	PP_CA_	California	LH	Forest Soil	Whole Community DNA	PCR	Amplicon	Pyrotag library	454 GS FLX Titanium	European Nucleotide Archive	https://www.ebi.ac.uk/ena/data/search?query=PRJEB9761	ERS803737	SAMEA3496588	ERX1051841	ERR974855	16S rRNA	27F	CAGTAGACGT	AGAGTTTGATCMTGGCTCAG	9/16/2011	0.5	39.26	-120.78	USA	0.1	1268	11.2	55	Mesic Ultic Haploxeralfs	Ponderosa pine, sugar pine, white fir, giant sequoia	Csa, Mediterranean hot summer	REF	O horizon	22	NA	NA	5.57	NA	NA
Cellulose	LH020	13C	IIKFCBR01.LH020_13C.bact	PP_CA_	California	LH	Forest Soil	Whole Community DNA	PCR	Amplicon	Pyrotag library	454 GS FLX Titanium	European Nucleotide Archive	https://www.ebi.ac.uk/ena/data/search?query=PRJEB9761	ERS803739	SAMEA3496590	ERX1051843	ERR974857	16S rRNA	27F	CATAGTAGTG	AGAGTTTGATCMTGGCTCAG	9/16/2011	0.5	39.26	-120.78	USA	0.3	1268	11.2	55	Mesic Ultic Haploxeralfs	Ponderosa pine, sugar pine, white fir, giant sequoia	Csa, Mediterranean hot summer	REF	A horizon	20.1	4.14	0.22	6.6	NA	19.14
Cellulose	BL025	13C	IIKFCBR02.BL025_13C.fungi	PP_CA_	California	BL	Forest Soil	Whole Community DNA	PCR	Amplicon	Pyrotag library	454 GS FLX Titanium	European Nucleotide Archive	https://www.ebi.ac.uk/ena/data/search?query=PRJEB9761	ERS803741	SAMEA3496592	ERX1051845	ERR974859	rRNA intergenic spacer analysis	ITS2	TGTGAGTAGT	TCCTCCGCTTATTGATATGC	9/16/2011	0.5	38.88	-120.64	USA	0.1	1350	11.2	55	Mesic Ultic Haploxeralfs	Ponderosa pine, sugar pine, white fir, giant sequoia	Csa, Mediterranean hot summer	OM1	O horizon	18	NA	NA	4.79	NA	NA
Cellulose	BL026	13C	IIKFCBR02.BL026_13C.fungi	PP_CA_	California	BL	Forest Soil	Whole Community DNA	PCR	Amplicon	Pyrotag library	454 GS FLX Titanium	European Nucleotide Archive	https://www.ebi.ac.uk/ena/data/search?query=PRJEB9761	ERS803743	SAMEA3496594	ERX1051847	ERR974861	rRNA intergenic spacer analysis	ITS2	TACTCTCGTG	TCCTCCGCTTATTGATATGC	9/16/2011	0.5	38.88	-120.64	USA	0.3	1350	11.2	55	Mesic Ultic Haploxeralfs	Ponderosa pine, sugar pine, white fir, giant sequoia	Csa, Mediterranean hot summer	OM1	A horizon	22	5.47	0.28	5.74	NA	19.78
Cellulose	BL031	13C	IIKFCBR02.BL031_13C.fungi	PP_CA_	California	BL	Forest Soil	Whole Community DNA	PCR	Amplicon	Pyrotag library	454 GS FLX Titanium	European Nucleotide Archive	https://www.ebi.ac.uk/ena/data/search?query=PRJEB9761	ERS803745	SAMEA3496596	ERX1051849	ERR974863	rRNA intergenic spacer analysis	ITS2	ACGCGATCGA	TCCTCCGCTTATTGATATGC	9/16/2011	0.5	38.88	-120.64	USA	0.1	1350	11.2	55	Mesic Ultic Haploxeralfs	Ponderosa pine, sugar pine, white fir, giant sequoia	Csa, Mediterranean hot summer	OM2	O horizon	29	NA	NA	5.73	NA	NA
Cellulose	BL032	13C	IIKFCBR02.BL032_13C.fungi	PP_CA_	California	BL	Forest Soil	Whole Community DNA	PCR	Amplicon	Pyrotag library	454 GS FLX Titanium	European Nucleotide Archive	https://www.ebi.ac.uk/ena/data/search?query=PRJEB9761	ERS803747	SAMEA3496598	ERX1051851	ERR974865	rRNA intergenic spacer analysis	ITS2	TCGTCGCTCG	TCCTCCGCTTATTGATATGC	9/16/2011	0.5	38.88	-120.64	USA	0.3	1350	11.2	55	Mesic Ultic Haploxeralfs	Ponderosa pine, sugar pine, white fir, giant sequoia	Csa, Mediterranean hot summer	OM2	A horizon	21	5.35	0.26	5.78	NA	20.64
Cellulose	BL037	13C	IIKFCBR02.BL037_13C.fungi	PP_CA_	California	BL	Forest Soil	Whole Community DNA	PCR	Amplicon	Pyrotag library	454 GS FLX Titanium	European Nucleotide Archive	https://www.ebi.ac.uk/ena/data/search?query=PRJEB9761	ERS803749	SAMEA3496600	ERX1051853	ERR974867	rRNA intergenic spacer analysis	ITS2	AGCTCACGTA	TCCTCCGCTTATTGATATGC	9/16/2011	0.5	38.88	-120.64	USA	0.1	1350	11.2	55	Mesic Ultic Haploxeralfs	Ponderosa pine, sugar pine, white fir, giant sequoia	Csa, Mediterranean hot summer	OM3	O horizon	21.1	NA	NA	5.19	NA	NA
Cellulose	BL038	13C	IIKFCBR02.BL038_13C.fungi	PP_CA_	California	BL	Forest Soil	Whole Community DNA	PCR	Amplicon	Pyrotag library	454 GS FLX Titanium	European Nucleotide Archive	https://www.ebi.ac.uk/ena/data/search?query=PRJEB9761	ERS803751	SAMEA3496602	ERX1051855	ERR974869	rRNA intergenic spacer analysis	ITS2	ACTACTATGT	TCCTCCGCTTATTGATATGC	9/16/2011	0.5	38.88	-120.64	USA	0.3	1350	11.2	55	Mesic Ultic Haploxeralfs	Ponderosa pine, sugar pine, white fir, giant sequoia	Csa, Mediterranean hot summer	OM3	A horizon	22	5.27	0.28	5.42	NA	18.87
Cellulose	BL043	13C	IIKFCBR02.BL043_13C.fungi	PP_CA_	California	BL	Forest Soil	Whole Community DNA	PCR	Amplicon	Pyrotag library	454 GS FLX Titanium	European Nucleotide Archive	https://www.ebi.ac.uk/ena/data/search?query=PRJEB9761	ERS803753	SAMEA3496604	ERX1051857	ERR974871	rRNA intergenic spacer analysis	ITS2	AGTCGAGAGA	TCCTCCGCTTATTGATATGC	9/16/2011	0.5	38.88	-120.64	USA	0.1	1350	11.2	55	Mesic Ultic Haploxeralfs	Ponderosa pine, sugar pine, white fir, giant sequoia	Csa, Mediterranean hot summer	REF	O horizon	27	NA	NA	4.51	NA	NA
Cellulose	BL044	13C	IIKFCBR02.BL044_13C.fungi	PP_CA_	California	BL	Forest Soil	Whole Community DNA	PCR	Amplicon	Pyrotag library	454 GS FLX Titanium	European Nucleotide Archive	https://www.ebi.ac.uk/ena/data/search?query=PRJEB9761	ERS803755	SAMEA3496606	ERX1051859	ERR974873	rRNA intergenic spacer analysis	ITS2	AGACTATACT	TCCTCCGCTTATTGATATGC	9/16/2011	0.5	38.88	-120.64	USA	0.3	1350	11.2	55	Mesic Ultic Haploxeralfs	Ponderosa pine, sugar pine, white fir, giant sequoia	Csa, Mediterranean hot summer	REF	A horizon	18	6.3	0.31	5.48	NA	20.32
Cellulose	BR049	13C	IIKFCBR02.BR049_13C.fungi	PP_CA_	California	BR	Forest Soil	Whole Community DNA	PCR	Amplicon	Pyrotag library	454 GS FLX Titanium	European Nucleotide Archive	https://www.ebi.ac.uk/ena/data/search?query=PRJEB9761	ERS803757	SAMEA3496608	ERX1051861	ERR974875	rRNA intergenic spacer analysis	ITS2	TACAGATCGT	TCCTCCGCTTATTGATATGC	9/16/2011	0.5	39.55	-121.04	USA	0.1	1135	11.2	55	Mesic Ultic Haploxeralfs	Ponderosa pine, sugar pine, white fir, giant sequoia	Csa, Mediterranean hot summer	OM1	O horizon	54	NA	NA	5.39	NA	NA
Cellulose	BR050	13C	IIKFCBR02.BR050_13C.fungi	PP_CA_	California	BR	Forest Soil	Whole Community DNA	PCR	Amplicon	Pyrotag library	454 GS FLX Titanium	European Nucleotide Archive	https://www.ebi.ac.uk/ena/data/search?query=PRJEB9761	ERS803759	SAMEA3496610	ERX1051863	ERR974877	rRNA intergenic spacer analysis	ITS2	ATACGACGTA	TCCTCCGCTTATTGATATGC	9/16/2011	0.5	39.55	-121.04	USA	0.3	1135	11.2	55	Mesic Ultic Haploxeralfs	Ponderosa pine, sugar pine, white fir, giant sequoia	Csa, Mediterranean hot summer	OM1	A horizon	35	6.39	0.25	5.87	NA	25.8
Cellulose	BR055	13C	IIKFCBR02.BR055_13C.fungi	PP_CA_	California	BR	Forest Soil	Whole Community DNA	PCR	Amplicon	Pyrotag library	454 GS FLX Titanium	European Nucleotide Archive	https://www.ebi.ac.uk/ena/data/search?query=PRJEB9761	ERS803761	SAMEA3496612	ERX1051865	ERR974879	rRNA intergenic spacer analysis	ITS2	TAGTGTAGAT	TCCTCCGCTTATTGATATGC	9/16/2011	0.5	39.55	-121.04	USA	0.1	1135	11.2	55	Mesic Ultic Haploxeralfs	Ponderosa pine, sugar pine, white fir, giant sequoia	Csa, Mediterranean hot summer	OM2	O horizon	50	NA	NA	5.73	NA	NA
Cellulose	BR056	13C	IIKFCBR02.BR056_13C.fungi	PP_CA_	California	BR	Forest Soil	Whole Community DNA	PCR	Amplicon	Pyrotag library	454 GS FLX Titanium	European Nucleotide Archive	https://www.ebi.ac.uk/ena/data/search?query=PRJEB9761	ERS803763	SAMEA3496614	ERX1051867	ERR974881	rRNA intergenic spacer analysis	ITS2	CGTCTAGTAC	TCCTCCGCTTATTGATATGC	9/16/2011	0.5	39.55	-121.04	USA	0.3	1135	11.2	55	Mesic Ultic Haploxeralfs	Ponderosa pine, sugar pine, white fir, giant sequoia	Csa, Mediterranean hot summer	OM2	A horizon	36	5.71	0.28	6.1	NA	20.91
Cellulose	BR061	13C	IIKFCBR02.BR061_13C.fungi	PP_CA_	California	BR	Forest Soil	Whole Community DNA	PCR	Amplicon	Pyrotag library	454 GS FLX Titanium	European Nucleotide Archive	https://www.ebi.ac.uk/ena/data/search?query=PRJEB9761	ERS803765	SAMEA3496616	ERX1051869	ERR974883	rRNA intergenic spacer analysis	ITS2	CGCGTATACA	TCCTCCGCTTATTGATATGC	9/16/2011	0.5	39.55	-121.04	USA	0.1	1135	11.2	55	Mesic Ultic Haploxeralfs	Ponderosa pine, sugar pine, white fir, giant sequoia	Csa, Mediterranean hot summer	OM3	O horizon	40	16.4	0.61	5.81	NA	27.1
Cellulose	BR062	13C	IIKFCBR02.BR062_13C.fungi	PP_CA_	California	BR	Forest Soil	Whole Community DNA	PCR	Amplicon	Pyrotag library	454 GS FLX Titanium	European Nucleotide Archive	https://www.ebi.ac.uk/ena/data/search?query=PRJEB9761	ERS803767	SAMEA3496618	ERX1051871	ERR974885	rRNA intergenic spacer analysis	ITS2	TGTACTACTC	TCCTCCGCTTATTGATATGC	9/16/2011	0.5	39.55	-121.04	USA	0.3	1135	11.2	55	Mesic Ultic Haploxeralfs	Ponderosa pine, sugar pine, white fir, giant sequoia	Csa, Mediterranean hot summer	OM3	A horizon	34	5.2	0.27	5.52	NA	19.2
Cellulose	BR067	13C	IIKFCBR02.BR067_13C.fungi	PP_CA_	California	BR	Forest Soil	Whole Community DNA	PCR	Amplicon	Pyrotag library	454 GS FLX Titanium	European Nucleotide Archive	https://www.ebi.ac.uk/ena/data/search?query=PRJEB9761	ERS803769	SAMEA3496620	ERX1051873	ERR974887	rRNA intergenic spacer analysis	ITS2	TCTATACTAT	TCCTCCGCTTATTGATATGC	9/16/2011	0.5	39.55	-121.04	USA	0.1	1135	11.2	55	Mesic Ultic Haploxeralfs	Ponderosa pine, sugar pine, white fir, giant sequoia	Csa, Mediterranean hot summer	REF	O horizon	61	NA	NA	4.95	NA	NA
Cellulose	BR068	13C	IIKFCBR02.BR068_13C.fungi	PP_CA_	California	BR	Forest Soil	Whole Community DNA	PCR	Amplicon	Pyrotag library	454 GS FLX Titanium	European Nucleotide Archive	https://www.ebi.ac.uk/ena/data/search?query=PRJEB9761	ERS803771	SAMEA3496622	ERX1051875	ERR974889	rRNA intergenic spacer analysis	ITS2	CGTAGACTAG	TCCTCCGCTTATTGATATGC	9/16/2011	0.5	39.55	-121.04	USA	0.3	1135	11.2	55	Mesic Ultic Haploxeralfs	Ponderosa pine, sugar pine, white fir, giant sequoia	Csa, Mediterranean hot summer	REF	A horizon	28	5.8	0.25	5.95	NA	23.2
Cellulose	LH001	13C	IIKFCBR02.LH001_13C.fungi	PP_CA_	California	LH	Forest Soil	Whole Community DNA	PCR	Amplicon	Pyrotag library	454 GS FLX Titanium	European Nucleotide Archive	https://www.ebi.ac.uk/ena/data/search?query=PRJEB9761	ERS803773	SAMEA3496624	ERX1051877	ERR974891	rRNA intergenic spacer analysis	ITS2	AGTACGCTAT	TCCTCCGCTTATTGATATGC	9/16/2011	0.5	39.26	-120.78	USA	0.1	1268	11.2	55	Mesic Ultic Haploxeralfs	Ponderosa pine, sugar pine, white fir, giant sequoia	Csa, Mediterranean hot summer	OM1	O horizon	14	NA	NA	3.67	NA	NA
Cellulose	LH002	13C	IIKFCBR02.LH002_13C.fungi	PP_CA_	California	LH	Forest Soil	Whole Community DNA	PCR	Amplicon	Pyrotag library	454 GS FLX Titanium	European Nucleotide Archive	https://www.ebi.ac.uk/ena/data/search?query=PRJEB9761	ERS803775	SAMEA3496626	ERX1051879	ERR974893	rRNA intergenic spacer analysis	ITS2	CGTGTCTCTA	TCCTCCGCTTATTGATATGC	9/16/2011	0.5	39.26	-120.78	USA	0.3	1268	11.2	55	Mesic Ultic Haploxeralfs	Ponderosa pine, sugar pine, white fir, giant sequoia	Csa, Mediterranean hot summer	OM1	A horizon	19	3.08	0.12	5.18	NA	24.79
Cellulose	LH007	13C	IIKFCBR02.LH007_13C.fungi	PP_CA_	California	LH	Forest Soil	Whole Community DNA	PCR	Amplicon	Pyrotag library	454 GS FLX Titanium	European Nucleotide Archive	https://www.ebi.ac.uk/ena/data/search?query=PRJEB9761	ERS803777	SAMEA3496628	ERX1051881	ERR974895	rRNA intergenic spacer analysis	ITS2	CACGCTACGT	TCCTCCGCTTATTGATATGC	9/16/2011	0.5	39.26	-120.78	USA	0.1	1268	11.2	55	Mesic Ultic Haploxeralfs	Ponderosa pine, sugar pine, white fir, giant sequoia	Csa, Mediterranean hot summer	OM2	O horizon	10	NA	NA	4.58	NA	NA
Cellulose	LH008	13C	IIKFCBR02.LH008_13C.fungi	PP_CA_	California	LH	Forest Soil	Whole Community DNA	PCR	Amplicon	Pyrotag library	454 GS FLX Titanium	European Nucleotide Archive	https://www.ebi.ac.uk/ena/data/search?query=PRJEB9761	ERS803779	SAMEA3496630	ERX1051883	ERR974897	rRNA intergenic spacer analysis	ITS2	CGATCGTATA	TCCTCCGCTTATTGATATGC	9/16/2011	0.5	39.26	-120.78	USA	0.3	1268	11.2	55	Mesic Ultic Haploxeralfs	Ponderosa pine, sugar pine, white fir, giant sequoia	Csa, Mediterranean hot summer	OM2	A horizon	17.1	3.63	0.16	6	NA	22.3
Cellulose	LH013	13C	IIKFCBR02.LH013_13C.fungi	PP_CA_	California	LH	Forest Soil	Whole Community DNA	PCR	Amplicon	Pyrotag library	454 GS FLX Titanium	European Nucleotide Archive	https://www.ebi.ac.uk/ena/data/search?query=PRJEB9761	ERS803780	SAMEA3496631	ERX1051884	ERR974898	rRNA intergenic spacer analysis	ITS2	CAGTAGACGT	TCCTCCGCTTATTGATATGC	9/16/2011	0.5	39.26	-120.78	USA	0.1	1268	11.2	55	Mesic Ultic Haploxeralfs	Ponderosa pine, sugar pine, white fir, giant sequoia	Csa, Mediterranean hot summer	OM3	O horizon	14.1	36.4	1.07	5.08	NA	34.09
Cellulose	LH014	13C	IIKFCBR02.LH014_13C.fungi	PP_CA_	California	LH	Forest Soil	Whole Community DNA	PCR	Amplicon	Pyrotag library	454 GS FLX Titanium	European Nucleotide Archive	https://www.ebi.ac.uk/ena/data/search?query=PRJEB9761	ERS803782	SAMEA3496633	ERX1051886	ERR974900	rRNA intergenic spacer analysis	ITS2	TGATACGTCT	TCCTCCGCTTATTGATATGC	9/16/2011	0.5	39.26	-120.78	USA	0.3	1268	11.2	55	Mesic Ultic Haploxeralfs	Ponderosa pine, sugar pine, white fir, giant sequoia	Csa, Mediterranean hot summer	OM3	A horizon	17	3.3	0.15	4.99	NA	21.55
Cellulose	LH019	13C	IIKFCBR02.LH019_13C.fungi	PP_CA_	California	LH	Forest Soil	Whole Community DNA	PCR	Amplicon	Pyrotag library	454 GS FLX Titanium	European Nucleotide Archive	https://www.ebi.ac.uk/ena/data/search?query=PRJEB9761	ERS803784	SAMEA3496635	ERX1051888	ERR974902	rRNA intergenic spacer analysis	ITS2	TACACACACT	TCCTCCGCTTATTGATATGC	9/16/2011	0.5	39.26	-120.78	USA	0.1	1268	11.2	55	Mesic Ultic Haploxeralfs	Ponderosa pine, sugar pine, white fir, giant sequoia	Csa, Mediterranean hot summer	REF	O horizon	22	NA	NA	5.57	NA	NA
Cellulose	LH020	13C	IIKFCBR02.LH020_13C.fungi	PP_CA_	California	LH	Forest Soil	Whole Community DNA	PCR	Amplicon	Pyrotag library	454 GS FLX Titanium	European Nucleotide Archive	https://www.ebi.ac.uk/ena/data/search?query=PRJEB9761	ERS803786	SAMEA3496637	ERX1051890	ERR974904	rRNA intergenic spacer analysis	ITS2	CATAGTAGTG	TCCTCCGCTTATTGATATGC	9/16/2011	0.5	39.26	-120.78	USA	0.3	1268	11.2	55	Mesic Ultic Haploxeralfs	Ponderosa pine, sugar pine, white fir, giant sequoia	Csa, Mediterranean hot summer	REF	A horizon	20.1	4.14	0.22	6.6	NA	19.14
Cellulose	BL026;BR050;LH002	13C	OM1_Metagenome_13C	PP_CA_	California	Pooled	Forest Soil	Whole Community DNA	Nextera	Shotgun Metagenome	100 bp paired read library	Illumina HiSeq 2500	European Nucleotide Archive	https://www.ebi.ac.uk/ena/data/search?query=PRJEB9761	ERS1099582	SAMEA3912448	ERX1413337	ERR1341755	WGS	NA	CGTACTAG	NA	9/16/2011	0.5	Pooled	Pooled	USA	0.3	Pooled	11.2	55	Mesic Ultic Haploxeralfs	Ponderosa pine, sugar pine, white fir, giant sequoia	Csa, Mediterranean hot summer	OM1	A horizon	Pooled	Pooled	Pooled	Pooled	Pooled	Pooled
Cellulose	BL038;BR062;LH014	13C	OM3_Metagenome_13C	PP_CA_	California	Pooled	Forest Soil	Whole Community DNA	Nextera	Shotgun Metagenome	100 bp paired read library	Illumina HiSeq 2500	European Nucleotide Archive	https://www.ebi.ac.uk/ena/data/search?query=PRJEB9761	ERS1099583	SAMEA3912449	ERX1413338	ERR1341756	WGS	NA	AGGCAGAA	NA	9/16/2011	0.5	Pooled	Pooled	USA	0.3	Pooled	11.2	55	Mesic Ultic Haploxeralfs	Ponderosa pine, sugar pine, white fir, giant sequoia	Csa, Mediterranean hot summer	OM3	A horizon	Pooled	Pooled	Pooled	Pooled	Pooled	Pooled
Cellulose	LH020;BR068;BL044	13C	REF_Metagenome_13C	PP_CA_	California	Pooled	Forest Soil	Whole Community DNA	Nextera	Shotgun Metagenome	100 bp paired read library	Illumina HiSeq 2500	European Nucleotide Archive	https://www.ebi.ac.uk/ena/data/search?query=PRJEB9761	ERS1099584	SAMEA3912450	ERX1413339	ERR1341757	WGS	NA	TCCTGAGC	NA	9/16/2011	0.5	Pooled	Pooled	USA	0.3	Pooled	11.2	55	Mesic Ultic Haploxeralfs	Ponderosa pine, sugar pine, white fir, giant sequoia	Csa, Mediterranean hot summer	REF	A horizon	Pooled	Pooled	Pooled	Pooled	Pooled	Pooled
Hemicellulose	BP479	12C	12C-OM1-Organic-BP-C1-1	IDF_BC_	British Columbia	BP	Forest Soil	Whole Community DNA	PCR	Amplicon	Pyrotag library	454 GS FLX Titanium	European Nucleotide Archive	https://www.ebi.ac.uk/ena/data/search?query=PRJEB9181	ERS713515	SAMEA3360063	ERX945177	ERR865549	16S rRNA	27F	TACAGATCGT	AGAGTTTGATCMTGGCTCAG	6/22/2010	0.5	50.93	-120.28	CANADA	0.1	1180	2.5	300	Brunisolic Gray Luvisol	Douglas fir, Lodgepole pine	Dfb, Humid Continental warm summer	OM1	O horizon	63.7	38.66	1.10	5.60	NA	35.84
Hemicellulose	BP482	12C	12C-OM1-Mineral-BP-C2-1	IDF_BC_	British Columbia	BP	Forest Soil	Whole Community DNA	PCR	Amplicon	Pyrotag library	454 GS FLX Titanium	European Nucleotide Archive	https://www.ebi.ac.uk/ena/data/search?query=PRJEB9181	ERS713517	SAMEA3360065	ERX945174	ERR865546	16S rRNA	27F	TGATACGTCT	AGAGTTTGATCMTGGCTCAG	6/22/2010	0.5	50.93	-120.28	CANADA	0.3	1180	2.5	300	Brunisolic Gray Luvisol	Douglas fir, Lodgepole pine	Dfb, Humid Continental warm summer	OM1	A horizon	25.5	2.98	0.11	5.35	NA	27.44
Hemicellulose	BP515	12C	12C-OM0-Organic-BP-C3-1	IDF_BC_	British Columbia	BP	Forest Soil	Whole Community DNA	PCR	Amplicon	Pyrotag library	454 GS FLX Titanium	European Nucleotide Archive	https://www.ebi.ac.uk/ena/data/search?query=PRJEB9181	ERS713519	SAMEA3360067	ERX945171	ERR865543	16S rRNA	27F	CATAGTAGTG	AGAGTTTGATCMTGGCTCAG	6/22/2010	0.5	50.93	-120.28	CANADA	0.1	1180	2.5	300	Brunisolic Gray Luvisol	Douglas fir, Lodgepole pine	Dfb, Humid Continental warm summer	REF	O horizon	61.0	44.38	1.25	5.35	NA	37.11
Hemicellulose	BP518	12C	12C-OM0-Mineral-BP-C4-1	IDF_BC_	British Columbia	BP	Forest Soil	Whole Community DNA	PCR	Amplicon	Pyrotag library	454 GS FLX Titanium	European Nucleotide Archive	https://www.ebi.ac.uk/ena/data/search?query=PRJEB9181	ERS713521	SAMEA3360069	ERX945169	ERR865541	16S rRNA	27F	ATACGACGTA	AGAGTTTGATCMTGGCTCAG	6/22/2010	0.5	50.93	-120.28	CANADA	0.3	1180	2.5	300	Brunisolic Gray Luvisol	Douglas fir, Lodgepole pine	Dfb, Humid Continental warm summer	REF	A horizon	19.0	2.42	0.10	5.58	NA	23.78
Hemicellulose	DC578	12C	12C-OM0-Mineral-DC-C4-1	IDF_BC_	British Columbia	DC	Forest Soil	Whole Community DNA	PCR	Amplicon	Pyrotag library	454 GS FLX Titanium	European Nucleotide Archive	https://www.ebi.ac.uk/ena/data/search?query=PRJEB9181	ERS713526	SAMEA3360074	ERX945170	ERR865542	16S rRNA	27F	ATAGAGTACT	AGAGTTTGATCMTGGCTCAG	6/25/2010	0.5	50.85	-120.42	CANADA	0.3	1150	2.5	300	Brunisolic Gray Luvisol	Douglas fir, Subalpine fir, Lodgepole pine	Dfb, Humid Continental warm summer	REF	A horizon	19.0	2.34	0.10	5.17	NA	23.60
Hemicellulose	OC407	12C	12C-OM1-Organic-OC-C1-1	IDF_BC_	British Columbia	OC	Forest Soil	Whole Community DNA	PCR	Amplicon	Pyrotag library	454 GS FLX Titanium	European Nucleotide Archive	https://www.ebi.ac.uk/ena/data/search?query=PRJEB9181	ERS713508	SAMEA3360056	ERX945178	ERR865550	16S rRNA	27F	ACGAGTGCGT	AGAGTTTGATCMTGGCTCAG	6/26/2010	0.5	50.88	-120.35	CANADA	0.1	1075	2.5	300	Brunisolic Gray Luvisol	Douglas fir	Dfb, Humid Continental warm summer	OM1	O horizon	50.0	33.83	1.41	5.50	NA	24.05
Hemicellulose	OC410	12C	12C-OM1-Mineral-OC-C2-1	IDF_BC_	British Columbia	OC	Forest Soil	Whole Community DNA	PCR	Amplicon	Pyrotag library	454 GS FLX Titanium	European Nucleotide Archive	https://www.ebi.ac.uk/ena/data/search?query=PRJEB9181	ERS713510	SAMEA3360058	ERX945175	ERR865547	16S rRNA	27F	AGACGCACTC	AGAGTTTGATCMTGGCTCAG	6/26/2010	0.5	50.88	-120.35	CANADA	0.3	1075	2.5	300	Brunisolic Gray Luvisol	Douglas fir	Dfb, Humid Continental warm summer	OM1	A horizon	23.0	1.79	0.11	5.62	NA	15.92
Hemicellulose	OC455	12C	12C-OM0-Organic-OC-C3-1	IDF_BC_	British Columbia	OC	Forest Soil	Whole Community DNA	PCR	Amplicon	Pyrotag library	454 GS FLX Titanium	European Nucleotide Archive	https://www.ebi.ac.uk/ena/data/search?query=PRJEB9181	ERS713512	SAMEA3360060	ERX945172	ERR865544	16S rRNA	27F	ATCAGACACG	AGAGTTTGATCMTGGCTCAG	6/26/2010	0.5	50.88	-120.35	CANADA	0.1	1075	2.5	300	Brunisolic Gray Luvisol	Douglas fir	Dfb, Humid Continental warm summer	REF	O horizon	37.0	44.18	1.39	5.41	NA	31.71
Hemicellulose	BP479	13C	13C-OM1-Organic-BP-C1-1	IDF_BC_	British Columbia	BP	Forest Soil	Whole Community DNA	PCR	Amplicon	Pyrotag library	454 GS FLX Titanium	European Nucleotide Archive	https://www.ebi.ac.uk/ena/data/search?query=PRJEB9181	ERS713516	SAMEA3360064	ERX945191	ERR865563	16S rRNA	27F	TCTCTATGCG	AGAGTTTGATCMTGGCTCAG	6/22/2010	0.5	50.93	-120.28	CANADA	0.1	1180	2.5	300	Brunisolic Gray Luvisol	Douglas fir, Lodgepole pine	Dfb, Humid Continental warm summer	OM1	O horizon	63.7	38.66	1.10	5.60	NA	35.84
Hemicellulose	BP482	13C	13C-OM1-Mineral-BP-C2-1	IDF_BC_	British Columbia	BP	Forest Soil	Whole Community DNA	PCR	Amplicon	Pyrotag library	454 GS FLX Titanium	European Nucleotide Archive	https://www.ebi.ac.uk/ena/data/search?query=PRJEB9181	ERS713518	SAMEA3360066	ERX945186	ERR865558	16S rRNA	27F	ACTAGCAGTA	AGAGTTTGATCMTGGCTCAG	6/22/2010	0.5	50.93	-120.28	CANADA	0.3	1180	2.5	300	Brunisolic Gray Luvisol	Douglas fir, Lodgepole pine	Dfb, Humid Continental warm summer	OM1	A horizon	25.5	2.98	0.11	5.35	NA	27.44
Hemicellulose	BP515	13C	13C-OM0-Organic-BP-C3-1	IDF_BC_	British Columbia	BP	Forest Soil	Whole Community DNA	PCR	Amplicon	Pyrotag library	454 GS FLX Titanium	European Nucleotide Archive	https://www.ebi.ac.uk/ena/data/search?query=PRJEB9181	ERS713520	SAMEA3360068	ERX945182	ERR865554	16S rRNA	27F	CGAGAGATAC	AGAGTTTGATCMTGGCTCAG	6/22/2010	0.5	50.93	-120.28	CANADA	0.1	1180	2.5	300	Brunisolic Gray Luvisol	Douglas fir, Lodgepole pine	Dfb, Humid Continental warm summer	REF	O horizon	61.0	44.38	1.25	5.35	NA	37.11
Hemicellulose	BP515	13C	13C-OM0-Organic-BP-C3-1	IDF_BC_	British Columbia	BP	Forest Soil	Whole Community DNA	PCR	Amplicon	Pyrotag library	454 GS FLX Titanium	European Nucleotide Archive	PRJEB9182	ERS713540	SAMEA3360088	ERX945201	ERR865573	rRNA intergenic spacer analysis	ITS2	CGTACAGTCA	TCCTCCGCTTATTGATATGC	6/22/2010	0.5	50.93	-120.28	CANADA	0.1	1180	2.5	300	Brunisolic Gray Luvisol	Douglas fir, Lodgepole pine	Dfb, Humid Continental warm summer	REF	O horizon	61.0	44.38	1.25	5.35	NA	37.11
Hemicellulose	BP518	13C	13C-OM0-Mineral-BP-C4-1	IDF_BC_	British Columbia	BP	Forest Soil	Whole Community DNA	PCR	Amplicon	Pyrotag library	454 GS FLX Titanium	European Nucleotide Archive	https://www.ebi.ac.uk/ena/data/search?query=PRJEB9181	ERS713522	SAMEA3360070	ERX945179	ERR865551	16S rRNA	27F	TCACGTACTA	AGAGTTTGATCMTGGCTCAG	6/22/2010	0.5	50.93	-120.28	CANADA	0.3	1180	2.5	300	Brunisolic Gray Luvisol	Douglas fir, Lodgepole pine	Dfb, Humid Continental warm summer	REF	A horizon	19.0	2.42	0.10	5.58	NA	23.78
Hemicellulose	DC545	13C	13C-OM1-Organic-DC-C1-1	IDF_BC_	British Columbia	DC	Forest Soil	Whole Community DNA	PCR	Amplicon	Pyrotag library	454 GS FLX Titanium	European Nucleotide Archive	https://www.ebi.ac.uk/ena/data/search?query=PRJEB9181	ERS713523	SAMEA3360071	ERX945192	ERR865564	16S rRNA	27F	TCTACGTAGC	AGAGTTTGATCMTGGCTCAG	6/25/2010	0.5	50.85	-120.42	CANADA	0.1	1150	2.5	300	Brunisolic Gray Luvisol	Douglas fir, Subalpine fir, Lodgepole pine	Dfb, Humid Continental warm summer	OM1	O horizon	57.7	38.70	1.31	5.57	NA	30.04
Hemicellulose	DC545	13C	13C-OM1-Organic-DC-C1-1	IDF_BC_	British Columbia	DC	Forest Soil	Whole Community DNA	PCR	Amplicon	Pyrotag library	454 GS FLX Titanium	European Nucleotide Archive	https://www.ebi.ac.uk/ena/data/search?query=PRJEB9182	ERS713550	SAMEA3360098	ERX945211	ERR865583	rRNA intergenic spacer analysis	ITS2	TACGTCATCA	TCCTCCGCTTATTGATATGC	6/25/2010	0.5	50.85	-120.42	CANADA	0.1	1150	2.5	300	Brunisolic Gray Luvisol	Douglas fir, Subalpine fir, Lodgepole pine	Dfb, Humid Continental warm summer	OM1	O horizon	57.7	38.70	1.31	5.57	NA	30.04
Hemicellulose	DC548	13C	13C-OM1-Mineral-DC-C2-1	IDF_BC_	British Columbia	DC	Forest Soil	Whole Community DNA	PCR	Amplicon	Pyrotag library	454 GS FLX Titanium	European Nucleotide Archive	https://www.ebi.ac.uk/ena/data/search?query=PRJEB9181	ERS713524	SAMEA3360072	ERX945187	ERR865559	16S rRNA	27F	AGACTATACT	AGAGTTTGATCMTGGCTCAG	6/25/2010	0.5	50.85	-120.42	CANADA	0.3	1150	2.5	300	Brunisolic Gray Luvisol	Douglas fir, Subalpine fir, Lodgepole pine	Dfb, Humid Continental warm summer	OM1	A horizon	27.7	2.25	0.11	5.16	NA	20.61
Hemicellulose	DC575	13C	13C-OM0-Organic-DC-C3-1	IDF_BC_	British Columbia	DC	Forest Soil	Whole Community DNA	PCR	Amplicon	Pyrotag library	454 GS FLX Titanium	European Nucleotide Archive	https://www.ebi.ac.uk/ena/data/search?query=PRJEB9181	ERS713525	SAMEA3360073	ERX945183	ERR865555	16S rRNA	27F	AGTACGCTAT	AGAGTTTGATCMTGGCTCAG	6/25/2010	0.5	50.85	-120.42	CANADA	0.1	1150	2.5	300	Brunisolic Gray Luvisol	Douglas fir, Subalpine fir, Lodgepole pine	Dfb, Humid Continental warm summer	REF	O horizon	58.3	44.30	1.07	5.00	NA	41.53
Hemicellulose	DC575	13C	13C-OM0-Organic-DC-C3-1	IDF_BC_	British Columbia	DC	Forest Soil	Whole Community DNA	PCR	Amplicon	Pyrotag library	454 GS FLX Titanium	European Nucleotide Archive	https://www.ebi.ac.uk/ena/data/search?query=PRJEB9182	ERS713542	SAMEA3360090	ERX945203	ERR865575	rRNA intergenic spacer analysis	ITS2	TCACGCGAGA	TCCTCCGCTTATTGATATGC	6/25/2010	0.5	50.85	-120.42	CANADA	0.1	1150	2.5	300	Brunisolic Gray Luvisol	Douglas fir, Subalpine fir, Lodgepole pine	Dfb, Humid Continental warm summer	REF	O horizon	58.3	44.30	1.07	5.00	NA	41.53
Hemicellulose	DC578	13C	13C-OM0-Mineral-DC-C4-1	IDF_BC_	British Columbia	DC	Forest Soil	Whole Community DNA	PCR	Amplicon	Pyrotag library	454 GS FLX Titanium	European Nucleotide Archive	https://www.ebi.ac.uk/ena/data/search?query=PRJEB9181	ERS713527	SAMEA3360075	ERX945180	ERR865552	16S rRNA	27F	CACGCTACGT	AGAGTTTGATCMTGGCTCAG	6/25/2010	0.5	50.85	-120.42	CANADA	0.3	1150	2.5	300	Brunisolic Gray Luvisol	Douglas fir, Subalpine fir, Lodgepole pine	Dfb, Humid Continental warm summer	REF	A horizon	19.0	2.34	0.10	5.17	NA	23.60
Hemicellulose	DC578	13C	13C-OM0-Mineral-DC-C4-1	IDF_BC_	British Columbia	DC	Forest Soil	Whole Community DNA	PCR	Amplicon	Pyrotag library	454 GS FLX Titanium	European Nucleotide Archive	https://www.ebi.ac.uk/ena/data/search?query=PRJEB9182	ERS713535	SAMEA3360083	ERX945196	ERR865568	rRNA intergenic spacer analysis	ITS2	TCGCTGCGTA	TCCTCCGCTTATTGATATGC	6/25/2010	0.5	50.85	-120.42	CANADA	0.3	1150	2.5	300	Brunisolic Gray Luvisol	Douglas fir, Subalpine fir, Lodgepole pine	Dfb, Humid Continental warm summer	REF	A horizon	19.0	2.34	0.10	5.17	NA	23.60
Hemicellulose	OC407	13C	13C-OM1-Organic-OC-C1-1	IDF_BC_	British Columbia	OC	Forest Soil	Whole Community DNA	PCR	Amplicon	Pyrotag library	454 GS FLX Titanium	European Nucleotide Archive	https://www.ebi.ac.uk/ena/data/search?query=PRJEB9181	ERS713509	SAMEA3360057	ERX945193	ERR865565	16S rRNA	27F	ACGCTCGACA	AGAGTTTGATCMTGGCTCAG	6/26/2010	0.5	50.88	-120.35	CANADA	0.1	1075	2.5	300	Brunisolic Gray Luvisol	Douglas fir	Dfb, Humid Continental warm summer	OM1	O horizon	50.0	33.83	1.41	5.50	NA	24.05
Hemicellulose	OC407	13C	13C-OM1-Organic-OC-C1-1	IDF_BC_	British Columbia	OC	Forest Soil	Whole Community DNA	PCR	Amplicon	Pyrotag library	454 GS FLX Titanium	European Nucleotide Archive	https://www.ebi.ac.uk/ena/data/search?query=PRJEB9182	ERS713552	SAMEA3360100	ERX945213	ERR865585	rRNA intergenic spacer analysis	ITS2	TGACGTATGT	TCCTCCGCTTATTGATATGC	6/26/2010	0.5	50.88	-120.35	CANADA	0.1	1075	2.5	300	Brunisolic Gray Luvisol	Douglas fir	Dfb, Humid Continental warm summer	OM1	O horizon	50.0	33.83	1.41	5.50	NA	24.05
Hemicellulose	OC410	13C	13C-OM1-Mineral-OC-C2-1	IDF_BC_	British Columbia	OC	Forest Soil	Whole Community DNA	PCR	Amplicon	Pyrotag library	454 GS FLX Titanium	European Nucleotide Archive	https://www.ebi.ac.uk/ena/data/search?query=PRJEB9181	ERS713511	SAMEA3360059	ERX945188	ERR865560	16S rRNA	27F	AGCACTGTAG	AGAGTTTGATCMTGGCTCAG	6/26/2010	0.5	50.88	-120.35	CANADA	0.3	1075	2.5	300	Brunisolic Gray Luvisol	Douglas fir	Dfb, Humid Continental warm summer	OM1	A horizon	23.0	1.79	0.11	5.62	NA	15.92
Hemicellulose	OC410	13C	13C-OM1-Mineral-OC-C2-1	IDF_BC_	British Columbia	OC	Forest Soil	Whole Community DNA	PCR	Amplicon	Pyrotag library	454 GS FLX Titanium	European Nucleotide Archive	https://www.ebi.ac.uk/ena/data/search?query=PRJEB9182	ERS713546	SAMEA3360094	ERX945207	ERR865579	rRNA intergenic spacer analysis	ITS2	ACAGTATATA	TCCTCCGCTTATTGATATGC	6/26/2010	0.5	50.88	-120.35	CANADA	0.3	1075	2.5	300	Brunisolic Gray Luvisol	Douglas fir	Dfb, Humid Continental warm summer	OM1	A horizon	23.0	1.79	0.11	5.62	NA	15.92
Hemicellulose	OC455	13C	13C-OM0-Organic-OC-C3-1	IDF_BC_	British Columbia	OC	Forest Soil	Whole Community DNA	PCR	Amplicon	Pyrotag library	454 GS FLX Titanium	European Nucleotide Archive	https://www.ebi.ac.uk/ena/data/search?query=PRJEB9181	ERS713513	SAMEA3360061	ERX945184	ERR865556	16S rRNA	27F	ATATCGCGAG	AGAGTTTGATCMTGGCTCAG	6/26/2010	0.5	50.88	-120.35	CANADA	0.1	1075	2.5	300	Brunisolic Gray Luvisol	Douglas fir	Dfb, Humid Continental warm summer	REF	O horizon	37.0	44.18	1.39	5.41	NA	31.71
Hemicellulose	OC455	13C	13C-OM0-Organic-OC-C3-1	IDF_BC_	British Columbia	OC	Forest Soil	Whole Community DNA	PCR	Amplicon	Pyrotag library	454 GS FLX Titanium	European Nucleotide Archive	https://www.ebi.ac.uk/ena/data/search?query=PRJEB9182	ERS713543	SAMEA3360091	ERX945204	ERR865576	rRNA intergenic spacer analysis	ITS2	ACTAGCAGTA	TCCTCCGCTTATTGATATGC	6/26/2010	0.5	50.88	-120.35	CANADA	0.1	1075	2.5	300	Brunisolic Gray Luvisol	Douglas fir	Dfb, Humid Continental warm summer	REF	O horizon	37.0	44.18	1.39	5.41	NA	31.71
Hemicellulose	OC458	13C	13C-OM0-Mineral-OC-C4-1	IDF_BC_	British Columbia	OC	Forest Soil	Whole Community DNA	PCR	Amplicon	Pyrotag library	454 GS FLX Titanium	European Nucleotide Archive	https://www.ebi.ac.uk/ena/data/search?query=PRJEB9181	ERS713514	SAMEA3360062	ERX945181	ERR865553	16S rRNA	27F	CTCGCGTGTC	AGAGTTTGATCMTGGCTCAG	6/26/2010	0.5	50.88	-120.35	CANADA	0.3	1075	2.5	300	Brunisolic Gray Luvisol	Douglas fir	Dfb, Humid Continental warm summer	REF	A horizon	13.0	1.81	0.10	5.68	NA	18.68
Hemicellulose	OC458	13C	13C-OM0-Mineral-OC-C4-1	IDF_BC_	British Columbia	OC	Forest Soil	Whole Community DNA	PCR	Amplicon	Pyrotag library	454 GS FLX Titanium	European Nucleotide Archive	https://www.ebi.ac.uk/ena/data/search?query=PRJEB9182	ERS713536	SAMEA3360084	ERX945197	ERR865569	rRNA intergenic spacer analysis	ITS2	AGTATACATA	TCCTCCGCTTATTGATATGC	6/26/2010	0.5	50.88	-120.35	CANADA	0.3	1075	2.5	300	Brunisolic Gray Luvisol	Douglas fir	Dfb, Humid Continental warm summer	REF	A horizon	13.0	1.81	0.10	5.68	NA	18.68
Hemicellulose	BL029	12C	12C-OM1-Organic-BL-029-1	PP_CA_	California	BL	Forest Soil	Whole Community DNA	PCR	Amplicon	Pyrotag library	454 GS FLX Titanium	European Nucleotide Archive	https://www.ebi.ac.uk/ena/data/search?query=PRJEB9181	ERS713528	SAMEA3360076	ERX945176	ERR865548	16S rRNA	27F	ACGCGAGTAT	AGAGTTTGATCMTGGCTCAG	9/16/2011	0.5	38.88	-120.64	USA	0.1	1350	11.2	55	Mesic Ultic Haploxeralfs	Ponderosa pine, sugar pine, white fir, giant sequoia	Csa, Mediterranean hot summer	OM1	O horizon	18.9	NA	NA	4.69	NA	NA
Hemicellulose	BL029	12C	12C-OM1-Organic-BL-029-1	PP_CA_	California	BL	Forest Soil	Whole Community DNA	PCR	Amplicon	Pyrotag library	454 GS FLX Titanium	European Nucleotide Archive	https://www.ebi.ac.uk/ena/data/search?query=PRJEB9182	ERS713534	SAMEA3360082	ERX945195	ERR865567	rRNA intergenic spacer analysis	ITS2	TCTGACGTCA	TCCTCCGCTTATTGATATGC	9/16/2011	0.5	38.88	-120.64	USA	0.1	1350	11.2	55	Mesic Ultic Haploxeralfs	Ponderosa pine, sugar pine, white fir, giant sequoia	Csa, Mediterranean hot summer	OM1	O horizon	18.9	NA	NA	4.69	NA	NA
Hemicellulose	BL030	12C	12C-OM1-Mineral-BL-030-2	PP_CA_	California	BL	Forest Soil	Whole Community DNA	PCR	Amplicon	Pyrotag library	454 GS FLX Titanium	European Nucleotide Archive	https://www.ebi.ac.uk/ena/data/search?query=PRJEB9181	ERS713483	SAMEA3360031	ERX945146	ERR865518	16S rRNA	27F	CACGCTACGT	AGAGTTTGATCMTGGCTCAG	9/16/2011	0.5	38.88	-120.64	USA	0.3	1350	11.2	55	Mesic Ultic Haploxeralfs	Ponderosa pine, sugar pine, white fir, giant sequoia	Csa, Mediterranean hot summer	OM1	A horizon	23.7	5.47	0.28	5.89	NA	19.78
Hemicellulose	BL030	12C	12C-OM1-Mineral-BL-030-1	PP_CA_	California	BL	Forest Soil	Whole Community DNA	PCR	Amplicon	Pyrotag library	454 GS FLX Titanium	European Nucleotide Archive	https://www.ebi.ac.uk/ena/data/search?query=PRJEB9181	ERS713531	SAMEA3360079	ERX945173	ERR865545	16S rRNA	27F	TACACACACT	AGAGTTTGATCMTGGCTCAG	9/16/2011	0.5	38.88	-120.64	USA	0.3	1350	11.2	55	Mesic Ultic Haploxeralfs	Ponderosa pine, sugar pine, white fir, giant sequoia	Csa, Mediterranean hot summer	OM1	A horizon	23.7	5.47	0.28	5.89	NA	19.78
Hemicellulose	BL030	12C	12C-OM1-Mineral-BL-030-1	PP_CA_	California	BL	Forest Soil	Whole Community DNA	PCR	Amplicon	Pyrotag library	454 GS FLX Titanium	European Nucleotide Archive	https://www.ebi.ac.uk/ena/data/search?query=PRJEB9182	ERS713533	SAMEA3360081	ERX945194	ERR865566	rRNA intergenic spacer analysis	ITS2	TGTCGTCGCA	TCCTCCGCTTATTGATATGC	9/16/2011	0.5	38.88	-120.64	USA	0.3	1350	11.2	55	Mesic Ultic Haploxeralfs	Ponderosa pine, sugar pine, white fir, giant sequoia	Csa, Mediterranean hot summer	OM1	A horizon	23.7	5.47	0.28	5.89	NA	19.78
Hemicellulose	BL045	12C	12C-OM0-Organic-BL-045-1	PP_CA_	California	BL	Forest Soil	Whole Community DNA	PCR	Amplicon	Pyrotag library	454 GS FLX Titanium	European Nucleotide Archive	https://www.ebi.ac.uk/ena/data/search?query=PRJEB9181	ERS713504	SAMEA3360052	ERX945145	ERR865517	16S rRNA	27F	TCTAGCGACT	AGAGTTTGATCMTGGCTCAG	9/16/2011	0.5	38.88	-120.64	USA	0.1	1350	11.2	55	Mesic Ultic Haploxeralfs	Ponderosa pine, sugar pine, white fir, giant sequoia	Csa, Mediterranean hot summer	REF	O horizon	25.8	NA	NA	5.28	NA	NA
Hemicellulose	BL046	12C	12C-OM0-Mineral-BL-046-1	PP_CA_	California	BL	Forest Soil	Whole Community DNA	PCR	Amplicon	Pyrotag library	454 GS FLX Titanium	European Nucleotide Archive	https://www.ebi.ac.uk/ena/data/search?query=PRJEB9181	ERS713493	SAMEA3360041	ERX945144	ERR865516	16S rRNA	27F	TACAGATCGT	AGAGTTTGATCMTGGCTCAG	9/16/2011	0.5	38.88	-120.64	USA	0.3	1350	11.2	55	Mesic Ultic Haploxeralfs	Ponderosa pine, sugar pine, white fir, giant sequoia	Csa, Mediterranean hot summer	REF	A horizon	17.5	6.30	0.31	5.49	NA	20.32
Hemicellulose	BR049	12C	12C-OM1-Organic-BR-C2-1	PP_CA_	California	BR	Forest Soil	Whole Community DNA	PCR	Amplicon	Pyrotag library	454 GS FLX Titanium	European Nucleotide Archive	https://www.ebi.ac.uk/ena/data/search?query=PRJEB9181	ERS713486	SAMEA3360034	ERX945147	ERR865519	16S rRNA	27F	AGACTATACT	AGAGTTTGATCMTGGCTCAG	6/22/2011	0.5	39.55	-121.04	USA	0.1	1135	11.2	55	Mesic Ultic Haploxeralfs	Ponderosa pine, sugar pine, white fir, giant sequoia	Csa, Mediterranean hot summer	OM1	O horizon	54.2	41.60	1.63	5.39	NA	25.48
Hemicellulose	LH001	12C	12C-OM1-Organic-LH-C1-1	PP_CA_	California	LH	Forest Soil	Whole Community DNA	PCR	Amplicon	Pyrotag library	454 GS FLX Titanium	European Nucleotide Archive	https://www.ebi.ac.uk/ena/data/search?query=PRJEB9181	ERS713499	SAMEA3360047	ERX945148	ERR865520	16S rRNA	27F	CAGTAGACGT	AGAGTTTGATCMTGGCTCAG	9/16/2011	0.5	39.26	-120.78	USA	0.1	1268	11.2	55	Mesic Ultic Haploxeralfs	Ponderosa pine, sugar pine, white fir, giant sequoia	Csa, Mediterranean hot summer	OM1	O horizon	14.4	44.78	1.01	3.67	NA	44.36
Hemicellulose	BL026	13C	13C-OM1-Mineral-BL-026-2	PP_CA_	California	BL	Forest Soil	Whole Community DNA	PCR	Amplicon	Pyrotag library	454 GS FLX Titanium	European Nucleotide Archive	https://www.ebi.ac.uk/ena/data/search?query=PRJEB9181	ERS713496	SAMEA3360044	ERX945159	ERR865531	16S rRNA	27F	CGAGAGATAC	AGAGTTTGATCMTGGCTCAG	9/16/2011	0.5	38.88	-120.64	USA	0.3	1350	11.2	55	Mesic Ultic Haploxeralfs	Ponderosa pine, sugar pine, white fir, giant sequoia	Csa, Mediterranean hot summer	OM1	A horizon	22.0	5.47	0.28	5.74	NA	19.78
Hemicellulose	BL027	13C	13C-OM1-Organic-BL-027-1	PP_CA_	California	BL	Forest Soil	Whole Community DNA	PCR	Amplicon	Pyrotag library	454 GS FLX Titanium	European Nucleotide Archive	https://www.ebi.ac.uk/ena/data/search?query=PRJEB9181	ERS713530	SAMEA3360078	ERX945189	ERR865561	16S rRNA	27F	CGACGTGACT	AGAGTTTGATCMTGGCTCAG	9/16/2011	0.5	38.88	-120.64	USA	0.1	1350	11.2	55	Mesic Ultic Haploxeralfs	Ponderosa pine, sugar pine, white fir, giant sequoia	Csa, Mediterranean hot summer	OM1	O horizon	16.7	NA	NA	4.90	NA	NA
Hemicellulose	BL027	13C	13C-OM1-Organic-BL-027-1	PP_CA_	California	BL	Forest Soil	Whole Community DNA	PCR	Amplicon	Pyrotag library	454 GS FLX Titanium	European Nucleotide Archive	https://www.ebi.ac.uk/ena/data/search?query=PRJEB9182	ERS713547	SAMEA3360095	ERX945208	ERR865580	rRNA intergenic spacer analysis	ITS2	TGTCACACGA	TCCTCCGCTTATTGATATGC	9/16/2011	0.5	38.88	-120.64	USA	0.1	1350	11.2	55	Mesic Ultic Haploxeralfs	Ponderosa pine, sugar pine, white fir, giant sequoia	Csa, Mediterranean hot summer	OM1	O horizon	16.7	NA	NA	4.90	NA	NA
Hemicellulose	BL028	13C	13C-OM1-Mineral-BL-028-2	PP_CA_	California	BL	Forest Soil	Whole Community DNA	PCR	Amplicon	Pyrotag library	454 GS FLX Titanium	European Nucleotide Archive	https://www.ebi.ac.uk/ena/data/search?query=PRJEB9181	ERS713497	SAMEA3360045	ERX945160	ERR865532	16S rRNA	27F	TCACGTACTA	AGAGTTTGATCMTGGCTCAG	9/16/2011	0.5	38.88	-120.64	USA	0.3	1350	11.2	55	Mesic Ultic Haploxeralfs	Ponderosa pine, sugar pine, white fir, giant sequoia	Csa, Mediterranean hot summer	OM1	A horizon	21.0	5.47	0.28	5.87	NA	19.78
Hemicellulose	BL029	13C	13C-OM1-Organic-BL-029-1	PP_CA_	California	BL	Forest Soil	Whole Community DNA	PCR	Amplicon	Pyrotag library	454 GS FLX Titanium	European Nucleotide Archive	https://www.ebi.ac.uk/ena/data/search?query=PRJEB9181	ERS713529	SAMEA3360077	ERX945190	ERR865562	16S rRNA	27F	ACTACTATGT	AGAGTTTGATCMTGGCTCAG	9/16/2011	0.5	38.88	-120.64	USA	0.1	1350	11.2	55	Mesic Ultic Haploxeralfs	Ponderosa pine, sugar pine, white fir, giant sequoia	Csa, Mediterranean hot summer	OM1	O horizon	18.9	NA	NA	4.69	NA	NA
Hemicellulose	BL029	13C	13C-OM1-Organic-BL-029-1	PP_CA_	California	BL	Forest Soil	Whole Community DNA	PCR	Amplicon	Pyrotag library	454 GS FLX Titanium	European Nucleotide Archive	https://www.ebi.ac.uk/ena/data/search?query=PRJEB9182	ERS713548	SAMEA3360096	ERX945209	ERR865581	rRNA intergenic spacer analysis	ITS2	TGAGTCAGTA	TCCTCCGCTTATTGATATGC	9/16/2011	0.5	38.88	-120.64	USA	0.1	1350	11.2	55	Mesic Ultic Haploxeralfs	Ponderosa pine, sugar pine, white fir, giant sequoia	Csa, Mediterranean hot summer	OM1	O horizon	18.9	NA	NA	4.69	NA	NA
Hemicellulose	BL030	13C	13C-OM1-Mineral-BL-030-2	PP_CA_	California	BL	Forest Soil	Whole Community DNA	PCR	Amplicon	Pyrotag library	454 GS FLX Titanium	European Nucleotide Archive	https://www.ebi.ac.uk/ena/data/search?query=PRJEB9181	ERS713484	SAMEA3360032	ERX945161	ERR865533	16S rRNA	27F	ATAGAGTACT	AGAGTTTGATCMTGGCTCAG	9/16/2011	0.5	38.88	-120.64	USA	0.3	1350	11.2	55	Mesic Ultic Haploxeralfs	Ponderosa pine, sugar pine, white fir, giant sequoia	Csa, Mediterranean hot summer	OM1	A horizon	23.7	5.47	0.28	5.89	NA	19.78
Hemicellulose	BL030	13C	13C-OM1-Mineral-BL-030-1	PP_CA_	California	BL	Forest Soil	Whole Community DNA	PCR	Amplicon	Pyrotag library	454 GS FLX Titanium	European Nucleotide Archive	https://www.ebi.ac.uk/ena/data/search?query=PRJEB9181	ERS713532	SAMEA3360080	ERX945185	ERR865557	16S rRNA	27F	TACACGTGAT	AGAGTTTGATCMTGGCTCAG	9/16/2011	0.5	38.88	-120.64	USA	0.3	1350	11.2	55	Mesic Ultic Haploxeralfs	Ponderosa pine, sugar pine, white fir, giant sequoia	Csa, Mediterranean hot summer	OM1	A horizon	23.7	5.47	0.28	5.89	NA	19.78
Hemicellulose	BL030	13C	13C-OM1-Mineral-BL-030-1	PP_CA_	California	BL	Forest Soil	Whole Community DNA	PCR	Amplicon	Pyrotag library	454 GS FLX Titanium	European Nucleotide Archive	https://www.ebi.ac.uk/ena/data/search?query=PRJEB9182	ERS713544	SAMEA3360092	ERX945205	ERR865577	rRNA intergenic spacer analysis	ITS2	ACACATACGC	TCCTCCGCTTATTGATATGC	9/16/2011	0.5	38.88	-120.64	USA	0.3	1350	11.2	55	Mesic Ultic Haploxeralfs	Ponderosa pine, sugar pine, white fir, giant sequoia	Csa, Mediterranean hot summer	OM1	A horizon	23.7	5.47	0.28	5.89	NA	19.78
Hemicellulose	BL030	13C	13C-OM1-Mineral-BL-030-2	PP_CA_	California	BL	Forest Soil	Whole Community DNA	PCR	Amplicon	Pyrotag library	454 GS FLX Titanium	European Nucleotide Archive	https://www.ebi.ac.uk/ena/data/search?query=PRJEB9182	ERS713545	SAMEA3360093	ERX945206	ERR865578	rRNA intergenic spacer analysis	ITS2	TACTCTCGTG	TCCTCCGCTTATTGATATGC	9/16/2011	0.5	38.88	-120.64	USA	0.3	1350	11.2	55	Mesic Ultic Haploxeralfs	Ponderosa pine, sugar pine, white fir, giant sequoia	Csa, Mediterranean hot summer	OM1	A horizon	23.7	5.47	0.28	5.89	NA	19.78
Hemicellulose	BL038	13C	13C-OM3-Mineral-BL-038-1	PP_CA_	California	BL	Forest Soil	Whole Community DNA	PCR	Amplicon	Pyrotag library	454 GS FLX Titanium	European Nucleotide Archive	https://www.ebi.ac.uk/ena/data/search?query=PRJEB9181	ERS713489	SAMEA3360037	ERX945166	ERR865538	16S rRNA	27F	ACGCTCGACA	AGAGTTTGATCMTGGCTCAG	9/16/2011	0.5	38.88	-120.64	USA	0.3	1350	11.2	55	Mesic Ultic Haploxeralfs	Ponderosa pine, sugar pine, white fir, giant sequoia	Csa, Mediterranean hot summer	OM3	A horizon	21.7	5.27	0.28	5.42	NA	18.87
Hemicellulose	BL040	13C	13C-OM3-Mineral-BL-040-1	PP_CA_	California	BL	Forest Soil	Whole Community DNA	PCR	Amplicon	Pyrotag library	454 GS FLX Titanium	European Nucleotide Archive	https://www.ebi.ac.uk/ena/data/search?query=PRJEB9181	ERS713490	SAMEA3360038	ERX945167	ERR865539	16S rRNA	27F	AGCACTGTAG	AGAGTTTGATCMTGGCTCAG	9/16/2011	0.5	38.88	-120.64	USA	0.3	1350	11.2	55	Mesic Ultic Haploxeralfs	Ponderosa pine, sugar pine, white fir, giant sequoia	Csa, Mediterranean hot summer	OM3	A horizon	22.2	5.27	0.28	5.86	NA	18.87
Hemicellulose	BL042	13C	13C-OM3-Mineral-BL-042-1	PP_CA_	California	BL	Forest Soil	Whole Community DNA	PCR	Amplicon	Pyrotag library	454 GS FLX Titanium	European Nucleotide Archive	https://www.ebi.ac.uk/ena/data/search?query=PRJEB9181	ERS713491	SAMEA3360039	ERX945168	ERR865540	16S rRNA	27F	ATATCGCGAG	AGAGTTTGATCMTGGCTCAG	9/16/2011	0.5	38.88	-120.64	USA	0.3	1350	11.2	55	Mesic Ultic Haploxeralfs	Ponderosa pine, sugar pine, white fir, giant sequoia	Csa, Mediterranean hot summer	OM3	A horizon	21.5	5.27	0.28	5.39	NA	18.87
Hemicellulose	BL044	13C	13C-OM0-Mineral-BL-044-1	PP_CA_	California	BL	Forest Soil	Whole Community DNA	PCR	Amplicon	Pyrotag library	454 GS FLX Titanium	European Nucleotide Archive	https://www.ebi.ac.uk/ena/data/search?query=PRJEB9181	ERS713492	SAMEA3360040	ERX945149	ERR865521	16S rRNA	27F	CTCGCGTGTC	AGAGTTTGATCMTGGCTCAG	9/16/2011	0.5	38.88	-120.64	USA	0.3	1350	11.2	55	Mesic Ultic Haploxeralfs	Ponderosa pine, sugar pine, white fir, giant sequoia	Csa, Mediterranean hot summer	REF	A horizon	17.8	6.30	0.31	5.48	NA	20.32
Hemicellulose	BL045	13C	13C-OM0-Organic-BL-045-1	PP_CA_	California	BL	Forest Soil	Whole Community DNA	PCR	Amplicon	Pyrotag library	454 GS FLX Titanium	European Nucleotide Archive	https://www.ebi.ac.uk/ena/data/search?query=PRJEB9181	ERS713505	SAMEA3360053	ERX945154	ERR865526	16S rRNA	27F	TCTATACTAT	AGAGTTTGATCMTGGCTCAG	9/16/2011	0.5	38.88	-120.64	USA	0.1	1350	11.2	55	Mesic Ultic Haploxeralfs	Ponderosa pine, sugar pine, white fir, giant sequoia	Csa, Mediterranean hot summer	REF	O horizon	25.8	NA	NA	5.28	NA	NA
Hemicellulose	BL045	13C	13C-OM0-Organic-BL-045-2	PP_CA_	California	BL	Forest Soil	Whole Community DNA	PCR	Amplicon	Pyrotag library	454 GS FLX Titanium	European Nucleotide Archive	https://www.ebi.ac.uk/ena/data/search?query=PRJEB9181	ERS713506	SAMEA3360054	ERX945155	ERR865527	16S rRNA	27F	TGTGAGTAGT	AGAGTTTGATCMTGGCTCAG	9/16/2011	0.5	38.88	-120.64	USA	0.1	1350	11.2	55	Mesic Ultic Haploxeralfs	Ponderosa pine, sugar pine, white fir, giant sequoia	Csa, Mediterranean hot summer	REF	O horizon	25.8	NA	NA	5.28	NA	NA
Hemicellulose	BL045	13C	13C-OM0-Organic-BL-045-1	PP_CA_	California	BL	Forest Soil	Whole Community DNA	PCR	Amplicon	Pyrotag library	454 GS FLX Titanium	European Nucleotide Archive	https://www.ebi.ac.uk/ena/data/search?query=PRJEB9182	ERS713537	SAMEA3360085	ERX945198	ERR865570	rRNA intergenic spacer analysis	ITS2	TACACGTGAT	TCCTCCGCTTATTGATATGC	9/16/2011	0.5	38.88	-120.64	USA	0.1	1350	11.2	55	Mesic Ultic Haploxeralfs	Ponderosa pine, sugar pine, white fir, giant sequoia	Csa, Mediterranean hot summer	REF	O horizon	25.8	NA	NA	5.28	NA	NA
Hemicellulose	BL045	13C	13C-OM0-Organic-BL-045-2	PP_CA_	California	BL	Forest Soil	Whole Community DNA	PCR	Amplicon	Pyrotag library	454 GS FLX Titanium	European Nucleotide Archive	https://www.ebi.ac.uk/ena/data/search?query=PRJEB9182	ERS713538	SAMEA3360086	ERX945199	ERR865571	rRNA intergenic spacer analysis	ITS2	TACGCTGTCT	TCCTCCGCTTATTGATATGC	9/16/2011	0.5	38.88	-120.64	USA	0.1	1350	11.2	55	Mesic Ultic Haploxeralfs	Ponderosa pine, sugar pine, white fir, giant sequoia	Csa, Mediterranean hot summer	REF	O horizon	25.8	NA	NA	5.28	NA	NA
Hemicellulose	BL046	13C	13C-OM0-Mineral-BL-046-1	PP_CA_	California	BL	Forest Soil	Whole Community DNA	PCR	Amplicon	Pyrotag library	454 GS FLX Titanium	European Nucleotide Archive	https://www.ebi.ac.uk/ena/data/search?query=PRJEB9181	ERS713494	SAMEA3360042	ERX945150	ERR865522	16S rRNA	27F	TCTCTATGCG	AGAGTTTGATCMTGGCTCAG	9/16/2011	0.5	38.88	-120.64	USA	0.3	1350	11.2	55	Mesic Ultic Haploxeralfs	Ponderosa pine, sugar pine, white fir, giant sequoia	Csa, Mediterranean hot summer	REF	A horizon	17.5	6.30	0.31	5.49	NA	20.32
Hemicellulose	BL047	13C	13C-OM0-Organic-BL-047-1	PP_CA_	California	BL	Forest Soil	Whole Community DNA	PCR	Amplicon	Pyrotag library	454 GS FLX Titanium	European Nucleotide Archive	https://www.ebi.ac.uk/ena/data/search?query=PRJEB9181	ERS713507	SAMEA3360055	ERX945156	ERR865528	16S rRNA	27F	ACGCGATCGA	AGAGTTTGATCMTGGCTCAG	9/16/2011	0.5	38.88	-120.64	USA	0.1	1350	11.2	55	Mesic Ultic Haploxeralfs	Ponderosa pine, sugar pine, white fir, giant sequoia	Csa, Mediterranean hot summer	REF	O horizon	29.8	NA	NA	4.90	NA	NA
Hemicellulose	BL047	13C	13C-OM0-Organic-BL-047-1	PP_CA_	California	BL	Forest Soil	Whole Community DNA	PCR	Amplicon	Pyrotag library	454 GS FLX Titanium	European Nucleotide Archive	https://www.ebi.ac.uk/ena/data/search?query=PRJEB9182	ERS713539	SAMEA3360087	ERX945200	ERR865572	rRNA intergenic spacer analysis	ITS2	TCGATCACGT	TCCTCCGCTTATTGATATGC	9/16/2011	0.5	38.88	-120.64	USA	0.1	1350	11.2	55	Mesic Ultic Haploxeralfs	Ponderosa pine, sugar pine, white fir, giant sequoia	Csa, Mediterranean hot summer	REF	O horizon	29.8	NA	NA	4.90	NA	NA
Hemicellulose	BL048	13C	13C-OM0-Mineral-BL-048-1	PP_CA_	California	BL	Forest Soil	Whole Community DNA	PCR	Amplicon	Pyrotag library	454 GS FLX Titanium	European Nucleotide Archive	https://www.ebi.ac.uk/ena/data/search?query=PRJEB9181	ERS713495	SAMEA3360043	ERX945151	ERR865523	16S rRNA	27F	ACTAGCAGTA	AGAGTTTGATCMTGGCTCAG	9/16/2011	0.5	38.88	-120.64	USA	0.3	1350	11.2	55	Mesic Ultic Haploxeralfs	Ponderosa pine, sugar pine, white fir, giant sequoia	Csa, Mediterranean hot summer	REF	A horizon	15.3	6.30	0.31	5.99	NA	20.32
Hemicellulose	BR049	13C	13C-OM1-Organic-BR-C2-1	PP_CA_	California	BR	Forest Soil	Whole Community DNA	PCR	Amplicon	Pyrotag library	454 GS FLX Titanium	European Nucleotide Archive	https://www.ebi.ac.uk/ena/data/search?query=PRJEB9181	ERS713487	SAMEA3360035	ERX945164	ERR865536	16S rRNA	27F	ACTGTACAGT	AGAGTTTGATCMTGGCTCAG	6/22/2011	0.5	39.55	-121.04	USA	0.1	1135	11.2	55	Mesic Ultic Haploxeralfs	Ponderosa pine, sugar pine, white fir, giant sequoia	Csa, Mediterranean hot summer	OM1	O horizon	54.2	41.60	1.63	5.39	NA	25.48
Hemicellulose	BR049	13C	13C-OM1-Organic-BR-C2-1	PP_CA_	California	BR	Forest Soil	Whole Community DNA	PCR	Amplicon	Pyrotag library	454 GS FLX Titanium	European Nucleotide Archive	https://www.ebi.ac.uk/ena/data/search?query=PRJEB9182	ERS713549	SAMEA3360097	ERX945210	ERR865582	rRNA intergenic spacer analysis	ITS2	TGTACTACTC	TCCTCCGCTTATTGATATGC	6/22/2011	0.5	39.55	-121.04	USA	0.1	1135	11.2	55	Mesic Ultic Haploxeralfs	Ponderosa pine, sugar pine, white fir, giant sequoia	Csa, Mediterranean hot summer	OM1	O horizon	54.2	41.60	1.63	5.39	NA	25.48
Hemicellulose	BR050	13C	13C-OM1-Mineral-BR-C1-1	PP_CA_	California	BR	Forest Soil	Whole Community DNA	PCR	Amplicon	Pyrotag library	454 GS FLX Titanium	European Nucleotide Archive	https://www.ebi.ac.uk/ena/data/search?query=PRJEB9181	ERS713485	SAMEA3360033	ERX945162	ERR865534	16S rRNA	27F	AGCGTCGTCT	AGAGTTTGATCMTGGCTCAG	6/22/2011	0.5	39.55	-121.04	USA	0.3	1135	11.2	55	Mesic Ultic Haploxeralfs	Ponderosa pine, sugar pine, white fir, giant sequoia	Csa, Mediterranean hot summer	OM1	A horizon	35.0	8.54	0.34	5.87	NA	25.34
Hemicellulose	BR067	13C	13C-OM0-Organic-BR-C4-1	PP_CA_	California	BR	Forest Soil	Whole Community DNA	PCR	Amplicon	Pyrotag library	454 GS FLX Titanium	European Nucleotide Archive	https://www.ebi.ac.uk/ena/data/search?query=PRJEB9181	ERS713498	SAMEA3360046	ERX945157	ERR865529	16S rRNA	27F	ACTACTATGT	AGAGTTTGATCMTGGCTCAG	6/22/2011	0.5	39.55	-121.04	USA	0.1	1135	11.2	55	Mesic Ultic Haploxeralfs	Ponderosa pine, sugar pine, white fir, giant sequoia	Csa, Mediterranean hot summer	REF	O horizon	60.7	39.24	1.24	4.95	NA	31.64
Hemicellulose	BR067	13C	13C-OM0-Organic-BR-C4-1	PP_CA_	California	BR	Forest Soil	Whole Community DNA	PCR	Amplicon	Pyrotag library	454 GS FLX Titanium	European Nucleotide Archive	https://www.ebi.ac.uk/ena/data/search?query=PRJEB9182	ERS713541	SAMEA3360089	ERX945202	ERR865574	rRNA intergenic spacer analysis	ITS2	ACATACGCGT	TCCTCCGCTTATTGATATGC	6/22/2011	0.5	39.55	-121.04	USA	0.1	1135	11.2	55	Mesic Ultic Haploxeralfs	Ponderosa pine, sugar pine, white fir, giant sequoia	Csa, Mediterranean hot summer	REF	O horizon	60.7	39.24	1.24	4.95	NA	31.64
Hemicellulose	BR068	13C	13C-OM0-Mineral-BR-C3-1	PP_CA_	California	BR	Forest Soil	Whole Community DNA	PCR	Amplicon	Pyrotag library	454 GS FLX Titanium	European Nucleotide Archive	https://www.ebi.ac.uk/ena/data/search?query=PRJEB9181	ERS713488	SAMEA3360036	ERX945152	ERR865524	16S rRNA	27F	CGTCTAGTAC	AGAGTTTGATCMTGGCTCAG	6/22/2011	0.5	39.55	-121.04	USA	0.3	1135	11.2	55	Mesic Ultic Haploxeralfs	Ponderosa pine, sugar pine, white fir, giant sequoia	Csa, Mediterranean hot summer	REF	A horizon	27.5	6.74	0.29	5.95	NA	23.37
Hemicellulose	LH001	13C	13C-OM1-Organic-LH-C1-1	PP_CA_	California	LH	Forest Soil	Whole Community DNA	PCR	Amplicon	Pyrotag library	454 GS FLX Titanium	European Nucleotide Archive	https://www.ebi.ac.uk/ena/data/search?query=PRJEB9181	ERS713500	SAMEA3360048	ERX945165	ERR865537	16S rRNA	27F	CGACGTGACT	AGAGTTTGATCMTGGCTCAG	9/16/2011	0.5	39.26	-120.78	USA	0.1	1268	11.2	55	Mesic Ultic Haploxeralfs	Ponderosa pine, sugar pine, white fir, giant sequoia	Csa, Mediterranean hot summer	OM1	O horizon	14.4	44.78	1.01	3.67	NA	44.36
Hemicellulose	LH001	13C	13C-OM1-Organic-LH-C1-1	PP_CA_	California	LH	Forest Soil	Whole Community DNA	PCR	Amplicon	Pyrotag library	454 GS FLX Titanium	European Nucleotide Archive	https://www.ebi.ac.uk/ena/data/search?query=PRJEB9182	ERS713551	SAMEA3360099	ERX945212	ERR865584	rRNA intergenic spacer analysis	ITS2	CTCGCGTGTC	TCCTCCGCTTATTGATATGC	9/16/2011	0.5	39.26	-120.78	USA	0.1	1268	11.2	55	Mesic Ultic Haploxeralfs	Ponderosa pine, sugar pine, white fir, giant sequoia	Csa, Mediterranean hot summer	OM1	O horizon	14.4	44.78	1.01	3.67	NA	44.36
Hemicellulose	LH002	13C	13C-OM1-Mineral-LH-C2-1	PP_CA_	California	LH	Forest Soil	Whole Community DNA	PCR	Amplicon	Pyrotag library	454 GS FLX Titanium	European Nucleotide Archive	https://www.ebi.ac.uk/ena/data/search?query=PRJEB9181	ERS713501	SAMEA3360049	ERX945163	ERR865535	16S rRNA	27F	TACACGTGAT	AGAGTTTGATCMTGGCTCAG	9/16/2011	0.5	39.26	-120.78	USA	0.3	1268	11.2	55	Mesic Ultic Haploxeralfs	Ponderosa pine, sugar pine, white fir, giant sequoia	Csa, Mediterranean hot summer	OM1	A horizon	18.5	4.78	0.16	5.18	NA	29.59
Hemicellulose	LH019	13C	13C-OM0-Organic-LH-C3-1	PP_CA_	California	LH	Forest Soil	Whole Community DNA	PCR	Amplicon	Pyrotag library	454 GS FLX Titanium	European Nucleotide Archive	https://www.ebi.ac.uk/ena/data/search?query=PRJEB9181	ERS713502	SAMEA3360050	ERX945158	ERR865530	16S rRNA	27F	TAGTGTAGAT	AGAGTTTGATCMTGGCTCAG	9/16/2011	0.5	39.26	-120.78	USA	0.1	1268	11.2	55	Mesic Ultic Haploxeralfs	Ponderosa pine, sugar pine, white fir, giant sequoia	Csa, Mediterranean hot summer	REF	O horizon	21.7	39.67	1.28	5.57	NA	30.95
Hemicellulose	LH020	13C	13C-OM0-Mineral-LH-C4-1	PP_CA_	California	LH	Forest Soil	Whole Community DNA	PCR	Amplicon	Pyrotag library	454 GS FLX Titanium	European Nucleotide Archive	https://www.ebi.ac.uk/ena/data/search?query=PRJEB9181	ERS713503	SAMEA3360051	ERX945153	ERR865525	16S rRNA	27F	TCGCACTAGT	AGAGTTTGATCMTGGCTCAG	9/16/2011	0.5	39.26	-120.78	USA	0.3	1268	11.2	55	Mesic Ultic Haploxeralfs	Ponderosa pine, sugar pine, white fir, giant sequoia	Csa, Mediterranean hot summer	REF	A horizon	20.1	4.14	0.22	6.60	NA	19.14
